# Photocatalytic remote C(sp^3^)–H alkylation of long-chain alkenes: A tandem multicomponent approach *via* radical translocation†

**DOI:** 10.1039/d5sc08683c

**Published:** 2026-01-21

**Authors:** Krishna Gopal Ghosh, Debabrata Das, Vinjamuri Srinivasu, Koustav Pal, Devarajulu Sureshkumar

**Affiliations:** a Department of Chemical Sciences, Indian Institute of Science Education and Research Kolkata Mohanpur-741246 West Bengal India suresh@iiserkol.ac.in

## Abstract

A breakthrough in organic synthesis has been accomplished by developing a redox-neutral, transition-metal-free tandem multicomponent reaction that combines perfluoroalkylation with the remote C(sp^3^)–H alkylation of terminal alkenes. This method exhibits exceptional chemo- and regioselectivity, leveraging organophotocatalysis under mild conditions. The versatility of this methodology is demonstrated through diverse 1,*n*-difunctionalized patterns (1, 6, 1, 7, 1, 11, and 1, 14) in unactivated alkenes through controlled radical translocation between secondary C(sp^3^)–carbon atoms, despite their similar bond dissociation energies (BDEs). Additionally, the study introduces a tandem multicomponent sequence encompassing the trifluoromethylation, 5-*exo-trig* cyclization, 1,5-radical translocation, and subsequent C(sp^3^)–H alkylation of 1,6-dialkenes to synthesize *trans*-1,2-disubstituted cyclopentanes. A key achievement of this work is the development of a multicomponent, transition-metal-free, tandem reaction for synthesizing highly substituted cyclopentane derivatives. This sequence involves two consecutive hydrogen atom transfer (HAT) processes: first, a 1,5-HAT from a secondary C(sp^3^)–carbon to a tertiary C(sp^3^)–carbon, followed by a 1,6-HAT from a vinylic carbon to a secondary carbon. This approach demonstrates excellent synthetic utility, as evidenced by its scalability and the facile conversion of the obtained products into diverse valuable functional groups, highlighting the broad applicability and potential of this innovative strategy.

## Abstract 2

A redox-neutral, metal-free organophotocatalytic tandem reaction enables the selective perfluoroalkylation and remote C(sp^3^)–H alkylation of alkenes, delivering diverse 1,*n*-difunctionalization and substituted cyclopentanes *via* controlled HAT.

## Graphical abstract

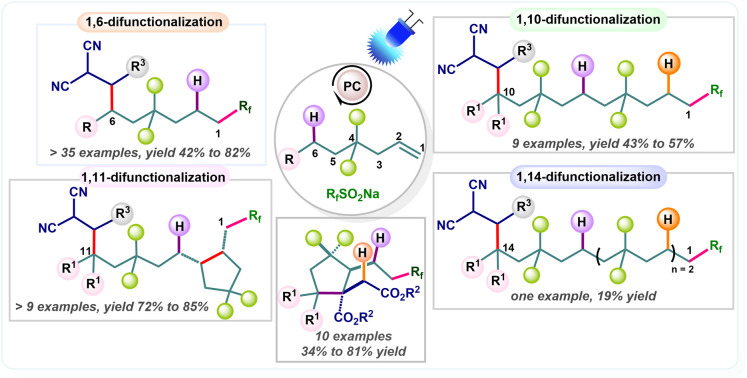

## Introduction

The efficient and selective functionalization of remote C(sp^3^)–H bonds in organic compounds has historically posed a considerable challenge, mainly due to the intrinsic chemical inertness of such bonds and the existence of numerous, ostensibly indistinguishable C–H bonds with comparable high bond dissociation energies (BDEs; generally between 90 and 100 kcal mol^−1^).^1^ This challenge has prompted the formulation of new strategies, notably intramolecular 1,*n* (*n* = 5, 6, 7) hydrogen atom transfer (HAT), which has surfaced as a potential method. This involves the homolytic cleavage of a remote, inert C(sp^3^)–H bond through a radical mechanism.

**Scheme 1:**
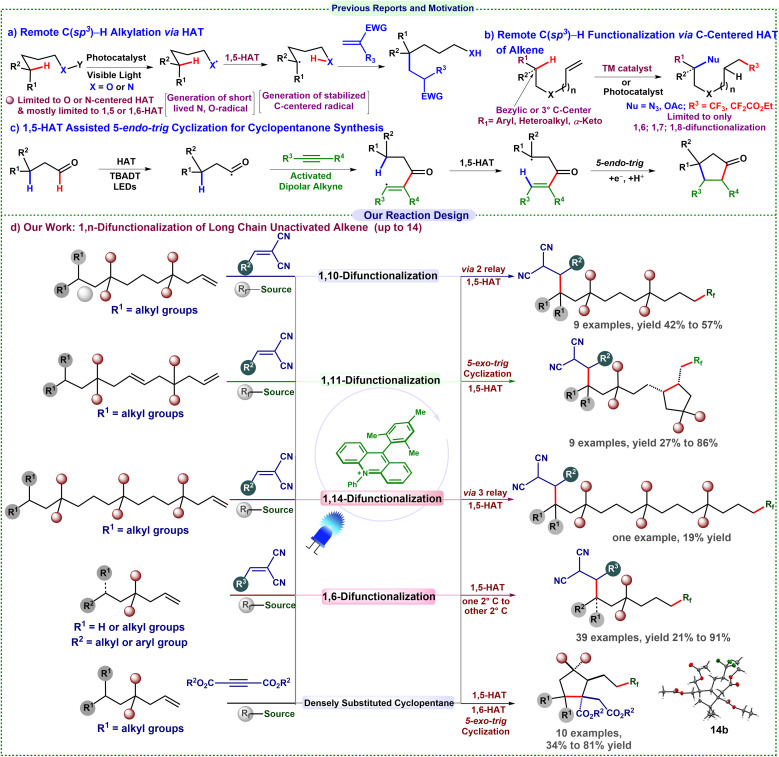
Previous reports and our reaction design.

Traditional techniques, like the Hoffman–Loffler–Freytag and Barton reactions, leveraged the elevated BDEs of N–H and O–H bonds (about 105 kcal mol^−1^),^2^ providing essential insights into this phenomenon. Recently, substantial advancements have been achieved in photocatalytic distal C(sp^3^)–H alkylation, primarily through 1,5-HAT from *N*-centered radicals, initiated by the Knowles and Rovis groups,^3^ which has broadened the synthetic toolkit (Scheme 1a). Despite these advancements, the scope of carbon-centered radical translocation has predominantly been restricted to aryl (113 kcal mol^−1^), vinylic (111 kcal mol^−1^), and primary (101 kcal mol^−1^) radicals.^4^ Selective radical translocation among C(sp^3^) centers is challenging due to their comparable BDE relative to heteroatom-mediated mechanisms.^5,6^ Furthermore, the majority of conventional methodologies have been limited to systems including *n* = 5–7 atoms (1, 5-, 1, 6-, or 1, 7-HAT). Extending these processes to longer distances (*n* ≥ 8) presents significant challenges, as managing radical migration over increased spatial separation frequently results in diminished selectivity and efficiency.^4,7^

Remote functionalization beyond *n* = 7 atoms is particularly crucial for late-stage functionalization and selective modification of substantial, biologically relevant compounds, including natural products, pharmaceuticals, and polymers.^8^ Nevertheless, very few examples are available. A significant deficiency in the existing literature concerns C(sp^3^)–H alkylation resulting from carbon-centered radical migration. Radical addition to alkene double bonds is a versatile strategy for generating carbon-centered radicals and facilitating difunctionalization of unactivated alkenes.^9^ Recent developments in remote alkene difunctionalization have predominantly concentrated on 1,6- and 1,7-difunctionalization (Scheme 1b).^4^ However, the remote 1,10-, 1,11-, and 1,14-difunctionalization of alkenes has not been investigated.

The trifluoromethyl group, acknowledged for its significance in medicinal chemistry, has been thoroughly examined in oxotrifluoromethylation, azidotrifluoromethylation, and carbotrifluoromethylation, particularly for the 1,6- and 1,7-difunctionalization of alkenes.^10^ Nonetheless, tandem trifluoromethylation succeeded by alkylation of alkenes, whether in a 1,2- or extended 1,*n*-fashion (up to 14 atoms), remains inadequately investigated. Furthermore, HAT between two secondary carbons is uncommon due to their similar reactivity and regioselectivity. Nagib and co-workers achieved a cobalt-catalyzed γ-C(sp^3^)–H functionalization of amines with a vinyl sulfonyl radical chaperone.^11^

Extending remote C(sp^3^)–H functionalization across large distances faces key challenges. (i) Selectivity: many C(sp^3^)–H sites, particularly secondary carbons, exhibit similar BDEs, complicating the selective HAT between secondary centers. (ii) Competing pathways: electrophilic or nucleophilic radicals may inadvertently interact with other π-systems or Michael acceptors, hence lowering selectivity. (iii) Side reactions: secondary carbon radicals exhibit a greater tendency for rearrangement or hydrogen abstraction, increasing by-products. (iv) Oxidation/carbocation formation: translocated radicals undergo further oxidation to carbocations, unless rapidly captured.

Most existing remote C(sp^3^)–H functionalizations use metal photocatalysts and are largely confined to proximal sites (C6 and C7). Metal-free methods are thus sought to avoid contamination and enhance the practicality of late-stage functionalization. We present a transition-metal-free, visible-light-driven method that facilitates remote 1,*n* (*n* = 14, 11, 10, 7, and 6) trifluoromethylation, succeeded by distal alkylation of alkenes, effectively addressing these challenges.

Selectivity arises from the electron-withdrawing CF_3_ group, which diminishes the adjacent radical nucleophilicity, thereby favouring translocation and distal coupling. Secondly, competing additions are reduced as the electrophilic CF_3_ radicals preferentially react with the unactivated alkene instead of electron-deficient Michael acceptors. Third, side reactions and hydrotrifluoromethylation are mitigated by judicious selection of reagents, stoichiometry, and visible-light conditions that promote the intended radical cascade. The conversion from radical to carbocation is inhibited by utilizing an electron-deficient acceptor that swiftly and selectively captures the nucleophilic radical before oxidation.

Motivated by a recent study from Zhu and co-workers on the synthesis of cyclopentanone derivatives using a photoinduced (3 + 2) cycloaddition involving HAT from a high-energy vinyl radical (Scheme 1c),^12^ we have further developed our methodology.^12^ Recognizing the immense synthetic utility of cyclopentane derivatives, we devised a tandem multicomponent, transition-metal-free reaction for their rapid synthesis using the bench-stable, inexpensive Langlois' reagent as the CF_3_ source.^13^ The sequence involves an initial HAT from a secondary to a tertiary carbon, followed by a second HAT from a vinylic to a secondary carbon, thus facilitating the synthesis of highly substituted cyclopentane derivatives. This operationally simple approach demonstrates both practicality and efficiency, enabling the construction of complex molecular architectures from readily available chemicals.

## Results and discussion

To develop a sustainable and operationally straightforward methodology for direct remote C(sp^3^)–H activation beyond *n* = 7, we began by targeting the 1,10-difunctionalization of unactivated alkenes. As a model substrate, we selected the unactivated alkene 1a (1 equiv.) and employed commercially available Langlois' reagent (CF_3_SO_2_Na, 2 equiv.) as a trifluoromethyl source along with Michael acceptor 2a (1.5 equiv.) as an alkylating reagent. AcOH (1 equiv.) was used as a proton source, and Acr-Mes^+^ClO_4_^−^ (1 mol%) served as a photocatalyst in DCM (2 mL). The reaction mixture was irradiated with a 456 nm blue LED (40 W) for 36 h, resulting in the desired 1,10-difunctionalized product 3a in a 31% yield. Additionally, 1,2-difunctionalized product 4a was obtained with a 16% yield and a minor yield of the simple hydrotrifluoromethylated product 5a (17%). To improve selectivity and minimize side products, we systematically screened a variety of photocatalysts. Among the tested catalysts, the organophotocatalyst Ph-Acr-Mes^+^BF_4_^−^ proved to be the most effective, increasing the yield of 3a to 40%, and of 4a to 25%, and reducing that of 5a to 18% (Table 1, entries 2–6). Subsequent solvent screening confirmed that DCM was the optimal solvent, delivering higher yields of 3a than other solvents (Table 1, entries 7–12). Further optimization focused on the amounts of CF_3_SO_2_Na and Michael acceptor 2a. Using 4 equiv. of CF_3_SO_2_Na and 2 equiv. 2a resulted in the highest yield of 3a at 61%, with that of 4a reduced to 14% and 5a to 6% (Table 1, entry 14). Adjusting the amount of AcOH had a negative effect: omitting AcOH or increasing its amount to 2 equiv. both led to lower yields (Table 1, entries 15 and 16). Among the acids tested, AcOH proved optimal, promoting faster protonation of the favored 1,10-addition intermediate, which is sterically and electronically favoured over those leading to 4a or 5a. Increased amounts of photocatalyst hampered the reaction (Table 1, entry 19). Additionally, control experiments showed no desired products were formed without the photocatalyst or light, highlighting their essential roles in this transformation (Table 1, entries 20 and 21). The optimal conditions were established as follows: 1 equiv. of 1a, 4 equiv. of CF_3_SO_2_Na, 2 equiv. of 2a, and 1 equiv. of AcOH with 1 mol% of photocatalyst in 2 mL DCM at room temperature, under 456 nm LED irradiation for 36 h.

**Table 1:** Optimization of the reaction conditions^a^

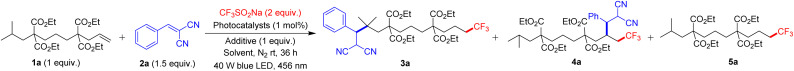						
Entry	Photocatalyst (1 mol%)	Solvent	Additive (1 equiv.)	3a (%)	4a (%)	5a (%)
1	Mes-Acr^+^ClO_4_^−^	DCM	AcOH	31	16	17
2	Ph-Acr-Mes^+^BF_4_^−^	DCM	AcOH	40	25	18
3	4-CzIPN	DCM	AcOH	19	12	4
4	Ir(dFCF_3_ppy)_2_(dtbbpy)	DCM	AcOH	2	6	1
5	Ru(bpy)_3_Cl_2_·6H_2_O	DCM	AcOH	Trace	Trace	Trace
6	T(*p*-CH_3_)PPT	DCM	AcOH	Trace	Trace	Trace
7	Ph-Acr-Mes^+^BF_4_^−^	CH_3_CN	AcOH	23	15	10
8	Ph-Acr-Mes^+^BF_4_^−^	EtOAc	AcOH	35	11	8
9	Ph-Acr-Mes^+^BF_4_^−^	MeOH	AcOH	31	17	7
10	Ph-Acr-Mes^+^BF_4_^−^	CHCl_3_	AcOH	13	14	8
11	Ph-Acr-Mes^+^BF_4_^−^	Acetone	AcOH	33	15	8
12	Ph-Acr-Mes^+^BF_4_^−^	CH_3_CN: DCM (1 : 1)	AcOH	30	13	10
11^b^	Ph-Acr-Mes^+^BF_4_^−^	DCM	AcOH	46	28	13
12^c^	Ph-Acr-Mes^+^BF_4_^−^	DCM	AcOH	50	10	18
13^d^	Ph-Acr-Mes^+^BF_4_^−^	DCM	AcOH	50	21	9
14^e^	Ph-Acr-Mes^+^BF_4_^−^	DCM	AcOH	61 (57)^j^	14	6
15^e^^,^^f^	Ph-Acr-Mes^+^BF_4_^−^	DCM	AcOH	43	16	10
16^e^	Ph-Acr-Mes^+^BF_4_^−^	DCM	—	42	16	5
17^e^	Ph-Acr-Mes^+^BF_4_^−^	DCM	HFIP	40	20	8
18^e^	Ph-Acr-Mes^+^BF_4_^−^	DCM	TFE	56	17	9
19^e^^,^^g^	Ph-Acr-Mes^+^BF_4_^−^	DCM	AcOH	45	18	4
20^e^^,^^h^	Ph-Acr-Mes^+^BF_4_^−^	DCM	AcOH	Nd	Nd	Nd
21^e^^,^^i^	Ph-Acr-Mes^+^BF_4_^−^	DCM	AcOH	Nd	Nd	Nd

a Reaction conditions: 1a (0.15 mmol, 1 equiv.), 2a (0.225 mmol, 1.5 equiv.), CF_3_SO_2_Na (0.3 mmol, 2 equiv.), photocatalyst (1 mol%), solvent (2.0 mL), irradiation with a 456 nm blue LED (40 W) under N_2_ atm, rt, 36 h, and ^1^H NMR yield using tetrachloroethane as an internal standard.

b 2 equiv. of 2a was used.

c 3 equiv. of CF_3_SO_2_Na.

d 3 equiv. of CF_3_SO_2_Na and 2 equiv. of 2a.

e 4 equiv. of CF_3_SO_2_Na and 2 equiv. of 2a.

f 2 equiv. of AcOH.

g 2 mol% Ph-Acr-Mes^+^BF_4_^−^.

h without light.

i without photocatalysts.

j isolated yield.

**Scheme 2:**
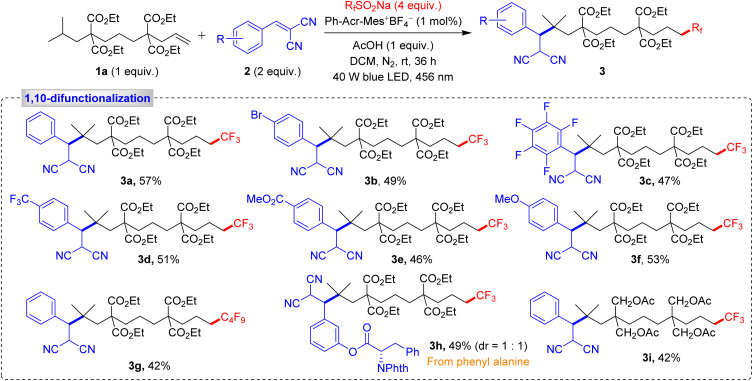
Substrate scope for the 1,10-difunctionalization of alkenes. Reaction conditions: 1a (0.15 mmol, 1 equiv.), 2 (0.3 mmol, 2 equiv.), R_f_SO_2_Na (0.6 mmol, 4 equiv.), Ph-Acr-Mes^+^BF_4_^−^ (1 mol%), AcOH (0.15 mmol, 1 equiv.), DCM (2.0 mL), irradiation with a 456 nm blue LED (40 W) under N_2_ atm., rt, 36 h.

Our research began with a comprehensive validation process, exploring a wide range of Michael acceptors and fluorine sources with unactivated alkene 1a. Under optimized conditions, we successfully obtained the 1,10-difunctionalized product 3a in a notable 57% yield, along with 14% of the 1,2-difunctionalized product 4a and 6% of the hydrotrifluoromethylated product 5a, as shown in Scheme 2. We further examined the reactivity of various Michael acceptors, including those with halogen substituents on the aryl ring and pentafluorobenzene derivatives. These reactions yielded products 3b and 3c at 49% and 47%, respectively. Michael acceptors containing electron-withdrawing or electron-donating groups also furnished the expected products 3d–3f with yields ranging from 46 to 53%. Additionally, perfluoroalkylated product 3g was synthesized in 42% yield, with the biologically active Michael acceptor yielding product 3h in 49% yield. An alkene with alkyl substituents at 4,8-positions afforded product 3i in 42% yield, indicating that gem-diesters are not essential and alkyl substituents can equally promote the reaction. Next, we extended our methodology for 1,11-difunctionalization using a dialkene to achieve selective remote C–H activation at the 11-position relative to the alkene.

**Scheme 3:**
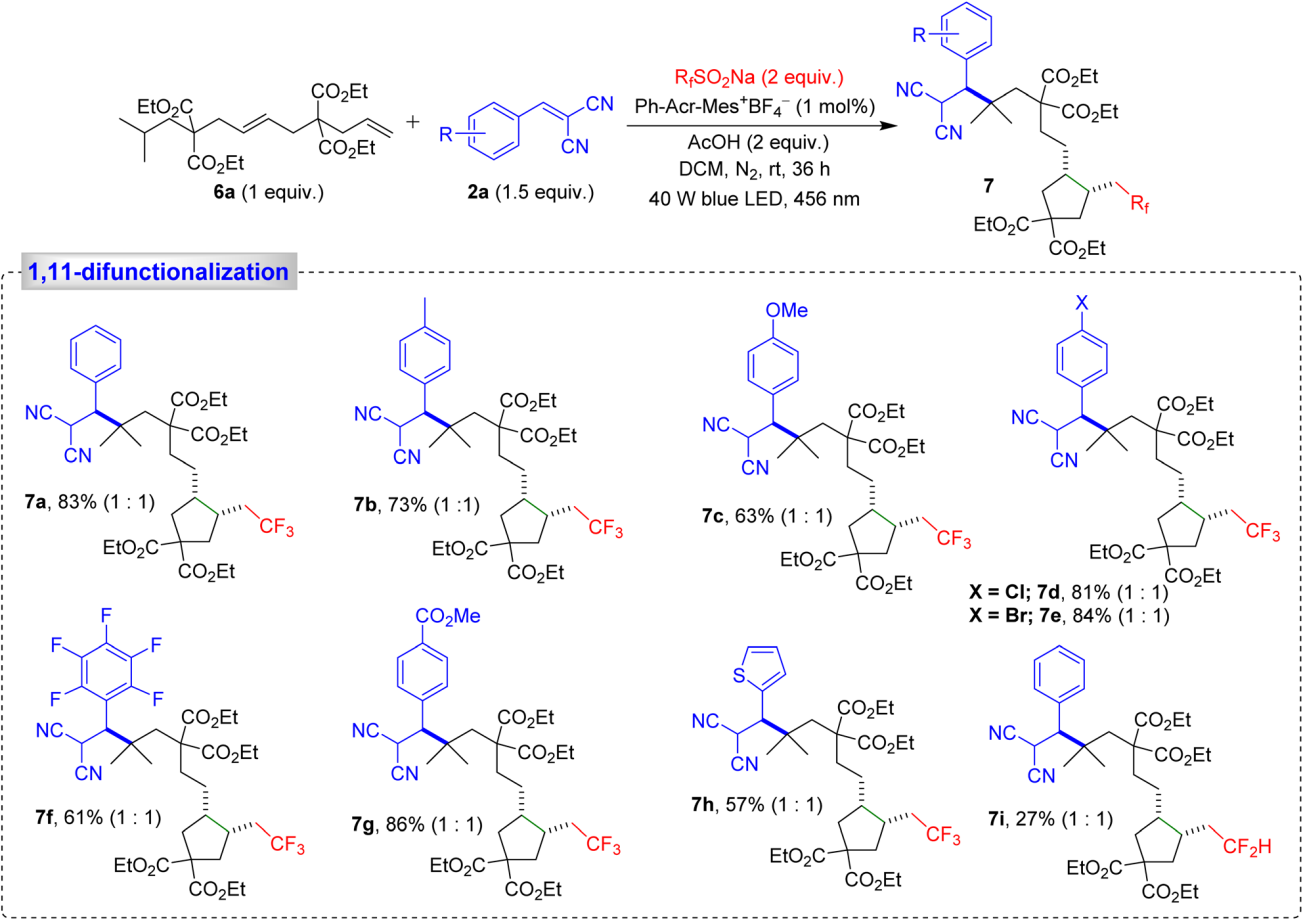
Substrate scope for the 1,11-difunctionalization of alkenes. Reaction conditions: 6a (0.15 mmol, 1 equiv.), 2 (0.3 mmol, 2 equiv.), R_f_SO_2_Na (0.3 mmol, 2 equiv.), Ph-Acr-Mes^+^BF_4_^−^ (1 mol%), AcOH (0.15 mmol, 1 equiv.), DCM (2.0 mL), irradiation with a 456 nm blue LED (40 W) under N_2_ atm., rt, 36 h. The diastereomeric ratio is given in parentheses.

When dialkene 6a was subjected to our modified reaction conditions, a remarkable tandem sequence unfolded, involving trifluoromethylation of the terminal alkene, 5-*exo-trig* cyclization, 1,5-HAT, and C(sp^3^)–H alkylation, leading to the formation of product 7a, with an impressive 83% yield (Scheme 3). This cascade reaction highlights the unique advantages of our methodology, which efficiently combines trifluoromethylation, cyclopentane ring formation, and alkylation in a single sequence. We also examined the effect of substituents on the Michael acceptors. A 4-methyl-substituted aryl ring produced the desired product 7b in 73% yield, while electron-donating substituents on the aryl ring led to product 7c in 63% yield. Halo-substituted phenyl rings generated products 7d–7f, with yields ranging from 61% to 84%, while a strongly electron-withdrawing substituent on the phenyl ring furnished the expected product 7g in an excellent 86% yield. Additionally, a thiophene-derived Michael acceptor yielded product 7h with a moderate 57% yield (Scheme 3). Next, we examined the scope of the perfluoroalkyl group CF_2_H as an alternative to the trifluoromethyl group. This furnished the corresponding product 7i with a satisfactory yield (Scheme 3).

**Scheme 4:**
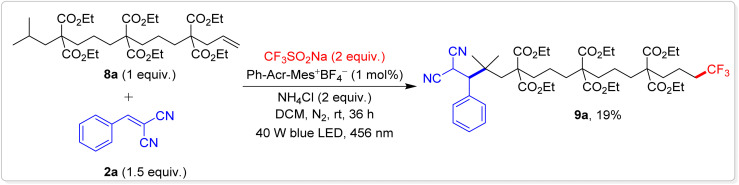
Substrate scope for the 1,14-difunctionalization of alkenes. Reaction conditions: 8a (0.15 mmol, 1 equiv.), 2a (0.3 mmol, 1.5 equiv.), CF_3_SO_2_Na (0.3 mmol, 2 equiv.), Ph-Acr-Mes^+^BF_4_^−^ (1 mol%), NH_4_Cl (0.3 mmol, 2 equiv.), DCM (2.0 mL), irradiation with a 456 nm blue LED (40 W) under N_2_ atm., rt, 36 h.

One of the most ground-breaking aspects of our study is the first reported example of 1,14-difunctionalization of unactivated alkenes. This was accomplished using an alkene with two secondary carbons at the 1,6- and 1,10-positions and a strategically engineered tertiary carbon at the 1,14-position (Scheme 4). Under our reaction conditions, alkene 8a underwent a tandem trifluoromethylation followed by three HAT processes resulting in the unprecedented 1,14-trifluoromethylative-alkylated product 9a with a 19% yield. This transformation was facilitated by a 1,13-radical translocation mechanism involving 1,5-HAT events. Several competing side reactions, such as 1,2- and 1,6-additions, along with hydrotrifluoromethylation, were observed, collectively diminishing the overall yield of the desired 1,14-difunctionalized product. The successful execution of this complex reaction opens up new possibilities for remote C(sp^3^)–H bond functionalization, pushing the boundaries of what can be achieved in this field.

**Scheme 5:**
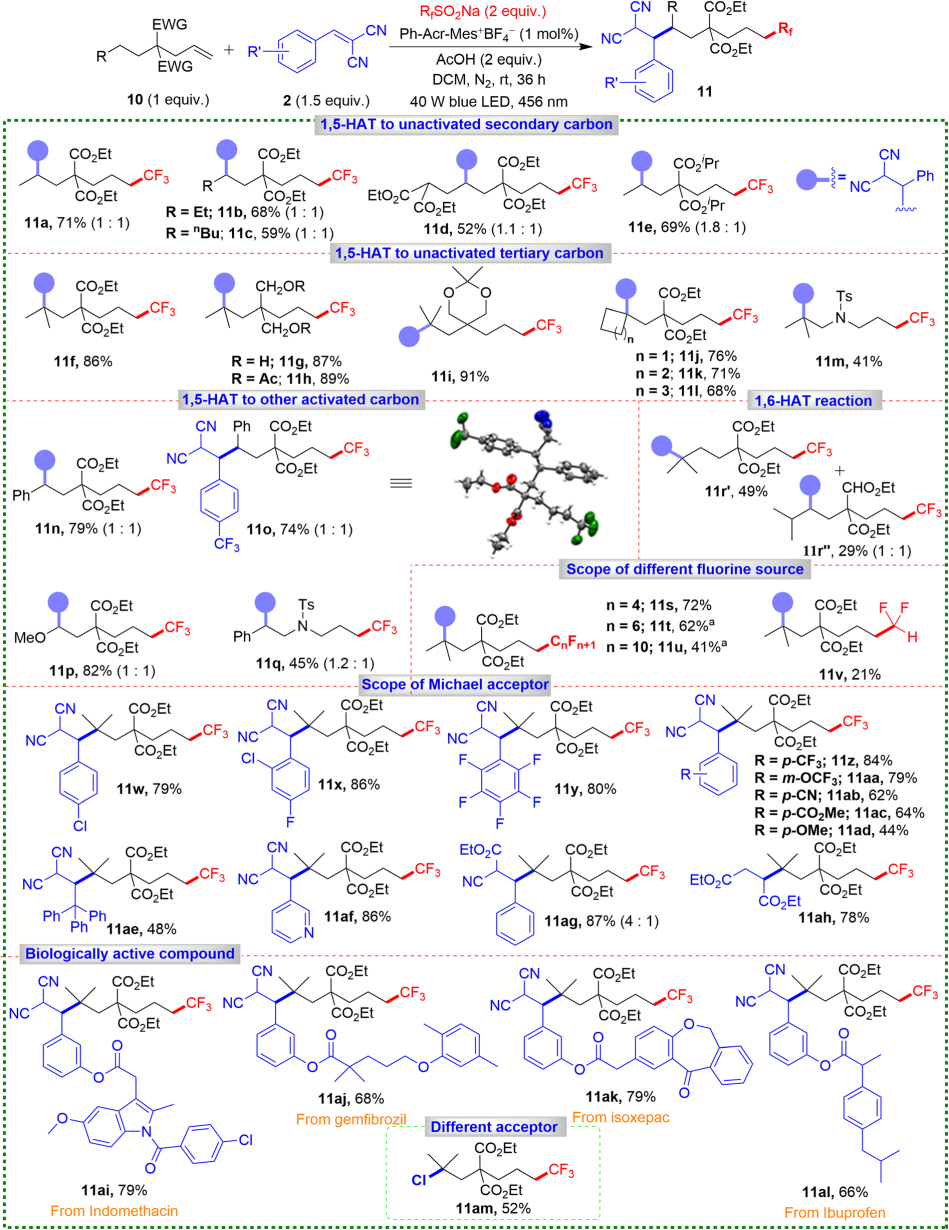
Substrate scope for 1,6-difunctionalization of alkenes. Reaction conditions: 10 (0.15 mmol, 1 equiv.), 2a (0.3 mmol, 1.5 equiv.), CF_3_SO_2_Na (0.3 mmol, 2 equiv.), Ph-Acr-Mes^+^BF_4_^−^ (1 mol%), AcOH (0.3 mmol, 2 equiv.), DCM (2.0 mL), irradiation with 456 nm blue LED (40 W) under N_2_ atm., rt, 36 h. Diastereomeric ratios are given in parentheses. ^*a*^2.5 mol% of Ph-Acr-Mes^+^BF_4_^−^.

We extended our 1,6-difunctionalization strategy to a broad range of unactivated alkenes with remote secondary C(sp^3^)–H bonds. These experiments demonstrated hydrogen migration between one secondary carbon and another with similar BDEs (Scheme 5). This approach efficiently synthesized the remote 1,6-difunctionalized product 11a, in a yield of 71% under modified conditions. For alkenes with longer alkyl chains and multiple secondary C(sp^3^)–H bonds, products 11b and 11c were obtained in good yields, showcasing high regioselectivity of the reaction. The robustness of our methodology was exemplified by the synthesis of product 11d, achieved in 52% yield, where the most acidic proton remains untouched. Introducing isopropyl-substituted ester groups expanded the scope, resulting in product 11e in a yield of 69%.

Extending the substrate scope to that containing a tertiary C(sp^3^)–H bond, product 11f was obtained with an impressive 86% yield. The different substituents on the alkyl chain furnished products 11g–11i in excellent yields (87–91%). Substrates containing more sterically hindered cyclic tertiary C(sp^3^)–H bonds also yielded products 11j–11l with minimal steric interference (68–76%). Introducing an –NTs group on the alkyl chain delivered 11m in a moderate 41% yield, and products 11n–11q were achieved in 45–82% yields when remote C(sp^3^)–H bonds or benzylic or heteroatom-containing bonds were activated. Notably, for substrates with a secondary C(sp^3^)–H bond at the 1,6-position and a tertiary C(sp^3^)–H bond at the 1,7-position, geometric factors favored 1,6-addition. However, the lower BDE of the tertiary C(sp^3^)–H bond led to the predominant formation of 1,7-difunctionalized product 11r′ (49%) alongside the 1,6-difunctionalized product 11r″ (29%). Next, we explored perfluoroalkyl sources previously undocumented (Scheme 5). Introducing the –C_4_F_9_ group gave product 11s in 72% yield. Other perfluoroalkyl groups (–C_6_F_13_ and –C_8_F_17_) delivered products 11t and 11u in 62% and 41% yields under higher photocatalyst loading (2.5 mol%).

Difluoromethylation gave 11v in a lower yield (21%), possibly due to the less electrophilic nature of the difluoromethyl group, causing direct difluoromethylation of the Michael acceptor 2a rather than the alkene. Our methodology accommodated various Michael acceptors (Scheme 5), yielding products 11w–11y in good yields, even with aryl ring-bearing electron-withdrawing and electron-donating groups (11z–11ad). Introducing a bulkier triphenyl-substituted Michael acceptor provided 11ae in 48% yield. Other acceptors furnished products 11af–11ah in excellent yields (78–87%), and biologically active derivatives (11ai–11al) were obtained in good yields (66–79%), illustrating the method's broad applicability, including in late-stage functionalization of pharmaceuticals. When we used *N*-chlorophthalimide instead of Michael acceptor, the 1,6-chlorotrifluoromethylated product 11am was obtained in 52% yield, which showed the future scope of this methodology.

**Scheme 6:**
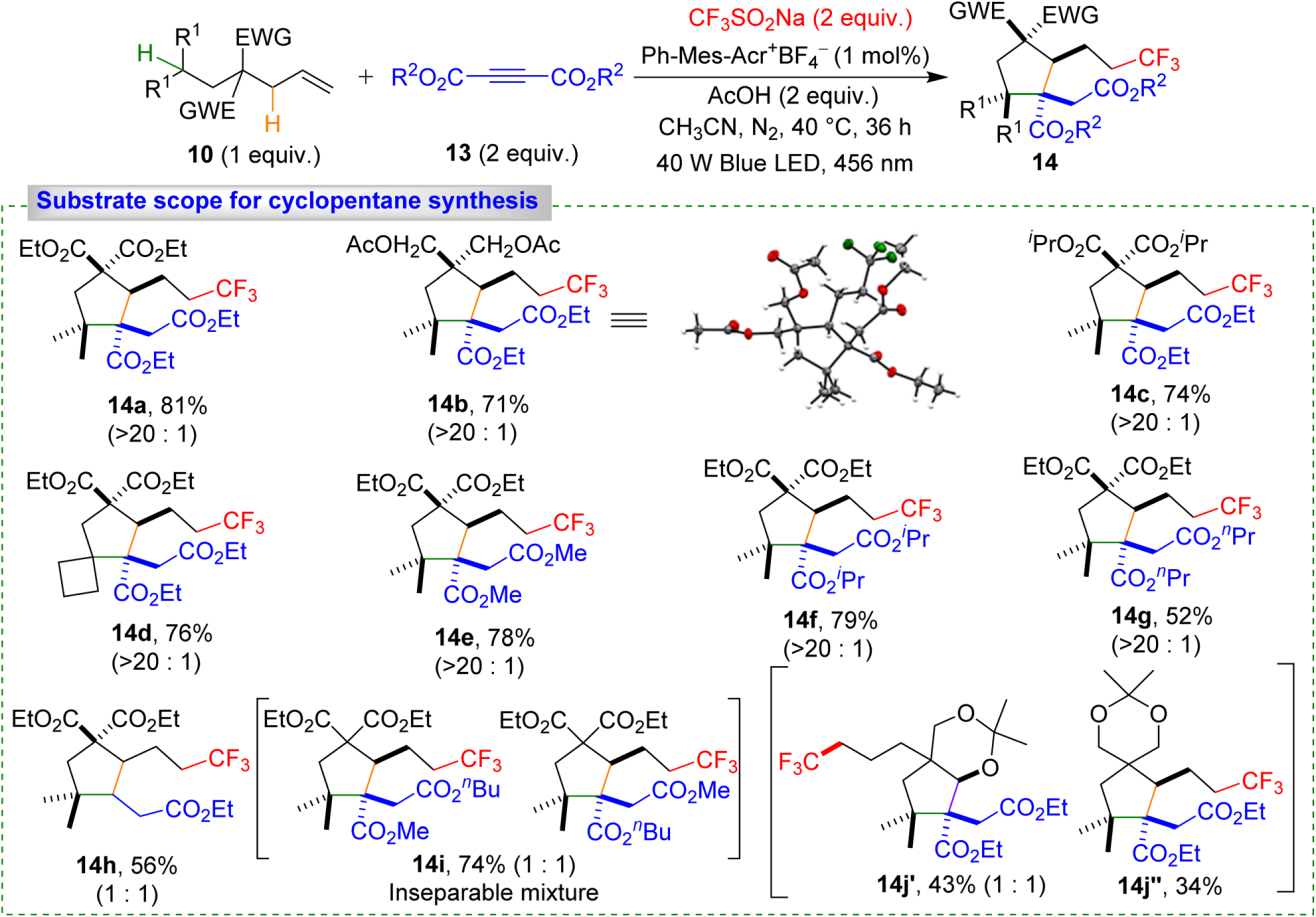
Substrate scope for cyclopentane derivatives. Reaction conditions: 10 (0.15 mmol, 1 equiv.), 13 (0.3 mmol, 1.5 equiv.), CF_3_SO_2_Na (0.3 mmol, 2 equiv.), Ph-Acr-Mes^+^BF_4_^−^ (1 mol%), AcOH (0.3 mmol, 2 equiv.), MeCN (2.0 mL), irradiation with a 456 nm blue LED (40 W) under N_2_ atm., 40 °C, 36 h. Diastereomeric ratios are given in parentheses.

Our strategy proved highly effective for synthesizing densely substituted cyclopentane derivatives *via* initial 1,5-HAT from secondary to tertiary carbon (Scheme 6). Optimized conditions (see ESI) afforded cyclopentane derivative product 14a with 81% yield. Alkenes with different alkyl chain substituents provided 14b–14c in 71–74% yield, and even bulkier cyclic substrates gave 14d in 76% yield. Electron-deficient alkynes also reacted smoothly, yielding 14e–14h in moderate to good yields. Mixed alkyne reactions produced 14i as a 1 : 1 mixture of two isomers in 74% yield. Alkenes with two distinct types of C(sp^3^)–H bonds in the second 1,6-HAT step yielded 14k′ and 14k″ in 43% and 34% yields, respectively. The slightly higher yield of 14k′ was attributed to the lower BDE of the oxygen-adjacent C(sp^3^)–H bond, a key factor in the second 1,6-HAT process. The high diastereoselectivity (>20 : 1) observed for products 14a–14g is likely governed by steric factors. In contrast, when a terminal alkyne was employed, the diastereoselectivity completely disappeared, affording product 14h as a 1 : 1 mixture of diastereomers.

**Scheme 7:**
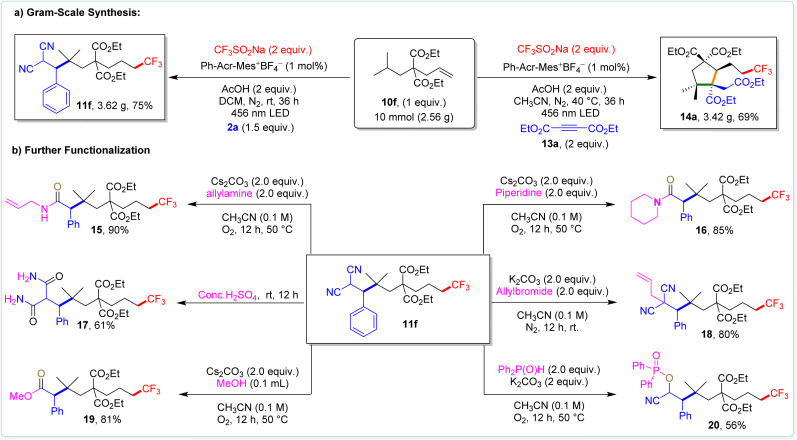
Gram-scale synthesis and further functionalization.

**Scheme 8:**
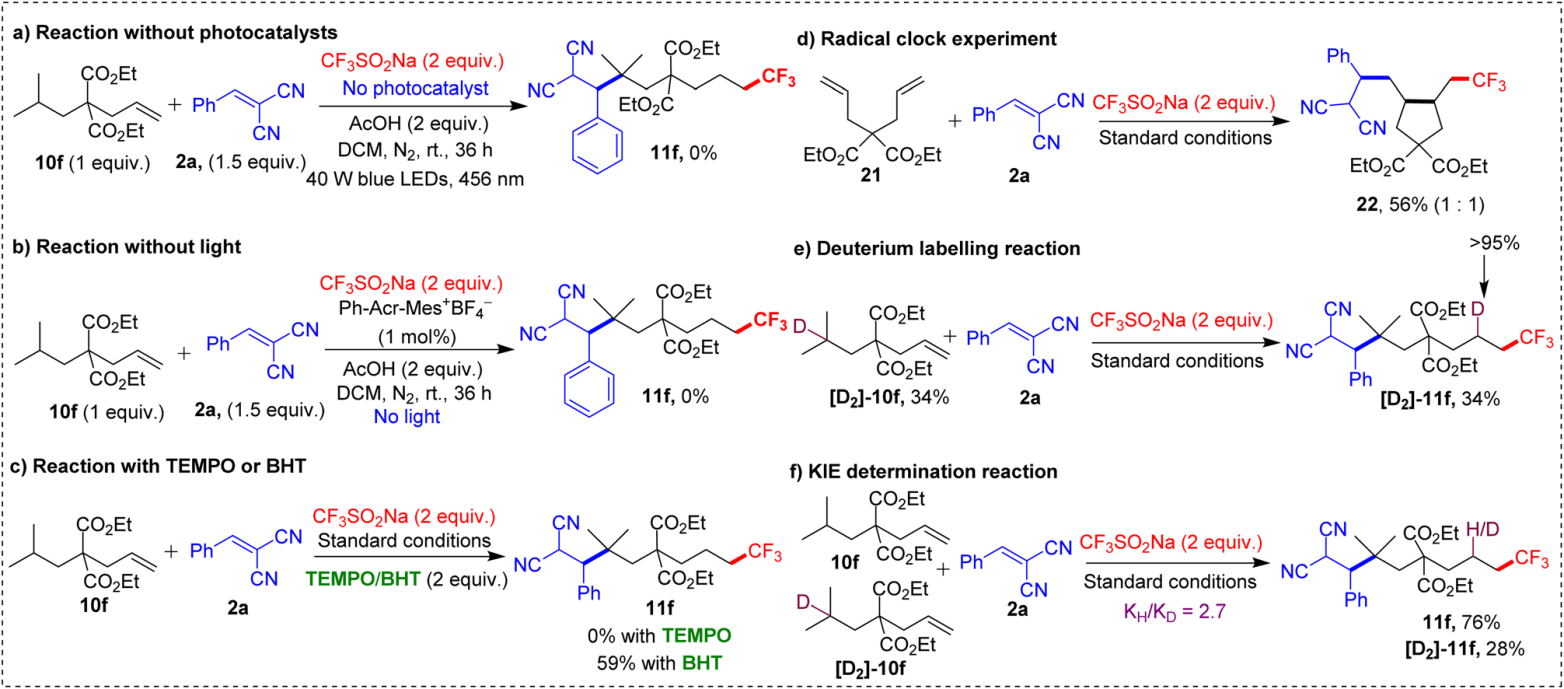
Control experiments and mechanistic studies.

To show the practical applicability, we synthesized compounds 11f and 14a on a 10 mmol scale, achieving 75% and 69% yields, respectively (Scheme 7a). Furthermore, we successfully converted 11f into various valuable functional groups (Scheme 7b), showcasing the versatility of our method for synthesizing compounds with diverse functionalities. To unravel the underlying mechanism of the 1,*n*-difunctionalization of alkenes, we conducted a series of carefully designed control experiments using 10f as a model substrate (Scheme 8a–8f).

The lack of product in the absence of photocatalyst and light highlighted their essential roles in driving the reaction. The use of radical scavengers such as 2,2,6,6-tetramethylpiperidine 1-oxyl (TEMPO) and butylated hydroxytoluene (BHT) confirmed the involvement of a radical pathway. TEMPO completely inhibited the reaction, and BHT significantly reduced the yield, indicating a radical mechanism. Further evidence for this radical pathway was provided by a radical clock experiment using 2a and dialkene 21, which yielded trifluoromethylation/5-*exo-trig* cyclization/alkylation product 22 in 56% yield. The 1,5-HAT was identified as the rate-determining step, based on a reaction with deuterated [D_2_]-10f, which gave [D_2_]-11f (>95% D) in 34% yield. The observed kinetic isotope effect (KIE) of 2.7 further reinforced this conclusion. Luminescence quenching experiments also shed light on the interaction between the Langlois' reagent and the photocatalyst, providing additional mechanistic insights.

**Scheme 9:**
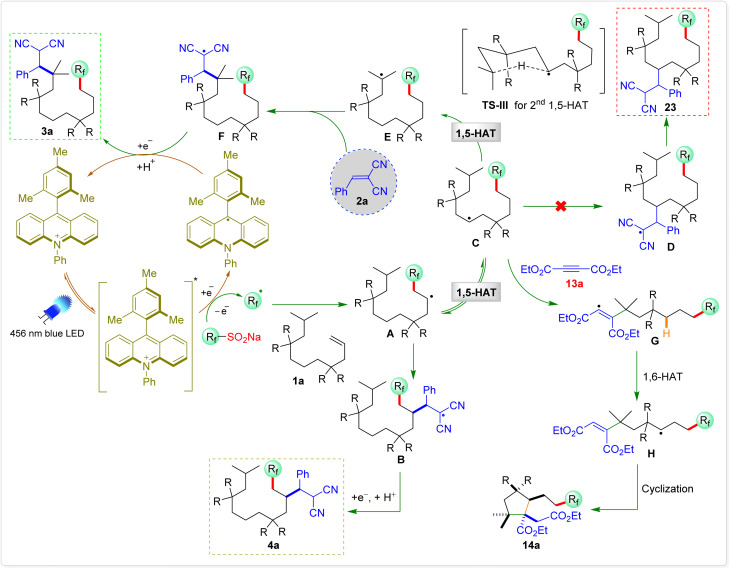
A plausible mechanism.

The proposed mechanism for the 1,10-difunctionalization of alkenes is depicted in Scheme 9. Under blue LED irradiation, the excited-state photocatalyst Ph-Mes-Acr^+^* is generated from Ph-Mes-Acr^+^. This excited-state photocatalyst (
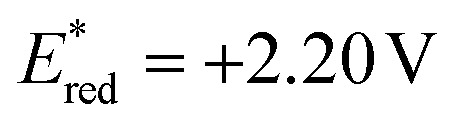
*vs.* saturated calomel electrode (SCE)) is capable of accepting an electron from CF_3_SO_2_Na (*E*_ox_ = 1.05 V *vs.* SCE), generating a trifluoromethyl radical and Ph-Mes-Acr^˙^ radical intermediate.^14^ The trifluoromethyl radical then reacts with alkene 1a to form the radical intermediate A, which earlier reacts with Michael acceptor 2a to produce the radical intermediate B or undergoes a 1,5-HAT (TS-I) to form radical intermediate C, which again undergoes a second 1,5-HAT (TS-III) to generate radical intermediate E, which reacts with 2a to form intermediate F.^15^ An electron transfer from Ph-Mes-Acr˙ to radical intermediate F followed by protonation yields the desired 1,10-difunctionalized products 3a, regenerating photocatalyst.

A similar reaction mechanism is proposed for the synthesis of cyclopentane derivatives. The trifluoromethyl radical first adds to alkene 10f, generating the alkyl radical intermediate A, which subsequently undergoes an intramolecular 1,5-HAT to afford radical intermediate C. This transformation effectively shifts the radical center to a more stabilized position. The resulting radical then adds to an electron-deficient alkyne 15a, forming the vinylic radical intermediate G. Importantly, this intermediate is electrophilic in nature and undergoes an intramolecular 1,6-HAT from the accessible C6 hydrogen atom (relative to the vinyl radical center), leading to the formation of alkyl radical intermediate H.^16*a*^ This sequential HAT process strategically relays the radical center along the molecular framework, positioning it at a site suitable for ring closure. The newly generated radical H then undergoes a kinetically favourable 5-*exo-trig* cyclization, forming the cyclopentane core.^16*b*^ Finally, single-electron reduction of the resulting radical and subsequent protonation furnish the highly substituted cyclopentane derivative 14a, while concurrently regenerating the photocatalyst.

## Conclusions

In summary, we have developed the remote tandem multicomponent 1,*n* (*n* = 10, 11, 14, 6, 7) difunctionalization (trifluoromethylation or perfluoroalkylation followed by alkylation) of an unactivated alkene *via* net 1,*n* radical translocations under a visible-light-driven straightforward process under milder reaction conditions. This redox-neutral and entirely transition-metal-free methodology is highly chemoselective and regioselective. We also developed a multicomponent transition-metal-free tandem reaction for synthesizing highly substituted cyclopropane derivatives by the visible-light-driven, straightforward remote functionalization of an unactivated alkene. A sequence of HAT processes is involved in the reaction. The first 1,5-HAT occurs from secondary carbon to tertiary carbon, followed by 1,6-HAT from vinylic carbon to secondary carbon, followed by 5-*exo-trig* radical cyclization. Bench-stable, cheap, and commercially available Langlois' reagent was used as a trifluoromethyl source. The scalability of the method has been shown by performing the reaction on the gram scale, and further functionalization shows the synthetic evolution of the developed methodology.

## Author contributions

K. G. G. and D. S. designed the project. K. G. G. performed optimization studies, D. D. and V. S. did substrate scope analysis, and K. P. did mechanistic studies. K. G. G., V. S. and D. S. wrote the manuscript. All authors have given approval to the final version of the manuscript.

## Conflicts of interest

There are no conflicts to declare.

## Supplementary Material

SC-OLF-D5SC08683C-s001

SC-OLF-D5SC08683C-s002

## Data Availability

CCDC 2416402 and 2264468 contain the supplementary crystallographic data for this paper.^17*a*,*b*^ The data supporting this article have been included as part of the supplementary information (SI). Supplementary information: detailed experimental procedures, characterization data (^1^H and ^13^C NMR spectra), and crystal structures. See DOI: https://doi.org/10.1039/d5sc08683c.
