# Supplementary material for: Photocatalytic remote C(sp3)–H alkylation of long-chain alkenes: A tandem multicomponent approach via radical translocation
Source: Chem Sci. 2026 Jan 21;17(12):5888–98. doi: 10.1039/d5sc08683c (PMC12863196; doi:10.1039/d5sc08683c)
Supplement: SC-017-D5SC08683C-s001 [file SC-017-D5SC08683C-s001.pdf]

Supplementary Information (SI) for Chemical Science.
This journal is © The Royal Society of Chemistry 2026

Supporting Information

Photocatalytic remote C(sp^3)–H alkylation of long-chain alkenes: A tandem multicomponent approach *via* radical translocation

Krishna Gopal Ghosh[a], Debabrata Das[a], Vinjamuri Srinivasu[a], Koustav Pal[a] and Devarajulu Sureshkumar*[a]
Department of Chemical Sciences, Indian Institute of Science Education and Research Kolkata, Mohanpur, West Bengal 741246

1. General information S2
2. Optimization of reaction conditions S3
3. Experimental procedure for synthesizing starting materials **1a**, **6a**, **8a** and **10** S5
4. Experimental procedure for synthesizing compounds **3**, **7**, **9a**, **11**, **14** S9
5. Gram-scale synthesis of **11f, 14a** S11
6. Further functionalization S12
7. Control experiments S14
8. Light ON/OFF experiments S17
9. Luminescence quenching studies S18
10. EPR Experiment S19
11. X-Ray data S22
12. Characterization data of the compounds **1**, **3, 6, 7, 8, 9, 10, 11,** and **14** S24
13. NMR spectra of the products S42

Department of Chemical Sciences
Indian Institute of Science Education and Research Kolkata
Mohanpur 741246, West Bengal, India.
E-mail: suresh@iiserkol.ac.in
Group webpage: https://www.iiserkol.ac.in/~suresh/

1. General Information:

Commercial reagents were purified before use, following the guidelines of L. L Chai and Armarego. All NMR spectra were recorded on 400 MHz JEOL and 500 MHz Bruker spectrometers. ^{1}H, ^{13}C, and ^{19}F spectral data are reported as chemical shifts (δ) in parts per million (ppm). Chemical shifts are reported in parts per million (ppm) relative to tetramethylsilane (TMS), which serves as the internal standard in $CDCl_3$. Chemical shifts (δ) are quoted in parts per million (ppm), and coupling constants (*J*) are measured in Hertz (Hz). The following abbreviations describe multiplicities s = singlet, d = doublet, t = triplet, q = quartet, pent = pentet, b = broad, m = multiplet. NMR spectra were processed in MestreNova, keeping the $CDCl_3$ residual peaks at 7.26 ppm (^{1}H) and 77.16 ppm (^{13}C). High-resolution mass spectra (HRMS, m/z) were recorded on a Waters ESI HRMS instrument. All fluorescence and UV−vis spectra were recorded in a Hitachi F-7000 spectrofluorometer and a Hitachi UV−Vis spectrophotometer. X-ray diffraction data for the crystal were collected at 100 K using a Rigaku (dual, Cu/Mo at zero Eos) diffractometer with monochromatic Cu-Kα radiation and a 100 μm beam size. IUPAC names were obtained using the ChemDraw service. Unless otherwise noted, all reactions were conducted in dried glassware with magnetic stirring under an argon atmosphere. All solvents were dried following the guidelines of L. L Chai and Armarego for the purification of laboratory chemicals. Organic solutions were concentrated under reduced pressure on a Büchi rotary evaporator. Flash column chromatography was performed over Merck silica gel (230−400 μm) using the eluent system described for each experiment. Thin-layer chromatography (TLC) was performed using Silicagel 60 F254 and visualized under ultraviolet light/iodine-chamber/$KMnO_4$ stain. The photocatalysts Ph-Acr-mes$^{+}BF_4^{-}$ were prepared using the procedures outlined in the literature. All other chemicals and reagents were obtained from commercial suppliers, including Sigma-Aldrich, Alfa Aesar, BLD Pharm, TCI, and Spectrochem. For general photocatalytic reactions, 40 W-456 nm blue LEDs (PR-160) were purchased from Kessil. They were placed approximately 2 inches away from the reaction tube, and a cooling fan was used approximately 15 inches from the reaction tube to maintain the ambient room temperature. The product yields were determined after purification by flash column chromatography using SiO_2, and ^{1}H NMR was used to determine the purity.

2. Optimization of the reaction conditions:

2.1. Optimization of the reaction conditions for the 1,6-difunctionalization of alkene:

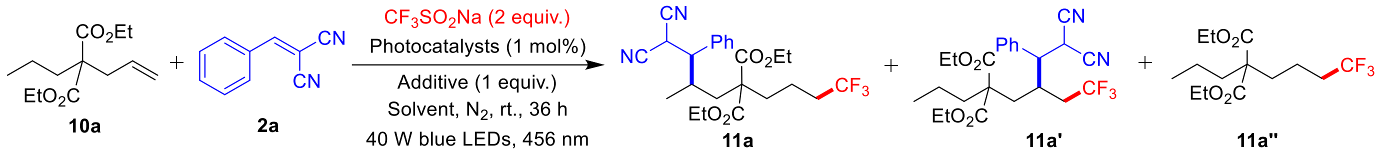

Entry	Photocatalyst (1 mol%)	Solvent	Additive (1 equiv.)	11a (%)	11a′ (%)	11a″ (%)
1	Acr-mes$^+ClO_4^-$	DCM	-	51 (1.3: 1)	25 (1.1: 1)	6
2	Ph-Acr-mes$^+ClO_4^-$	DCM	-	57 (1.1: 1)	24 (1.1: 1)	<5
3	T(*p*-CH_3)PPT	DCM	-	<5	<5	<5
4	Ir($dfCF_3$)(dtbbpy)$_2PF_6$	DCM	-	<5	<5	<5
5	Ph-Acr-mes$^+ClO_4^-$	DCE	-	55 (1.1: 1)	28 (1: 1)	<5
6	Ph-Acr-mes$^+ClO_4^-$	$CHCl_3$	-	28 (1.1: 1)	17 (1: 1)	<5
7	Ph-Acr-mes$^+ClO_4^-$	CH_3CN	-	56 (1 : 1)	19 (1 : 1)	11
8	Ph-Acr-mes$^+ClO_4^-$	Benzene	-	<5	<5	<5
9	Ph-Acr-mes$^+ClO_4^-$	Toluene	-	<5	<5	<5
10	Ph-Acr-mes$^+ClO_4^-$	EtOAc	-	55 (1 : 1)	21 (1 : 1)	<5
11	Ph-Acr-mes$^+ClO_4^-$	DCM	NH_4Cl	62 (1.1: 1)	26 (1.1: 1)	11
12	Ph-Acr-mes$^+ClO_4^-$	DCM	TBACl	61 (1.1: 1)	11 (10: 1)	17
13	Ph-Acr-mes$^+ClO_4^-$	DCM	*p*-TSOH	42 (1: 1)	35 (1: 1)	24
14	Ph-Acr-mes$^+ClO_4^-$	DCM	TFA	38 (1: 1)	22 (1: 1)	21
15	Ph-Acr-mes$^+ClO_4^-$	DCM	TfOH	27 (1: 1)	23 (1: 1)	31
16	Ph-Acr-mes$^+ClO_4^-$	DCM	HFIP	57 (1: 1)	30 (1: 1)	11
17	Ph-Acr-mes$^+ClO_4^-$	DCM	AcOH	72 (1: 1)	19 (1: 1)	8
**18[c]**	**Ph-Acr-mes$^+ClO_4^-$**	**DCM**	**AcOH**	**74 (1: 1)**	**18 (1: 1)**	**9**
18[d]	Ph-Acr-mes$^+ClO_4^-$	DCM	AcOH	45 (1: 1)	44 (1: 1)	16
19[e]	Ph-Acr-mes$^+ClO_4^-$	DCM	*p*-TsOH	0	0	87

[a] Reaction conditions: **1a** (0.15 mmol, 1 equiv.), **2a** (0.23 mmol, 1.5 equiv.), CF_3SO_2Na (0.3 mmol, 2 equiv.), photocatalyst (1 mol%), solvent (2.0 mL), irradiation with 456 nm blue LED (40 W) under N_2 atm, rt, 36 h, [b] ^1H NMR Yield using tetrachloroethane as an internal standard, [c] 2 equiv. of AcOH was used, [d] 1 mL of DCM was used, [e] 2 equiv. of *p*-TsOH was used.

2.2. Optimization of the reaction conditions for the cyclopentane derivative synthesis:

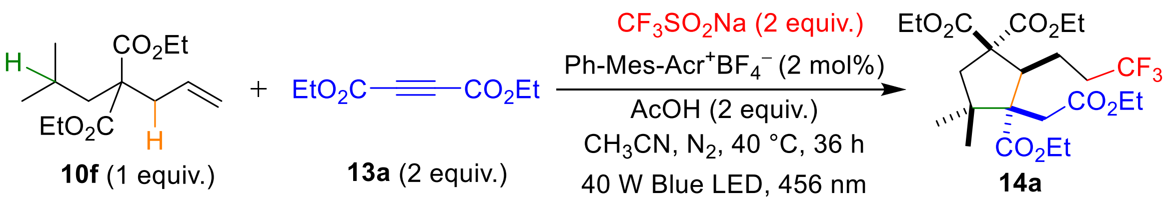

Entry	Deviation from the standard reaction conditions	Yield 14a (%)[b]
1	-	76
2	3 equiv. of CF_3SO_2Na used	64
3	3 equiv. of **2a** used	59
4	5 mol% catalysts used	62
5	36 h reaction time	81
6	1 mol% catalysts used, 36 h	86
7	3 equiv. of **2a** used, 36 h	69
8	3 equiv. of **2a** and 1.5 equiv. of CF_3SO_2Na used, 36 h	58
9	3 mL CH_3CN used, 36 h	62
10	1 mL CH_3CN used, 36 h	48
11	1 mol% catalysts used at 40 °C, 36 h	89

[a] Reaction conditions: **1a** (0.15 mmol, 1 equiv.), **2a** (0.3 mmol, 2 equiv.), CF_3SO_2Na (0.3 mmol, 2 equiv.), photocatalyst (2 mol%), solvent (2.0 mL), AcOH (1 equiv.), irradiation with 456 nm Blue LED (40 W) under N_2 atm, rt, 24 h, [b] ^1H NMR Yield using tetrachloroethane as internal standard.

3. The general route for the synthesis of starting materials 1a, 6a, 8a and 10:

3.1. Preparation of tetraethyl 10-methylundec-1-ene-4,4,8,8-tetracarboxylate (1a):

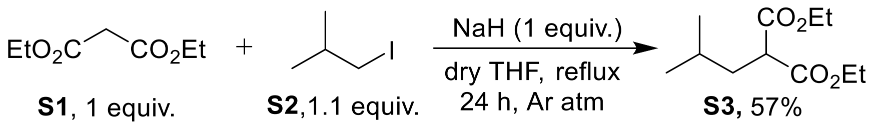

A 250 mL two-neck round-bottom flask, equipped with a magnetic stirrer, was placed in an oil bath. The flask was flushed with argon, and a reflux condenser connected to an argon-filled balloon was attached to one of its necks. Sodium hydride(NaH 60% in mineral oil; 2.0 g, 50 mmol, 1 equiv.) was carefully added to the flask under an argon atmosphere. The second neck was sealed with a septum. Freshly dried THF (100 mL) was added to the flask *via* a syringe, forming a suspension. Diethyl malonate (**S1**, 8.01 g, 50 mmol, 1 equiv.) was then introduced dropwise to the stirred NaH/THF mixture using a syringe. The reaction mixture was heated at 60 °C for 1 h to form the enolate, cooled to room temperature, and 1-iodo-2-methylpropane (**S2**, 55 mmol, 1.1 equiv.) was added *via* syringe through the septum. The reaction was heated to reflux for 24 h under argon. After cooling to room temperature, the mixture was cautiously quenched with saturated aq. NH_4Cl solution and extracted with ethyl acetate (3 × 50 mL). The combined organic layers were dried over anhydrous Na_2SO_4, concentrated under reduced pressure, and purified by column chromatography using a hexane/ethyl acetate mixture as the eluent to obtain product **S3** in 57% yield (6.16 g).

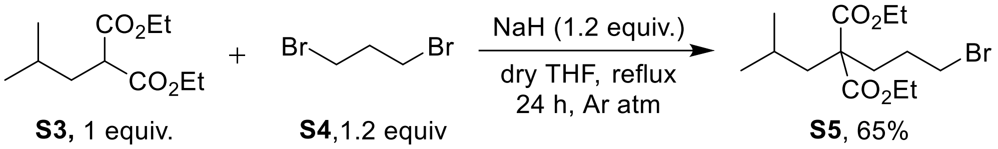

A 250 mL two-neck round-bottom flask, equipped with a magnetic stirrer, was placed in an oil bath and flushed with argon. A reflux condenser connected to an argon-filled balloon was attached to one of the necks. Sodium hydride (NaH 60% in mineral oil; 30 mmol, 1.2 equiv.) was carefully added to the flask under an atmosphere. The second neck was sealed with a septum. Freshly dried THF (100 mL) was added to the flask *via* a syringe, forming a suspension. Diethyl 2-isobutyl malonate (**S3**, 5.4 g, 25 mmol, 1 equiv.) was then introduced dropwise to the stirred NaH/THF mixture using a syringe. The reaction mixture was heated at 60 °C for 1 h to generate the enolate, cooled to room temperature, and 1,3-dibromopropane (30 mmol, 1.2 equiv.) was added *via* a syringe through the septum. The reaction was heated to reflux for 24 h under argon. After cooling to room temperature, the mixture was cautiously quenched with saturated aq. NH_4Cl and then extracted with ethyl acetate (3 × 50 mL). The combined organic layers were dried over anhydrous Na_2SO_4, concentrated under reduced pressure, and purified by column chromatography using a hexane/ethyl acetate mixture as the eluent to afford product **S5** in 65% yield (5.48 g).

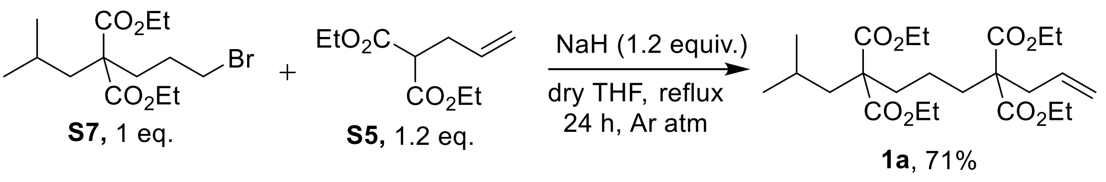

A 100 mL two-neck round-bottom flask, equipped with a magnetic stirrer, was placed in an oil bath and flushed with argon. A reflux condenser connected to an argon-filled balloon was attached to one of the necks. Sodium hydride (NaH 60% in mineral oil; 19.2 mmol, 1.2 equiv.) was carefully added to the flask in an argon atmosphere. The second neck was sealed with a septum. Freshly dried THF (100 mL) was added to the flask *via* a syringe, forming a suspension. Diethyl allyl malonate (3.84 g, 19.2 mmol, 1.2 equiv.) was added dropwise to the stirred NaH/THF mixture using a syringe. The reaction mixture was heated at 60 °C for 1 h to generate the enolate, cooled to room temperature, and diethyl 2-(3-bromopropyl)-2-isobutylmalonate (16 mmol, 1 equiv.) was added *via* syringe through the septum. The reaction was heated to reflux for 24 h under argon. After cooling to room temperature, the mixture was cautiously quenched with saturated aq. NH_4Cl and extracted with ethyl acetate (3 × 50 mL). The combined organic layers were dried over anhydrous Na_2SO_4, concentrated

under reduced pressure, and purified by column chromatography using hexane/ethyl acetate as eluent to afford the tetraethyl 10-methylundec-1-ene-4,4,8,8-tetracarboxylate (**1a**) in 71% yield (5.19 g).

3.2. Preparation of tetraethyl (*E*)-11-methyldodeca-1,6-diene-4,4,9,9-tetracarboxylate (6a):

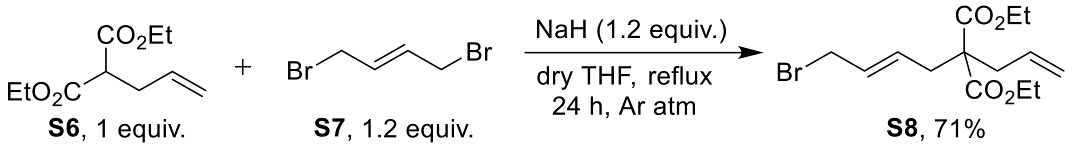

A 100 mL two-neck round-bottom flask, equipped with a magnetic stirrer, was placed in an oil bath and flushed with argon. A reflux condenser connected to an argon-filled balloon was attached to one of the necks. Sodium hydride (NaH 60% in mineral oil; 12 mmol, 1.2 equiv.) was carefully added to the flask under an argon atmosphere. The second neck was sealed with a septum. Freshly dried THF (50 mL) was added to the flask *via* a syringe, forming a suspension. Diethyl allyl malonate (2.0 g, 10 mmol, 1 equiv.) was added dropwise to the stirred NaH/THF mixture using a syringe. Then the reaction mixture was heated at 60 °C for 1 h to generate the enolate, cooled to room temperature, and (*E*)-1,4-dibromobut-2-ene (12 mmol, 1.2 equiv.) was added *via* syringe through the septum. Then, the reaction mixture was heated to reflux for 24 h under argon. After cooling to room temperature, the mixture was cautiously quenched with saturated aq. NH_4Cl and extracted with ethyl acetate (3 × 50 mL). The combined organic layers were dried over anhydrous Na_2SO_4, concentrated under reduced pressure, and purified by column chromatography using a hexane/ethyl acetate mixture as the eluent to obtain product **S8** in 71% yield (2.35 g).

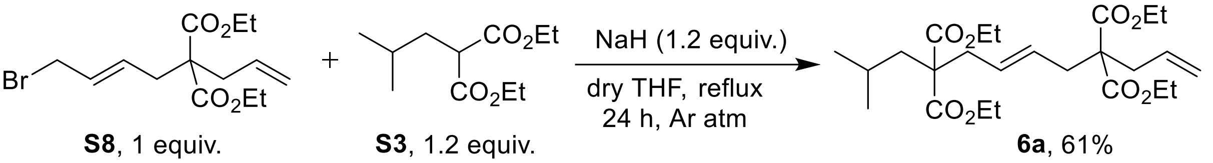

A 100 mL two-neck round-bottom flask, equipped with a magnetic stirrer, was placed in an oil bath and flushed with argon. A reflux condenser connected to an argon-filled balloon was attached to one of the necks. Sodium hydride (NaH 60% in mineral oil; 8.4 mmol, 1.2 equiv.) was carefully added to the flask under an argon atmosphere. The second neck was sealed with a septum. Freshly dried THF (50 mL) was added to the flask *via* a syringe, forming a suspension. Diethyl allyl malonate (**S3**, 7 mmol, 1 equiv.) was added dropwise to the stirred mixture using a syringe. Then the reaction mixture was heated at 60 °C for 1 h to generate the enolate, cooled to room temperature, and diethyl (*E*)-2-allyl-2-(4-bromobut-2-en-1-yl)malonate (**S8**, 8.4 mmol, 1.2 equiv.) was added *via* syringe through the septum. The reaction was heated to reflux for 24 h under argon. After cooling to room temperature, the mixture was cautiously quenched with saturated aq. NH_4Cl and extracted with ethyl acetate (3 × 50 mL). The combined organic layers were dried over anhydrous Na_2SO_4, concentrated under reduced pressure, and purified by column chromatography using a hexane/ethyl acetate mixture as the eluent to afford product **6a** in 61% yield (2.01 g).

3.3. Preparation of hexaethyl 14-methylpentadec-1-ene-4,4,8,8,12,12-hexacarboxylate (8a):

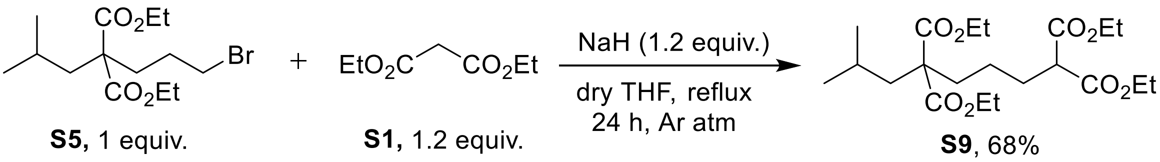

A 250 mL two-neck round-bottom flask, equipped with a magnetic stirrer, was placed in an oil bath and flushed with argon. A reflux condenser connected to an argon-filled balloon was attached to one of the necks. Sodium hydride (NaH 60% in mineral oil; 30 mmol, 1.2 equiv.) was carefully added to the flask under an argon atmosphere. The second neck was sealed with a septum. Freshly dried THF (100 mL) was added to the flask *via* a syringe, forming a suspension. Diethyl malonate (**S1**, 4.8 g, 30 mmol, 1.2 equiv.) was added dropwise to the stirred syringe under stirring conditions. Then, the reaction mixture was heated at 60 °C for 1 h to deprotonate S1 and generate the enolate fully. It was cooled to room temperature,

and diethyl 2-(3-bromopropyl)-2-isobutylmalonate (**S5**, 8.43 g, 25 mmol, 1 equiv.) was added *via* syringe through the septum. The reaction mixture was heated to reflux for 24 h under argon. After cooling to room temperature, the mixture was cautiously quenched with saturated aq. NH_4Cl and extracted with ethyl acetate (3 × 100 mL). The combined organic layers were dried over anhydrous Na_2SO_4, concentrated under reduced pressure, and purified by column chromatography using a hexane/ethyl acetate mixture as the eluent to afford the product **S9** in 68% yield (7.1 g).

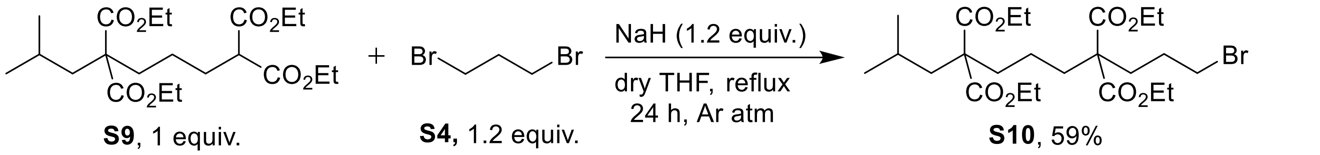

A 100 mL two-neck round-bottom flask, equipped with a magnetic stirrer, was placed in an oil bath and flushed with argon. A reflux condenser connected to an argon-filled balloon was attached to one of the necks. Sodium hydride (NaH 60% in mineral oil; 17 mmol, 1 equiv.) was carefully added to the flask under an argon atmosphere. The second neck was sealed with a septum. Freshly dried THF (50 mL) was added to the flask *via* a syringe, forming a suspension. Tetraethyl 7-methyloctane-1,1,5,5-tetracarboxylate (**S9**, 7.1 g, 17 mmol, 1 equiv.) was added dropwise to the stirred NaH/THF mixture using a syringe. The reaction mixture was heated at 60 °C for 1 h to deprotonate **S9** and generate the enolate fully. It was then cooled to room temperature, and 1,3-dibromopropane (20.4 mmol, 1.2 equiv.) was added *via* syringe through the septum. The reaction mixture was heated to reflux for 24 h under argon. After cooling to room temperature, the mixture was cautiously quenched with saturated aq. NH_4Cl and extracted with ethyl acetate (3 × 50 mL). The combined organic layers were dried over anhydrous Na_2SO_4, concentrated under reduced pressure, and purified by column chromatography using a hexane/ethyl acetate mixture as the eluent to obtain the product **S10** in 59% yield (5.4 g).

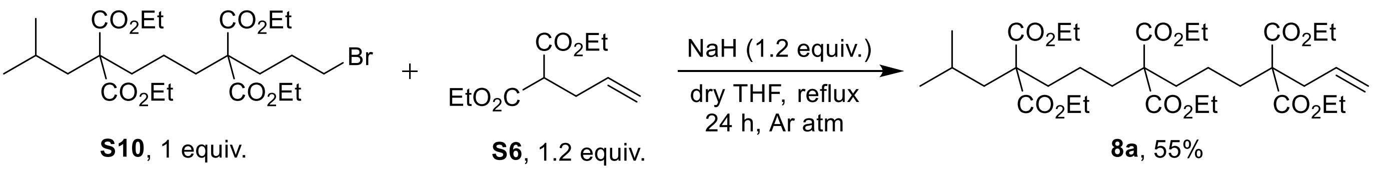

A 100 mL two-neck round-bottom flask, equipped with a magnetic stirrer, was placed in an oil bath and flushed with argon. A reflux condenser connected to an argon-filled balloon was attached to one of the necks. Sodium hydride (NaH 60% in mineral oil; 9 mmol, 1 equiv.) was carefully added under an argon atmosphere. The second neck was sealed with a septum. Freshly dried THF (50 mL) was added to the flask *via* a syringe, forming a suspension. Compound **S6** (10.8 mmol, 1.2 equiv.) was added dropwise to the stirred NaH/THF mixture using a syringe. The reaction mixture was heated at 60 °C for 1 h to deprotonate **S6** and generate the enolate fully. It was then cooled to room temperature, and compound **S10** (4.8 g, 9 mmol, 1 equiv.) was added *via* syringe through the septum. The reaction mixture was heated to reflux for 24 h under argon. After cooling to room temperature, the mixture was cautiously quenched with saturated aq. NH_4Cl and extracted with ethyl acetate (3 × 50 mL). The combined organic layers were dried over anhydrous Na_2SO_4, concentrated under reduced pressure, and purified by column chromatography using a hexane/ethyl acetate mixture as the eluent to afford product **8a** in 55% yield (3.2 g).

3.4. General procedure for the synthesis of 10:

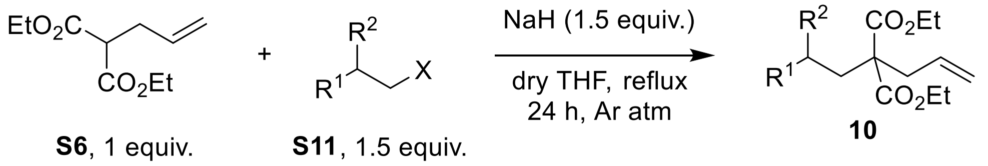

A 100 mL two-neck round-bottom flask, equipped with a magnetic stirrer, was placed in an oil bath and flushed with argon. A reflux condenser connected to an argon-filled balloon was attached to one neck, and the other neck was sealed with a

septum. Sodium hydride (NaH 60% in mineral oil; 15 mmol, 1.5 equiv.) was quickly transferred into the flask under an argon atmosphere. The flask was flushed with argon, and the septum was secured. Freshly dried THF (50 mL) was added *via* syringe, followed by dropwise addition of diethyl allyl malonate (**S6**, 2.0 g, 10 mmol, 1 equiv.) using a syringe. The suspension was stirred and heated to 60 °C for 1 h. After cooling to room temperature, the appropriate alkyl halide (**S11**, iodide or bromide, 15 mmol, 1.5 equiv.) was added *via* a syringe through the septum. The septum was replaced with a glass stopper under continuous argon flow, and the mixture was heated to reflux for 24 h. After cooling to room temperature, the reaction was quenched by carefully adding a saturated aq. NH_4Cl solution. The mixture was extracted with ethyl acetate (3 × 50 mL), and the combined organic layers were dried over anhydrous Na_2SO_4. The mixture was then concentrated under reduced pressure and purified by column chromatography using a hexane/ethyl acetate mixture as the eluent, affording the product **10**.

3.5. Preparation of diethyl 2-allyl-2-(2-methylpropyl-2-*D*)malonate (D2-10g):

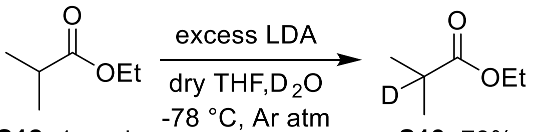

S12, 1 equiv. **S13**, 78%

A 2 L two-neck round-bottom flask, equipped with a magnetic stirring bar, was fitted with an adapter connected to an argon-filled balloon *via* one of its necks. The system was purged with argon (3 times), and the second neck was sealed with a glass stopper, ensuring the setup was airtight. The flask was immersed in a cooling bath at 0 °C. Freshly dried hexane (100 mL) and freshly distilled diisopropyl amine (215 mmol, 1.2 equiv.) were added *via* syringe. After equilibrating at 0 °C, ^{n}BuLi (2.5 M in hexane, 215 mmol, 1.2 equiv.) was added dropwise under vigorous stirring. The mixture was stirred for 10 minutes, and the hexane was evaporated under reduced pressure. The residue (solid LDA) was backfilled with argon and redissolved in freshly dried THF (600 mL). The solution was cooled to –78 °C, and a solution of ethylisobutyrate in anhydrous THF (100 ml) was added dropwise with continuous stirring. After 1 h, a second portion of ^{n}BuLi (2.5 M in hexane, 215 mmol, 1.2 equiv.) was added slowly, and stirring continued for 30 mins. The reaction mixture was quenched by dropwise addition of D_2O (538 mmol, 3 equiv.) in anhydrous THF 9100 mL) at –78 °C. The cooling bath was removed, and the mixture was allowed to warm to room temperature. Diethyl ether (300 mL)and 6 N HCl (100 mL) were added, and the mixture was transferred to a separatory funnel. After vigorous shaking, the solution was allowed to settle down. The organic layers were collected and washed sequentially with 3 N HCl (50 mL), saturated aq. $NaHCO_3$ (50 mL) and brine (50 mL) solution, then dried over anhydrous Na_2SO_4. The product was isolated *via* fractional distillation under atmospheric pressure at 120 °C (reported boiling point 113 °C). The target molecule, ethyl 2-methylpropanoate-2-D, was obtained in 78% yield (with a minor amount of diethyl ether impurity) and 98% ${}^{2}H$ purity, as determined by ${}^{1}H$ NMR.

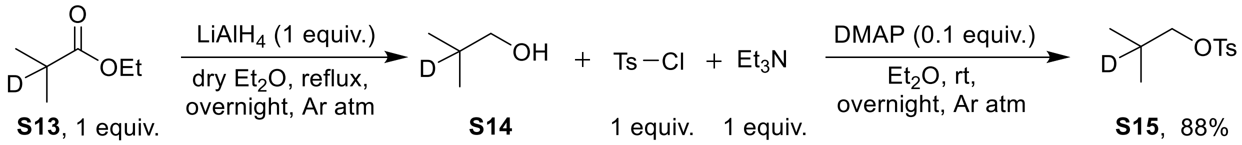

S13, 1 equiv. **S14** 1 equiv. 1 equiv. **S15**, 88%

A 250 mL two-neck round-bottom flask, equipped with a magnetic stirrer, was fitted with a reflux condenser connected to an argon-filled balloon. $LiAlH_4$ (50 mmol, 1 equiv.) was added to the flask, which was then flushed with argon and sealed with a septum. Freshly dried diethyl ether (75 mL) was added, and the reaction mixture was cooled to 0 °C using an ice bath. Ethyl 2-deutero-2-methylpropanoate (**S13**, 5.85 g, 50 mmol, 1 equiv.) was added dropwise *via* a syringe under stirring. After complete addition, the reaction mixture was allowed to warm to room temperature and heated under reflux. The reaction was stirred overnight under reflux, cooled to room temperature, and then to 0 °C. It was cautiously quenched by the dropwise addition of a saturated aq. Na_2SO_4 until a white precipitate persisted. The precipitate was filtered and washed with diethyl ether (3 x 50 mL). The combined organic layers were dried over anhydrous Na_2SO_4 overnight. The resulting diethyl ether solution of 2-deuterated isopropanol **S14** was carried forward for tosylation. The diethyl ether solution of **S14** was transferred to a 250 mL two-neck round-bottom flask equipped with a magnetic stirrer and an argon balloon. Triethyl amine (50 mmol, 1 equiv.) and DMAP (5 mmol, 0.1 equiv.) were added, and the mixture was cooled to 0 °C using an ice

bath. *p*-Toluenesulfonyl chloride (9.5 g, 50 mmol, 1 equiv.) was added portion-wise under argon with vigorous stirring. The second neck of the flask was sealed with a glass stopper. The reaction mixture was allowed to warm to room temperature and stirred overnight. The mixture was quenched with 0.1 N HCl (100 mL), and the organic layer was separated. The aqueous layer was extracted with DCM (3 × 50 mL). The combined layers were dried over anhydrous Na_2SO_4, and the solvent was removed under reduced pressure. The crude was purified by column chromatography using hexane/ethyl acetate as eluent to afford 2-methylpropyl-2-*D* 4-methylbenzenesulfonate (**S15**) in 88% yield (10.1 g).

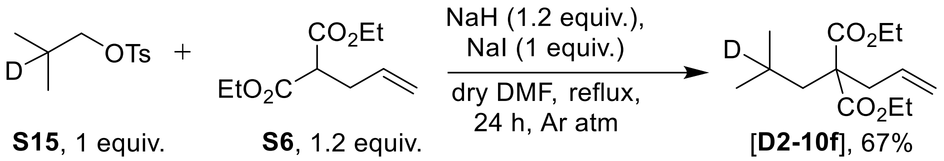

A 100 mL two-neck round-bottom flask equipped with a magnetic stirrer was placed in an oil bath. A reflux condenser connected to an argon-filled balloon was attached to the flask. NaH (60% in mineral oil) (6 mmol, 1.2 equiv.) was added to the flask, which was then flushed with argon and sealed with a septum. Freshly dried DMF (30 mL) was added to the flask, followed by the dropwise addition of diethyl allyl malonate (**S6**, 5 mmol, 1 equiv.) through a syringe under stirring. The reaction mixture was heated at 60 °C for 1 h and then allowed to cool to room temperature. Next, 2-methylpropyl-2-*D* 4-methylbenzenesulfonate (**S15**, 1.0 g, 5 mmol, 1 equiv.) and NaI (5 mmol, 1 equiv.) were added to the reaction mixture at room temperature. The septum was replaced with a stopper under argon flushing, and the mixture was heated to reflux for 24 h. After cooling to room temperature, the reaction was quenched with a saturated aq. NH_4Cl solution. The mixture was extracted with ethyl acetate (3 × 30 mL), and the combined organic layers were dried over anhydrous Na_2SO_4. The solvent was removed under reduced pressure, and the crude product was purified by column chromatography using a hexane/ethyl acetate mixture as eluent to furnish **D2-10f** in 67% yield (0.86 g).

4. Experimental procedure for synthesizing compounds 3, 7, 9a, 11, 14:

4.1. Experimental procedure for synthesizing compounds 3:

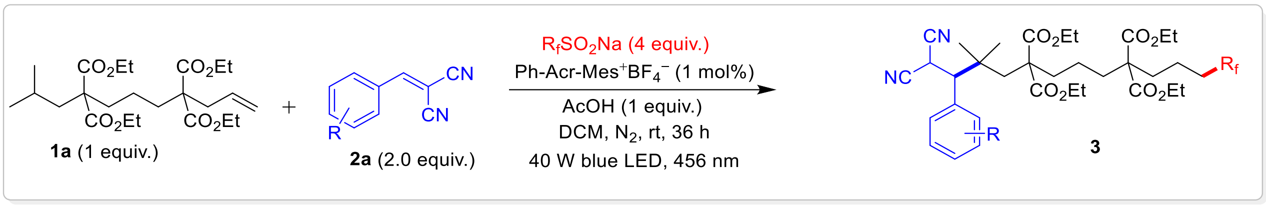

An oven-dried 20 mL reaction tube equipped with a magnetic stirring bar was charged with **1a** (68.3 mg, 0.15 mmol, 1 equiv.), **2** (0.3 mmol, 2 equiv.), Ph-Mes-$Acr^+BF_4^-$ (0.69 mg, 0.0015 mmol, 1 mol%) and R_fSO_2Na (0.6 mmol, 4 equiv.). The reaction tube was vacuumed, backfilled with nitrogen (3 cycles), and fitted with a septum. Dry DCM (2 mL) and AcOH (0.15 mmol, 1 equiv.) were added sequentially *via* a syringe through the septum. The reaction tube was positioned ~3 cm away from a Kessil 40 W 456 nm LED setup, and a cooling fan was placed ~30 cm away to maintain ambient temperature. After stirring for 36 h under irradiation, the reaction was quenched with water (10 mL) and extracted with DCM (3 x 20 mL). The combined organic layers were dried over anhydrous Na_2SO_4, and the solvent was evaporated under reduced pressure. The crude product was purified by flash column chromatography (230-400 mesh silica gel, hexane/ethyl acetate gradient) to afford product **3.**

4.2. Experimental procedure for synthesizing compounds 7:

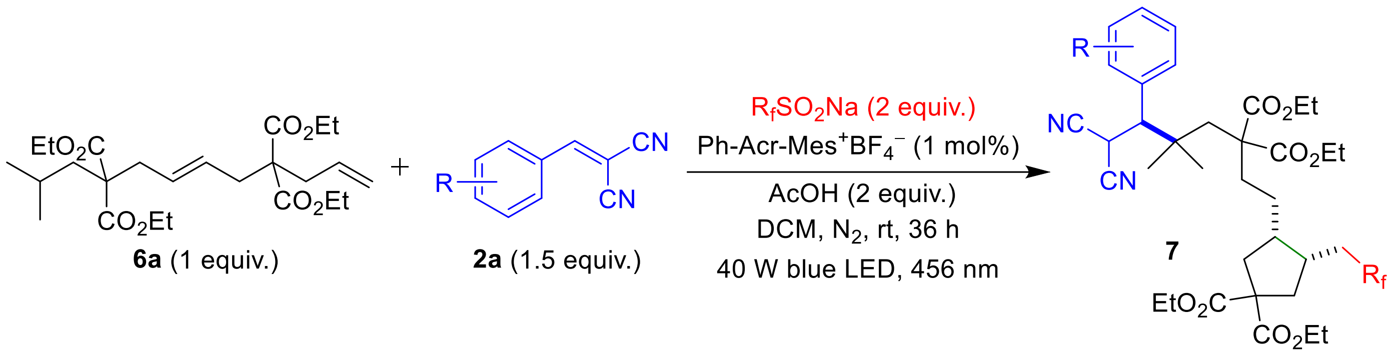

An oven-dried 20 mL reaction tube equipped with a magnetic stirring bar was charged with **6a** (70.1 mg, 0.15 mmol, 1 equiv.), **2** (0.23 mmol, 1.5 equiv.), Ph-Mes-Acr$^+$BF$_4^-$ (0.69 mg, 0.0015 mmol, 1 mol%) and R$_f$SO$_2$Na (0.3 mmol, 2 equiv.). The reaction tube was vacuumed and backfilled with nitrogen (3 cycles) and fitted with a septum. Dry DCM (2 mL) and AcOH (0.3 mmol, 2 equiv.) were added sequentially *via* a syringe through the septum. The reaction tube was positioned ~3 cm away from a Kessil 40 W 456 nm LED setup, and a cooling fan was placed ~30 cm away to maintain ambient temperature. After stirring for 36 h under irradiation, the reaction was quenched with water (10 mL) and extracted with DCM (3 x 20 mL). The combined organic layers were dried over anhydrous Na$_2$SO$_4$, and the solvent was evaporated under reduced pressure. The crude product was then purified by flash column chromatography (230-400 mesh silica gel, hexane/ethyl acetate gradient) to afford product **7.**

4.3. Experimental procedure for synthesizing compounds 9a:

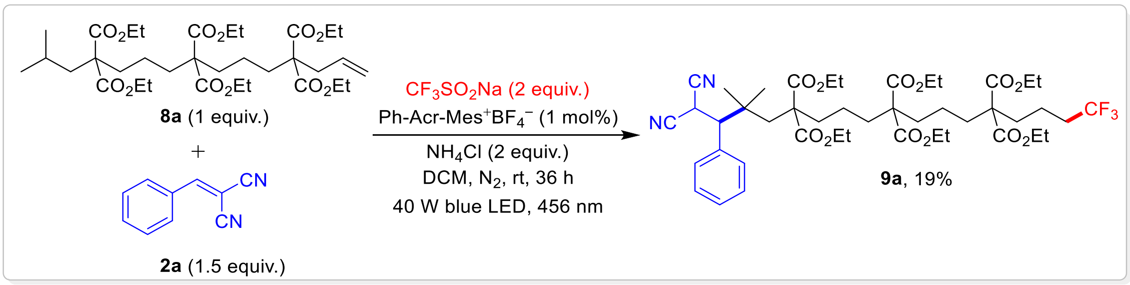

An oven-dried 20 mL reaction tube equipped with a magnetic stirring bar was charged with **8a** (98.5 mg, 0.15 mmol, 1 equiv.), **2a** (46.2 g, 0.3 mmol, 1.5 equiv.), Ph-Mes-Acr$^+$BF$_4^-$ (0.69 mg, 0.0015 mmol, 1 mol%) and CF$_3$SO$_2$Na (45.6 mg, 0.3 mmol, 2 equiv.). The reaction tube was vacuumed and backfilled with nitrogen (3 cycles) and fitted with a septum. Dry DCM (2 mL) and NH$_4$Cl (0.3 mmol, 2 equiv.) were sequentially added. The reaction tube was positioned ~3 cm away from a Kessil 40 W 456 nm LED setup, and a cooling fan was placed ~30 cm away to maintain ambient temperature. After stirring for 36 h under irradiation, the reaction was quenched with water (10 mL) and extracted with DCM (3 x 20 mL). The combined organic layers were dried over Na$_2$SO$_4$, and the solvent was evaporated under reduced pressure. The crude product was then purified by flash column chromatography (230-400 mesh silica gel, hexane/ethyl acetate gradient) to afford the product **9a** in 19% yield (25 mg).

4.4. Experimental procedure for synthesizing compounds 11:

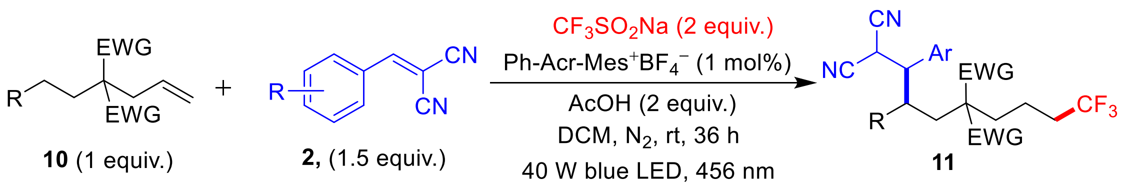

An oven-dried 20 mL reaction tube equipped with a magnetic stirring bar was charged with **10** (0.15 mmol, 1 equiv.), **2a** (0.23 mmol, 1.5 equiv.), Ph-Mes-Acr$^+$BF$_4^-$ (0.69 mg, 0.0015 mmol, 1 mol%) and CF_3SO_2Na (45.6 mg, 0.3 mmol, 2 equiv.). The reaction tube was vacuumed and backfilled with nitrogen (3 cycles) and fitted with a septum. Dry DCM (2 mL) and AcOH (0.3 mmol, 2 equiv.) were added sequentially *via* a syringe through the septum. The reaction tube was positioned ~3 cm away from a Kessil 40 W 456 nm LED setup, and a cooling fan was placed ~30 cm away to maintain ambient temperature. After stirring for 36 h under irradiation, the reaction was quenched with water (10 mL) and extracted with DCM (3 x 20 mL). The combined organic layers were dried over Na_2SO_4, and the solvent was evaporated under reduced pressure. The crude product was then purified by flash column chromatography (230-400 mesh silica gel, hexane/ethyl acetate gradient) to afford product **11**.

4.5. Experimental procedure for synthesizing compounds 14:

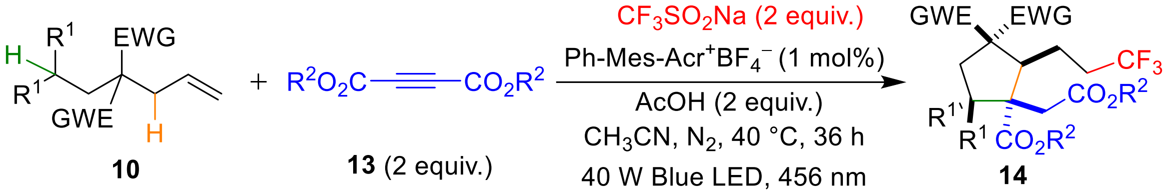

An oven-dried 20 mL reaction tube equipped with a magnetic stirring bar was charged with **10** (0.15 mmol, 1 equiv.), Ph-Mes-Acr$^+$BF$_4^-$ (0.69 mg, 0.0015 mmol, 1 mol%) and CF_3SO_2Na (45.6 mg, 0.3 mmol, 2 equiv.). The reaction tube was vacuumed and backfilled with nitrogen (3 cycles) and fitted with a septum. Dry CH_3CN (2 mL), **13** (0.3 mmol, 2 equiv.), and AcOH (0.3 mmol, 2 equiv.) were added sequentially *via* a syringe through the septum. The reaction tube was immersed in a 40 °C water bath (250 mL glass beaker) and positioned ~3 cm away from a Kessil 40 W 456 nm LED setup. After stirring for 36 h under irradiation, the reaction was quenched with water (10 mL) and extracted with DCM (3 x 20 mL). The combined organic layers were dried over anhydrous Na_2SO_4, and the solvent was evaporated under reduced pressure. The crude product was then purified by flash column chromatography (230-400 mesh silica gel, using a hexane/ethyl acetate gradient) to afford product **14**.

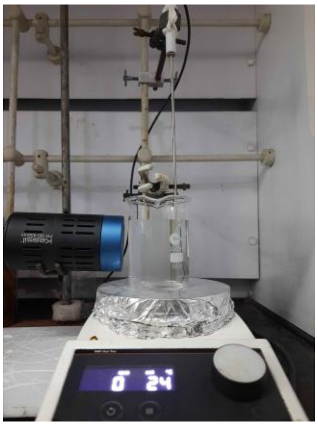

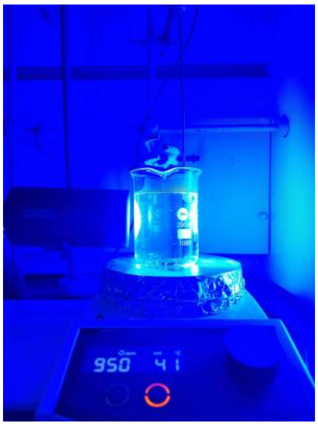

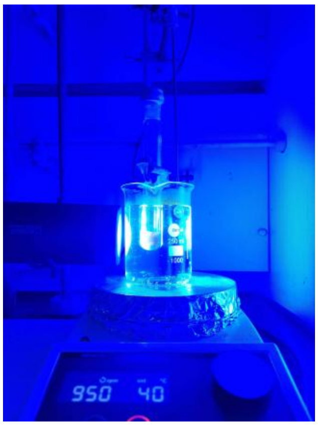

5.1. Gram-scale synthesis of compound 11f:

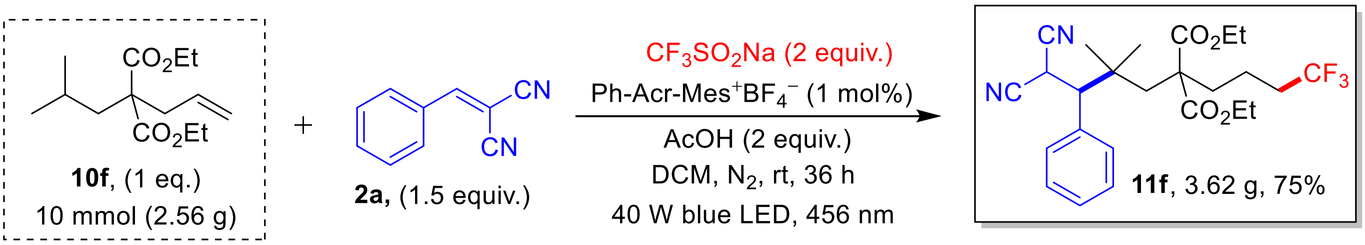

An oven-dried 100 mL two-neck round-bottom flask equipped with a magnetic stirring bar was charged with **10f** (2.56 g, 10 mmol, 1 equiv.), **2a** (2.31 g, 15 mmol, 1.5 equiv.), Ph-Mes-Acr$^+$BF$_4^-$ (46.1 mg, 0.1 mmol, 1 mol%) and CF_3SO_2Na (3.04 g,

20 mmol, 2 equiv.). After fitting a septum on one neck and an adapter with a stopcock on the other, the reaction flask was vacuumed and backfilled with nitrogen (5 cycles). A nitrogen balloon was attached to the adapter, and dry DCM (30 mL) and AcOH (20 mmol, 2 equiv.) were added sequentially *via* a syringe through the septum. Then, the septum was replaced with a stopper, and the flask was placed ~3 cm away from two Kessil 40 W 456 nm LED setups and ~30 cm away from a cooling fan to maintain ambient temperature. After stirring for 36 h under irradiation, the reaction was quenched with water (50 mL) and extracted with DCM (3 x 50 mL). The combined organic layers were dried over Na_2SO_4, and the solvent was evaporated under reduced pressure. The crude product was then purified by flash column chromatography (230-400 mesh silica gel, 20% ethyl acetate/hexane as the eluent) to afford the yellowish oil product **11f** in 75% yield (3.62 g).

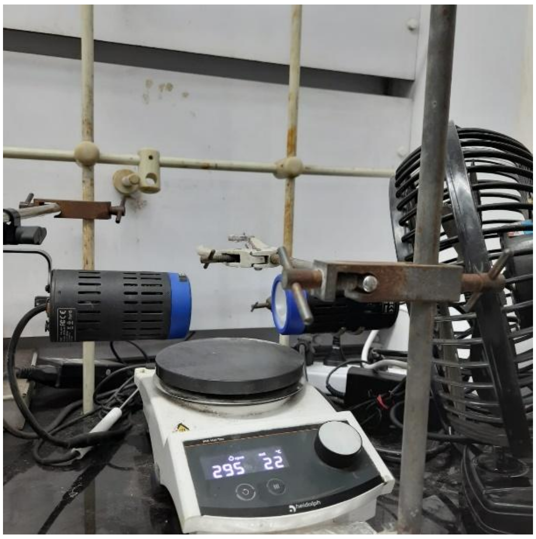

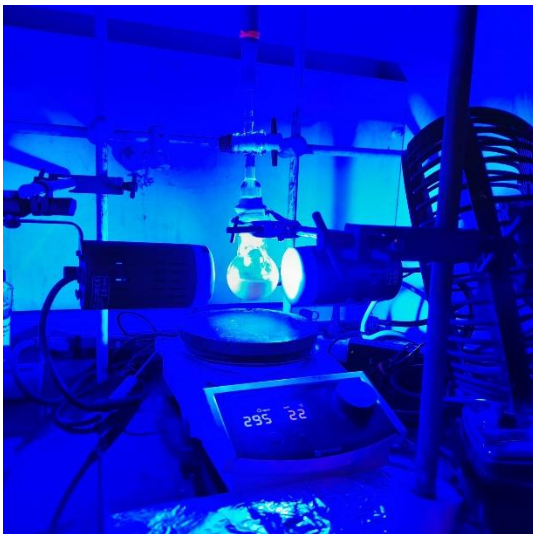

5.2. Gram-scale synthesis of compound 14a:

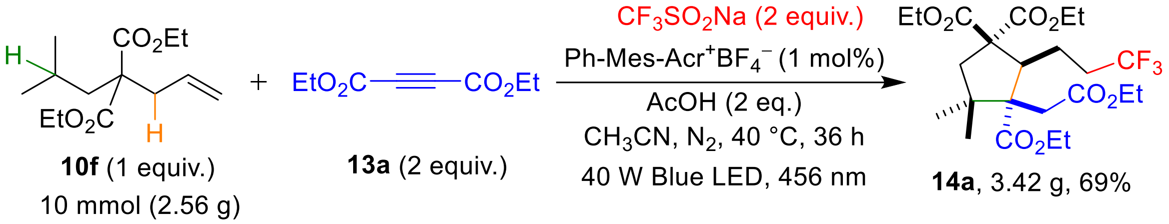

An oven-dried 100 mL two-neck round-bottom flask equipped with a magnetic stirring bar was charged with **10f** (2.56 g, 10 mmol, 1 equiv.), **13** (3.4 g, 20 mmol, 2 equiv.), Ph-Mes-Acr$^+$BF$_4^-$ (0.1 mmol, 1 mol%) and CF_3SO_2Na (4.56 g, 30 mmol, 2 equiv.). After fitting a septum on one neck and an adapter with a stopcock on the other, the reaction flask was vacuumed and backfilled with nitrogen (5 cycles). A nitrogen balloon was attached to the adapter, and 30 mL of dry CH_3CN, followed by AcOH (1.1 mL, 20 mmol, 2 equiv.), were added through a syringe. The septum was replaced with a stopper, and the flask was placed ~3 cm away from two Kessil 40 W 456 nm LED lamps and ~30 cm away from a cooling fan to maintain room temperature. After stirring for 36 h under irradiation, the reaction was quenched with water (50 mL) and extracted with DCM (3 x 50 mL). The combined organic layers were dried over Na_2SO_4, and the solvent was evaporated under reduced pressure. The crude product was then purified by flash column chromatography (230-400 mesh silica gel, 10% ethyl acetate/hexane as the eluent) to afford the yellowish oil product **14a** in 69% yield (3.42 g).

6. Procedure for further functionalization of the product 11f:

6.1. Synthesis of diethyl (S)-2-(4-(allylamino)-2,2-dimethyl-4-oxo-3-phenylbutyl)-2-(4,4,4-trifluorobutyl)malonate (15):

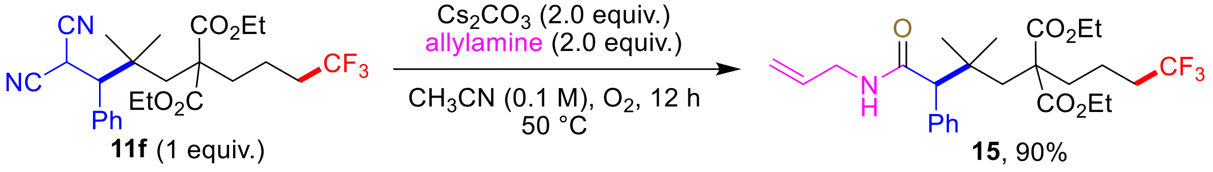

An oven-dried 20 mL reaction tube equipped with a magnetic stirring bar was charged with **11f** (96.1 mg, 0.2 mmol, 1.0 equiv.) and Cs_2CO_3 (130.3 mg, 0.4 mmol, 2.0 equiv.). The reaction tube was evacuated under vacuum and then backfilled with oxygen, and this process was repeated four times. Subsequently, dry acetonitrile (2 mL) and allyl amine (22.8 mg, 0.4 mmol, 2.0 equiv.) were added *via* a syringe. This mixture was placed on a magnetic stirrer with continuous O_2 bubbling (delivered through a long needle connected to an oxygen balloon) at room temperature. The reaction was heated to 50 °C and stirred for 12 h. After cooling to room temperature, the reaction was quenched with water (10 mL) and extracted with DCM (3 × 20 mL). The combined organic layers were dried over anhydrous Na_2SO_4, filtered, and the solvent was evaporated under reduced pressure. The crude product was purified by flash column chromatography (230-400 mesh silica gel, hexane/ethyl acetate gradient) to afford the product **15** in 90% yield (89.9 mg).

6.2. Synthesis of diethyl (S)-2-(2,2-dimethyl-4-oxo-3-phenyl-4-(piperidin-1-yl)butyl)-2-(4,4,4-trifluorobutyl)malonate (16):

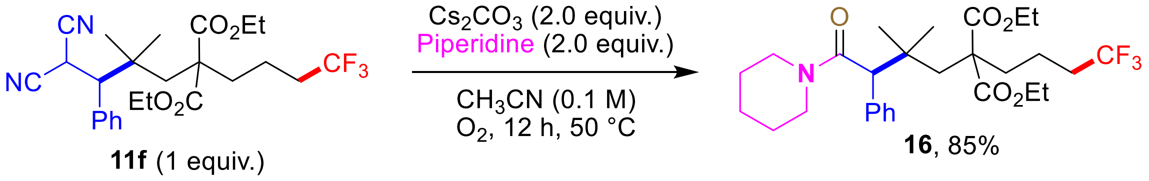

An oven-dried 20 mL reaction tube equipped with a magnetic stirring bar was charged with **11f** (96.1 mg, 0.2 mmol, 1.0 equiv.) and Cs_2CO_3 (130.3 mg, 0.4 mmol, 2.0 equiv.). The reaction tube was evacuated under vacuum and then backfilled with oxygen, a cycle that was repeated four times. Subsequently, dry acetonitrile (2 mL) and piperidine (34 mg, 0.4 mmol, 2.0 equiv.) were added *via* a syringe. This mixture was placed on a magnetic stirrer with continuous O_2 bubbling (delivered through a long needle connected to an oxygen balloon) at room temperature. The reaction was heated to 50 °C and stirred for 12 h. After cooling to room temperature, the reaction was quenched with water (10 mL), and extracted with DCM (3 × 20 mL). The combined organic layers were dried over anhydrous Na_2SO_4, filtered, and the solvent was evaporated under reduced pressure. The crude product was then purified by flash column chromatography (230-400 mesh silica gel, hexane/ethyl acetate gradient) to afford the product **16** in 85% yield (89.7 mg).

6.3. Synthesis of diethyl (S)-2-(5-amino-4-carbamoyl-2,2-dimethyl-5-oxo-3-phenylpentyl)-2-(4,4,4-trifluorobutyl) malonate (17):

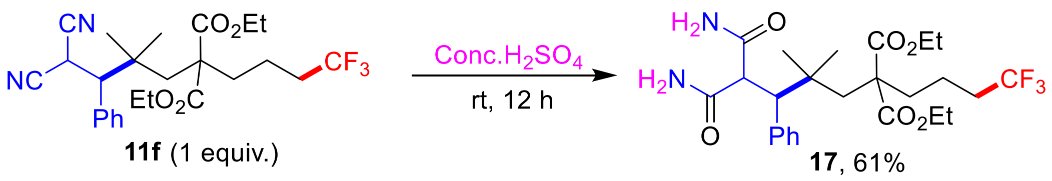

An oven-dried 20 mL reaction tube equipped with a magnetic stirring bar was charged with **11f** (96.1 mg, 0.2 mmol, 1.0 equiv.), 1.0 mL of concentrated sulphuric acid. The reaction tube was fitted with a rubber septum. This mixture was placed on a magnetic stirrer and stirred for 12 h at room temperature. The reaction was quenched by pouring into cold water (10 mL) and extracted with DCM (3 × 20 mL). The combined organic layers were dried over anhydrous Na_2SO_4, filtered, and the solvent was evaporated under reduced pressure. The crude product was purified by flash column chromatography (230-400 mesh silica gel, hexane/ethyl acetate gradient) to afford product **17** in 61% yield (63 mg).

6.4. Synthesis of diethyl (S)-2-(4,4-dicyano-2,2-dimethyl-3-phenylhept-6-en-1-yl)-2-(4,4,4-trifluorobutyl)malonate (18):

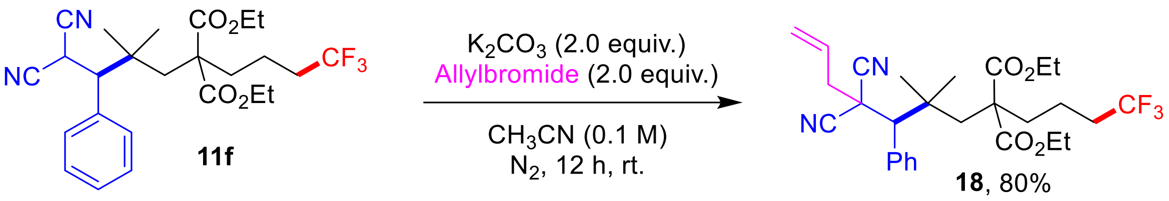

An oven-dried 20 mL reaction tube equipped with a magnetic stirring bar was charged with **11f** (96.1 mg, 0.2 mmol, 1.0 equiv.), K_2CO_3 (55.2 mg, 0.4 mmol, 2.0 equiv.), 2 mL of acetonitrile, and allyl bromide (0.4 mmol, 2.0 equiv.). The reaction tube was fitted with a rubber septum and placed on a magnetic stirrer. The reaction mixture was stirred at room temperature for 12 h. After completion, the reaction was quenched with water (10 mL), and extracted with DCM (3 × 20 mL). The combined organic layers were dried over anhydrous Na_2SO_4, filtered, and the solvent was evaporated under reduced pressure. The crude product was then purified by flash column chromatography (230-400 mesh silica gel, hexane/ethyl acetate gradient) to afford the product **18** in 80% yield (83.2 mg).

6.5. Synthesis of 4,4-diethyl 1-methyl (S)-8,8,8-trifluoro-2,2-dimethyl-1-phenyloctane-1,4,4-tricarboxylatemalonate (19):

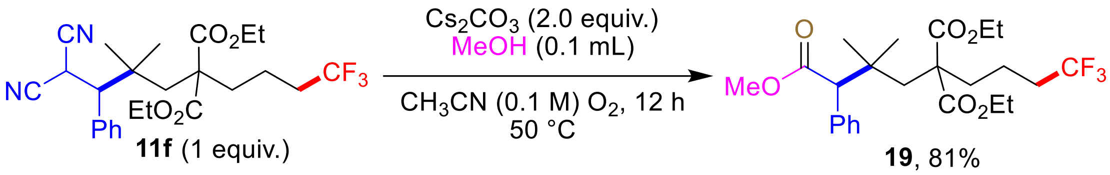

An oven-dried 20 mL reaction tube equipped with a magnetic stirring bar was charged with **11f** (96.1 mg, 0.2 mmol, 1 equiv.), and K_2CO_3 (55.2 mg, 0.4 mmol, 2 equiv.). The reaction tube was sealed with a septum after being evacuated under vacuum and backfilled with oxygen (3 cycles). Subsequently, dry acetonitrile (2 mL) and MeOH (0.1 mL) were added through the septum by a syringe and placed on a magnetic stirrer with continuous O_2 bubbling (delivered through a long needle connected to an oxygen balloon) at room temperature. After stirring for 12 h, the reaction was quenched with water (10 mL), and extracted with DCM (3 x 20 mL). The combined organic layers were dried over anhydrous Na_2SO_4, and the solvent was evaporated under reduced pressure. The crude product was then purified by flash column chromatography (230-400 mesh silica gel, hexane/ethyl acetate gradient) to afford the product **19** in 81% yield (76.8 mg).

6.6. Synthesis of diethyl 2-((3S)-4-cyano-4-((diphenylphosphoryl)oxy)-2,2-dimethyl-3-phenylbutyl)-2-(4,4,4-trifluoro butyl)malonate (20):

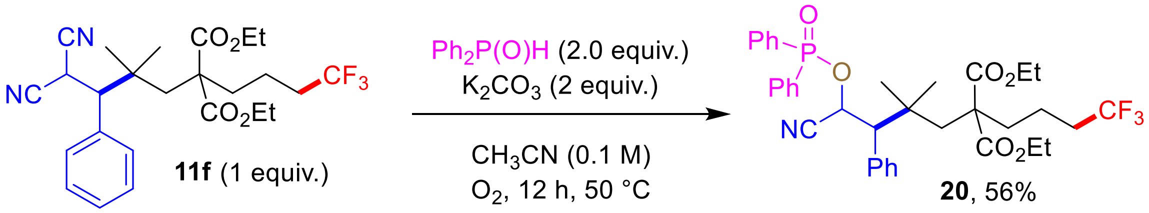

An oven-dried 20 mL reaction tube equipped with a magnetic stirring bar was charged with **11f** (96.1 mg, 0.2 mmol, 1 equiv.), DPPO (80.9 mg, 0.4 mmol, 2 equiv.) and K_2CO_3 (55.2 mg, 0.4 mmol, 2 equiv.). The reaction tube was evacuated under vacuum, backfilled with oxygen (3 cycles), and sealed with a septum. To this mixture, dry acetonitrile (2 mL) was added *via* a syringe and placed on a magnetic stirrer with continuous O_2 bubbling (delivered through a long needle connected to an oxygen balloon) at room temperature. The reaction was heated to 50 °C and stirred for 12 h. The reaction mixture was cooled to room temperature, quenched with water (10 mL), and extracted with DCM (3 × 20 mL). The combined organic layers were dried over anhydrous Na_2SO_4, and the solvent was evaporated under reduced pressure. The crude product was then purified by flash column chromatography (230-400 mesh silica gel, hexane/ethyl acetate gradient) to afford the product **20** in 56% yield (75.2 mg).

7. Control experiments:

7.1. Reaction without photocatalyst:

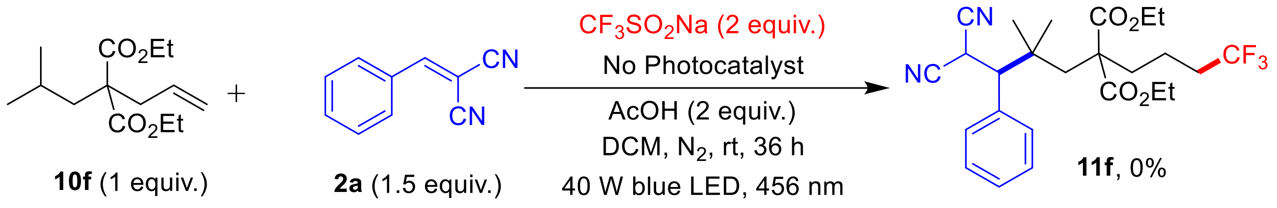

An oven-dried 20 mL reaction tube equipped with a magnetic stir bar was charged with **10f** (38.4 mg, 0.15 mmol, 1 equiv.), **2a** (0.23 mmol, 1.5 equiv.) and CF_3SO_2Na (45.6 mg, 0.3 mmol, 2 equiv.). The reaction tube was evacuated under vacuum for 3 minutes and backfilled with nitrogen; this cycle was repeated three times. The tube was securely sealed with a rubber septum under continuous nitrogen flow. Freshly dried DCM (2 mL) and AcOH (18 mg, 0.3 mmol, 2 equiv.) were added *via* a syringe. The mixture was placed on a magnetic stirrer and irradiated with a Kessil 40 W, 456 nm blue LED, positioned ~3 cm from the reaction tube. A cooling fan was placed ~30 cm away to maintain the system at room temperature. After irradiation for 24 h, the reaction mixture was diluted with DCM (5 mL), followed by the addition of brine solution (10 mL). The aqueous layer was extracted with DCM (3 × 20 mL), and the combined organic layers were dried over anhydrous Na_2SO_4, filtered, and the solvent was evaporated under reduced pressure. ^1H NMR analysis of the crude sample (using tetrachloroethane as an internal standard) revealed no detectable formation of the desired product **11f**. This result confirms the essential role of the photocatalyst in the transformation.

7.2. Reaction without light:

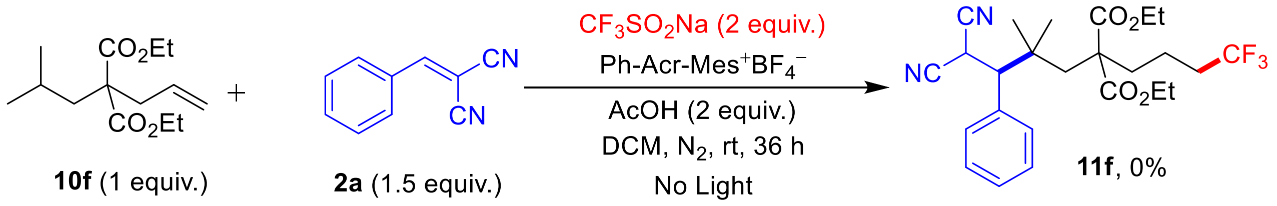

An oven-dried 20 mL reaction tube equipped with a magnetic stir bar was charged with **10g** (38.4 mg, 0.2 mmol, 1 equiv.), **2a** (46.2 mg, 0.3 mmol, 1.5 equiv.) CF_3SO_2Na (45.6 mg, 0.4 mmol, 2 equiv.), and Ph-Mes-Acr$^+BF_4^-$ (0.69 mg, 0.0015 mmol, 1 mol%). The reaction tube was evacuated under vacuum for 3 minutes and backfilled with nitrogen; this cycle was repeated three times. The tube was securely sealed with a rubber septum under continuous nitrogen flow. Freshly dried DCM (2 mL) and AcOH (xx mL, 0.3 mmol, 2 equiv.) were added *via* a syringe. The mixture was placed on a magnetic stirrer, and the reaction was conducted in the absence of light (no LED irradiation). After irradiation for 24 h, the reaction mixture was diluted with DCM (5 mL), followed by the addition of brine solution (10 mL). The aqueous layer was extracted with DCM (3 × 20 mL). The combined organic layers were dried over anhydrous Na_2SO_4, filtered, and the solvent was evaporated under reduced pressure. ^1H analysis of the crude sample (using tetrachloroethane as an internal standard) confirmed the absence of the desired product **11f**, demonstrating the necessity of light for this transformation.

7.3. Reaction with TEMPO/BHT:

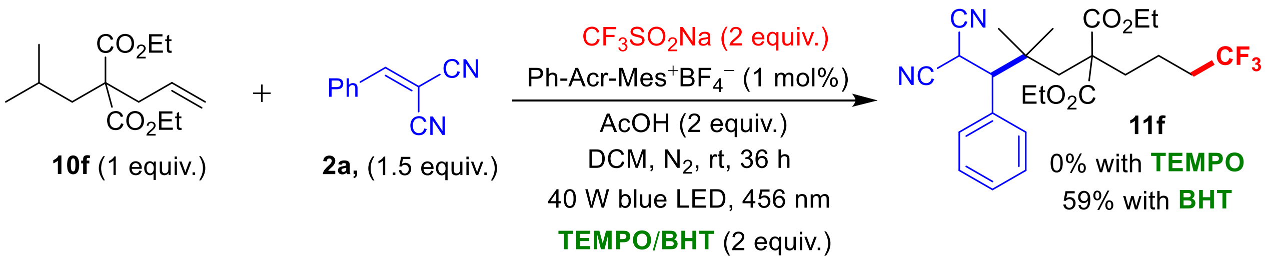

An oven-dried 20 mL reaction tube equipped with a magnetic stir bar was charged with **1a** (38.4 mg, 0.2 mmol, 1 equiv.), CF_3SO_2Na (0.4 mmol, 2 equiv.), Ph-Mes-Acr$^+BF_4^-$ (0.69 mg, 0.0015 mmol, 1 mol%), and TEMPO (62.5 mg, 0.4 mmol, 2 equiv.) were added. The reaction tube was evacuated under vacuum for 3 minutes and backfilled with nitrogen; this cycle was

repeated three times. The tube was securely sealed with a rubber septum under continuous nitrogen flow. Freshly dried DCM (2 mL) and AcOH (18 mg, 0.4 mmol, 2 equiv.) were added *via* a syringe. The mixture was placed on a magnetic stirrer and irradiated with a Kessil 40 W, 456 nm blue LED, positioned ~3 cm from the reaction tube. A cooling fan was placed ~30 cm away to maintain the system at room temperature. After irradiation for 24 h, the reaction mixture was diluted with DCM (5 mL), followed by the addition of brine solution (10 mL), and then extracted with DCM (3 × 20 mL). The combined organic layers were dried over anhydrous Na_2SO_4, filtered, and the solvent was evaporated under reduced pressure. ^1H analysis of the crude sample (using tetrachloroethane as an internal standard) confirmed no desired product **11f** formation. When the reaction was performed with 2 equiv. of BHT, a diminished yield of **11f** was observed. These two experiments prove that the reaction proceeds through the radical mechanism.

7.4. Radical clock experiment:

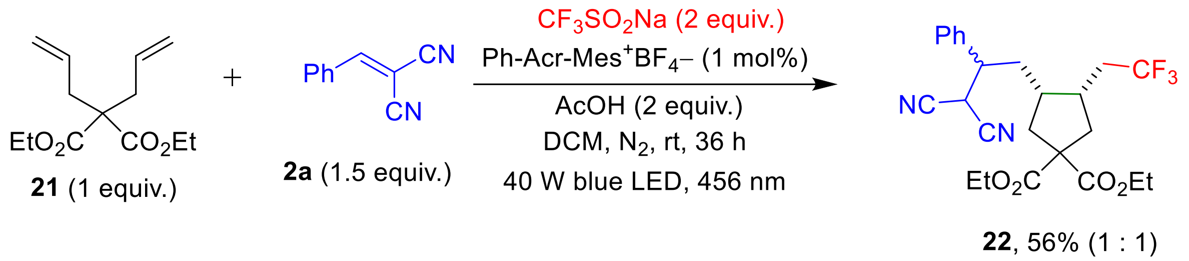

An oven-dried 20 mL reaction tube equipped with a magnetic stirring bar was charged with **21** (36 mg, 0.15 mmol, 1 equiv.), **2a** (46.2 mg, 0.23 mmol, 1.5 equiv.), Ph-Mes-Acr$^+$BF$_4^-$ (0.69 mg, 0.0015 mmol, 1 mol%) and CF_3SO_2Na (45.2 mg, 0.3 mmol, 2 equiv.). The reaction tube was evacuated under vacuum and backfilled with nitrogen; this cycle was repeated three times. The reaction tube was securely sealed with a rubber. Dry DCM (2 mL) and AcOH (0.3 mmol, 2 equiv.) were added *via* a syringe through the septum. The mixture was placed on a magnetic stirrer and irradiated with a Kessil 40 W, 456 nm blue LED, positioned ~3 cm from the reaction tube. A cooling fan was placed ~30 cm away to maintain the system at room temperature. After stirring for 36 h under irradiation, the reaction was diluted with water (10 mL) and extracted with DCM (3 x 20 mL). The combined organic layers were dried over anhydrous Na_2SO_4, filtered, and the solvent was evaporated under reduced pressure. The crude product was then purified by flash column chromatography (230-400 mesh silica gel, hexane/ethyl acetate gradient) to afford the pure product **22** in 56% yield (39 mg).

7.5. Deuterium labelling experiment:

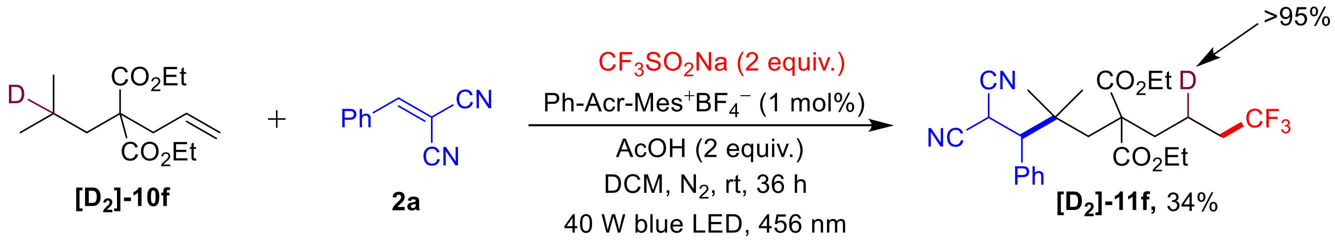

An oven-dried 20 mL reaction tube equipped with a magnetic stirring bar was charged with **[D$_2$]-10f** (38.6 mg, 0.15 mmol, 1 equiv.), **2a** (46.2 mg, 0.23 mmol, 1.5 equiv.), Ph-Mes-Acr$^+$BF$_4^-$ (0.69 mg, 0.0015 mmol, 1 mol%) and CF_3SO_2Na (0.3 mmol, 2 equiv.). The reaction tube was evacuated under vacuum and backfilled with nitrogen; this cycle was repeated several times. The reaction tube was securely sealed with a rubber. Dry DCM (2 mL) and AcOH (18 mg, 0.3 mmol, 2 equiv.) were added *via* a syringe through the septum. The mixture was placed on a magnetic stirrer and irradiated with a Kessil 40 W, 456 nm blue LED, positioned ~3 cm from the reaction tube. A cooling fan was placed ~30 cm away to maintain the system at room temperature. After stirring for 36 h under irradiation, the reaction was diluted with water (10 mL), and extracted with DCM (3 x 20 mL). The combined organic layers were dried over anhydrous Na_2SO_4, filtered, and the solvent was evaporated under reduced pressure. The crude product was then purified by flash column chromatography (230-400 mesh silica gel, hexane/ethyl acetate gradient) to afford the product **[D$_2$]-11f** in 34% yield (24.6 mg).

7.6. Experimental procedure to determine KIE:

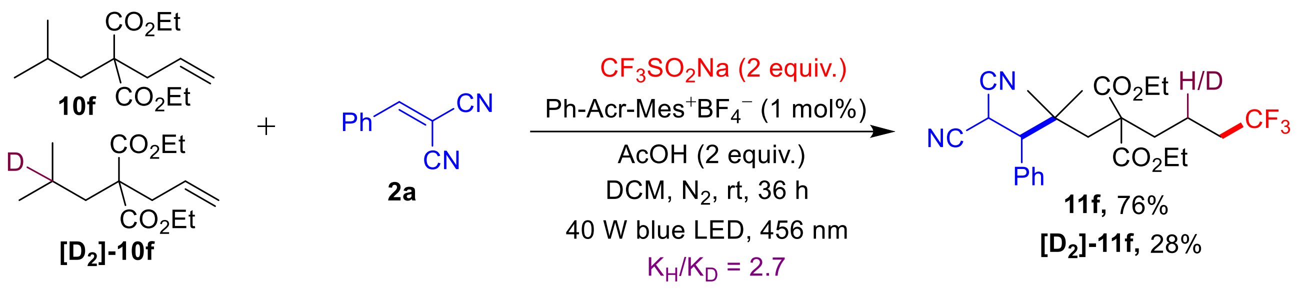

An oven-dried 20 mL reaction tube equipped with a magnetic stirring bar was charged with **10f** (19.2 mg, 0.075 mmol, 0.5 equiv.), and **[D₂]-10f**, (19.4 mg, 0.075 mmol, 0.5 equiv.), **2a** (46.2 g, 0.23 mmol, 1.5 equiv.), Ph-Mes-Acr$^+$BF$_4^-$ (0.69 mg, 0.0015 mmol, 1 mol%) and CF_3SO_2Na (45.6 mg, 0.3 mmol, 2 equiv.). The reaction tube was evacuated under vacuum and backfilled with nitrogen; this cycle was repeated several times. The reaction tube was securely sealed with a rubber. Dry DCM (2 mL) and AcOH (18 mg, 0.3 mmol, 2 equiv.) were added *via* a syringe through the septum. The mixture was placed on a magnetic stirrer and irradiated with a Kessil 40 W, 456 nm blue LED, positioned ~3 cm from the reaction tube. A cooling fan was placed ~30 cm away to maintain the system at room temperature. After stirring for 36 h under irradiation, the reaction was diluted with water (10 mL), and extracted with DCM (3 x 20 mL). The combined organic layers were dried over Na_2SO_4, filtered, and the solvent was evaporated under reduced pressure. The crude was subjected to ^{1}H and ^{19}F NMR analysis, and from the crude NMR data, the ratio of **11f** : **[D2]-11f** was found to be 76 : 28, and from this, the KIE was determined as 2.7 for the reaction.

8. Light ON/OFF experiments:

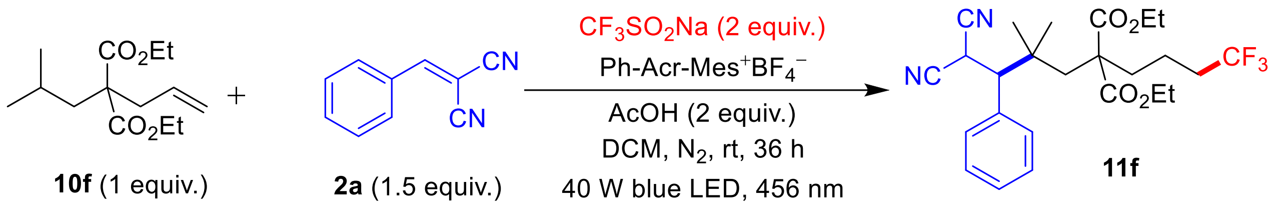

An oven-dried 20 mL reaction tube equipped with a magnetic stirring bar was charged with **10f** (38.4 mg, 1 mmol, 1 equiv.), **2a** (46.2 mg, 1.5 mmol, 1.5 equiv.), Ph-Mes-Acr$^+$BF$_4^-$ (0.69 mg, 0.01 mmol, 1 mol%) and CF_3SO_2Na (45.6 mg, 2 mmol, 2 equiv.). The reaction tube was evacuated under vacuum and backfilled with nitrogen; this cycle was repeated several times. The reaction tube was securely sealed with a rubber septum under argon flow. Freshly dried DCM (20 mL) and benzotrifluoride (1 mmol, 1 equiv.) (was used as an internal standard for ^{19}F NMR) were added *via* a syringe, followed by AcOH (2 equiv.). The mixture was placed on a magnetic stirrer and irradiated with a Kessil 40 W, 456 nm blue LED, positioned ~3 cm from the reaction tube. A cooling fan was placed ~30 cm away to maintain the system at room temperature. The light on/off experiment was performed by alternating between light and dark conditions (light : dark; 1 : 1 h) for 10 h. At the end of each interval, the reaction progress was monitored by ^{19}F NMR using benzotrifluoride as the internal standard. As shown in the Figure, the reaction progressed exclusively during the light periods and halted in the dark, confirming the necessity of continuous irradiation. This observation also indicates that the transformation does not involve a chain propagation mechanism.

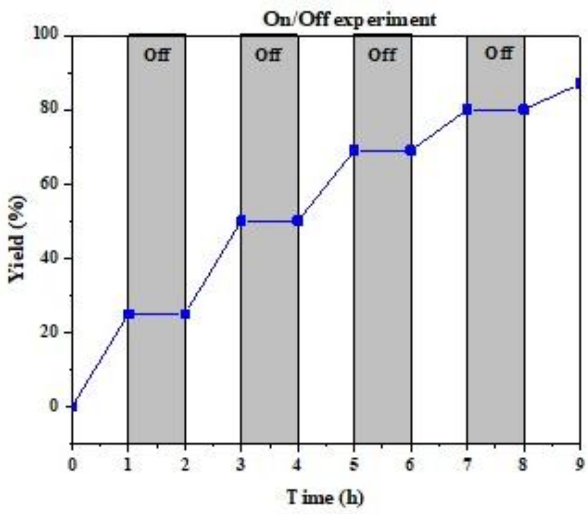

9. Luminescence quenching experiments for mechanistic elucidation:

To perform luminescence quenching studies, 10 mM of commercially available Ph-Acr-$Mes^+BF_4^-$, 1 M of Michael acceptor **2a,** 1 M of alkene **10f**, 1 M of CF_3SO_2Na and 1 M of AcOH solution in spectroscopic grade acetonitrile were prepared as a stock solution. All other solutions with different concentrations were prepared by diluting the stock solution.

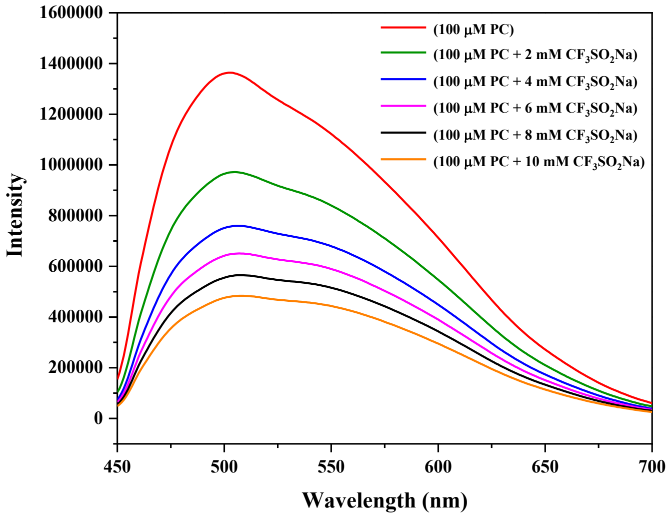

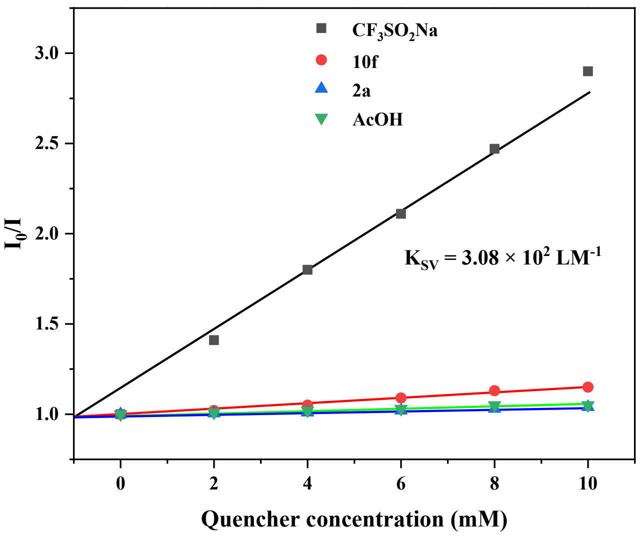

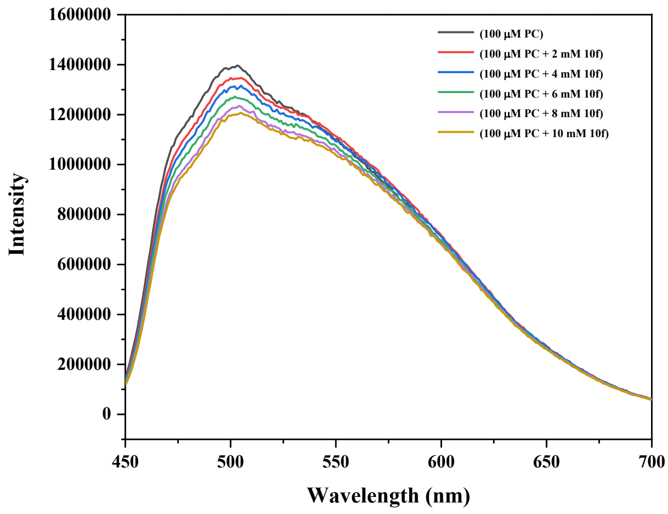

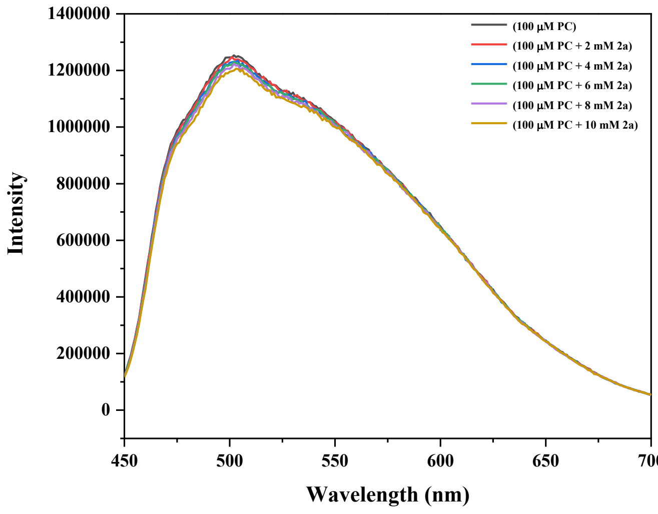

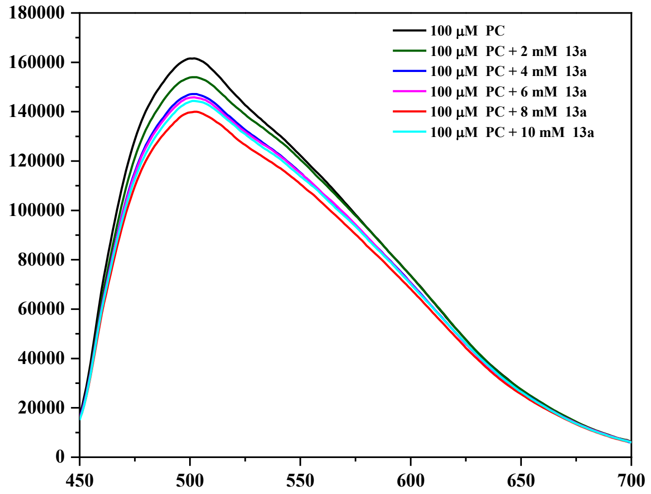

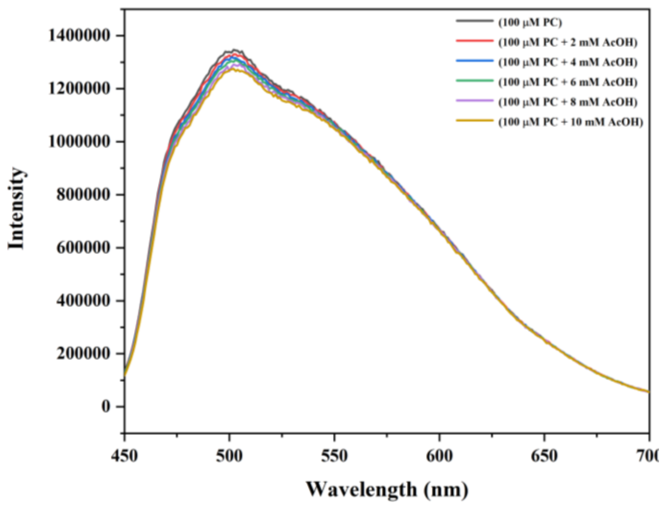

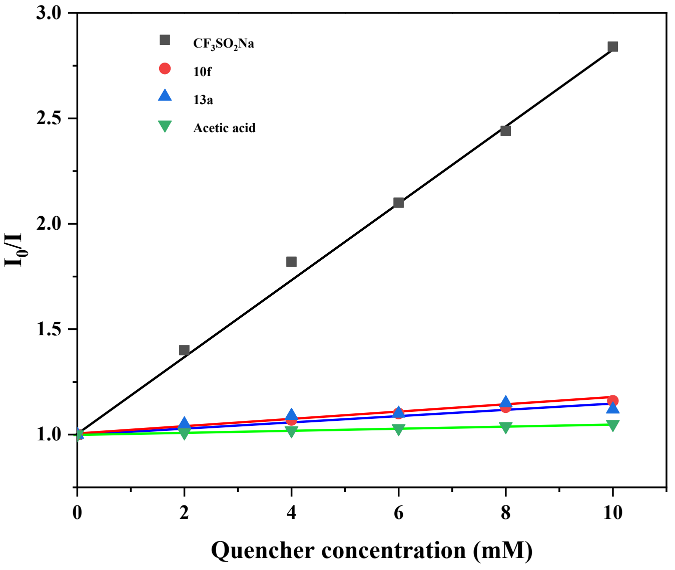

In MeCN Solution: a) 100 µM Mes-Acr$^+$ClO$_4^-$ vs. CF$_3$SO$_2$Na at 440 nm; b) Stern Volmer plot of Luminescence quenching of 100 µM Mes-Acr$^+$ClO$_4$ $^-$ vs. CF$_3$SO$_2$Na, **10f**, **2a** and AcOH; c) 100 µM Mes-Acr$^+$ClO$_4^-$ vs. **10f** at 440 nm; d) 100 µM Mes-Acr$^+$ClO$_4^-$ vs. **2a** at 440 nm; e) 100 µM Mes-Acr$^+$ClO$_4^-$ vs. **13a** at 440 nm; f) 100 µM Mes-Acr$^+$ClO$_4^-$ vs. AcOH at 440 nm; g) Stern Volmer plot of Luminescence quenching of 100 µM Mes-Acr$^+$ClO$_4^-$ vs. CF$_3$SO$_2$Na, **10f**, **13a** and AcOH.

10. EPR experiment:

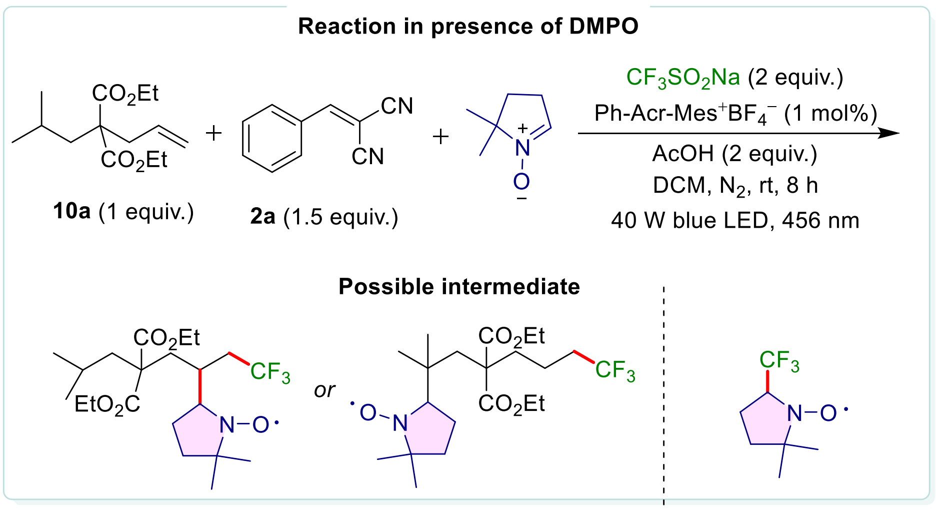

Experimental procedure: To an oven-dried 20 mL reaction tube equipped with a magnetic stir bar, **10a** (51.2 mg, 0.2 mmol, 1 equiv.), **2a** (46.2 mg, 0.3 mmol, 1.5 equiv.), CF_3SO_2Na (62 mg, 0.4 mmol, 2.0 equiv.), Ph-Acr-$Mes^+BF_4^-$ (0.9 mg, 1 mol%) were added. Then, the reaction tube was subjected to a vacuum for 5 minutes and subsequently backfilled with argon; this cycle was repeated four more times. This cycle was repeated four more times. A rubber septum was placed tightly on the top of the reaction tube, with continuous oxygen flow. Freshly dried CH_3CN 2 mL was added using a syringe. Then DMPO (45 mg, 2 equiv.) was added through a syringe. Then, the reaction tube was placed on a magnetic stirrer and irradiated using a 456 nm blue LED (40 W, Kessil), maintaining a distance of ~3 cm between them. A cooling fan was installed approximately one foot from the system to maintain a room temperature. After 30 min., the reaction was removed from the setup, and the reaction mixture was poured into liq. N_2. The above reaction mixture was transferred to an EPR tube using a syringe, and the EPR measurement was performed.

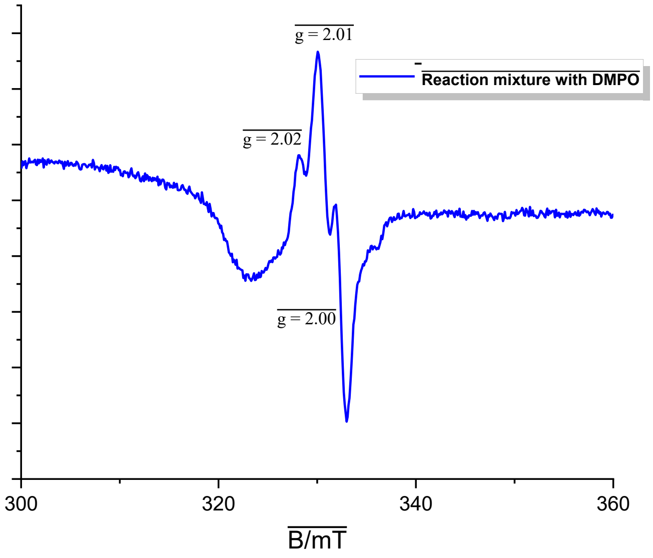

The DMPO spin trap studies were recorded at 80 K temperature using a Bruker EPR A300 spectrometer. In all cases, the microwave frequency was 9.3 GHz, the power was 2.55 mW, the modulation frequency was 100 kHz, and the modulation amplitude was 10.00 G.

HRMS of the DMPO adduct/Intermediate: At the same time, some amount of the reaction mixture was taken out, and HRMS was done.

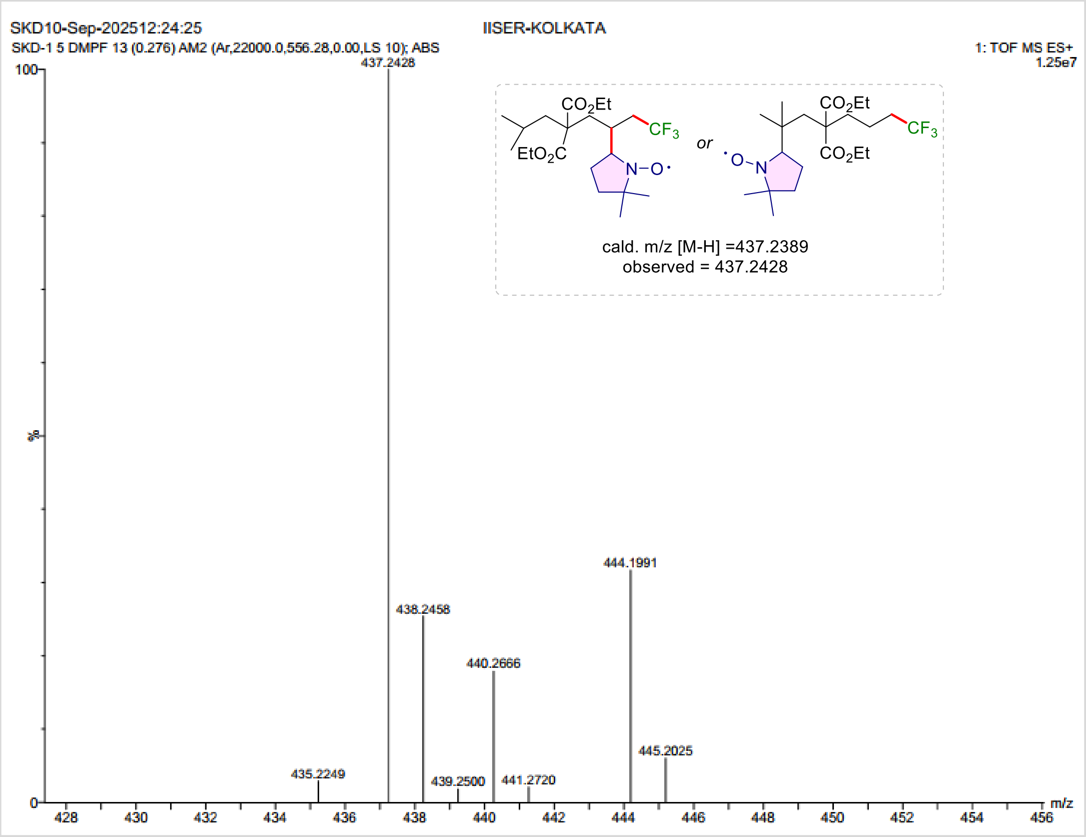

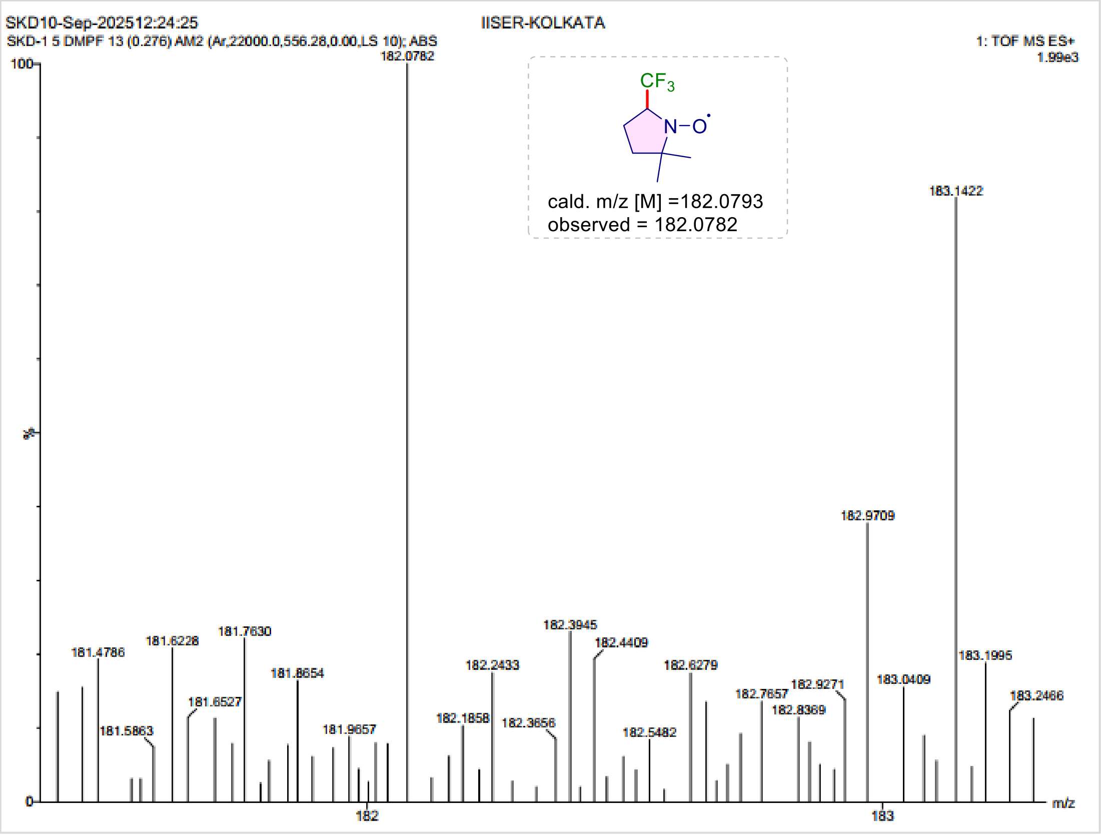

11. X-ray crystallographic data:

11.1. X-ray crystallographic data of 11o with 50% ellipsoid contour probability:

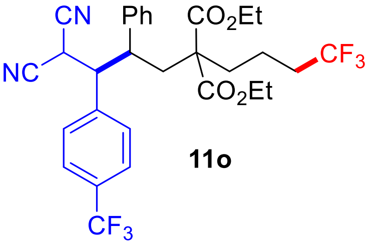

11o

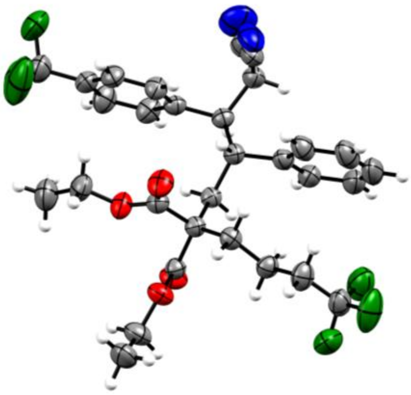

Table S1 Crystal data and structure refinement for Compound 11o:

Identification code	Compound **11o**
Empirical formula	$C_{30}H_{30}F_6N_2O_4$
CCDC Number	2416402
Formula weight	596.56
Temperature/K	298.0
Crystal system	monoclinic
Space group	$P2_1/c$
a/Å	12.8044(3)
b/Å	19.2055(4)
c/Å	13.1893(3)
α/°	90
β/°	114.067(2)
γ/°	90
Volume/Å^3	2961.49(12)
Z	4
ρ_{calc}g/cm^3	1.338
μ/mm^{-1}	0.981
F(000)	1240.0
Crystal size/mm^3	0.05 × 0.01 × 0.01
Radiation	CuKα (λ = 1.54184)
2θ range for data collection/°	7.562 to 136.374
Index ranges	-15 ≤ h ≤ 15, -22 ≤ k ≤ 21, -10 ≤ l ≤ 15
Reflections collected	22004
Independent reflections	5362 [R_{int} = 0.0478, R_{sigma} = 0.0373]
Data/restraints/parameters	5362/0/381
Goodness-of-fit on F^2	1.057
Final R indexes [I>=2σ (I)]	R_1 = 0.0657, wR_2 = 0.1494
Final R indexes [all data]	R_1 = 0.0803, wR_2 = 0.1578
Largest diff. peak/hole / e Å^{-3}	0.45/-0.31

11.2. X-ray crystallographic data of 14b with 50% ellipsoid contour probability:

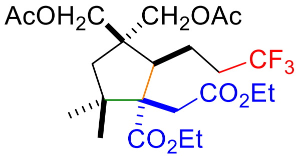

14b

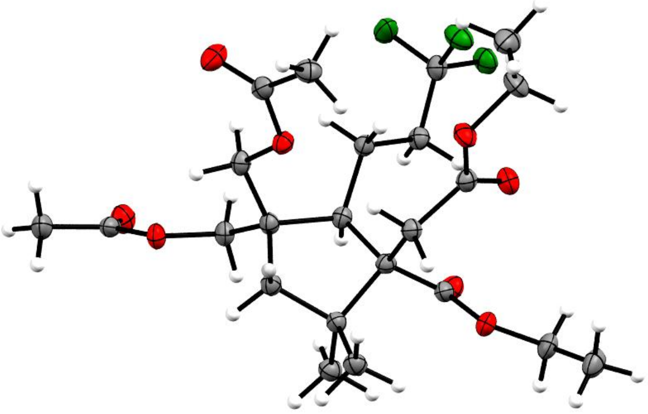

Table S2 Crystal data and structure refinement for Compound 14b:

Identification code	Compound **14b**
Empirical formula	$C_{22}H_{35}F_3O_8$
CCDC Number	2264468
Formula weight	496.51
Temperature/K	100.01(10)
Crystal system	monoclinic
Space group	$P2_1/c$
a/Å	8.70612(15)
b/Å	10.77758(19)
c/Å	26.5637(4)
α/°	90
β/°	99.3234(16)
γ/°	90
Volume/Å³	2459.57(7)
Z	4
ρ_{calc}g/cm³	1.341
μ/mm^{-1}	0.978
F(000)	1056.0
Crystal size/mm³	0.05 × 0.01 × 0.01
Radiation	CuKα (λ = 1.54184)
2θ range for data collection/°	6.744 to 136.438
Index ranges	-10 ≤ h ≤ 10, -12 ≤ k ≤ 12, -31 ≤ l ≤ 31
Reflections collected	41855
Independent reflections	4479 [R_{int} = 0.1984, R_{sigma} = 0.0593]
Data/restraints/parameters	4479/0/314
Goodness-of-fit on F²	1.095
Final R indexes [I>=2σ (I)]	R_1 = 0.0564, wR_2 = 0.1378
Final R indexes [all data]	R_1 = 0.0564, wR_2 = 0.1446
Largest diff. peak/hole / e Å^{-3}	0.29/-0.34

12. Characterization data:

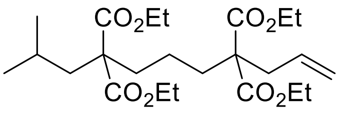

1a

Tetraethyl-10-methylundec-1-ene-4,4,8,8-tetracarboxylate **(1a):** Yield 71% (5.1 g); R_f = 0.4 (ethyl acetate/n-hexane, 1 : 9); colorless oil, ^{1}H NMR (400 MHz, CHLOROFORM-*D*) δ 5.68 – 5.50 (m, 1H), 5.08 (ddt, *J* = 4.2, 2.1, 0.9 Hz, 1H), 5.05 (q, *J* = 1.1 Hz, 1H), 4.24 – 4.01 (m, 8H), 2.61 (dt, *J* = 7.5, 1.2 Hz, 2H), 2.06 – 1.80 (m, 6H), 1.56 (dt, *J* = 13.1, 6.5 Hz, 1H), 1.35 – 1.17 (m, 12H), 1.14 – 0.99 (m, 2H), 0.85 (d, *J* = 6.7 Hz, 6H); ^{13}C NMR (101 MHz, CHLOROFORM-*D*) δ 172.1, 171.2, 132.6, 119.0, 61.3, 61.2, 57.3, 56.9, 40.5, 37.0, 32.7, 32.5, 24.1, 23.8, 18.8, 14.2, 14.2; HRMS (ESI) m/z [M + Na]$^+$ calcd for $C_{24}H_{40}O_8Na$: 479.2621, found : 479.2623.

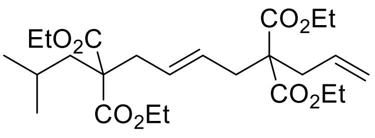

6a

Tetraethyl-(E)-11-methyldodeca-1,6-diene-4,4,9,9-tetracarboxylate (**6a**): Yield 61% (2.01 g); R_f = 0.3 (ethyl acetate/n-hexane, 1 : 9); colorless oil; ^{1}H NMR (500 MHz, $CDCl_3$) δ 5.71 – 5.52 (m, 1H), 5.42 – 5.26 (m, 2H), 5.09 (dd, *J* = 13.4, 8.0 Hz, 2H), 4.16 (p, *J* = 7.3 Hz, 8H), 2.71 – 2.48 (m, 6H), 1.82 (d, *J* = 6.3 Hz, 2 H), 1.64 (dt, *J* = 13.1, 6.5 Hz, 1H), 1.23 (t, *J* = 7.1 Hz, 12H), 0.86 (d, *J* = 6.6 Hz, 6H); ^{13}C NMR (101 MHz, $CDCl_3$) δ 171.7, 170.8, 132.5, 129.1, 128.2, 119.3, 61.3, 61.2, 61.2, 57.4, 57.1, 40.6, 36.7, 36.1, 35.6, 24.1, 23.7, 23.7, 14.2, 14.1; HRMS (ESI) m/z [M + H]$^+$ calcd for $C_{25}H_{41}O_8$: 469.2801, found: 469.2804.

H

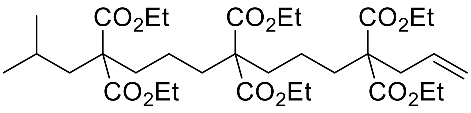

8a

Hexaethyl-14-methylpentadec-1-ene-4,4,8,8,12,12-hexa carboxylate (**8a**): Yield 55% (3.2 g); R_f = 0.3 (ethyl acetate/n-hexane, 1 : 9); colorless solid, ^{1}H NMR (500 MHz, $CDCl_3$) δ 5.60 (dt, *J* = 16.9, 7.5 Hz, 1H), 5.19 – 4.96 (m, 2H), 4.22 – 4.01 (m, 12H), 2.60 (d, *J* = 7.3 Hz, 2H), 1.95 – 1.76 (m, 10H), 1.55 (td, *J* = 13.0, 6.5 Hz, 1H), 1.22 (dd, *J* = 11.5, 6.9 Hz, 18H), 1.13 – 0.98 (m, 4H), 0.85 (d, *J* = 6.6 Hz, 6H); ^{13}C NMR (126 MHz, $CDCl_3$) δ 172.0, 171.5, 171.1, 132.6, 119.0, 61.3, 61.2, 61.1, 57.5, 57.3, 57.0, 40.7, 37.1, 33.1, 33.1, 32.9, 32.6, 24.2, 23.8, 19.2, 18.8, 14.2, 14.2, 14.2; HRMS (ESI) m/z [M + H]$^+$ calcd for $C_{34}H_{57}O_{12}$: 657.3850, found : 657.3853.

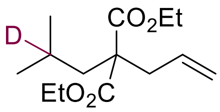

[D$_2$]-10f

Diethyl-2-allyl-2-(2-methylpropyl-2-d)malonate (**[D$_2$]-10f**): Yield 67% (0.86 g); R_f = 0.4 (ethyl acetate/n-hexane, 1 : 9); colorless oil; ^{1}H NMR (500 MHz, $CDCl_3$) δ 5.74 – 5.51 (m, 1H), 5.15 – 4.90 (m, 2H), 4.25 – 3.97 (m, 4H), 2.68 (d, *J* = 7.4 Hz, 2H), 1.85 (s, 2H), 1.24 (t, *J* = 7.1 Hz, 6H), 0.86 (s, 6H); ^{13}C NMR (126 MHz, $CDCl_3$) δ 171.8, 132.9, 118.9, 61.2, 57.1, 40.6, 37.3, 23.7, 14.2; HRMS (ESI) m/z [M + Na]$^+$ calcd for $C_{14}H_{23}DO_4Na$: 280.1635, found : 280.1642.

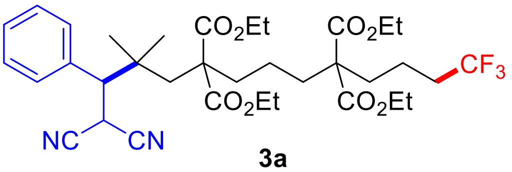

3a

Tetraethyl-13,13-dicyano-1,1,1-trifluoro-11,11-dimethyl-12-phenyltridecane-5,5,9,9-tetracarboxylate (**3a**): Yield 57% (58.2 mg); R_f = 0.3 (ethyl acetate/n-hexane, 2 : 8); colorless oil; ^{1}H NMR (500 MHz, $CDCl_3$) δ 7.40 (td, *J* = 5.3, 2.8 Hz, 5H), 4.45 (dd, *J* = 5.0, 0.8 Hz, 1H), 4.13 (m, 8H), 3.03 (d, *J* = 4.9 Hz, 1H), 2.25 (d, *J* = 14.9 Hz, 1H), 2.12 – 2.00 (m, 3H), 1.96 (m, 2H), 1.91 – 1.79 (m, 4H), 1.46 – 1.36 (m, 2H), 1.22 (tt, *J* = 7.1, 1.7 Hz, 14H), 1.12 (s, 3H), 1.09 – 1.00 (m, 2H), 0.97 (s, 3H), 0.86 (m, 1H); ^{13}C NMR (126 MHz, $CDCl_3$) δ 171.7, 171.5, 171.1, 135.6, 128.9, 126.9 (q, *J* = 276.5 Hz), 113.4, 113.2, 61.8, 61.7, 61.5, 57.4, 57.2, 56.7, 41.9, 38.2, 35.7, 34.8, 33.9 (q, *J* = 28.6 Hz), 32.7, 31.7, 26.1, 25.8, 25.0, 19.4, 17.2 (q, *J* = 3.1 Hz), 14.2, 14.1, 14.0, 14.0; ^{19}F NMR (471 MHz, $CDCl_3$) δ -66.33 (t, *J* = 10.8 Hz); HRMS (ESI) m/z [M + Na]$^+$ calcd for $C_{35}H_{48}F_3N_2O_8$ 681.3357 found 681.3369.

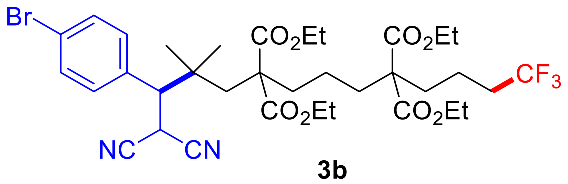

3b

Tetraethyl-12-(4-bromophenyl)-13,13-dicyano-1,1,1-trifluoro-11,11-dimethyltridecane-5,5,9,9-tetra carboxylate (**3b**): Yield 49% (55.8 mg); R_f = 0.4 (ethyl acetate/n-hexane, 2 : 8); colorless oil; ^{1}H NMR (500 MHz, $CDCl_3$) δ 7.57 – 7.51 (m, 2H), 7.31 (d, *J* = 8.1 Hz, 2H), 4.47 (d, *J* = 4.7 Hz, 1H), 4.23 – 4.07 (m, 8H), 3.04 (d, *J* = 4.7 Hz, 1H), 2.24 (d, *J* = 15.0 Hz, 1H), 2.06 (qt, *J* = 9.0, 6.9 Hz, 2H), 2.01 – 1.92 (m, 3H), 1.86 (m, 4H), 1.47 – 1.37 (m, 2H), 1.28 – 1.18 (m, 12H), 1.12 (s, 3H), 1.08 – 0.98 (m, 2H), 0.94 (s, 3H); ^{13}C NMR (126 MHz, $CDCl_3$) δ 171.7, 171.4, 171.1, 134.6, 132.2, 127.0 (q, *J* = 276.4 Hz), 123.3, 113.2, 113.0, 61.9, 61.8, 61.5, 57.2, 56.7, 56.5, 42.3, 38.2, 36.0, 34.0 (q, *J* = 28.7 Hz), 32.8, 31.8, 26.1, 25.9, 24.9, 22.8, 19.5, 17.2 (q, *J* = 2.9 Hz), 14.2, 14.1, 14.0; ^{19}F NMR (471 MHz, $CDCl_3$) δ -66.30 (t, *J* = 10.8 Hz); HRMS (ESI) m/z [M + Na]$^+$ calcd for $C_{35}H_{46}BrF_3N_2O_8Na$ 783.2261 found 783.2102.

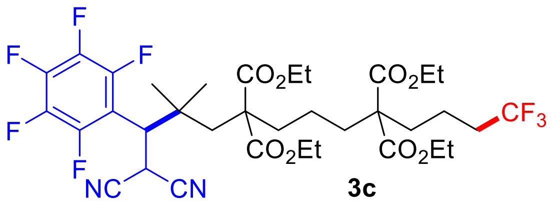

3c

Tetraethyl-13,13-dicyano-1,1,1-trifluoro-11,11-dimethyl-12-(perfluorophenyl) tridecane-5,5,9,9-tetracarboxylate (**3c**): Yield 47% (54.3 mg); R_f = 0.4 (ethyl acetate/n-hexane, 2 : 8); colorless oil; ^{1}H NMR (500 MHz, $CDCl_3$) δ 4.51 (d, J = 9.8 Hz, 1H), 4.15 (m, 8H), 3.73 (d, J = 9.8 Hz, 1H), 2.19 (d, J = 14.8 Hz, 1H), 2.10 (d, J = 14.8 Hz, 1H), 2.03 – 1.95 (m, 4H), 1.92 – 1.78 (m, 5H), 1.46 – 1.35 (m, 2H), 1.32 – 1.15 (m, 12H), 1.10 (s, 3H), 1.08 (s, 3H), 1.06 – 0.95 (m, 2H); ^{13}C NMR (126 MHz, $CDCl_3$) δ 171.3, 171.3, 171.1, 171.1, 127.0 (q, J = 276.4 Hz), 112.8, 112.0, 110.3, 61.8, 61.8, 61.5, 57.2, 56.9, 56.7, 49.4, 40.3, 40.3, 39.8, 35.5, 33.9 (q, J = 28.9 Hz), 32.7, 31.8, 25.7, 25.2, 22.9, 22.9, 19.5, 17.2 (q, J = 2.9 Hz), 14.1, 14.0; ^{19}F NMR (471 MHz, $CDCl_3$) δ -66.39 (t, J = 10.8 Hz), -132.78 (d, J = 22.7 Hz), -136.64 (dt, J = 23.1, 7.0 Hz), -150.42 (t, J = 21.3 Hz), -159.09 (td, J = 21.9, 7.2 Hz), -159.29 (td, J = 22.2, 7.7 Hz); HRMS (ESI) m/z $[M + Na]^+$ calcd for $C_{35}H_{42}F_8N_2O_8Na$ 793.2706 found 793.2706.

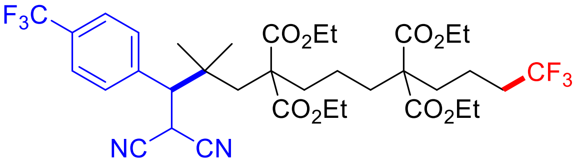

3d

Tetraethyl-13,13-dicyano-1,1,1-trifluoro-11,11-dimethyl-12-(4-(trifluoromethyl)phenyl)tridecane-5,5,9,9-tetracarboxylate (**3d**): Yield 51% (57.3 mg); R_f = 0.4 (ethyl acetate/n-hexane, 2 : 8); colorless oil; ^{1}H NMR (500 MHz, $CDCl_3$) δ 7.68 (d, J = 8.2 Hz, 2H), 7.58 (d, J = 8.0 Hz, 2H), 4.54 (d, J = 4.5 Hz, 1H), 4.21 – 4.08 (m, 8H), 3.17 (d, J = 4.6 Hz, 1H), 2.28 (d, J = 15.0 Hz, 1H), 2.12 – 1.92 (m, 5H), 1.91 – 1.79 (m, 4H), 1.47 – 1.37 (m, 2H), 1.23 (m, 12H), 1.16 (s, 3H), 1.13 – 0.99 (m, 2H), 0.94 (s, 3H), 0.91 – 0.81 (m, 2H); ^{13}C NMR (101 MHz, $CDCl_3$) δ 171.8, 171.4, 171.1, 139.6 (q, J = 1.4 Hz), 131.4, 131.1, 131.1, 130.4, 128.3, 125.9 (q, J = 3.8 Hz), 125.6, 125.3, 122.6, 113.1, 112.9, 62.0, 61.8, 61.5, 57.2, 56.7, 56.4, 42.4, 38.2, 36.2, 33.9 (q, J = 28.8 Hz), 32.7, 31.8, 29.8, 26.2, 26.0, 24.8, 19.4, 17.2 (d, J = 3.0 Hz), 14.2, 14.2, 14.0, 14.0; HRMS (ESI) m/z $[M + Na]^+$ calcd for $C_{36}H_{46}F_6N_2O_8Na$ 771.3051 found 771.3034.

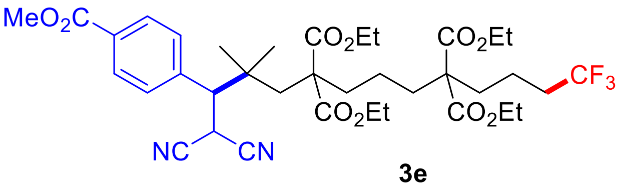

3e

Tetraethyl-13,13-dicyano-1,1,1-trifluoro-12-(4-(methoxycarbonyl) phenyl)-11,11-dimethyltridecane-5,5,9,9-tetracarboxylate (**3e**): Yield 46% (51.0 mg); R_f = 0.2 (ethyl acetate/n-hexane, 2 : 8); colorless solid; ^{1}H NMR (500 MHz, $CDCl_3$) δ 8.07 (d, J = 8.5 Hz, 2H), 7.51 (d, J = 7.8 Hz, 2H), 4.51 (d, J = 4.8 Hz, 1H), 4.21 – 4.10 (m, 8H), 3.92 (s, 3H), 3.15 (d, J = 4.8 Hz, 1H), 2.26 (d, J = 15.0 Hz, 1H), 2.09 – 1.81 (m, 9H), 1.48 – 1.36 (m, 2H), 1.22 (dd, J = 12.3, 7.0 Hz, 12H), 1.14 (s, 3H), 1.04 (dd, J = 18.3, 12.7 Hz, 2H), 0.95 (s, 3H); ^{13}C NMR (126 MHz, $CDCl_3$) δ 171.58, 171.28, 170.97, 166.39, 140.47, 130.65, 129.99, 127.91, 126.81 (q, J = 276.3 Hz), 112.80, 61.77, 61.66, 61.4, 57.0, 56.6, 56.6, 52.3, 42.2, 38.1, 35.9, 33.8 (q, J = 29.0 Hz), 32.6, 31.7, 26.0, 25.8, 24.7, 19.3, 17.0 (q, J = 2.7 Hz), 14.0, 13.9, 13.9; ^{19}F NMR (471 MHz, $CDCl_3$) δ -66.32 (t, J = 11 Hz); HRMS (ESI) m/z $[M + Na]^+$ calcd for $C_{37}H_{49}F_3N_2O_{10}Na$ 761.3232 found 761.3230.

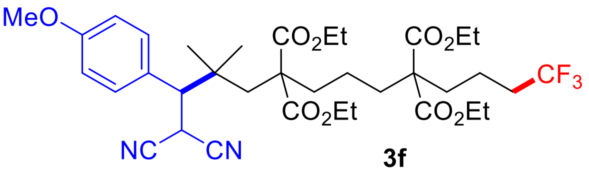

3f

Tetraethyl-13,13-dicyano-1,1,1-trifluoro-12-(4-methoxyphenyl)-11,11-dimethyltridecane-5,5,9,9-tetracarboxylate (**3f**): Yield 53% (56.5 mg); R_f = 0.3 (ethyl acetate/n-hexane, 2 : 8); Yellow oil; ^{1}H NMR (500 MHz, $CDCl_3$) δ 7.33 (d, J = 8.3 Hz, 2H), 6.94 – 6.88 (m, 2H), 4.41 (d, J = 4.9 Hz, 1H), 4.28 – 4.02 (m, 8H), 3.81 (s, 3H), 2.98 (d, J = 4.9 Hz, 1H), 2.22 (d, J = 14.9 Hz, 1H), 2.12 – 2.02 (m, 4H), 2.00 – 1.92 (m, 2H), 1.90 – 1.79 (m, 5H). 1.46 – 1.34 (m, 2H), 1.22 (m, 12H), 1.10 (s, 3H), 1.03 (q, J = 8.5 Hz, 3H), 0.96 (s, 3H); ^{13}C NMR (126 MHz, $CDCl_3$) δ 171.7, 171.6, 171.2, 135.6, 128.9, 126.9 (q, J = 276.5 Hz), 113.5, 113.3, 61.6, 61.7, 61.6, 57.4, 57.2, 56.7, 42.0, 38.2, 35.8, 34.9, 33.9 (q, J = 28.4 Hz), 32.7, 31.8, 26.1, 25.8, 25.0, 19.4, 17.2 (q, J = 3.1 Hz), 14.2, 14.2, 14.1, 14.0; ^{19}F NMR (471 MHz, $CDCl_3$) δ -66.33 (t, J = 10.8 Hz); HRMS (ESI) m/z $[M + Na]^+$ calcd for $C_{36}H_{49}F_3N_2O_9Na$ 733.3282 found 733.3284.

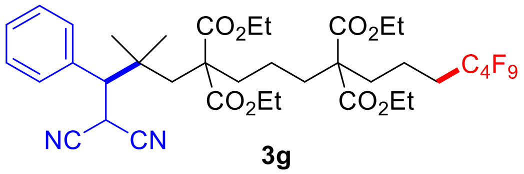

3g

Tetraethyl-1,1-dicyano-16,16,16,16,16,16,16,16,16-nonafluoro-3,3-dimethyl-2-phenyl-16l12-hexadeca-13,15-diyne-5,5,9,9-tetracarboxylate (**3g**): Yield 42% (52.1 mg); R_f = 0.4 (ethyl acetate/n-hexane, 2 : 8); Yellow oil; ^{1}H NMR (500 MHz, $CDCl_3$) δ 7.44 – 7.35 (m, 5H), 4.45 (d, *J* = 5.0 Hz, 1H), 4.20 – 4.11 (m, 8H), 3.04 (d, *J* = 5.0 Hz, 1H), 2.25 (d, *J* = 14.9 Hz, 1H), 2.11 – 1.85 (m, 9H), 1.53 – 1.45 (m, 2H), 1.26 – 1.19 (m, 12H), 1.13 (s, 3H), 1.09 – 1.01 (m, 2H), 0.98 (s, 3H); ^{13}C NMR (126 MHz, $CDCl_3$) δ 171.7, 171.5, 171.1, 135.6, 129.9, 129.0, 113.4, 113.2, 61.8, 61.7, 61.5, 57.5, 57.3, 56.8, 42.0, 38.3, 35.8, 32.9, 32.2, 26.2, 25.8, 25.0, 19.5, 14.2, 14.0, 14.0; ^{19}F NMR (376 MHz, CHLOROFORM-*D*) δ -80.91 (tt, *J* = 9.5, 3.2 Hz), -114.40 (ddd, *J* = 19.4, 13.7, 6.9 Hz), -124.38 (d, *J* = 10.4 Hz), -125.90 (ddd, *J* = 13.4, 10.1, 2.6 Hz); HRMS (ESI) m/z [M + Na]+ calcd for $C_{38}H_{48}F_9N_2O_8$: 831.3267 found : 831.3276.

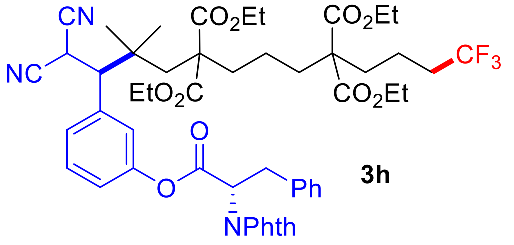

3h

2-(((S)-1-(3-(1,1-Dicyano-5,5,9,9-tetrakis(ethoxycarbonyl)-13,13,13-trifluoro-3,3-dimethyltridecan-2-yl)phenoxy)-1-oxo-3-phenylpropan-2-yl)carbamoyl)benzoic acid (**3h**): (dr = 1 : 1); Yield 49% (71.5 mg); R_f = 0.2 (ethyl acetate/n-hexane, 2 : 8); Yellow oil; ^{1}H NMR (500 MHz, $CDCl_3$) δ 7.83 – 7.77 (m, 4H), 7.72 – 7.66 (m, 4H), 7.42 (dd, *J* = 10.1, 5.6 Hz, 2H), 7.34 (s, 2H), 7.25 – 7.07 (m, 14H), 5.39 (dd, *J* = 5.1, 1.5 Hz, 1H), 5.37 (dd, *J* = 5.1, 1.5 Hz, 1H), 4.49 (t, *J* = 5.2 Hz, 2H), 4.20 – 4.09 (m, 16H), 3.72 – 3.66 (m, 2H), 3.57 (dd, *J* = 14.3, 11.1 Hz, 2H), 3.08 (d, *J* = 4.7 Hz, 2H), 2.30 – 2.23 (m, 2H), 2.10 – 1.83 (m, 18H), 1.42 (m, 4H), 1.23 (m, 24H), 1.16 (s, 3H), 1.14 (s, 3H), 1.10 – 1.00 (m, 4H), 0.95 (s, 3H), 0.93 (s, 3H); ^{13}C NMR (126 MHz, $CDCl_3$) δ 171.7, 171.7, 171.4, 171.1, 167.7, 167.6, 167.4, 167.4, 150.7, 150.7, 137.3, 137.3, 136.4, 136.4, 134.3, 131.6, 130.0, 129.1, 128.7, 127.1, 126.9 (q, *J* = 276.5 Hz), 123.7, 123.7, 122.1, 122.0, 113.2, 113.1, 113.0, 113.0, 61.8, 61.8, 61.8, 61.5, 57.2, 56.8, 56.6, 56.4, 53.3, 42.5, 42.3, 38.3, 36.1, 36.0, 35.1, 35.1, 33.9 (q, *J* = 28.7 Hz), 32.8, 31.8, 26.3, 25.6, 24.9, 24.8, 19.4, 17.2 (q, *J* = 2.8 Hz), 14.1, 14.0, 14.0; ^{19}F NMR (471 MHz, $CDCl_3$) δ -66.29 (t, *J* = 10.7 Hz); HRMS (ESI) m/z [M + Na]+ calcd for $C_{52}H_{58}F_3N_3O_{12}Na$: 996.3870 found : 996.3875.

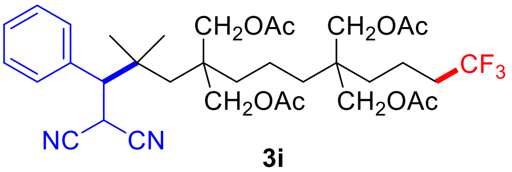

3i

2,6-bis(acetoxymethyl)-2-(4,4-dicyano-2,2-dimethyl-3-phenylbutyl)-6-(4,4,4-trifluorobutyl)heptane-1,7-diyl diacetate (**3i**): Yield 42% (42.6 mg); R_f = 0.2 (ethyl acetate/n-hexane, 2 : 8); colorless oil; ^{1}H NMR (400 MHz, CHLOROFORM-*D*) δ 7.41 (t, *J* = 2.5 Hz, 5H), 4.36 (d, *J* = 5.4 Hz, 1H), 4.06 – 3.78 (m, 8H), 2.97 (d, *J* = 5.5 Hz, 1H), 2.10 (d, *J* = 10.7 Hz, 2H), 2.05 (d, *J* = 1.2 Hz, 6H), 2.02 (s, 3H), 2.00 (s, 3H), 1.70 (d, *J* = 14.7 Hz, 1H), 1.55 (s, 1H), 1.46 (dt, *J* = 11.3, 6.9 Hz, 2H), 1.40 – 1.28 (m, 5H), 1.22 (s, 3H), 1.18 (s, 2H), 1.16 (s, 3H), 1.06 (s, 1H); ^{13}C NMR (101 MHz, CHLOROFORM-*D*) δ 170.9, 170.8, 135.7, 129.9, 129.1, 129.0, 127.08 (q, *J* = 276.6 Hz), 113.4, 113.1, 66.6, 66.5, 65.7, 65.6, 59.3, 41.5, 39.7, 39.0, 38.8, 34.6, 34.3 (q, *J* = 28.3 Hz), 31.8, 30.6, 27.2, 26.7, 24.8, 20.9, 20.9, 20.9, 16.4, 15.8; ^{19}F NMR (376 MHz, CHLOROFORM-*D*) δ -66.03 (t, *J* = 10.8 Hz); HRMS (ESI) m/z [M + Na]+ calcd for $C_{35}H_{47}F_3N_2O_8Na$: 703.3182 found : 703.3187.

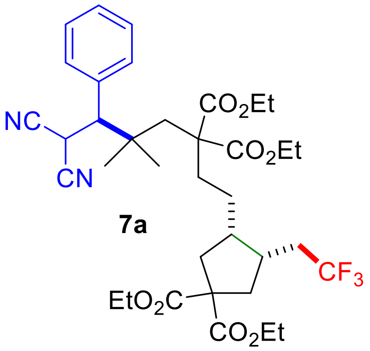

7a

Diethyl-3-(7,7-dicyano-3,3-bis(ethoxycarbonyl)-5,5-dimethyl-6-phenylheptyl)-4-(2,2,2-trifluoroethyl)cyclopentane-1,1-dicarboxylate (**7a**): Yield 83% (86.2 mg); (dr = 1 : 1); R_f = 0.3 (ethyl acetate/n-hexane, 2 : 8); colorless oil; ^{1}H NMR (500 MHz, $CDCl_3$) δ 7.47 – 7.34 (m, 10H), 4.54 (d, *J* = 4.8 Hz, 1H), 4.51 (d, *J* = 4.9 Hz, 1H), 4.17 (m, 16H), 3.08 (d, *J* = 4.8 Hz, 1H), 3.03 (d, *J* = 4.9 Hz, 1H), 2.46 (dd, *J* = 13.6, 6.4 Hz, 2H), 2.35 – 2.20 (m, 6H), 2.11 – 1.82 (m, 16H), 1.28 – 1.19 (m, 24H), 1.15 (s, 3H), 1.09 (d, *J* = 9.4 Hz, 4H), 1.06 – 1.01 (m, 4H), 0.99 – 0.91 (m, 5H); ^{13}C NMR (126 MHz, $CDCl_3$) δ 172.6, 172.5, 172.4, 171.8, 171.7, 171.5, 171.5, 135.7, 135.6, 129.9, 128.8, 127.1 (d, *J* = 275.8 Hz), 113.5, 113.5, 113.3, 113.3, 61.7, 61.7, 61.7, 61.6, 58.3, 58.3, 57.7, 56.9, 56.5, 42.7, 42.1, 42.1, 41.5, 38.4, 38.3, 38.2, 38.0, 38.0, 36.4, 36.3, 34.9, 34.2, 33.1 (q, *J* = 27.8 Hz), 33.0 (q, *J* = 27.8 Hz), 26.0, 25.8, 25.7, 25.0, 24.9, 23.4, 23.3, 14.2, 14.0, 13.9; ^{19}F NMR (471 MHz, $CDCl_3$) δ -64.32 (t, *J* = 8.0 Hz), -64.36 (t, *J* = 7.9 Hz); HRMS (ESI) m/z [M + H]+ calcd for $C_{36}H_{48}F_3N_2O_8$ 693.3357 found 693.3360.

Diethyl-3-(7,7-dicyano-3,3-bis(ethoxycarbonyl)-5,5-dimethyl-6-(p-tolyl)heptyl)-4-(2,2,2-trifluoroethyl)cyclopentane-1,1-dicarboxylate (**7b**): Yield 73% (77.1 mg); (dr = 1 : 1); R_f = 0.5 (ethyl acetate/n-hexane, 2 : 8); colorless oil; ^{1}H NMR (400 MHz, $CDCl_3$) δ 7.30 (d, *J* = 7.9 Hz, 4H), 7.18 (d, *J* = 7.8 Hz, 4H), 4.50 (d, *J* = 4.8 Hz, 1H), 4.48 (d, *J* = 5.0 Hz, 1H), 4.20 – 4.13 (m, 16H), 3.03 (d, *J* = 4.8 Hz, 1H), 2.98 (d, *J* = 4.9 Hz, 1H), 2.48 – 2.42 (m, 2H), 2.34 (s, 6H), 2.32 – 2.18 (m, 6H), 2.12 – 1.91 (m, 16H), 1.25 – 1.20 (m, 24H), 1.14 – 0.93 (m, 16H); ^{13}C NMR (101 MHz, $CDCl_3$) δ 172.6, 172.6, 172.4, 171.9, 171.8, 171.6, 171.6, 138.7, 138.7, 132.6, 132.5, 129.8, 129.8, 129.7, 129.7, 129.6, 127.1 (q, *J* = 279.2 Hz), 113.6, 113.6, 113.4, 113.4, 61.8, 61.8, 61.8, 61.7, 61.7, 61.7, 61.7, 58.3, 58.3, 57.5, 56.7, 56.5, 56.5, 42.7, 42.1, 42.1, 41.4, 38.4, 38.3, 38.2, 38.1, 38.0, 36.4, 34.9, 34.2, 33.1 (q, *J* = 28.1 Hz), 33.0 (q, *J* = 28.6 Hz), 26.0, 25.8, 25.1, 25.0, 23.4, 23.3, 21.1, 14.1, 14.0, 13.9, 13.9; ^{19}F NMR (376 MHz, $CDCl_3$) δ -62.70 – -65.85 (m); HRMS (ESI) m/z [M + Na]$^+$ calcd for $C_{37}H_{49}F_3N_2O_8Na$: 729.3339 found : 729.3328.

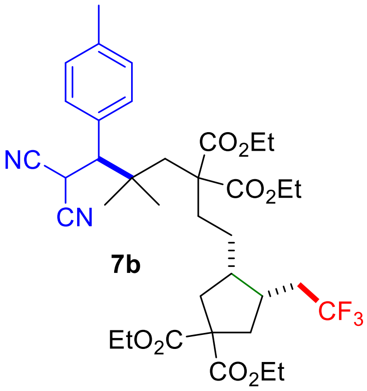

7b

Diethyl-3-(7,7-dicyano-3,3-bis(ethoxycarbonyl)-6-(4-methoxyphenyl)-5,5-dimethylheptyl)-4-(2,2,2-trifluoroethyl)cyclopentane-1,1-dicarboxylate (**7c**): Yield 63% (67.9 mg); (dr = 1 : 1); R_f = 0.2 (ethyl acetate/n-hexane, 2 : 8); colorless oil; ^{1}H NMR (400 MHz, $CDCl_3$) δ 7.36 (m, 4H), 6.92 (ddd, *J* = 8.9, 4.9, 1.3 Hz, 4H), 4.52 – 4.50 (m, 1H), 4.49 – 4.47 (m, 1H), 4.23 – 4.14 (m, 16H), 3.81 (d, *J* = 4.9 Hz, 6H), 3.05 (d, *J* = 4.6 Hz, 1H), 2.99 (d, *J* = 4.8 Hz, 1H), 2.51 – 2.40 (m, 2H), 2.38 – 2.19 (m, 6H), 2.11 – 1.86 (m, 16H), 1.27 – 1.21 (m, 24H), 1.14 – 0.94 (m, 16H); ^{13}C NMR (101 MHz, $CDCl_3$) δ 172.6, 172.5, 172.4, 171.8, 171.8, 171.6, 171.5, 159.8, 159.8, 131.1, 131.0, 131.0, 131.0, 127.5, 127.4, 127.1 (q, *J* = 281.9 Hz) 114.2, 113.6, 113.6, 113.4, 113.4, 61.8, 61.7, 61.7, 61.6, 61.6, 58.3, 58.3, 57.1, 56.5, 56.3, 55.2, 42.8, 42.7, 42.1, 42.1, 41.5, 41.4, 38.3, 38.0, 36.4, 36.3, 34.9, 34.1, 33.0 (q, *J* = 27.4 Hz), 33.0 (q, *J* = 27.7 Hz), 25.9, 25.7, 25.2, 25.1, 23.3, 23.3, 14.0, 14.0, 13.9; ^{19}F NMR (376 MHz, $CDCl_3$) δ -64.17 – -64.32 (m); HRMS (ESI) m/z [M + Na]$^+$ calcd for $C_{37}H_{49}F_3N_2O_9Na$: 745.3288 found : 745.3280.

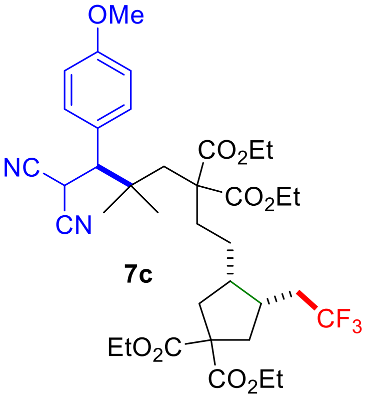

7c

Diethyl-3-(6-(4-chlorophenyl)-7,7-dicyano-3,3-bis(ethoxycarbonyl)-5,5-dimethylheptyl)-4-(2,2,2-trifluoroethyl)cyclopentane-1,1-dicarboxylate (**7d**): Yield 81% (88.3 mg); (dr = 1 : 1); R_f = 0.4 (ethyl acetate/n-hexane, 2 : 8); colorless oil; ^{1}H NMR (400 MHz, $CDCl_3$) δ 7.42 – 7.36 (m, 8H), 4.59 (d, *J* = 4.4 Hz, 1H), 4.55 (d, *J* = 4.5 Hz, 1H), 4.22 – 4.13 (m, 16H), 3.11 (d, *J* = 4.4 Hz, 1H), 3.04 (d, *J* = 4.5 Hz, 1H), 2.48 (ddd, *J* = 13.6, 6.7, 3.0 Hz, 2H), 2.36 – 2.19 (m, 6H), 2.14 – 1.88 (m, 16H), 1.25 – 1.20 (m, 24H), 1.16 – 0.91 (m, 16H); ^{13}C NMR (101 MHz, $CDCl_3$) δ 172.7, 172.6, 172.4, 171.9, 171.8, 171.5, 135.0, 135.0, 134.2, 134.0, 131.3, 129.1, 127.1 (q, *J* = 276.9 Hz), 113.4, 113.4, 113.1, 113.1, 61.9, 61.8, 61.8, 61.8, 61.8, 58.3, 58.2, 56.8, 56.5, 56.5, 55.8, 43.1, 42.1, 42.0, 41.7, 38.4, 38.4, 38.3, 38.2, 38.1, 38.0, 36.5 (q, *J* = 1.2 Hz), 36.4 (d, *J* = 2.1 Hz), 35.3, 34.5, 33.1 (q, *J* = 28.5 Hz), 33.0 (q, *J* = 28.6 Hz), 26.0, 25.9, 25.9, 24.9, 24.8, 23.3, 23.2, 14.1, 14.0, 14.0, 13.9; ^{19}F NMR (376 MHz, $CDCl_3$) δ -64.18 – -64.42; HRMS (ESI) m/z [M + H]$^+$ calcd for $C_{36}H_{47}ClF_3N_2O_8$: 727.2973 found : 727.2982.

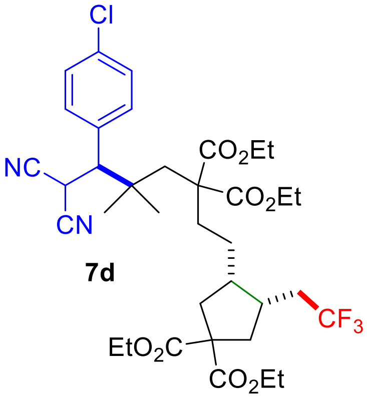

7d

Diethyl-3-(6-(4-bromophenyl)-7,7-dicyano-3,3-bis(ethoxycarbonyl)-5,5-dimethylheptyl)-4-(2,2,2-trifluoroethyl)cyclopentane-1,1-dicarboxylate (**7e**): Yield 84% (96.6 mg); (dr = 1 : 1); R_f = 0.5 (ethyl acetate/n-hexane, 2 : 8); colorless oil; ^{1}H NMR (400 MHz, $CDCl_3$) δ 7.43 – 7.38 (m, 8H), 4.54 (d, *J* = 4.8 Hz, 1H), 4.52 (d, *J* = 4.9 Hz, 1H), 4.18 – 4.08 (m, 16H), 3.08 (d, *J* = 4.7 Hz, 1H), 3.0 3 (d, *J* = 4.9 Hz, 1H), 2.46 (dd, *J* = 13.6, 6.5 Hz, 2H), 2.29 (m, 6H), 2.12 – 1.92 (m, 16H), 1.24 – 1.18 (m, 24H), 1.16 – 0.95 (m, 16H); ^{13}C NMR (126 MHz, $CDCl_3$) δ 172.6, 172.6, 172.4, 171.9, 171.8, 171.6, 171.6, 135.7, 135.6, 130.0, 128.9, 127.2 (q, *J* = 276.3 Hz), 113.5, 113.5, 113.3, 113.3, 61.8, 61.8, 61.8, 61.7, 61.7, 58.4, 58.4, 57.8, 57.0, 56.6, 42.8, 42.1, 42.1, 41.6, 38.5, 38.4, 38.3, 38.3, 38.1, 38.1, 36.4 (q, *J* = 2.0 Hz), 36.3 (q, *J* = 1.7 Hz), 35.0, 34.3, 33.1 (q, *J* = 28.6 Hz), 33.1 (q, *J* = 28.6 Hz), 26.0, 26.0, 25.9, 25.8, 25.0, 24.9, 23.5, 23.4, 14.1, 14.0, 14.0, 14.0; ^{19}F NMR (376 MHz, $CDCl_3$) δ -64.14 – -64.30 (m); HRMS (ESI) m/z [M + H]$^+$ calcd for $C_{36}H_{47}BrF_3N_2O_8$: 771.2468 found : 771.2472.

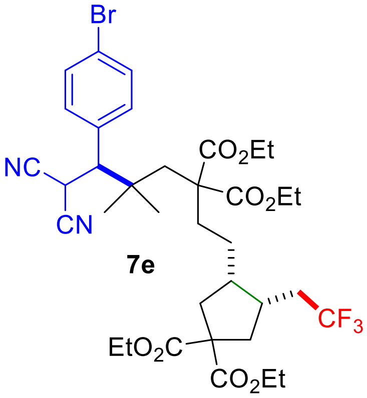

7e

Diethyl-3-(7,7-dicyano-3,3-bis(ethoxycarbonyl)-5,5-dimethyl-6-(perfluorophenyl)heptyl)-4-(2,2,2-trifluoroethyl)cyclopentane-1,1-dicarboxylate (**7f**): Yield 61% (71.6 mg); (dr = 1 : 1); R_f = 0.4 (ethyl acetate/n-hexane, 2 : 8); colorless oil; ^{1}H NMR (400 MHz, $CDCl_3$) δ 4.66 (d, *J* = 9.7 Hz, 1H), 4.61 (d, *J* = 9.5 Hz, 1H), 4.23 – 4.13 (m, 16H), 3.77 (dd, *J* = 12.9, 9.7 Hz, 2H), 2.53 – 2.44 (m, 2H), 2.36 – 2.17 (m, 6H), 2.11 – 1.80 (m, 16H), 1.25 – 1.18 (m, 24H), 1.15 – 1.01 (m, 16H); ^{13}C NMR (101 MHz, $CDCl_3$) δ 172.7, 172.7, 172.7, 172.7, 172.7, 172.4, 172.4, 172.4, 172.4, 172.4, 171.5, 171.4, 171.4, 171.4, 171.4, 171.4, 171.3, 145.7, 145.4, 145.4, 141.5, 138.1, 138.0, 133.7, 130.2, 128.6, 127.1, 127.1, 113.0, 112.9, 112.2, 112.1, 110.3, 110.3, 110.3, 61.9, 61.9, 61.8, 61.8, 61.8, 61.7, 58.3, 58.3, 58.3, 56.6, 56.6, 56.5, 56.4, 56.4, 56.4, 56.4, 56.4, 49.5, 49.0, 42.1, 42.0, 41.2, 40.5, 39.9, 39.9, 39.8, 39.8, 39.8, 38.4, 38.1, 36.6, 36.5, 34.7, 34.5, 33.1, 33.0; ^{19}F NMR (376 MHz, $CDCl_3$) δ -64.31 – -64.51 (m), -132.52 (dd, *J* = 54.2, 22.9 Hz), -136.51 (t, *J* = 22.7 Hz), -150.31 – -150.91 (m), -158.33 – -159.50 (m); HRMS (ESI) m/z [M + H]$^+$ calcd for $C_{36}H_{43}F_8N_2O_8$: 783.2892 found : 783.2898.

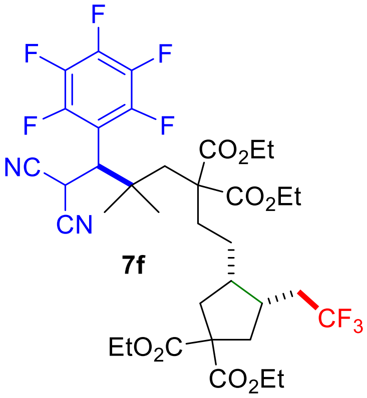

7f

Diethyl-3-(7,7-dicyano-3,3-bis(ethoxycarbonyl)-6-(4-(methoxycarbonyl)phenyl)-5,5-dimethylheptyl)-4-(2,2,2-trifluoroethyl)cyclopentane-1,1-dicarboxylate (**7g**): Yield 86% (96.2 mg); (dr = 1 : 1); R_f = 0.2 (ethyl acetate/n-hexane, 2 : 8); colorless oil; ^{1}H NMR (500 MHz, $CDCl_3$) δ 8.07 (dd, *J* = 8.6, 1.1 Hz, 4H), 7.54 (d, *J* = 7.8 Hz, 4H), 4.62 (d, *J* = 4.6 Hz, 1H), 4.58 (d, *J* = 4.8 Hz, 1H), 4.24 – 4.13 (m, 16H), 3.92 (s, 6H), 3.20 (d, *J* = 4.6 Hz, 1H), 3.14 (d, *J* = 4.8 Hz, 1H), 2.48 (m, 2H), 2.35 – 2.21 (m, 6H), 2.14 – 1.87 (m, 16H), 1.26 – 1.20 (m, 24H), 1.19 – 0.92 (m, 16H); ^{13}C NMR (101 MHz, $CDCl_3$) δ 172.7, 172.7, 172.4, 171.9, 171.8, 171.5, 166.6, 166.6, 140.7, 140.6, 130.8, 130.7, 130.1, 127.1 (q, *J* = 277.9 Hz), 113.4, 113.3, 113.0, 113.0, 62.0, 61.9, 61.9, 61.9, 61.8, 61.8, 58.3, 58.3, 57.3, 56.5, 56.5, 56.3, 52.4, 43.3, 42.1, 42.1, 41.8, 38.5, 38.4, 38.4, 38.3, 38.1, 38.1, 36.6 (q, *J* = 1.7 Hz), 36.5 (q, *J* = 0.7 Hz), 35.4, 34.6, 33.2 (q, *J* = 28.3 Hz), 33.1 (q, *J* = 28.6 Hz), 26.1, 26.1, 26.0, 26.0, 24.8, 24.7, 23.3, 23.2, 14.1, 14.0, 14.0, 14.0; ^{19}F NMR (471 MHz, $CDCl_3$) δ -64.29 – -64.48 (m); HRMS (ESI) m/z [M + H]$^+$ calcd for $C_{3038}H_{50}F_3N_2O_{10}$: 751.3418 found : 751.3425.

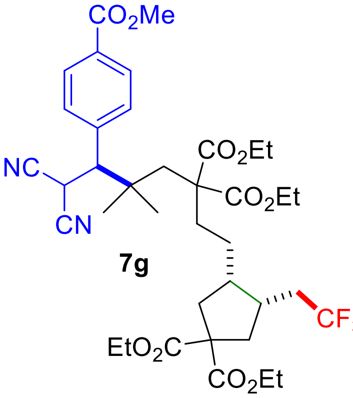

7g

Diethyl-3-(7,7-dicyano-3,3-bis(ethoxycarbonyl)-5,5-dimethyl-6-(thiophen-2-yl)heptyl)-4-(2,2,2-trifluoroethyl)cyclopentane-1,1-dicarboxylate (**7h**): Yield 57% (59.3 mg); (dr = 1 : 1); R_f = 0.4 (ethyl acetate/n-hexane, 2 : 8); colorless oil; ^{1}H NMR (500 MHz, $CDCl_3$) δ 7.33 (dd, *J* = 4.7, 2.6 Hz, 2H), 7.25 (dd, *J* = 5.6, 2.2 Hz, 2H), 7.07 (m, 2H), 4.59 (d, *J* = 3.7 Hz, 1H), 4.55 (d, *J* = 3.9 Hz, 1H), 4.27 – 4.14 (m, 16H), 3.49 (d, *J* = 3.7 Hz, 1H), 3.42 (d, *J* = 3.8 Hz, 1H), 2.50 – 2.46 (m, 2H), 2.36 – 2.22 (m, 6H), 2.12 – 1.84 (m, 16H), 1.28 – 1.23 (m, 24H), 1.21 – 0.95 (m, 16H); ^{13}C NMR (101 MHz, $CDCl_3$) δ 172.7, 172.7, 172.4, 172.0, 171.9, 171.5, 171.5, 136.5, 136.4, 128.9, 128.8, 127.3, 127.3, 127.2 (q, *J* = 277.6 Hz), 126.2, 126.1, 113.2, 113.2, 113.1, 113.1, 62.0, 61.9, 61.9, 61.9, 61.9, 61.8, 61.8, 61.8, 58.4, 58.3, 56.6, 56.5, 56.5, 56.5, 53.4, 52.3, 43.1, 42.1, 42.1, 41.7, 38.6, 38.6, 38.5, 38.4, 38.1, 38.1, 36.5, 35.5, 34.7, 33.2 (q, *J* = 27.9 Hz), 33.1 (d, *J* = 27.6 Hz), 26.2, 26.2, 26.0, 25.9, 25.7, 23.4, 23.3, 14.1, 14.1, 14.1, 14.0, 14.0; ^{19}F NMR (471 MHz, $CDCl_3$) δ -64.29 – -64.41 (m); HRMS (ESI) m/z [M + H]$^+$ calcd for $C_{34}H_{46}F_3N_2O_8S$: 699.2927 found : 699.2932.

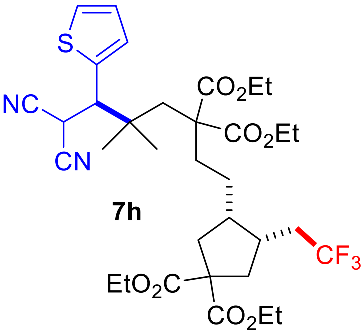

7h

Diethyl-s3-(7,7-dicyano-3,3-bis(ethoxycarbonyl)-5,5-dimethyl-6-phenylheptyl)-4-(2,2-difluoroethyl)cyclopentane-1,1-dicarboxylate (**7i**): Yield 27% (27.0 mg); (dr = 1 : 1); R_f = 0.3 (ethyl acetate/n-hexane, 2 : 8); colorless oil; ^{1}H NMR (400 MHz, $CDCl_3$) δ 7.46 – 7.38 (m, 10H), 5.99 – 5.66 (m, 2H), 4.54 (d, *J* = 4.8 Hz, 1H), 4.51 (d, *J* = 4.9 Hz, 1H), 4.17 (m, 16H), 3.08 (d, *J* = 4.8 Hz, 1H), 3.04 (d, *J* = 4.9 Hz, 1H), 2.41 – 2.21 (m, 8H), 2.06 – 1.83 (m, 16H), 1.25 – 1.21 (m, 24H), 1.17 – 0.95 (m, 16H); ^{13}C NMR (126 MHz, $CDCl_3$) δ 172.7, 172.7, 172.5, 171.9, 171.8, 171.6, 135.7, 135.6, 130.0, 128.9, 117.2 (t, *J* = 239.5 Hz), 117.2 (t, *J* = 239.5 Hz), 113.5, 113.5, 113.3, 113.3, 62.4, 61.8, 61.8, 61.7, 58.5, 58.5, 57.6, 56.9, 56.6, 42.8, 42.5, 42.5, 41.8, 38.4, 38.4, 38.3, 38.3, 38.2, 38.1, 36.6 – 36.3 (m), 35.0, 34.5, 33.3 (t, *J* = 20.4 Hz), 33.3 (t, *J* = 20.5 Hz), 26.0, 26.0, 25.9, 25.0, 24.9, 23.8, 23.7, 14.1, 14.0, 14.0, 14.0; ^{19}F NMR (471 MHz, $CDCl_3$) δ -113.57 – -116.47 (m); HRMS (ESI) m/z [M + H]$^+$ calcd for $C_{36}H_{49}F_2N_2O_8$: 675.3457 found : 675.3465.

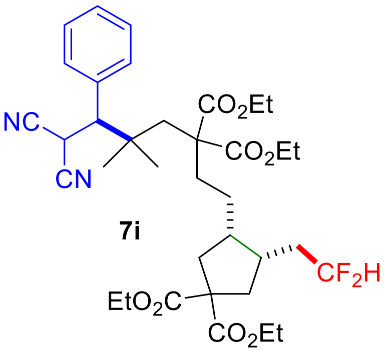

7i

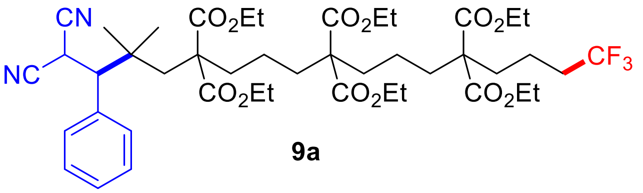

9a

Hexaethyl-17,17-dicyano-1,1,1-trifluoro-15,15-dimethyl-16-phenylheptadecane-5,5,9,9,13,13-hexacarboxylate (**9a**): Yield 19% (25.2 mg); R_f = 0.3 (ethyl acetate/n-hexane, 3 : 7); colorless oil; ^{1}H NMR (500 MHz, $CDCl_3$) δ 7.48 – 7.31 (m, 5H), 4.50 (d, *J* = 4.8 Hz, 1H), 4.19 – 4.10 (m, 12H), 3.04 (d, *J* = 4.8 Hz, 1H), 2.25 (d, *J* = 14.9 Hz, 1H), 2.12 – 2.02 (m, 3H), 1.96 – 1.77 (m, 10H), 1.49 – 1.41 (m, 2H), 1.27 – 1.21 (m, 18H), 1.13 (s, 3H), 1.09 – 1.02 (m, 4H), 0.97 (s, 3H); ^{13}C NMR (126 MHz, $CDCl_3$) δ 171.8, 171.5, 171.3, 171.2, 135.7, 130.0, 128.9, 127.0 (q, *J* = 277.2 Hz), 113.4, 113.3, 61.7, 61.7, 61.4, 61.3, 57.4, 57.3, 57.3, 56.8, 42.1, 38.2, 35.9, 34.0 (q, *J* = 28.6 Hz), 33.2, 33.1, 33.0, 31.9, 26.1, 25.8, 25.0, 19.5, 19.1, 17.2 (q, *J* = 2.7 Hz), 14.2, 14.0, 14.0; ^{19}F NMR (471 MHz, $CDCl_3$) δ -66.28 (t, *J* = 10.8 Hz); HRMS (ESI) m/z [M + Na]$^+$ calcd for $C_{46}H_{63}F_3N_2O_{12}Na$ 903.4225 found 903.4237.

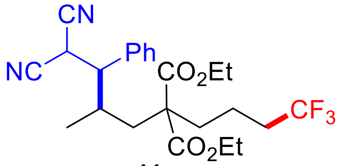

11a

Diethyl-2-(4,4-dicyano-2-methyl-3-phenylbutyl)-2-(4,4,4-trifluorobutyl) malonate (**11a**): Yield 71% (49.4 mg); (dr = 1 : 1); Isomer (**11a'**): R_f = 0.4 (ethyl acetate/n-hexane, 2 : 8); colorless oil; ^{1}H NMR (500 MHz, $CDCl_3$) δ 7.46 – 7.33 (m, 3H), 7.27 (d, *J* = 5.1 Hz, 2H), 4.40 (d, *J* = 7.3 Hz, 1H), 4.33 – 4.13 (m, 4H), 3.07 (t, *J* = 7.4 Hz, 1H), 2.28 (dd, *J* = 13.8, 6.8 Hz, 1H), 2.22 – 2.06 (m, 3H), 2.04 – 1.91 (m, 2H), 1.67 (dd, *J* = 14.6, 8.8 Hz, 1H), 1.55 – 1.44 (m, 2H), 1.34 – 1.21 (m, 6H), 0.79 (d, *J* = 6.7 Hz, 3H); ^{13}C NMR (126 MHz, $CDCl_3$) δ 171.5, 171.1, 135.1, 129.2, 129.1, 128.9, 126.9 (q, *J* = 276.6 Hz), 112.3, 112.1, 62.1, 62.0, 56.9, 52.8, 38.5, 33.9 (q, *J* = 28.7 Hz), 33.1, 31.4, 27.5, 17.5, 17.4 (q, *J* = 3.7 Hz), 14.1, 14.1; ^{19}F NMR (471 MHz, $CDCl_3$) δ -66.23 (t, *J* = 10.7 Hz); Isomer (**11a''**): R_f = 0.3 (ethyl acetate/n-hexane, 2 : 8); colorless oil; ^{1}H NMR (500 MHz, $CDCl_3$) δ 7.46 – 7.35 (m, 3H), 7.32 (d, *J* = 6.8 Hz, 2H), 4.26 (d, *J* = 6.2 Hz, 1H), 4.24 – 4.05 (m, 4H), 2.95 (dd, *J* = 8.8, 6.4 Hz, 1H), 2.36 – 2.11 (m, 1H), 2.02 – 1.84 (m, 4H), 1.76 – 1.63 (m, 2H), 1.24 (t, *J* = 7.1 Hz, 3H), 1.18 (t, *J* = 7.1 Hz, 3H), 1.15 – 1.07 (m, 1H), 1.03 (d, *J* = 6.6 Hz, 3H), 1.00 – 0.88 (m, 1H); ^{13}C NMR (126 MHz, $CDCl_3$) δ 171.5, 170.9, 136.1, 129.4, 129.3, 128.4, 126.8 (q, *J* = 276.5 Hz), 112.2, 112.0, 61.8, 61.7, 56.3, 52.9, 36.1, 33.8 (q, *J* = 28.8 Hz), 31.8, 31.7, 27.3, 18.4, 16.9 (d, *J* = 2.9 Hz), 14.0, 14.0; ^{19}F NMR (471 MHz, $CDCl_3$) δ -66.23 (t, *J* = 10.7 Hz); HRMS (ESI) m/z [M + Na]$^+$ calcd for $C_{24}H_{29}F_3N_2O_4Na$ 489.1972, found 489.1957.

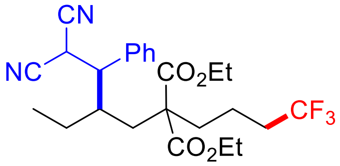

11b

Diethyl-2-(4,4-dicyano-2-ethyl-3-phenylbutyl)-2-(4,4,4-trifluorobutyl) malonate (**11b**): Yield 68% (49.0 mg); (dr = 1 : 1); R_f = 0.4 (ethyl acetate/n-hexane, 2 : 8); colorless oil; ^{1}H NMR (500 MHz, $CDCl_3$) δ 7.45 – 7.34 (m, 6H), 7.34 – 7.26 (m, 4H), 4.46 (dd, *J* = 6 .5, 1.5 Hz, 1H), 4.41 (d, *J* = 8.1 Hz, 1H), 4.32 – 4.08 (m, 8H), 3.26 (t, *J* = 7.4 Hz, 1H), 3.16 (dd, *J* = 8.1, 6.7 Hz, 1H), 2.23 – 1.80 (m, 13H), 1.55 – 1.43 (m, 3H), 1.36 – 1.21 (m, 17H), 1.14 – 1.04 (m, 1H), 1.02 – 0.91 (m, 3H), 0.72 (t, *J* = 7.3 Hz, 3H); ^{13}C NMR (126 MHz, $CDCl_3$) δ 171.7, 171.6, 171.2, 171.0, 135.2, 135.1, 129.3, 129.2, 129.1, 129.0, 128.7, 126.9 (q , *J* = 276.4 Hz), 126.8 (q, *J* = 276.4 Hz), 112.7, 112.5, 112.3, 112.2, 62.1, 62.0, 61.9, 57.0, 56.6, 56.5, 49.8, 48.0, 37.0, 36.1, 34.0 (d, *J* = 28.9 Hz), 33.9 (q, *J* = 28.9 Hz) 33.3, 33.0, 32.9, 28.6, 27.8, 27.48, 27.4, 27.0, 26.5, 25.9, 23.4, 22.8, 17.5 (q, *J* = 3.0 Hz), 17.1 (q, *J* = 2.9 Hz), 14.1, 14.0, 9.4, 8.7; ^{19}F NMR (471 MHz, $CDCl_3$) δ -66.16 – -66.35 (m); HRMS (ESI) m/z [M + Na]$^+$ calcd for $C_{25}H_{31}F_3N_2O_4Na$ 503.2128, found 503.2116.

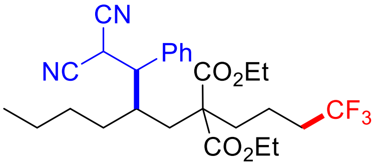

11c

Diethyl-2-(2-(2,2-dicyano-1-phenylethyl)hexyl)-2-(4,4,4-trifluoro butyl)malonate (**11c**): Yield 59% (45.1 mg); (dr = 1 : 1); R_f = 0.4 (ethyl acetate/n-hexane, 2 : 8); colorless oil; ^{1}H NMR (500 MHz, $CDCl_3$) δ 7.45 – 7.35 (m, 6H), 7.31 – 7.25 (m, 4H), 4.44 – 4.39 (m, 2H), 4.34 – 4.06 (m, 8H), 3.30 (dd, *J* = 8.5, 6.3 Hz, 1H), 3.19 (t, *J* = 7.4 Hz, 1H), 2.23 – 1.82 (m, 13H), 1.53 – 1.39 (m, 4H), 1.33 – 1.19 (m, 19H), 1.19 – 1.01 (m, 6H), 0.94 (t, *J* = 7.2 Hz, 3H), 0.74 (t, *J* = 6.9 Hz, 3H); ^{13}C NMR (126 MHz, $CDCl_3$) δ 171.7, 171.5, 171.3, 171.1, 135.1, 135.0, 129.2, 129.1, 129.0, 128.8, 126.9 (q, *J* = 276.5 Hz), 112.8, 112.5, 112.3, 112.3, 62.1, 62.0, 61.9, 57.1, 56.6, 50.0, 48.5, 36.2, 35.6, 34.1, 34.0 (q, *J* = 29.0 Hz), 33.9 (q, *J* = 28.8 Hz), 33.5, 33.3, 33.2, 31.2, 30.2, 27.7, 27.7, 26.9, 23.0, 23.0, 17.5 (q, *J* = 2.9 Hz), 17.2 (q, *J* = 3.0 Hz), 14.2, 14.1, 14.0, 13.9; ^{19}F NMR (471 MHz, $CDCl_3$) δ -66.20 – -66.34 (m); HRMS (ESI) m/z [M + Na]$^+$ calcd for $C_{27}H_{35}F_3N_2O_4Na$ 531.2441, found 531.2453.

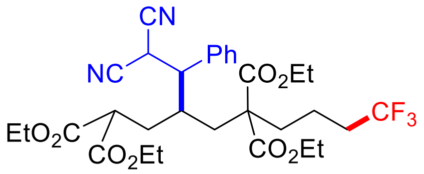

11d

Tetraethyl-3-(2,2-dicyano-1-phenylethyl)-9,9,9-trifluorononane-1,1,5,5-tetracarboxylate (**11d**): Yield 52% (48.8 mg); (dr = 1.1 : 1); R_f = 0.5 (ethyl acetate/n-hexane, 3 : 7); yellow oil; ^{1}H NMR (500 MHz, $CDCl_3$) δ 7.44 – 7.35 (m, 6.6H), 7.22 (t, *J* = 5.9 Hz, 4.4H), 4.63 (d, *J* = 10.5 Hz, 1.1H), 4.46 (d, *J* = 11.4 Hz, 1H), 4.42 – 4.12 (m, 17.6H), 3.60 (dd, *J* = 10.0, 5.0 Hz, 1.1H), 3.54 (dd, *J* = 11.2, 2.1 Hz, 1H), 3.39 – 3.31 (m, 2.1H), 2.25 – 1.87 (m, 17.3H), 1.83 – 1.71 (m, 2.2H), 1.48 – 1.26 (m, 30.8H); ^{13}C NMR (126 MHz, $CDCl_3$) δ 171.6, 171.3, 171.2, 171.1, 169.4, 169.2, 169.0, 168.9, 133.7, 133.3, 129.3, 129.2, 129.1, 128.9, 126.9 (q, *J* = 276.7 Hz), 126.8 (q, *J* = 276.4 Hz), 112.7, 112.6, 112.3, 112.2, 62.4, 62.3, 62.1, 61.9, 57.2, 56.8, 49.5, 49.2, 49.1, 48.3, 35.7, 34.3 – 33.4 (m), 32.5, 31.1, 30.9, 29.8, 26.5, 17.6 – 17.3 (m), 14.2, 14.1, 14.0; ^{19}F NMR (471 MHz, $CDCl_3$) δ -66.31 – -66.43 (m); HRMS (ESI) m/z [M + H]$^+$ calcd for $C_{31}H_{40}F_3N_2O_8$: 625.2737 found : 625.2745.

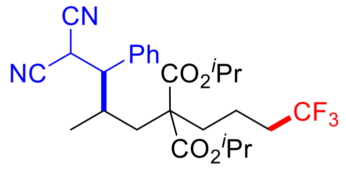

11e

Diisopropyl-2-(4,4-dicyano-2-methyl-3-phenylbutyl)-2-(4,4,4-trifluorobutyl)malonate (**11e**): Yield 69% (51.1 mg); (dr = 1.8 : 1); R_f = 0.5 (ethyl acetate/n-hexane, 2 : 8); colorless oil; ^{1}H NMR (500 MHz, $CDCl_3$) δ 7.45 – 7.36 (m, 8.4H) , 7.33 (d, *J* = 6.6 Hz, 3.6H), 7.27 (d, *J* = 5.5 Hz, 2H), 5.15 – 4.92 (m, 5.6H), 4.42 (d, *J* = 7.3 Hz, 1H), 4.27 (d, *J* = 6.2 Hz, 1.8H), 3.09 (t, *J* = 7.5 Hz, 1H), 2.95 (dd, *J* = 8.7, 6.3 Hz, 1.8H), 2.34 – 1.85 (m, 16.4H), 1.74 – 1.58 (m, 8H), 1.54 – 1.45 (m, 2H), 1.30 – 1.21 (m, 18H), 1.18 (t, *J* = 6.6 Hz, 9H), 1.15 – 1.09 (m, 1.8H), 1.05 (d, *J* = 6.6 Hz, 5.4H), 1.00 – 0.86 (m, 3.2H), 0.79 (d, *J* = 6.7 Hz, 3H); ^{13}C NMR (126 MHz, $CDCl_3$) δ 171.2, 171.1, 170.6, 170.4, 136.2, 135.2, 129.5, 129.4, 129.2, 129.0, 128.9, 128.4, 126.9 (q, *J* = 276.4 Hz), 126.8 (q, *J* = 276.4 Hz), 112.4, 112.3, 112.1, 112.0, 69.9, 69.7, 69.6, 69.5, 56.8, 56.2, 53.0, 52.9, 38.3, 36.2, 36.0, 34.0 (q, *J* = 28.9 Hz), 33.9 (q, *J* = 28.8 Hz), 33.1, 31.9, 31.8, 31.3, 27.5, 27.4, 21.7, 21.6, 18.4, 17.5, 17.4 (q, *J* = 3.0 Hz), 16.9 (q, *J* = 3.0 Hz); ^{19}F NMR (471 MHz, $CDCl_3$) δ -66.09 – -66.24 (m); HRMS (ESI) m/z [M + Na]$^+$ calcd for $C_{26}H_{33}F_3N_2O_4Na$: 517.2290 found : 517.2295.

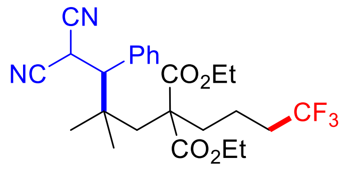

11f

Diethyl-2-(4,4-dicyano-2,2-dimethyl-3-phenylbutyl)-2-(4,4,4-trifluoro butyl)malonate (**11f**): Yield 86% (61.7 mg); R_f = 0.4 (ethyl acetate/n-hexane, 2 : 8); yellow oil; ^{1}H NMR (500 MHz, $CDCl_3$) δ 7.45 – 7.37 (m, 5H), 4.45 (d, *J* = 5.0 Hz, 1H), 4.27 – 4.04 (m, 4H), 3.06 (d, *J* = 5.0 Hz, 1H), 2.28 (d, *J* = 14.9 Hz, 1H), 2.09 (d, *J* = 14.9 Hz, 1H), 2.02 (ddd, *J* = 17.0, 10.7, 7.2 Hz, 4H), 1.41 (qt, *J* = 12.3, 6.3 Hz, 2H), 1.24 (td, *J* = 7.1, 1.3 Hz, 6H), 1.15 (s, 3H), 1.00 (s, 3H). ^{13}CNMR (126 MHz, $CDCl_3$) δ 171.5, 171.3, 135.5, 130.8, 130.1, 129.7, 129.0, 128.9, 126.8 (q, *J* = 276.2 Hz), 113.4, 113.2, 61.9, 61.8, 57.2, 56.7, 42.4, 38.3, 34.9, 33.8 (q, *J* = 28.9 Hz), 26.0, 25.9, 25.0, 17.6 (q, *J* = 3.1 Hz), 14.0, 13.9; ^{19}F NMR (471 MHz, $CDCl_3$) δ -66.23 (t, *J* = 10.7 Hz), -66.23 (t, *J* = 10.7 Hz); HRMS (ESI) m/z [M + Na]$^+$ calcd for $C_{25}H_{31}F_3N_2O_4Na$ 503.2128 found 503.2155.

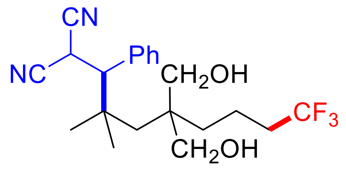

11g

2-(8,8,8-Trifluoro-4,4-bis(hydroxymethyl)-2,2-dimethyl-1-phenyloctyl) malononitrile (**11g**): Yield 87% (51.6 mg); R_f = 0.3 (ethyl acetate/n-hexane, 3 : 7); Yellow oil; ^{1}H NMR (500 MHz, $CDCl_3$) δ 7.51 – 7.43 (m, 2H), 7.42 – 7.35 (m, 3H), 4.67 (d, *J* = 4.7 Hz, 1H), 3.70 – 3.61 (m, 3H), 3.56 (d, *J* = 10.6 Hz, 1H), 3.23 (d, *J* = 4.6 Hz, 1H), 2.32 (s, 2H), 2.08 – 1.95 (m, 2H), 1.69 (d, *J* = 15.2 Hz, 1H), 1.50 – 1.37 (m, 5H), 1.25 (s, 3H), 1.07 (s, 3H); ^{13}CNMR (126 MHz, $CDCl_3$) δ 136.1, 130.0, 128.9, 128.9, 127.0 (d, *J* = 276.5 Hz), 113.9, 113.7, 68.6, 67.9, 56.9, 43.6, 39.4, 39.1, 34.4 (q, *J* = 28.5 Hz), 32.3, 32.1, 27.7, 27.2, 25.1, 24.9, 16.1 (q, *J* = 2.9 Hz). ^{19}F NMR (471 MHz, $CDCl_3$) δ -66.12 (t, *J* = 10.8 Hz); HRMS (ESI) m/z [M + Na]$^+$ calcd for $C_{21}H_{27}F_3N_2O_2Na$ 419.1917 found 419.1917.

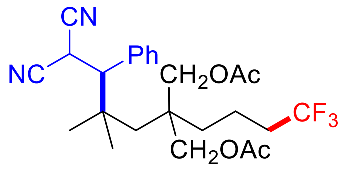

11h

2-(4,4-Dicyano-2,2-dimethyl-3-phenylbutyl)-2-(4,4,4-trifluorobutyl) propane-1,3-diyl diacetate (**11h**): Yield 89% (64.2 mg); R_f = 0.2 (ethyl acetate/n-hexane, 3 : 7); colorless solid; ^{1}H NMR (500 MHz, $CDCl_3$) δ 7.39 (d, *J* = 4.7 Hz, 5H), 4.32 (d, *J* = 5.5 Hz, 1H), 3.96 (d, *J* = 7.7 Hz, 4H), 2.97 (d, *J* = 5.4 Hz, 1H), 2.01 (d, *J* = 2.9 Hz, 8H), 1.67 (d, *J* = 14.8 Hz, 1H), 1.55 (d, *J* = 14.7 Hz, 1H), 1.48 – 1.35 (m, 4H), 1.23 (s, 3H), 1.18 (s, 3H). ^{13}C NMR (126 MHz, $CDCl_3$) δ 170.7, 135.6, 129.8, 129.1, 129.0, 126.9 (d, *J* = 276.5 Hz), 113.3, 113.1, 66.4, 58.9, 41.4, 39.2, 39.0, 34.1 (q, *J* = 28.7 Hz), 33.1, 32.5, 27.3, 26.4, 24.8, 20.9, 20.8, 16.0 (q, *J* = 3.0 Hz). ^{19}F NMR (471 MHz, $CDCl_3$) δ -66.05 (t, *J* = 10.7 Hz); HRMS (ESI) m/z [M + Na]$^+$ calcd for $C_{25}H_{31}F_3N_2O_4Na$: 503.2128 found : 503.2227.

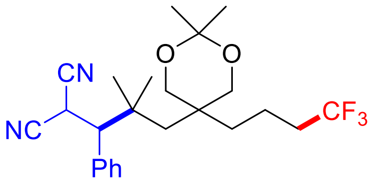

11i

2-(3-(2,2-Dimethyl-5-(4,4,4-trifluorobutyl)-1,3-dioxan-5-yl)-2,2-dimethyl-1-phenylpropyl) malononitrile (**11i**): Yield 91% (59.6 mg); R_f = 0.4 (ethyl acetate/n-hexane, 2 : 8); colorless oil; ^{1}H NMR (500 MHz, $CDCl_3$) δ 7.47 – 7.36 (m, 6.25H), 4.66 (d, J = 4.7 Hz, 0.25H), 4.57 (d, J = 5.2 Hz, 1H), 3.70 (d, J = 11.8 Hz, 1H), 3.66 – 3.55 (m, 4H), 3.24 (d, J = 4.7 Hz, 0.25H), 3.12 (d, J = 5.1 Hz, 1H), 2.17 (s, 1.25H), 2.06 – 1.96 (m, 2.5H), 1.77 (d, J = 15.2 Hz, 1H), 1.68 (d, J = 15.3 Hz, 0.25H), 1.55 – 1.34 (m, 12.25H), 1.24 (s, 3.25H), 1.12 (s, 3H), 1.07 (s, 0.75H); ^{13}C NMR (126 MHz, $CDCl_3$) δ 136.07, 135.79, 129.89, 128.97, 128.94, 128.87, 126.94 (q, J = 276.6 Hz), 113.86, 113.57, 113.19, 98.66, 69.22, 68.53, 68.39, 67.84, 57.39, 56.81, 43.64, 39.63, 39.54, 39.09, 39.00, 37.42, 34.39 (q, J = 28.2 Hz), 34.26 (q, J = 28.7 Hz), 32.73, 32.32, 31.04, 27.66, 27.41, 27.23, 26.75, 25.06, 24.94, 24.91, 22.98, 16.11 (q, J = 2.3 Hz), 15.95 (q, J = 2.9 Hz); ^{19}F NMR (471 MHz, $CDCl_3$) δ -66.03 – -66.17 (m); HRMS (ESI) m/z $[M + Na]^+$ calcd for $C_{24}H_{31}F_3N_2O_2Na$: 459.2235 found : 459.2242.

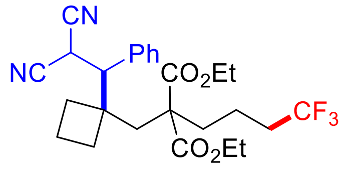

11j

Diethyl-2-((1-(2,2-dicyano-1-phenylethyl)cyclobutyl)methyl)-2-(4,4,4-trifluorobutyl)malonate (**11j**): Yield 76% (55.9 mg); R_f = 0.4 (ethyl acetate/n-hexane, 2 : 8); colorless oil; ^{1}H NMR (500 MHz, $CDCl_3$) δ 7.57 – 7.51 (m, 2H), 7.48 – 7.35 (m, 3H), 4.98 (d, J = 4.6 Hz, 1H), 4.35 – 4.19 (m, 3H), 4.05 (dq, J = 10.9, 7.1 Hz, 1H), 3.48 (d, J = 4.6 Hz, 1H), 2.81 – 2.70 (m, 1H), 2.61 (dd, J = 15.2, 1.1 Hz, 1H), 2.18 – 2.03 (m, 4H), 1.95 (td, J = 13.2, 4.8 Hz, 1H), 1.87 – 1.73 (m, 3H), 1.70 – 1.38 (m, 4H), 1.29 (td, J = 7.1, 4.3 Hz, 6H). ^{13}C NMR (126 MHz, $CDCl_3$) δ 172.6, 170.8, 136.6, 129.7, 129.4, 129.1, 128.9, 128.8, 126.9 (q, J = 276.6 Hz), 113.8, 113.4, 62.5, 62.0, 56.4, 50.1, 44.6, 42.7, 37.8, 33.9 (q, J = 28.8 Hz), 29.9, 29.8, 29.7, 29.5, 24.9, 24.7, 17.5 (q, J = 3.1 Hz), 15.0, 14.2, 14.1, 14.0; ^{19}F NMR (471 MHz, $CDCl_3$) δ -66.19 (t, J = 10.7 Hz); HRMS (ESI) m/z $[M + Na]^+$ calcd for $C_{26}H_{31}F_3N_2O_4Na$ 515.2128 found 515.2117.

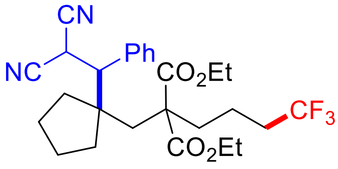

11k

Diethyl-2-((1-(2,2-dicyano-1-phenylethyl)cyclopentyl)methyl)-2-(4,4,4-trifluorobutyl)malonate (**11k**): Yield 71% (53.5 mg); R_f = 0.4 v(ethyl acetate/n-hexane, 2 : 8); colorless oil; ^{1}H NMR (500 MHz, $CDCl_3$) δ 7.53 – 7.30 (m, 5H), 4.86 (d, J = 3.8 Hz, 1H), 4.41 – 4.21 (m, 3H), 4.18 – 4.06 (m, 1H), 3.49 (d, J = 3.7 Hz, 1H), 2.55 (d, J = 15.2 Hz, 1H), 2.20 – 2.01 (m, 4H), 1.98 – 1.82 (m, 2H), 1.70 – 1.63 (m, 1H), 1.56 – 1.21 (m, 14H); ^{13}C NMR (126 MHz, $CDCl_3$) δ 172.5, 171.2, 136.2, 130.0, 128.9, 126.9 (q, J = 276.3 Hz), 114.0, 113.7, 62.5, 62.1, 56.5, 51.3, 49.8, 42.1, 37.7, 36.7, 35.3, 33.9 (q, J = 28.9 Hz), 25.8, 25.1, 24.1, 17.6 (q, J = 2.9 Hz), 14.2, 14.1; ^{19}F NMR (471 MHz, $CDCl_3$) δ -66.26 (t, J = 11.0 Hz); HRMS (ESI) m/z $[M + H]^+$ calcd for $C_{27}H_{34}F_3N_2O_4$: 507.2471 found : 507.2479.

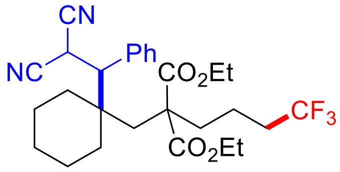

11l

Diethyl-2-((1-(2,2-dicyano-1-phenylethyl)cyclohexyl)methyl)-2-(4,4,4-trifluorobutyl)malonate (**11l**): Yield 68% (53.2 mg); R_f = 0.5 (ethyl acetate/n-hexane, 2 : 8); Yellow oil; ^{1}H NMR (500 MHz, $CDCl_3$) δ 7.56 – 7.30 (m, 5H), 4.56 (s, 1H), 4.29 – 4.11 (m, 4H), 3.66 (d, J = 3.1 Hz, 1H), 2.44 (d, J = 15.3 Hz, 1H), 2.30 (d, J = 15.3 Hz, 1H), 2.17 – 1.92 (m, 4H), 1.54 – 1.19 (m, 18H); ^{13}C NMR (126 MHz, $CDCl_3$) δ 172.1, 171.4, 135.5, 130.3, 130.2, 129.0, 128.9, 126.9 (q, J = 276.2 Hz), 113.8, 113.7, 62.2, 61.9, 56.4, 50.2, 41.0, 38.1, 36.8, 34.1, 33.9 (q, J = 29.0 Hz), 32.0, 25.4, 24.8, 21.7, 21.6, 17.7 (q, J = 2.5 Hz), 14.1, 14.0; ^{19}F NMR (471 MHz, $CDCl_3$) δ -66.18 (t, J = 10.6 Hz); HRMS (ESI) m/z $[M + Na]^+$ calcd for $C_{28}H_{35}F_3N_2O_4Na$ 543.2447 found 543.2446.

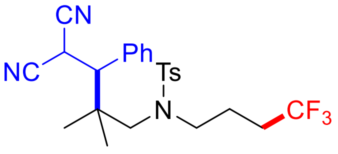

11m

N-(4,4-Dicyano-2,2-dimethyl-3-phenylbutyl)-4-methyl-N-(4,4,4-trifluorobutyl)benzenesulfonamide (**11m**): Yield 41% (30.2 mg); R_f = 0.3 (ethyl acetate/n-hexane, 3 : 7); colorless solid; ^{1}H NMR (500 MHz, $CDCl_3$) δ 7.72 – 7.66 (m, 2H), 7.48 (d, J = 6.9 Hz, 2H) , 7.46 – 7.39 (m, 3H), 7.36 (d, J = 8.0 Hz, 2H), 4.74 (d, J = 4.3 Hz, 1H), 3.47 (d, J = 14.8 Hz, 1H), 3.40 (d, J = 4.4 Hz, 1H), 3.16 (m, 1H), 3.00 (m, 1H), 2.76 (d, J = 14.9 Hz, 1H), 2.46 (s, 3H), 2.11 – 1.94 (m, 3H), 1.89 – 1.77 (m, 1H), 1.30 (s, 3H), 0.92 (s, 3H). ^{13}CNMR (126 MHz, $CDCl_3$) δ 144.60, 135.65, 134.70, 130.24, 129.80, 129.21, 129.10, 127.76, 126.80 (q, J = 276.9 Hz) 113.76, 112.99, 59.46, 52.00, 51.41, 39.71, 31.45 (q, J = 29.5 Hz), 26.49, 25.26, 23.70, 22.04 (q, J = 3.1 Hz), 21.69. ^{19}F NMR (471 MHz, $CDCl_3$) δ -65.73 (t, J = 10.6 Hz); HRMS (ESI) m/z $[M + Na]^+$ calcd for $C_{25}H_{29}F_3N_3O_2S$: 492.1933 found 492.1933.

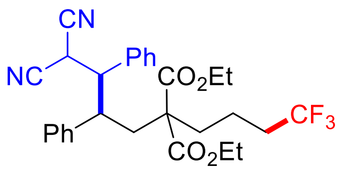

11n

Diethyl-2-(4,4-dicyano-2,3-diphenylbutyl)-2-(4,4,4-trifluorobutyl)malonate (**11n**): Yield 79% (62.5 mg); (dr = 1 : 1); Isomer (**11n'**): R_f = 0.5 (ethyl acetate/n-hexane, 2 : 8); colorless oil; ^{1}H NMR (400 MHz, $CDCl_3$) δ 7.34 – 7.23 (m, 4H), 7.23 – 7.13 (m, 3H), 6.91 (dd, *J* = 7.5, 1.3 Hz, 3H), 4.20 – 4.05 (m, 2H), 4.01 (t, *J* = 6.1 Hz, 1H), 3.99 – 3.81 (m, 2H), 3.51 (dd, *J* = 9.1, 6.5 Hz, 1H), 3.34 – 3.22 (m, 1H), 2.48 (dd, *J* = 14.7, 1.7 Hz, 1H), 2.31 (dd, *J* = 14.7, 10.4 Hz, 1H), 1.93 – 1.75 (m, 2H), 1.60 – 1.47 (m, 2H), 1.25 – 1.08 (m, 8H); ^{13}C NMR (126 MHz, $CDCl_3$) δ 170.8, 170.6, 137.3, 133.6, 129.4, 129.0, 128.7, 128.5, 128.1, 126.7 (q, *J* = 276.5 Hz), 112.6, 111.8, 62.1, 61.7, 57.4, 53.1, 43.6, 36.0, 33.7 (q, *J* = 29.0 Hz), 31.5, 27.4, 17.1 (q, *J* = 3.0 Hz), 14.1, 14.0; ^{19}F NMR (376 MHz, $CDCl_3$) δ -66.35 (t, *J* = 10.7 Hz); Isomer (**11n''**): R_f = 0.4 (ethyl acetate/n-hexane, 2 : 8); colorless oil; ^{1}H NMR (400 MHz, $CDCl_3$) δ 7.60 – 7.26 (m, 10H), 4.15 – 4.05 (m, 1H), 4.02 – 3.92 (m, 1H), 3.80 – 3.62 (m, 1H), 3.42 – 3.28 (m, 2H), 3.27 – 3.14 (m, 2H), 2.45 – 2.35 (m, 1H), 2.06 (d, *J* = 15.0 Hz, 1H), 1.85 – 1.57 (m, 5H), 1.11 (t, *J* = 7.1 Hz, 3H), 0.98 (t, *J* = 7.1 Hz, 3H), 0.89 – 0.76 (m, 1H); ^{13}C NMR (126 MHz, $CDCl_3$) δ 170.6, 170.0, 139.2, 135.1, 129.6, 129.5, 129.2, 128.6, 126.6 (q, *J* = 276.4 Hz), 111.9, 111.2, 61.5, 61.0, 56.2, 53.4, 43.7, 34.6, 33.6 (q, *J* = 28.8 Hz), 30.5, 28.3, 16.5, 13.8, 13.6; ^{19}F NMR (376 MHz, $CDCl_3$) δ -66.35 (t, *J* = 10.7 Hz); HRMS (ESI) m/z $[M + Na]^+$ calcd for $C_{29}H_{31}F_3N_2O_4Na$: 551.2134 found : 551.2138.

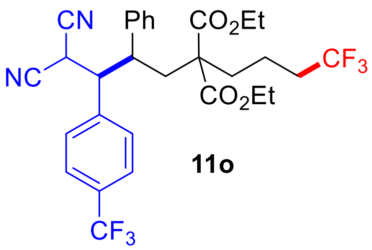

11o

Diethyl-2-(4,4-dicyano-2-phenyl-3-(4-(trifluoromethyl)phenyl)butyl)-2-(4,4,4-trifluorobutyl) malonate (**11o**): Yield 74% (66.0 mg); (dr = 1 : 1); Isomer (**11o'**): R_f = 0.5 (ethyl acetate/n-hexane, 2 : 8); colorless oil; ^{1}H NMR (400 MHz, $CDCl_3$) δ 7.52 (d, *J* = 8.3 Hz, 2H), 7.24 – 7.15 (m, 3H), 7.08 (d, *J* = 8.1 Hz, 2H), 6.90 (s, 2H), 4.21 – 4.06 (m, 3H), 4.04 – 3.84 (m, 2H), 3.62 – 3.54 (m, 1H), 3.35 – 3.26 (m, 1H), 2.48 (dd, *J* = 14.7, 1.7 Hz, 1H), 2.32 (dd, *J* = 14.7, 10.3 Hz, 1H), 1.93 – 1.78 (m, 2H), 1.68 – 1.55 (m, 2H), 1.24 (m, 5H), 1.16 (t, *J* = 7.1 Hz, 3H); ^{13}C NMR (126 MHz, $CDCl_3$) δ 170.53, 170.37, 137.73, 137.04, 130.93, 129.66, 128.58, 128.22, 125.50 (q, *J* = 3.8 Hz), 111.94, 111.27, 62.06, 61.67, 57.22, 52.71, 43.44, 35.95, 33.53 (q, *J* = 28.7 Hz), 31.41, 27.11, 16.96 (d, *J* = 3.0 Hz), 13.95, 13.83; ^{19}F NMR (471 MHz, $CDCl_3$) δ -62.80, -66.40, -66.42, -66.45.; Isomer (**11o''**): R_f = 0.4 (ethyl acetate/n-hexane, 2 : 8); colorless oil; ^{1}H NMR (400 MHz, $CDCl_3$) δ 7.76 (d, *J* = 8.2 Hz, 2H), 7.65 (d, *J* = 8.1 Hz, 2H), 7.41 (dd, *J* = 22.5, 6.9 Hz, 5H), 4.16 – 4.04 (m, 1H), 3.97 – 3.86 (m, 1H), 3.82 – 3.68 (m, 1H), 3.55 – 3.44 (m, 1H), 3.37 (d, *J* = 3.8 Hz, 1H), 3.34 – 3.17 (m, 2H), 2.40 (dd, *J* = 14.9, 10.7 Hz, 1H), 1.99 (d, *J* = 14.7 Hz, 1H), 1.89 – 1.56 (m, 4H), 1.11 (t, *J* = 7.1 Hz, 3H), 1.02 (t, *J* = 7.2 Hz, 3H), 0.95 – 0.75 (m, 2H); ^{13}C NMR (126 MHz, $CDCl_3$) δ 170.59, 170.08, 139.21, 138.79, 132.02 (q, *J* = 32.9 Hz), 129.88, 129.28, 129.05, 126.64 (q, *J* = 276.4 Hz), 126.60 (q, *J* = 3.7 Hz), 123.78 (q, *J* = 272.4 Hz), 111.69, 110.99, 61.77, 61.36, 56.44, 53.18, 43.70, 34.85, 33.64 (q, *J* = 28.9 Hz), 30.73, 28.22, 16.75 (q, *J* = 2.8 Hz), 13.90, 13.78; ^{19}F NMR (471 MHz, $CDCl_3$) δ -62.90, -66.43, -66.45, -66.48. HRMS (ESI) m/z $[M + H]^+$ calcd for $C_{29}H_{31}F_6N_2O_4$: 597.2183 found 597.2188.

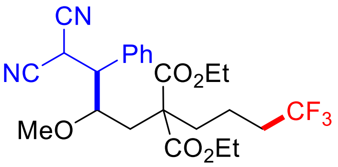

11p

Diethyl-2-(4,4-dicyano-2-methoxy-3-phenylbutyl)-2-(4,4,4-trifluorobutyl)malonate (**11p**): Yield 82% (59.4 mg); (dr = 1 : 1); R_f = 0.3 (ethyl acetate/n-hexane, 2 : 8); Yellow oil; ^{1}H NMR (500 MHz, $CDCl_3$) δ 7.49 – 7.27 (m, 10H), 4.40 (d, *J* = 4.8 Hz, 1H), 4.28 (d, *J* = 10.0 Hz, 1H), 4.23 – 4.08 (m, 8H), 3.87 – 3.76 (m, 2H), 3.53 – 3.45 (m, 3H), 3.43 – 3.39 (m, 4H), 3.25 (dd, *J* = 9.3, 4.8 Hz, 1H), 2.09 – 1.80 (m, 12H), 1.26 – 1.05 (m, 15H); ^{13}C NMR (126 MHz, $CDCl_3$) δ 171.0, 170.9, 170.8, 134.1, 134.0, 130.3, 130.1, 129.7, 129.5, 129.3, 129.2, 128.6, 126.84 (q, *J* = 276.5 Hz), 126.80 (q, *J* = 276.5 Hz), 112.4, 112.3, 112.2, 112.0, 78.3, 78.1, 62.0, 61.9, 61.8, 61.6, 60.0, 58.6, 55.8, 55.4, 52.6, 50.7, 36.2, 34.7, 34.2, 33.86 (q, *J* = 28.8 Hz), 33.84 (d, *J* = 28.8 Hz), 32.1, 31.9, 26.4, 26.3, 17.1 (q, *J* = 3.0 Hz), 17.0 (q, *J* = 2.9 Hz), 14.1, 14.0; ^{19}F NMR (471 MHz, $CDCl_3$) δ -66.19 – -66.35 (m); HRMS (ESI) m/z $[M + Na]^+$ calcd for $C_{24}H_{29}F_3N_2O_5Na$ 505.1921 found 505.1910.

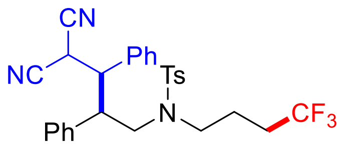

11q

N-(4,4-Dicyano-2,3-diphenylbutyl)-4-methyl-N-(4,4,4-trifluorobutyl) benzenesulfonamide (**11q**): Yield 45% (36.5 mg); (dr = 1 : 0.8); R_f = 0.2 (ethyl acetate/n-hexane, 3 : 7); colorless solid; ^{1}H ^{1}H NMR (400 MHz, $CDCl_3$) δ 7.65 – 7.54 (m, 5.4H), 7.47 – 7.28 (m, 9.2H), 7.25 – 7.18 (m, 5.4H), 7.11 (d, *J* = 8.1 Hz, 1.6H), 6.96 (dd, *J* = 7.7, 1.4 Hz, 2H), 6.86 – 6.83 (m, 1.6H), 4.29 (d, *J* = 8.8 Hz, 1H), 3.93 (td, *J* = 11.7, 3.1 Hz, 0.8H), 3.78 – 3.70 (m, 2H), 3.65 – 3.59 (m, 1.6H), 3.25 – 3.16 (m, 2.8H), 2.99 (t, *J* = 7.7 Hz, 2H), 2.94 – 2.80 (m, 2.4H), 2.57 – 2.47 (m, 1H), 2.46 – 2.42 (m, 3.8H), 2.37 (s, 2.4H), 1.94 – 1.80 (m, 3H), 1.59 – 1.47 (m, 2.4H); ^{13}C NMR (126 MHz, $CDCl_3$) δ 144.3, 143.8, 138.1, 136.2, 135.4, 134.8, 134.7, 133.7, 130.4, 130.2, 130.1, 129.8, 129.7, 129.3, 129.1, 128.9, 128.8, 128.3, 127.6, 127.5, 126.8 (q, *J* = 276.4 Hz), 112.5, 111.9, 111.8, 111.0, 53.6, 52.5, 50.4, 49.7, 49.3, 48.4, 47.9, 46.2, 31.3, 31.0 (q, *J* = 29.4 Hz), 30.9 (q, *J* = 29.1 Hz), 28.7, 27.6, 21.7, 21.6, 21.0 (q, *J* = 2.7 Hz), 20.8 (q, *J* = 2.7 Hz); ^{19}F NMR (471 MHz, $CDCl_3$) δ -65.98 (t, *J* = 10.9 Hz), -66.08 (t, *J* = 10.7 Hz); HRMS (ESI) m/z $[M + H]^+$ calcd for $C_{29}H_{29}F_3N_3O_2S$: 540.1933 found: 540.1924.

Diethyl-2-(5,5-dicyano-3,3-dimethyl-4-phenylpentyl)-2-(4,4,4-trifluorobutyl)malonate (**11r'**): Yield 49% (36.1 mg); R_f = 0.3 (ethyl acetate/n-hexane, 2 : 8); Colorless oil; ^{1}H NMR (500 MHz, $CDCl_3$) δ 7.48 – 7.30 (m, 5H), 4.35 (d, *J* = 5.4 Hz, 1H), 4.22 – 4.09 (m, 4H), 3.08 (d, *J* = 5.4 Hz, 1H), 2.11 – 1.98 (m, 2H), 1.96 – 1.79 (m, 4H), 1.45 – 1.33 (m, 2H), 1.29 – 1.18 (m, 8H), 1.15 (s, *J* = 5.6 Hz, 3H), 1.02 (s, 3H); ^{13}C NMR (126 MHz, $CDCl_3$) δ 171.2, 171.1, 135.7, 129.6, 128.9, 126.9 (q, *J* = 276.7 Hz), 113.4, 113.1, 61.7, 57.1, 54.8, 37.3, 35.1, 33.9 (q, *J* = 28.8 Hz), 31.9, 27.0, 25.5, 25.3, 24.9, 17.1 (q, *J* = 2.8 Hz), 14.2, 14.1; ^{19}F NMR (471 MHz, $CDCl_3$) δ -66.22 (t, *J* = 10.6 Hz); HRMS (ESI) m/z $[M + Na]^+$ calcd for $C_{26}H_{33}F_3N_2O_4Na$ 517.2285 found 817.2289.

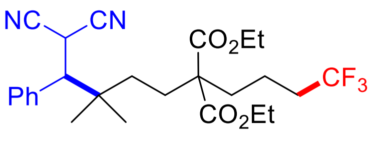

11r'

Diethyl-2-(4,4-dicyano-2-isopropyl-3-phenylbutyl)-2-(4,4,4-trifluorobutyl)malonate (**11r''**): Yield 29% (21.5 mg); (dr = 1 : 1); R_f = 0.4 (ethyl acetate/n-hexane, 2 : 8); Colorless oil; ^{1}H NMR (500 MHz, $CDCl_3$) δ 7.44 – 7.28 (m, 10H), 4.65 (d, *J* = 6.9 Hz, 1H), 4.57 (d, *J* = 9.2 Hz, 1H), 4.35 – 4.12 (m, 8H), 3.39 (dd, *J* = 9.1, 4.7 Hz, 1H), 3.15 (t, *J* = 7.0 Hz, 1H), 2.22 – 1.52 (m, 20H), 1.38 – 1.25 (m, 12H), 1.10 (d, *J* = 7.0 Hz, 3H), 0.84 (d, *J* = 7.0 Hz, 3H), 0.76 (d, *J* = 6.9 Hz, 3H), 0.31 (d, *J* = 6.9 Hz, 3H); ^{13}C NMR (126 MHz, $CDCl_3$) δ 171.9, 171.4, 171.3, 136.5, 135.9, 130.9, 129.6, 129.2, 129.1, 129.0, 129.0, 128.9, 126.9 (q, *J* = 276.2 Hz), 126.8 (q, *J* = 276.4 Hz), 113.0, 112.8, 112.6, 112.5, 62.2, 62.1, 61.9, 57.1, 57.0, 49.3, 47.3, 41.4, 40.7, 33.93 (q, *J* = 28.8 Hz), 33.9, 33.88 (q, *J* = 28.8 Hz), 30.6, 28.8, 28.6, 28.2, 21.6, 20.4, 18.2, 17.5 (q, *J* = 2.6 Hz), 17.3 (q, *J* = 2.8 Hz) 14.1, 14.0; ^{19}F NMR (471 MHz, $CDCl_3$) δ -66.21 – -66.36 (m); HRMS (ESI) m/z $[M + H]^+$ calcd for $C_{26}H_{34}F_3N_2O_4$ 495.2465 found 495.2492.

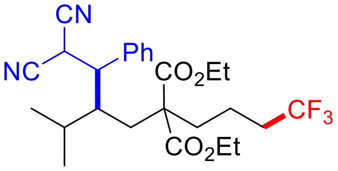

11r''

Diethyl-2-(4,4-dicyano-2,2-dimethyl-3-phenylbutyl)-2-(4,4,5,5,6,6,7,7,7-nonafluoroheptyl)malonate (**11s**): Yield 72% (68.4 mg); R_f = 0.3 (ethyl acetate/n-hexane, 1 : 9); Yellow oil; ^{1}H NMR (500 MHz, $CDCl_3$) δ 7.52 – 7.26 (m, 5H), 4.44 (d, *J* = 4.9 Hz, 1H), 4.24 – 4.12 (m, 4H), 3.06 (d, *J* = 4.9 Hz, 1H), 2.29 (d, *J* = 14.9 Hz, 1H), 2.13 – 1.95 (m, 5H), 1.53 – 1.42 (m, 3H), 1.28 – 1.24 (m, 6H), 1.17 (s, 3H), 1.01 (s, 3H); ^{13}C NMR (126 MHz, $CDCl_3$) δ 171.6, 171.3, 135.5, 129.9, 129.0, 113.3, 113.2, 62.0, 61.9, 57.3, 56.8, 42.6, 38.3, 35.4, 30.96 (t, *J* = 22.1 Hz), 26.1, 26.0, 25.0, 16.0 (t, *J* = 3.5 Hz), 14.1, 14.0; ^{19}F NMR (471 MHz, $CDCl_3$) δ -81.03 (t, *J* = 9.7 Hz), -114.32 – -114.61 (m), -124.27 – -124.68 (m), -125.95 – -126.22 (m); HRMS (ESI) m/z $[M + Na]^+$ calcd for $C_{28}H_{31}F_9N_2O_4Na$ 653.2032 found 653.2027.

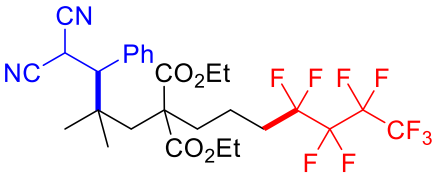

11s

Diethyl-2-(4,4-dicyano-2,2-dimethyl-3-phenylbutyl)-2-(4,4,5,5,6,6,7,7,8,8,9,9,9-tridecafluorononyl) malonate (**11t**): Yield 62% (67.5 mg); R_f = 0.4 (ethyl acetate/n-hexane, 1 : 9); Yellow oil; ^{1}H NMR (500 MHz, $CDCl_3$) δ 7.49 – 7.28 (m, 5H), 4.45 (d, *J* = 4.9 Hz, 1H), 4.28 – 4.09 (m, 4H), 3.06 (d, *J* = 4.9 Hz, 1H), 2.29 (d, *J* = 14.9 Hz, 1H), 2.17 – 1.95 (m, 5H), 1.52 – 1.41 (m, 2H), 1.29 – 1.24 (m, 6H), 1.17 (s, 3H), 1.01 (s, 3H); ^{13}C NMR (126 MHz, $CDCl_3$) δ 171.6, 171.3, 135.5, 129.9, 129.0, 113.3, 113.2, 62.0, 61.9, 57.3, 56.8, 42.6, 38.3, 35.4, 31.1 (t, *J* = 22.3 Hz), 26.1, 26.0, 25.1, 16.1 (t, *J* = 3.4 Hz), 14.1, 14.0; ^{19}F NMR (471 MHz, $CDCl_3$) δ -80.78 (t, *J* = 9.9 Hz), -114.08 – -114.42 (m), -121.93 (bs), -122.89 (bs), -123.54 (bs), -125.96 – -126.32 (m); HRMS (ESI) m/z $[M + H]^+$ calcd for $C_{30}H_{32}F_{13}N_2O_4N$ 731.2149 found 731.2158.

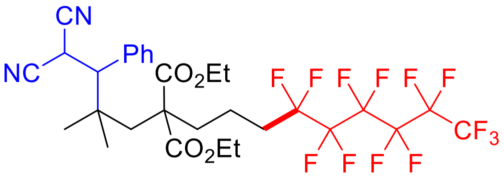

11t

Diethyl-2-(4,4-dicyano-2,2-dimethyl-3-phenylbutyl)-2-(4,4,5,5,6,6,7,7,8,8,9, 9,10,10,11,11,11-heptadecafluoroundecyl)malonate (**11u**): Yield 41% (51.2 mg); R_f = 0.5 (ethyl acetate/n-hexane, 1 : 9); Yellow oil; ^{1}H NMR (500 MHz, $CDCl_3$) δ 7.48 – 7.33 (m, 5H), 4.44 (d, *J* = 4.9 Hz, 1H), 4.29 – 4.09 (m, 4H), 3.06 (d, *J* = 4.9 Hz, 1H), 2.29 (d, *J* = 14.9 Hz, 1H), 2.16 – 1.93 (m, 5H), 1.47 (td, *J* = 11.1, 4.2 Hz, 2H), 1.30 – 1.23 (m, 6H), 1.17 (s, 3H), 1.01 (s, 3H); ^{13}C NMR (126 MHz, $CDCl_3$) δ 171.6, 171.3, 135.5, 129.9, 129.1, 129.0, 113.3, 113.2, 62.0, 61.9, 57.3, 56.8, 42.7, 38.3, 35.4, 31.1 (t, *J* = 21.8 Hz), 26.1, 26.0, 25.1, 16.1 (t, *J* = 3.5 Hz), 14.1, 14.0; ^{19}F NMR (471 MHz, $CDCl_3$) δ -80.72 (t, *J* = 10.0 Hz), -114.05 – -114.35 (m), -121.70 (bs), -121.90 (bs), -122.69 (bs), -123.47 (bs), -125.87 – -126.15 (m); HRMS (ESI) m/z $[M + H]^+$ calcd for $C_{32}H_{32}F_{17}N_2O_4$: 831.2091 found: 831.2089.

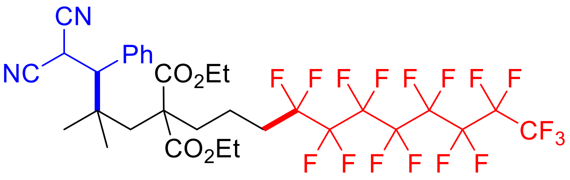

11u

Diethyl-2-(4,4-dicyano-2,2-dimethyl-3-phenylbutyl)-2-(4,4-difluorobutyl)malonate (**11v**): Yield 21% (14.6 mg); R_f = 0.4 (ethyl acetate/n-hexane, 1 : 9); Yellow oil; ^{1}H NMR (500 MHz, $CDCl_3$) δ 7.46 – 7.37 (m, 5H), 5.77 (tt, *J* = 56.6, 4.3 Hz, 1H), 4.46 (d, *J* = 4.9 Hz, 1H), 4.24 – 4.12 (m, 4H), 3.06 (d, *J* = 4.9 Hz, 1H), 2.28 (d, *J* = 14.9 Hz, 1H), 2.09 (d, *J* = 15.0 Hz, 1H), 2.04 – 1.96 (m, 2H), 1.86 – 1.76 (m, 2H), 1.27 – 1.25 (m, 8H), 1.16 (s, 3H), 1.00 (s, 3H); ^{13}C NMR (126 MHz, $CDCl_3$) δ 171.7, 171.5, 135.6, 129.9, 129.0, 116.7 (t, *J* = 239.5 Hz), 113.4, 113.2, 61.9, 61.8, 57.2, 56.8, 42.4, 38.3, 35.3, 34.0 (t, *J* = 21.0 Hz), 26.1, 26.0, 25.0, 17.5 (t, *J* = 5.9 Hz), 14.1, 14.1; ^{19}F NMR (376 MHz, $CDCl_3$) δ -116.09 (dt, *J* = 56.5, 17.4 Hz); HRMS (ESI) m/z $[M + Na]^+$ calcd for $C_{25}H_{32}F_2N_2O_4Na$ 485.2222 found 485.2228.

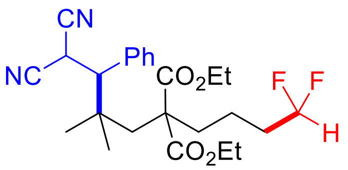

11v

Diethyl-2-(3-(4-chlorophenyl)-4,4-dicyano-2,2-dimethylbutyl)-2-(4,4,4-trifluorobutyl)malonate (**11w**): Yield 79% (61.0 mg); R_f = 0.5 (ethyl acetate/n-hexane, 2 : 8); colorless oil; ^{1}H NMR (500 MHz, $CDCl_3$) δ 7.39 (s, 4H), 4.47 (d, *J* = 4.6 Hz, 1H), 4.19 (m, 4H), 3.08 (d, *J* = 4.6 Hz, 1H), 2.27 (d, *J* = 15.0 Hz, 1H), 2.13 – 1.94 (m, 5H), 1.51 – 1.34 (m, 2H), 1.26 (td, *J* = 7.1, 2.9 Hz, 6H), 1.16 (s, 3H), 0.96 (s, 3H); ^{13}C NMR (126 MHz, $CDCl_3$) δ 171.6, 171.2, 135.2, 134.0, 131.2, 129.2, 126.8 (d, *J* = 276.4 Hz), 113.2, 112.9, 62.1, 62.0, 56.7, 56.1, 42.9, 38.3, 35.2, 33.8 (q, *J* = 29.0 Hz), 26.0, 26.0, 25.0, 17.6 (q, *J* = 3.2 Hz), 14.0, 14.0. ^{19}F NMR (471 MHz, $CDCl_3$) δ -66.22 (t, *J* = 10.6 Hz); HRMS (ESI) m/z $[M + Na]^+$ calcd for $C_{25}H_{30}ClF_3N_2O_4Na$ 537.1738 found 537.1724.

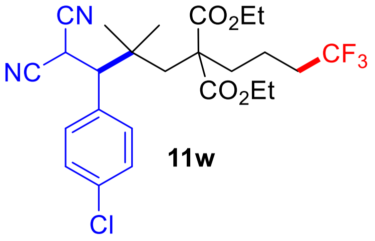

11w

Diethyl-2-(3-(2-chloro-4-fluorophenyl)-4,4-dicyano-2,2-dimethyl-butyl)-2-(4,4,4-trifluorobutyl) malonate (**11x**): Yield 86% (68.7 mg); R_f = 0.5 (ethyl acetate/n-hexane, 2 : 8); colorless oil; ^{1}H NMR (500 MHz, $CDCl_3$) δ 7.77 (dd, *J* = 8.9, 5.7 Hz, 1H), 7.29 – 7.23 (m, 1H), 7.11 (m, 1H), 4.43 (d, *J* = 5.3 Hz, 1H), 4.27 – 4.12 (m, 4H), 3.93 (d, *J* = 5.2 Hz, 1H), 2.21 (s, 2H), 2.05 (m, 4H), 1.50 – 1.37 (m, 2H), 1.25 (td, *J* = 7.2, 1.4 Hz, 6H), 1.16 (s, 3H), 1.03 (s, 3H). ^{13}C NMR (126 MHz, $CDCl_3$) δ 171.2, 162.0 (d, *J* = 253.1 Hz), 137.1 (d, *J* = 9.9 Hz), 130.1 (d, *J* = 8.8 Hz), 130.0, 129.5 (d, *J* = 3.8 Hz), 126.7 (q, *J* = 276.4 Hz), 118.0 (d, *J* = 24.4 Hz), 114.9 (d, *J* = 21.2 Hz), 112.9, 112.3, 77.3, 77.1, 76.8, 62.0, 61.9, 61.9, 56.6, 50.0, 41.8, 39.2, 34.2, 33.7 (q, *J* = 28.8 Hz), 25.0, 24.4, 17.6 (q, *J* = 3.0 Hz), 14.1, 13.9, 13.9; ^{19}F NMR (471 MHz, $CDCl_3$) δ -66.22 (t, *J* = 10.8 Hz), -108.52 – -110.85 (m); HRMS (ESI) m/z $[M + Na]^+$ calcd for $C_{25}H_{29}ClF_4N_2O_4Na$ 555.1644 found 555.1638.

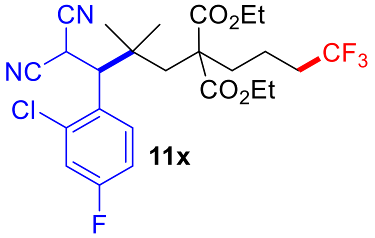

11x

Diethyl-2-(4,4-dicyano-2,2-dimethyl-3-(perfluorophenyl)butyl)-2-(4,4,4-trifluorobutyl)malonate (**11y**): Yield 80% (68.5 mg); R_f = 0.3 (ethyl acetate/n-hexane, 1 : 9); colorless oil; ^{1}H NMR (500 MHz, $CDCl_3$) δ 4.50 (d, *J* = 10.6 Hz, 1H), 4.28 – 4.13 (m, 4H), 3.76 (d, *J* = 9.8 Hz, 1H), 2.24 (d, *J* = 14.8 Hz, 1H), 2.13 (d, *J* = 14.9 Hz, 1H), 2.10 – 1.92 (m, 4H), 1.44 (p, *J* = 8.0 Hz, 2H), 1.26 (m, 6H), 1.11 (s, 6H); ^{13}C NMR (126 MHz, $CDCl_3$) δ 171.0, 170.9, 126.7 (q, *J* = 276.3 Hz), 112.6, 111.8, 110.2, 110.1, 76.8, 61.9, 61.9, 56.5, 49.1, 49.1, 41.0, 41.0, 39.7, 34.7, 33.7 (q, *J* = 28.9 Hz), 25.7, 25.7, 25.0, 25.0, 22.8, 22.8, 17.6 (q, *J* = 3.1 Hz), 14.1, 13.9; ^{19}F NMR (471 MHz, $CDCl_3$) δ -66.24 (td, *J* = 10.6, 6.7 Hz), -132.77 (dd, *J* = 22.3, 7.1 Hz), -136.49 – -136.65 (m), 148.70 – 134.15 (m), -150.19 (dt, *J* = 41.0, 20.4 Hz), -158.87 (dtd, *J* = 29.3, 21.8, 7.3 Hz), -159.15 (dtd, *J* = 30.5, 22.4, 7.6 Hz); HRMS (ESI) m/z $[M + Na]^+$ calcd for $C_{25}H_{26}F_8N_2O_4Na$ 593.1657 found 593.1655.

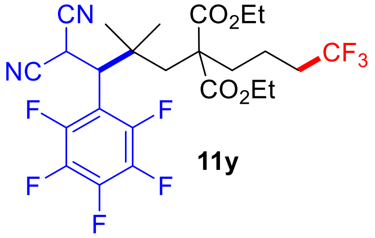

11y

Diethyl-2-(4,4-dicyano-2,2-dimethyl-3-(4-(trifluoromethyl)phenyl)-butyl)-2-(4,4,4-trifluorobutyl)malonate (**11z**): Yield 84% (69.1 mg); R_f = 0.4 (ethyl acetate/n-hexane, 2 : 8); colorless oil; ^{1}H NMR (500 MHz, $CDCl_3$) δ 7.68 (d, *J* = 8.1 Hz, 2H), 7.59 (d, *J* = 7.9 Hz, 2H), 4.54 (d, *J* = 4.5 Hz, 1H), 4.27 – 4.04 (m, 4H), 3.20 (d, *J* = 4.5 Hz, 1H), 2.31 (d, *J* = 14.9 Hz, 1H), 2.13 – 1.94 (m, 5H), 1.52 – 1.34 (m, 2H), 1.26 (td, *J* = 7.1, 4.2 Hz, 6H), 1.19 (s, 3H), 0.96 (s, 3H); ^{13}C NMR (126 MHz, $CDCl_3$) δ 171.5, 171.1, 139.4, 131.2 (q, *J* = 32.9 Hz), 130.3, 126.7 (q, *J* = 272.1 Hz), 125.8 (q, *J* = 3.8 Hz), 125.6, 123.8 (q, *J* = 272.1 Hz), 123.4, 113.0, 112.7, 62.0, 61.9, 56.5, 56.1, 42.9, 38.2, 35.3, 33.7 (q, *J* = 28.9 Hz), 26.0, 26.0, 25.8, 24.7, 17.5 (q, *J* = 3.0 Hz), 13.9, 13.8; ^{19}F NMR (471 MHz, $CDCl_3$) δ -62.86, -66.24 (t, *J* = 10.7 Hz); HRMS (ESI) m/z $[M + Na]^+$ calcd for $C_{26}H_{30}F_6N_2O_4Na$ 571.2002 found 571.1980.

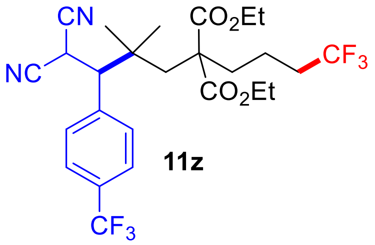

11z

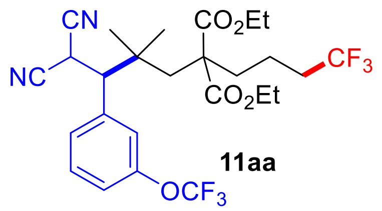

Diethyl-2-(3-(4-chlorophenyl)-4,4-dicyano-2,2-dimethylbutyl)-2-(4,4,4-trifluorobutyl)malonate (**11aa**): Yield 79% (66.9 mg); R_f = 0.4 (ethyl acetate/n-hexane, 2 : 8); colorless oil; ^{1}H NMR (500 MHz, $CDCl_3$) δ 7.46 (t, *J* = 7.9 Hz, 1H), 7.41 (d, *J* = 7.9 Hz, 1H), 7.28 (dd, *J* = 14.2, 5.9 Hz, 2H), 4.50 (dd, *J* = 4.7, 1.4 Hz, 1H), 4.27 – 4.10 (m, 4H), 3.13 (d, *J* = 4.6 Hz, 1H), 2.29 (d, *J* = 14.9 Hz, 1H), 2.02 (m, 5H), 1.52 – 1.32 (m, 2H), 1.26 (m, 6H), 1.18 (s, 3H), 0.97 (s, 3H); ^{13}C NMR (126 MHz, $CDCl_3$) δ 171.6, 171.2, 149.4 (d, *J* = 1.9 Hz), 137.8, 130.4, 126.8 (d, *J* = 276.3 Hz), 121.5, 120.6 (d, *J* = 257.8 Hz), 113.1, 112.8, 62.1, 62.0, 56.7, 56.1, 43.0, 38.3, 35.4, 33.8 (q, *J* = 28.9 Hz), 26.0, 26.0, 24.9, 17.6 (q, *J* = 3.0 Hz), 14.0, 14.0; ^{19}F NMR (471 MHz, $CDCl_3$) δ -57.92, -66.25 (t, *J* = 10.6 Hz); HRMS (ESI) m/z $[M + Na]^+$ calcd for $C_{26}H_{30}F_6N_2O_5Na$ 587.1951 found 587.1948.

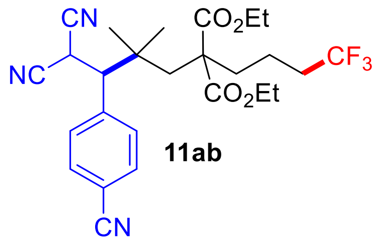

Diethyl-2-(4,4-dicyano-3-(4-cyanophenyl)-2,2-dimethylbutyl)-2-(4,4,4-trifluorobutyl)malonate (**11ab**): Yield 62% (47.0 mg); R_f = 0.3 (ethyl acetate/n-hexane, 2 : 8); colorless oil; ^{1}H NMR (500 MHz, $CDCl_3$) δ 7.76 – 7.70 (m, 2H), 7.59 (d, *J* = 7.9 Hz, 2H), 4.57 (d, *J* = 4.4 Hz, 1H), 4.27 – 4.11 (m, 4H), 3.22 (d, *J* = 4.4 Hz, 1H), 2.31 (d, *J* = 15.0 Hz, 1H), 2.05 (m, 4H), 1.96 (d, *J* = 14.9 Hz, 1H), 1.52 – 1.33 (m, 2H), 1.27 (td, *J* = 7.1, 4.7 Hz, 6H), 1.19 (s, 3H), 0.93 (s, 3H); ^{13}C NMR (126 MHz, $CDCl_3$) δ 171.6, 171.2, 140.9, 132.7, 130.8, 126.8 (q, *J* = 276.3 Hz), 118.1, 113.2, 113.0, 112.7, 77.4, 77.2, 76.9, 62.3, 62.1, 56.7, 56.0, 43.3, 38.4, 35.6, 33.8 (q, *J* = 28.9 Hz), 26.2, 26.1, 24.8, 17.6 (q, *J* = 3.0 Hz), 14.0, 14.0; ^{19}F NMR (471 MHz, $CDCl_3$) δ -66.19 (t, *J* = 10.7 Hz); HRMS (ESI) m/z $[M + Na]^+$ calcd for $C_{26}H_{30}F_3N_3O_4Na$ 528.2081 found 528.2092.

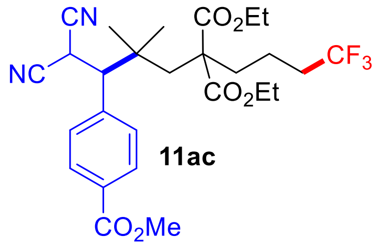

Diethyl-2-(4,4-dicyano-3-(4-(methoxycarbonyl)phenyl)-2,2-dimethylbutyl)-2-(4,4,4 - trifluorobutyl)malonate (**11ac**): Yield 64% (51.7 mg); R_f = 0.2 (ethyl acetate/n-hexane, 2 : 8); colorless oil; ^{1}H NMR (500 MHz, $CDCl_3$) δ 8.15 – 7.97 (m, 2H), 7.52 (d, *J* = 7.9 Hz, 2H), 4.52 (d, *J* = 4.9 Hz, 1H), 4.26 – 4.16 (m, 4H), 3.92 (s, 3H), 3.17 (d, *J* = 4.8 Hz, 1H), 2.28 (d, *J* = 14.9 Hz, 1H), 2.11 – 1.93 (m, 5H), 1.50 – 1.34 (m, 3H), 1.24 (td, *J* = 7.1, 2.4 Hz, 6H), 1.17 (s, 3H), 0.97 (s, 3H); ^{13}C NMR (126 MHz, $CDCl_3$) δ 171.5, 171.2, 166.5, 140.5, 130.8, 130.1, 126.8 (q, *J* = 276.3 Hz), 113.1, 112.9, 62.1, 61.9, 56.6, 56.6, 52.4, 42.8, 38.3, 35.2, 33.8 (q, *J* = 28.8 Hz), 26.0, 24.8, 17.6 (q, *J* = 3.0 Hz), 14.0, 14.0; ^{19}F NMR (471 MHz, $CDCl_3$) δ -66.22; HRMS (ESI) m/z $[M + Na]^+$ calcd for $C_{27}H_{33}F_3N_2O_6Na$ 561.2183 found 561.2196.

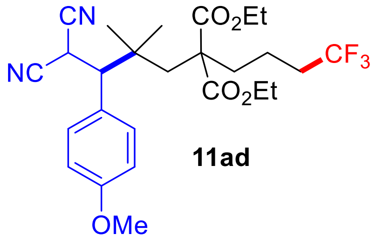

Diethyl-2-(4,4-dicyano-3-(4-methoxyphenyl)-2,2-dimethylbutyl)-2-(4,4,4-trifluorobutyl) malonate (**11ad**): Yield 44% (33.7 mg); R_f = 0.3 (ethyl acetate/n-hexane, 2 : 8); colorless oil; ^{1}H NMR (500 MHz, $CDCl_3$) δ 7.34 (d, *J* = 8.2 Hz, 2H), 6.94 – 6.89 (m, 2H), 4.41 (d, *J* = 4.8 Hz, 1H), 4.28 – 4.06 (m, 4H), 3.81 (s, 3H), 3.01 (d, *J* = 4.8 Hz, 1H), 2.25 (d, *J* = 14.9 Hz, 1H), 2.11 – 1.94 (m, 5H), 1.41 (m, 2H), 1.25 (td, *J* = 7.1, 1.3 Hz, 6H), 1.13 (s, 3H), 0.99 (s, 3H). $^{13}C\{^1H\}$ NMR (126 MHz, $CDCl_3$) δ 171.4, 171.2, 159.8, 130.9, 127.3, 126.7 (d, *J* = 276.2 Hz), 114.2, 113.3, 113.2, 61.8, 61.7, 56.6, 56.6, 55.2, 42.3, 38.3, 34.8, 33.7 (q, *J* = 28.8 Hz), 25.8, 25.8, 25.1, 17.5 (q, *J* = 3.0 Hz), 13.9, 13.9; ^{19}F NMR (471 MHz, $CDCl_3$) δ -66.23 (t, *J* = 10.8 Hz); HRMS (ESI) m/z $[M + Na]^+$ calcd for $C_{26}H_{33}F_3N_2O_5Na$ 533.2234 found 533.2216.

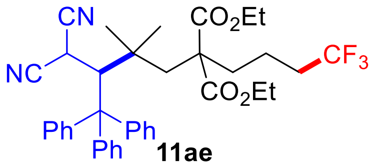

Diethyl-2-(3-(dicyanomethyl)-2,2-dimethyl-4,4,4-triphenylbutyl)-2-(4,4,4-trifluorobutyl) malonate (**11ae**): Yield 48% (46.7 mg); R_f = 0.4 (ethyl acetate/n-hexane, 1 : 9); colorless oil; ^{1}H NMR (500 MHz, $CDCl_3$) δ 7.42 (d, *J* = 8.4 Hz, 6H), 7.34 (t, *J* = 7.6 Hz, 6H), 7.24 (t, *J* = 7.3 Hz, 3H), 4.23 – 4.12 (m, 4H), 3.75 (s, 1H), 3.19 (dd, *J* = 14.8, 11.0 Hz, 1H), 2.56 (d, *J* = 15.0 Hz, 1H), 2.09 – 2.02 (m, 3H), 1.98 – 1.91 (m, 1H), 1.90 – 1.82 (m, 1H), 1.65 (d, *J* = 15.0 Hz, 1H), 1.47 (d, *J* = 10.9 Hz, 1H), 1.26 – 1.21 (m, 6H), 1.10 (s, 3H), 0.93 (s, 3H); ^{13}C NMR (126 MHz, $CDCl_3$) δ 171.3, 171.2, 146.0, 128.8, 127.0, 125.7 (q, *J* = 278.3 Hz), 114.0, 112.6, 61.7, 61.6, 56.7, 56.5, 48.3, 41.6, 38.7, 37.9, 34.6, 34.0, 33.9 (q, *J* = 28.4 Hz), 26.1, 23.2, 21.8, 17.8 (q, *J* = 2.6 Hz), 14.1, 14.1; ^{19}F NMR (471 MHz, $CDCl_3$) δ -66.19 (t, *J* = 10.5 Hz); HRMS (ESI) m/z $[M + Na]^+$ calcd for $C_{38}H_{41}F_3N_2O_4Na$: 669.2916 found : 669.2921.

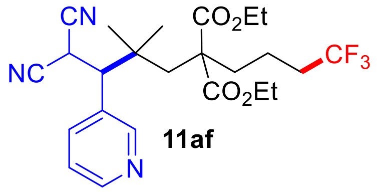

11af

Diethyl-2-(4,4-dicyano-2,2-dimethyl-3-(pyridin-3-yl)butyl)-2-(4,4,4-trifluorobutyl)malonate **(11af):** Yield 86% (62.1 mg); R_f = 0.3 (ethyl acetate/n-hexane, 3 : 7); Yellow oil; ^{1}H NMR (500 MHz, $CDCl_3$) δ 8.66 (dd, *J* = 4.9, 1.6 Hz, 1H), 8.61 (d, *J* = 2.5 Hz, 1H), 7.93 (dt, *J* = 8.2, 2.0 Hz, 1H), 7.38 (dd, *J* = 8.0, 4.8 Hz, 1H), 4.57 (d, *J* = 4.3 Hz, 1H), 4.27 – 4.11 (m, 4H), 3.16 (d, *J* = 4.3 Hz, 1H), 2.31 (d, *J* = 14.9 Hz, 1H), 2.13 – 1.93 (m, 5H), 1.44 (m, 2H), 1.26 (td, *J* = 7.2, 1.7 Hz, 6H), 1.18 (s, 3H), 0.95 (s, 3H); = 14.9 Hz, 1H), 1.52 – 1.33 (m, 2H), 1.27 (td, *J* = 7.1, 4.7 Hz, 6H), 1.19 (s, 3H), 0.93 (s, 3H); ^{13}C NMR (126 MHz, $CDCl_3$) δ 171.6, 171.1, 151.6, 150.3, 136.4, 128.5, 126.8 (q, *J* = 276.3 Hz), 124.3, 113.0, 112.7, 62.3, 62.2, 56.7, 55.8, 43.3, 38.5, 35.4, 33.8 (q, *J* = 28.7 Hz), 26.3, 26.2, 24.8, 17.6 (q, *J* = 2.9 Hz), 14.1, 14.0; ^{19}F NMR (471 MHz, $CDCl_3$) δ -66.20 (t, *J* = 10.8 Hz); HRMS (ESI) m/z [M + Na]$^+$ calcd for $C_{24}H_{30}F_3N_3O_4Na$ 504.2081 found 504.2069.

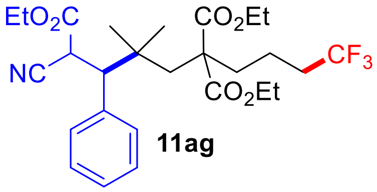

11ag

Triethyl-1-cyano-9,9,9-trifluoro-3,3-dimethyl-2-phenylnonane-1,5,5-tricarboxylate (**11ag**): Yield 87% (68.9 mg); (dr = 1 : 0.25); R_f = 0.4 (ethyl acetate/n-hexane, 2 : 8); colorless oil; ^{1}H NMR (500 MHz, $CDCl_3$) δ 7.42 (d, *J* = 6.2 Hz, 2H), 7.34 – 7.27 (m, 3.5H) , 7.19 (d, *J* = 6.6 Hz, 0.5H), 4.19 – 3.90 (m, 8.75H), 3.25 (d, *J* = 9.4 Hz, 0.25H), 3.13 (d, *J* = 4.7 Hz, 1H), 2.27 – 2.18 (m, 2.5H), 2.08 – 1.99 (m, 5H), 1.45 – 1.36 (m, 2.5H), 1.24 – 1.19 (m, 7H), 1.13 (s, *J* = 5.6 Hz, 3.75H), 1.03 (s, 3H), 1.01 – 0.93 (m, 4H); ^{13}C NMR (126 MHz, $CDCl_3$) δ 171.6, 171.5, 166.0, 165.7, 137.7, 136.4, 130.2, 128.3, 128.2, 128.1, 127.9, 126.9 (q, *J* = 276.5 Hz), 117.6, 117.3, 62.9, 62.8, 61.6, 61.5, 58.3, 57.4, 56.8, 41.9, 40.4, 39.4, 39.0, 38.0, 37.9, 34.2, 33.9 (d, *J* = 28.8 Hz), 26.7, 26.0, 25.4, 25.1, 17.66 (d, *J* = 2.9 Hz), 14.0, 13.7, 13.6; ^{19}F NMR (471 MHz, $CDCl_3$) δ -66.19 – -66.38 (m); HRMS (ESI) m/z [M + Na]$^+$ calcd for $C_{27}H_{36}F_3NO_6Na$: 550.2392 found : 550.2397.

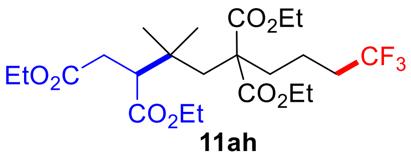

11ah

Tetraethyl-9,9,9-trifluoro-3,3-dimethylnonane-1,2,5,5-tetracarboxylate **(11ah)**: Yield 78% (58.3 mg); R_f = 0.3 (ethyl acetate/n- hexane, 2 : 8); Yellow oil; ^{1}H NMR (500 MHz, $CDCl_3$) δ 4.30 – 3.99 (m, 8H), 2.83 – 2.72 (m, 1H), 2.62 (d, *J* = 11.8 Hz, 1H), 2.49 (d, *J* = 16.5 Hz, 1H), 2.15 (d, *J* = 15.2 Hz, 1H), 2.10 – 1.94 (m, 5H), 1.50 – 1.35 (m, 2H), 1.31 – 1.17 (m, 12H), 1.04 – 0.82 (m, 6H); ^{13}C NMR (126 MHz, $CDCl_3$) δ 173.7, 172.5, 171.6, 171.6, 126.9 (q, *J* = 276.3 Hz), 61.5, 60.8, 60.5, 56.8, 52.8, 40.9, 35.9, 34.3, 33.9, 33.9 (q, *J* = 28.8 Hz), 32.7, 25.7, 24.7, 17.7 (q, *J* = 2.6 Hz), 14.3, 14.2, 14.0; ^{19}F NMR (471 MHz, $CDCl_3$) δ -66.33 (t, *J* = 11.1 Hz); HRMS (ESI) m/z [M + Na]$^+$ calcd for $C_{23}H_{37}F_3O_8Na$ 521.2333 found 521.2320.

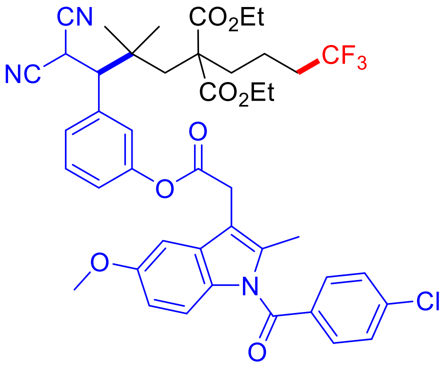

11ai

Diethyl-2-(3-(3-(2-(1-(4-chlorobenzoyl)-5-methoxy-2-methyl-1H-indol-3-yl)acetoxy)phenyl)-4,4-dicyano-2,2-dimethylbutyl)-2-(4,4,4-trifluorobutyl)malonate (**11ai**): Yield 79% (99.1 mg); R_f = 0.2 (ethyl acetate/n-hexane, 3 : 7); yellow solid; ^{1}H NMR (500 MHz, $CDCl_3$) δ 7.68 (d, *J* = 8.4 Hz, 2H), 7.48 (d, *J* = 8.4 Hz, 2H), 7.40 (t, *J* = 8.0 Hz, 1H), 7.30 (s, 1H), 7.19 (s, 1H), 7.12 (d, *J* = 8.1 Hz, 1H), 7.04 (d, *J* = 2.3 Hz, 1H), 6.89 (d, *J* = 9.0 Hz, 1H), 6.69 (dd, *J* = 9.0, 2.5 Hz, 1H), 4.47 (d, *J* = 4.6 Hz, 1H), 4.24 – 4.12 (m, 4H), 3.92 (s, 2H), 3.84 (s, 3H), 3.08 (d, *J* = 4.5 Hz, 1H), 2.45 (s, 3H), 2.27 (d, *J* = 15.0 Hz, 1H), 2.10 – 1.94 (m, 5H), 1.47 – 1.35 (m, 2H), 1.27 – 1.22 (m, 6H), 1.16 (s, 3H), 0.95 (s, 3H); ^{13}C NMR (126 MHz, $CDCl_3$) δ 173.3, 170.7, 167.9, 155.9, 151.3, 137.1, 136.9, 136.4, 133.2, 133.1, 132.4, 131.2, 130.2, 129.3, 126.9 (q, *J* = 276.6 Hz), 122.6, 121.6, 120.9, 113.0, 112.6, 111.9, 108.2, 101.9, 62.1, 62.0, 56.9, 55.7, 52.8, 38.5, 33.9 (q, *J* = 28.7 Hz), 33.1, 31.8, 31.4, 27.5, 17.5, 17.4 (q, *J* = 3.7 Hz), 14.1, 14.1, 13.3; ^{19}F NMR (471 MHz, $CDCl_3$) δ -66.18 (t, *J* = 10.6 Hz); HRMS (ESI) m/z [M + Na]$^+$ calcd for $C_{44}H_{45}ClF_3N_3O_8Na$: 858.2745 found : 858.2754.

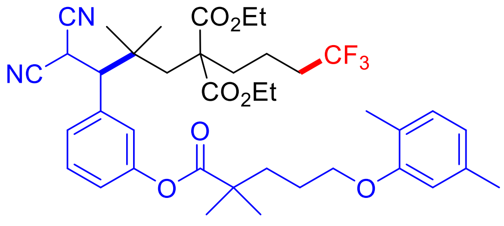

11aj

Diethyl-2-(4,4-dicyano-3-(3-((5-(2,5-dimethylphenoxy)-2,2-dimethylpentanoyl)oxy)phenyl)-2,2-dimethylbutyl)-2-(4,4,4-trifluorobutyl)malonate (**11aj**): Yield 68% (74.1 mg); R_f = 0.3 (ethyl acetate/n-hexane, 1 : 9); colorless oil; ^{1}H NMR (500 MHz, $CDCl_3$) δ 7.41 (t, *J* = 7.9 Hz, 1H), 7.34 – 7.27 (m, 1H), 7.13 – 7.04 (m, 2H), 7.01 (d, *J* = 7.5 Hz, 1H), 6.67 (d, *J* = 7.4 Hz, 1H), 6.63 (s, 1H), 4.43 (d, *J* = 5.0 Hz, 1H), 4.28 – 4.11 (m, 4H), 3.99 (t, *J* = 5.4 Hz, 2H), 3.06 (d, *J* = 5.0 Hz, 1H), 2.33 – 2.24 (m, 4H), 2.18 (d, *J* = 7.3 Hz, 3H), 2.09 – 1.98 (m, 5H), 1.90 – 1.85 (m, 3H), 1.48 – 1.41 (m, 1H), 1.38 (s, 6H), 1.26 (td, *J* = 7.1, 1.1 Hz, 6H), 1.16 (s, 3H), 0.98 (s, 3H); ^{13}C NMR (126 MHz, $CDCl_3$) δ 176.2, 171.6, 171.3, 157.0, 151.2, 137.0, 136.6,

130.5, 129.9, 126.9 (q, *J* = 276.4 Hz), 123.7, 122.1, 120.9, 113.2, 113.0, 112.1, 67.9, 62.0, 61.9, 56.7, 56.5, 42.7, 42.6, 38.4, 37.3, 35.2, 33.8 (q, *J* = 28.8 Hz), 26.1, 25.8, 25.4, 25.2, 24.8, 21.5, 17.6 (q, *J* = 2.9 Hz), 15.9, 14.0, 14.0; ^{19}F NMR (471 MHz, $CDCl_3$) δ -66.19 (t, *J* = 10.6 Hz); HRMS (ESI) m/z [M + Na]$^+$ calcd for $C_{40}H_{51}F_3N_2O_7Na$ 751.3546 found : 751.3543.

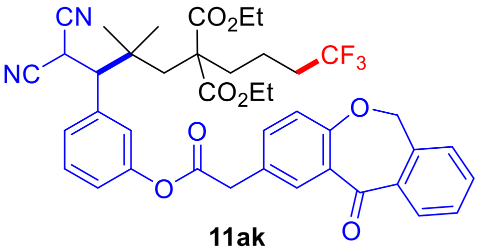

11ak

Diethyl-2-(4,4-dicyano-2,2-dimethyl-3-(3-(2-(11-oxo-6,11-dihydrodibenzo[b,e]oxepin-2-yl)acetoxy)phenyl)butyl)-2-(4,4,4-trifluorobutyl)malonate (**11ak**): Yield 79% (88.4 mg); R_f = 0.3 (ethyl acetate/n-hexane, 1 : 9); colorless oil; ^{1}H NMR (500 MHz, $CDCl_3$) δ 8.21 (d, *J* = 2.3 Hz, 1H), 7.90 (dd, *J* = 7.7, 1.2 Hz, 1H), 7.56 (td, *J* = 7.5, 1.4 Hz, 1H), 7.53 – 7.44 (m, 2H), 7.44 – 7.34 (m, 2H), 7.30 (d, *J* = 6.6 Hz, 1H), 7.24 – 7.12 (m, 2H), 7.07 (d, *J* = 8.4 Hz, 1H), 5.20 (s, 2H), 4.49 (d, *J* = 4.9 Hz, 1H), 4.22 – 4.09 (m, 4H), 3.90 (s, 2H), 3.09 (d, *J* = 4.9 Hz, 1H), 2.27 (d, *J* = 14.9 Hz, 1H), 2.07 – 1.99 (m, 4H), 1.71 – 1.67 (m, 1H), 1.45 – 1.36 (m, 2H), 1.26 – 1.21 (m, 6H), 1.16 (s, *J* = 8.9 Hz, 3H), 0.97 (s, *J* = 6.1 Hz, 3H); ^{13}C NMR (126 MHz, $CDCl_3$) δ 190.9, 171.6, 171.2, 169.7, 160.8, 150.8, 140.5, 137.0, 136.4, 135.6, 133.0, 132.7, 129.9, 129.6, 129.4, 128.0, 127.1, 125.4, 122.0, 121.4, 113.2, 113.0, 73.8, 62.0, 61.9, 56.6, 56.4, 40.3, 38.3, 33.9, 33.8 (q, *J* = 28.8 Hz), 26.1, 25.8, 25.7, 25.0, 24.8, 17.6 (q, *J* = 3.0 Hz), 14.0, 14.0; ^{19}F NMR (376 MHz, CHLOROFORM-*D*) δ -66.24 (t, *J* = 10.8 Hz); HRMS (ESI) m/z [M + Na]$^+$ calcd for $C_{41}H_{41}F_3N_2O_8Na$: 769.2713 found : 769.2705.

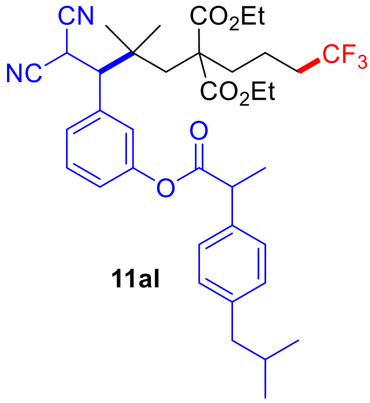

11al

Diethyl-2-(4,4-dicyano-3-(3-((2-(4-isobutylphenyl)propanoyl)oxy)phenyl)-2,2-dimethylbutyl)-2-(4,4,4-trifluorobutyl)malonate (**11al**): Yield 66% (67.4 mg); (dr = 1 : 1); R_f = 0.4 (ethyl acetate/n-hexane, 1 : 9); colorless oil; ^{1}H NMR (500 MHz, $CDCl_3$) δ 7.37 (t, *J* = 7.9 Hz, 1H), 7.29 (d, *J* = 8.0 Hz, 2H), 7.19 – 7.06 (m, 3H), 7.06 – 7.00 (m, 1H), 4.45 (t, *J* = 4.7 Hz, 1H), 4.25 – 4.11 (m, 4H), 3.94 (q, *J* = 7.1 Hz, 1H), 3.06 (d, *J* = 4.8 Hz, 1H), 2.47 (d, *J* = 7.2 Hz, 2H), 2.26 (d, *J* = 14.9 Hz, 1H), 2.10 – 1.93 (m, 5H), 1.91 – 1.82 (m, 1H), 1.60 (dd, *J* = 7.2, 2.3 Hz, 4H), 1.49 – 1.38 (m, 2H), 1.27 – 1.22 (m, 6H), 1.15 (d, *J* = 2.3 Hz, 3H), 0.96 (d, *J* = 2.7 Hz, 3H), 0.91 (d, *J* = 6.6 Hz, 6H); ^{13}C NMR (126 MHz, $CDCl_3$) δ 172.9, 172.9, 171.4, 171.1, 151.0, 140.9, 137.1, 137.0, 136.8, 136.8, 129.7, 129.6, 127.2, 127.2, 126.7 (q, *J* = 276.4 Hz), 121.9, 121.8, 113.0, 113.0, 112.8, 61.9, 61.8, 56.5, 56.3, 56.2, 45.3, 45.2, 45.0, 42.8, 42.7, 38.2, 35.2, 33.7 (q, *J* = 28.9 Hz), 30.2, 26.0, 26.0, 25.7, 24.7, 22.4, 22.4, 18.5, 18.5, 17.5 (q, *J* = 2.9 Hz), 13.9, 14.0; ^{19}F NMR (471 MHz, $CDCl_3$) δ -66.20 (t, *J* = 10.9 Hz); HRMS (ESI) m/z [M + Na]$^+$ calcd for $C_{38}H_{47}F_3N_2O_6Na$: 707.3284 found : 707.3292.

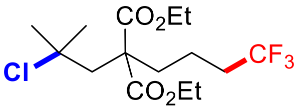

11am

Diethyl-2-(2-chloro-2-methylpropyl)-2-(4,4,4-trifluorobutyl)malonate (**11am**): Yield 52% (28.1 mg); R_f = 0.5 (ethyl acetate/n-hexane, 1 : 9); colorless oil; ^{1}H NMR (400 MHz, $CDCl_3$) δ 4.28 – 4.10 (m, 4H), 2.57 (s, 2H), 2.27 – 2.17 (m, 2H), 2.14 – 2.03 (m, 2H), 1.59 (s, 6H), 1.55 – 1.46 (m, 2H), 1.25 (t, *J* = 7.1 Hz, 6H); ^{13}C NMR (126 MHz, $CDCl_3$) δ 171.2, 127.0 (q, *J* = 276.3 Hz), 68.3, 61.8, 57.0, 45.6, 34.3, 33.9, 33.9 (q, *J* = 28.8 Hz), 17.5 (q, *J* = 3.0 Hz), 14.0; ^{19}F NMR (376 MHz, $CDCl_3$) δ -66.28 (t, *J* = 10.8 Hz); HRMS (ESI) m/z [M + Na]$^+$ calcd for $C_{15}H_{25}ClF_3O_4$: 361.1393 found 361.1393.

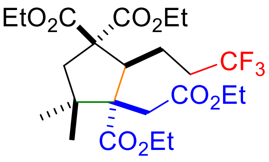

14a

Triethyl-(2S,3S)-3-(2-ethoxy-2-soxoethyl)-4,4-dimethyl-2-(3,3,3-trifluoropropyl)cyclopentane-1,1,3-tricarboxylate (**14a**): Yield 81% (60.2 mg); (dr = >20 : 1); R_f = 0.4 (ethyl acetate/n-hexane, 2 : 8); colorless oil; ^{1}H NMR (500 MHz, $CDCl_3$) δ 4.27 – 3.99 (m, 8H), 3.75 (dd, *J* = 9.7, 3.8 Hz, 1H), 2.97 (d, *J* = 16.3 Hz, 1H), 2.87 (d, *J* = 16.3 Hz, 1H), 2.65 (d, *J* = 14.3 Hz, 1H), 2.39 – 2.24 (m, 2H), 2.15 (d, *J* = 14.3 Hz, 1H), 1.96 – 1.87 (m, 1H), 1.34 – 1.25 (m, 10H), 1.21 (t, *J* = 7.2 Hz, 3H), 1.14 (s, 3H), 0.85 (s, 3H); ^{13}C NMR (126 MHz, $CDCl_3$) δ 173.1, 173.0, 172.4, 171.9, 127.4 (q, *J* = 276.1 Hz), 62.3, 62.0, 61.2, 60.9, 60.8, 59.7, 48.3, 47.5, 44.6, 34.8, 33.5 (q, *J* = 27.8 Hz), 25.8, 24.1, 20.7 (q, *J* = 3.2 Hz), 14.3, 14.1, 14.0, 13.9; ^{19}F NMR (471 MHz, $CDCl_3$) δ -67.09 (t, *J* = 10.6 Hz); HRMS (ESI) m/z [M + Na]$^+$ calcd for $C_{23}H_{35}F_3O_8Na$ 519.2176, found 519.2270.

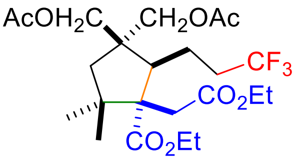

14b

((2R,3S)-3-(2-Ethoxy-2-oxoethyl)-3-(ethoxycarbonyl)-4,4-dimethyl-2-(3,3,3-trifluoropropyl)cyclopentane-1,1-diyl)bis(methylene) diacetate (**14b**): Yield 71% (52.8 mg); (dr = >20 : 1); R_f = 0.3 (ethyl acetate/n-hexane, 3 : 7); colorless solid; ^{1}H NMR (500 MHz, $CDCl_3$) δ 4.26 – 4.02 (m, 7H), 4.00 – 3.94 (m, 1H), 3.07 (d, *J* = 16.4 Hz, 1H), 2.86 (t, *J* = 6.7 Hz, 1H), 2.58 (d, *J* = 16.4 Hz, 1H), 2.40 – 2.15 (m, 2H), 2.08 (s, 3H), 2.03 (s, 3H), 1.76 – 1.62 (m, 2H), 1.57 – 1.46 (m, 2H), 1.26 (t, *J* = 7.1 Hz, 3H), 1.21 (t, *J* = 7.2 Hz, 3H), 1.08 (s, 3H), 0.95 (s, 3H); ^{13}C NMR (126 MHz, $CDCl_3$) δ 173.3, 172.1, 170.9, 170.8, 127.3 (q, *J* = 276.3 Hz), 69.6, 66.4, 61.0, 60.9, 60.0, 47.8, 46.4, 44.6, 43.8, 35.6, 34.1 (q, *J* = 27.8

Hz), 27.1, 25.2, 20.9, 20.8, 19.3 (q, J = 2.7 Hz), 14.2, 14.0; ^{19}F NMR (471 MHz, $CDCl_3$) δ -67.15 (t, J = 10.7 Hz); HRMS (ESI) m/z [M + H]$^+$ calcd for $C_{23}H_{36}F_3O_8$ 497.2362, found : 497.2368.

3-Ethyl-1,1-diisopropyl-(2S,3S)-3-(2-ethoxy-2-oxoethyl)-4,4-dimethyl-2-(3,3,3-trifluoropropyl)cyclopentane-1,1,3-tricarboxylate (**14c**): Yield 74% (58.4 mg); (dr = >20 : 1); R_f = 0.5 (ethyl acetate/n-hexane, 2 : 8); colorless oil; ^{1}H NMR (500 MHz, $CDCl_3$) δ 5.11 – 5.00 (m, 2H), 4.54 – 4.22 (m, 1H), 4.18 – 3.99 (m, 3H), 3.71 (dd, J = 8.9, 4.6 Hz, 1H), 2.93 (q, J = 16.3 Hz, 2H), 2.59 (d, J = 14.3 Hz, 1H), 2.44 – 2.23 (m, 2H), 2.14 (d, J = 14.3 Hz, 1H), 1.93 – 1.79 (m, 1H), 1.53 – 1.40 (m, 1H), 1.29 – 1.21 (m, 18H), 1.13 (s, 3H), 0.83 (s, 3H); ^{13}C NMR (126 MHz, $CDCl_3$) δ 173.1, 172.5, 172.5, 171.5, 127.4 (q, J = 276.4 Hz), 70.7, 69.5, 61.3, 60.8, 59.7, 48.4, 47.7, 44.6, 34.8, 33.8 (q, J = 27.8 Hz), 25.8, 24.1, 21.7, 21.6, 21.5, 20.6 (q, J = 2.8 Hz), 14.3, 14.1; ^{19}F NMR (471 MHz, $CDCl_3$) δ -66.89 (t, J = 10.8 Hz); HRMS (ESI) m/z [M + H]$^+$ calcd for $C_{25}H_{40}F_3O_8$: 525.2675, found : 525.2677.

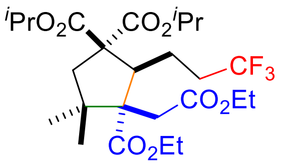

14c

Triethyl-(5S,6S)-5-(2-ethoxy-2-oxoethyl)-6-(3,3,3-trifluoropropyl)spiro [3.4]octane-5,7,7-tricarboxylate (**14d):** Yield 76% (57.4 mg); (dr = >20 : 1); R_f = 0.4 (ethyl acetate/n-hexane, 2 : 8); colorless oil; ^{1}H NMR (500 MHz, $CDCl_3$) δ 4.37 – 4.28 (m, 1H), 4.24 – 4.02 (m, 7H), 3.36 (dd, J = 9.2, 4.4 Hz, 1H), 2.84 (d, J = 17.2 Hz, 1H), 2.72 (d, J = 14.2 Hz, 1H), 2.67 – 2.52 (m, 3H), 2.38 – 2.25 (m, 2H), 2.07 – 1.94 (m, 1H), 1.85 – 1.69 (m, 2H), 1.68 – 1.60 (m, 2H), 1.51 – 1.43 (m, 1H), 1.32 – 1.20 (m, 13H); ^{13}C NMR (126 MHz, $CDCl_3$) δ 173.3, 172.1, 170.9, 170.8, 127.3 (q, J = 276.4 Hz), 69.6, 66.4, 61.0, 60.8, 60.0, 47.8, 46.3, 45.4, 44.6, 43.8, 38.6, 35.6, 34.1 (q, J = 27.8 Hz), 27.1, 25.2, 20.9, 20.8, 19.3 (q, J = 2.7 Hz), 14.2, 14.0; ^{19}F NMR (471 MHz, $CDCl_3$) δ -67.10 (t, J = 11.1 Hz); HRMS (ESI) m/z [M + H]$^+$ calcd for $C_{24}H_{36}F_3O_8$ 509.2362, found : 509.2336.

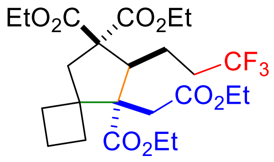

14d

1,1-Diethyl-3-methyl-(2S,3S)-3-(2-methoxy-2-oxoethyl)-4,4-dimethyl-2-(3,3,3trifluoropropyl) cyclopentane-1,1,3-tricarboxylate (**14e**): Yield 78% (54.6 mg); (dr = >20 : 1); R_f = 0.5 (ethyl acetate/n-hexane, 2 : 8); colorless oil; ^{1}H NMR (500 MHz, $CDCl_3$) δ 4.30 – 4.12 (m, 4H), 3.78 – 3.72 (m, 4H), 3.61 (s, 3H), 3.01 – 2.88 (m, 2H), 2.61 (d, J = 14.3 Hz, 1H), 2.39 – 2.24 (m, 2H), 2.18 (d, J = 14.3 Hz, 1H), 1.87 – 1.74 (m, 1H), 1.38 – 1.22 (m, 7H), 1.13 (s, 3H), 0.82 (s, 3H); ^{13}C NMR (126 MHz, $CDCl_3$) δ 173.6, 173.0, 171.9, 127.3 (q, J = 276.3 Hz), 62.4, 62.0, 61.3, 60.0, 52.0, 51.9, 48.3, 47.5, 44.7, 34.4, 33.4 (q, J = 27.9 Hz), 25.8, 24.0, 20.6 (q, J = 3.0 Hz), 14.1, 13.9; ^{19}F NMR (471 MHz, $CDCl_3$) δ -67.17 (t, J = 10.8 Hz); HRMS (ESI) m/z [M + H]$^+$ calcd for $C_{21}H_{31}F_3O_8Na$: 491.1869, found : 491.1874.

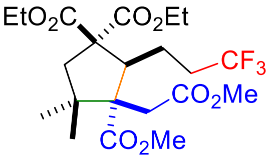

14e

1,1-Diethyl-3-isopropyl-(2S,3S)-3-(2-isopropoxy-2-oxoethyl)-4,4-dimethyl-2-(3,3,3-trifluoropropyl)cyclopentane-1,1,3-tricarboxylate (**14f**): Yield 79% (62.2 mg); (dr = >20 : 1); R_f = 0.5 (ethyl acetate/n-hexane, 2 : 8); colorless oil; ^{1}H NMR (500 MHz, $CDCl_3$) δ 5.13 – 4.99(m, 2H), 4.57 – 4.20 (m, 1H), 4.15 – 3.96 (m, 3H), 3.69 (dd, J = 8.7, 4.6 Hz, 1H), 2.97 (q, J = 16.5 Hz, 2H), 2.61 (d, J = 14.3 Hz, 1H) , 2.45 – 2.22 (m, 2H), 2.16 (d, J = 14.2 Hz, 1H), 1.92 – 1.78 (m, 1H), 1.58 – 1.39 (m, 1H), 1.31 – 1.18 (m, 18H), 1.16 (s, 3H), 0.87 (s, 3H); ^{13}C NMR (126 MHz, $CDCl_3$) δ 173.2, 172.4, 171.8, 171.8, 127.4 (q, J = 276.3 Hz), 68.5, 68.2, 62.3, 61.9, 61.1, 59.5, 48.3, 47.5, 44.5, 35.1, 33.5 (q, J = 28.0 Hz), 25.6, 24.0, 22.0, 21.9, 21.8, 21.5, 20.7 (q, J = 3.1 Hz), 14.1, 13.9; ^{19}F NMR (471 MHz, $CDCl_3$) δ -66.06 (t, J = 10.6 Hz); HRMS (ESI) m/z [M + H]$^+$ calcd for $C_{25}H_{40}F_3O_8$ 525.2675, found : 525.2669.

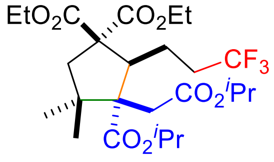

14f

1,1-Diethyl 3-propyl (2S,3S)-4,4-dimethyl-3-(2-oxo-2-propoxyethyl)-2-(3,3,3-trifluoropropyl) cyclopentane-1,1,3-tricarboxylate (**14g**): Yield 52% (40.6 mg); (dr = >20 : 1); R_f = 0.4 (ethyl acetate/n-hexane, 2 : 8); colorless oil; ^{1}H NMR (500 MHz, $CDCl_3$) δ 4.28 – 3.74 (m, 65H), 3.08 – 2.78 (m, 2H), 2.65 (d, J = 13.8 Hz, 1H), 2.39 – 2.06 (m, 3H), 2.01 – 1.82 (m, 1H), 1.75 – 1.54 (m, 5H), 1.34 – 1.19 (m, 6H), 1.14 (s, 2H), 1.05 – 0.71 (m, 10H); ^{13}C NMR (126 MHz, $CDCl_3$) δ 173.1, 173.1, 172.5, 171.8, 127.3 (q, J = 276.4 Hz), 66.7, 66.5, 62.3, 62.0, 61.2, 59.8, 48.3, 47.4, 44.6, 34.7, 33.5 (q, J = 28.1 Hz), 25.8, 24.0, 22.0, 22.0, 21.8, 20.6 (q, J = 3.2 Hz), 14.1, 13.9, 10.8, 10.4; ^{19}F NMR (471 MHz, $CDCl_3$) δ -66.85 (t, J = 10.7 Hz), -67.06 (t, J = 10.9 Hz); HRMS (ESI) m/z [M + H]$^+$ calcd for $C_{25}H_{40}F_3O_8$ 525.2675, found : 525.2677.

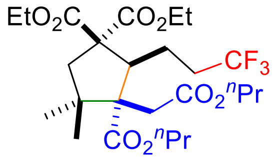

14g

Diethyl-(2S,3R)-3-(2-ethoxy-2-oxoethyl)-4,4-dimethyl-2-(3,3,3-trifluoro propyl)cyclopentane-1,1-dicarboxylate (**14h**): Yield 56% (35.4 mg); (dr = 1 : 1); R_f = 0.3 (ethyl acetate/n-hexane, 1 : 9); yellow oil; ^{1}H NMR (500 MHz, $CDCl_3$) δ 4.31 – 4.07 (m, 12H), 3.24 – 3.10 (m, 1H), 2.62 – 2.49 (m, 3H), 2.43 – 2.25 (m, 4H), 2.24 – 2.07 (m, 5H), 2.05 – 1.96 (m, 3H), 1.74 – 1.62 (m, 2H), 1.55 – 1.48 (m, 1H), 1.48 – 1.39 (m, 1H), 1.30 – 1.23 (m, 18H), 1.09 – 0.87 (m, 9H), 0.78 (s, 3H); ^{13}C NMR (126 MHz, $CDCl_3$) δ 174.0, 173.3, 173.3, 173.1, 171.7, 171.7, 62.5, 61.9, 61.9, 61.7, 61.7, 60.8, 60.7, 50.4, 49.8, 48.8, 48.4, 47.5, 46.2, 41.3, 39.9, 34.1, 32.9 (d, *J* = 28.5 Hz), 31.0, 29.9, 27.9, 25.1, 23.1, 22.5 (q, *J* = 3.4 Hz), 20.8 (q, *J* = 3.6 Hz), 14.3, 14.2, 14.1, 14.1, 14.0; ^{19}F NMR (471 MHz, $CDCl_3$) δ -67.08(t, *J* = 10.7 Hz); δ -66.93 (t, *J* = 10.3 Hz); HRMS (ESI) m/z $[M + H]^+$ calcd for $C_{20}H_{32}F_3O_6$ 425.2151, found : 425.2158.

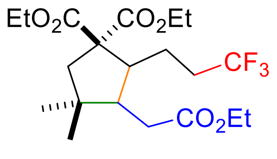

14h

1,1-Diethyl-3-methyl-(2S,3S)-3-(2-butoxy-2-oxoethyl)-4,4-dimethyl-2-(3,3,3-trifluoropropyl) cyclopentane-1,1,3-tricarboxylate (**14i'**) + *3-butyl-1,1-diethyl (2S,3S)-3-(2-methoxy-2-oxoethyl)-4,4-dimethyl-2-(3,3,3-trifluoropropyl)cyclopentane-1,1,3-tricarboxylate* (**14i''**): Yield 74% (56.4 mg); (**14i'** : **14i''** = 1 : 1, Inseparable mixture); R_f = 0.5 (ethyl acetate/n-hexane, 2 : 8); colorless oil; ^{1}H NMR (500 MHz, $CDCl_3$) δ 4.28 – 4.05 (m, 11H), 4.00 – 3.88 (m, 1H), 3.77 – 3.65 (m, 5H), 3.60 (s, 3H), 3.02 – 2.84 (m, 4H), 2.64 (t, *J* = 13.6 Hz, 2H), 2.37 – 2.22 (m, 4H), 2.15 (dd, *J* = 14.3, 7.6 Hz, 2H), 1.94 – 1.79 (m, 2H), 1.72 – 1.62 (m, 2H), 1.59 – 1.49 (m, 2H), 1.44 – 1.24 (m, 18H), 1.13 (d, *J* = 3.1 Hz, 6H), 0.95 – 0.82 (m, 12H); ^{13}C NMR (126 MHz, $CDCl_3$) δ 173.6, 173.1, 173.1, 172.9, 172.6, 171.9, 171.8, 127.3 (q, *J* = 276.3 Hz), 64.9, 62.3, 62.3, 62.0, 61.2, 61.2, 59.9, 59.9, 51.9, 48.3, 48.3, 47.5, 47.4, 44.7, 44.6, 34.7, 34.5, 33.5 (q, *J* = 27.7 Hz), 33.4 (q, *J* = 28.2 Hz), 30.6, 30.5, 25.8, 25.8, 24.0, 20.6, 19.5, 19.2, 14.1, 13.9, 13.8, 13.7; ^{19}F NMR (471 MHz, $CDCl_3$) δ -67.05 (t, *J* = 10.9 Hz), -67.19 (t, *J* = 10.9 Hz); HRMS (ESI) m/z $[M + H]^+$ calcd for $C_{24}H_{38}F_3O_8$ 511.2519, found : 511.2521.

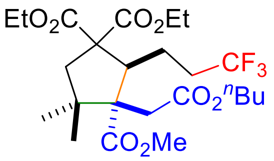

14i'

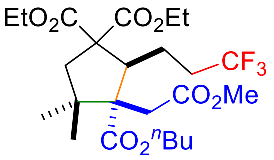

14i''

Ethyl-(7R,7aR)-7-(2-ethoxy-2-oxoethyl)-2,2,6,6-tetramethyl-4a-(4,4,4-trifluorobutyl)hexahydrocyclopenta[d][1,3]dioxine-7-carboxylate (**14j'**): Yield 43% (29.3 mg); (dr = >20 : 1); R_f = 0.4 (ethyl acetate/n-hexane, 2 : 8); colorless oil; ^{1}H NMR (500 MHz, $CDCl_3$) δ 4.2 8 (s, 1H), 4.21 – 4.01 (m, 4H), 3.59 (d, *J* = 11.3 Hz, 1H), 3.44 (d, *J* = 11.3 Hz, 1H), 2.83 (d, *J* = 16.9 Hz, 1H), 2.70 (d, *J* = 16.9 Hz, 1H), 2.17 – 1.98 (m, 4H), 1.65 (d, *J* = 13.7 Hz, 1H), 1.56 (s, 5H), 1.47 – 1.35 (m, 1H), 1.30 – 1.22 (m, 12H), 1.18 (s, 3H), 0.89 (s, 3H); ^{13}C NMR (126 MHz, $CDCl_3$) δ 174.6, 172.5, 127.3 (q, *J* = 276.5 Hz), 99.5, 82.4, 63.0, 61.3, 60.5, 60.3, 47.9, 47.4, 45.7, 34.4 (, *J* = 29.0 Hz), 34.3, 33.6, 27.5, 27.4, 26.8, 22.9, 17.6, 17.5, 14.4, 14.2; ^{19}F NMR (471 MHz, $CDCl_3$) δ -66.55 (t, *J* = 11.1 Hz); HRMS (ESI) m/z $[M + Na]^+$ calcd for $C_{22}H_{35}F_3O_6Na$: 475.2280, found : 475.2290.

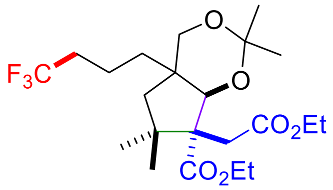

14j'

Ethyl-(1R,2S)-2-(2-ethoxy-2-oxoethyl)-3,3,8,8-tetramethyl-1-(3,3,3-trifluoro-propyl)-7,9-dioxaspiro[4.5]decane-2-carboxylate (**14j''**): Yield 34% (23.1 mg); (dr = >20 : 1); R_f = 0.3 (ethyl acetate/n-hexane, 2 : 8); colorless oil; ^{1}H NMR (500 MHz, $CDCl_3$) δ 4.35 – 4.23 (m, 1H), 4.21 – 4.05 (m, 3H), 3.97 (d, *J* = 11.6 Hz, 1H), 3.78 (d, *J* = 11.3 Hz, 1H), 3.57 (dd, *J* = 11.7, 1.3 Hz, 1H), 3.49 (dd, *J* = 11.4, 1.3 Hz, 1H), 3.14 (d, *J* = 16.2 Hz, 1H), 2.50 (t, *J* = 6.9 Hz, 1H), 2.41 – 2.24 (m, 3H), 2.02 (d, *J* = 14.5 Hz, 1H), 1.82 – 1.66 (m, 2H), 1.66 – 1.61 (m, 1H), 1.43 (s, 3H), 1.39 (s, 3H), 1.30 (t, *J* = 7.2 Hz, 3H), 1.25 (t, *J* = 7.2 Hz, 3H), 1.12 (s, 3H), 0.93 (s, 3H); ^{13}C NMR (126 MHz, $CDCl_3$) δ 173.3, 172.1, 127.3 (d, *J* = 276.6 Hz), 98.3, 70.1, 68.1, 61.0, 60.8, 59.9, 49.3, 47.7, 44.1, 41.1, 36.3, 34.3 (q, *J* = 27.7 Hz), 26.8, 25.0, 21.1, 19.0 (q, *J* = 2.5 Hz), 14.3, 14.0; ^{19}F NMR (471 MHz, $CDCl_3$) δ -66.98 (t, *J* = 11.0 Hz); HRMS (ESI) m/z $[M + H]^+$ calcd for $C_{22}H_{35}F_3O_6Na$: 475.2280, found : 475.2290.

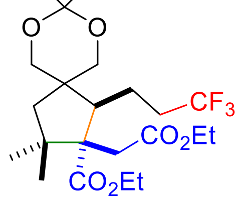

14j''

Diethyl-2-(4-(allylamino)-2,2-dimethyl-4-oxo-3-phenylbutyl)-2-(4,4,4-trifluorobutyl)malonate (**15**): Yield 90% (89.9 mg); colorless solid; ^{1}H NMR (500 MHz, $CDCl_3$) δ 7.48 – 7.37 (m, 2H), 7.31 – 7.25 (m, 3H), 5.79 – 5.71 (m, 1H), 5.62 (t, *J* = 5.6 Hz, 1H), 5.14 – 4.97 (m, 2H), 4.19 – 4.05 (m, 4H), 3.94 – 3.86 (m, 1H), 3.76 – 3.68 (m, 1H), 3.03 (s, 1H), 2.39 (d, *J* = 15.0 Hz, 1H), 2.16 (d, *J* = 15.0 Hz, 1H), 2.08 – 1.99 (m, 4H), 1.45 – 1.36 (m, 2H), 1.22 – 1.15 (m, 6H), 1.07 (s, 3H), 0.99 (s, 3H); ^{13}C NMR (126 MHz, $CDCl_3$) δ 172.1, 171.9, 171.9, 136.3, 134.3, 130.5, 128.0, 127.4, 127.0 (q, *J* = 276.5 Hz), 116.3, 65.3, 61.4, 61.3, 56.9, 41.9, 41.0, 37.9, 33.9 (q, *J* = 28.7 Hz), 33.9, 24.8, 24.6, 17.5, 17.6 (q, *J* = 2.9 Hz); ^{19}F NMR (471 MHz, $CDCl_3$) δ -66.26 (t, *J* = 10.9 Hz); HRMS (ESI) m/z $[M + H]^+$ calcd for $C_{26}H_{37}F_3NO_5$ 500.2618, found 500.2625.

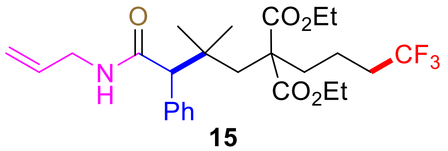

15

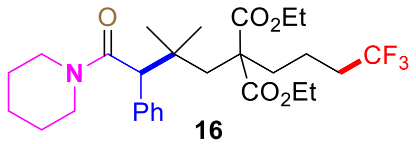

16

Diethyl-2-(2,2-dimethyl-4-oxo-3-phenyl-4-(piperidin-1-yl)butyl)-2-(4,4,4-trifluorobutyl) malonate (**16**): Yield 85% (89.7 mg); colorless oil; ^{1}H NMR (500 MHz, $CDCl_3$) δ 7.33 – 7.21 (m, 5H), 4.18 – 4.04 (m, 4H), 3.74 – 3.62 (m, 1H), 3.57 (s, 1H), 3.40 – 3.22 (m, 3H), 2.46 (d, J = 15.0 Hz, 1H), 2.21 (d, J = 15.0 Hz, 1H), 2.08 – 1.98 (m, 4H), 1.54 – 1.40 (m, 5H), 1.38 – 1.26 (m, 2H), 1.23 – 1.15 (m, 6H), 1.09 (s, 3H), 0.97 (s, 3H), 0.85 – 0.71 (m, 1H); ^{13}C NMR (126 MHz, $CDCl_3$) δ 172.1, 172.0, 170.5, 136.3, 130.8, 128.1, 127.0 (q, J = 276.4 Hz), 123.7, 61.2, 61.2, 59.2, 57.0, 47.4, 43.1, 41.0, 38.2, 34.0 (q, J = 28.7 Hz), 33.7, 26.1, 25.7, 25.6, 24.7, 24.6, 17.6 (q, J = 2.9 Hz), 14.0, 14.0; ^{19}F NMR (471 MHz, $CDCl_3$) δ -66.25 (t, J = 11.0 Hz); HRMS (ESI) m/z $[M + H]^+$ calcd for $C_{28}H_{41}F_3NO_5$ 528.2931, found 528.2938.

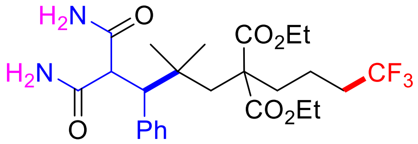

17

Diethyl-2-(5-amino-4-carbamoyl-2,2-dimethyl-5-oxo-3-phenylpentyl)-2-(4,4,4-trifluorobutyl)malonate (**17**): Yield 61% (63 mg); colorless solid; ^{1}H NMR (500 MHz, $CDCl_3$) δ 7.26 – 7.14 (m, 5H), 6.97 (s, 1H), 6.20 (s, 1H), 5.69 (s, 1H), 5.24 (s, 1H), 4.19 – 4.00 (m, 4H), 3.89 (d, J = 10.8 Hz, 1H), 3.28 (d, J = 10.9 Hz, 1H), 2.26 – 2.13 (m, 2H), 2.05 – 1.93 (m, 4H), 1.41 – 1.28 (m, 2H), 1.24 – 1.10 (m, 6H), 1.01 (s, 3H), 0.85 (s, 3H); ^{13}C NMR (126 MHz, $CDCl_3$) δ 173.4, 172.0, 172.0, 171.9, 139.1, 127.7, 127.1, 126.9 (q, J = 276.5 Hz) 61.5, 61.4, 59.7, 57.1, 56.9, 39.6, 38.2, 33.9 (q, J = 28.8 Hz), 33.5, 26.8, 25.5, 17.5 (q, J = 2.4 Hz), 14.0, 14.0; ^{19}F NMR (471 MHz, $CDCl_3$) δ -66.25 (t, J = 10.6 Hz); HRMS (ESI) m/z $[M + H]^+$ calcd for $C_{25}H_{35}F_3O_6N_2Na$ 539.2339, found 539.2343.

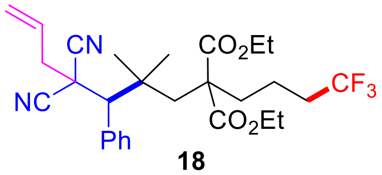

18

Diethyl-2-(4,4-dicyano-2,2-dimethyl-3-phenylhept-6-en-1-yl)-2-(4,4,4-trifluorobutyl)malonate (**18**): Yield 80% (83 mg); colorless oil; ^{1}H NMR (400 MHz, $CDCl_3$) δ 7.70 (dd, J = 6.7, 1.2 Hz, 1H), 7.49 – 7.27 (m, 3H), 7.04 (dt, J = 6.7, 2.0 Hz, 1H), 5.90 – 5.75 (m, 1H), 5.38 – 5.30 (m, 1H), 5.17 (dq, J = 16.9, 1.2 Hz, 1H), 4.25 – 4.04 (m, 4H), 2.91 (s, 1H), 2.46 – 2.26 (m, 4H), 2.06 – 1.94 (m, 4H), 1.42 – 1.32 (m, 2H), 1.26 – 1.16 (m, 12H); ^{13}C NMR (126 MHz, $CDCl_3$) δ 171.5, 171.4, 135.1, 133.5, 128.8, 128.7, 128.7, 128.5, 127.8, 126.9 (q, J = 276.5 Hz), 123.6, 116.9, 116.2, 62.4, 61.6, 56.8, 44.3, 42.5, 39.6, 37.1, 34.4, 33.9 (q, J = 28.8 Hz), 27.3, 26.1, 17.6 (q, J = 3.0 Hz), 14.0, 14.0; ^{19}F NMR (471 MHz, $CDCl_3$) δ -66.29 (t, J = 10.6 Hz); HRMS (ESI) m/z $[M + H]^+$ calcd for $C_{28}H_{35}F_3$ N_2O_4Na 543.2441, found 543.2449.

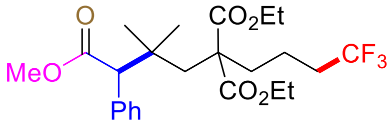

19

4,4-Diethyl-1-methyl 8,8,8-trifluoro-2,2-dimethyl-1-phenyloctane-1,4,4-tricarboxylate (**19**): Yield 81% (76.8 mg); yellow oil; ^{1}H NMR (500 MHz, $CDCl_3$) δ 7.41 – 7.26 (m, 5H), 4.20 – 4.07 (m, 4H), 3.64 (s, 3H), 3.44 (s, 1H), 2.35 (d, J = 15.1 Hz, 1H), 2.10 – 2.00 (m, 5H), 1.44 – 1.37 (m, 2H), 1.23 – 1.17 (m, 6H), 1.03 (s, 3H), 0.97 (s, 3H); ^{13}C NMR (126 MHz, $CDCl_3$) δ 173.2, 171.8, 171.8, 135.2, 130.5, 128.1, 127.6, 127.0 (q, J = 279.4 Hz), 63.5, 61.4, 56.8, 51.6, 40.7, 37.7, 34.0 (q, J = 28.8 Hz), 33.8, 24.6, 24.5, 17.6 (q, J = 3.0 Hz), 14.0, 14.0; ^{19}F NMR (471 MHz, $CDCl_3$) δ -66.30 (t, J = 10.7 Hz); HRMS (ESI) m/z $[M + H]^+$ calcd for $C_{24}H_{33}F_3O_6Na$ 497.2121, found 497.2125.

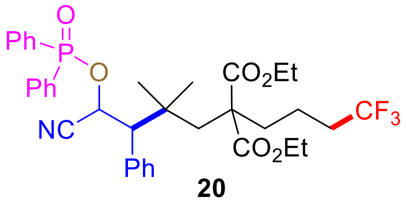

20

Diethyl 2-(4-cyano-4-((diphenylphosphoryl)oxy)-2,2-dimethyl-3-phenylbutyl)-2-(4,4,4-trifluorobutyl)malonate (**20**): Yield 56% (75.2 mg); (dr = 1 : 0.4); yellow oil; ^{1}H NMR (500 MHz, $CDCl_3$) δ 7.83 – 7.33 (m, 21H), 5.79 (dd, J = 8.9, 4.7 Hz, 0.4H), 5.68 (dd, J = 9.5, 5.6 Hz, 1H), 4.15 – 4.05 (m, 5.6H), 3.05 (d, J = 5.6 Hz, 1H), 2.89 (d, J = 4.6 Hz, 0.4H), 2.25 (m, 1.4H), 2.07 – 1.85 (m, 7H), 1.38 – 1.30 (m, 2.8H), 1.20 – 1.14 (m, 8.4H), 1.07 – 1.01 (m, 8.4H); ^{13}C NMR (126 MHz, $CDCl_3$) δ 171.5, 171.4, 171.4, 136.3, 136.0, 133.2, 133.2, 133.1, 133.1, 132.8, 132.8, 132.3, 132.2, 132.1, 132.0, 131.4, 131.3, 131.3, 131.1, 131.1, 130.7, 130.3, 130.2, 130.0, 130.0, 129.2, 128.9, 128.9, 128.8, 128.8, 128.7, 128.7, 128.6, 128.5, 128.4, 128.0, 126.9 (q, J = 276.6 Hz), 117.4, 117.3, 117.0, 62.7, 62.6, 62.6, 62.2, 62.2, 61.6, 61.6, 61.5, 61.5, 61.4, 56.7, 41.9, 41.7, 37.9, 37.4, 34.2, 33.9 (q, J = 28.8 Hz), 29.8, 26.3, 25.8, 25.7, 25.6, 17.6 (q, J = 3.0 Hz), 14.0, 13.9; ^{19}F NMR (471 MHz, $CDCl_3$) -65.67 – -66.06 (m), ^{31}P NMR (202 MHz, $CDCl_3$) δ 36.61 – 35.63 (m); HRMS (ESI) m/z $[M + H]^+$ calcd for $C_{36}H_{42}F_3NO_6P$ 672.2696, found 672.2706.

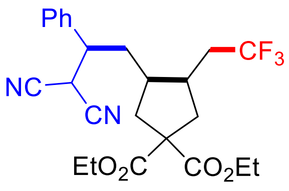

22

Diethyl-3-(3,3-dicyano-2-phenylpropyl)-4-(2,2,2-trifluoroethyl) cyclopentane-1,1-dicarboxylate (**22**): Yield 56% (39.0 mg); (dr = 1 : 1); R_f = 0.4 (ethyl acetate/n-hexane, 2 : 8); colorless oil; ^{1}H NMR (500 MHz, $CDCl_3$) δ 7.45 – 7.29 (m, 10H), 4.27 – 4.10 (m, 8H), 3.90 (t, *J* = 6.1 Hz, 2H), 3.44 (m, 1H), 3.24 (m, 1H), 2.62 (dd, *J* = 13.9, 7.1 Hz, 1H), 2.41 – 1.89 (m, 19H), 1.28 – 1.19 (m, 12H); ^{13}C NMR (126 MHz, $CDCl_3$) δ 172.6, 172.4, 172.1, 172.0, 136.7, 135.5, 129.7, 129.7, 129.6, 129.4, 129.4, 128.1, 127.8, 127.0 (q, *J* = 277.1 Hz), 126.7 (q, *J* = 277.1 Hz), 111.8, 111.8, 111.7, 62.2, 62.0, 61.9, 58.3, 58.2, 45.2, 44.5, 40.1, 38.7, 38.4, 38.2, 38.1, 37.1, 36.9, 35.9, 33.78 (q, *J* = 28.6 Hz), 32.78 (q, *J* = 28.1 Hz), 31.7, 30.8, 30.1, 29.8, 14.1, 14.1, 14.1, 14.1; ^{19}F NMR (471 MHz, $CDCl_3$) δ -64.15 (t, *J* = 10.6 Hz), -64.57 (t, *J* = 10.6 Hz).

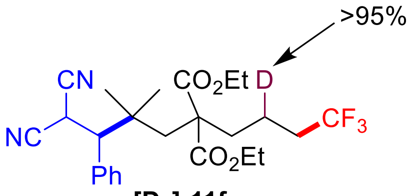

[D$_2$]-11f

Diethyl-2-(4,4-dicyano-2,2-dimethyl-3-phenylbutyl)-2-(4,4,4-trifluorobutyl-2-d)malonate (**[D$_2$]-10f**): Yield 34% (24.6 mg); R_f = 0.4 (ethyl acetate/n-hexane, 2 : 8); yellow oil; ^{1}H NMR (500 MHz, $CDCl_3$) δ 7.57 – 7.30 (m, 5H), 4.45 (d, *J* = 4.9 Hz, 1H), 4.25 – 4.09 (m, 4H), 3.06 (d, *J* = 4.9 Hz, 1H), 2.28 (d, *J* = 14.9 Hz, 1H), 2.14 – 1.94 (m, 5H), 1.45 – 1.34 (m, 1H), 1.25 (dd, *J* = 7.3, 6.5 H z, 6H), 1.16 (s, 3H), 1.00 (s, 3H); ^{13}C NMR (126 MHz, $CDCl_3$) δ 171.6, 171.3, 135.5, 129.9, 129.0, 129.0, 127.9, 126.8 (d, *J* = 276.5 Hz), 113.3, 113.2, 62.0, 61.9, 57.2, 56.7, 42.5, 38.3, 34.9, 33.8 (q, *J* = 28.5 Hz), 26.0, 25.9, 25.0, 17.5 – 17.0 (m), 14.0, 14.0; ^{19}F NMR (471 MHz, $CDCl_3$) δ -66.23 (t, *J* = 10.6 Hz); HRMS (ESI) m/z [M + H]$^+$ calcd for $C_{25}H_{31}DF_3N_2O_4$: 482.2377 found : 482.2380.

12. NMR spectra of the products:

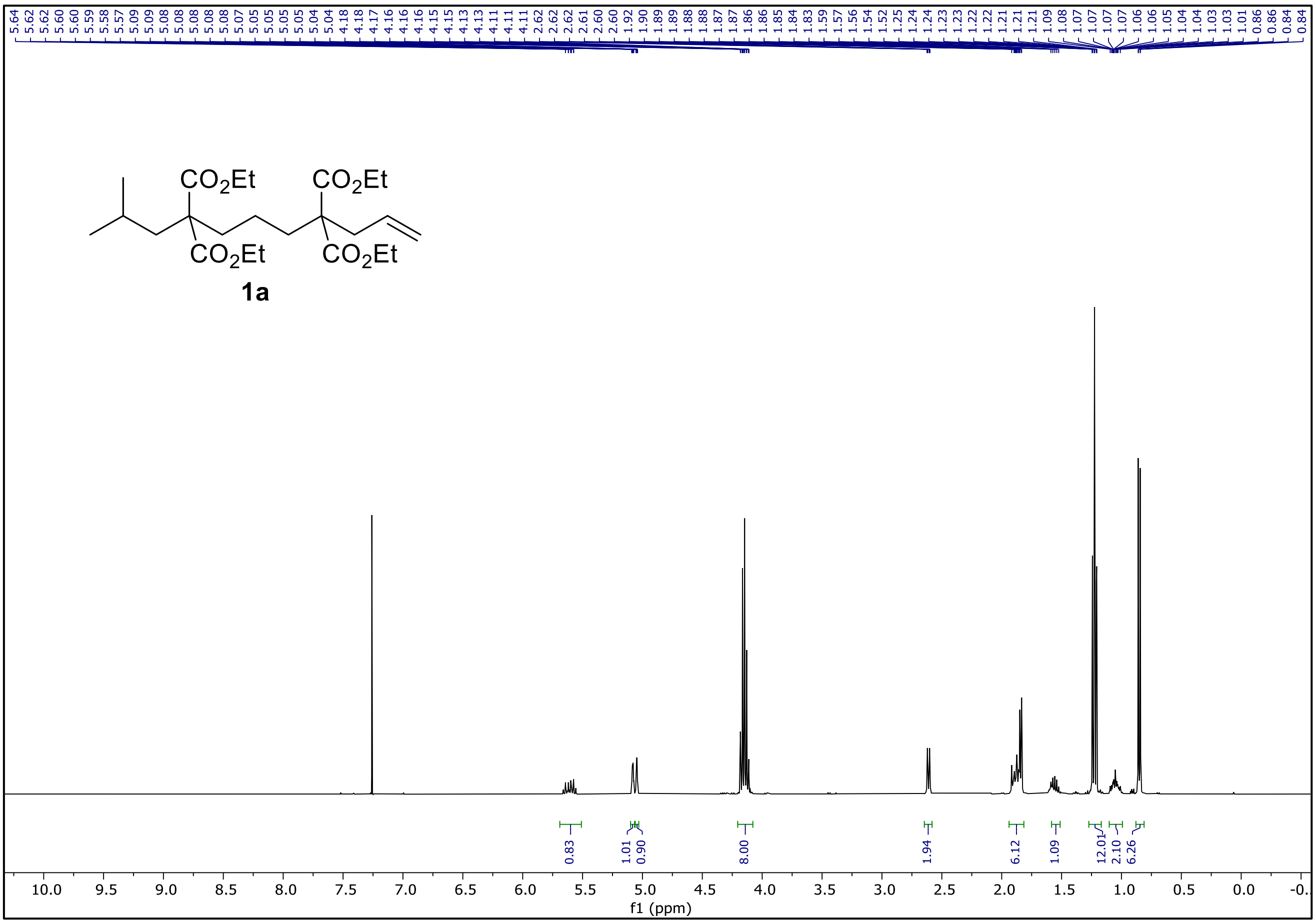

^{1}H NMR of compound **1a** (500 MHz, $CDCl_3$)

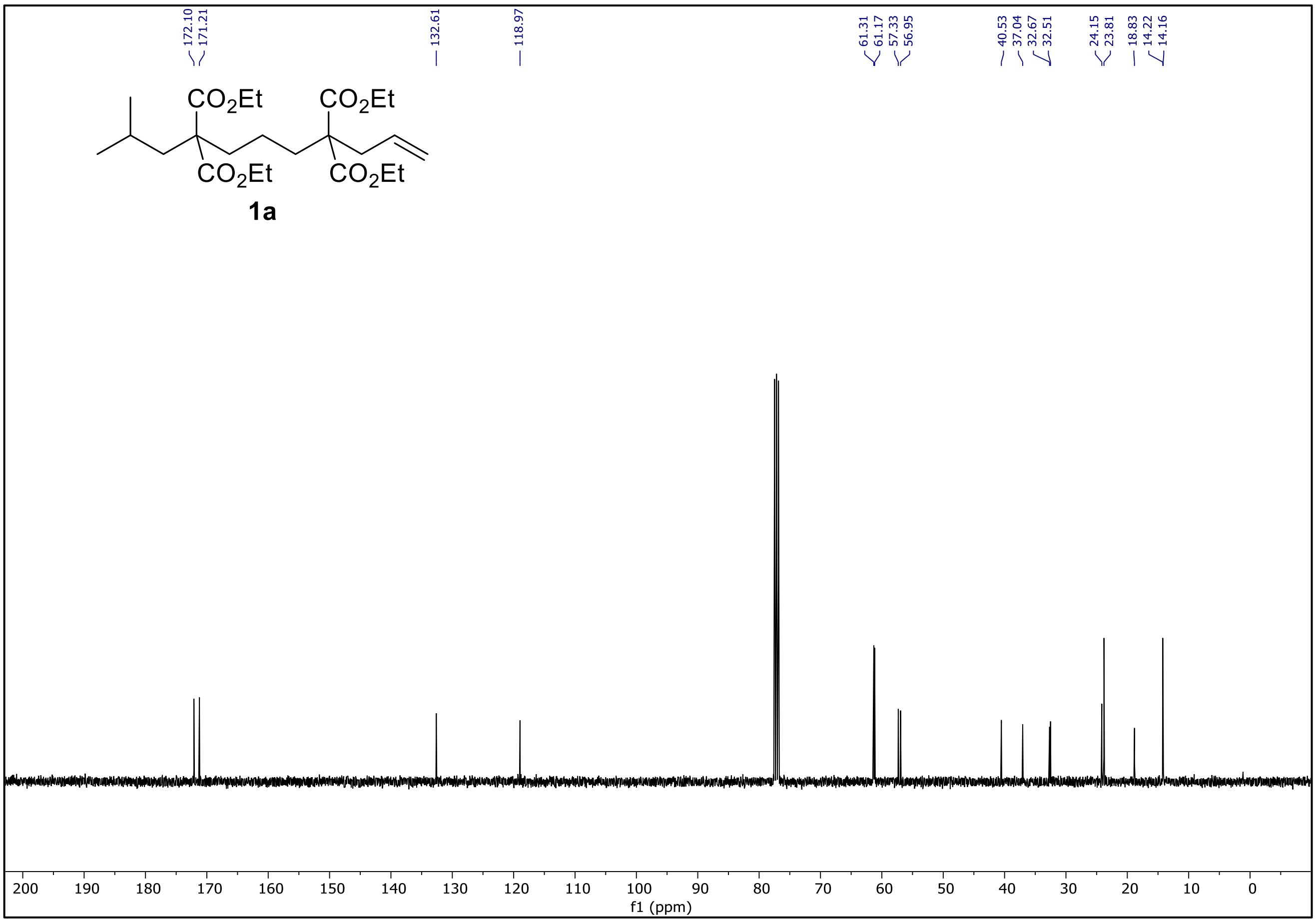

$^{13}C\{^1H\}$ NMR of compound **1a** (126 MHz, $CDCl_3$)

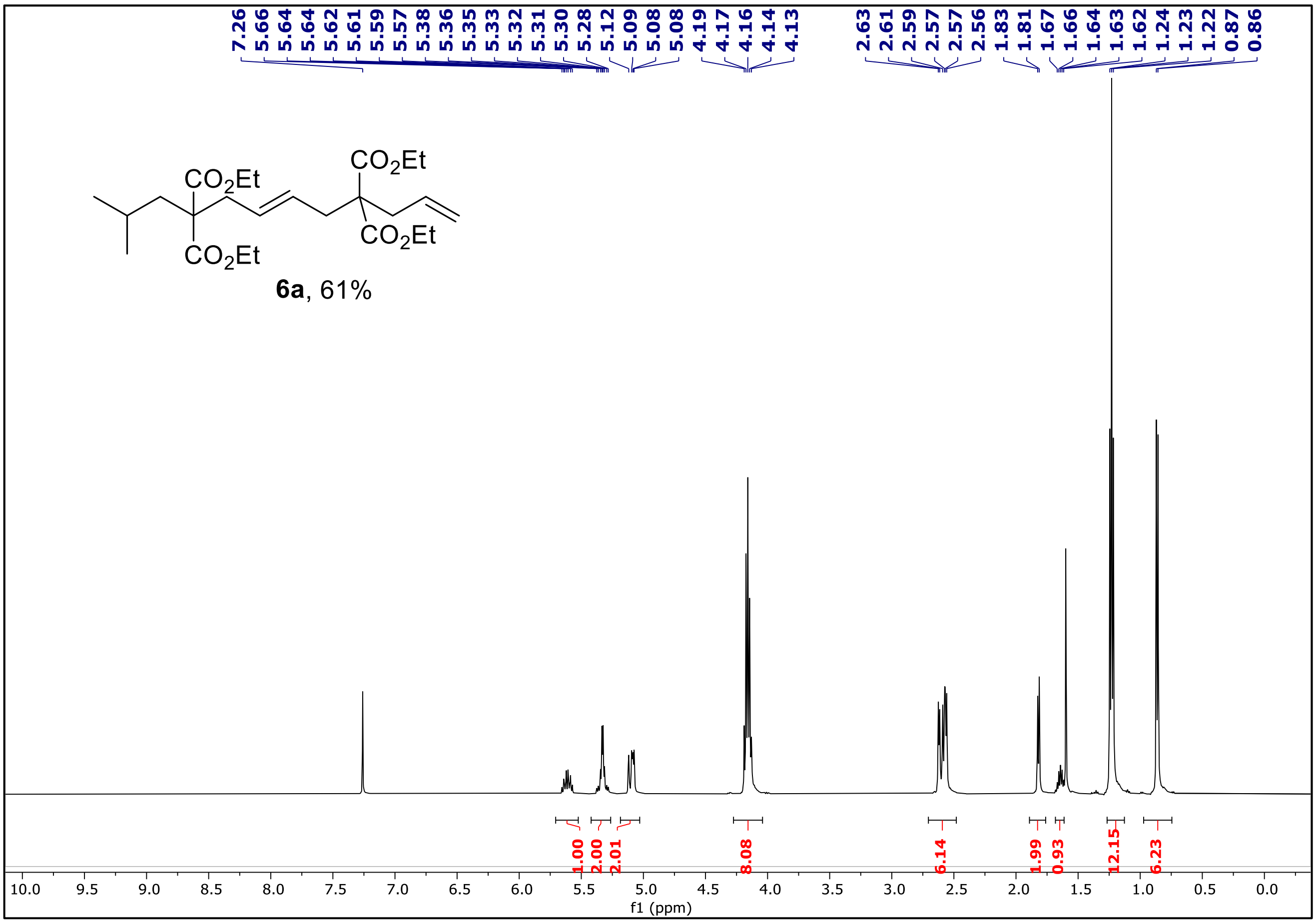

¹H NMR of compound **6a** (500 MHz, $CDCl_3$)

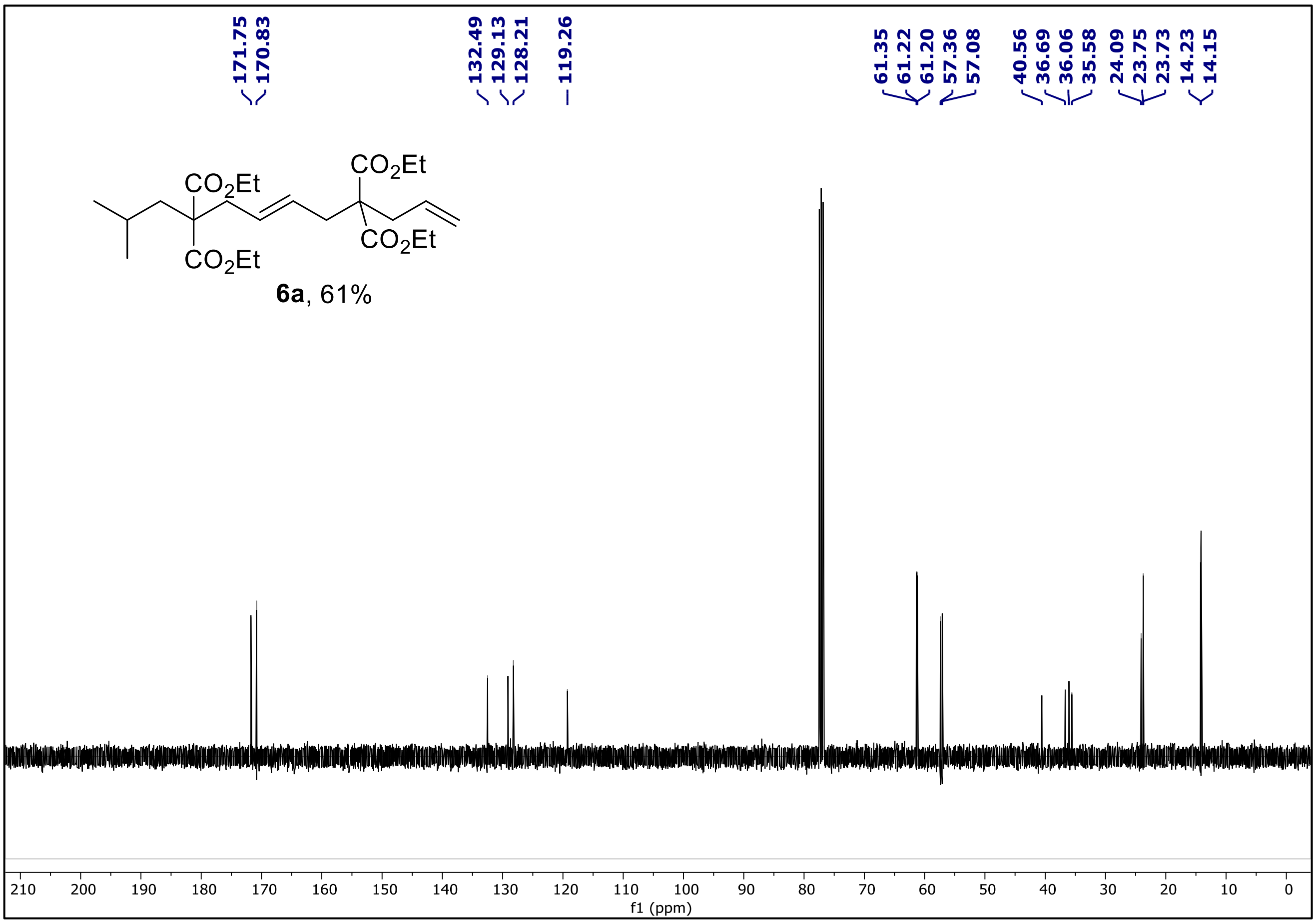

¹³C{¹H} NMR of compound **6a** (101 MHz, $CDCl_3$)

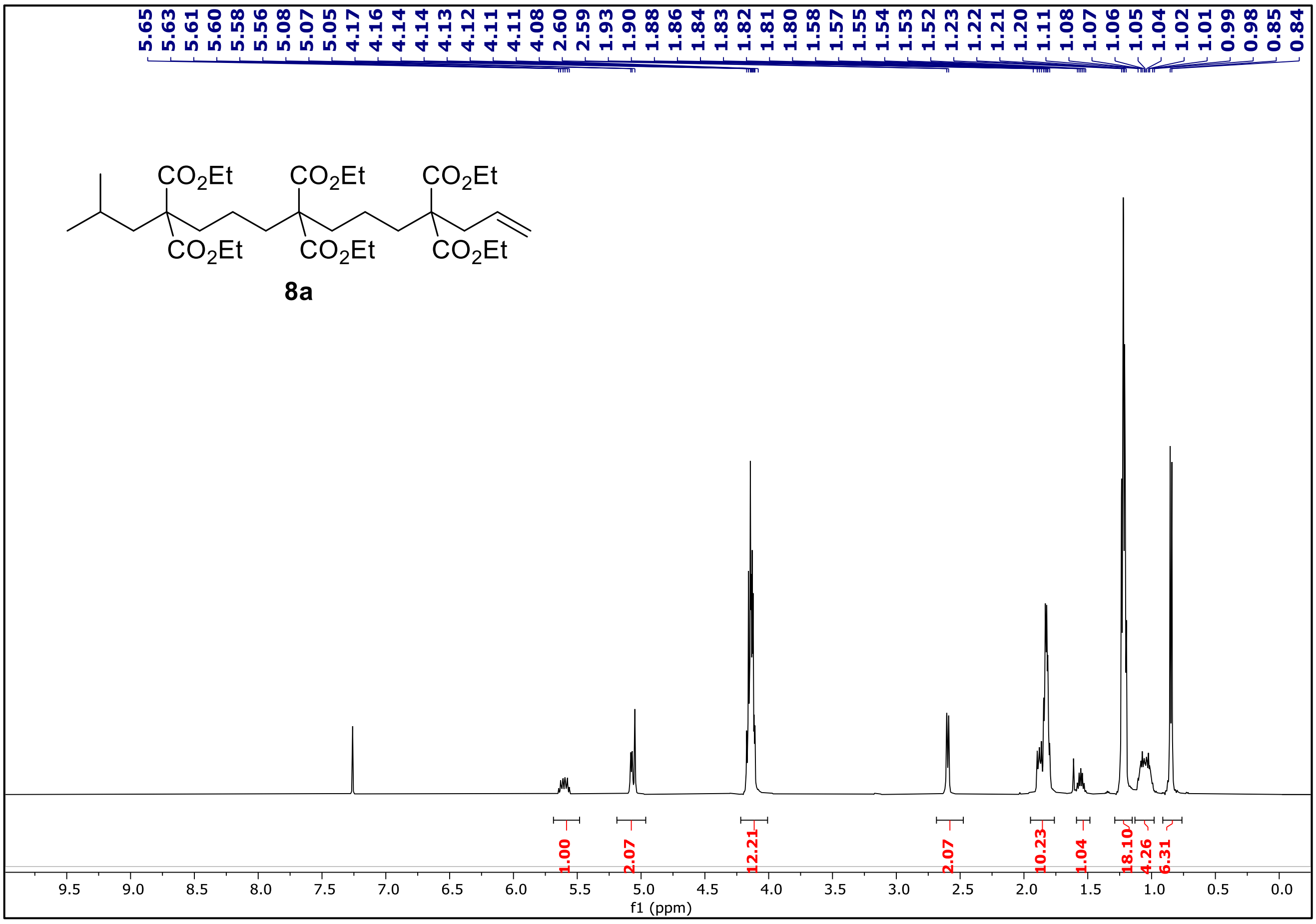

^{1}H NMR of compound **8a** (500 MHz, $CDCl_3$)

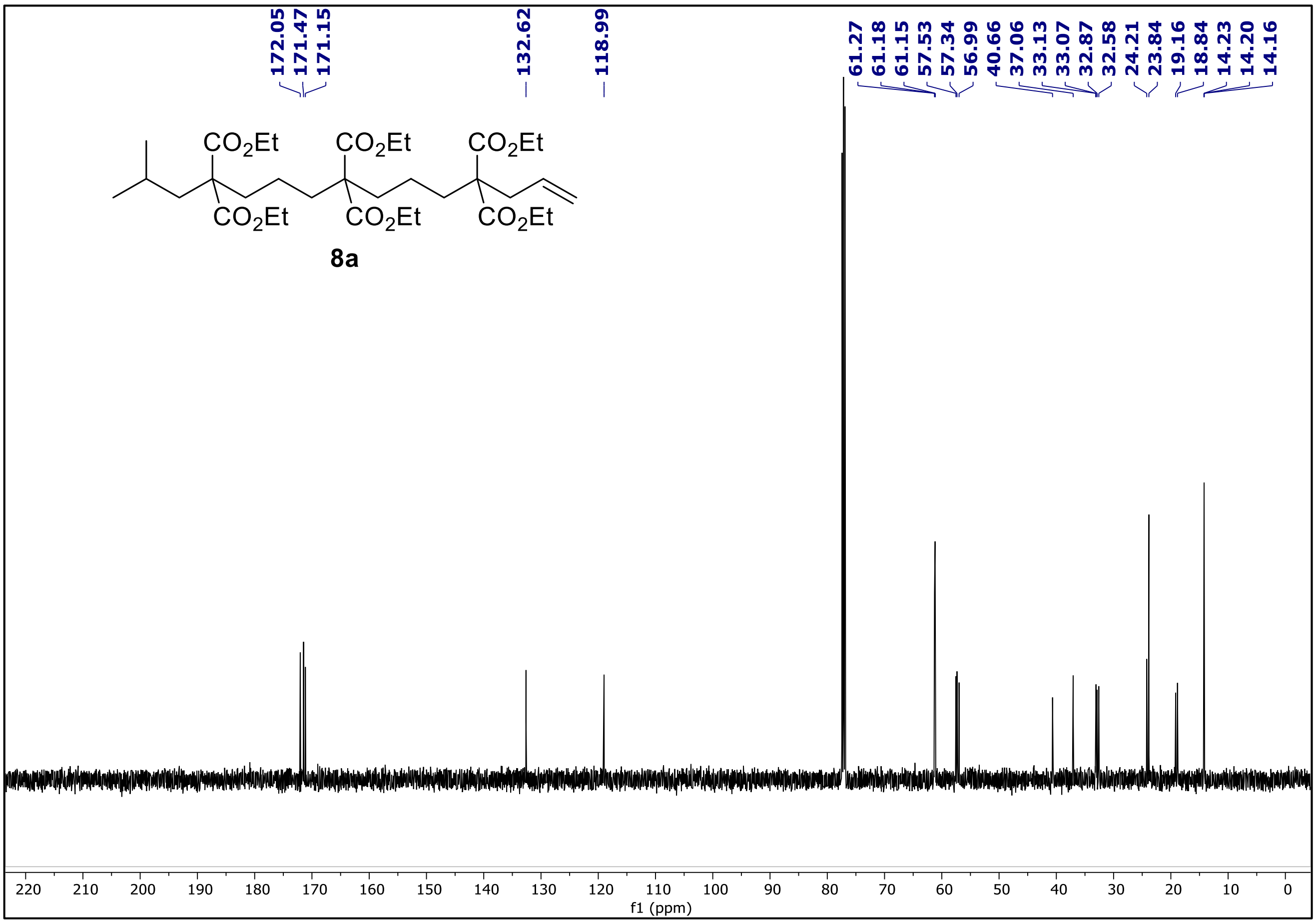

$^{13}C\{^1H\}$ NMR of compound **8a** (126 MHz, $CDCl_3$)

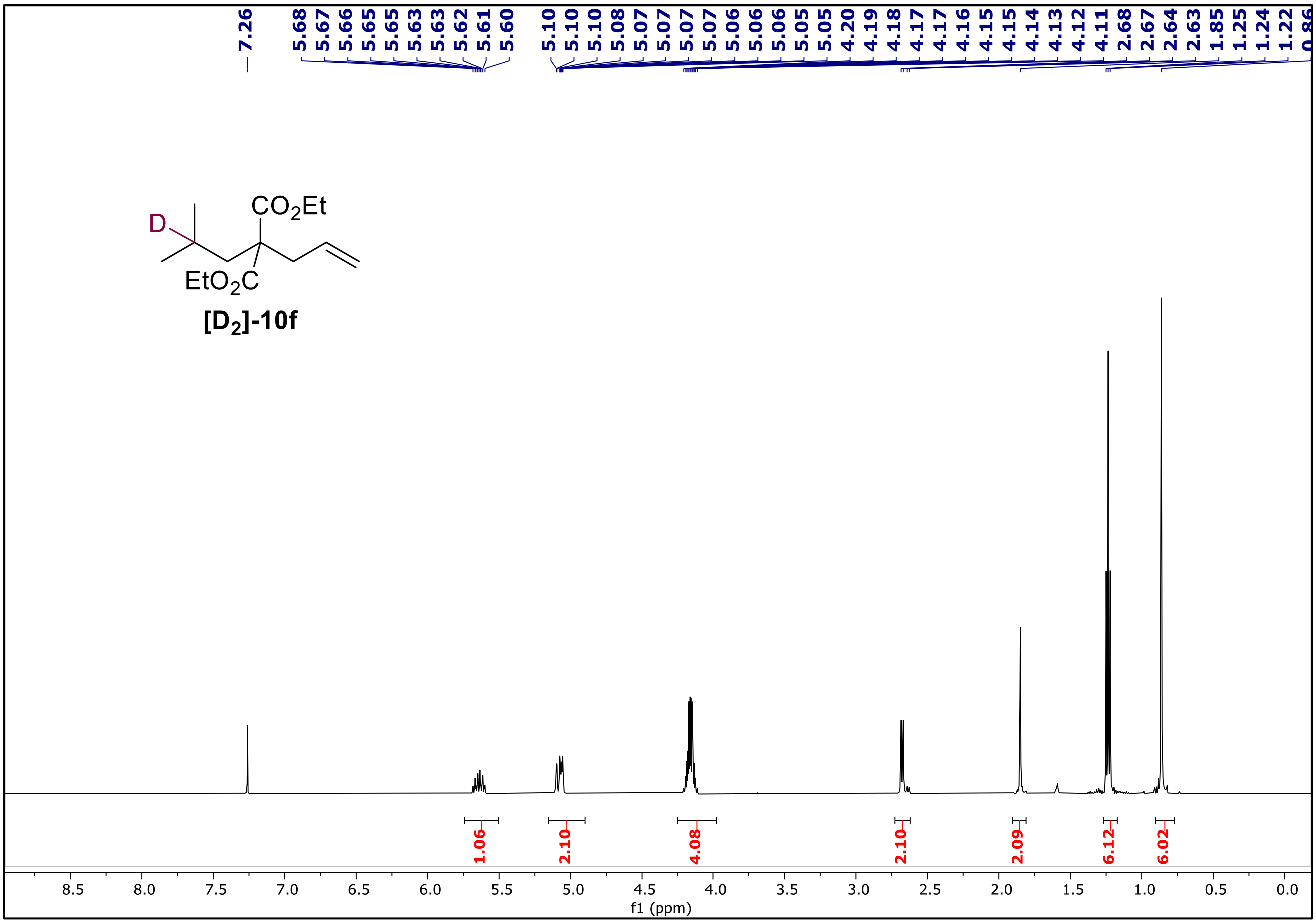

^{1}H NMR of compound **[D$_2$]-10f** (500 MHz, CDCl$_3$)

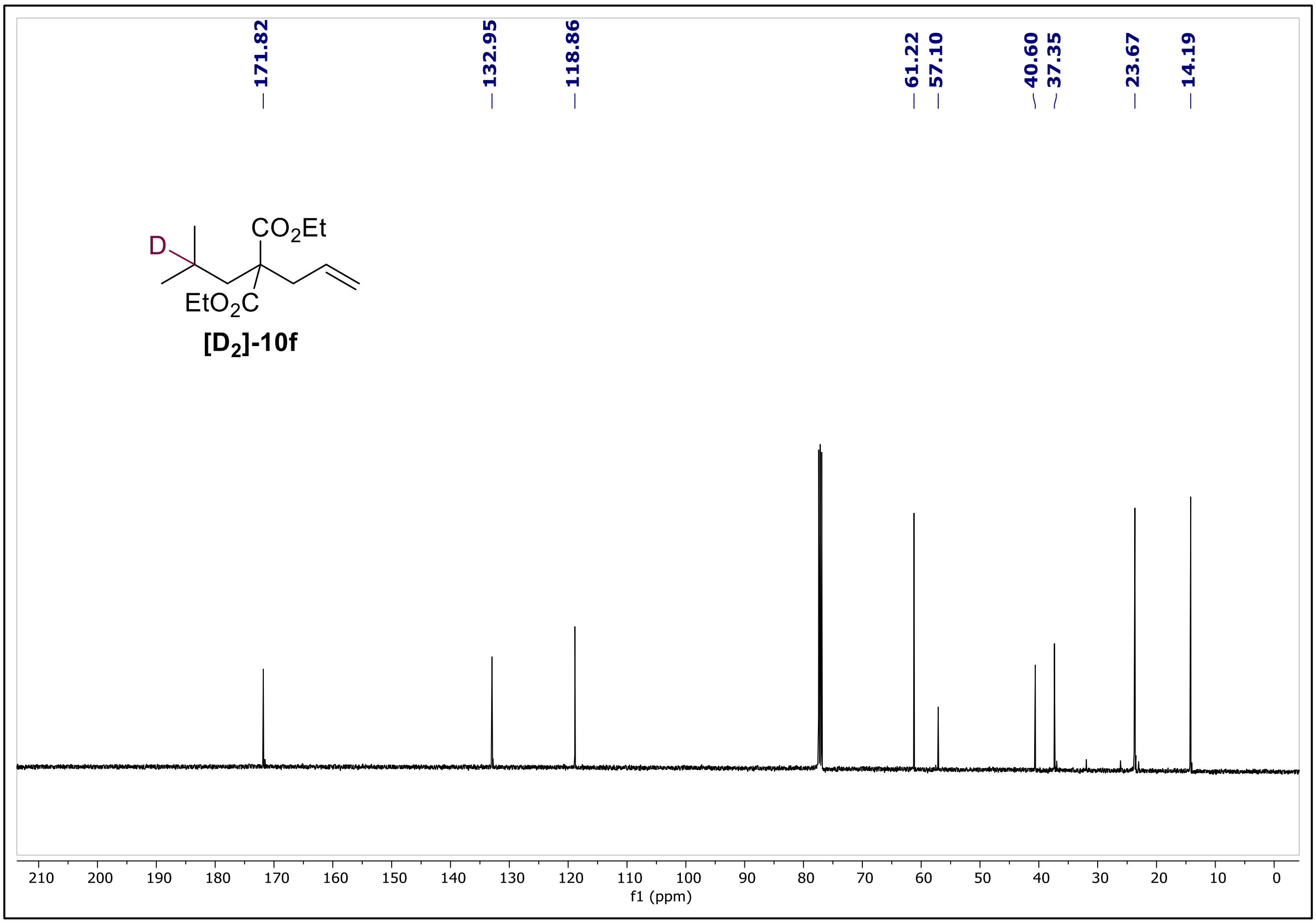

^{13}C{^{1}H} NMR of compound **[D$_2$]-10f** (126 MHz, CDCl$_3$)

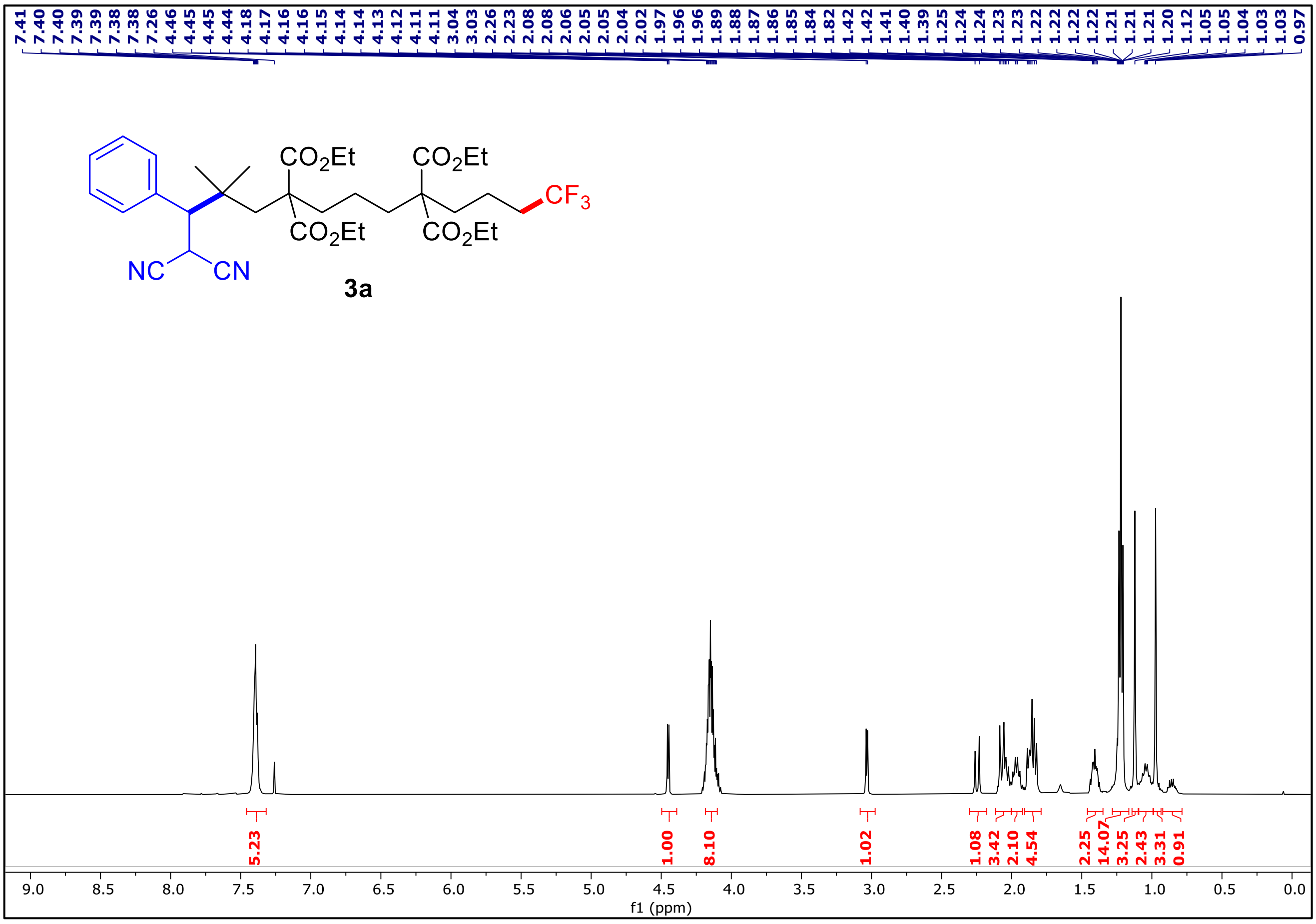

^{1}H NMR of compound **3a** (500 MHz, $CDCl_3$)

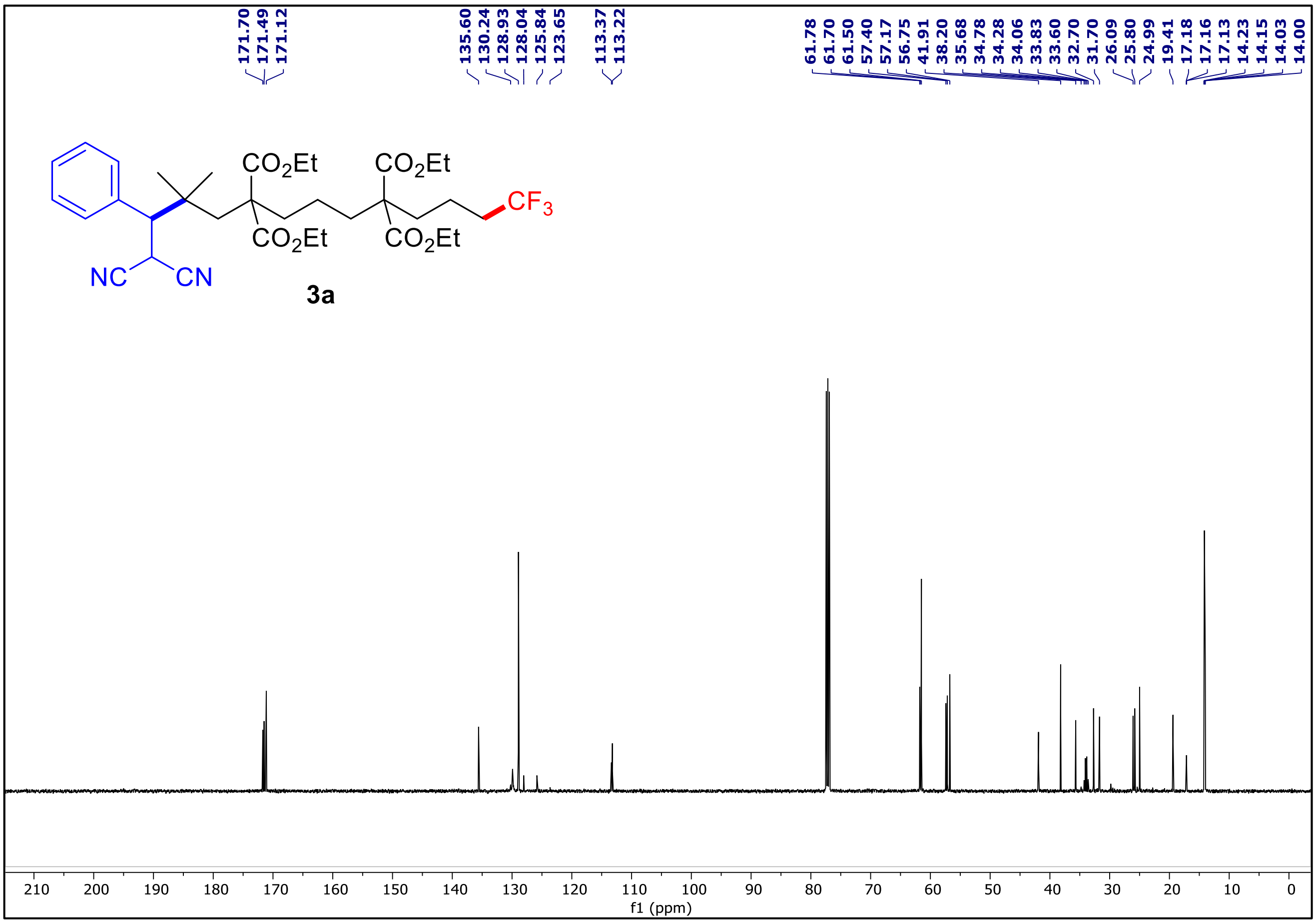

$^{13}C\{^{1}H\}$ NMR of compound **3a** (126 MHz, $CDCl_3$)

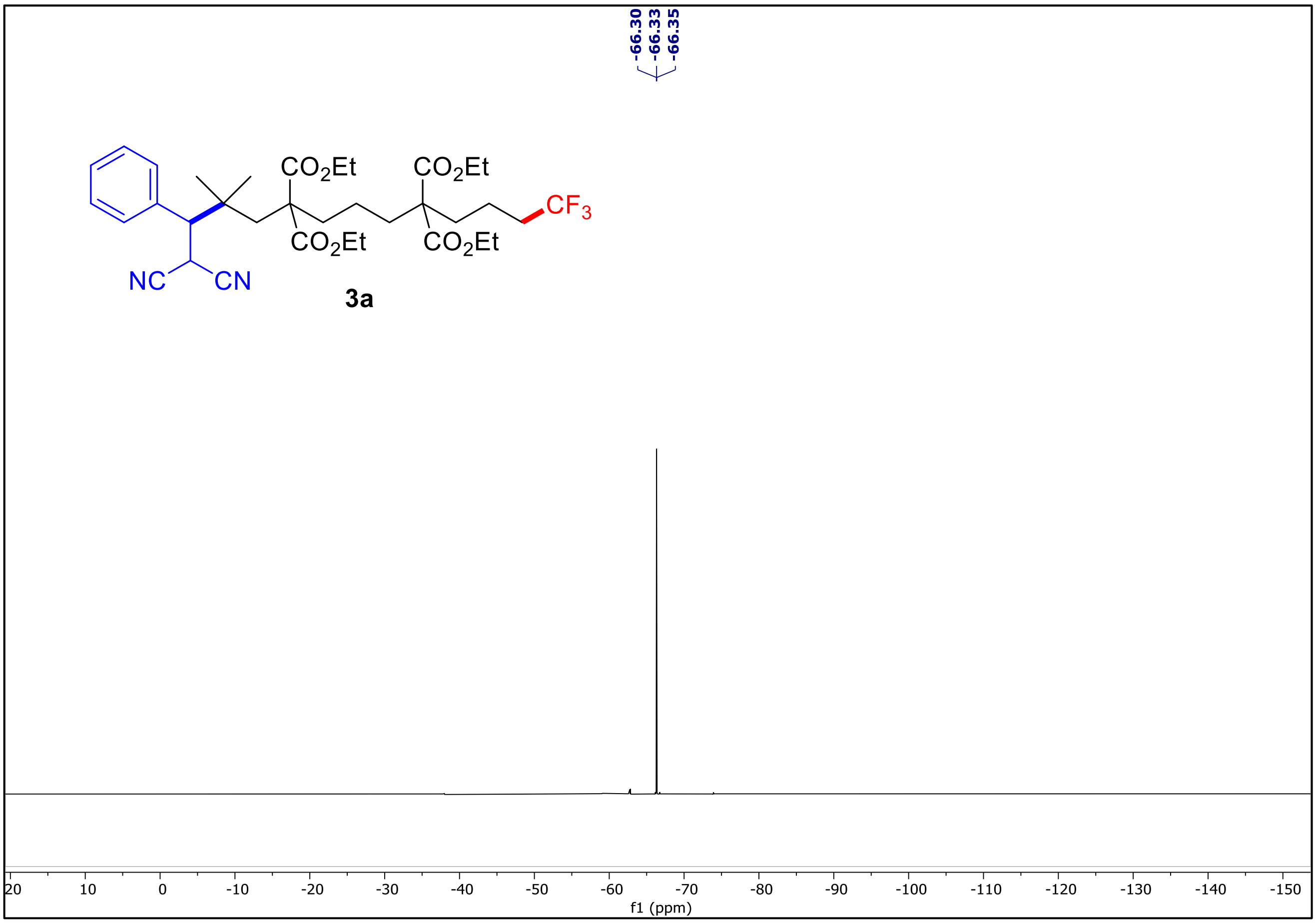

^{19}F NMR of compound **3a** (471 MHz, $CDCl_3$)

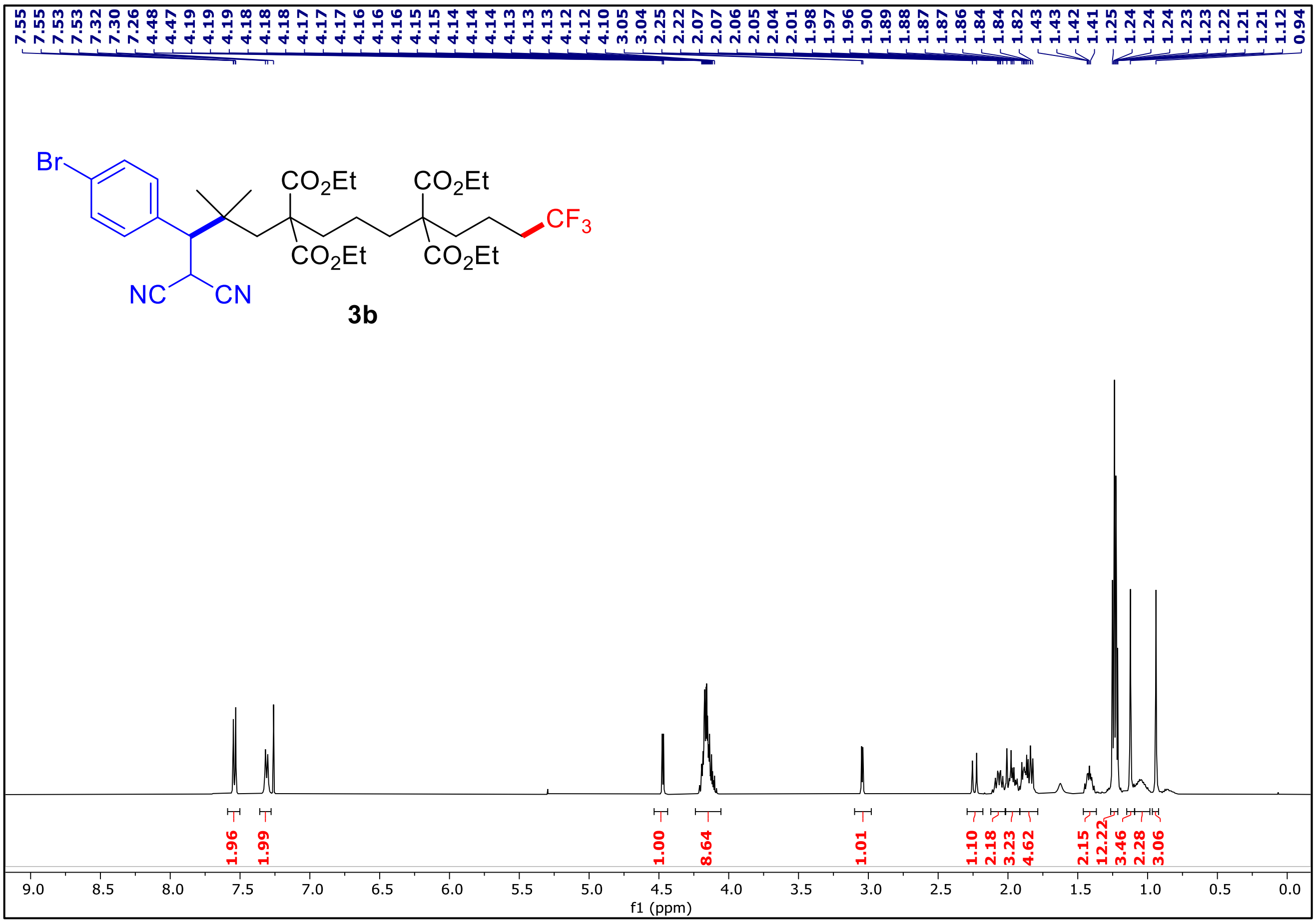

^{1}H NMR of compound **3b** (500 MHz, $CDCl_3$)

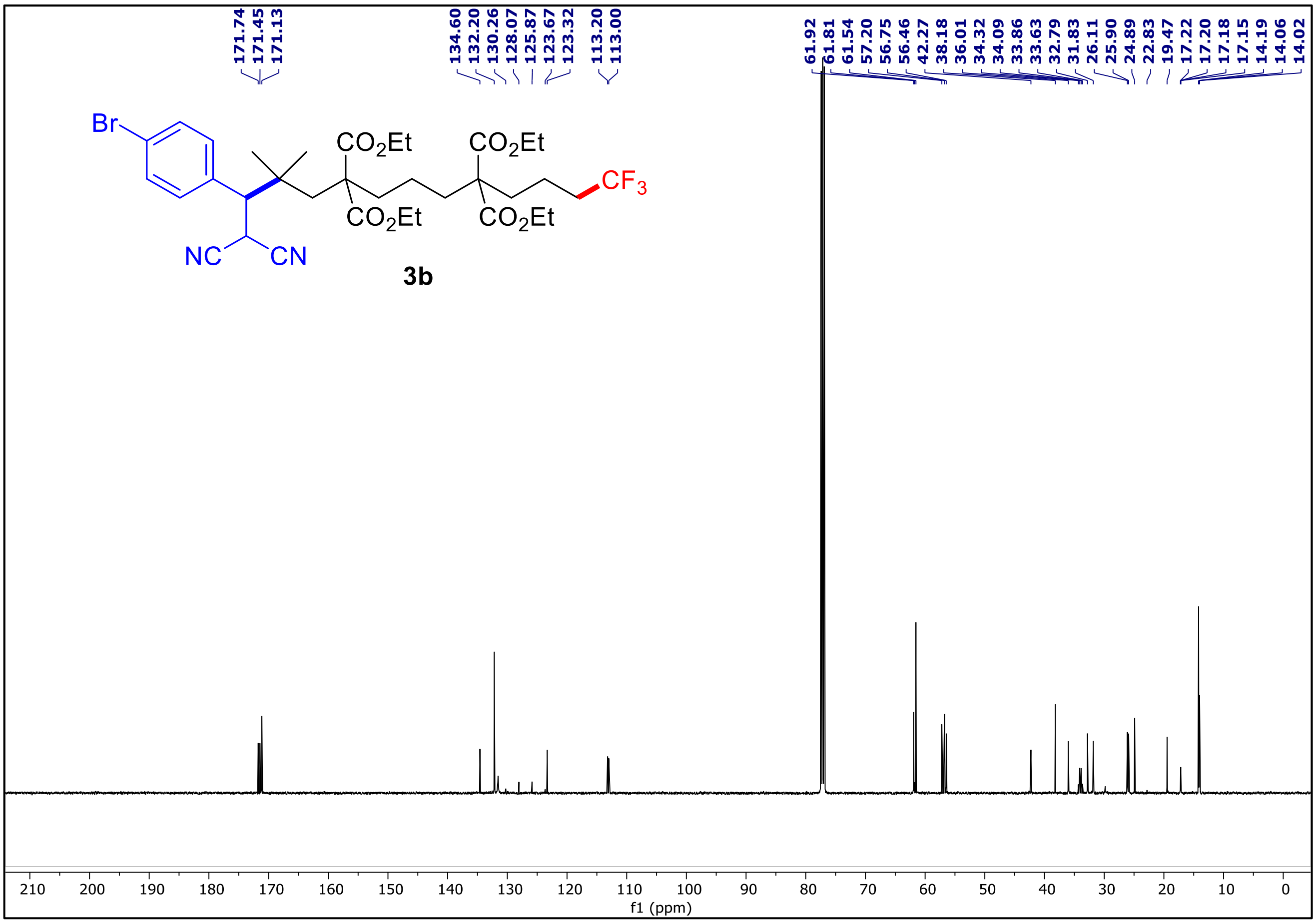

$^{13}C\{^{1}H\}$ NMR of compound **3b** (126 MHz, $CDCl_3$)

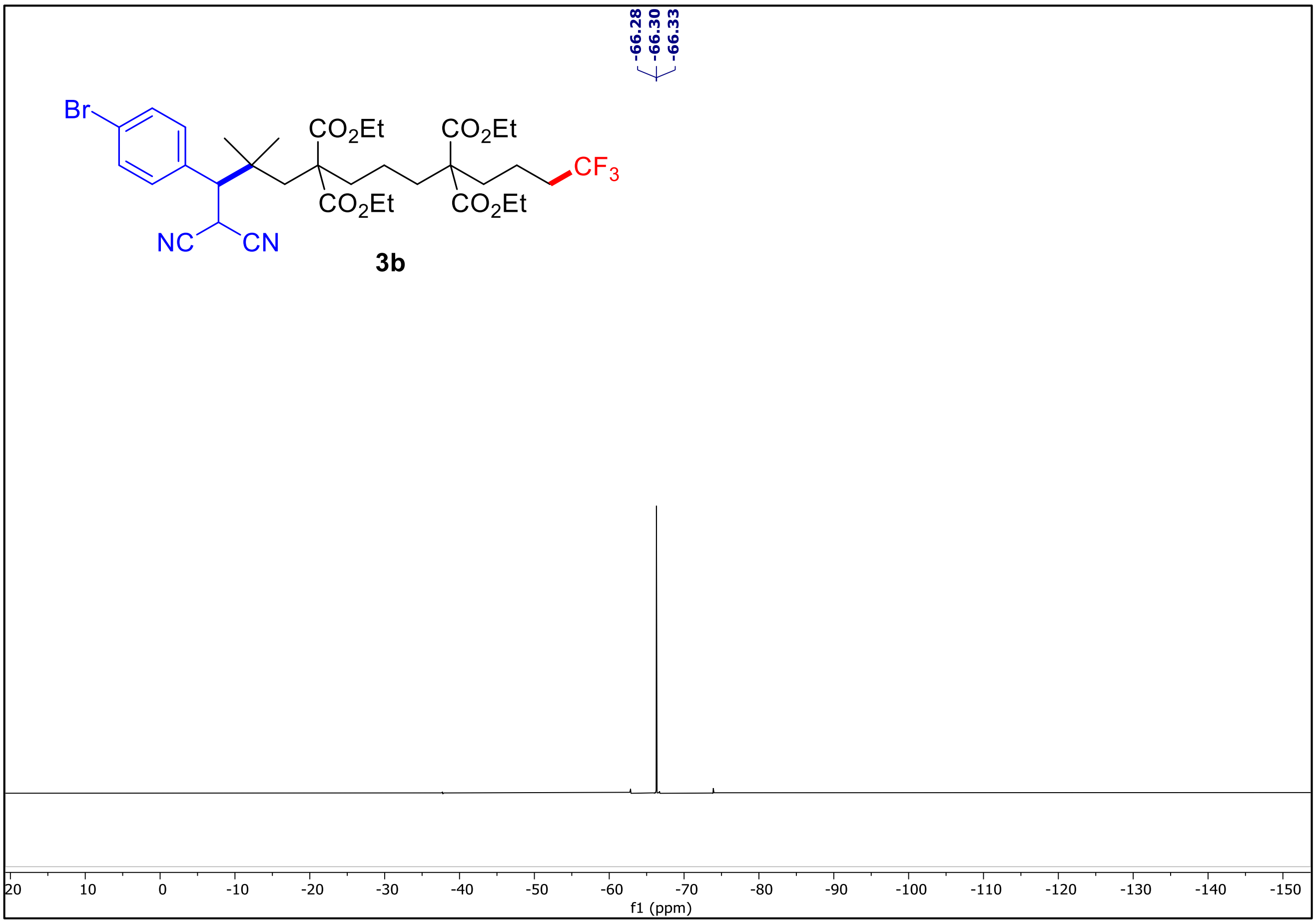

^{19}F NMR of compound **3b** (471 MHz, $CDCl_3$)

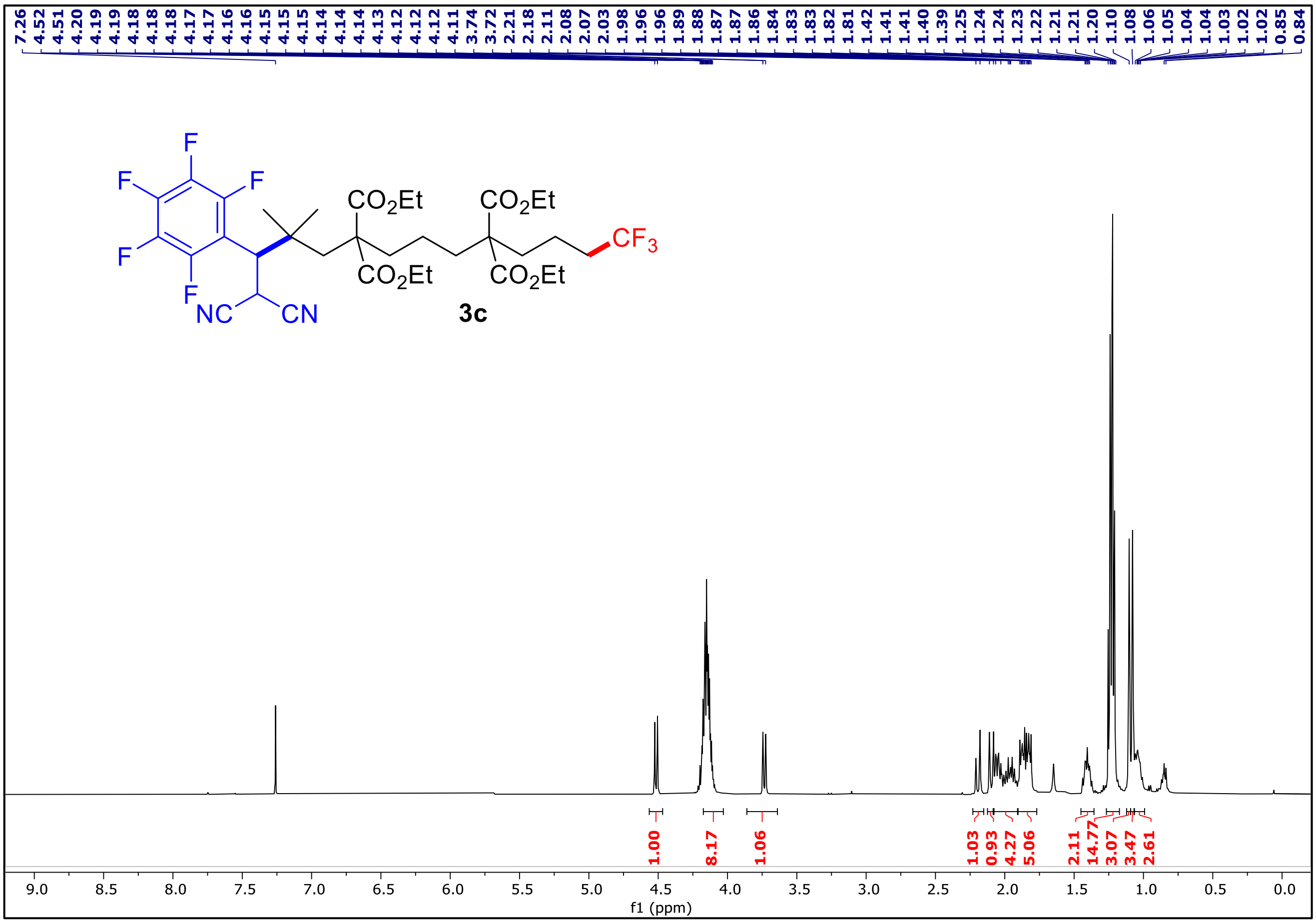

^{1}H NMR of compound **3c** (500 MHz, CDCl$_3$)

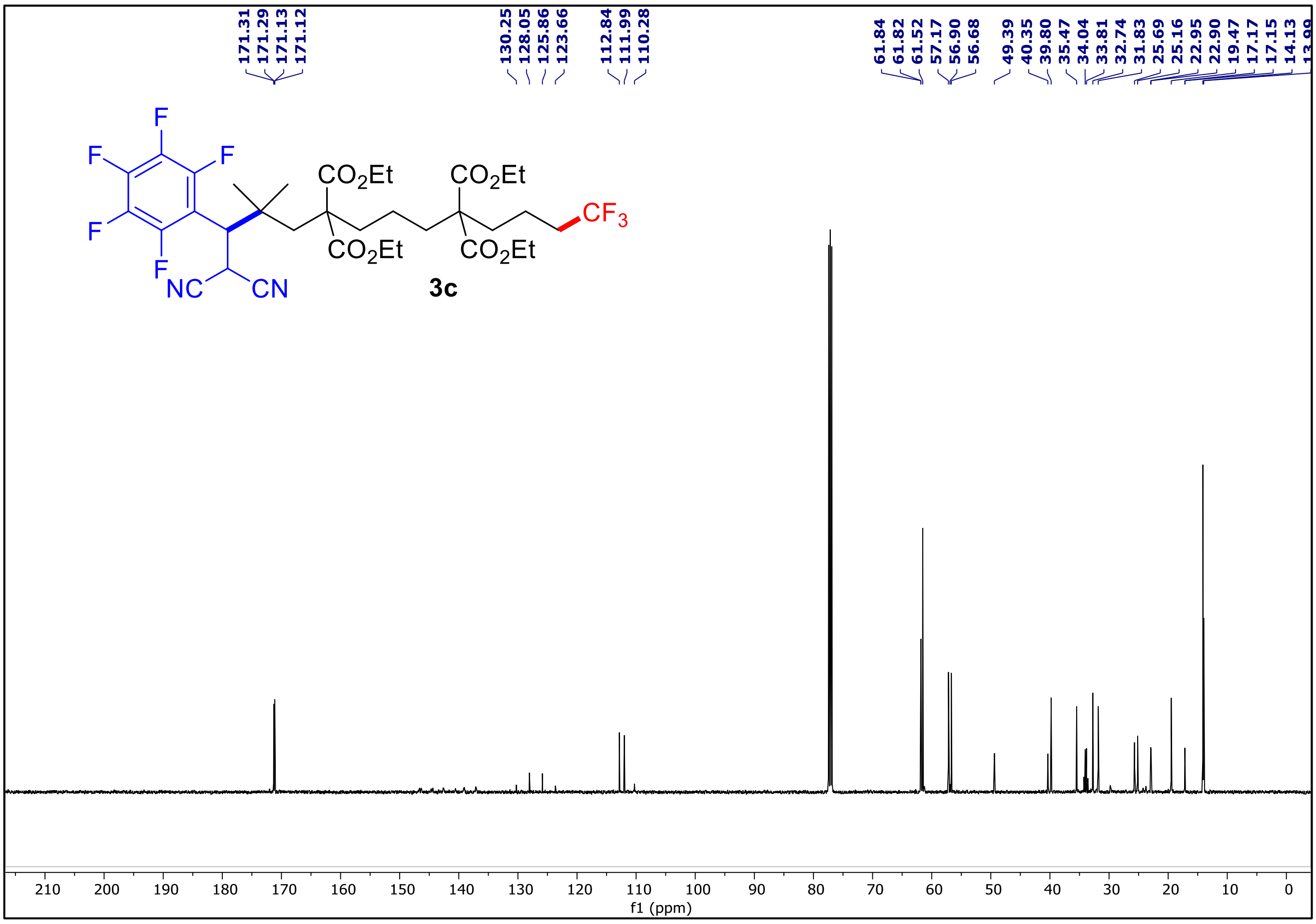

^{13}C{^{1}H} NMR of compound **3c** (126 MHz, CDCl$_3$)

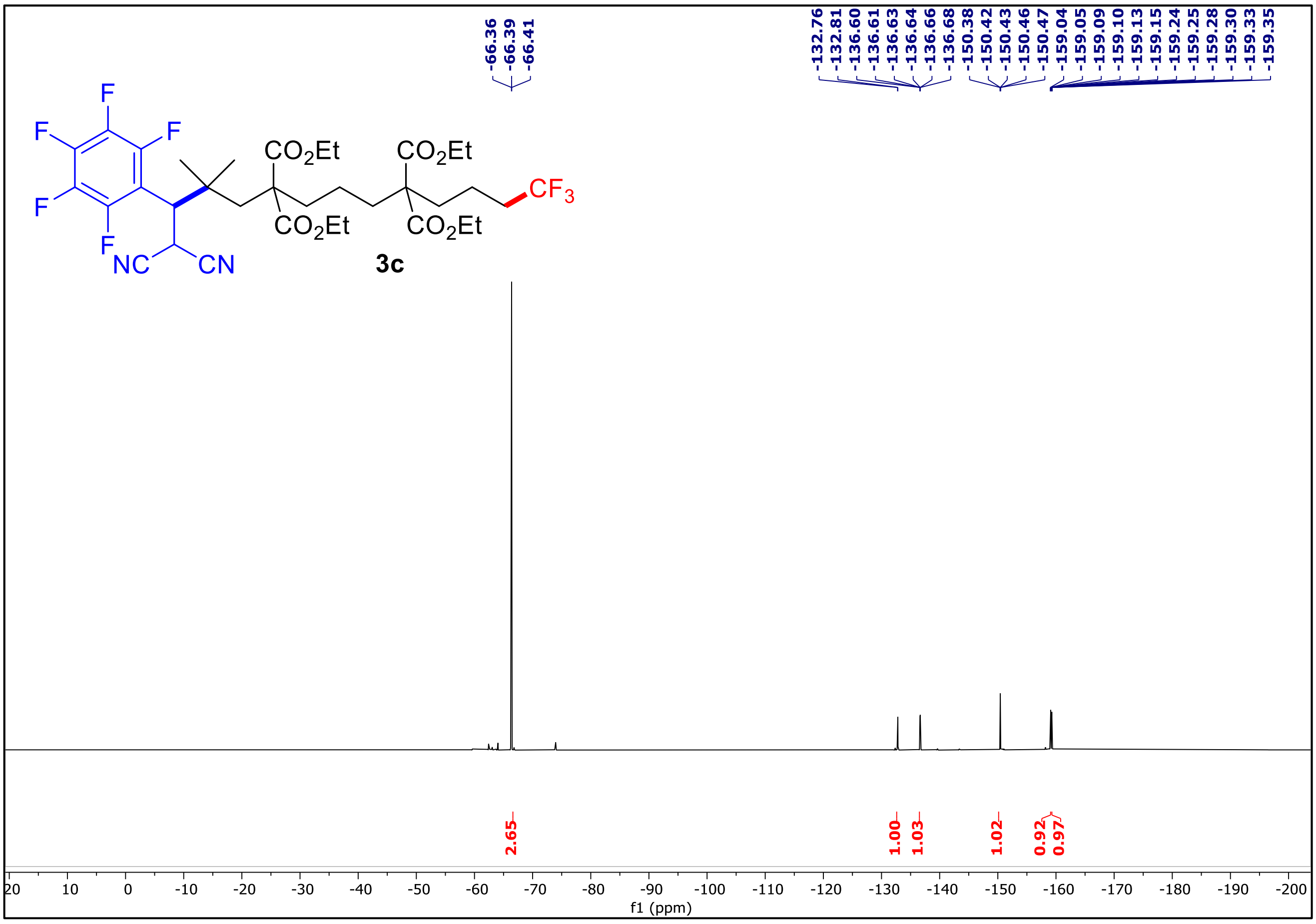

^{19}F NMR of compound **3c** (471 MHz, $CDCl_3$)

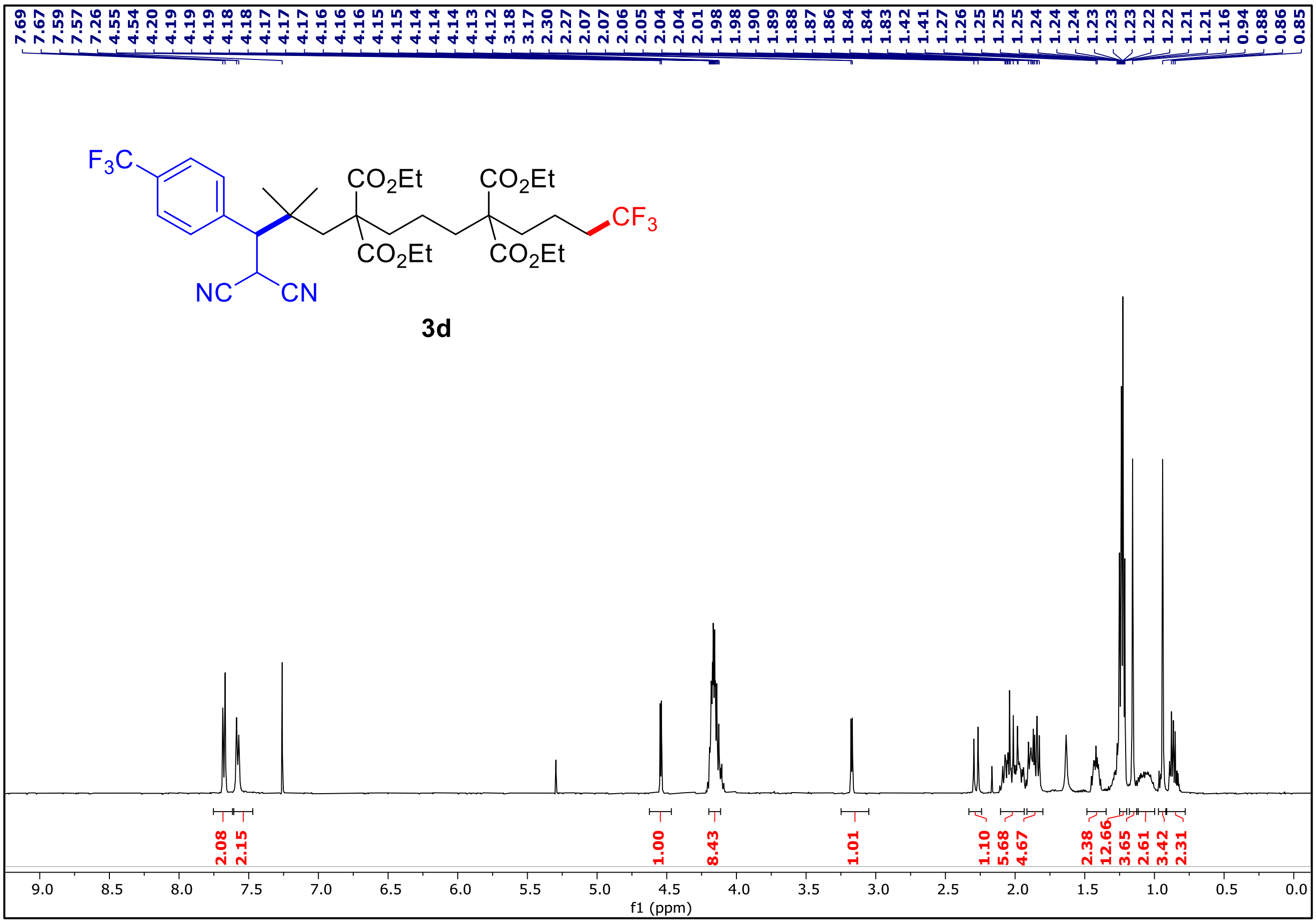

^{1}H NMR of compound **3d** (500 MHz, $CDCl_3$)

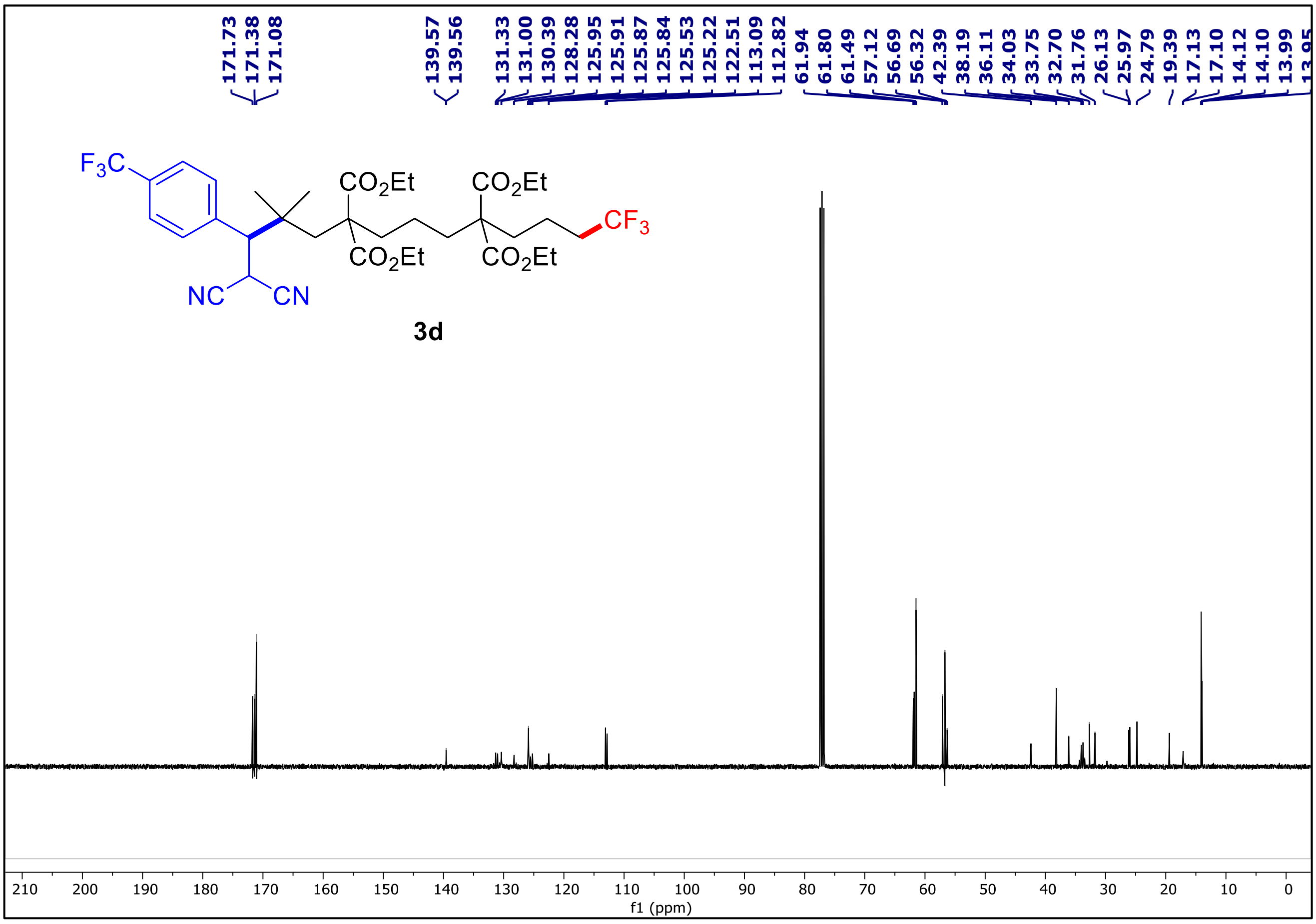

$^{13}C\{^{1}H\}$ NMR of compound **3d** (101 MHz, $CDCl_3$)

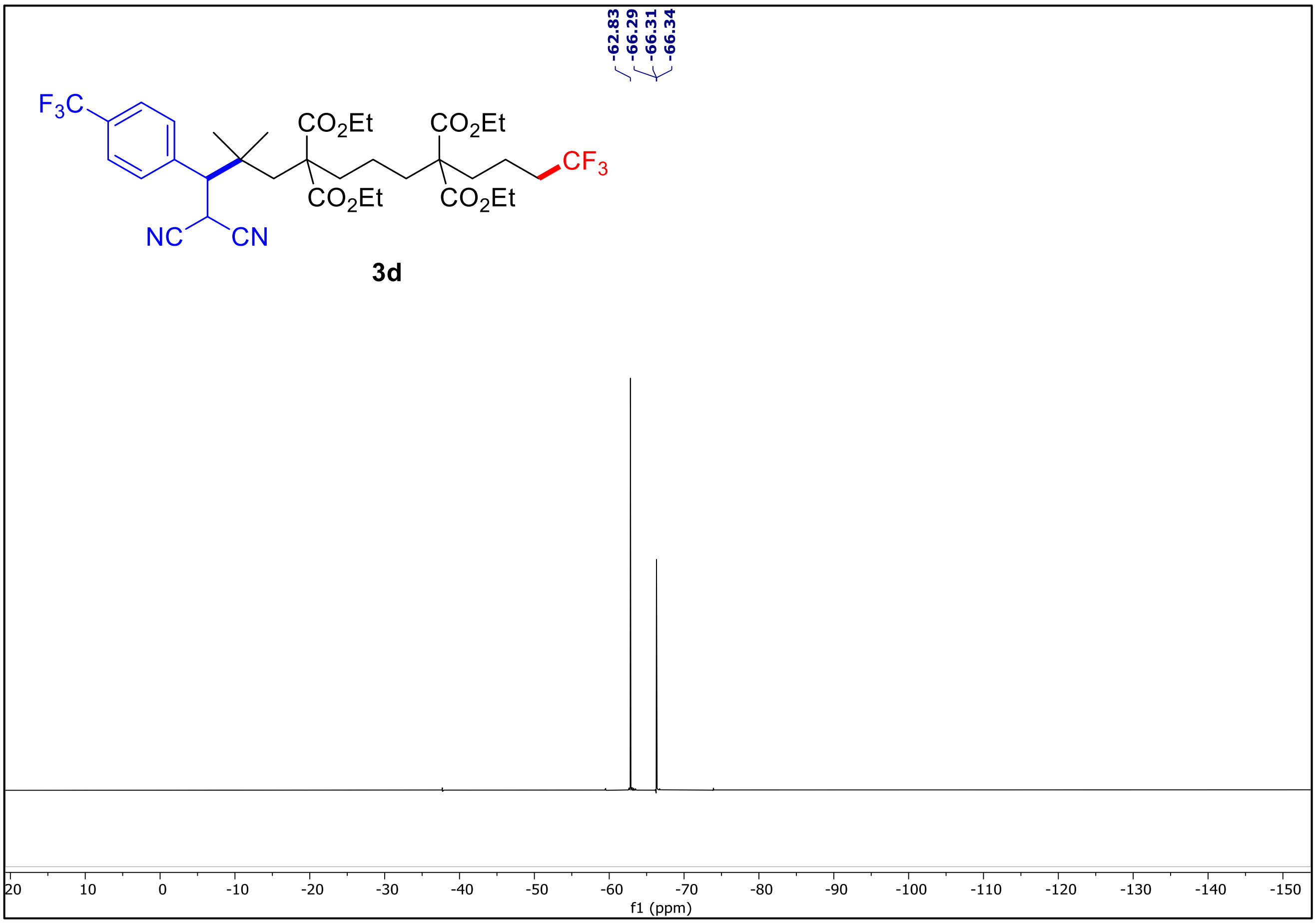

^{19}F NMR of compound **3d** (471 MHz, $CDCl_3$)

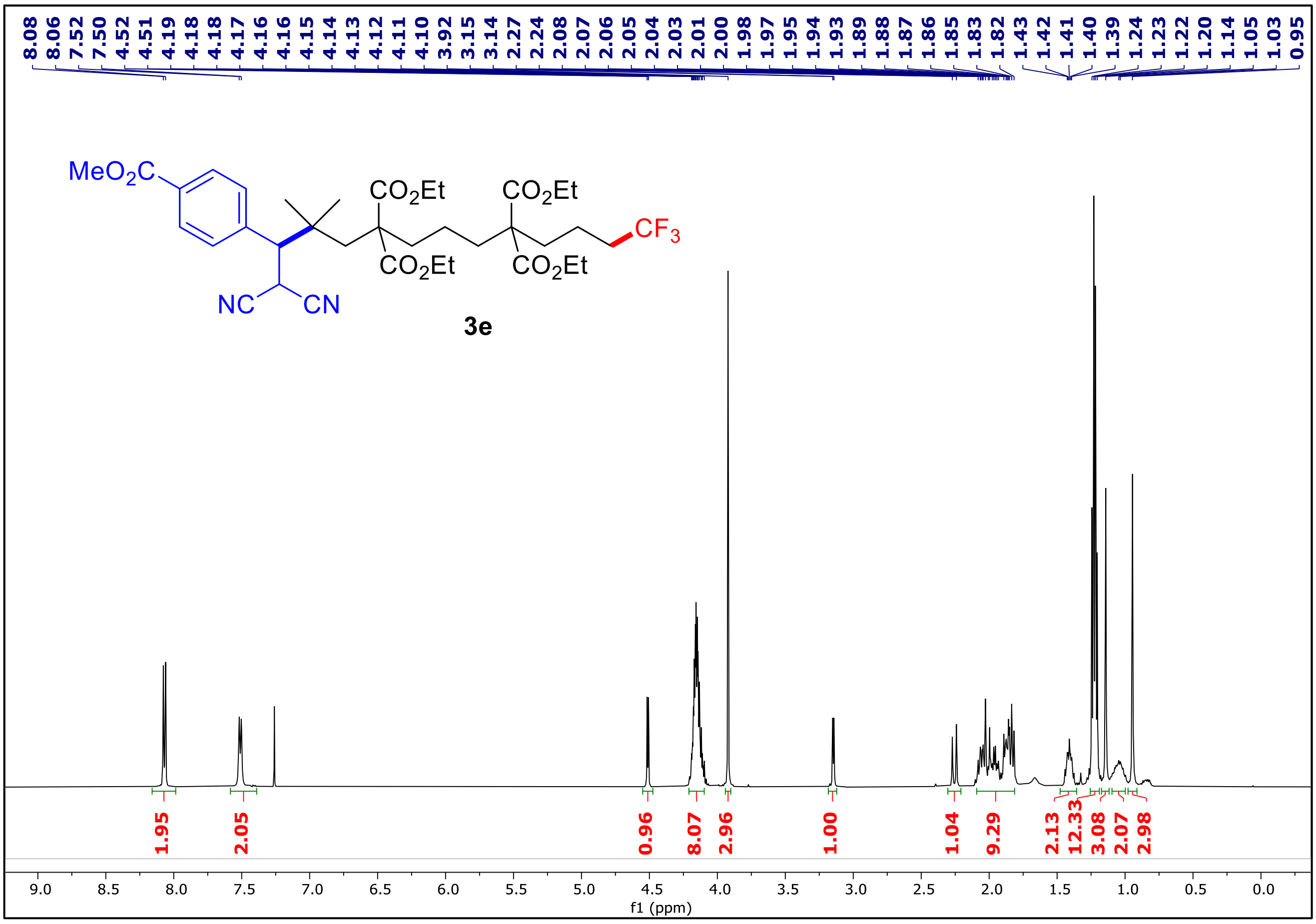

^{1}H NMR of compound **3e** (500 MHz, $CDCl_3$)

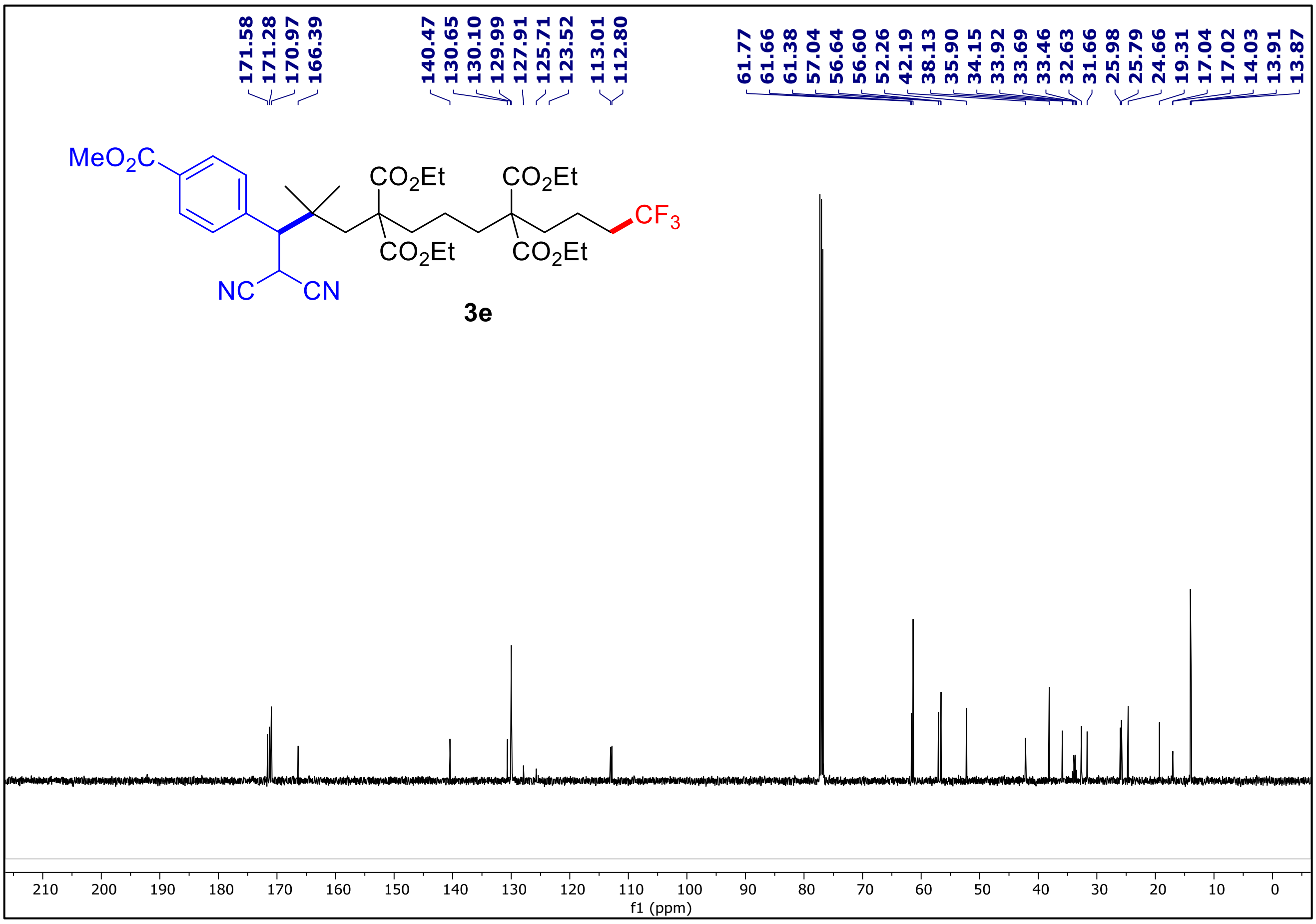

$^{13}C\{^{1}H\}$ NMR of compound **3e** (126 MHz, $CDCl_3$)

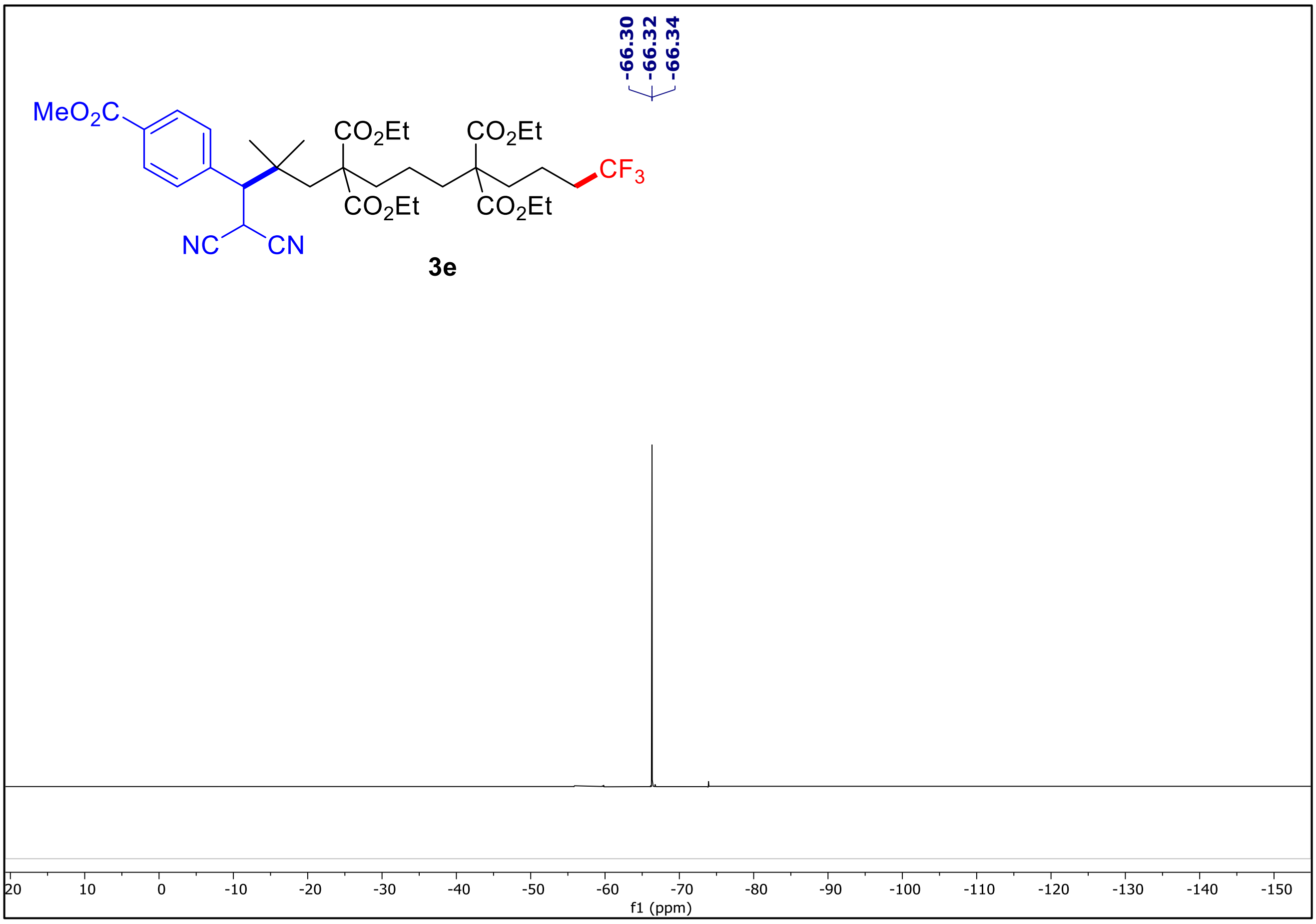

^{19}F NMR of compound **3e** (471 MHz, $CDCl_3$)

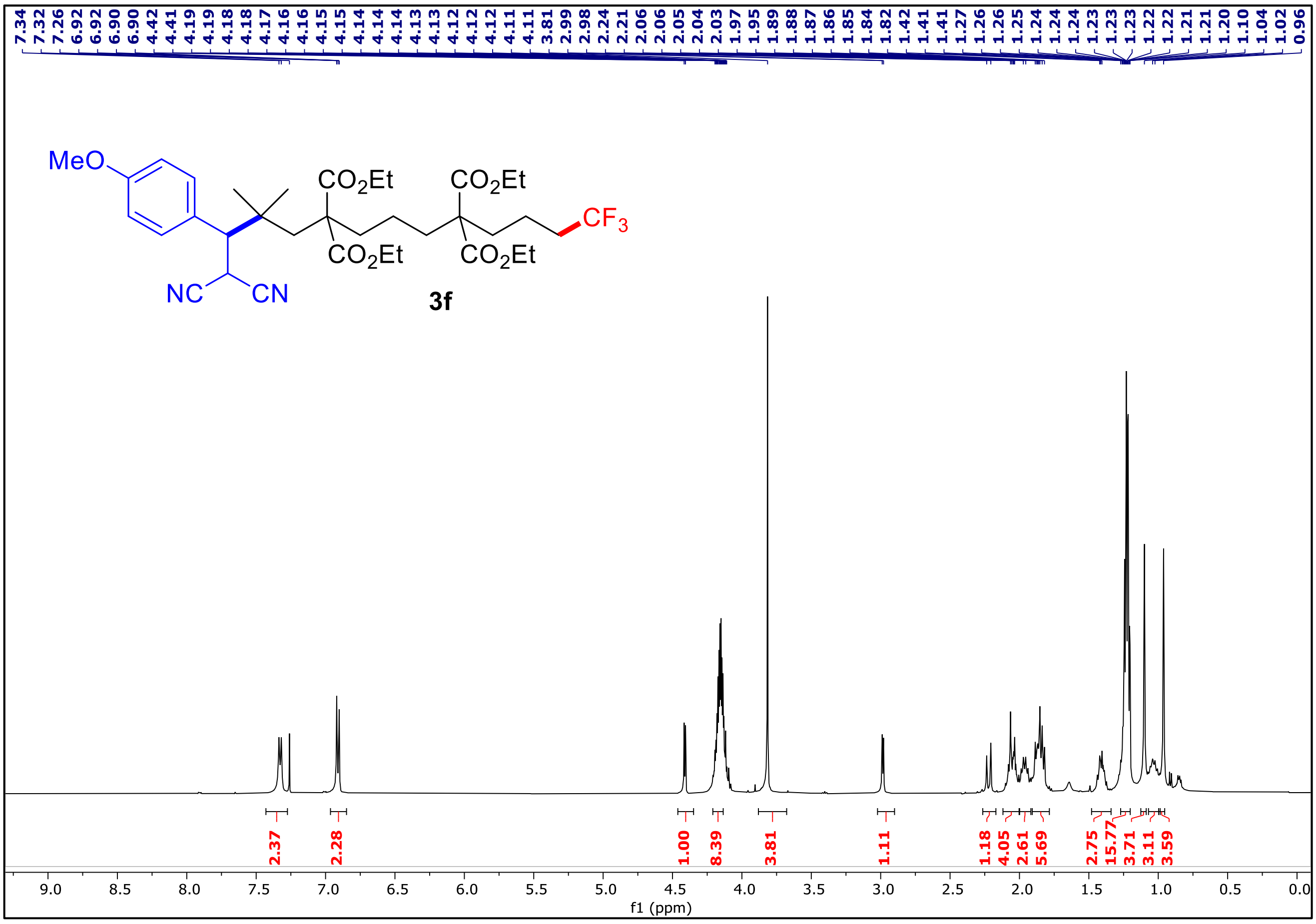

^{1}H NMR of compound **3f** (500 MHz, $CDCl_3$)

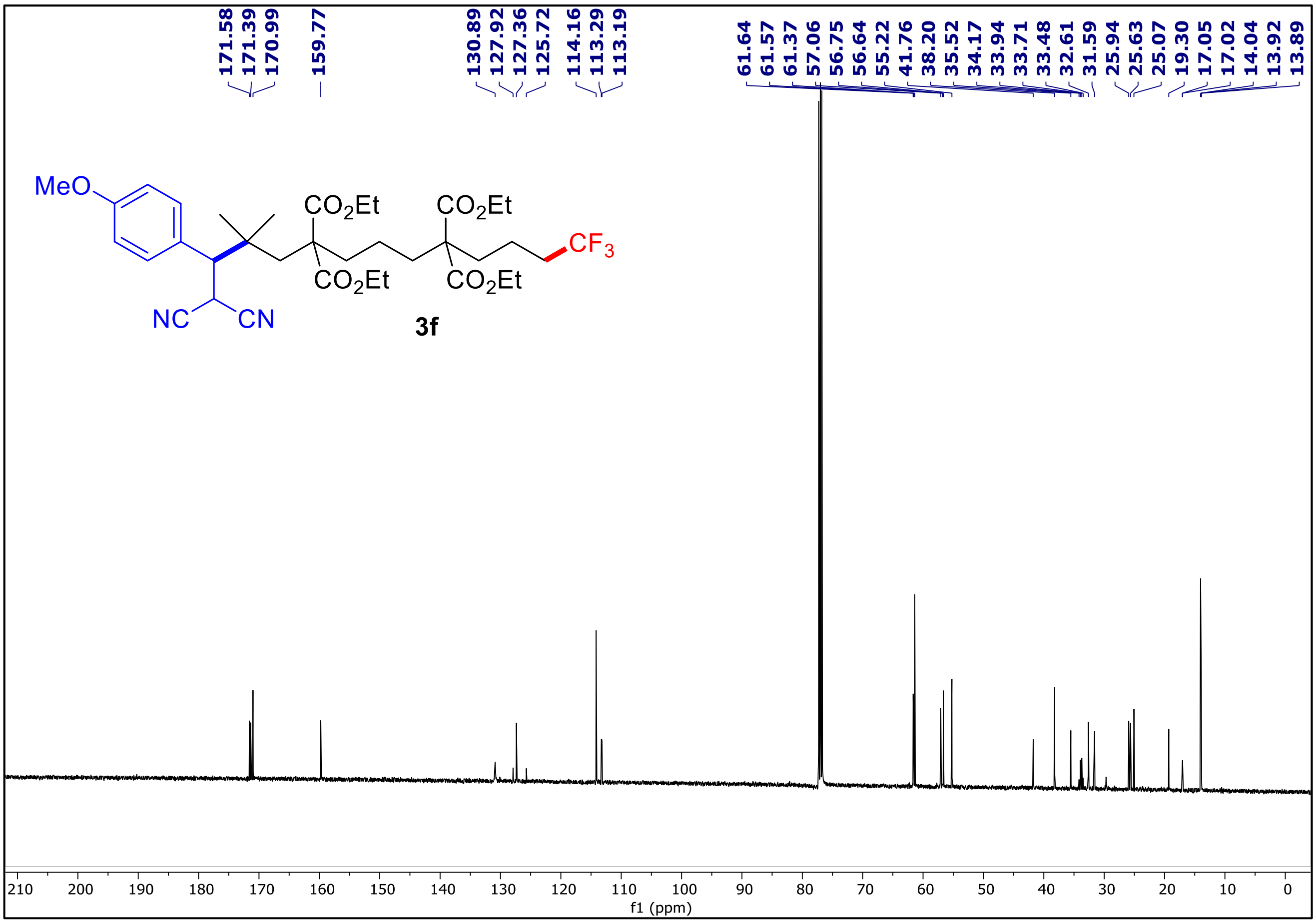

$^{13}C\{^{1}H\}$ NMR of compound **3f** (126 MHz, $CDCl_3$)

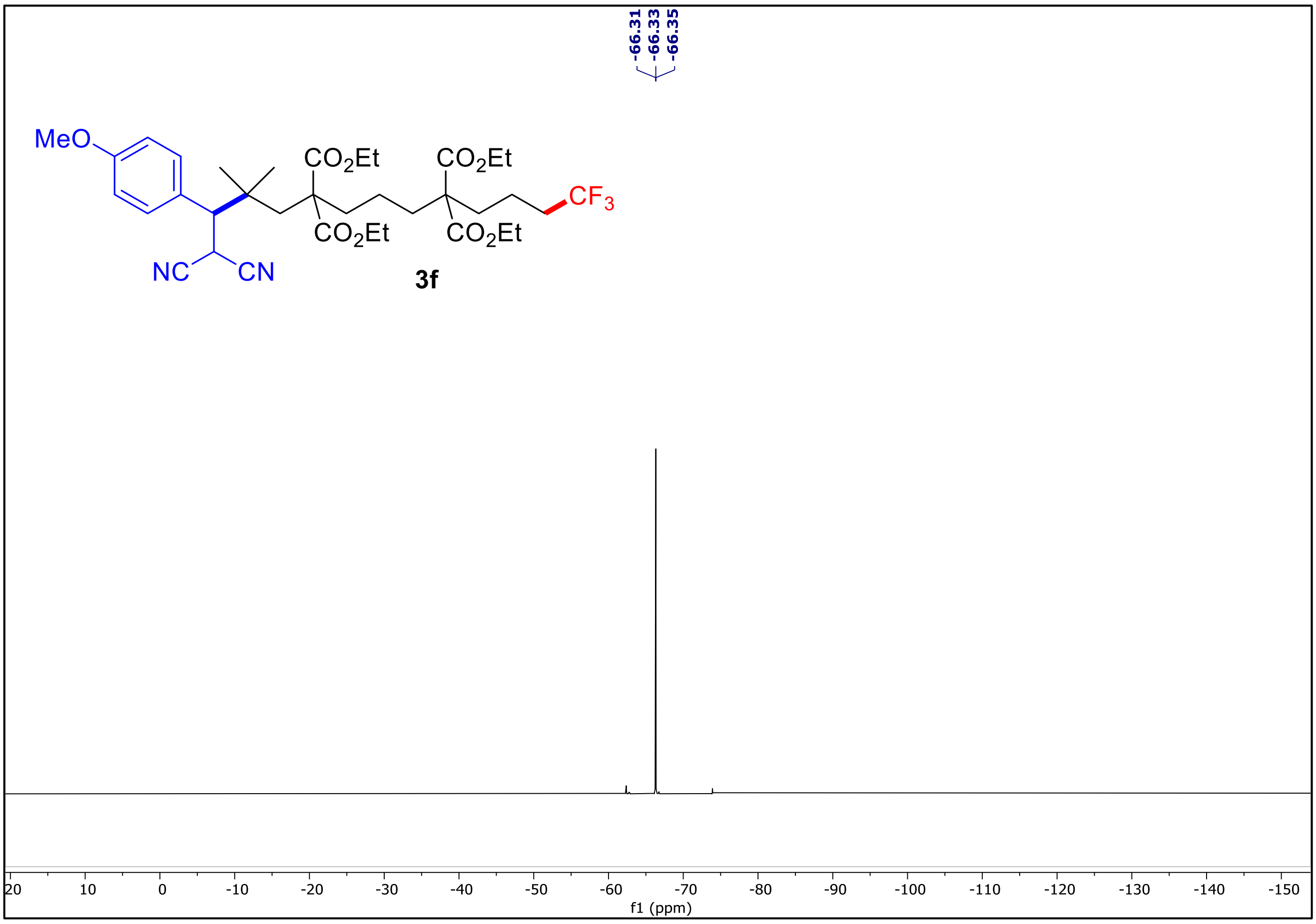

^{19}F NMR of compound **3f** (471 MHz, $CDCl_3$)

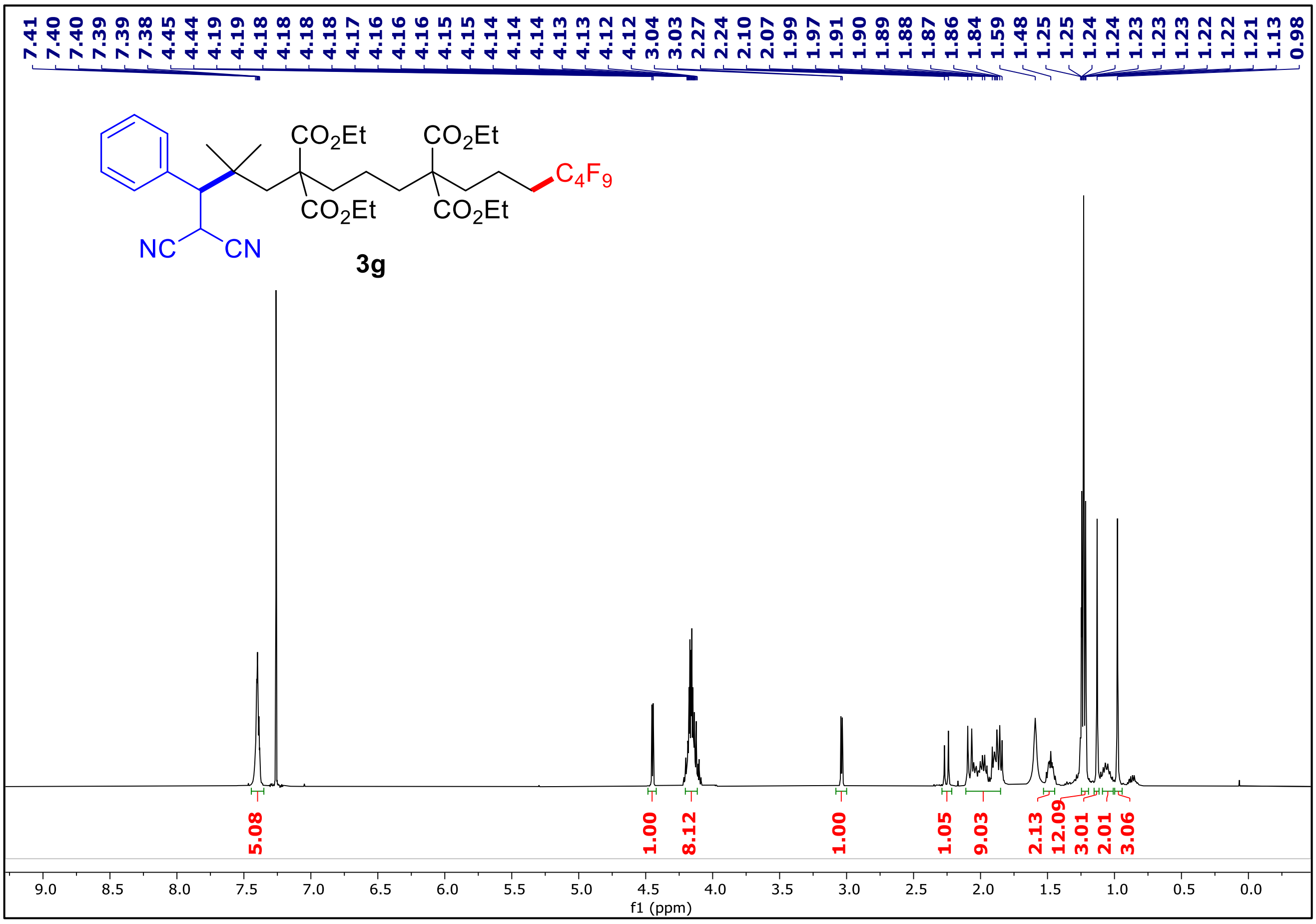

^{1}H NMR of compound **3g** (500 MHz, $CDCl_3$)

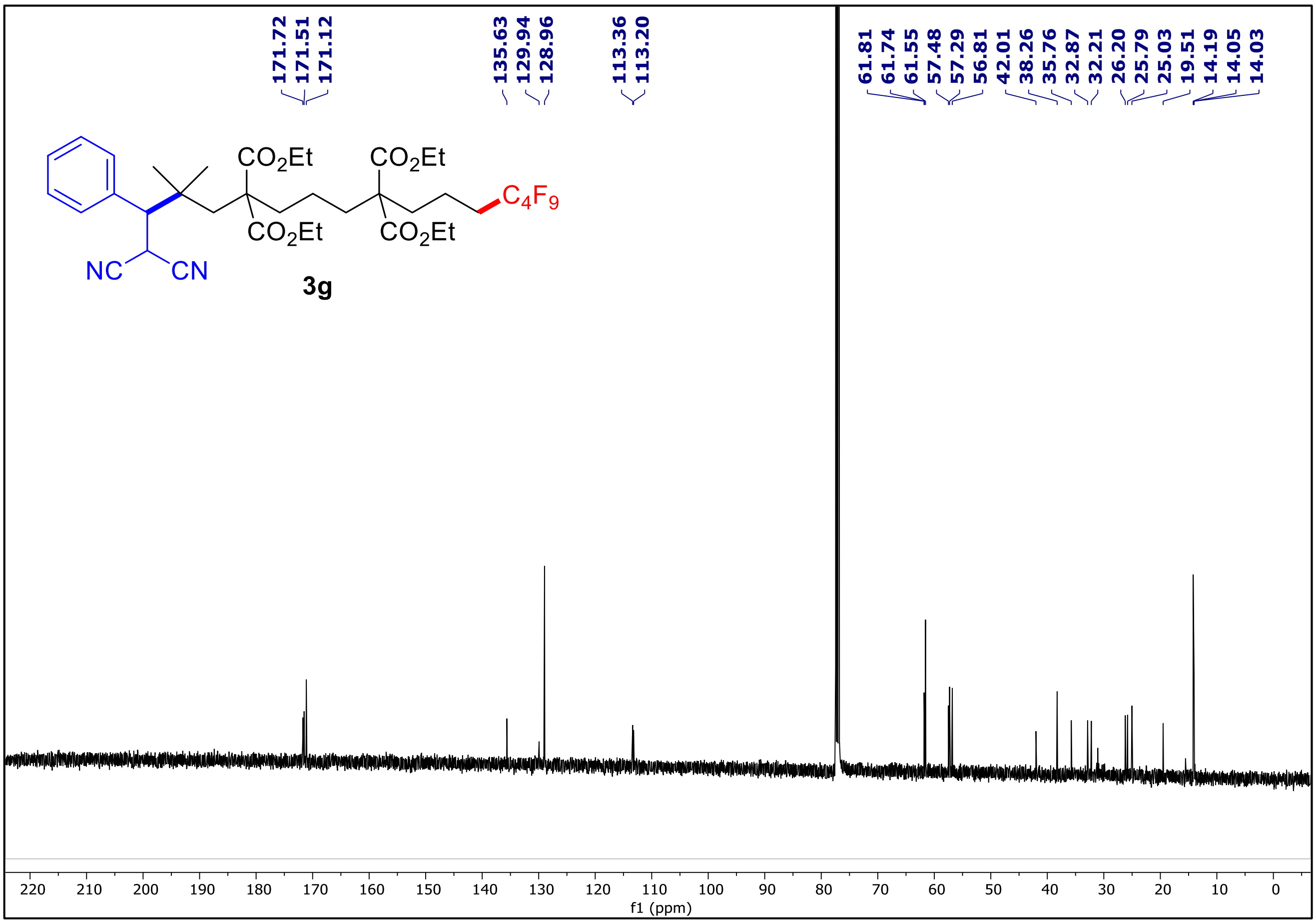

$^{13}C\{^{1}H\}$ NMR of compound **3g** (126 MHz, $CDCl_3$)

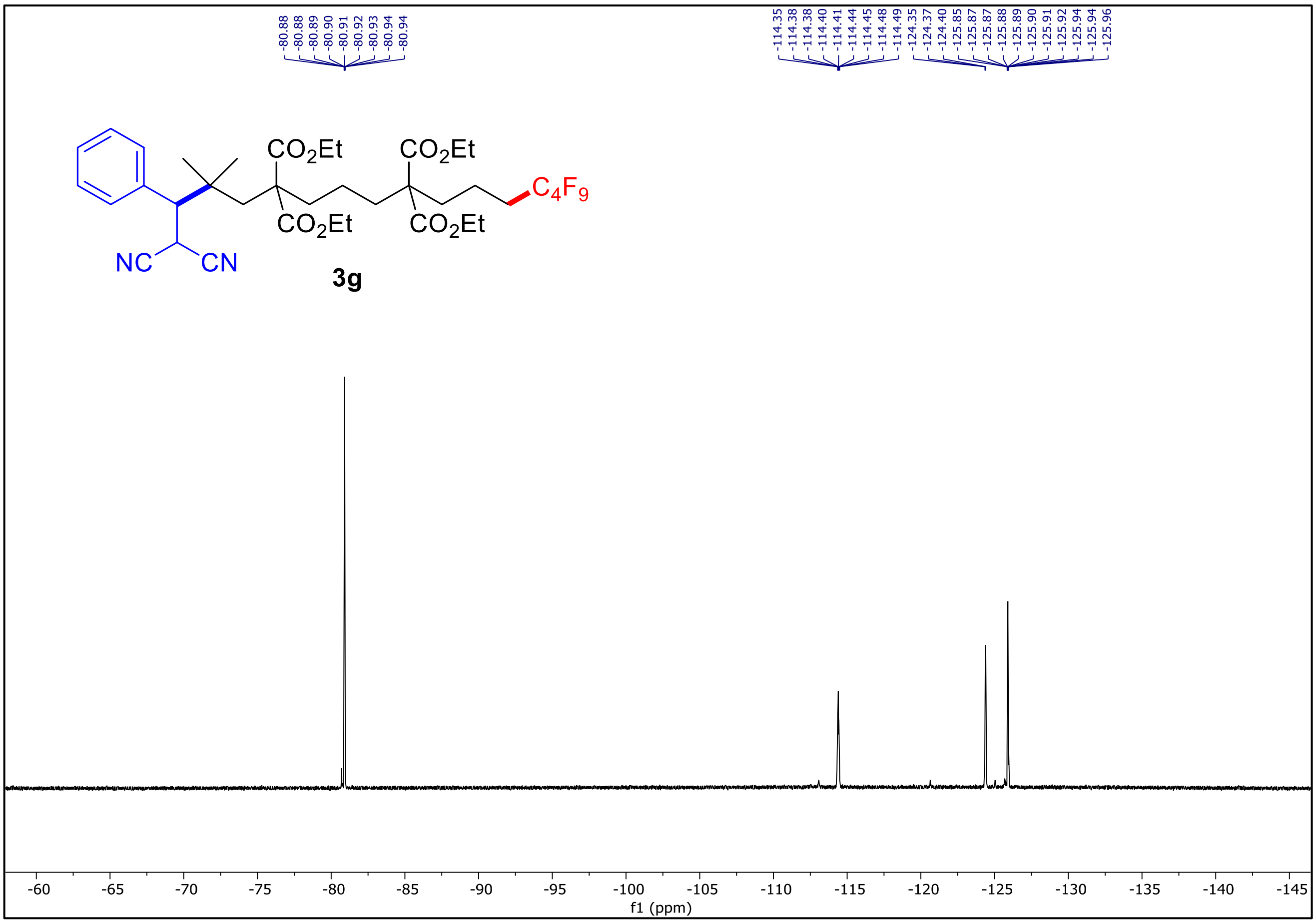

^{19}F NMR of compound **3g** (376 MHz, $CDCl_3$)

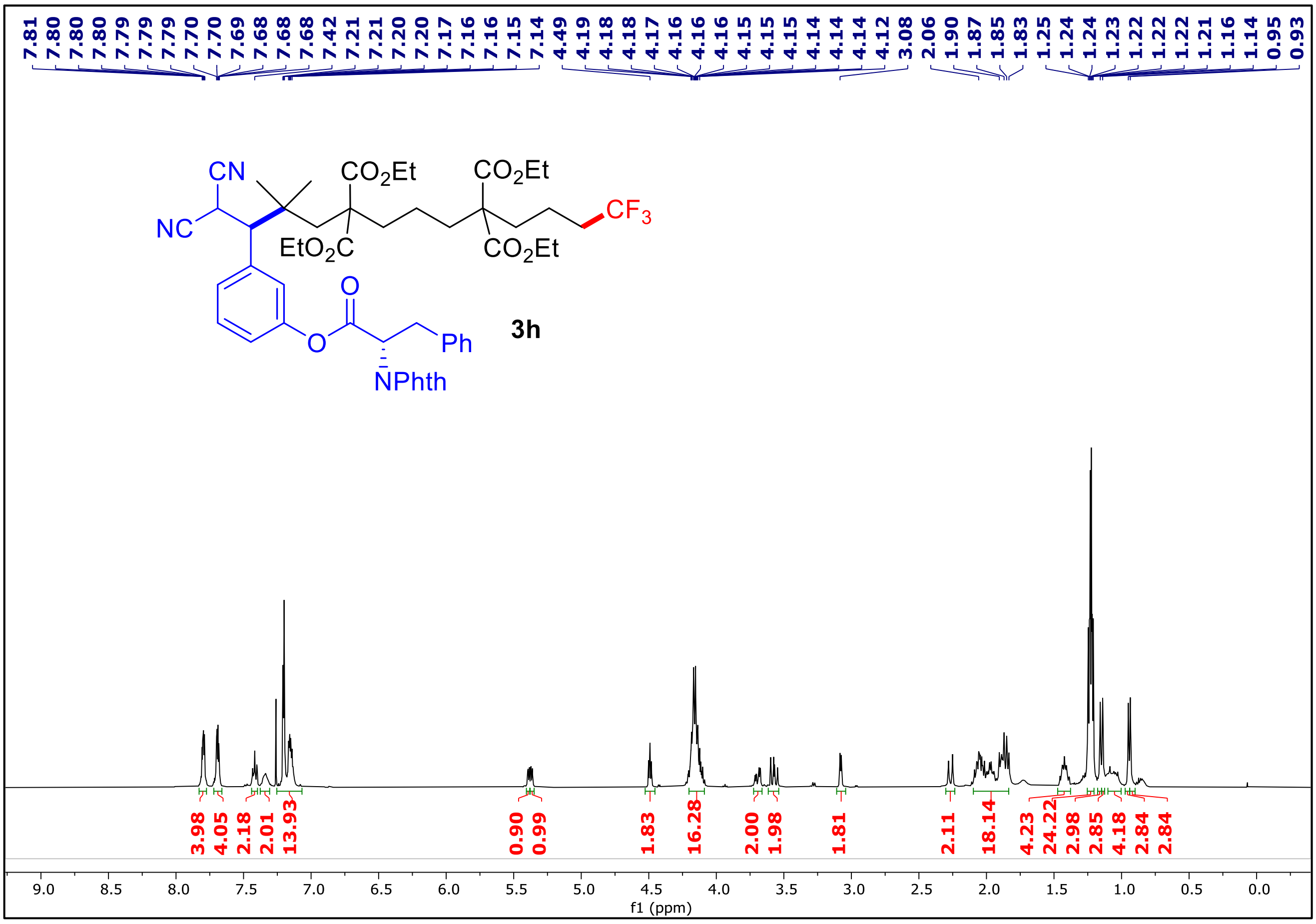

^{1}H NMR of compound **3h** (500 MHz, $CDCl_3$)

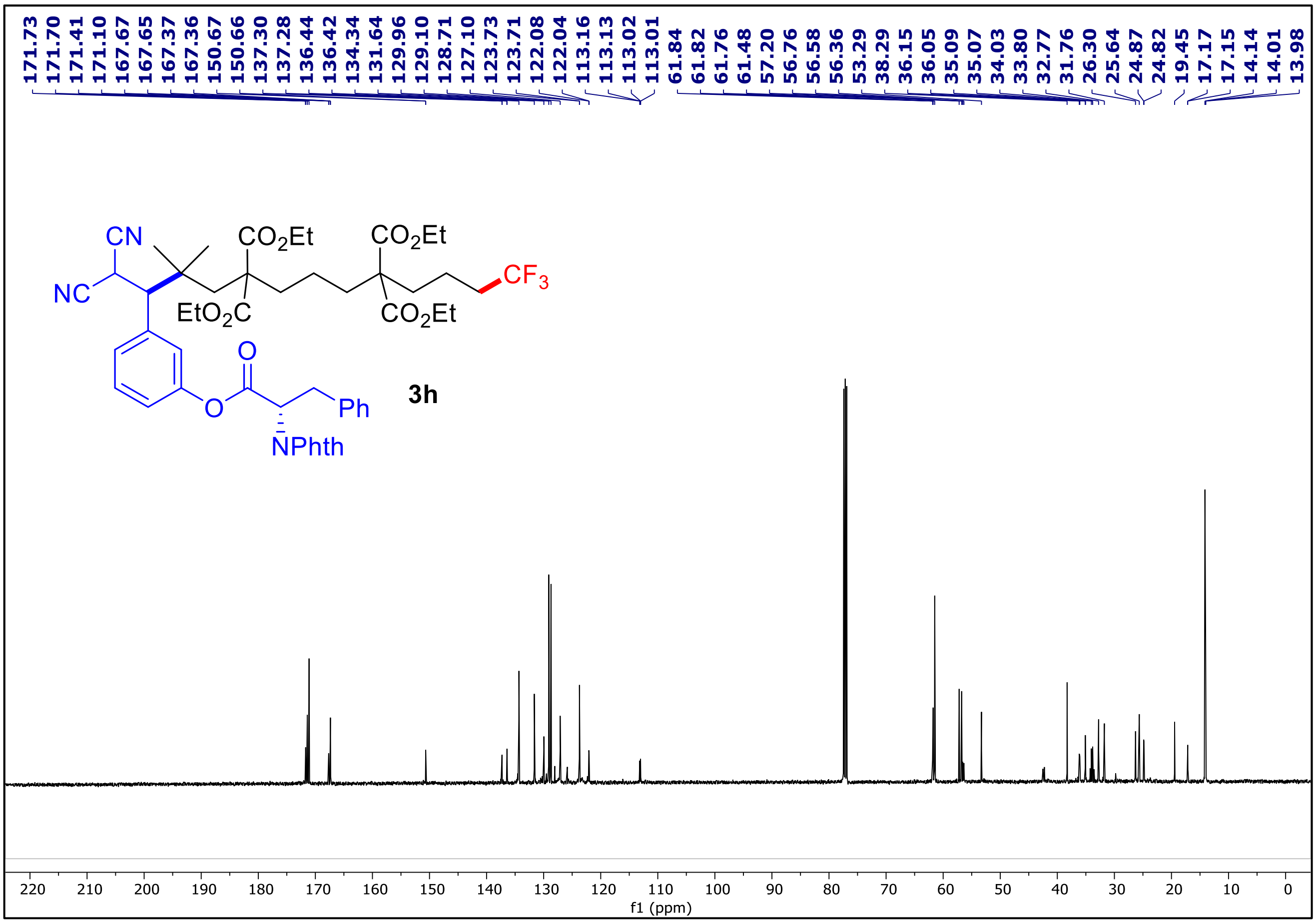

$^{13}C\{^1H\}$ NMR of compound **3h** (126 MHz, $CDCl_3$)

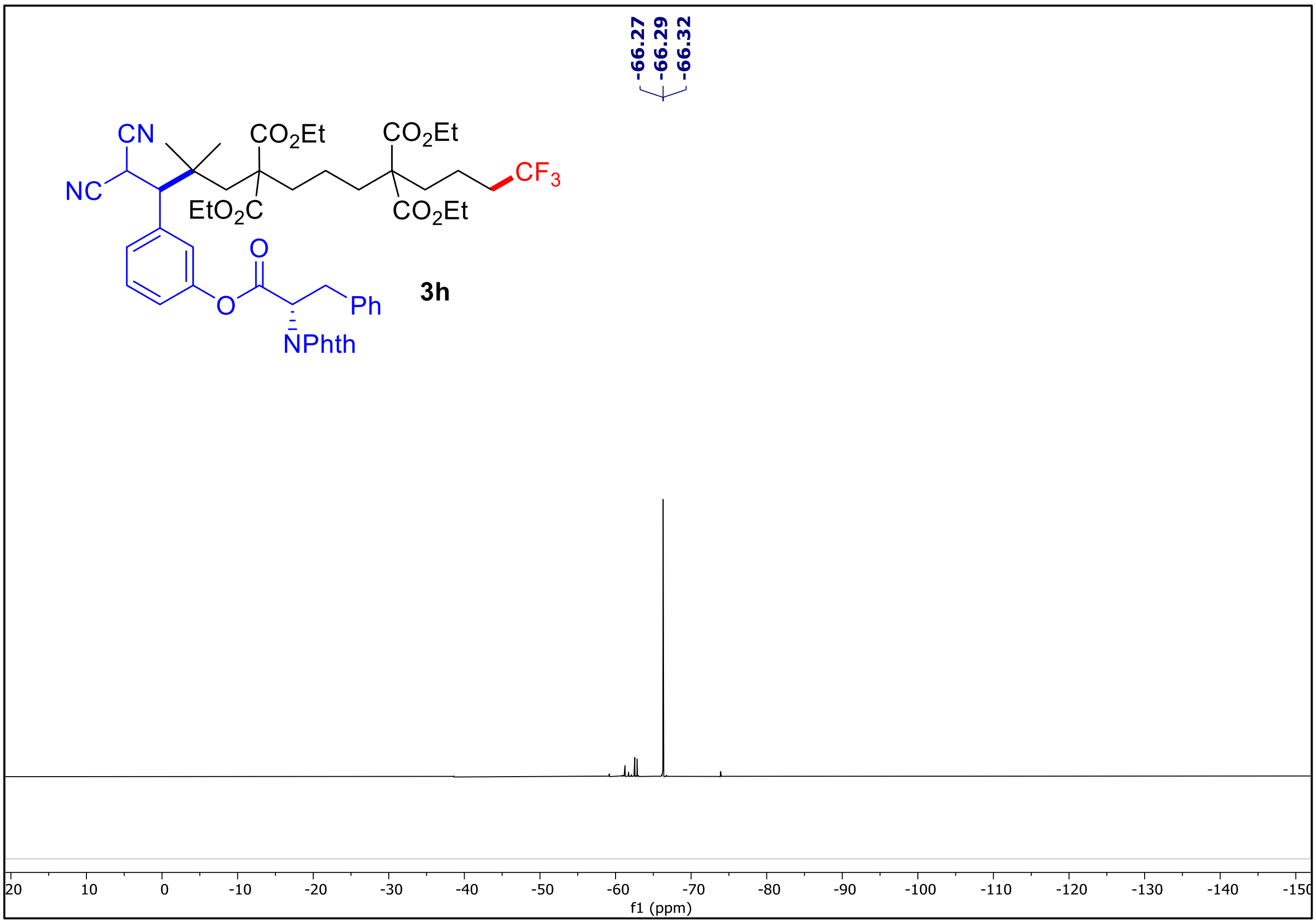

^{19}F NMR of compound **3h** (471 MHz, $CDCl_3$)

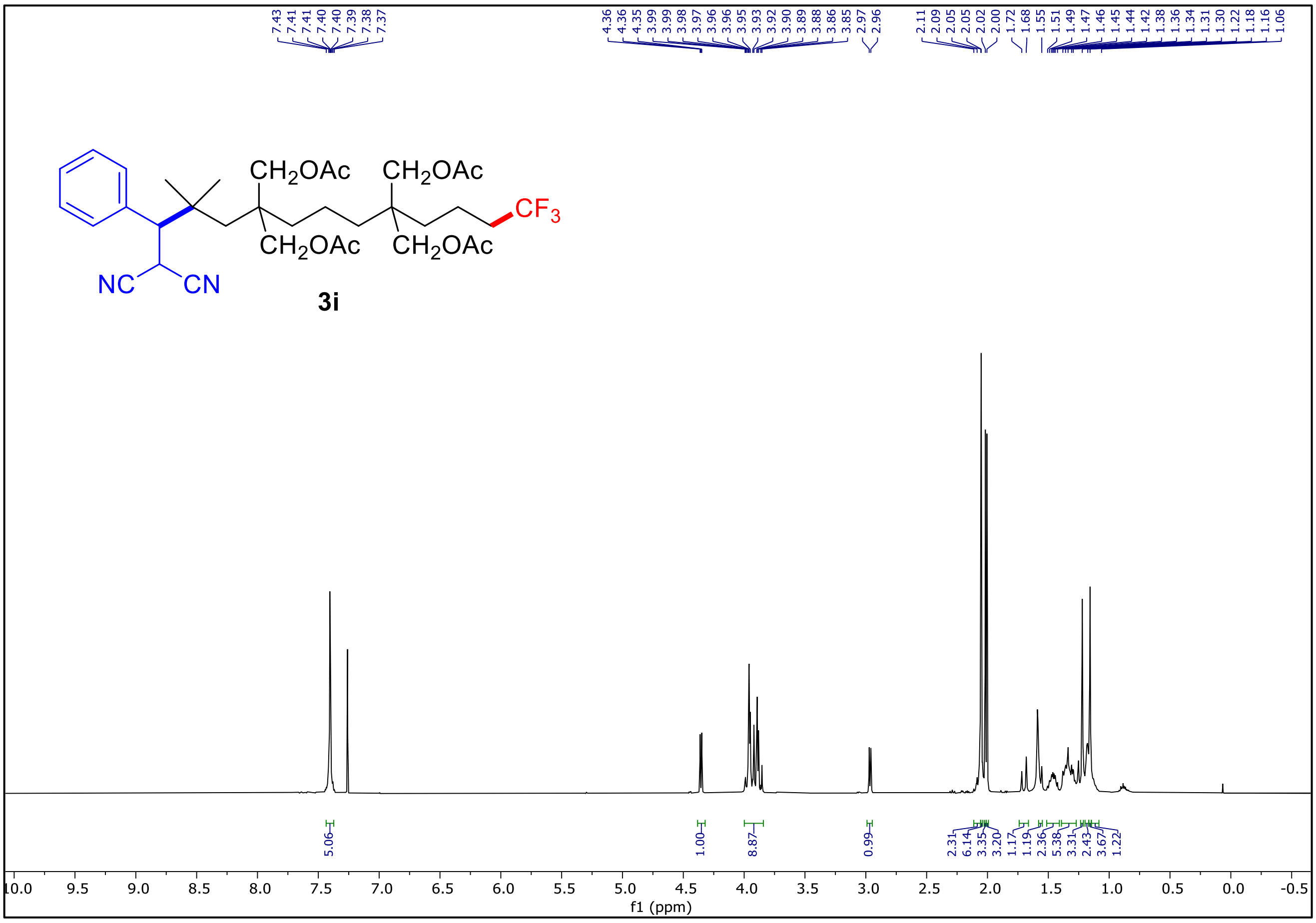

^{1}H NMR of compound **3i** (400 MHz, $CDCl_3$)

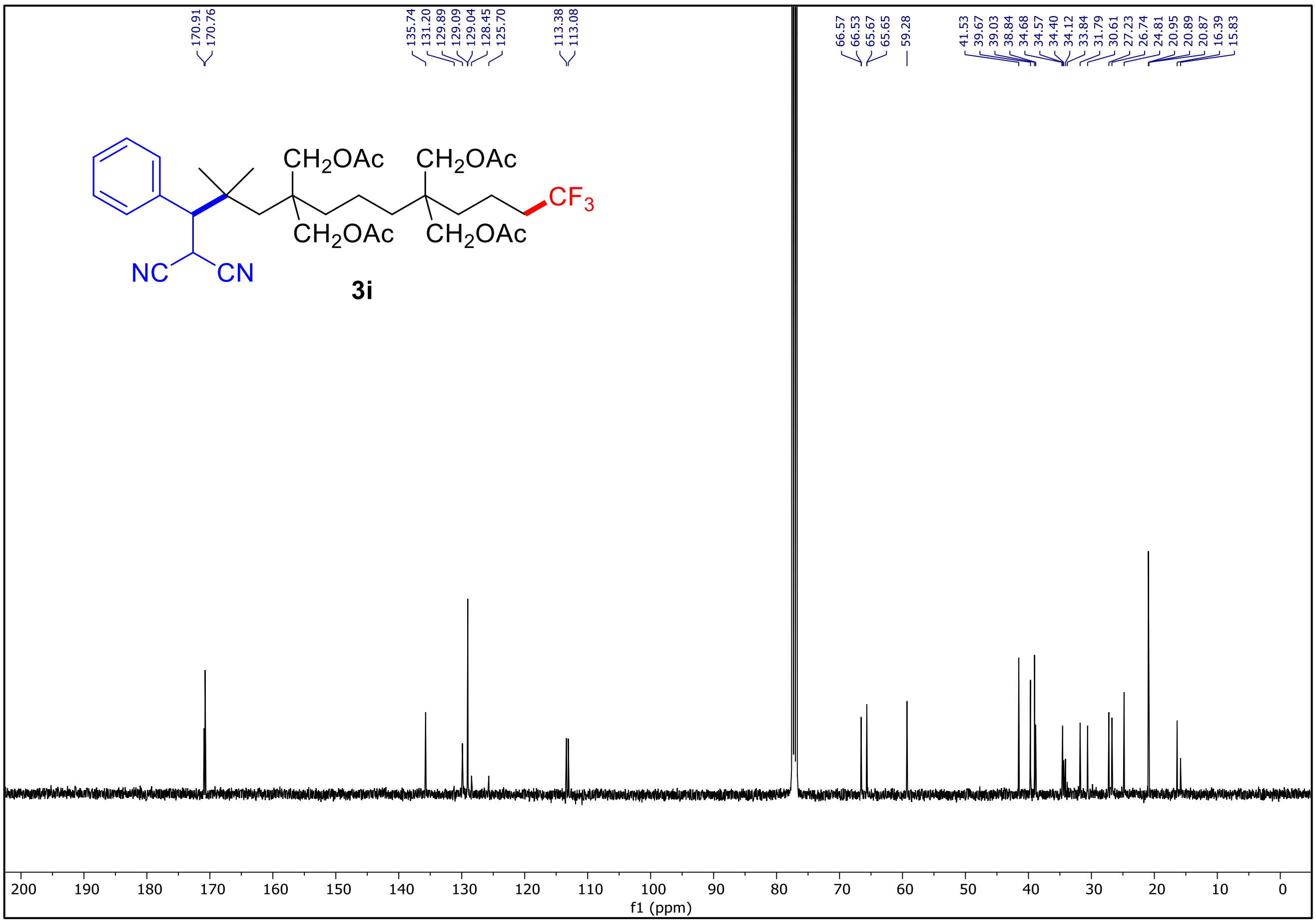

$^{13}C\{^1H\}$ NMR of compound **3i** (101 MHz, $CDCl_3$)

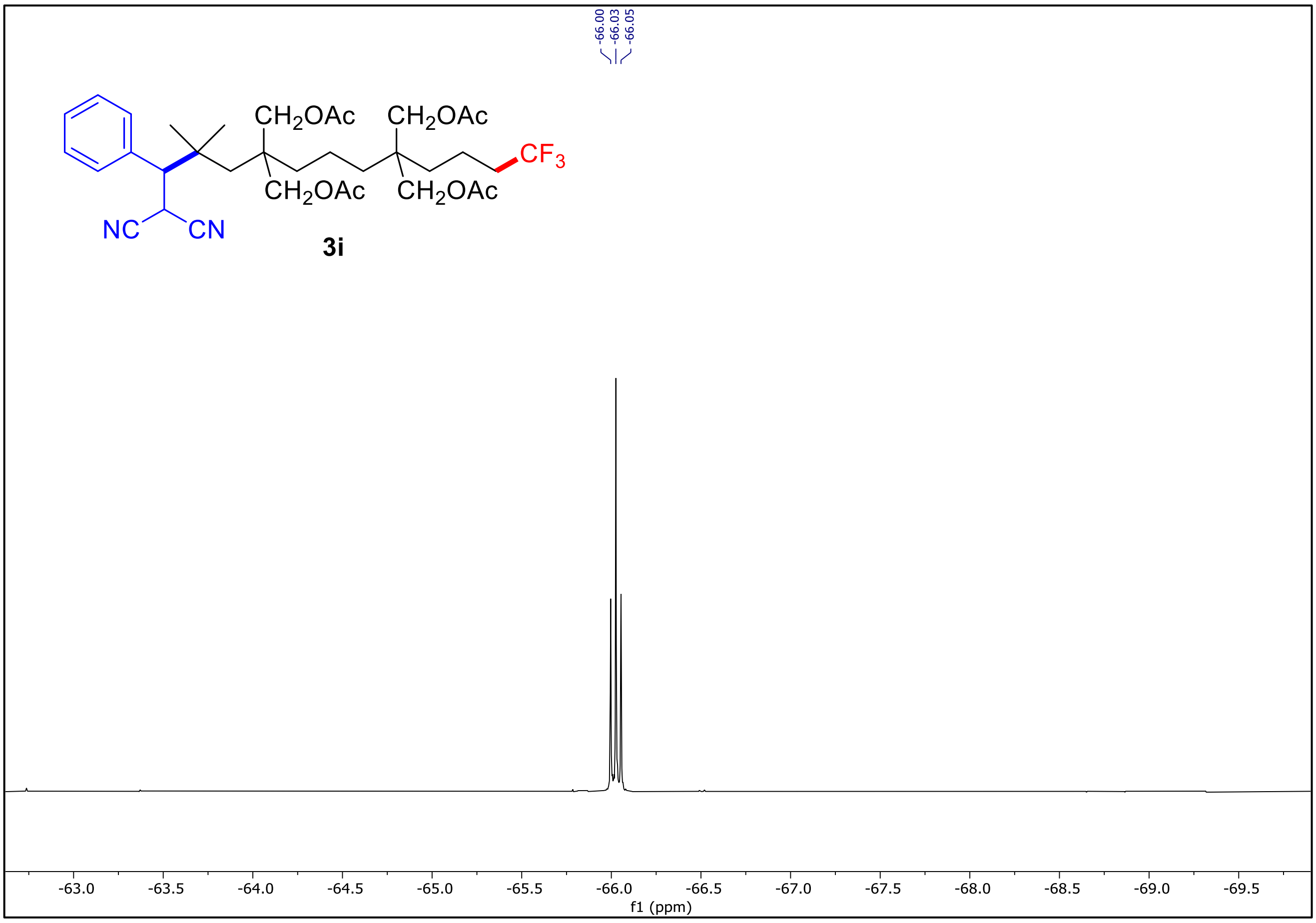

^{19}F NMR of compound **3i** (376 MHz, $CDCl_3$)

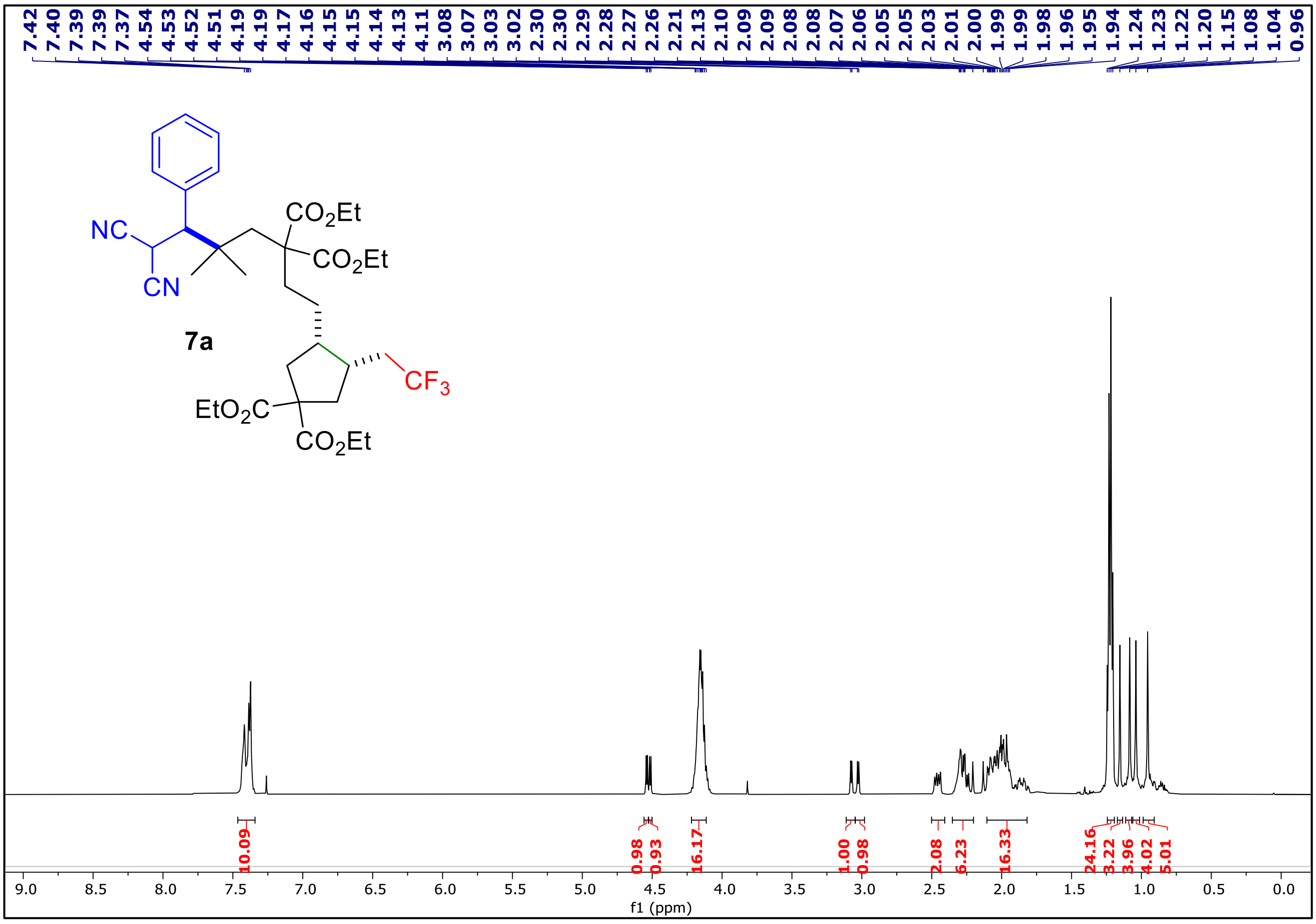

^{1}H NMR of compound **7a** (500 MHz, $CDCl_3$)

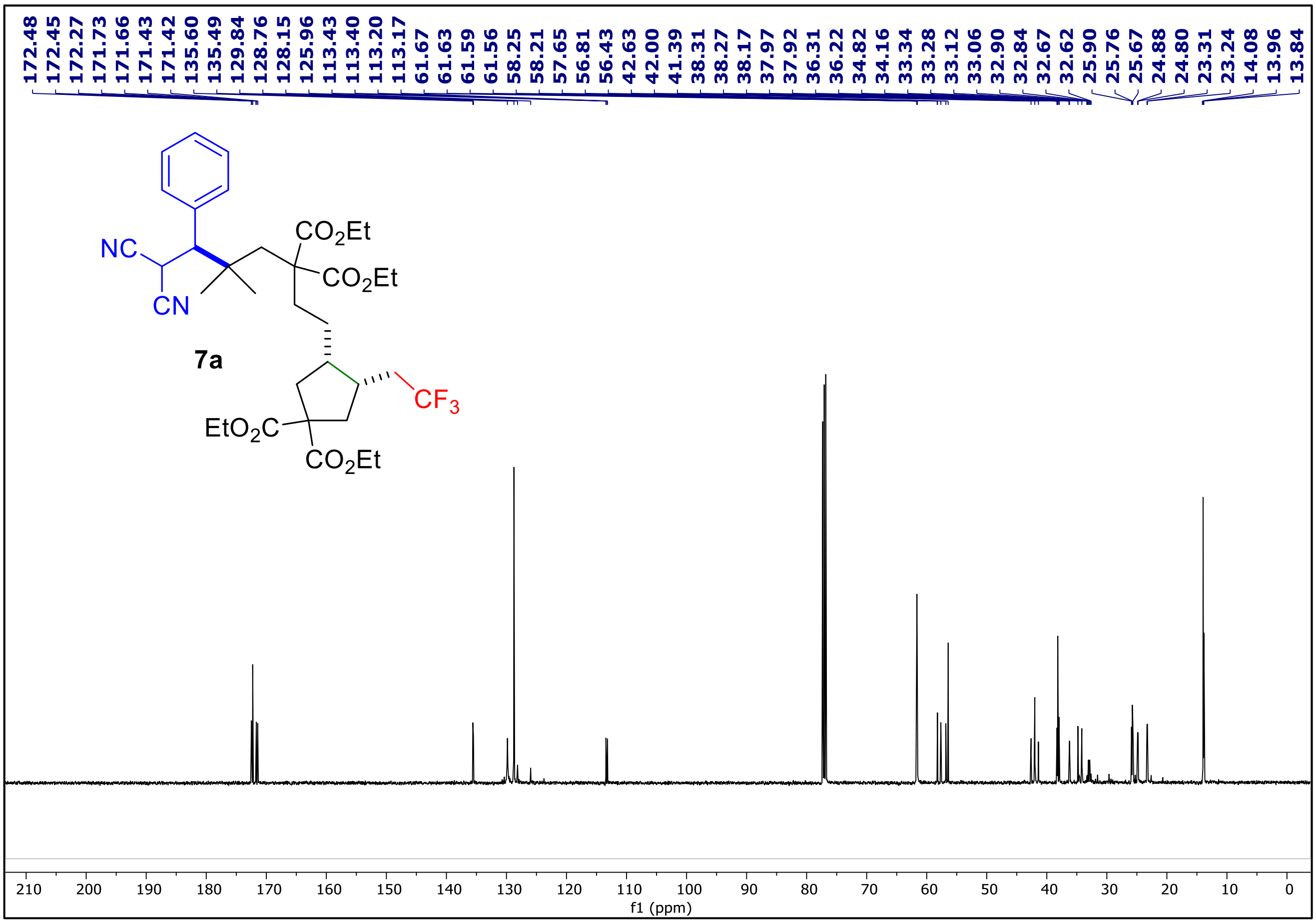

$^{13}C\{^1H\}$ NMR of compound **7a** (126 MHz, $CDCl_3$)

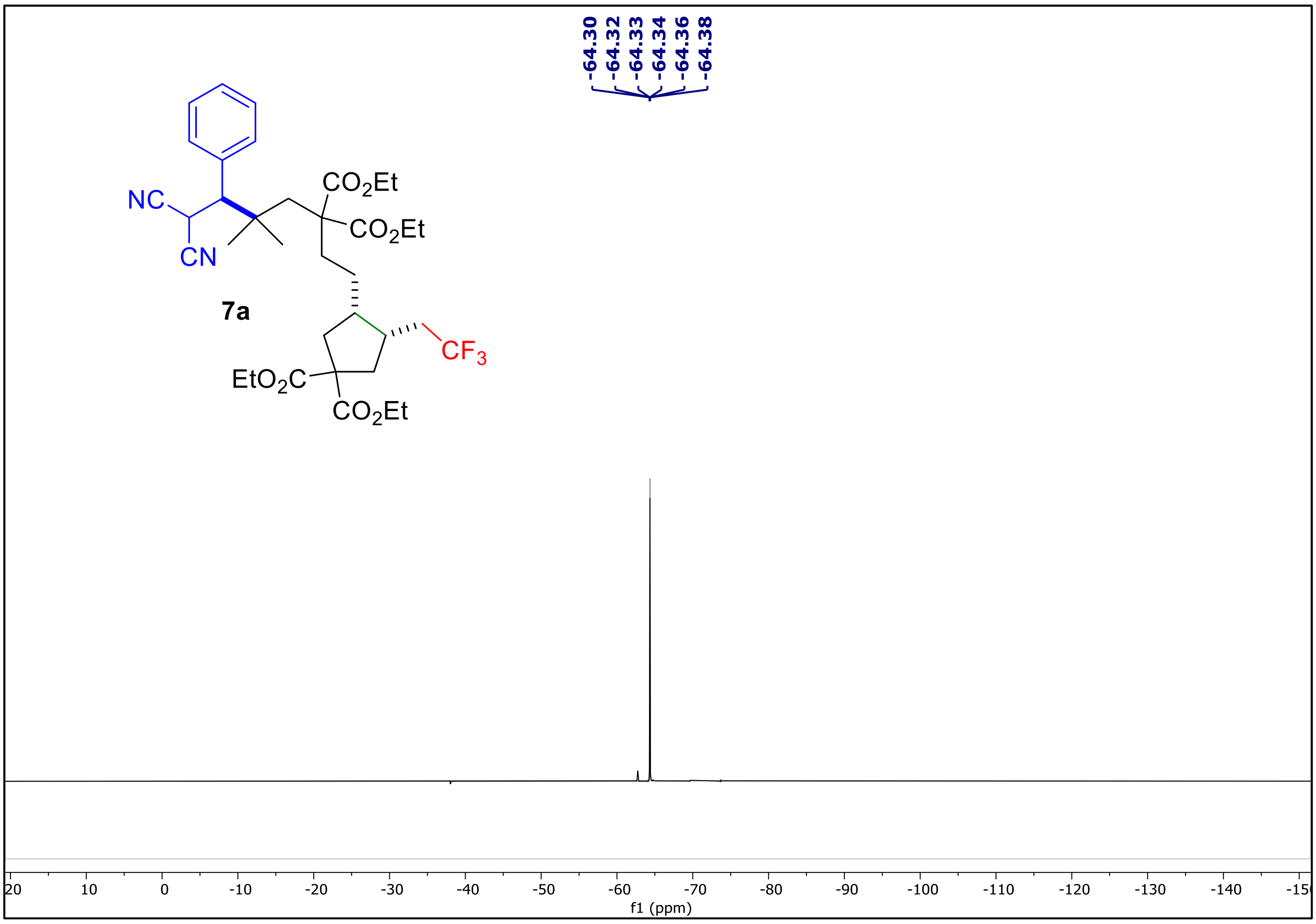

^{19}F NMR of compound **7a** (471 MHz, $CDCl_3$)

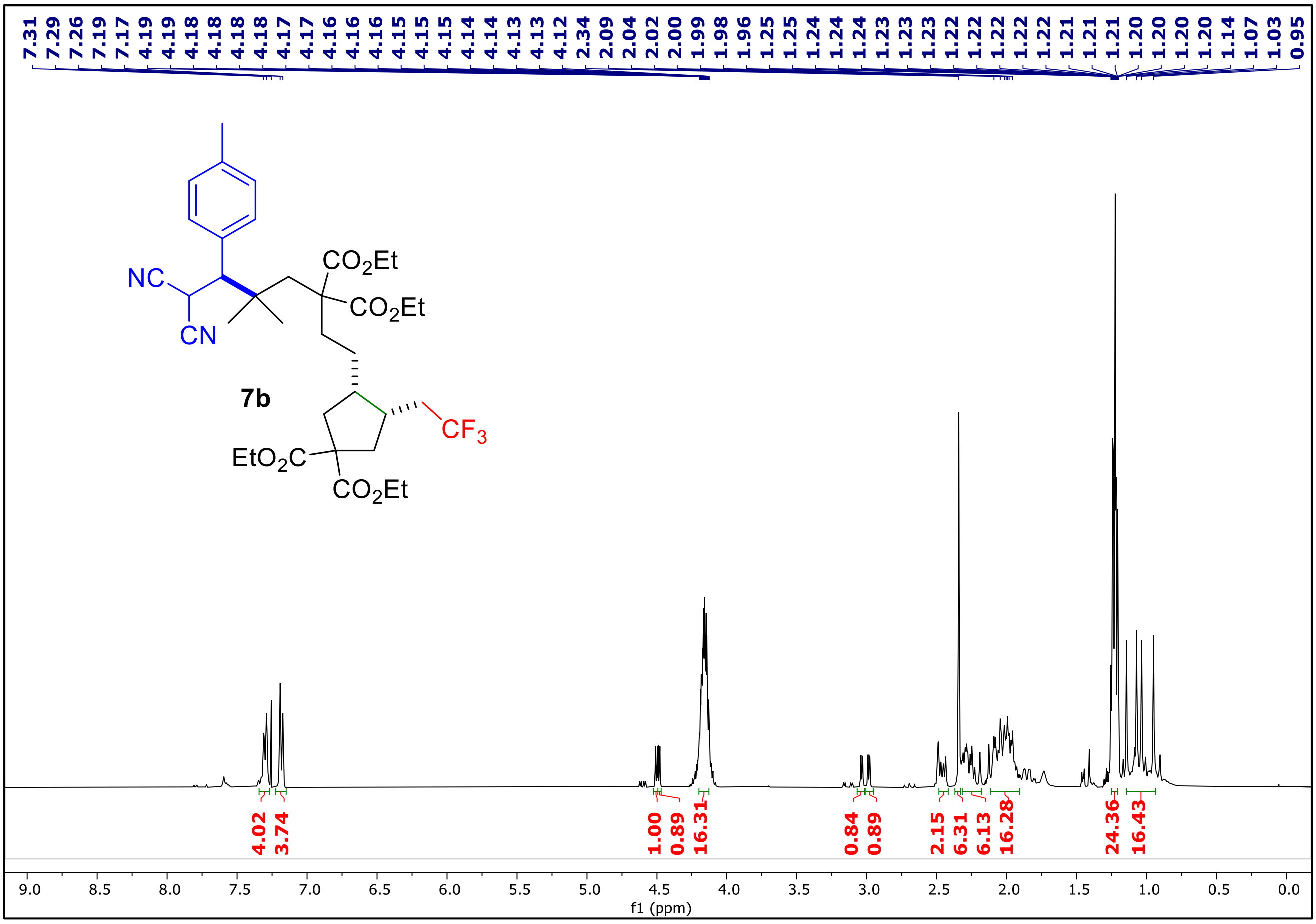

^{1}H NMR of compound **7b** (400 MHz, $CDCl_3$)

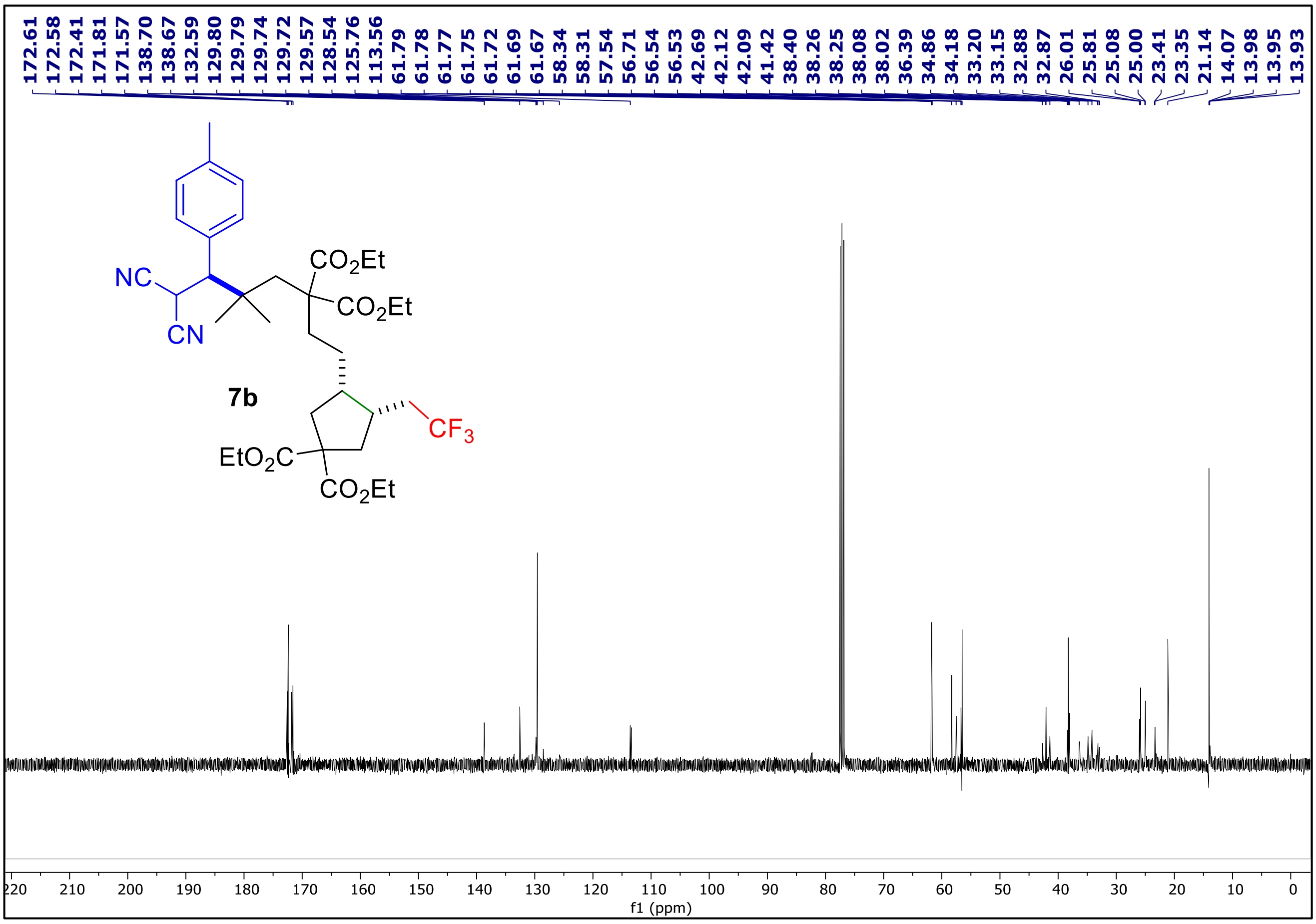

$^{13}C\{^1H\}$ NMR of compound **7b** (101 MHz, $CDCl_3$)

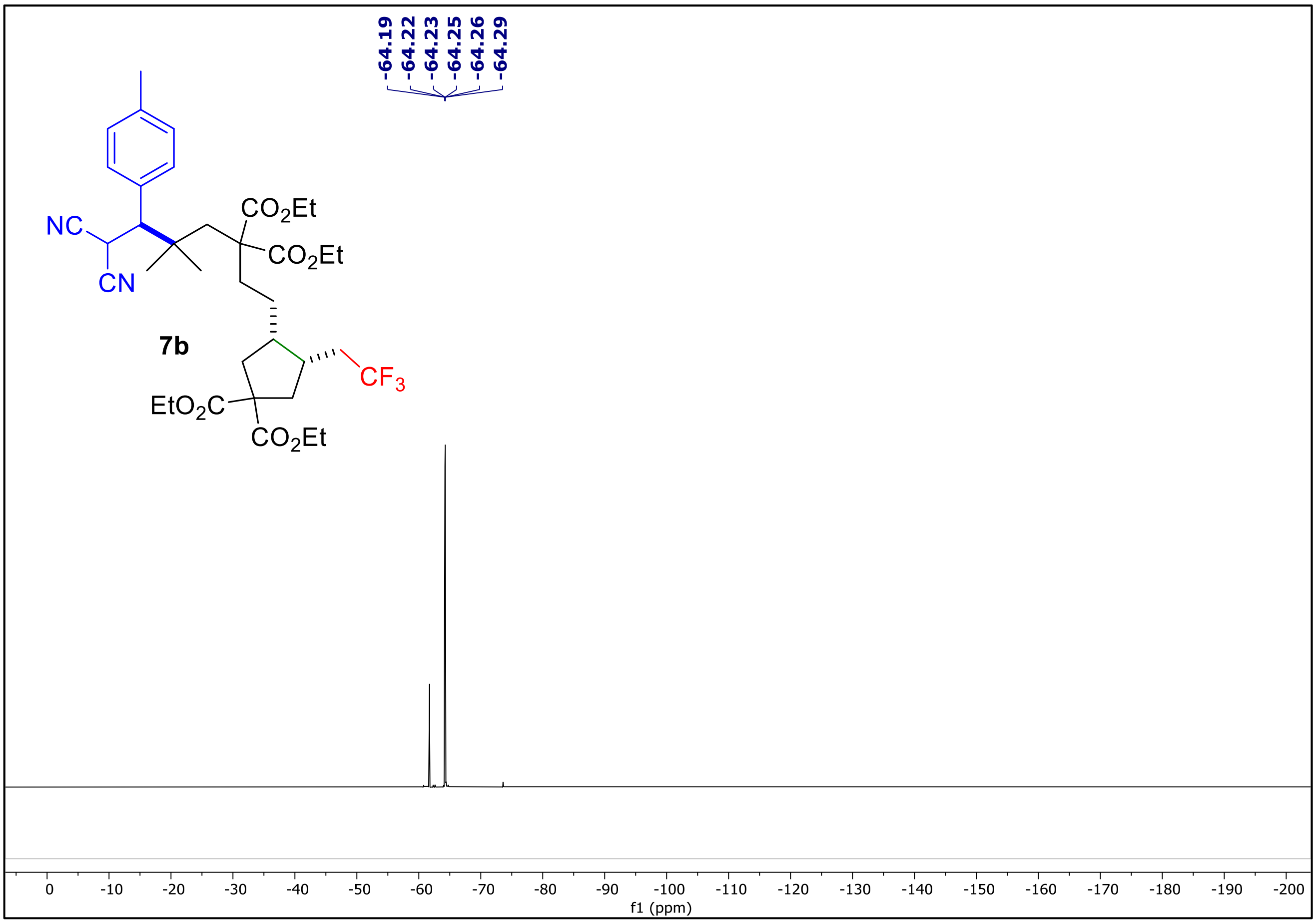

^{19}F NMR of compound **7b** (376 MHz, $CDCl_3$)

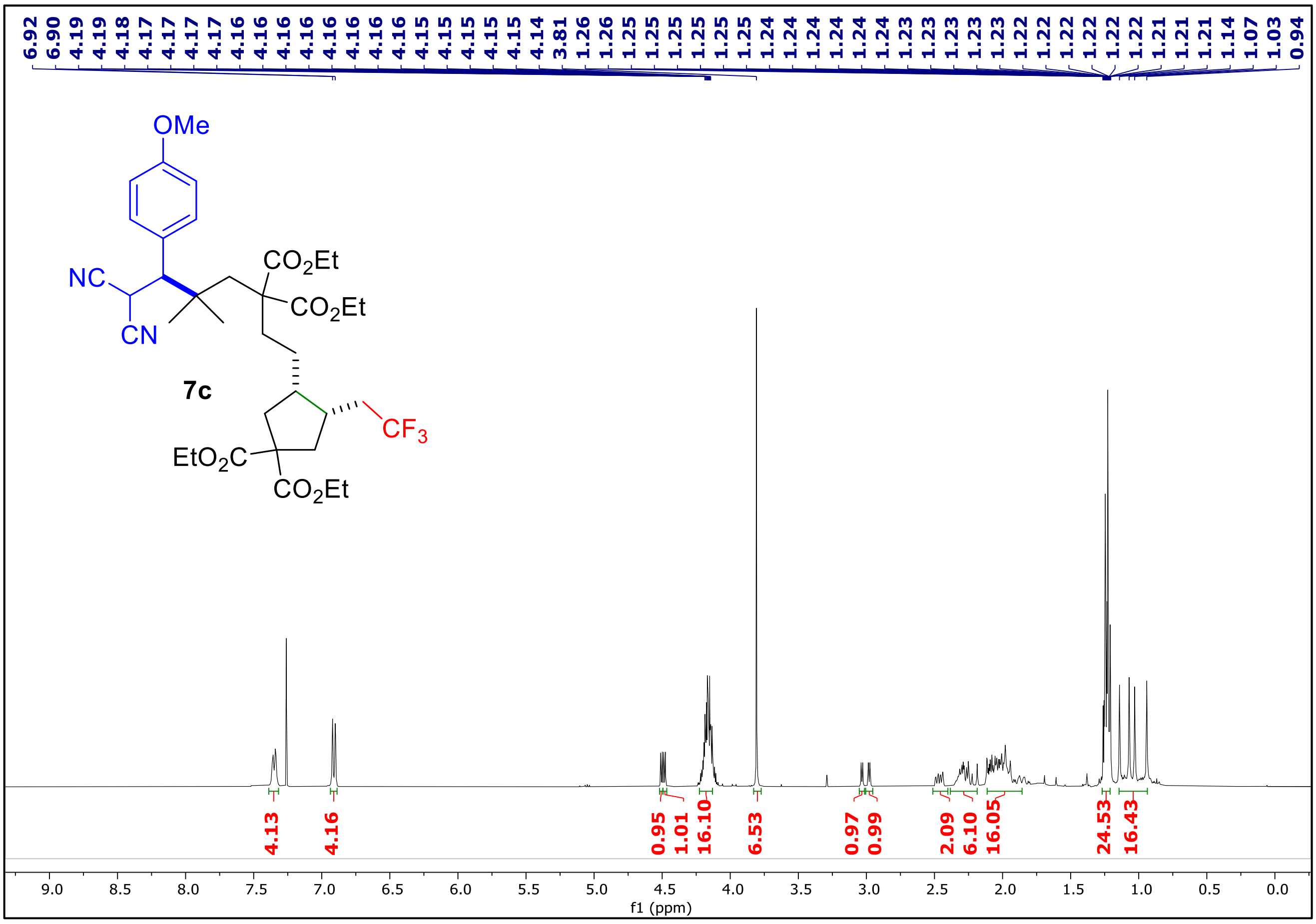

^{1}H NMR of compound **7c** (400 MHz, $CDCl_3$)

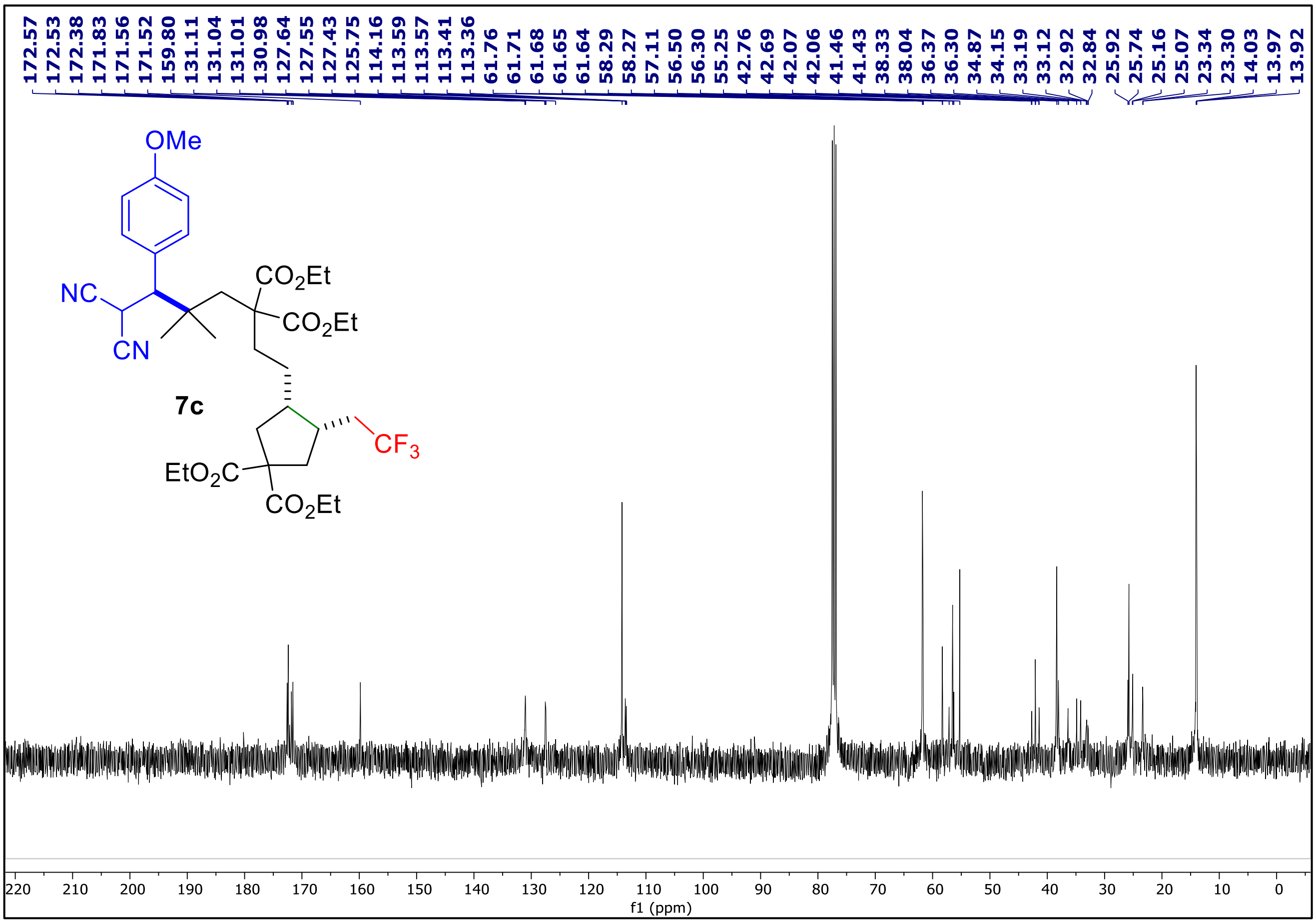

$^{13}C\{^{1}H\}$ NMR of compound **7c** (101 MHz, $CDCl_3$)

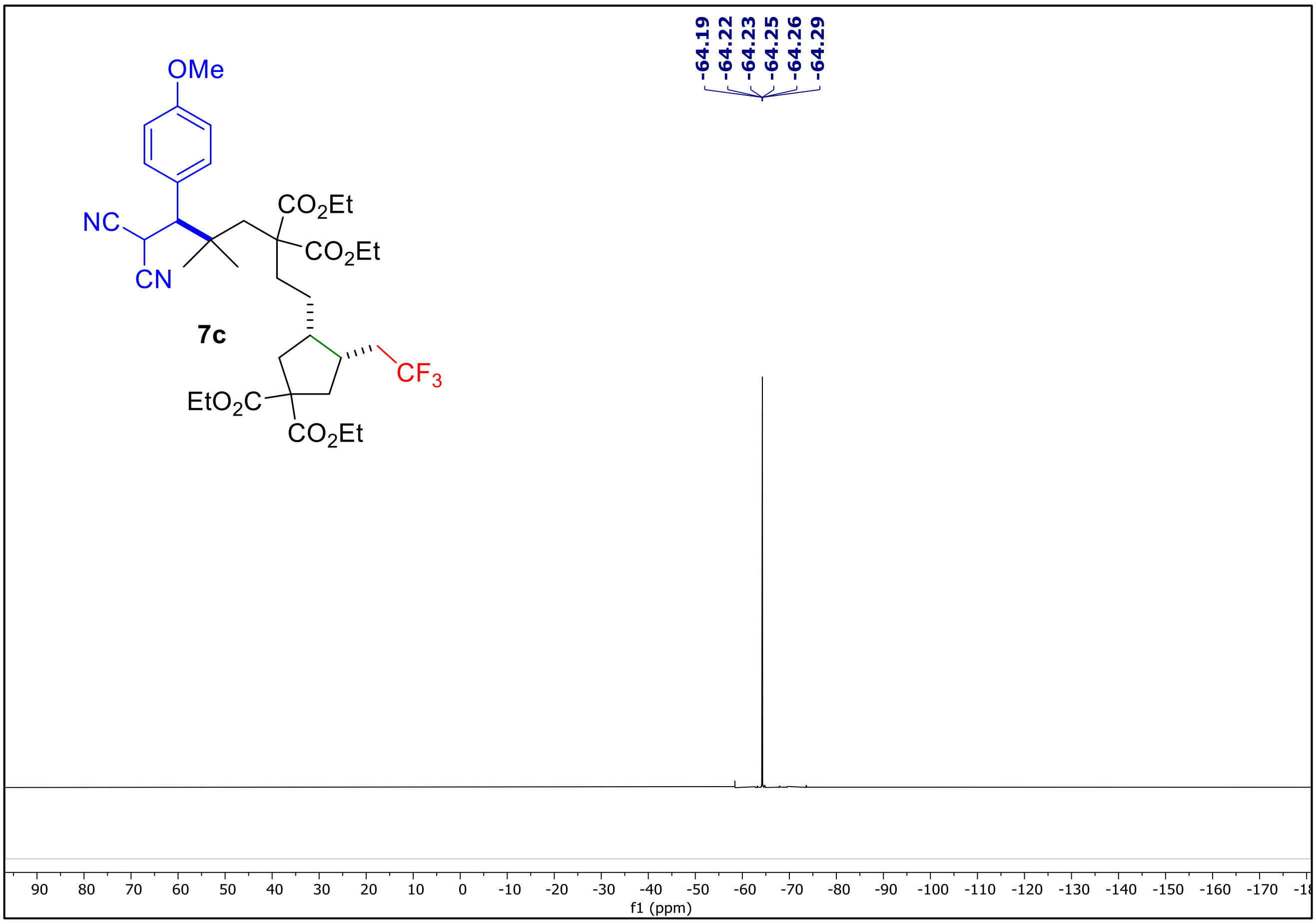

^{19}F NMR of compound **7c** (376 MHz, $CDCl_3$)

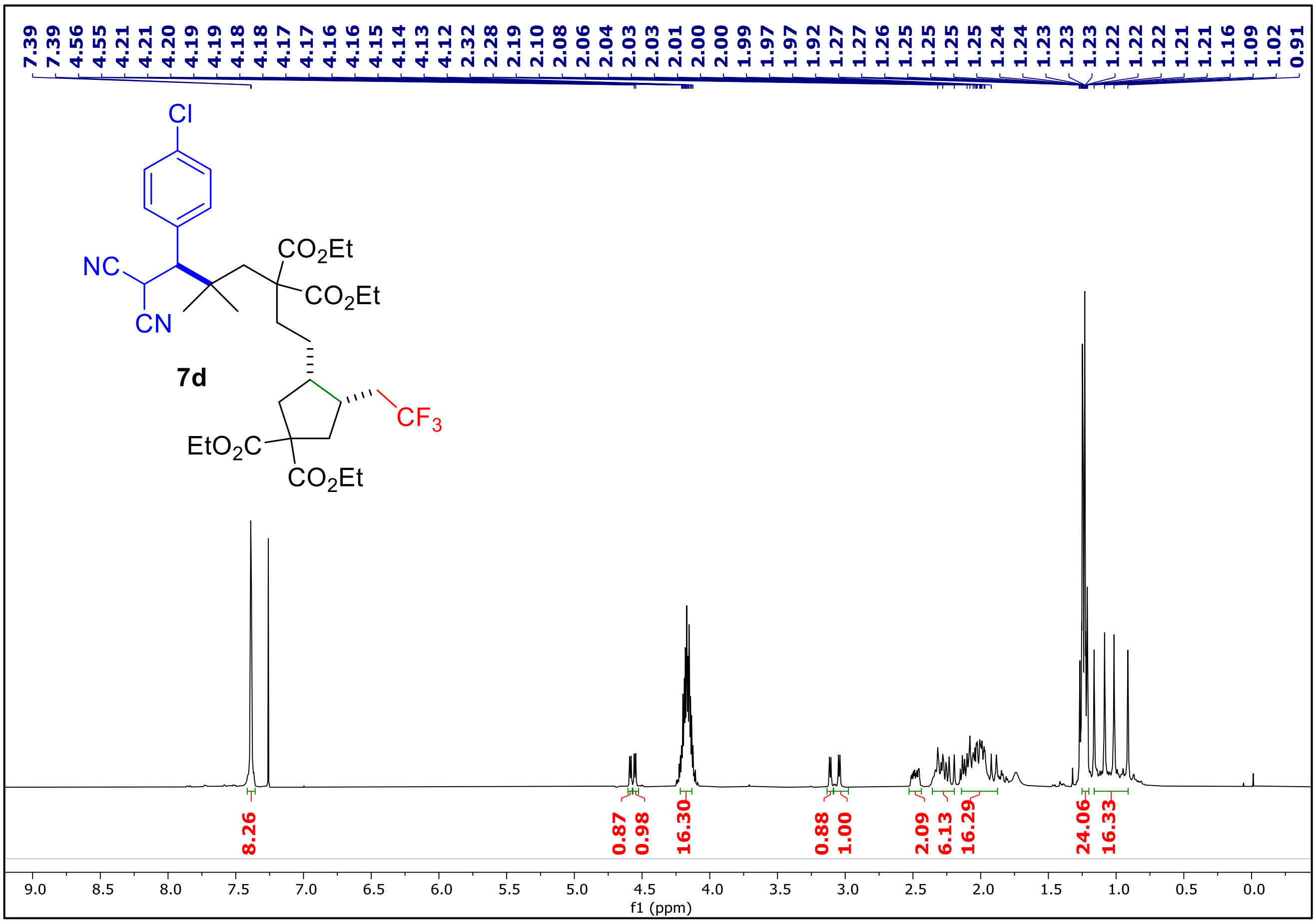

^{1}H NMR of compound **7d** (400 MHz, $CDCl_3$)

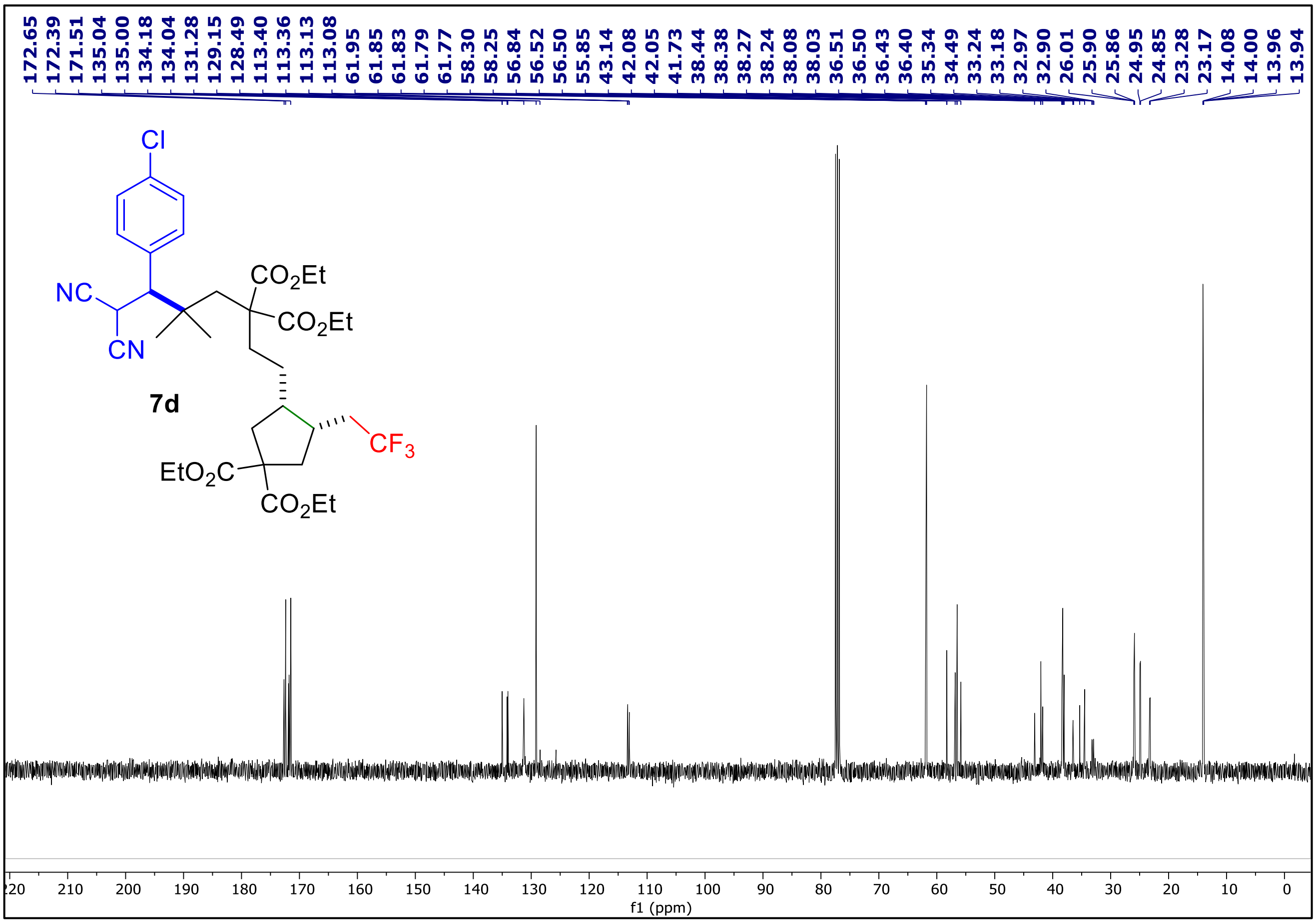

$^{13}C\{^{1}H\}$ NMR of compound **7d** (101 MHz, $CDCl_3$)

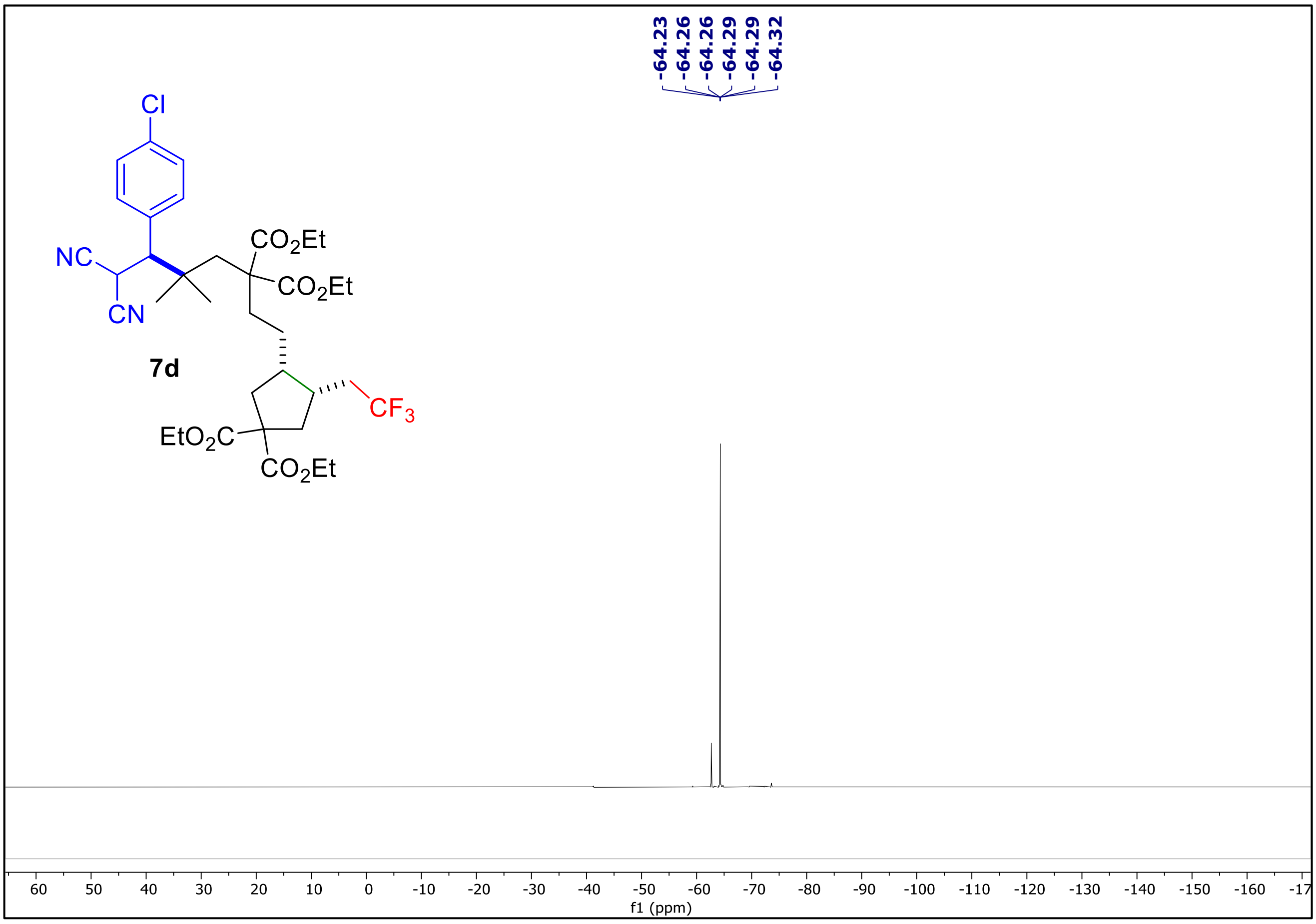

^{19}F NMR of compound **7d** (376 MHz, $CDCl_3$)

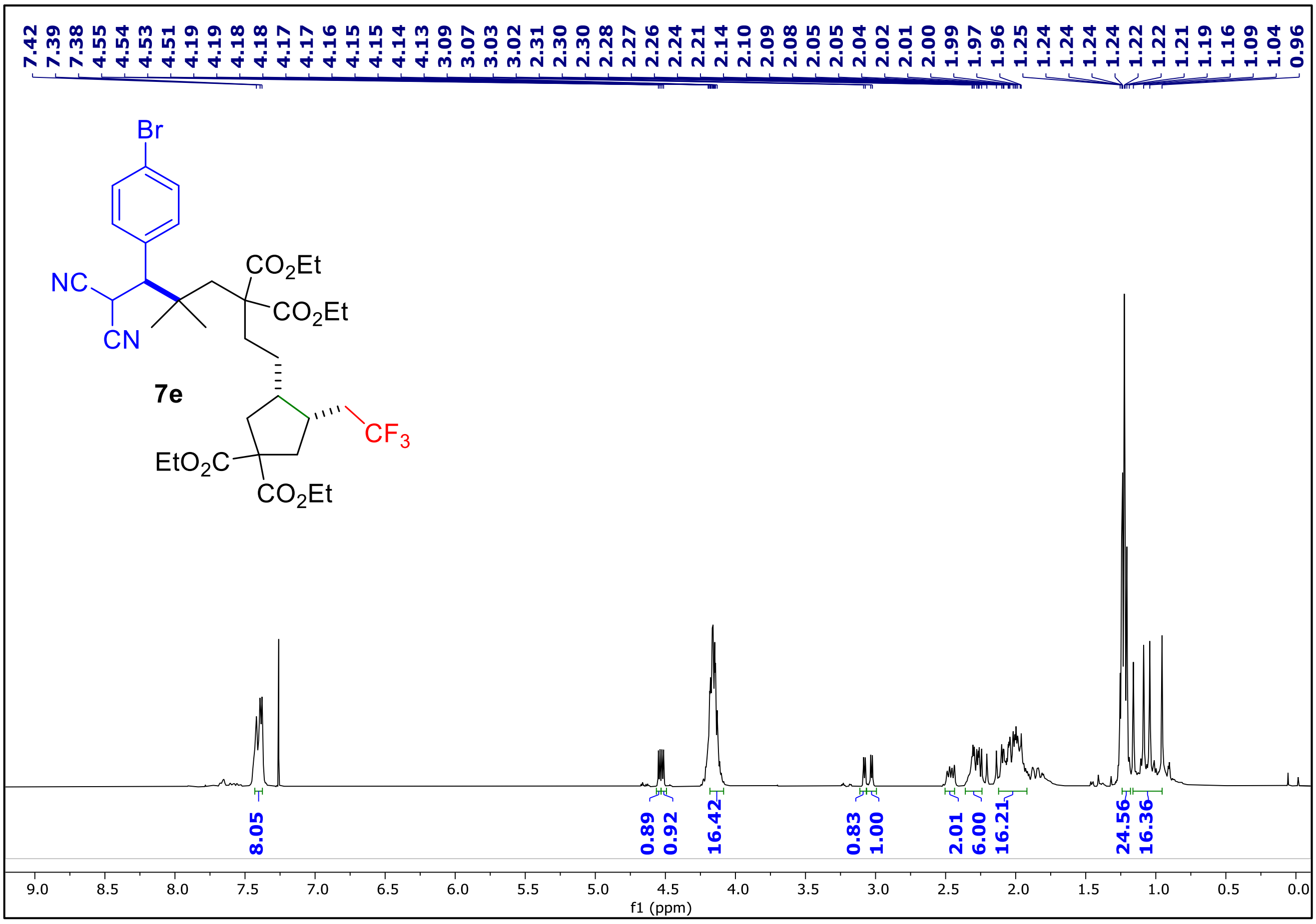

^{1}H NMR of compound **7e** (400 MHz, $CDCl_3$)

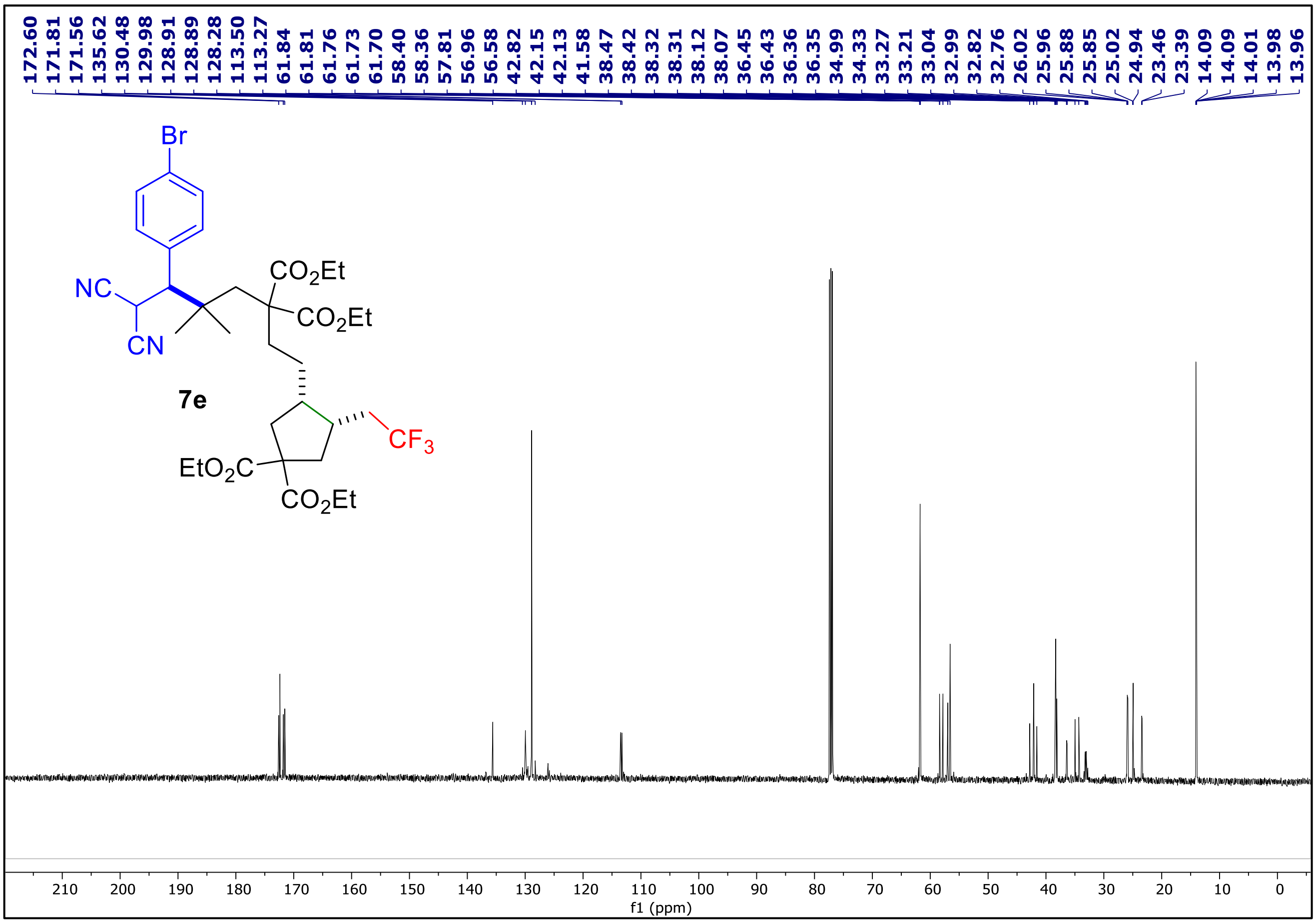

$^{13}C\{^{1}H\}$ NMR of compound **7e** (126 MHz, $CDCl_3$)

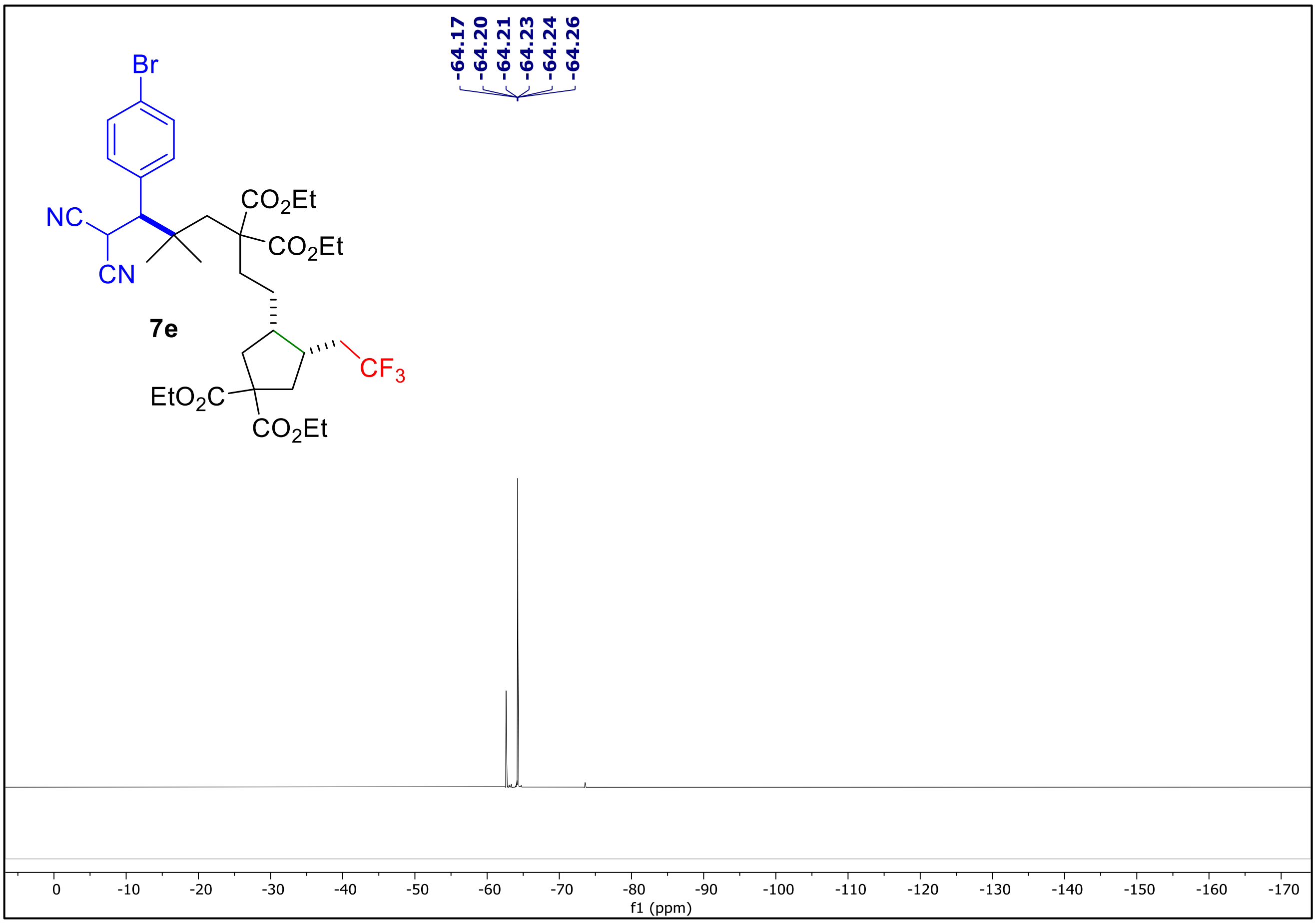

^{19}F NMR of compound **7e** (376 MHz, $CDCl_3$)

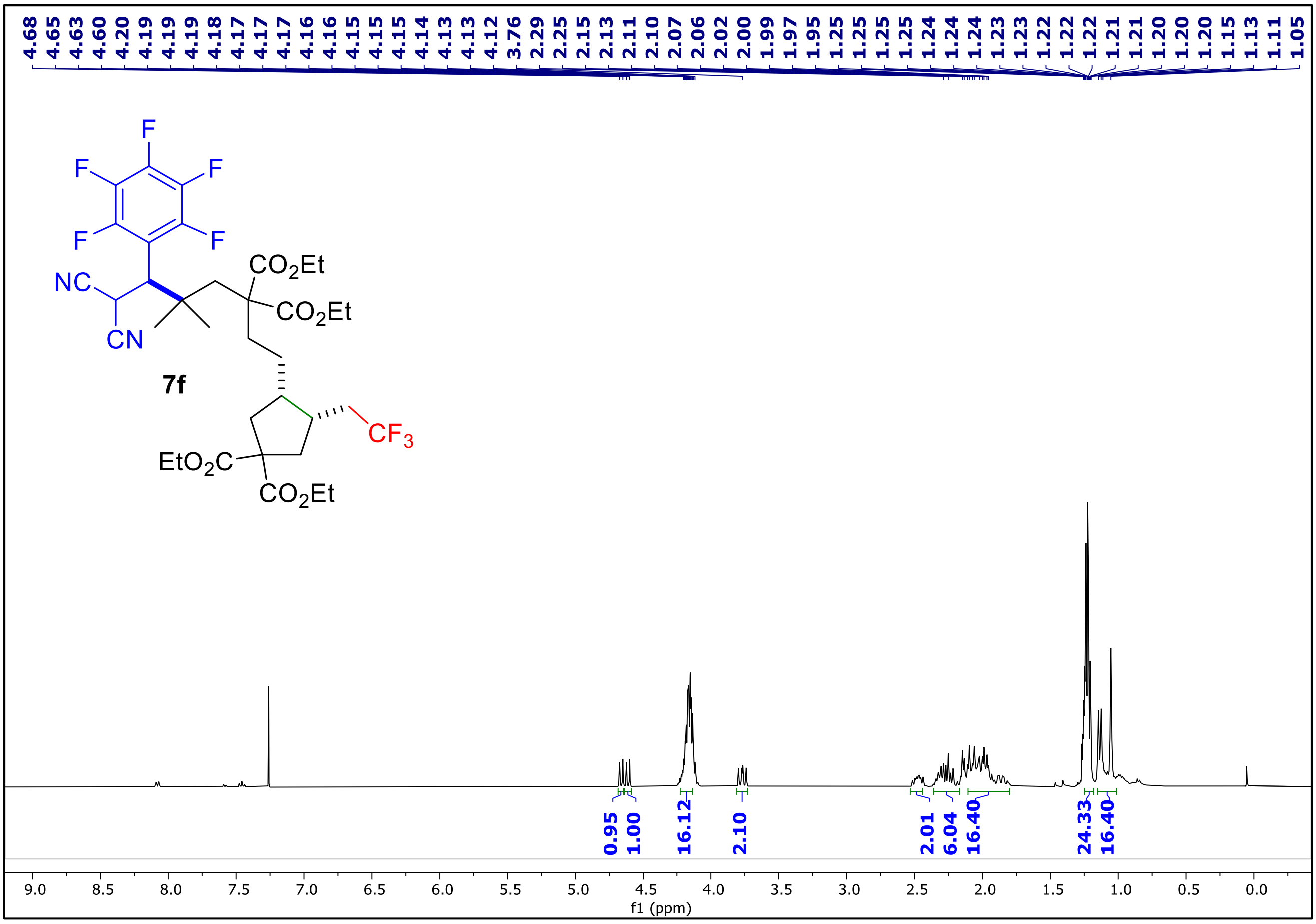

^{1}H NMR of compound **7f** (400 MHz, $CDCl_3$)

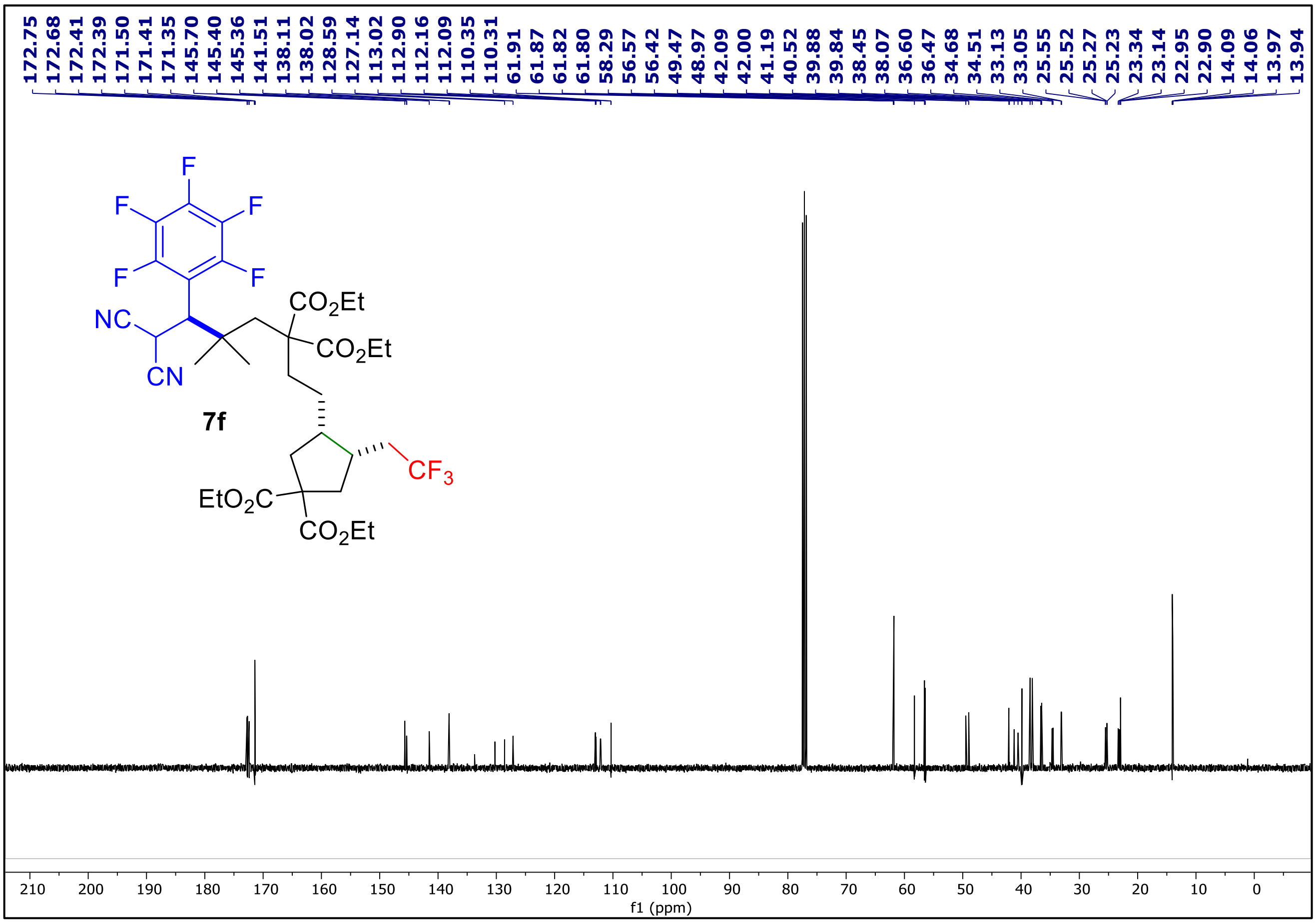

^{13}C{^{1}H} NMR of compound **7f** (101 MHz, $CDCl_3$)

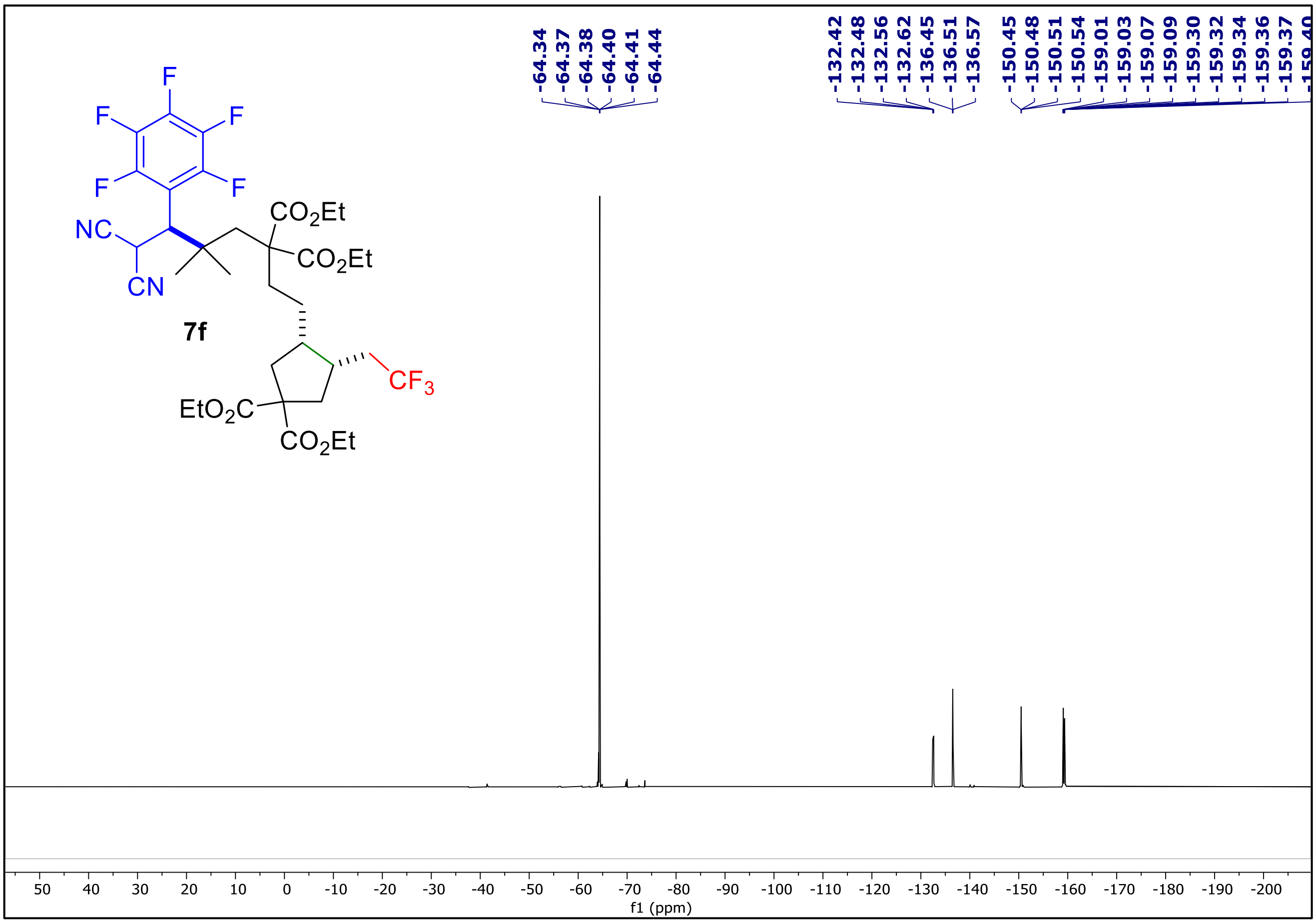

^{19}F NMR of compound **7f** (376 MHz, $CDCl_3$)

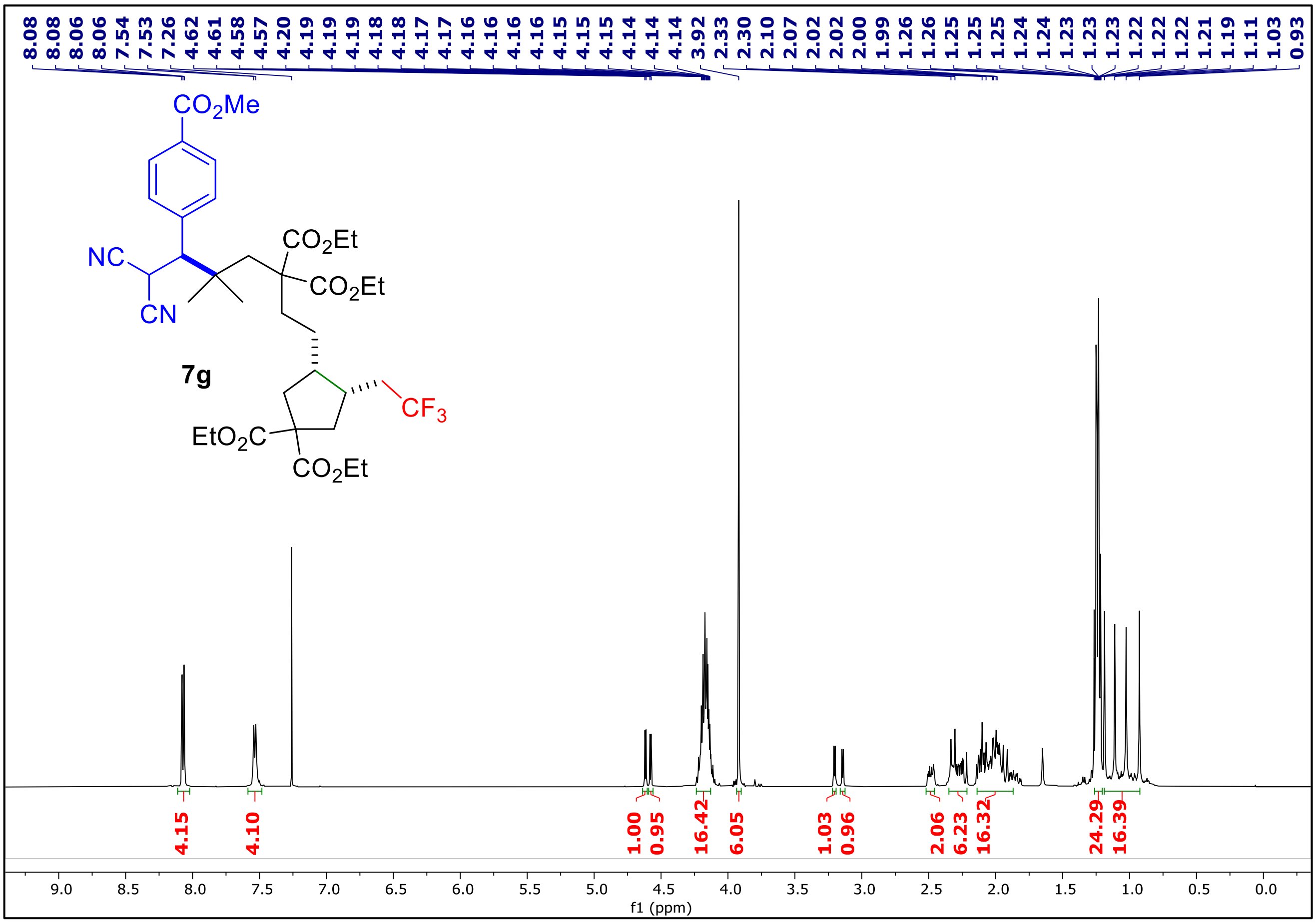

^{1}H NMR of compound **7g** (500 MHz, $CDCl_3$)

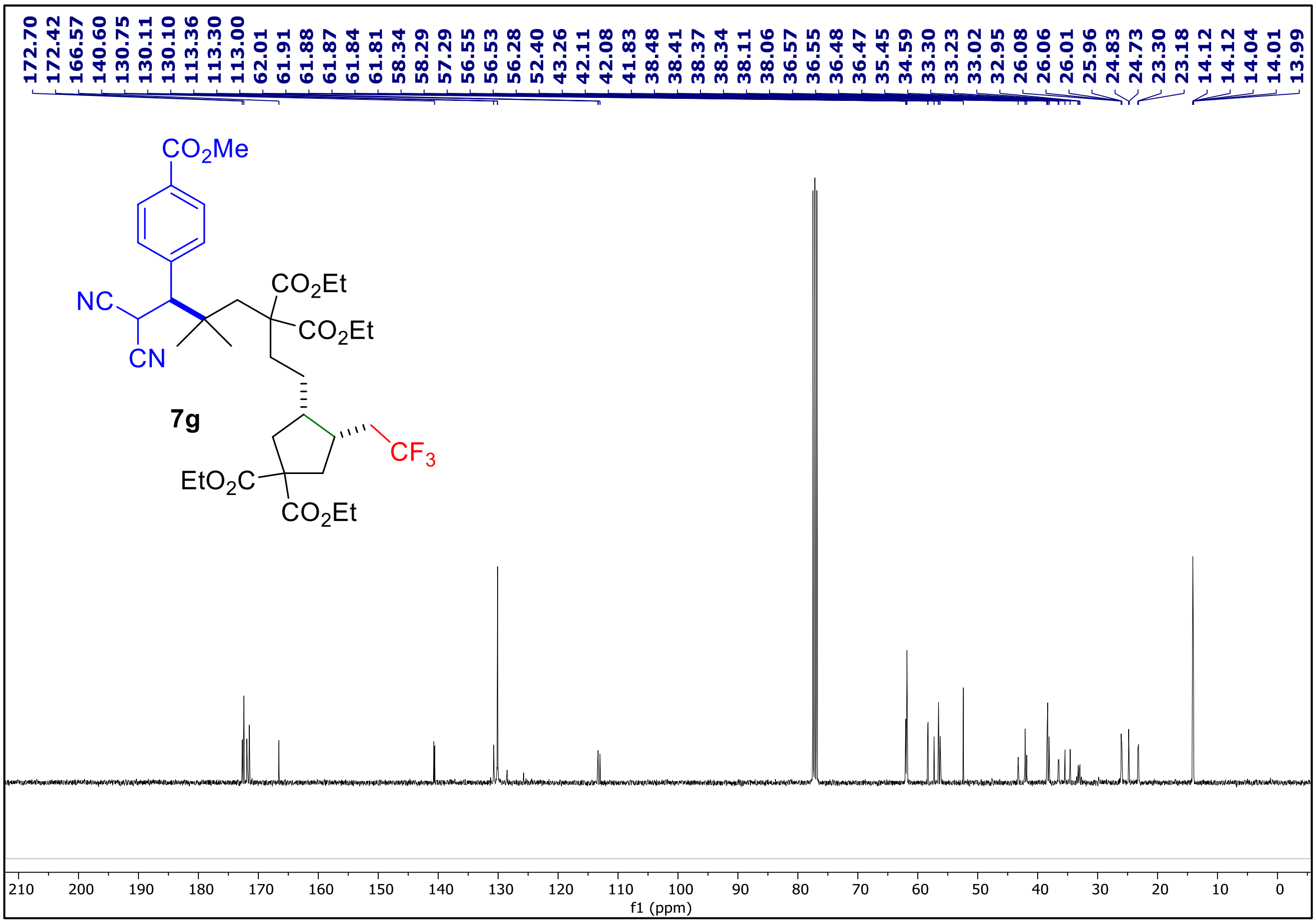

$^{13}C\{^1H\}$ NMR of compound **7g** (101 MHz, $CDCl_3$)

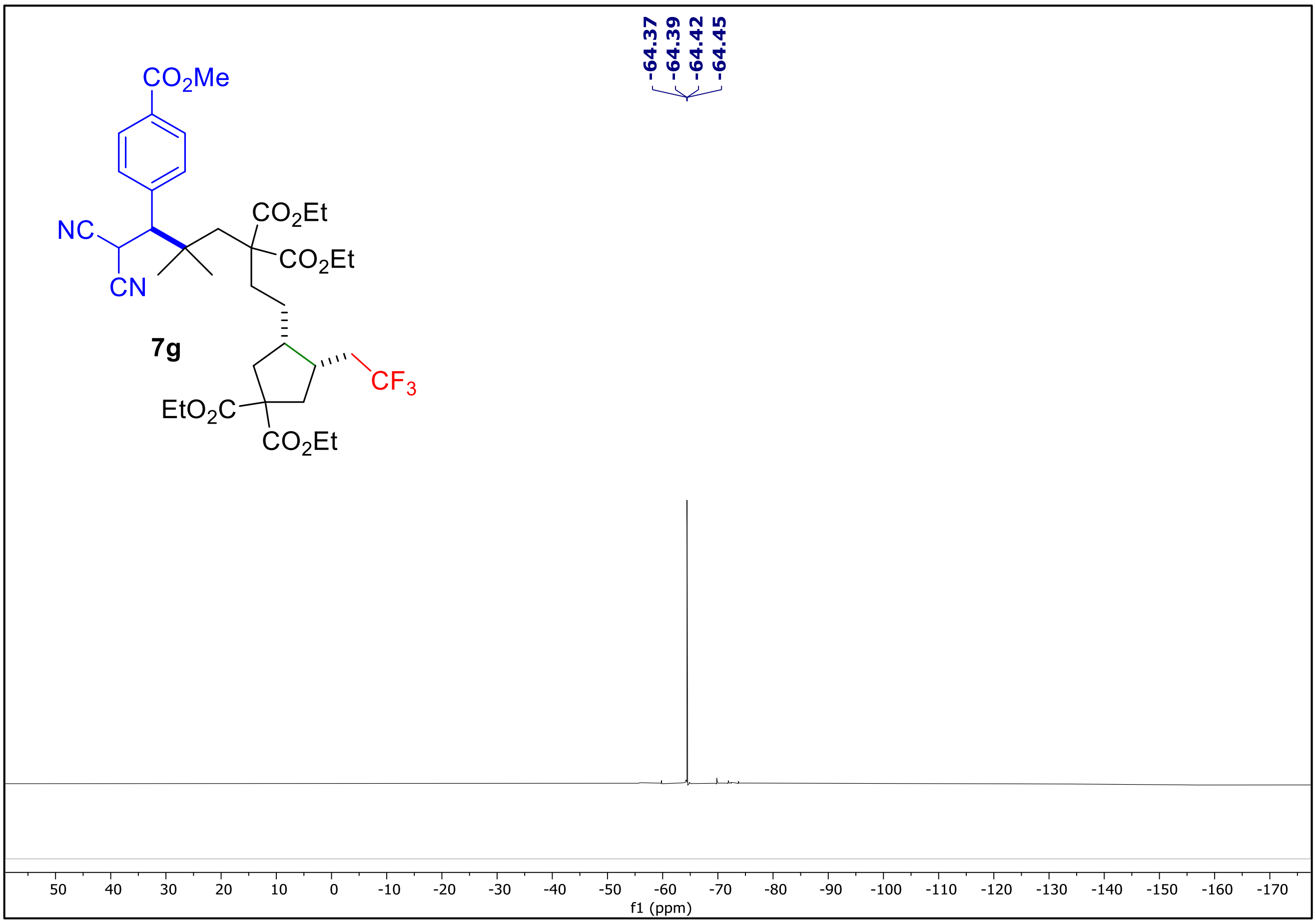

^{19}F NMR of compound **7g** (471 MHz, $CDCl_3$)

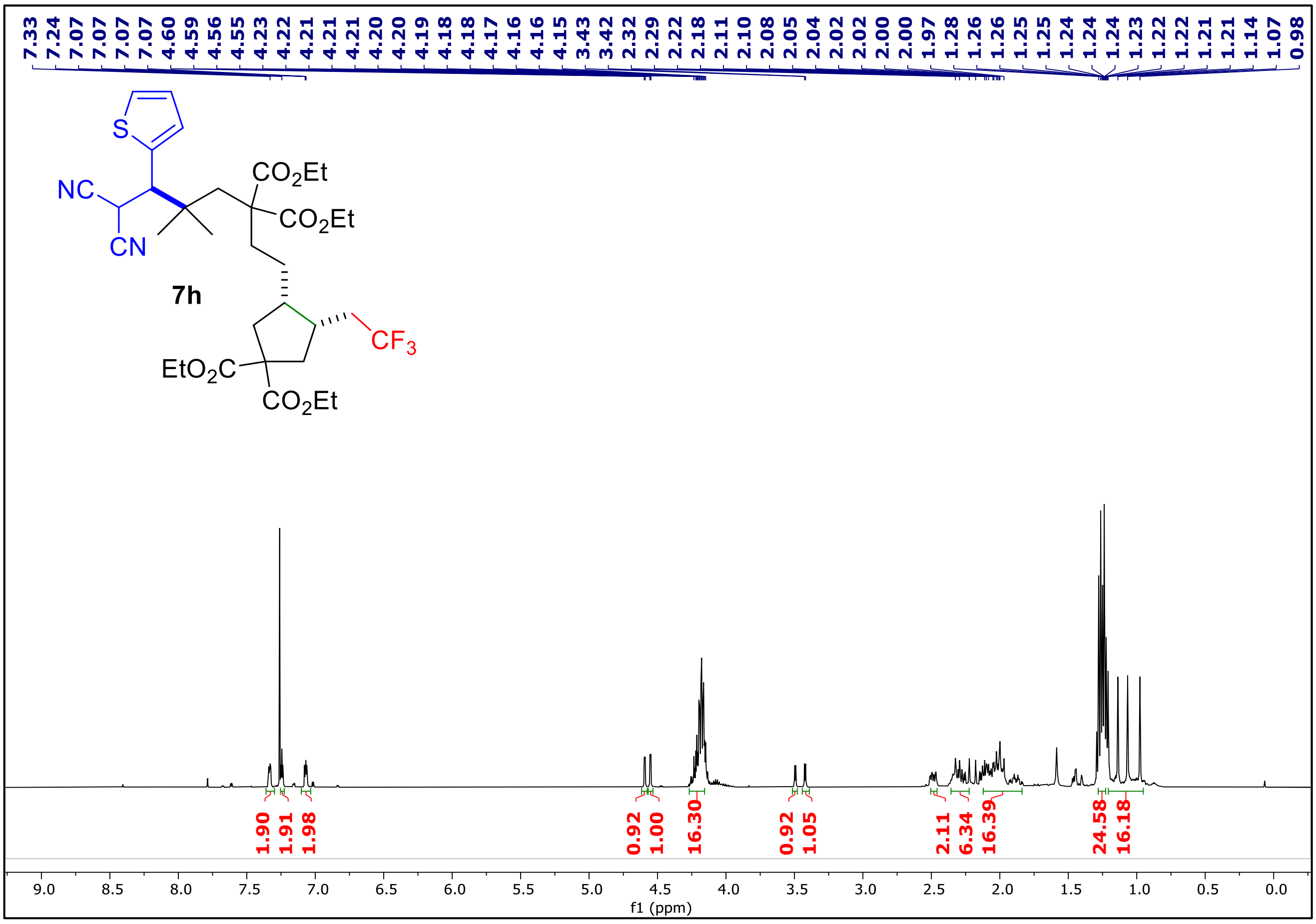

^{1}H NMR of compound **7h** (500 MHz, $CDCl_3$)

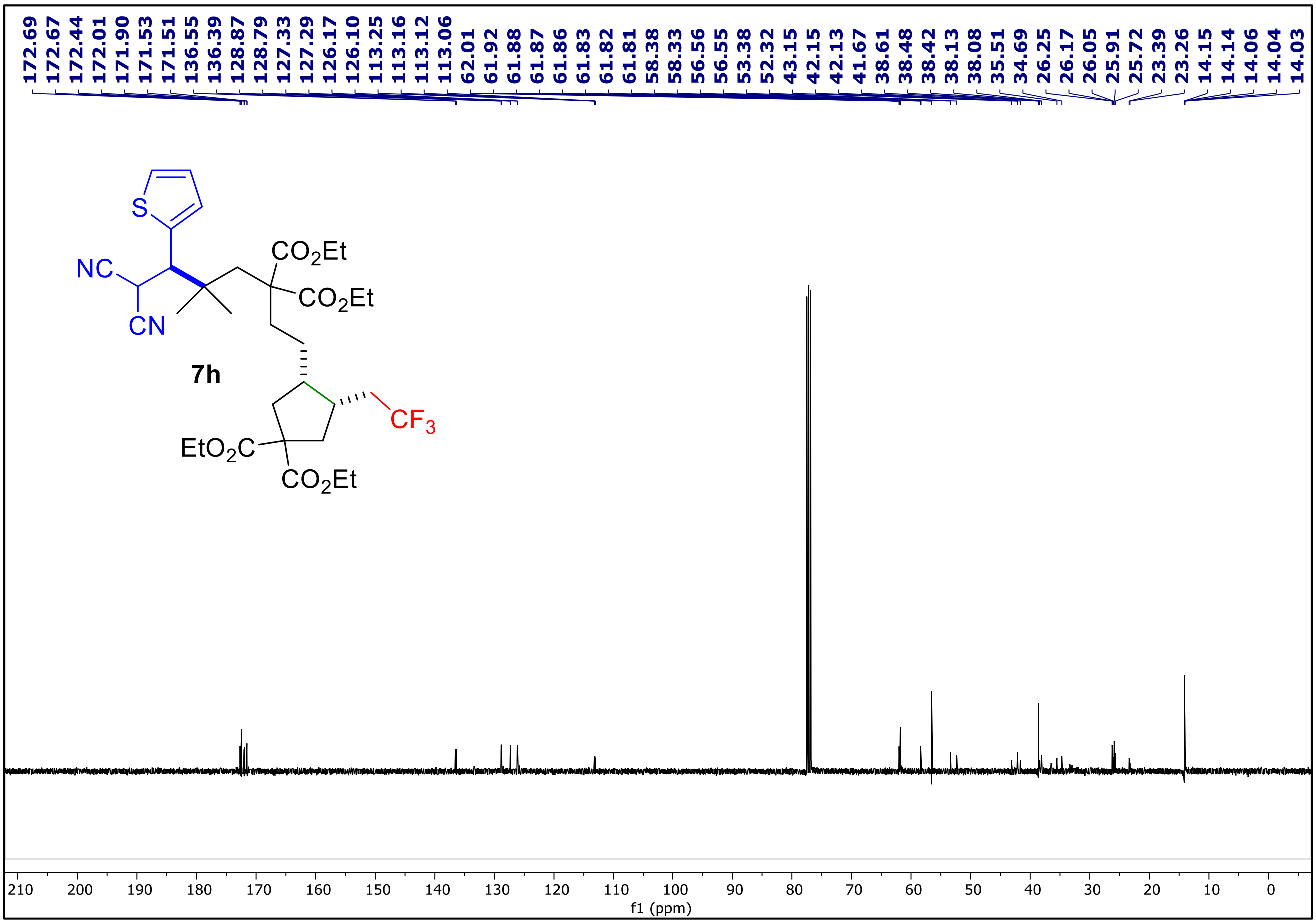

$^{13}C\{^1H\}$ NMR of compound **7h** (101 MHz, $CDCl_3$)

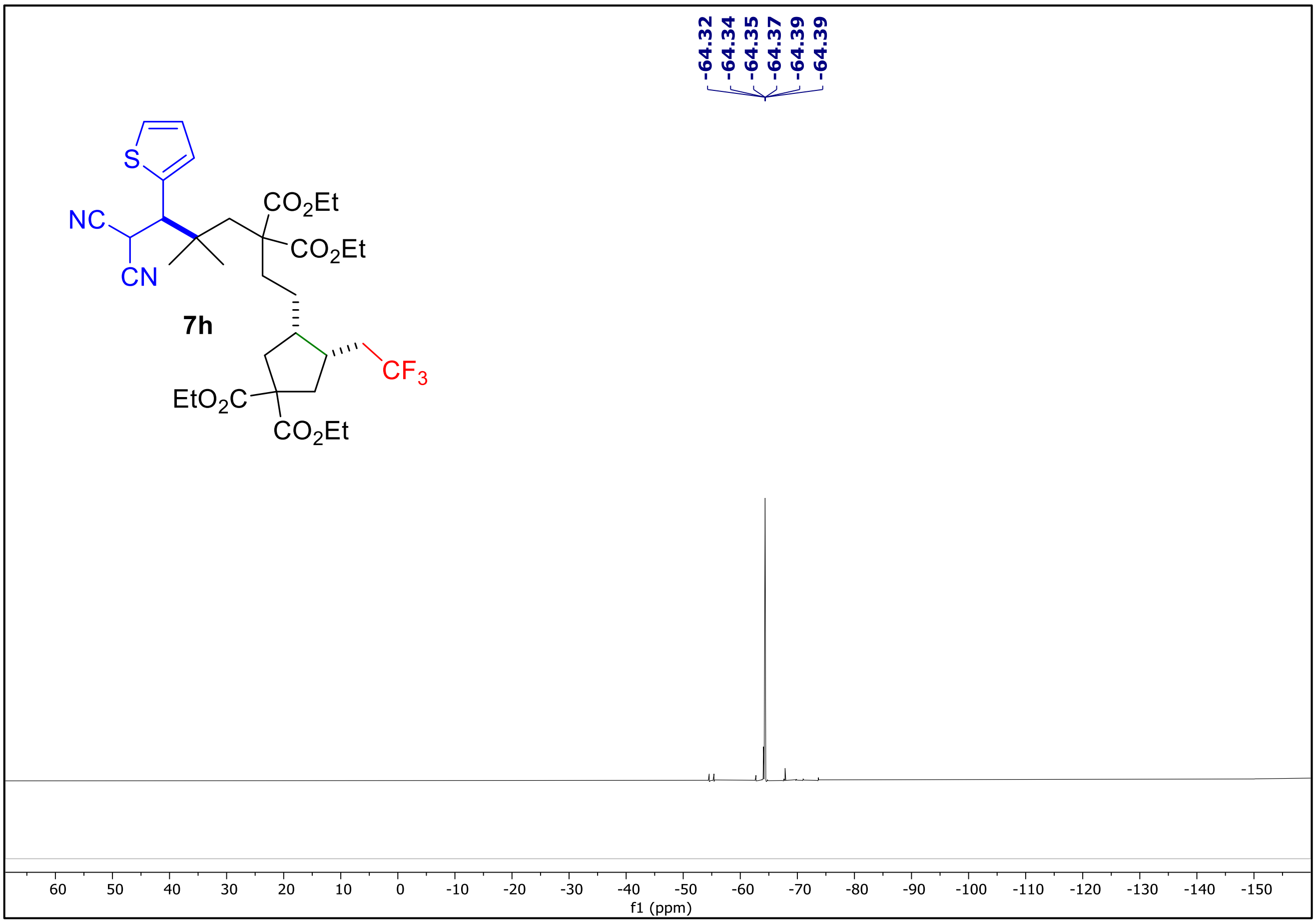

^{19}F NMR of compound **7h** (471 MHz, $CDCl_3$)

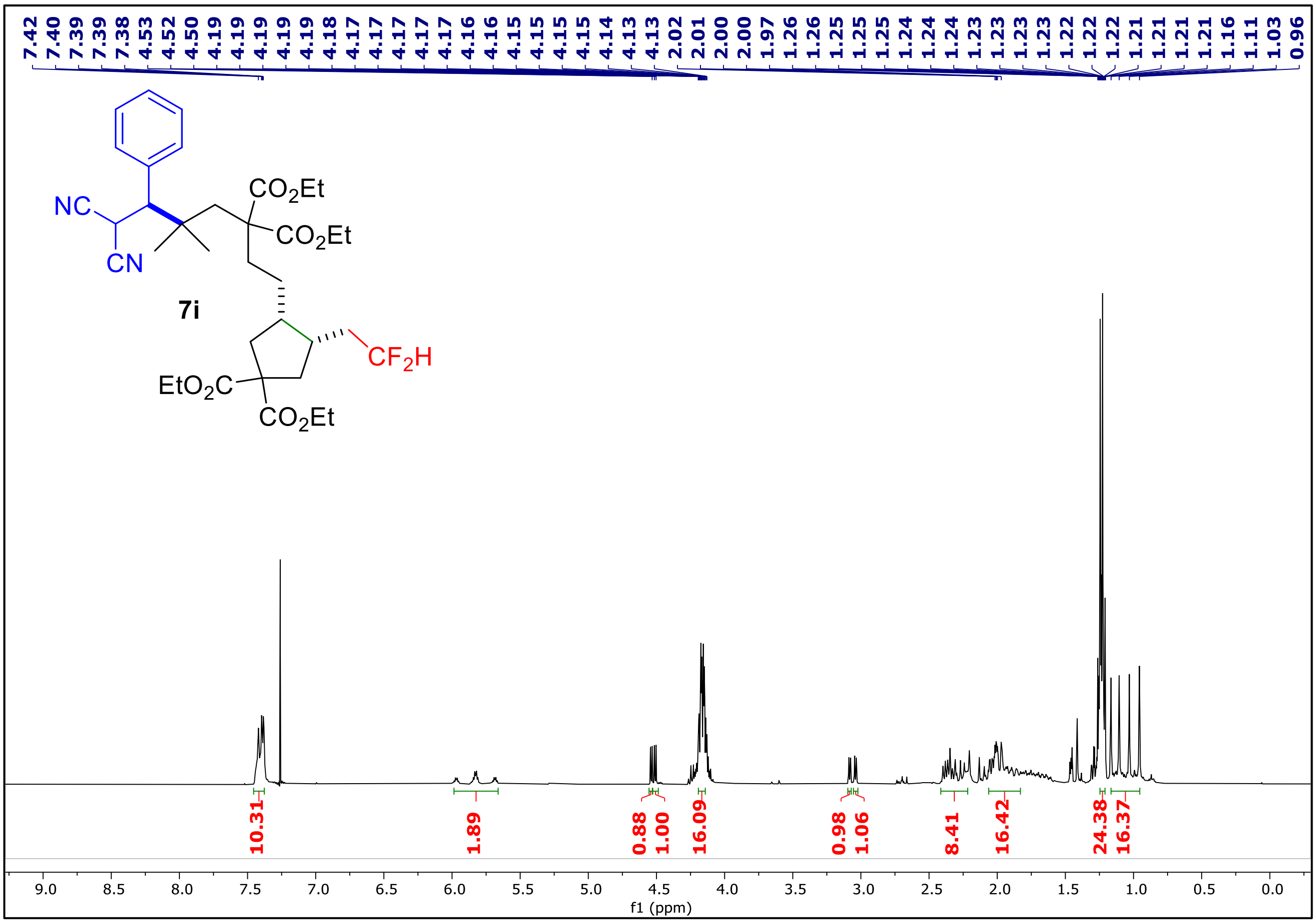

^{1}H NMR of compound **7i** (400 MHz, $CDCl_3$)

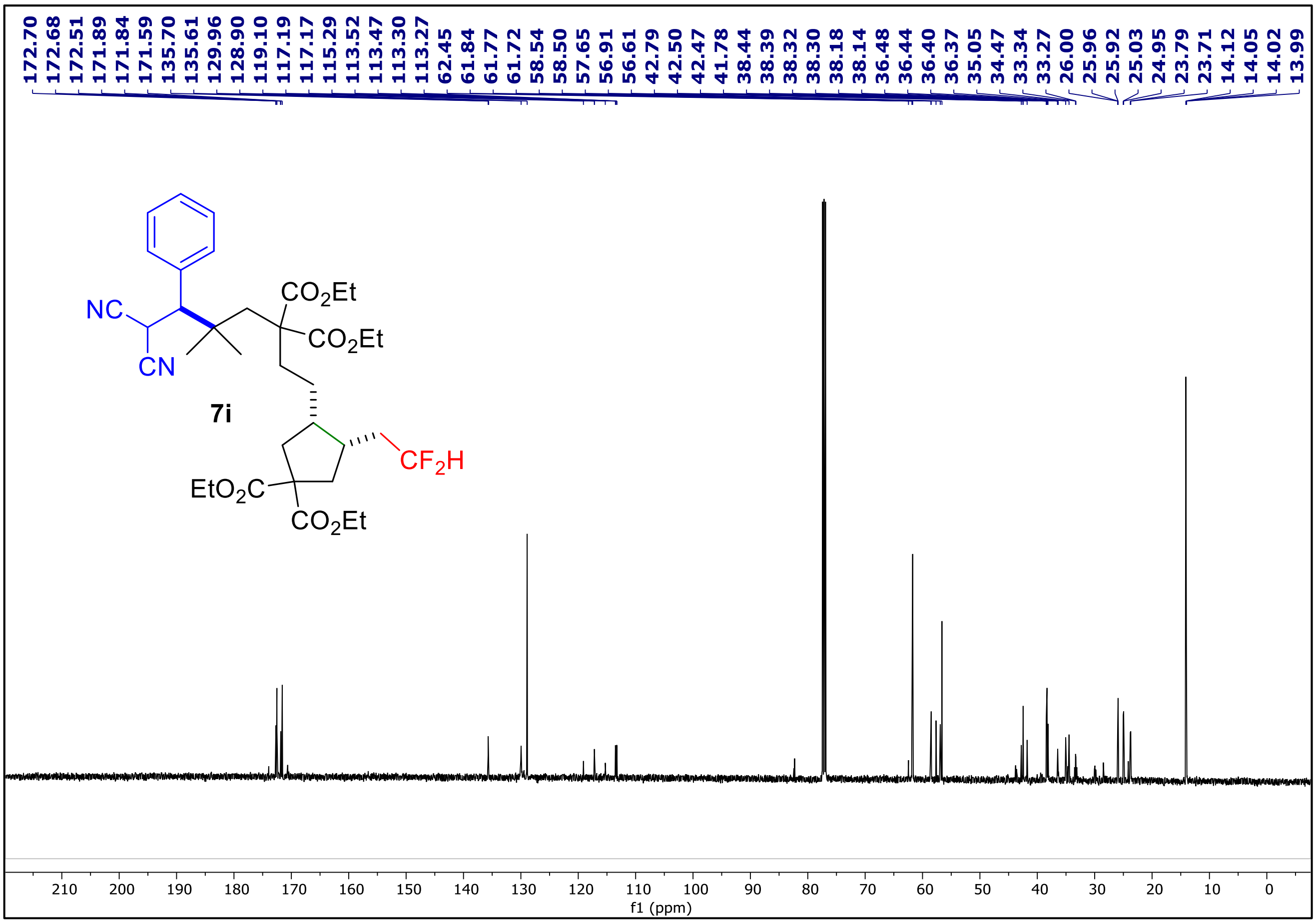

$^{13}C\{^{1}H\}$ NMR of compound **7i** (126 MHz, $CDCl_3$)

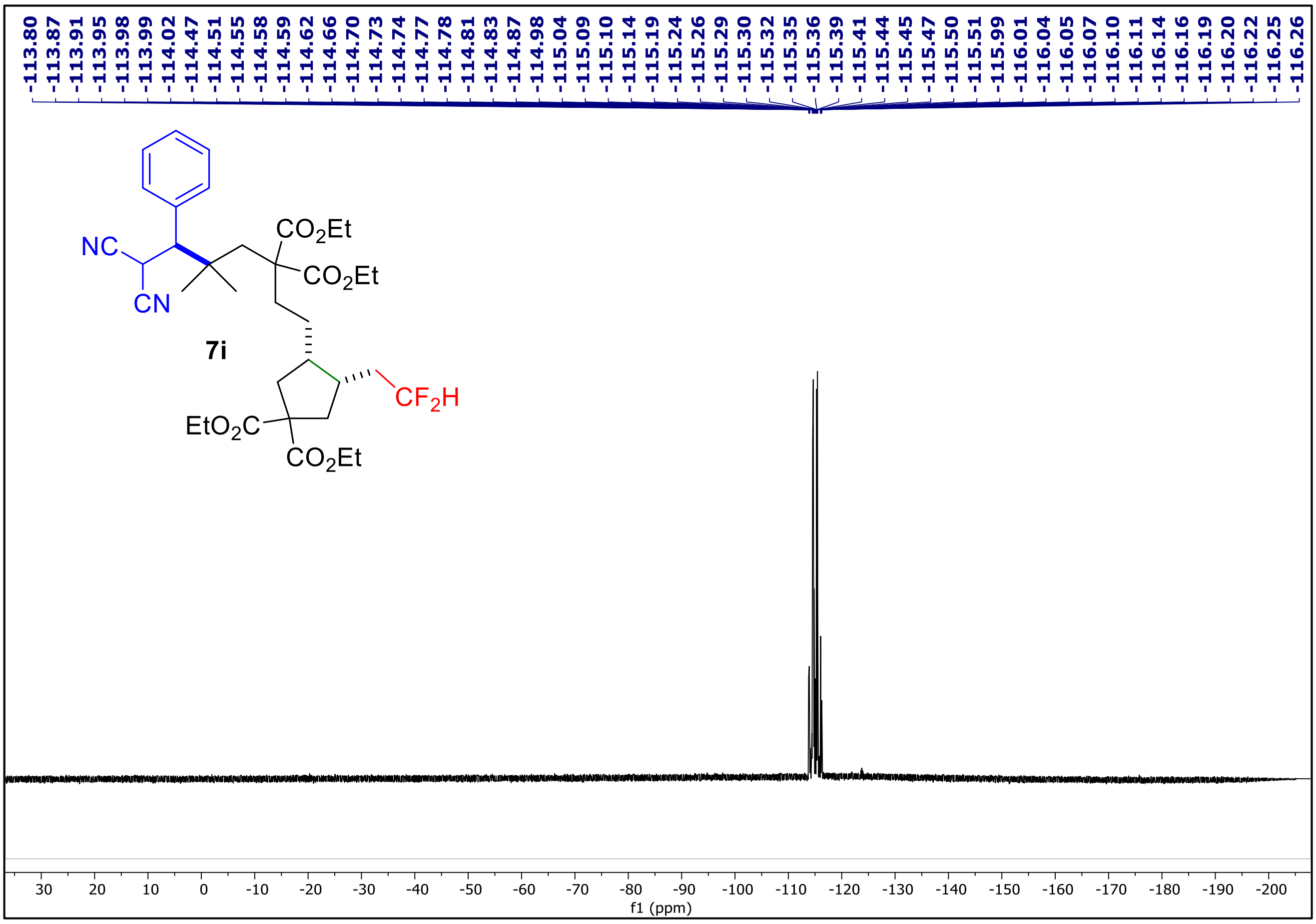

^{19}F NMR of compound **7i** (471 MHz, $CDCl_3$)

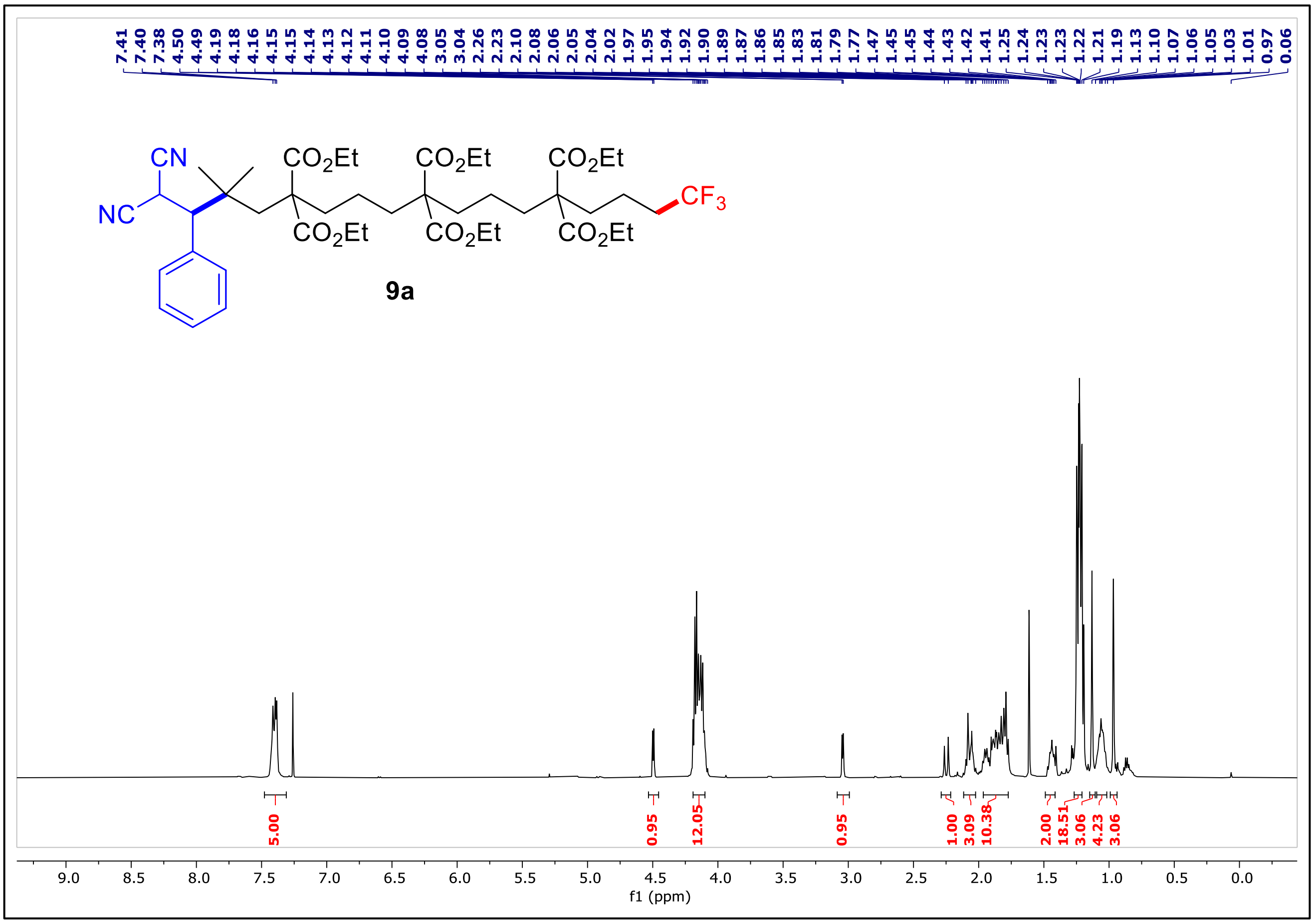

^{1}H NMR of compound **9a** (500 MHz, CDCl$_3$)

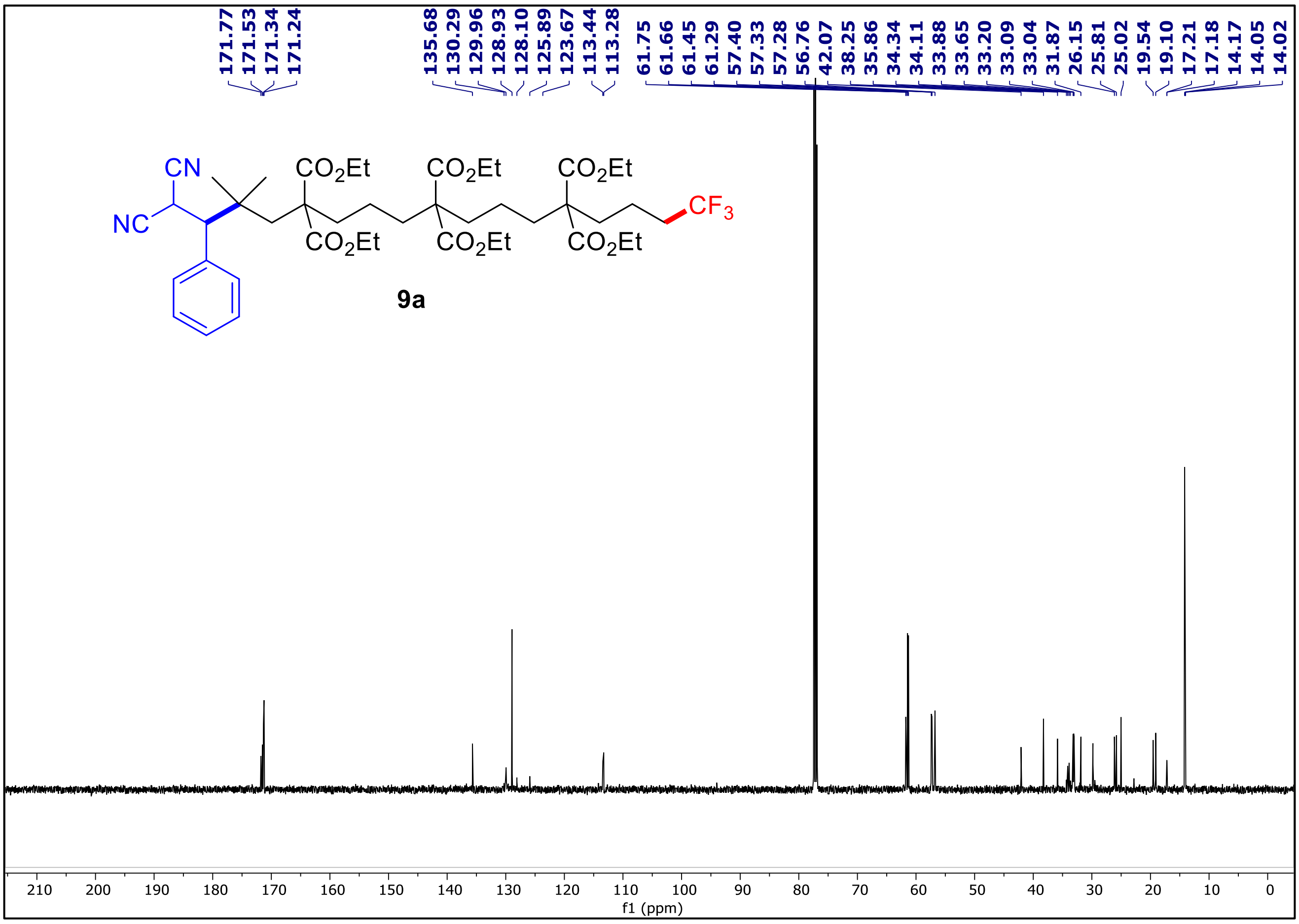

^{13}C{^{1}H} NMR of compound **9a** (126 MHz, CDCl$_3$)

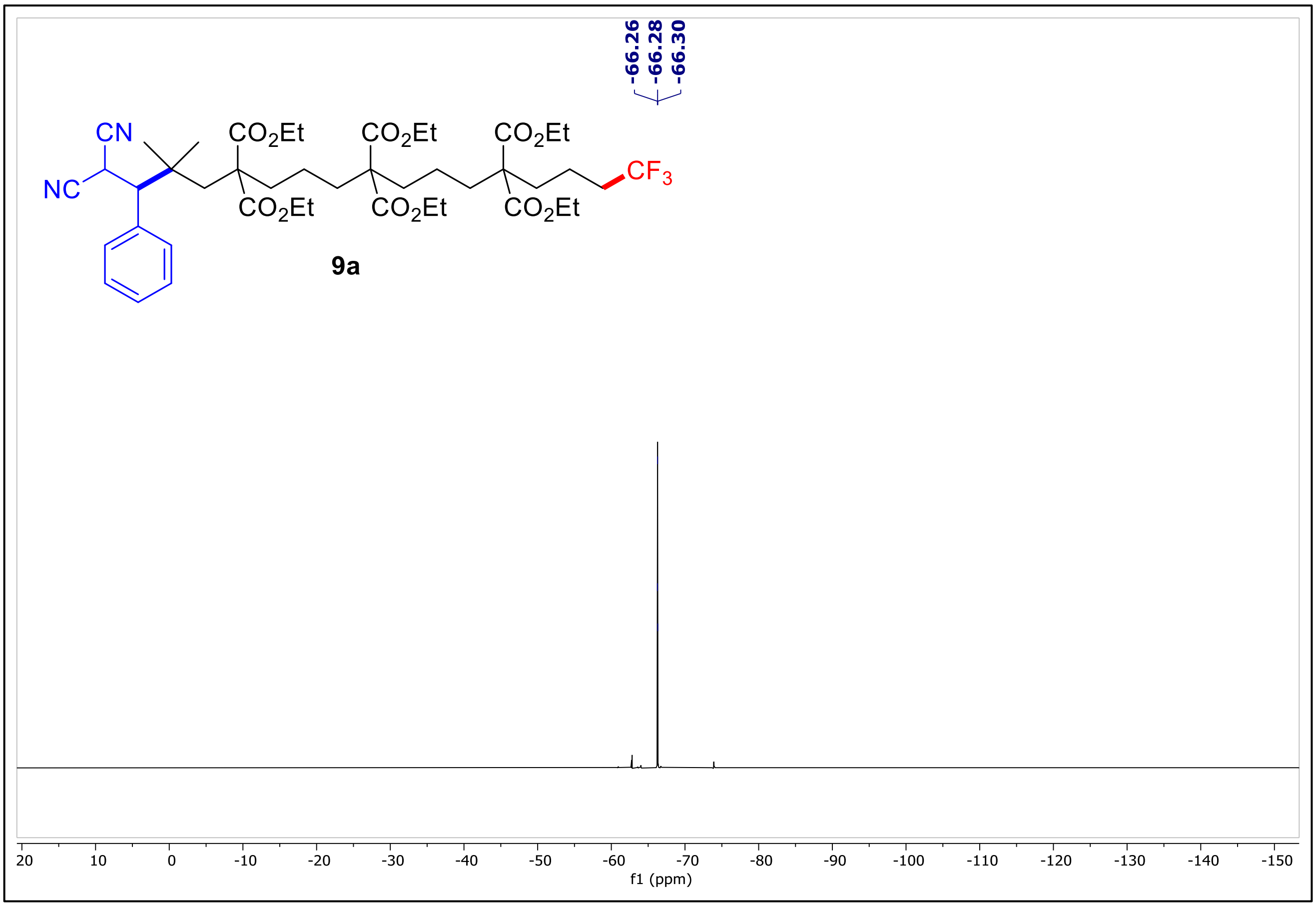

^{19}F NMR of compound 9**a** (471 MHz, $CDCl_3$)

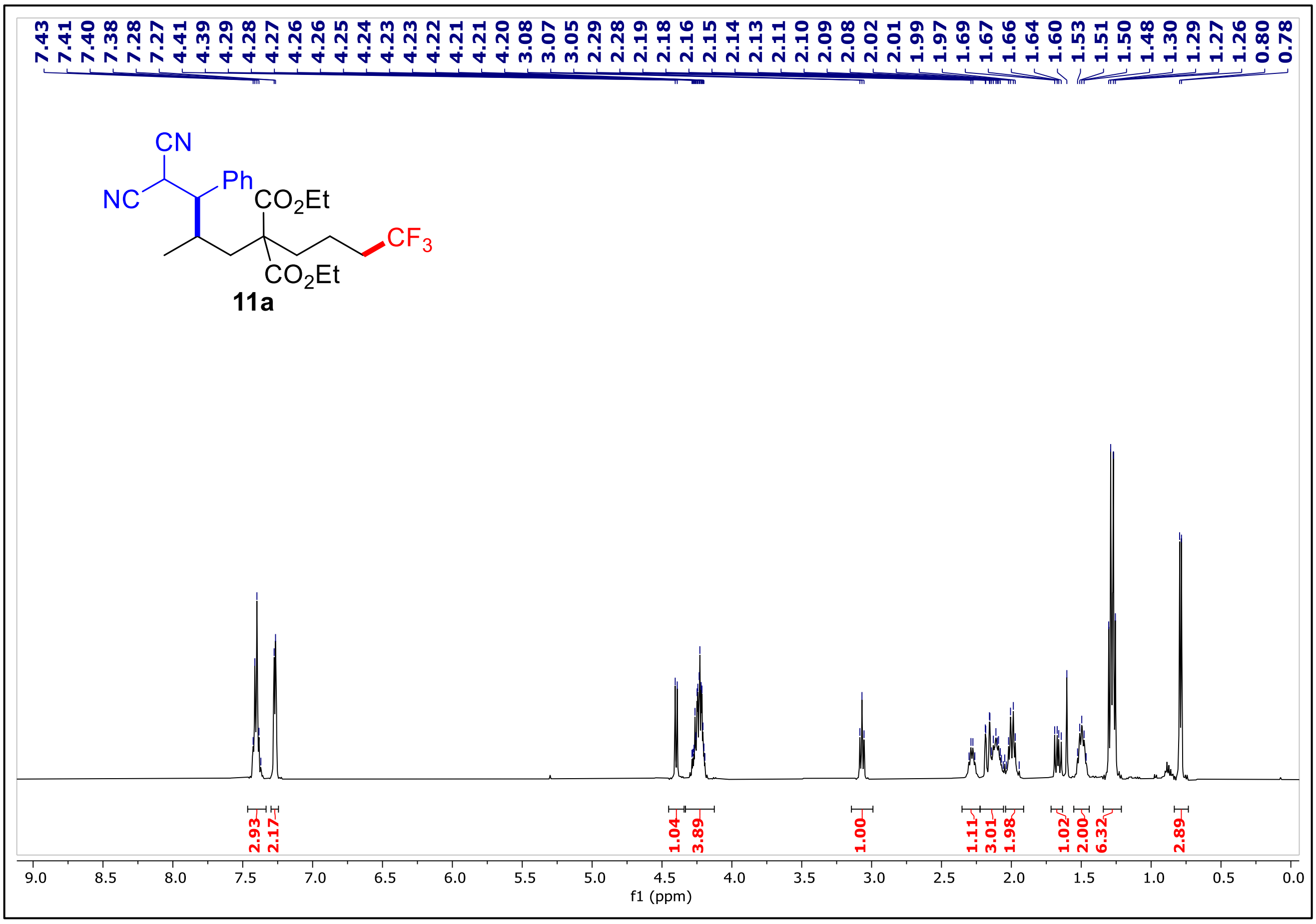

^{1}H NMR of compound **11a'** (500 MHz, $CDCl_3$)

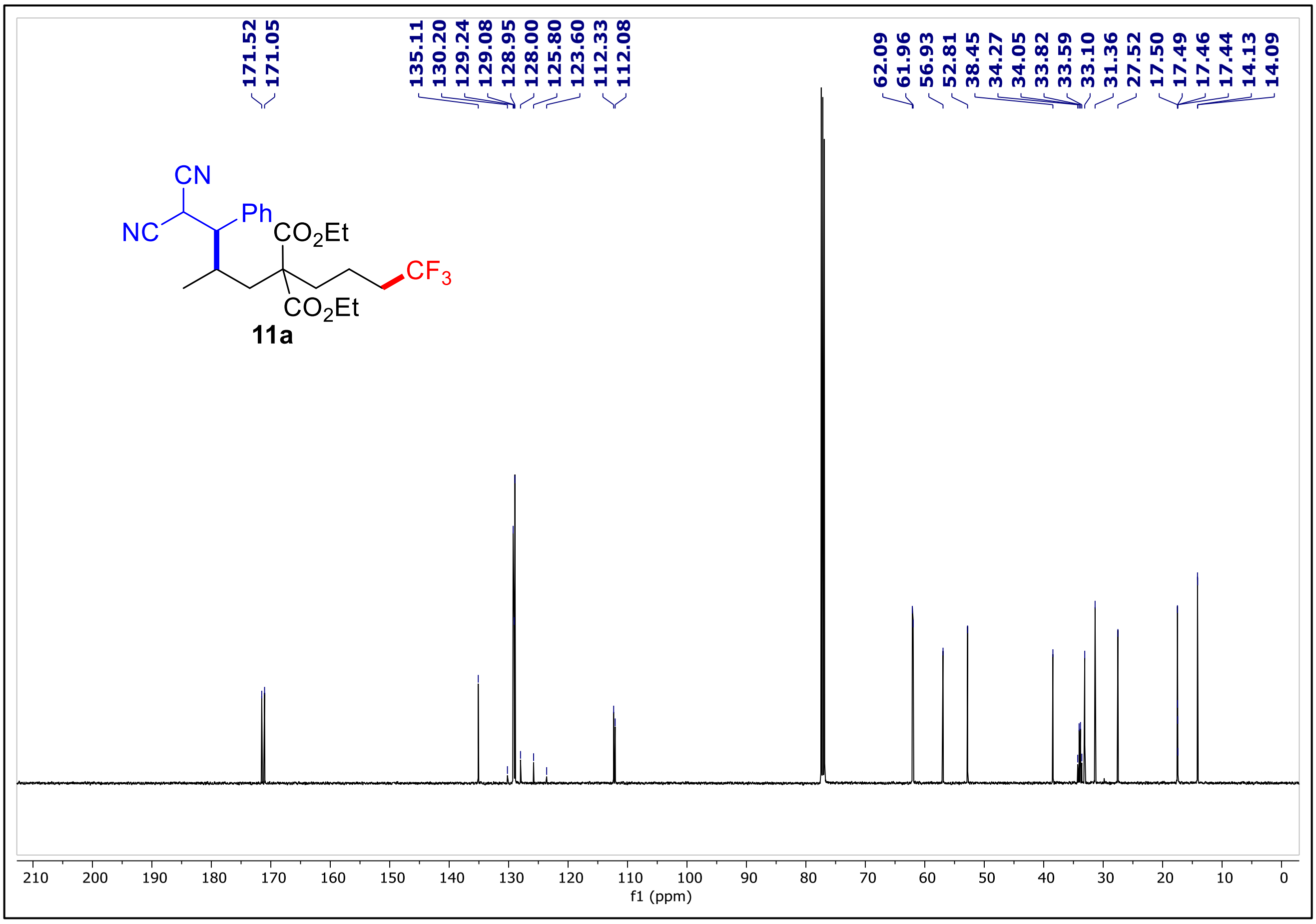

$^{13}C\{^{1}H\}$ NMR of compound **11a′** (126 MHz, $CDCl_3$)

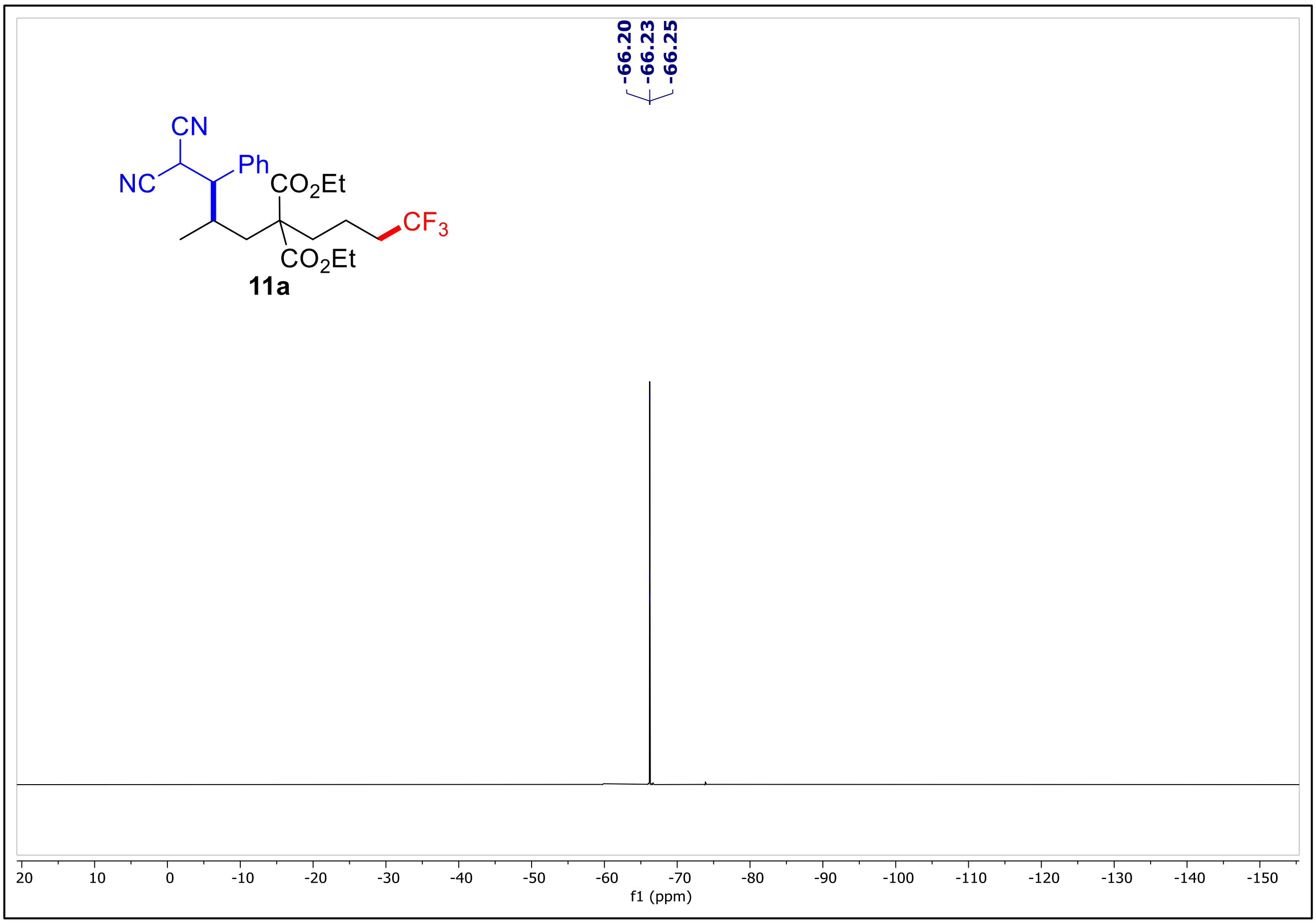

^{19}F NMR of compound **11a′** (471 MHz, $CDCl_3$)

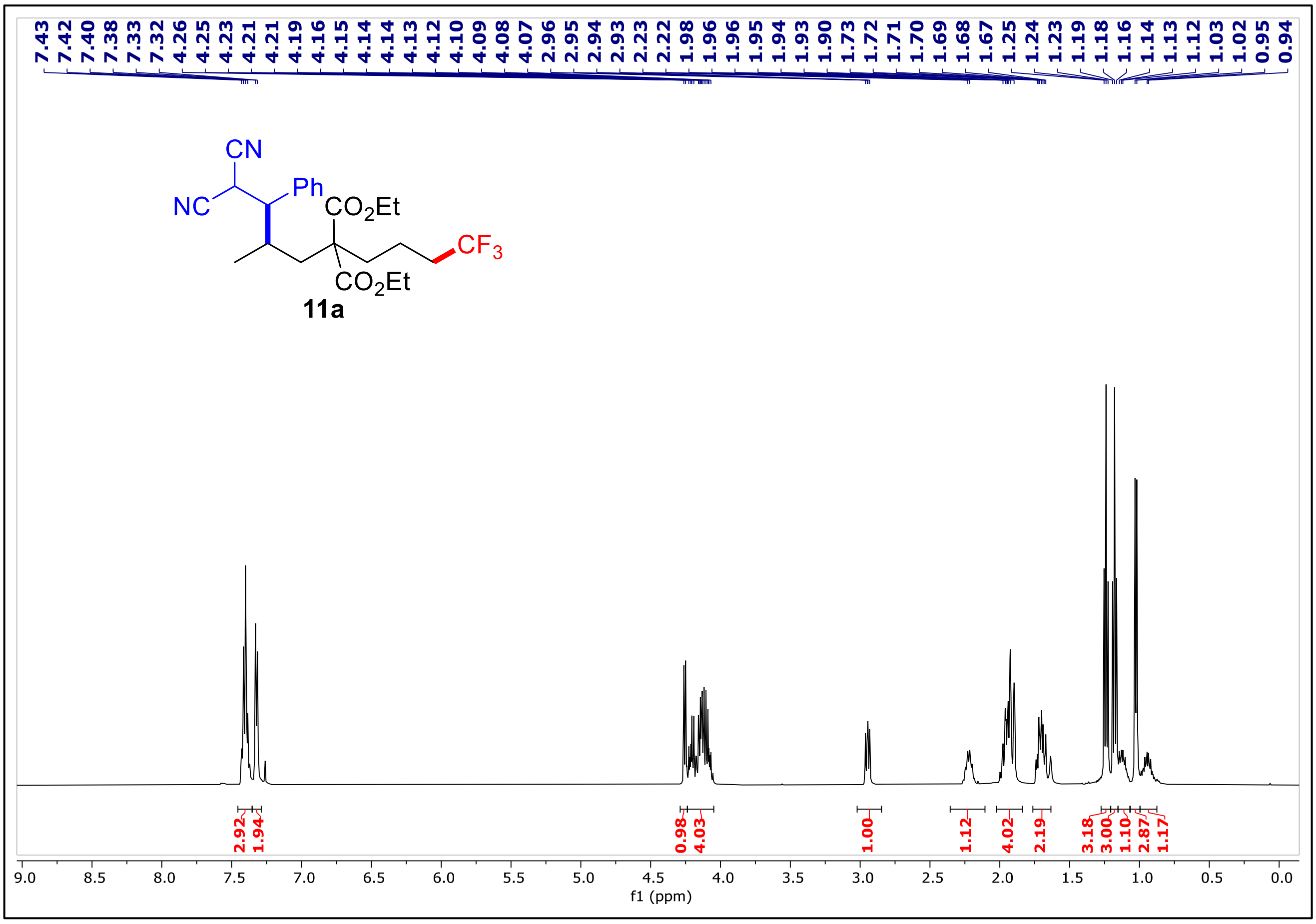

^{1}H NMR of compound **11a″** (500 MHz, $CDCl_3$)

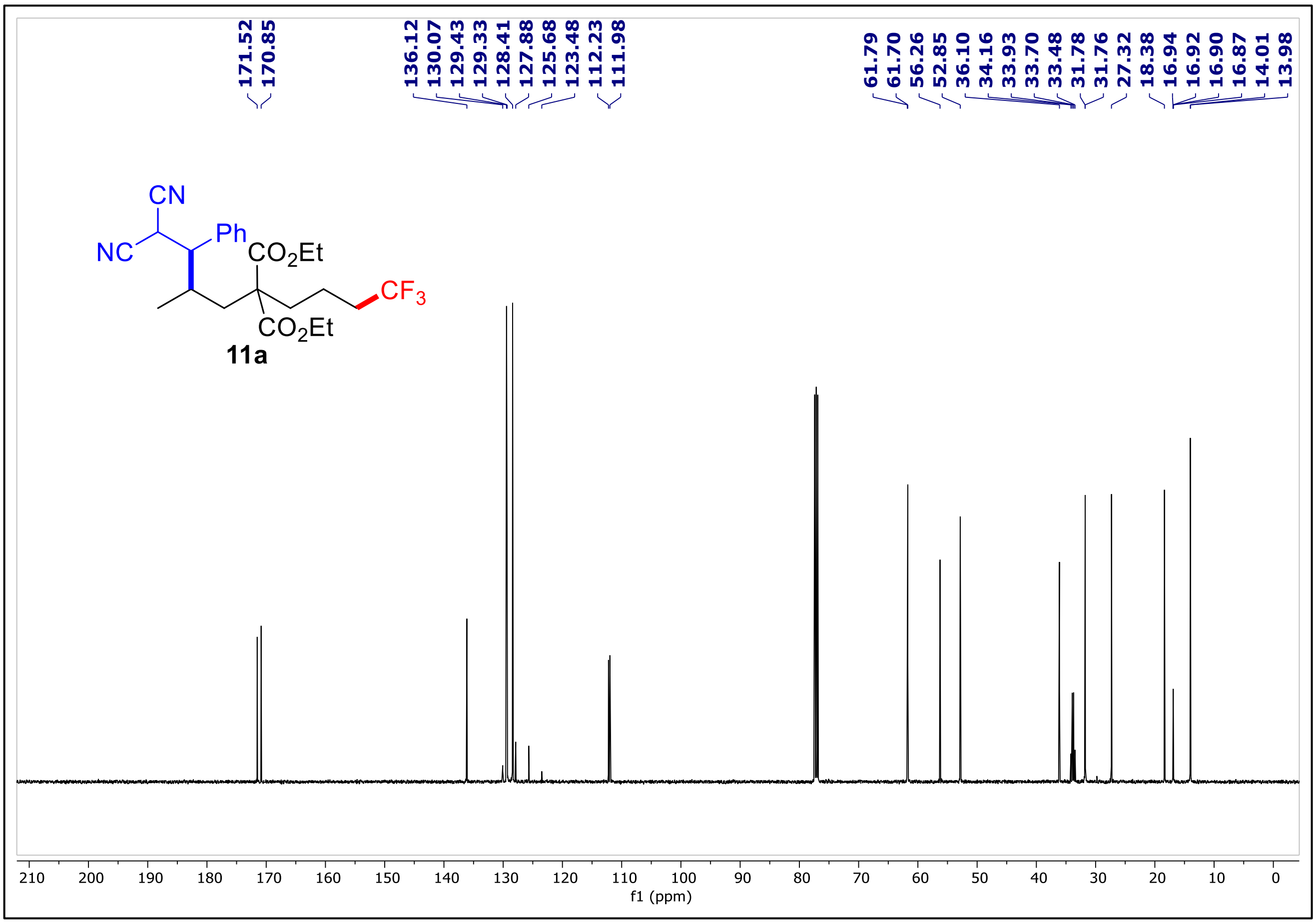

$^{13}C\{^{1}H\}$ NMR of compound **11a″** (126 MHz, $CDCl_3$)

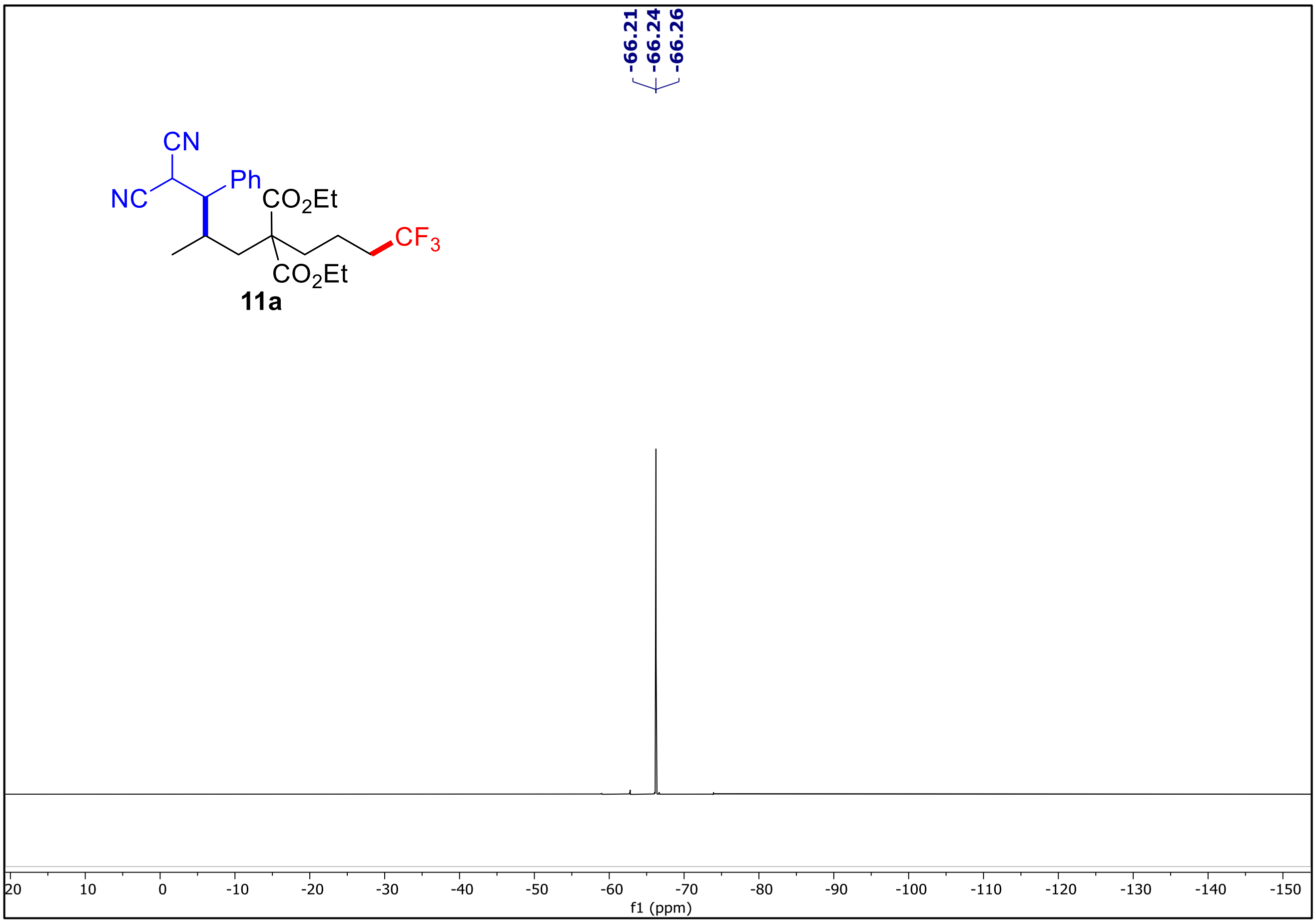

^{19}F NMR of compound **11a″** (471 MHz, $CDCl_3$)

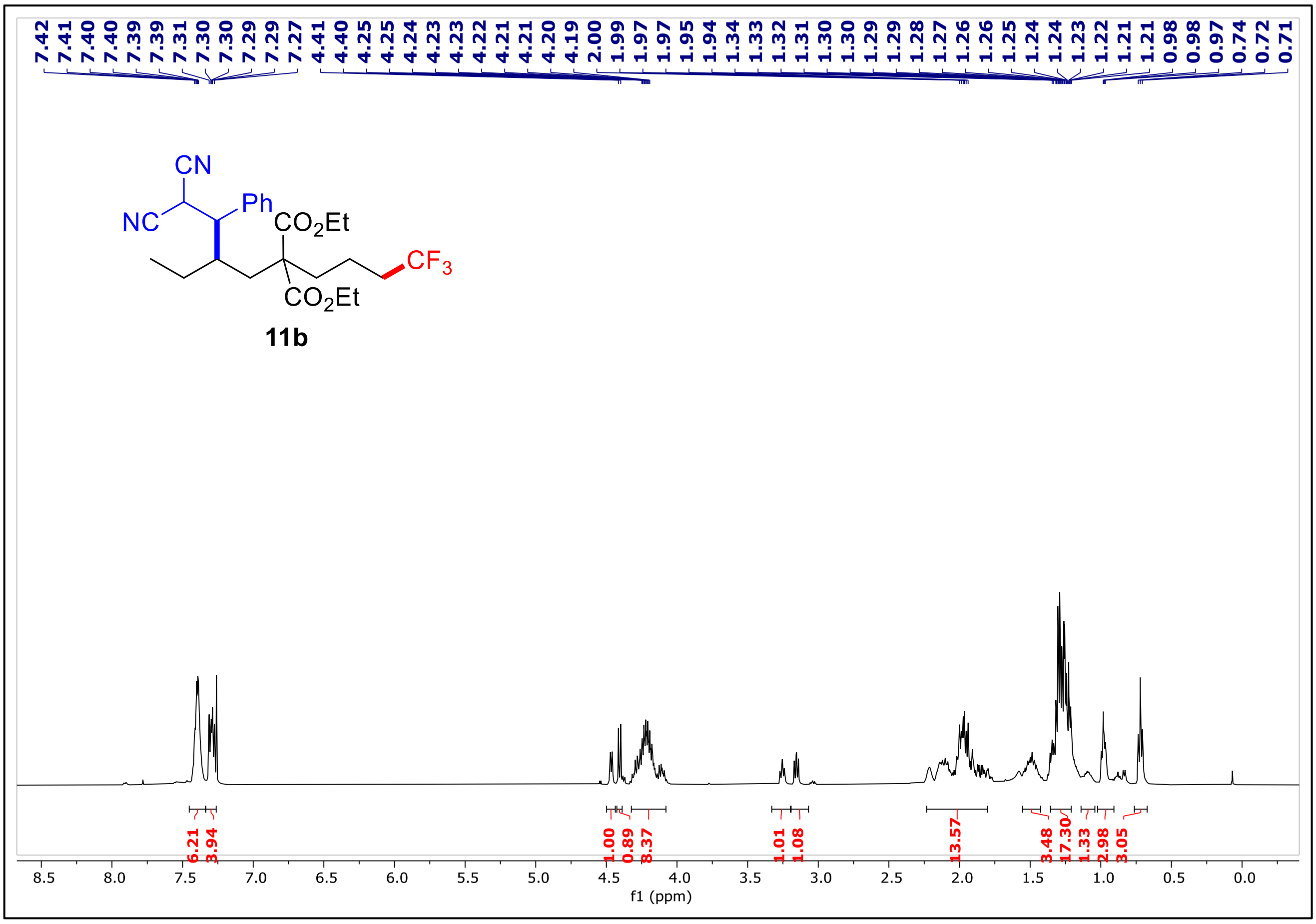

^{1}H NMR of compound **11b** (500 MHz, $CDCl_3$)

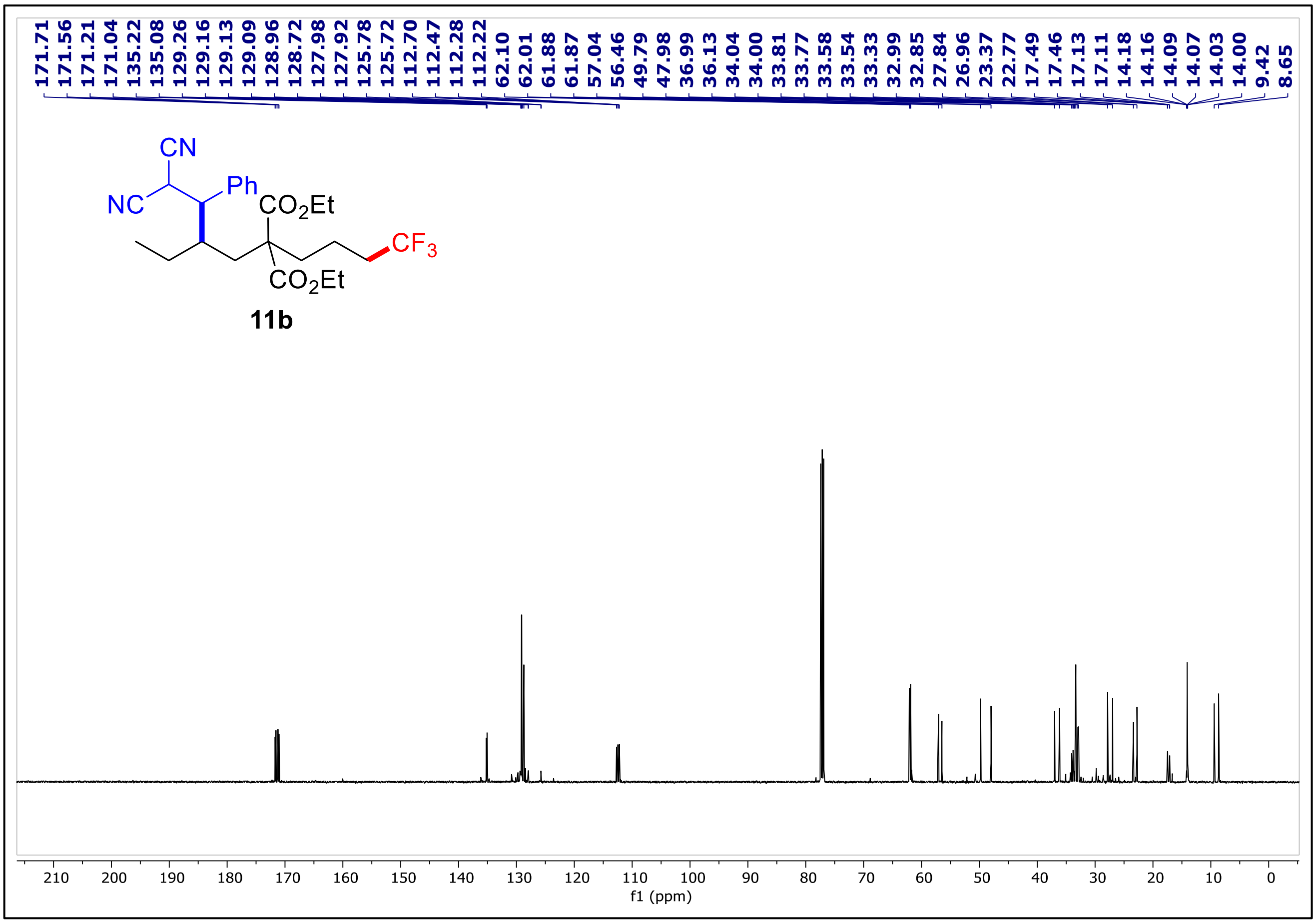

$^{13}C\{^1H\}$ NMR of compound **11b** (126 MHz, $CDCl_3$)

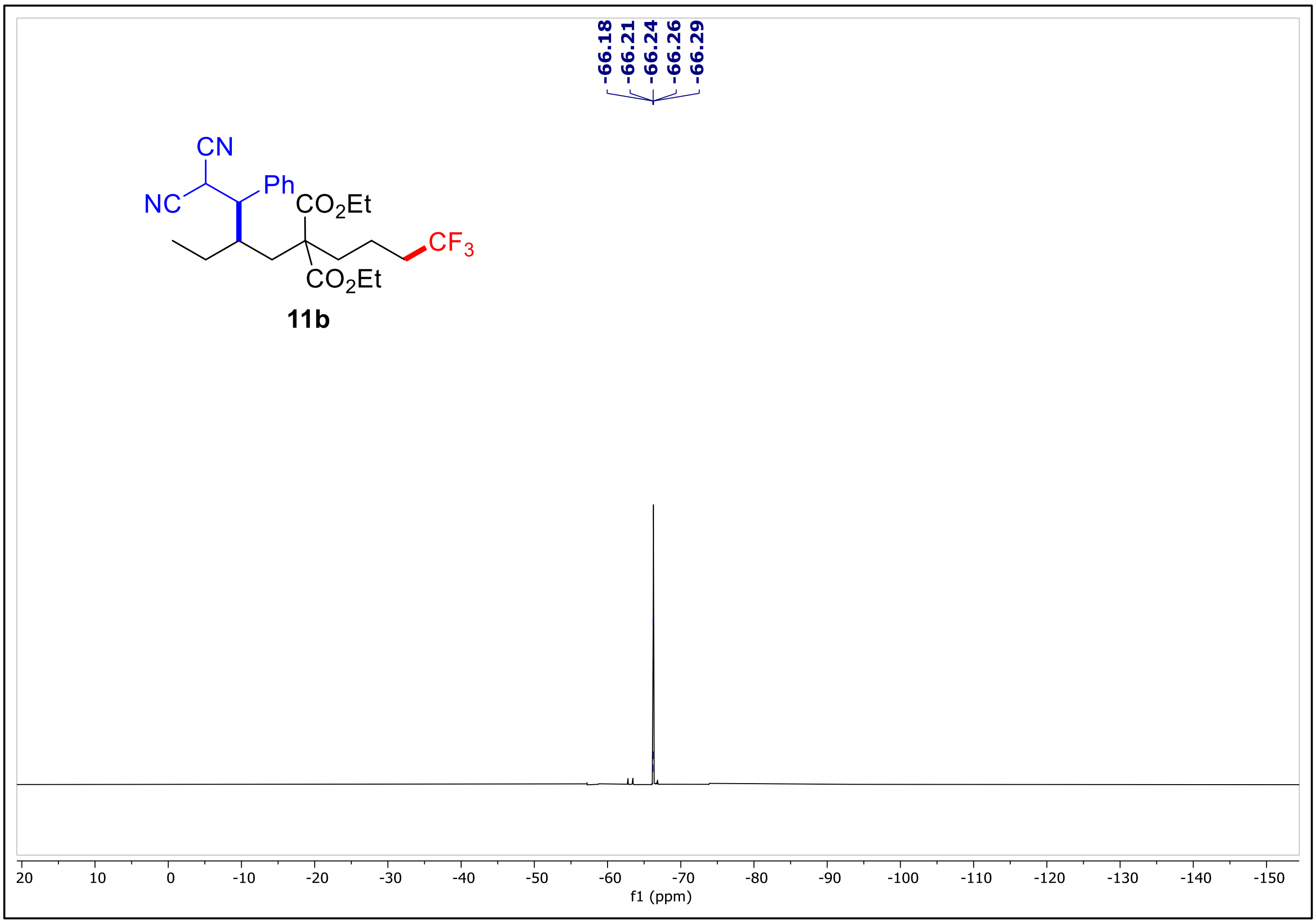

^{19}F NMR of compound **11b** (471 MHz, $CDCl_3$)

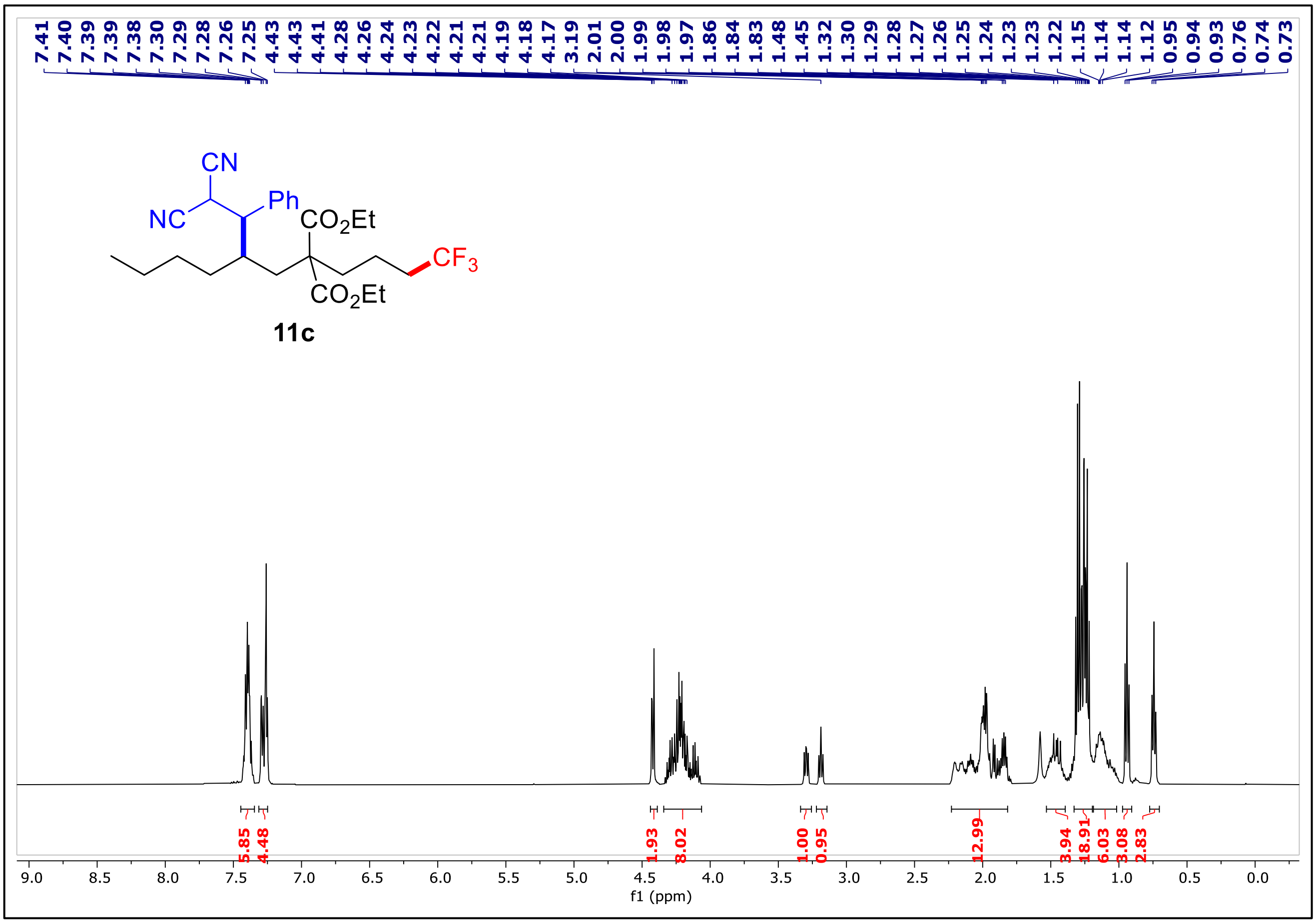

^{1}H NMR of compound **11c** (500 MHz, $CDCl_3$)

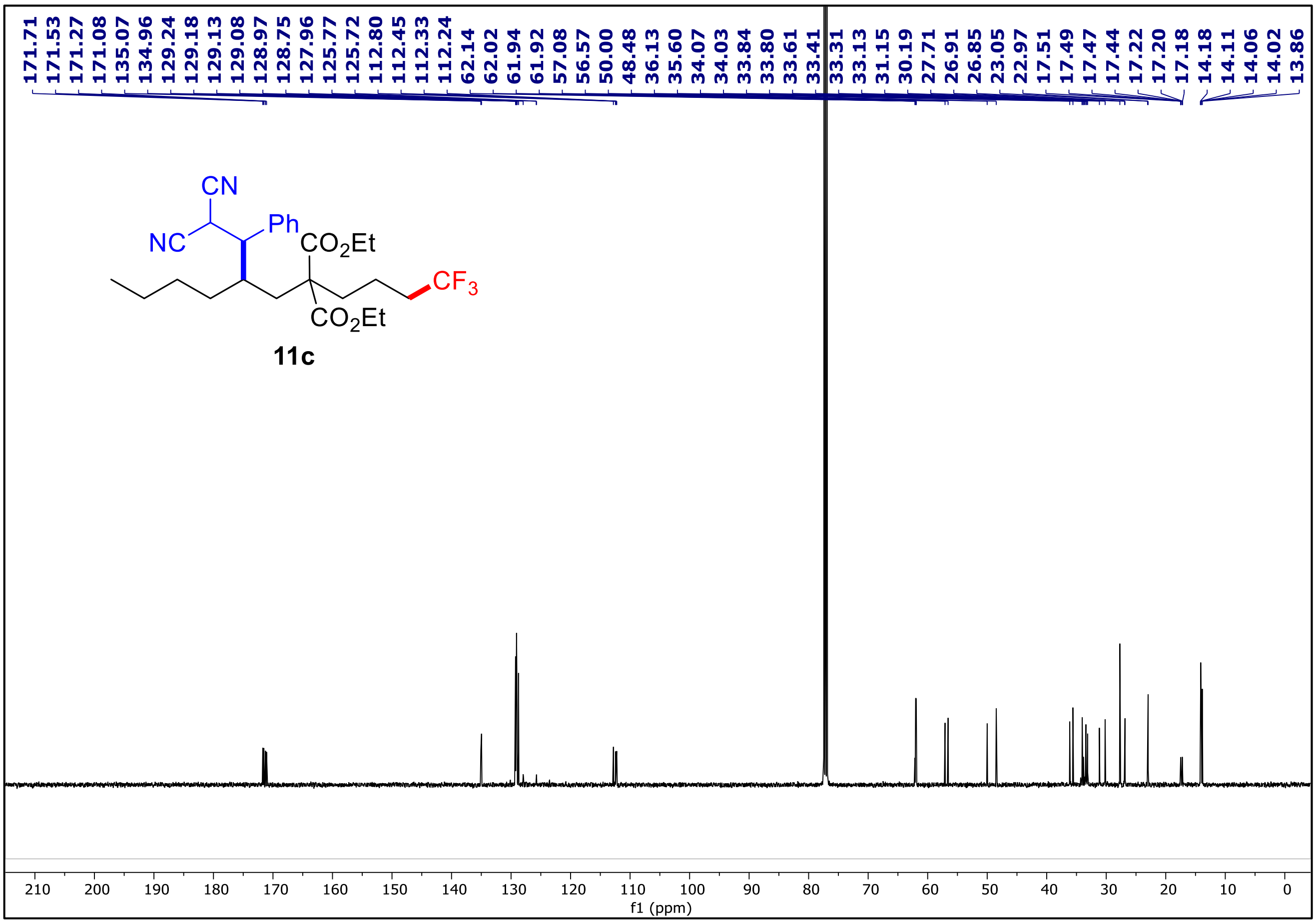

$^{13}C\{^1H\}$ NMR of compound **11c** (126 MHz, $CDCl_3$)

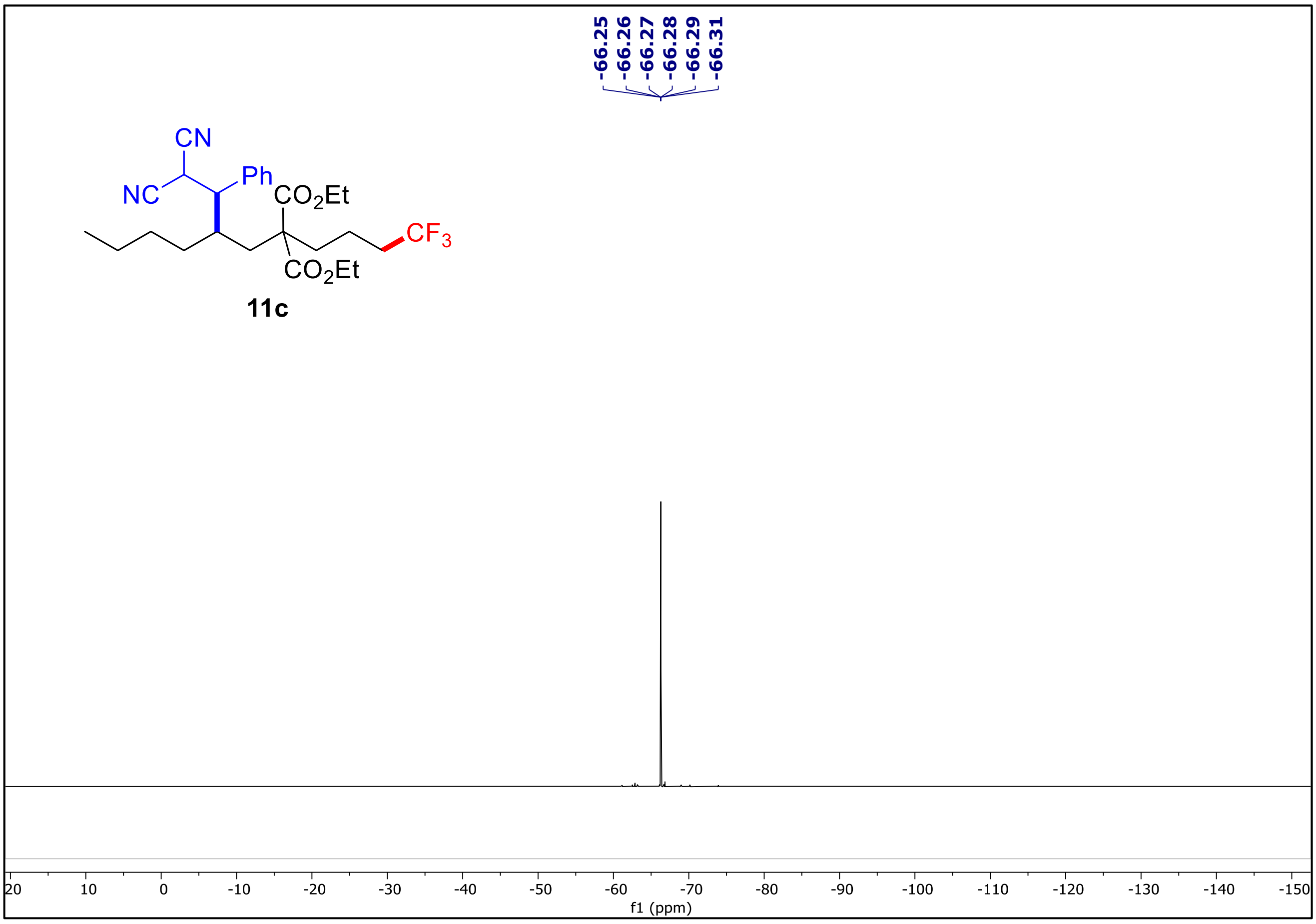

^{19}F NMR of compound **11c** (471 MHz, $CDCl_3$)

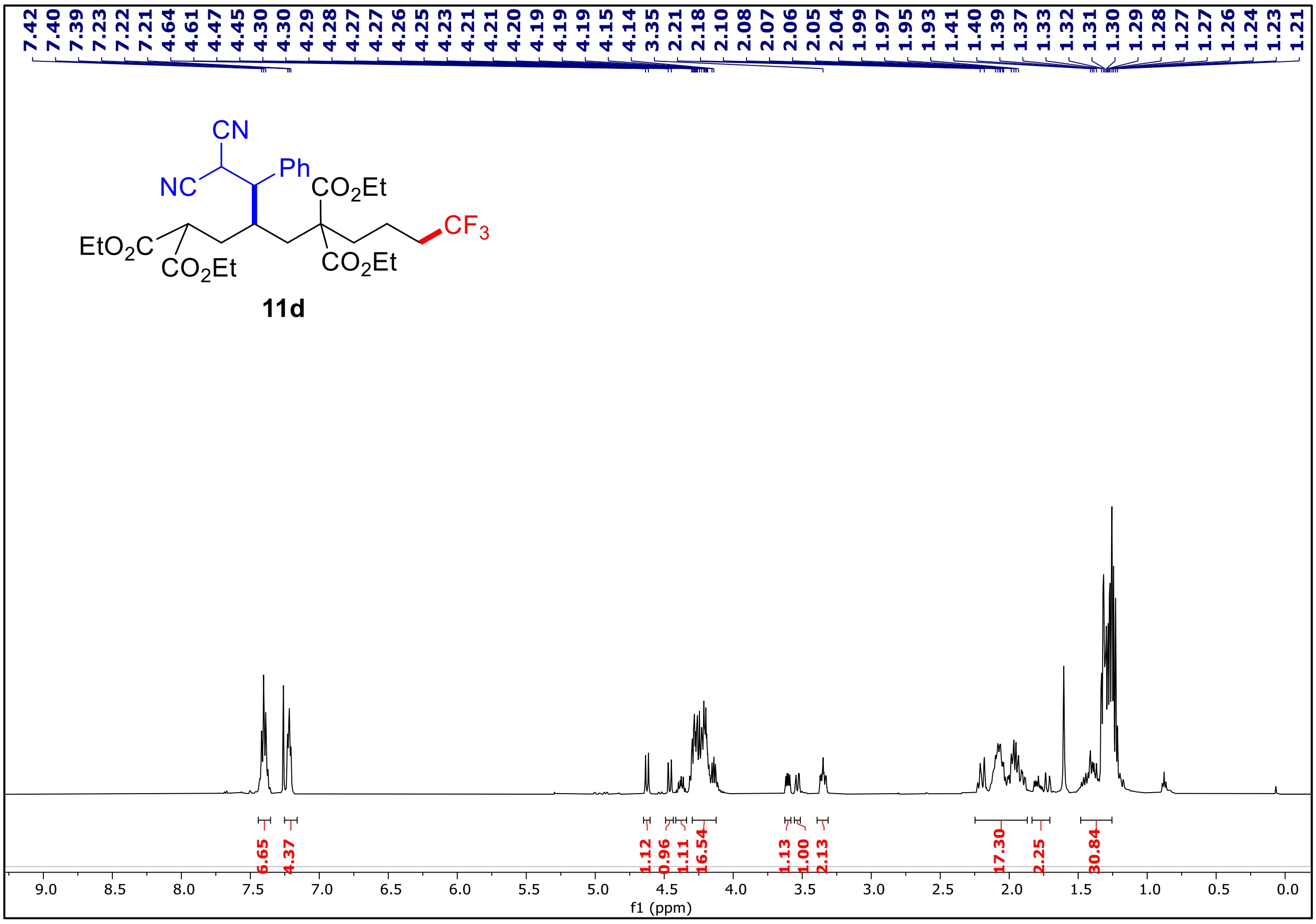

^{1}H NMR of compound **11d** (500 MHz, $CDCl_3$)

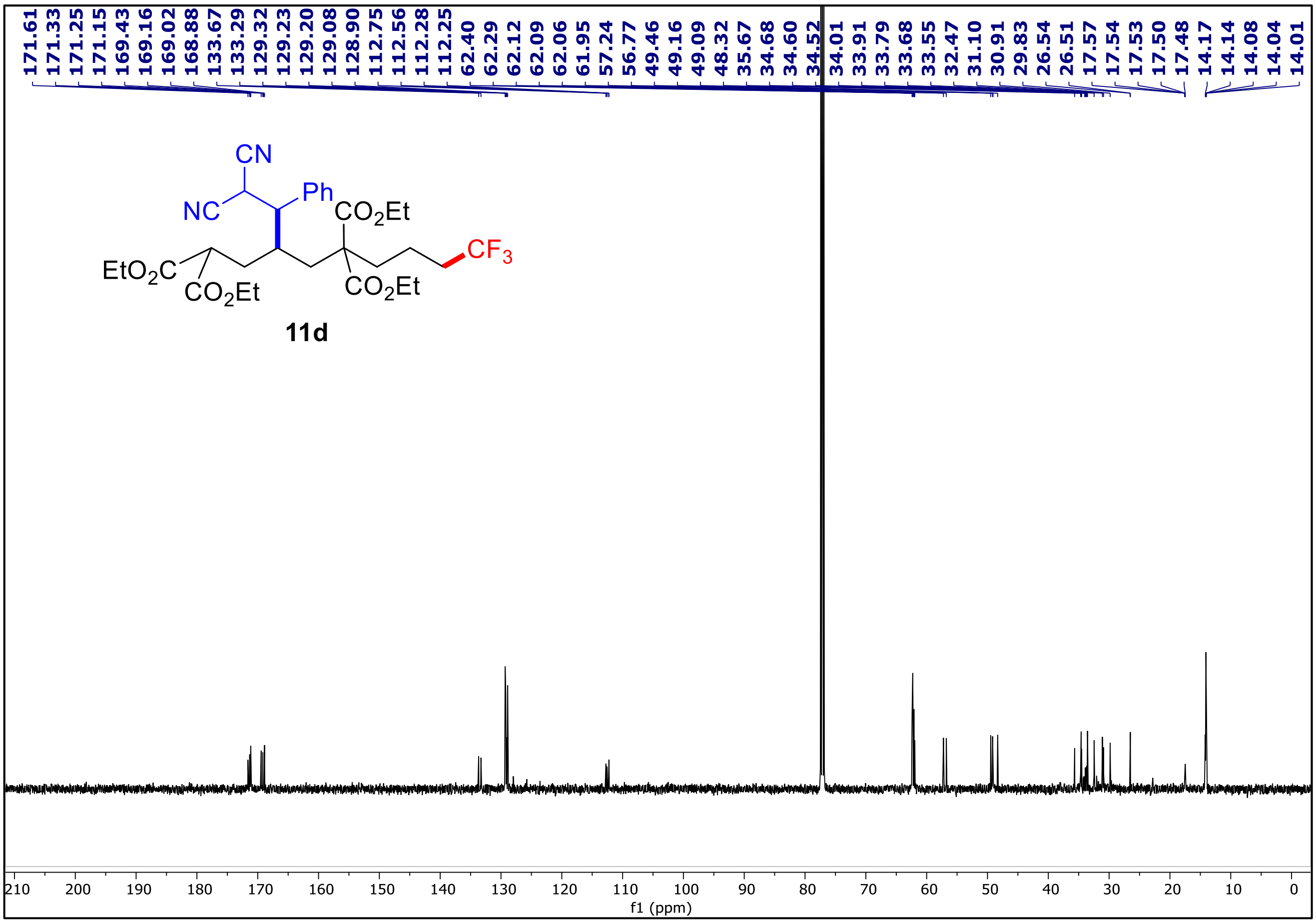

$^{13}C\{^{1}H\}$ NMR of compound **11d** (126 MHz, $CDCl_3$)

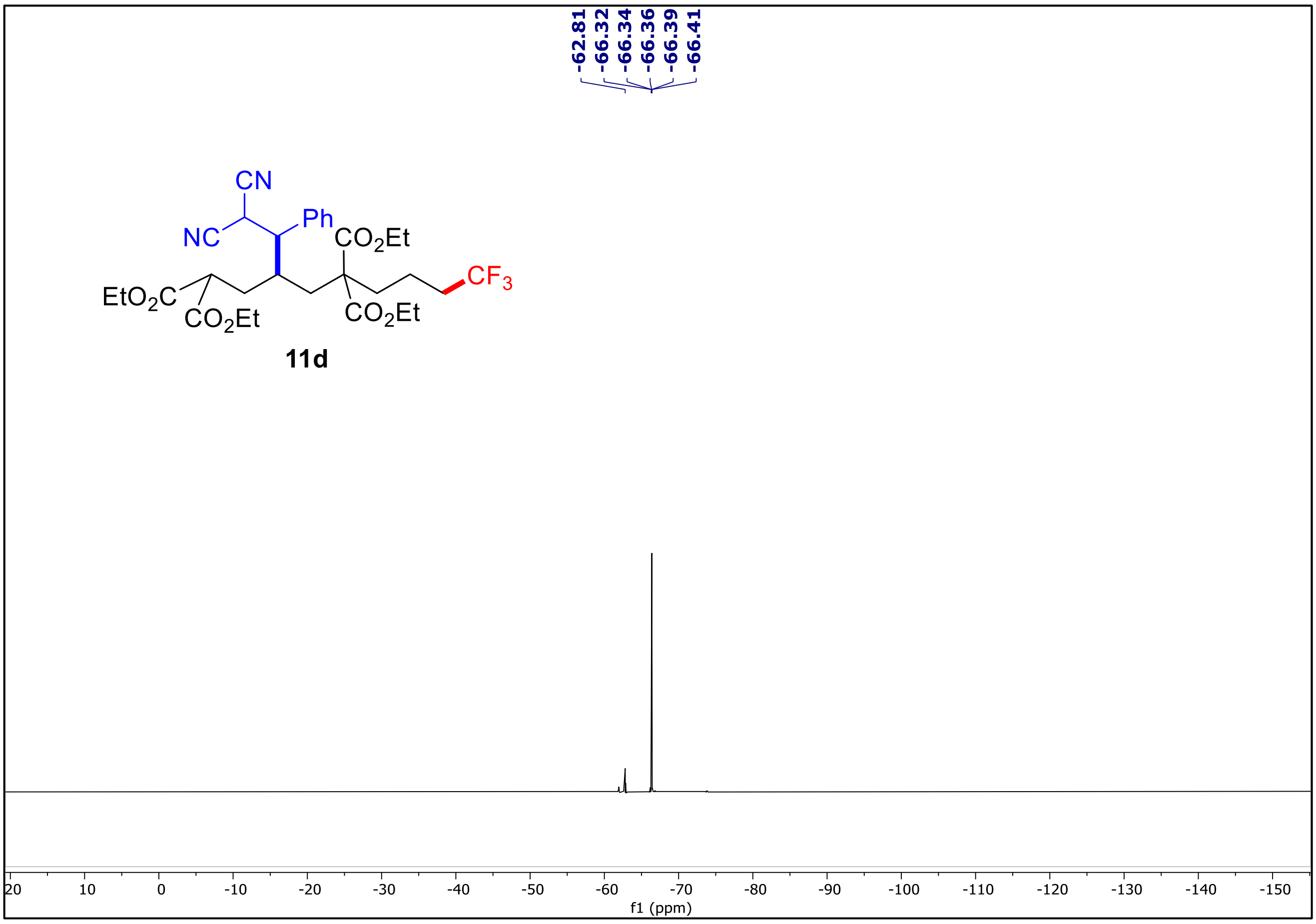

^{19}F NMR of compound **11d** (471 MHz, $CDCl_3$)

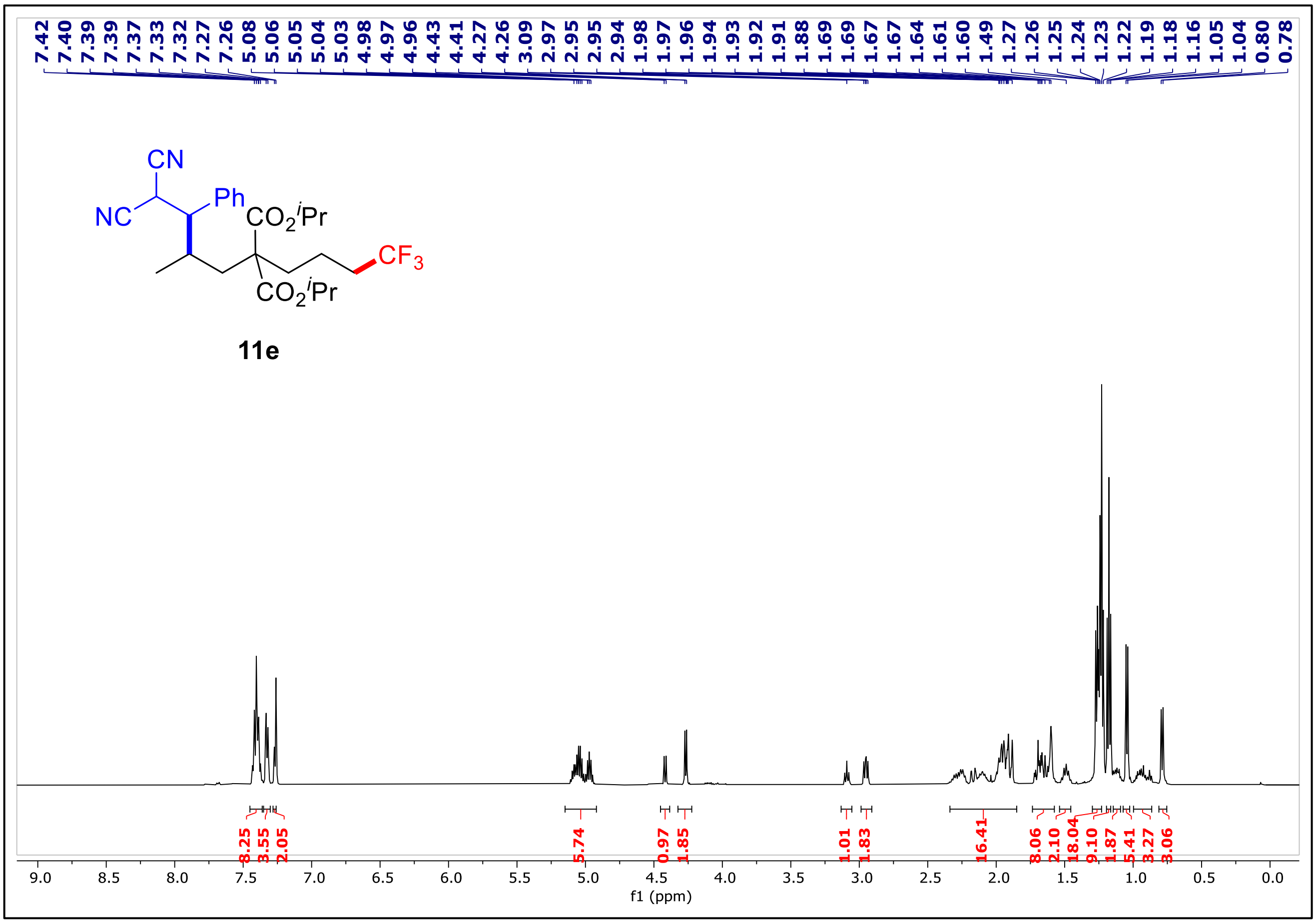

^{1}H NMR of compound **11e** (500 MHz, $CDCl_3$)

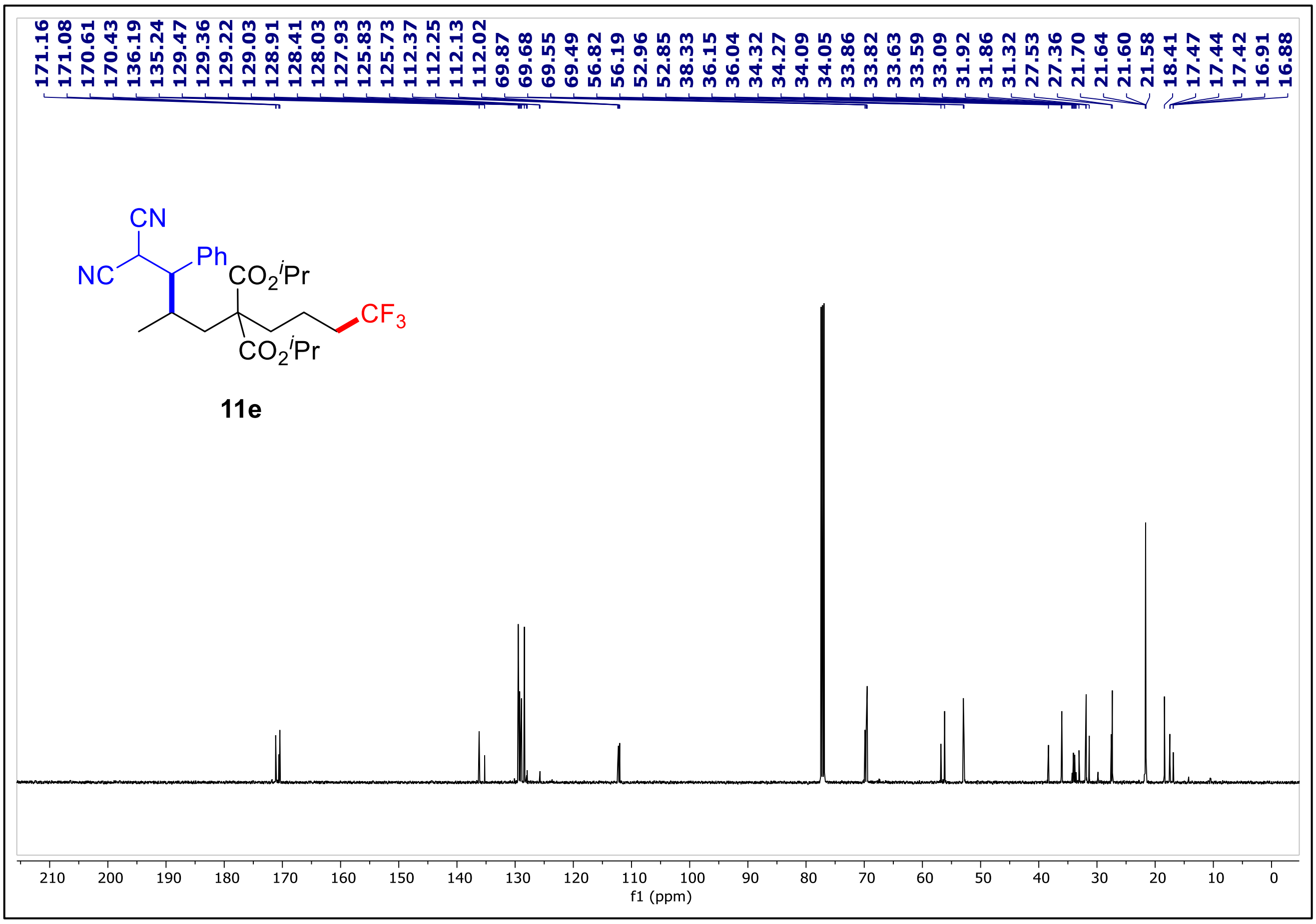

$^{13}C\{^{1}H\}$ NMR of compound **11e** (126 MHz, $CDCl_3$)

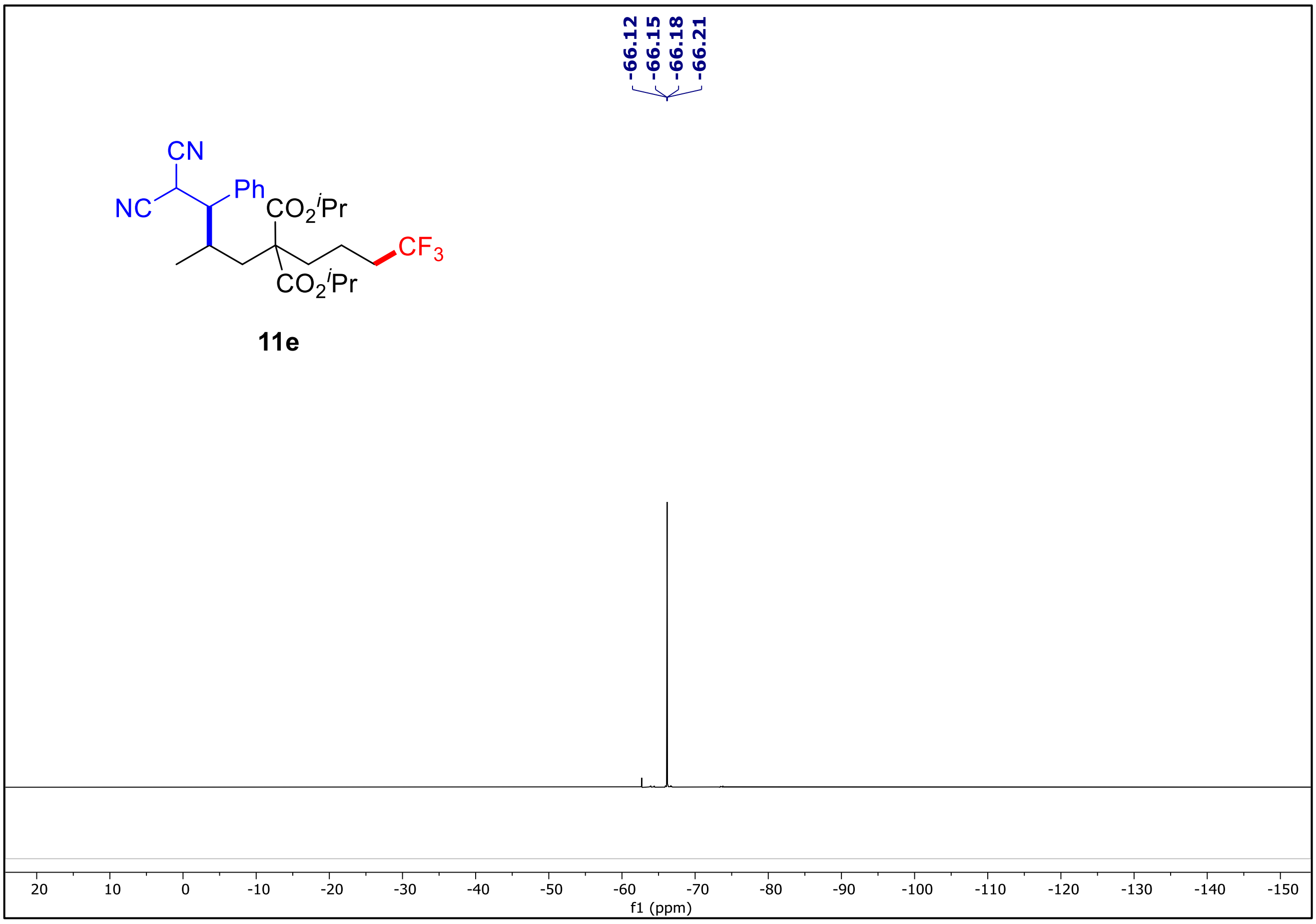

^{19}F NMR of compound **11e** (471 MHz, $CDCl_3$)

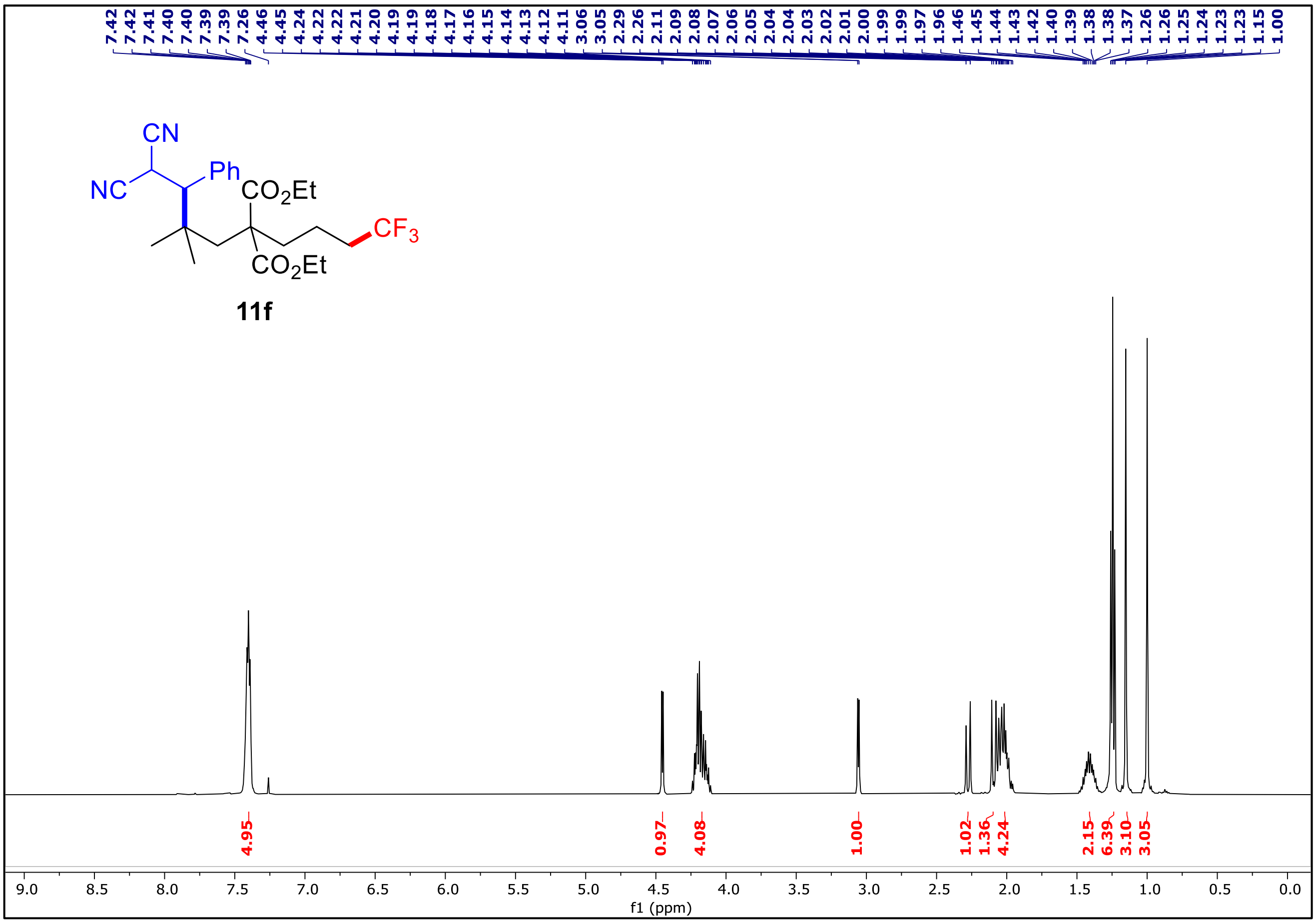

^{1}H NMR of compound **11f** (500 MHz, $CDCl_3$)

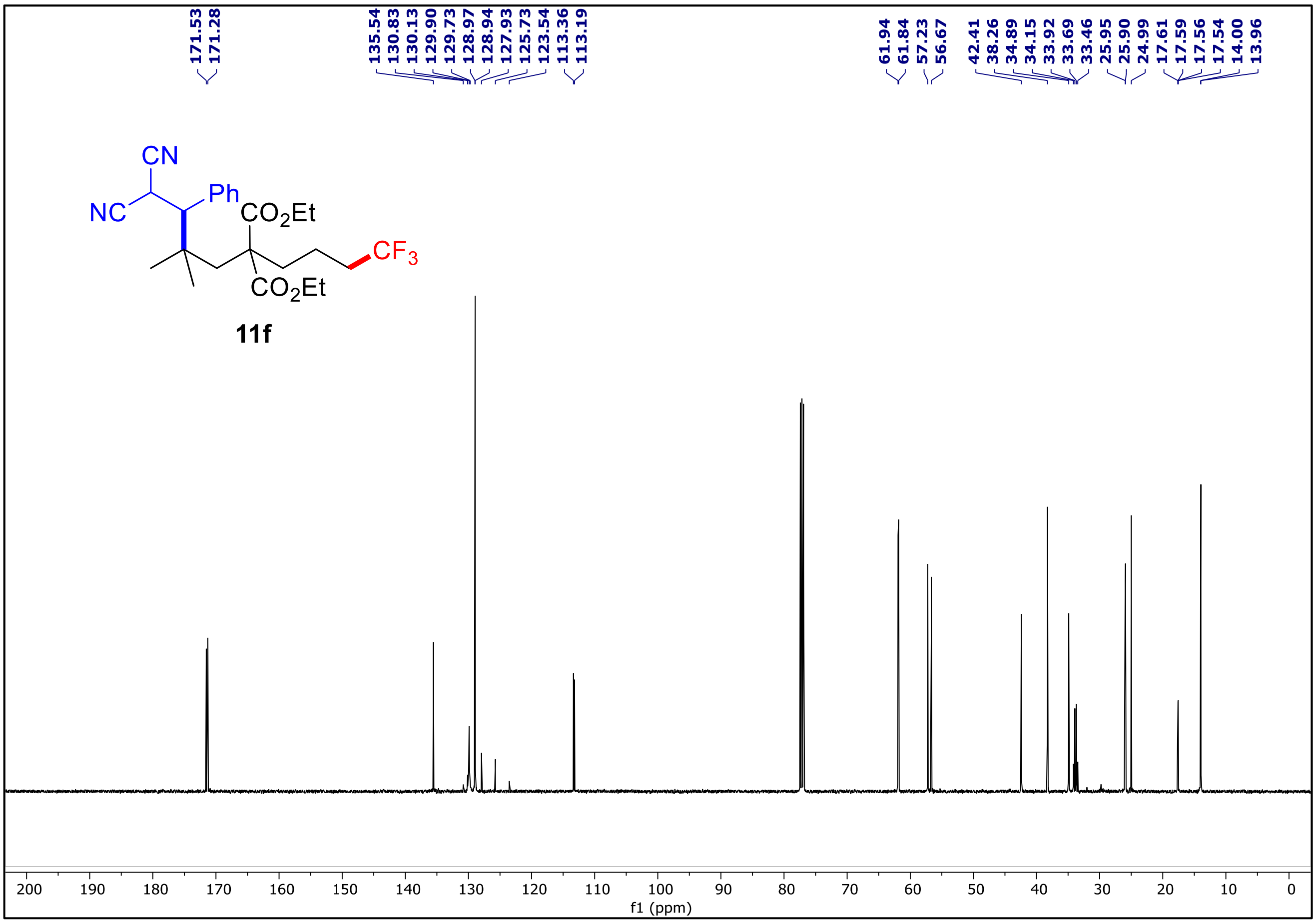

$^{13}C\{^1H\}$ NMR of compound **11f** (126 MHz, CDCl3)

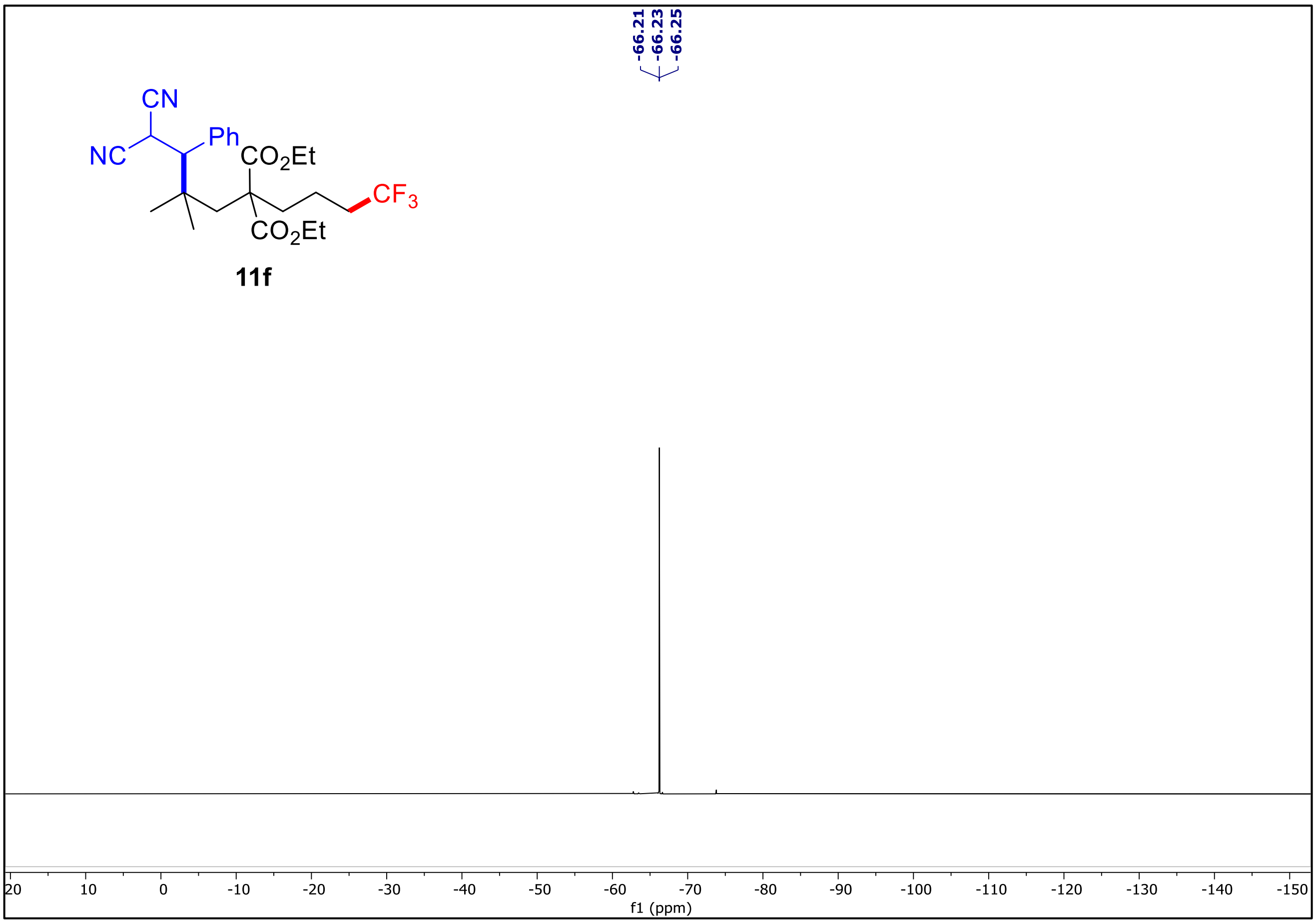

^{19}F NMR of compound **11f** (471 MHz, CDCl3)

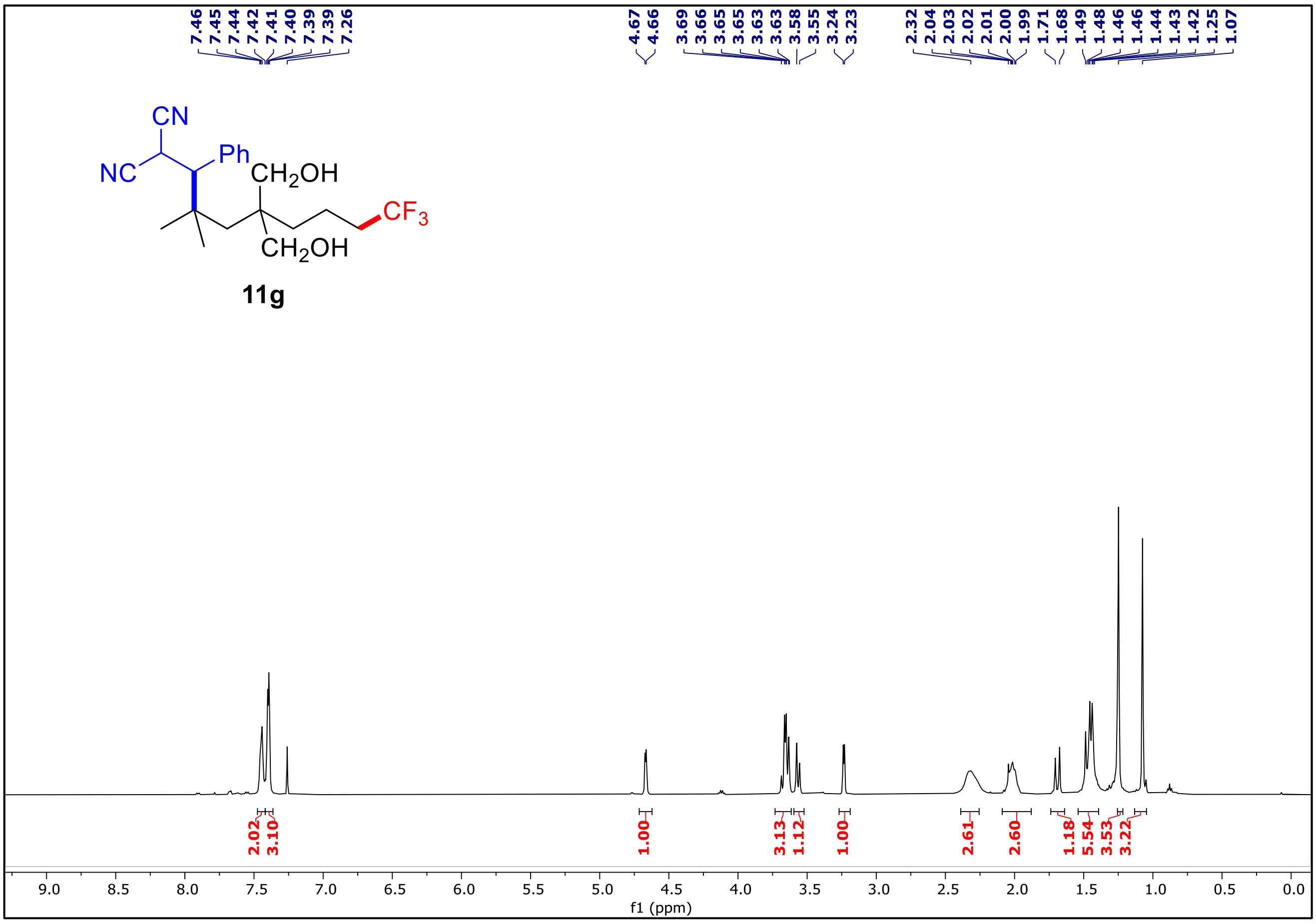

^{1}H NMR of compound **11g** (500 MHz, $CDCl_3$)

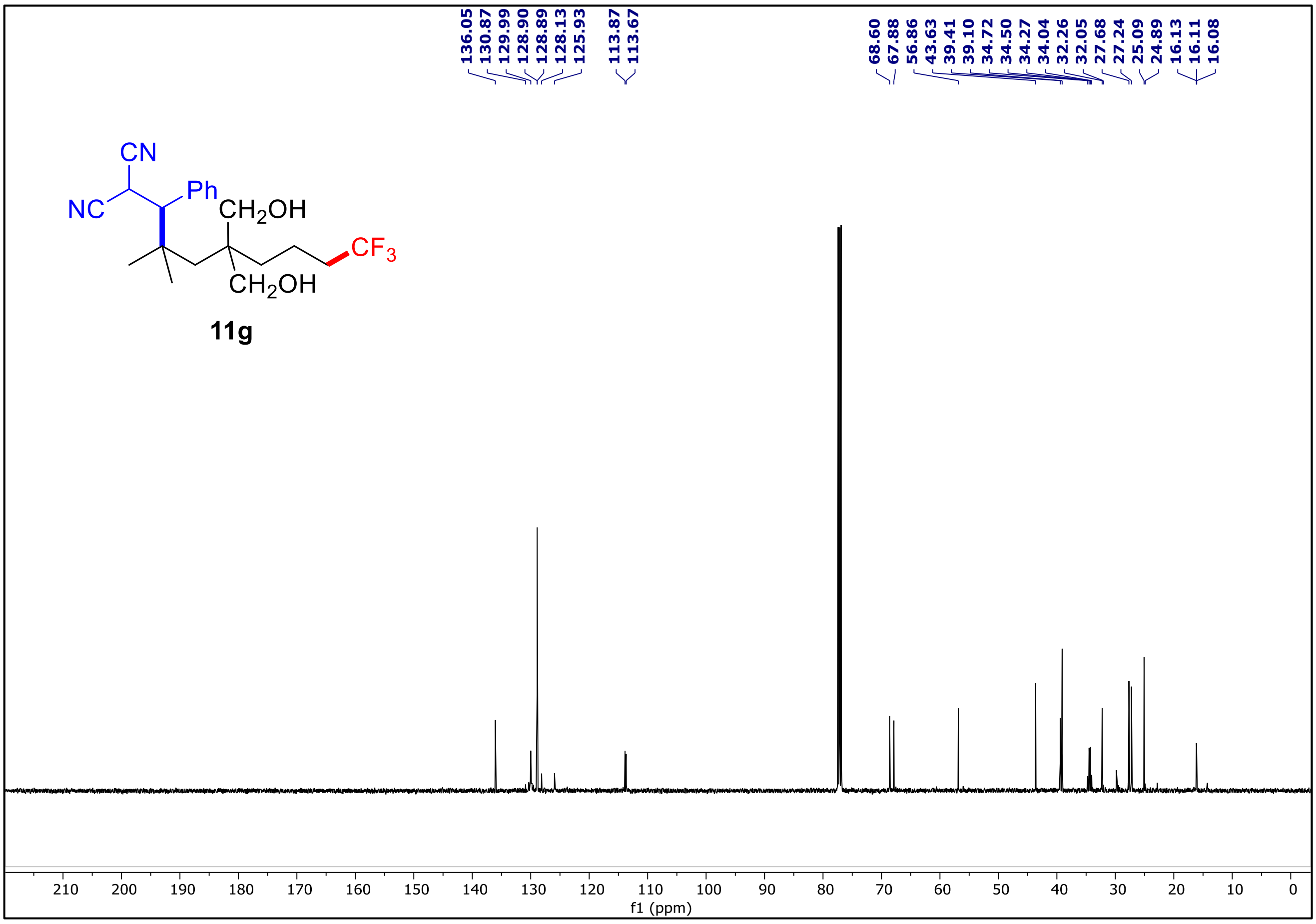

$^{13}C\{^{1}H\}$ NMR of compound **11g** (126 MHz, $CDCl_3$)

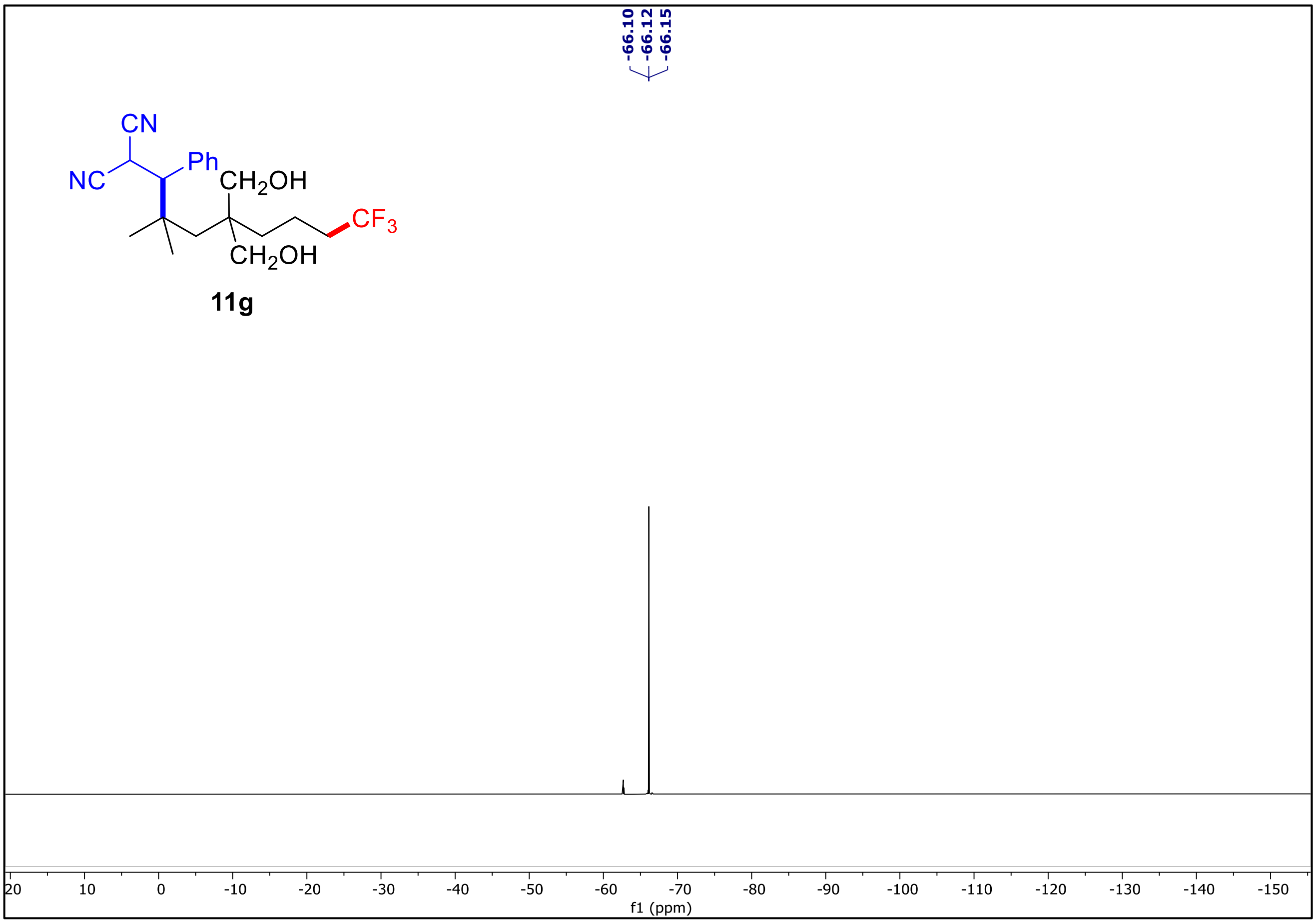

^{19}F NMR of compound **11g** (471 MHz, $CDCl_3$)

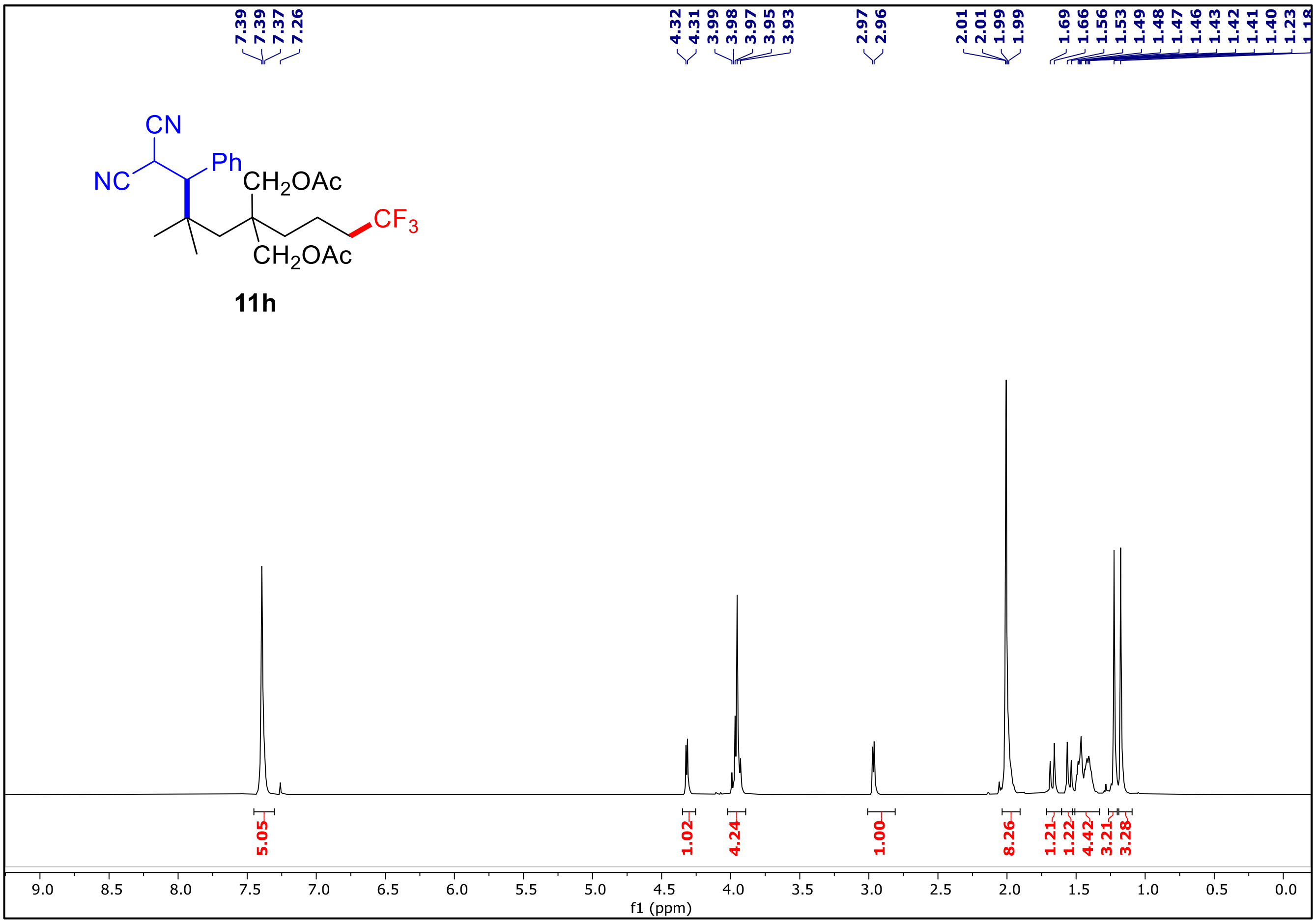

^{1}H NMR of compound **11h** (500 MHz, $CDCl_3$)

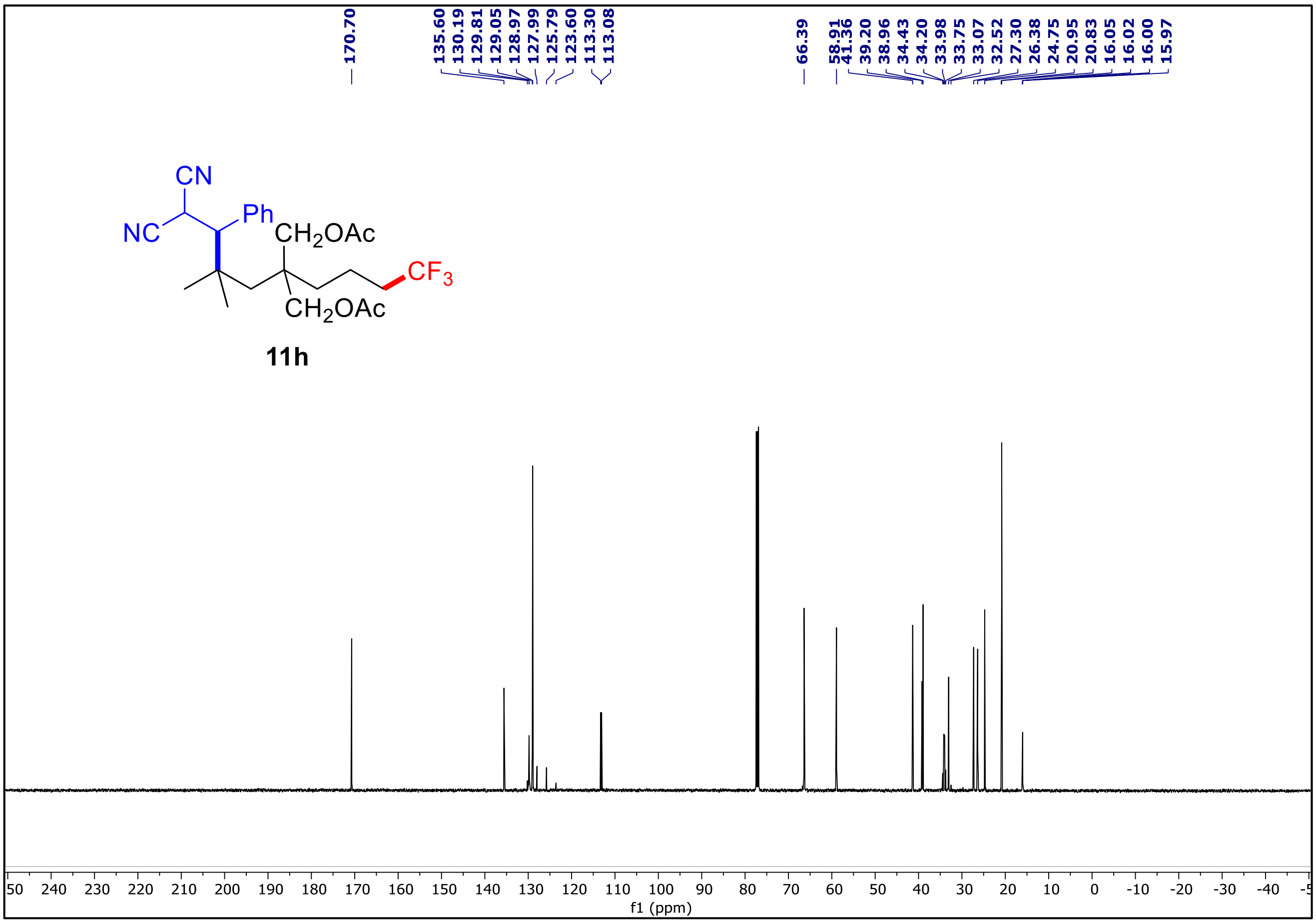

$^{13}C\{^1H\}$ NMR of compound **11h** (126 MHz, $CDCl_3$)

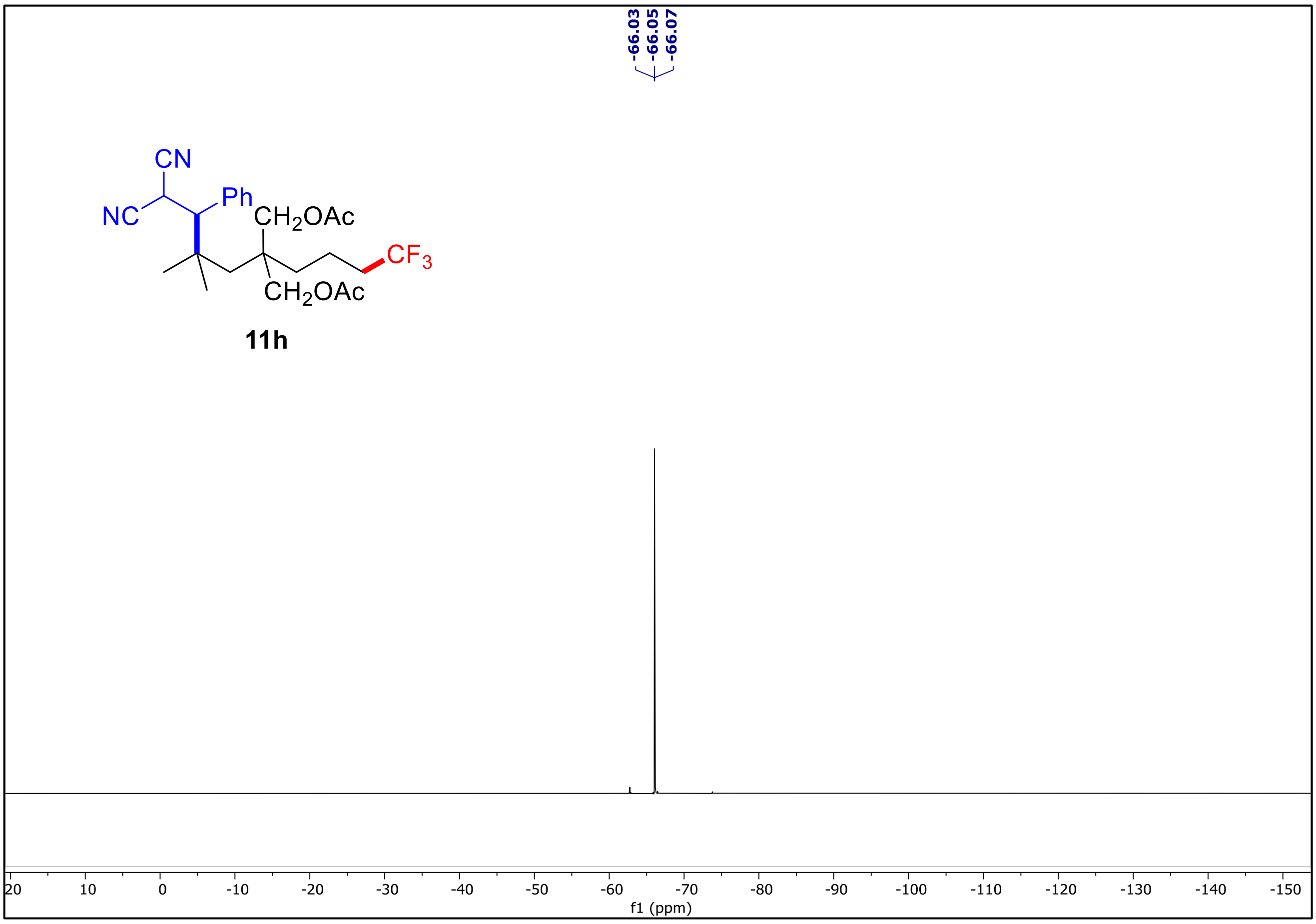

^{19}F NMR of compound **11h** (471 MHz, $CDCl_3$)

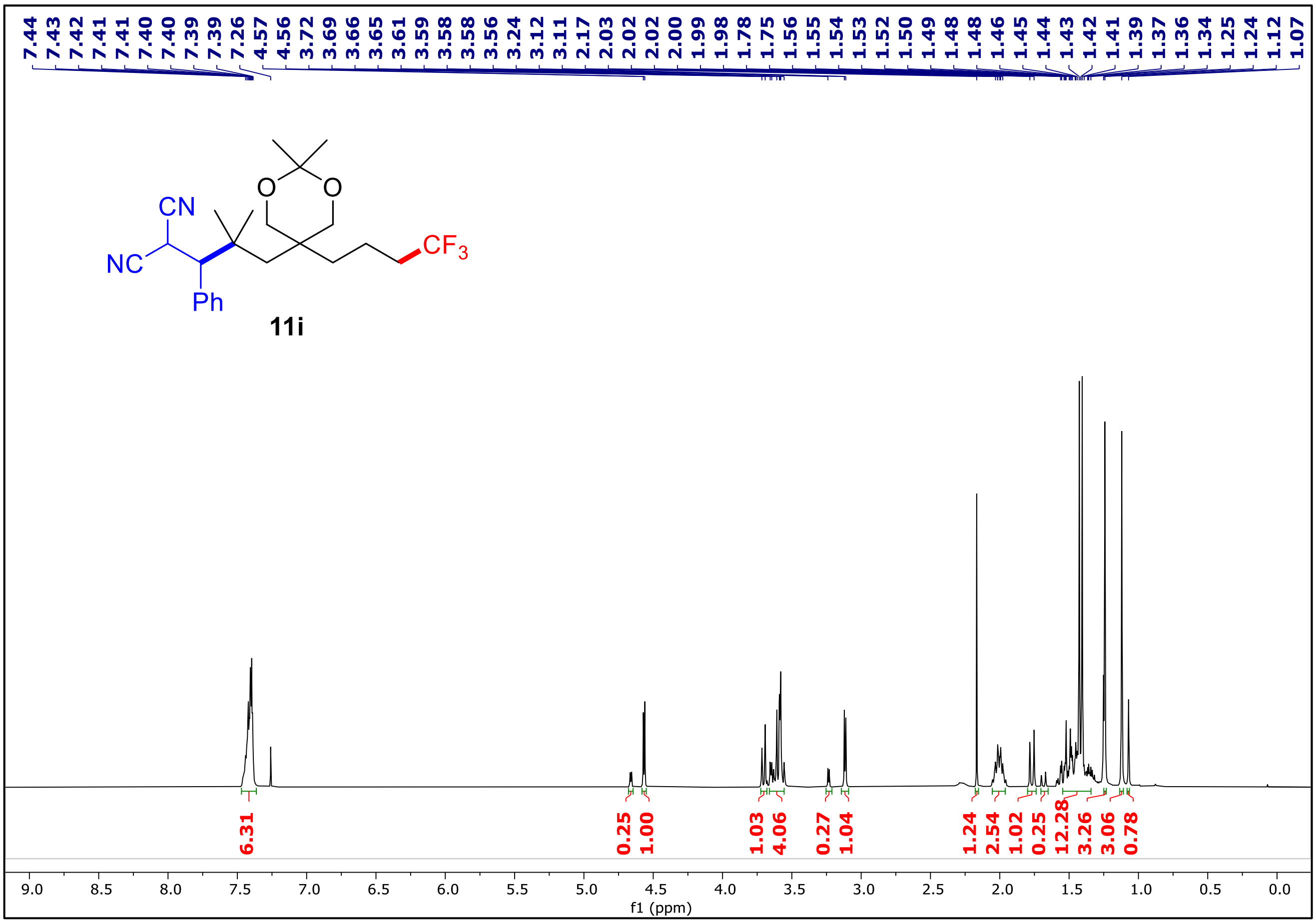

^{1}H NMR of compound **11i** (500 MHz, $CDCl_3$)

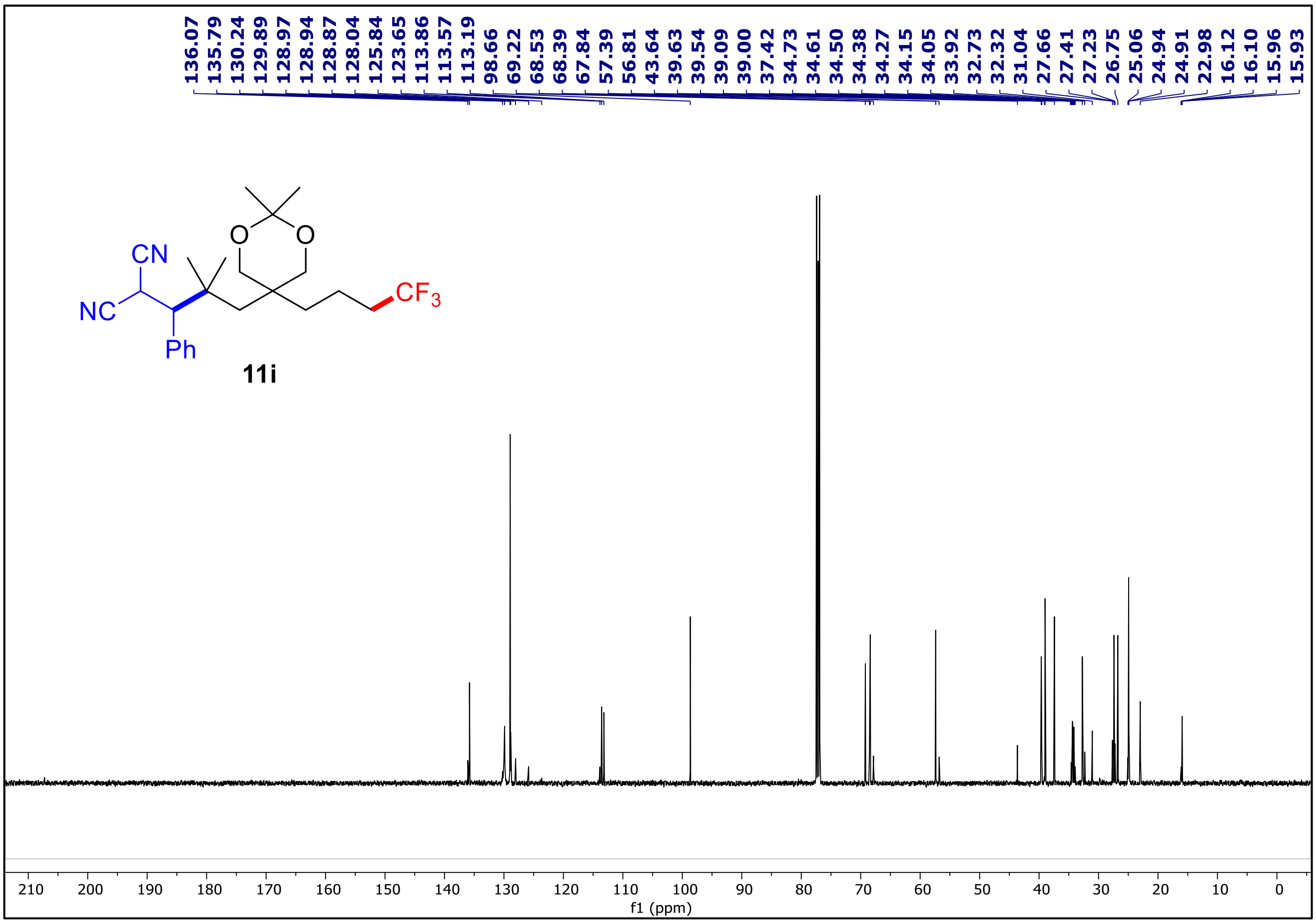

$^{13}C\{^1H\}$ NMR of compound **11i** (126 MHz, $CDCl_3$)

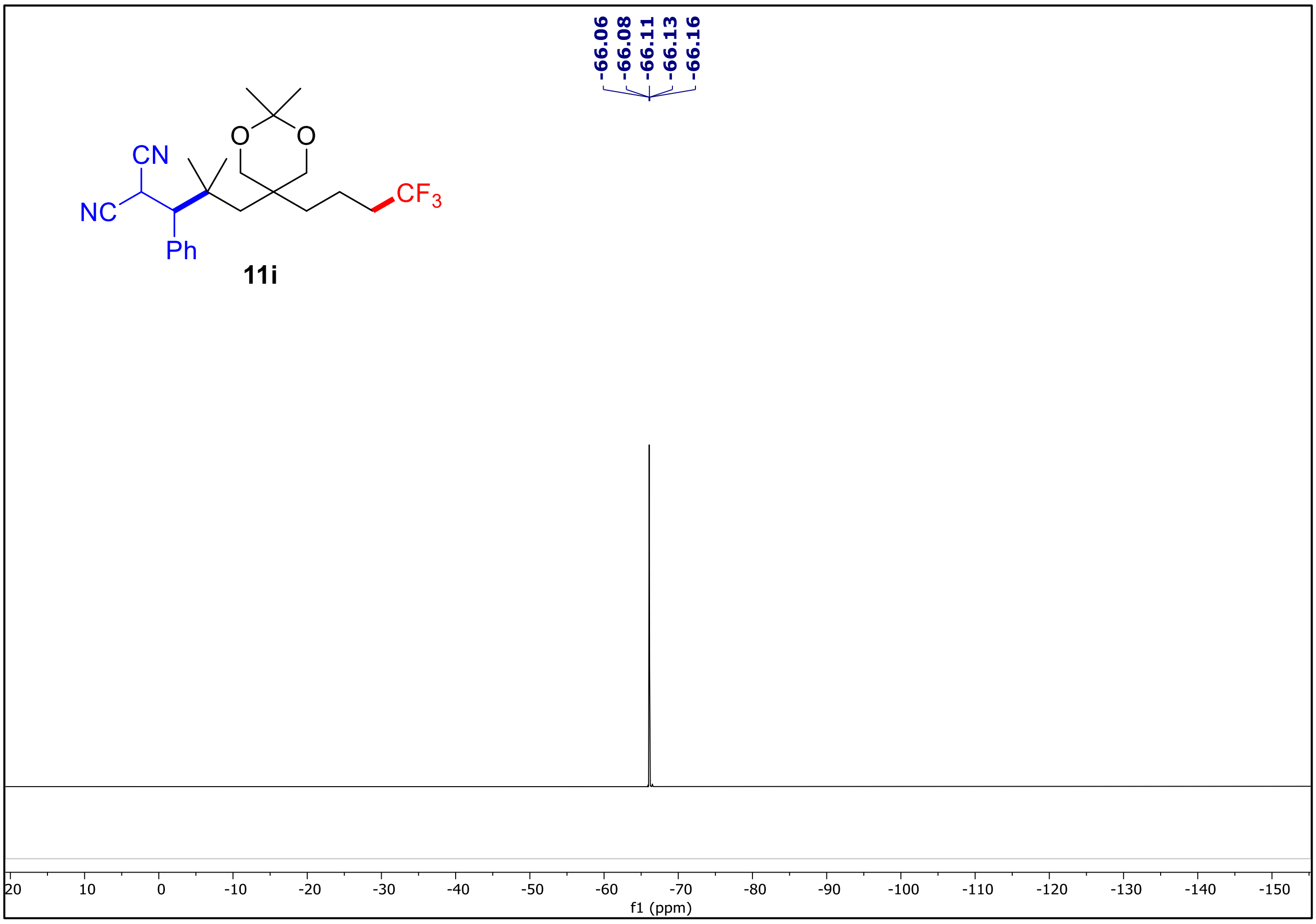

^{19}F NMR of compound **11i** (471 MHz, $CDCl_3$)

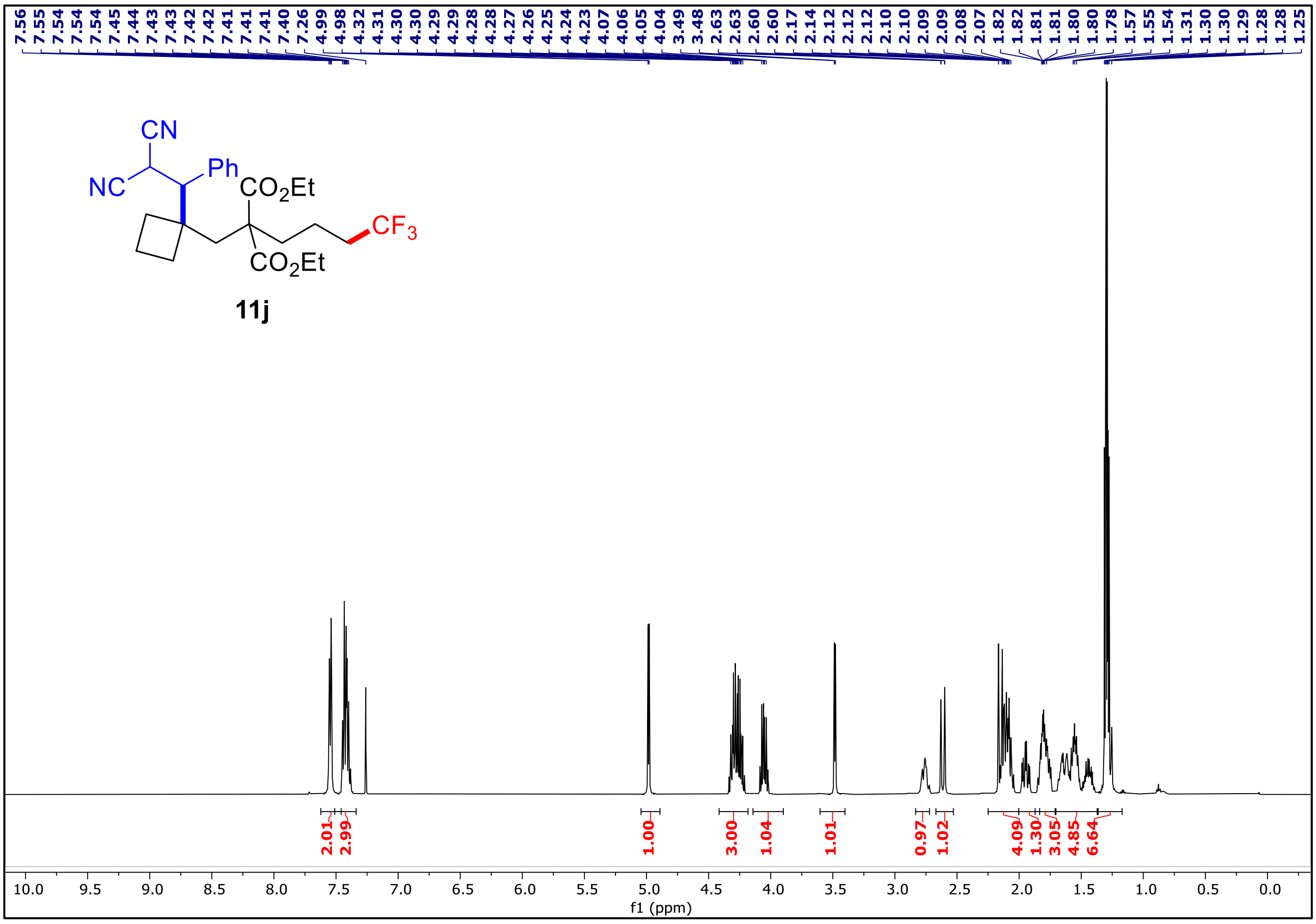

^{1}H NMR of compound **11j** (500 MHz, $CDCl_3$)

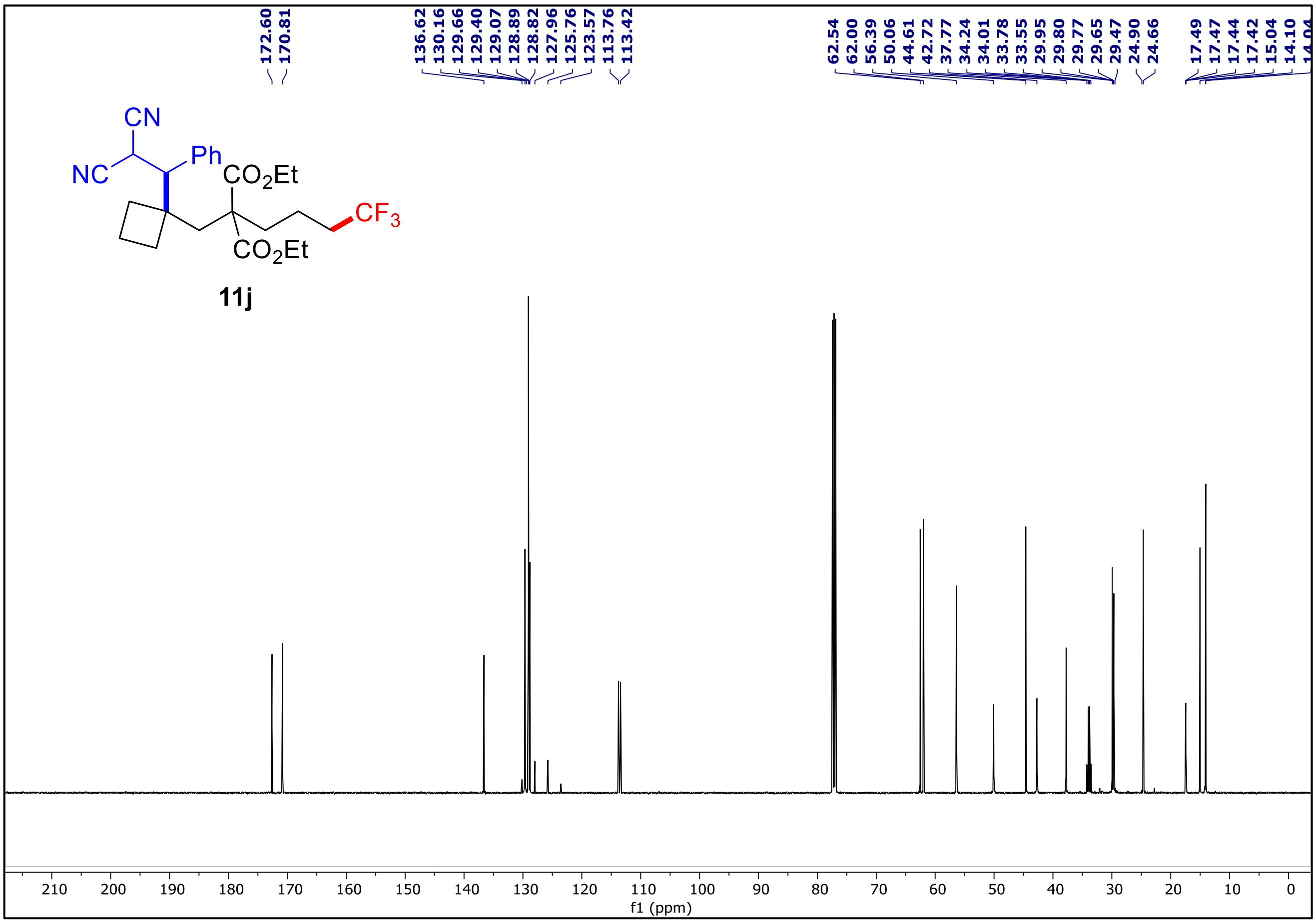

$^{13}C\{^{1}H\}$ NMR of compound **11j** (126 MHz, $CDCl_3$)

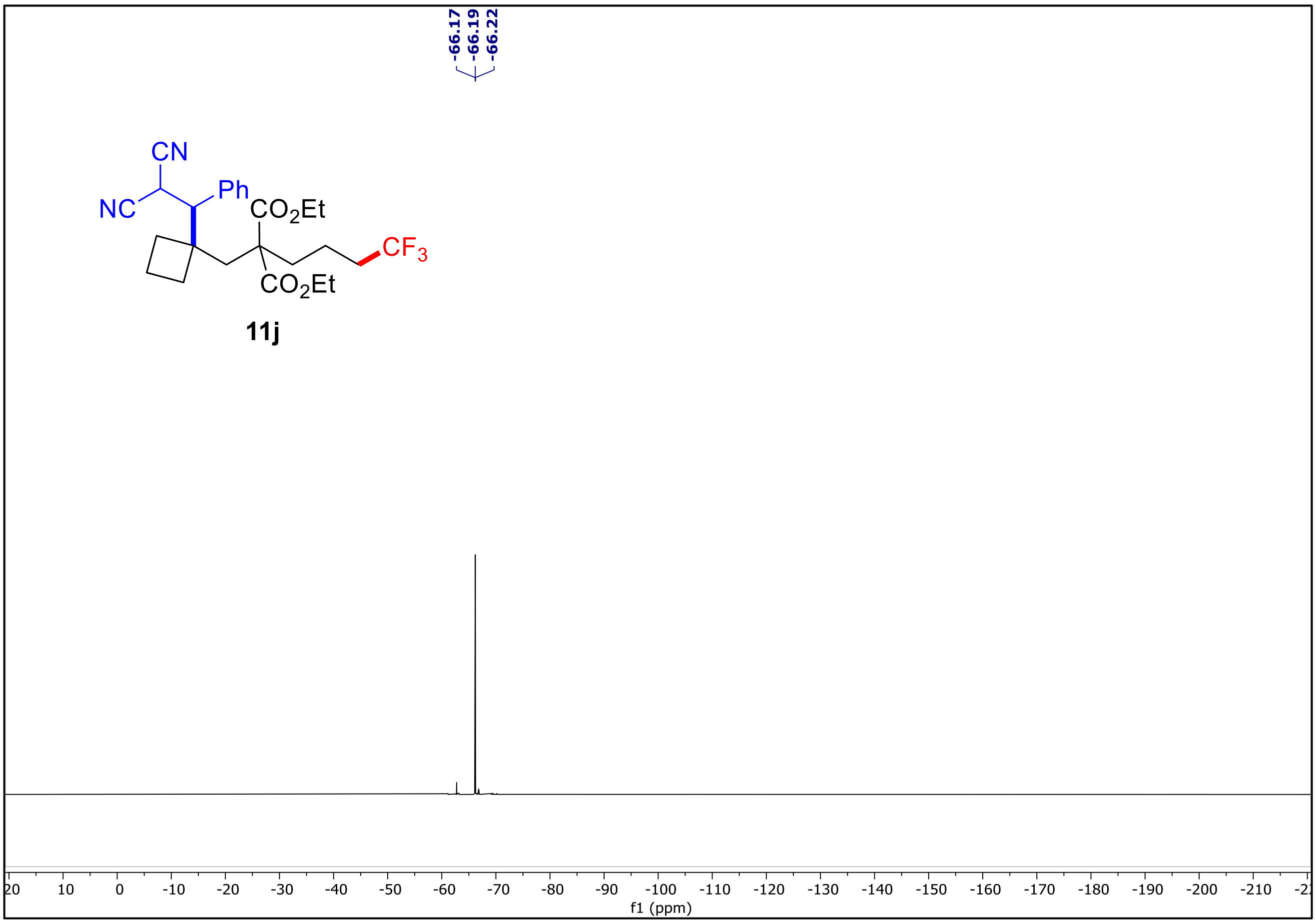

^{19}F NMR of compound **11j** (471 MHz, $CDCl_3$)

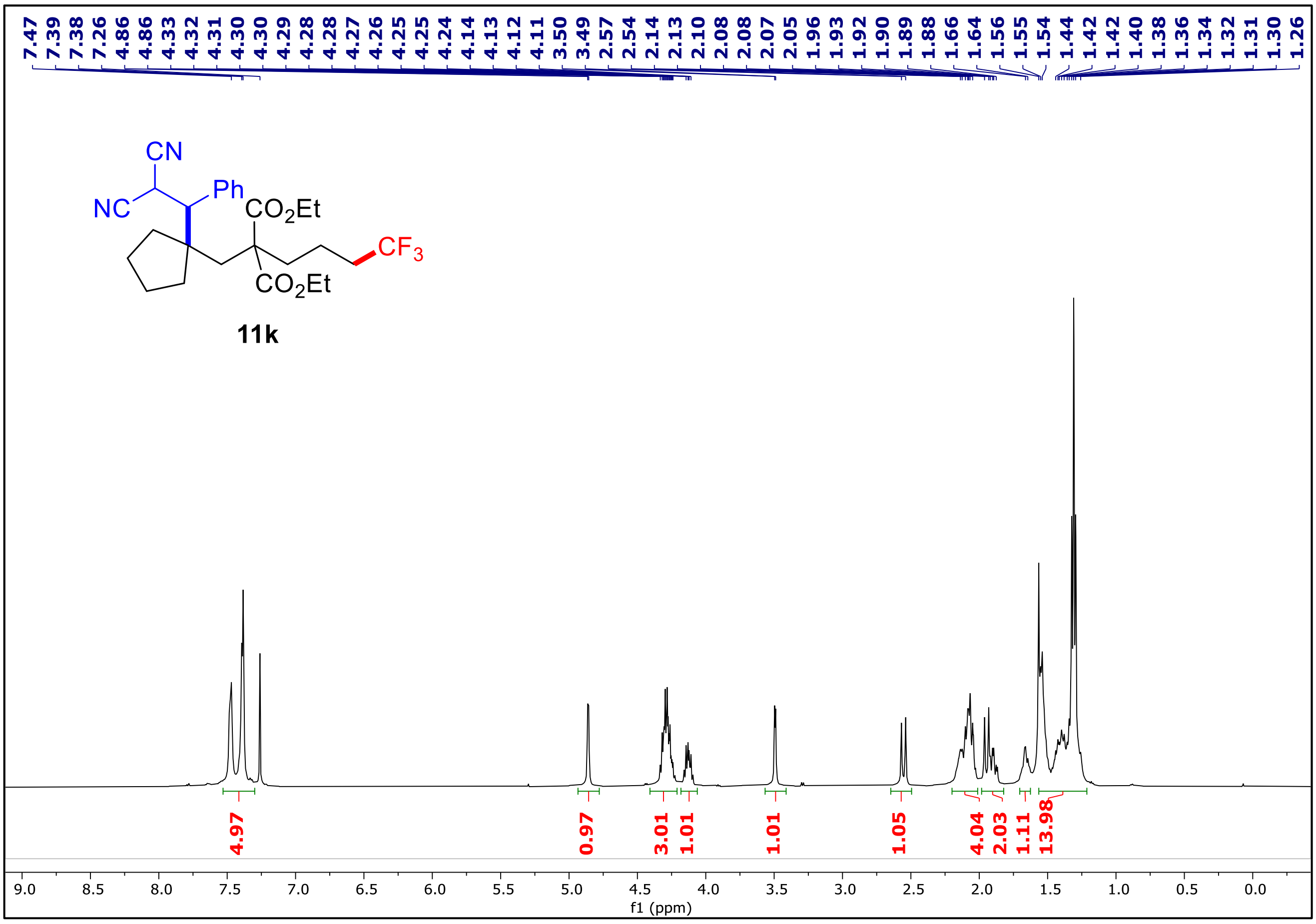

^{1}H NMR of compound **11k** (500 MHz, CDCl$_3$)

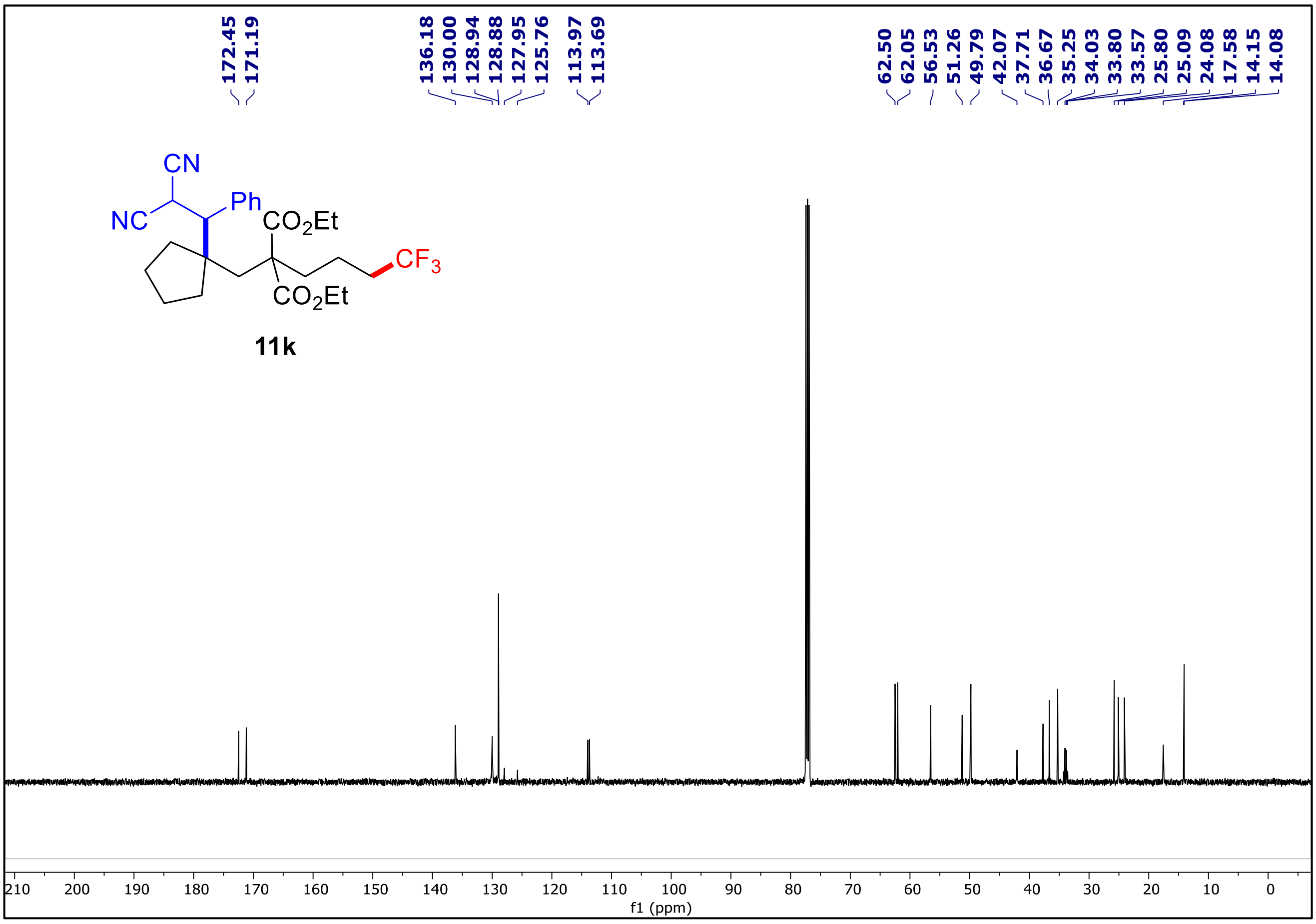

^{13}C{^{1}H} NMR of compound **11k** (126 MHz, CDCl$_3$)

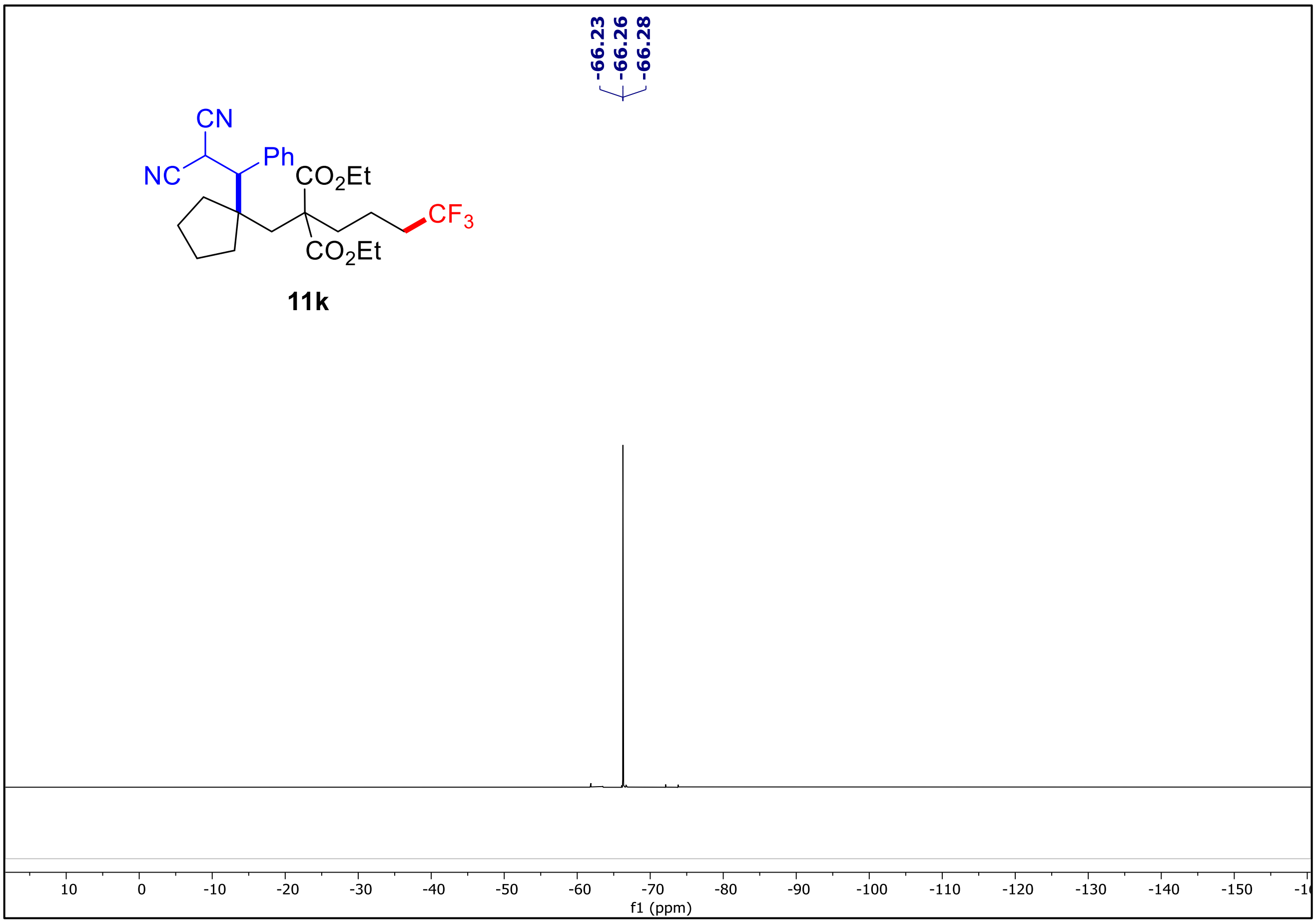

^{19}F NMR of compound **11k** (471 MHz, $CDCl_3$)

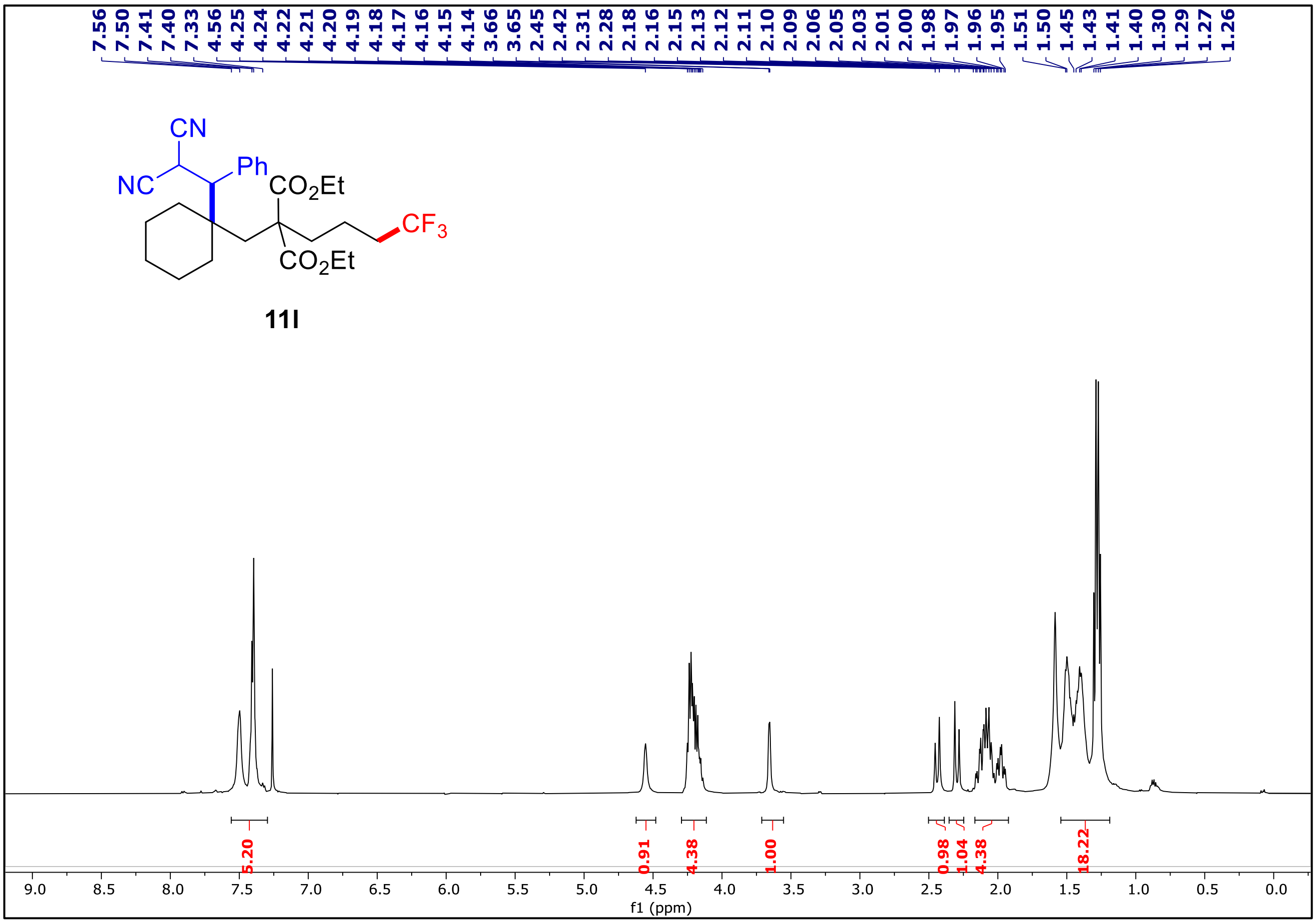

^{1}H NMR of compound **11l** (500 MHz, $CDCl_3$)

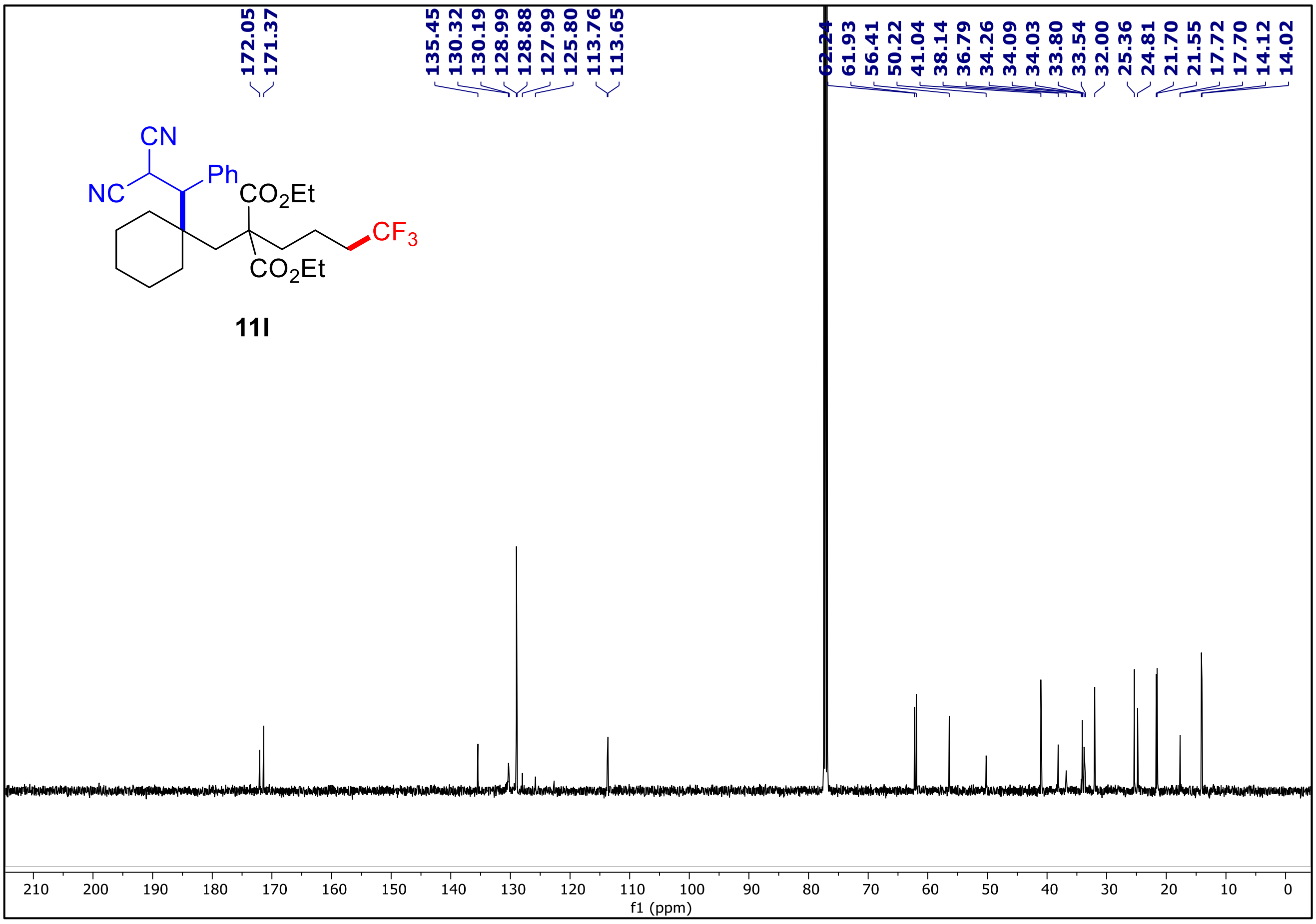

$^{13}C\{^{1}H\}$ NMR of compound **11l** (126 MHz, $CDCl_3$)

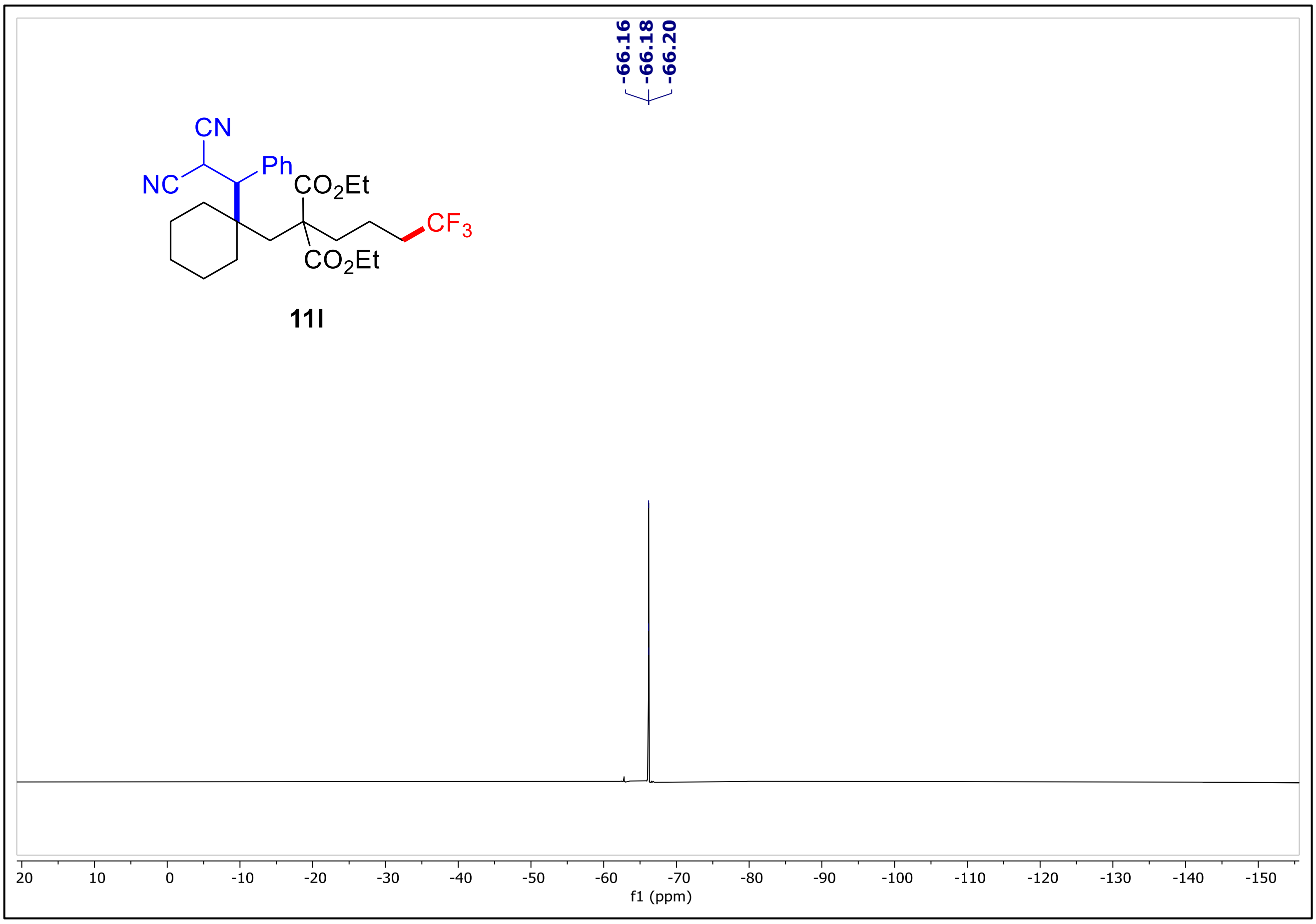

^{19}F NMR of compound **11l** (471 MHz, $CDCl_3$)

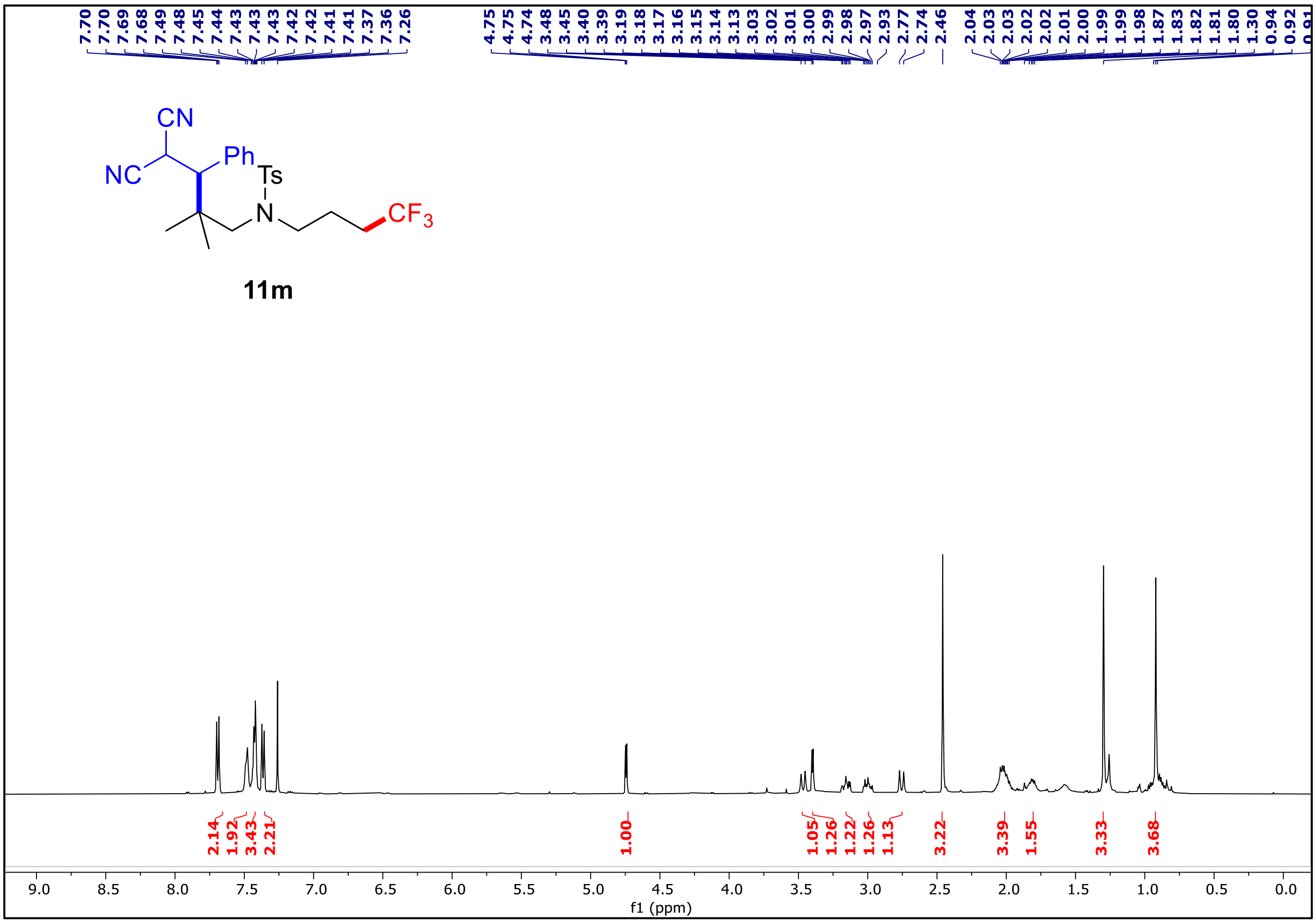

^{1}H NMR of compound **11m** (500 MHz, $CDCl_3$)

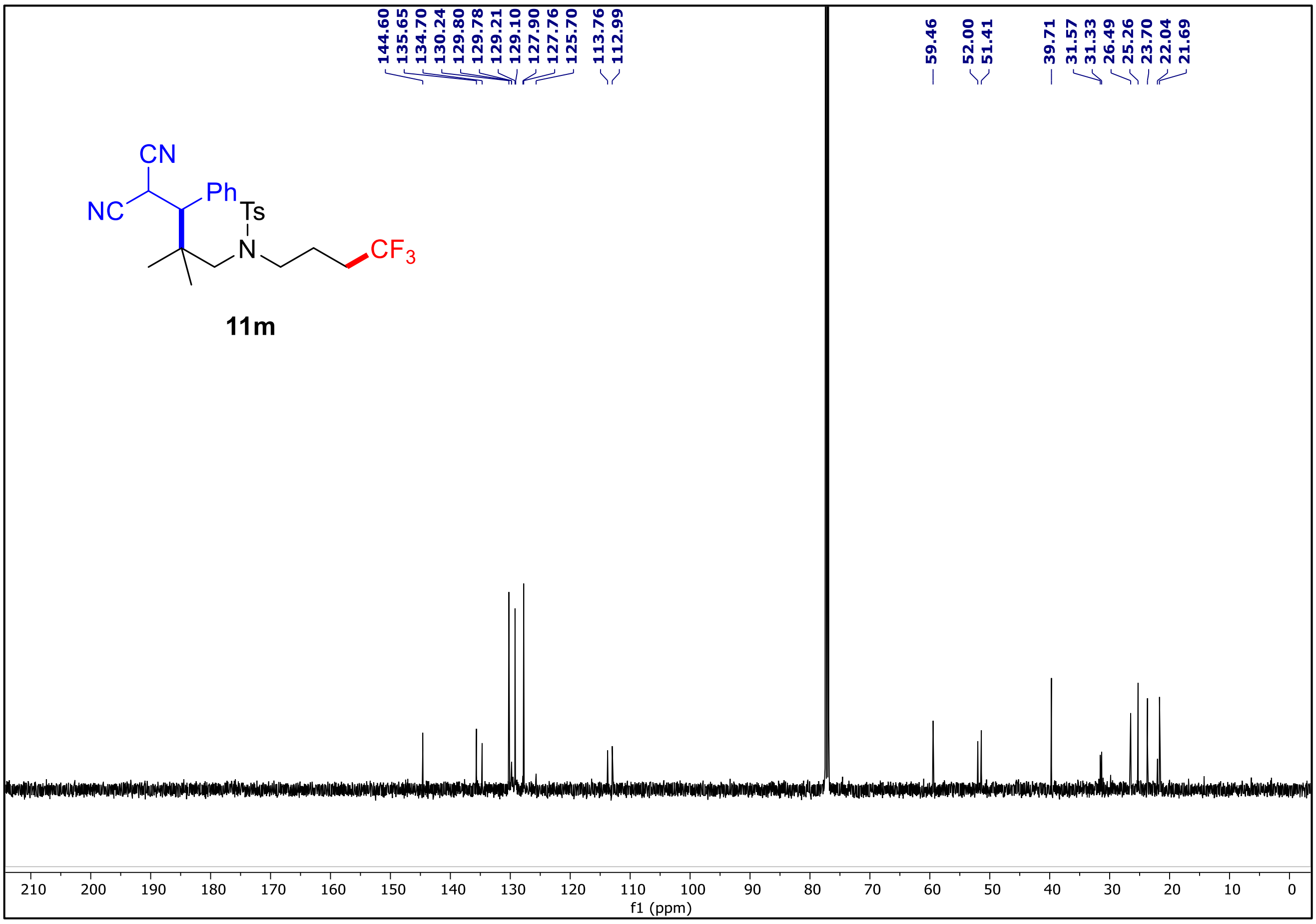

^{13}C{^{1}H} NMR of compound **11m** (126 MHz, $CDCl_3$)

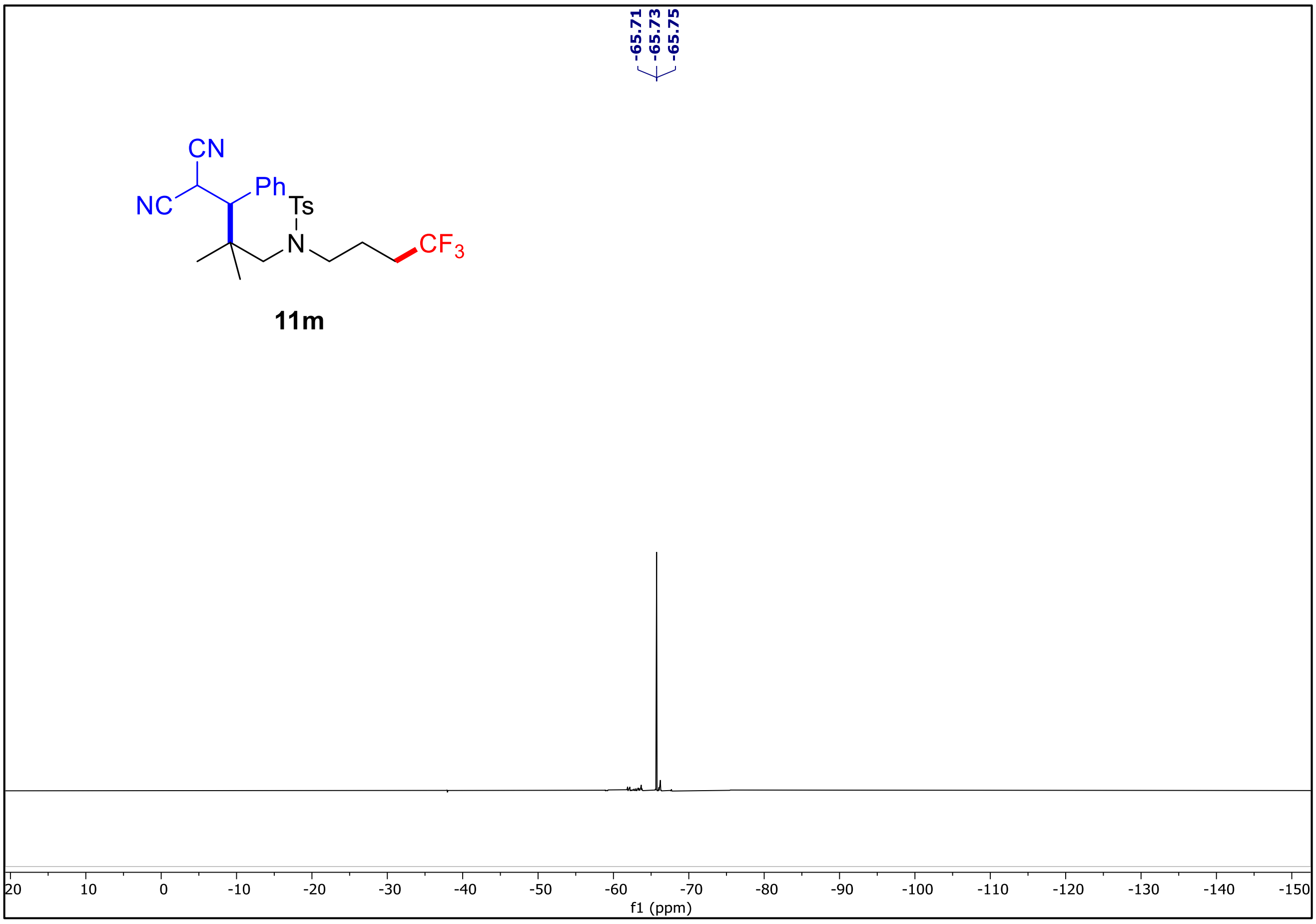

^{19}F NMR of compound **11m** (471 MHz, $CDCl_3$)

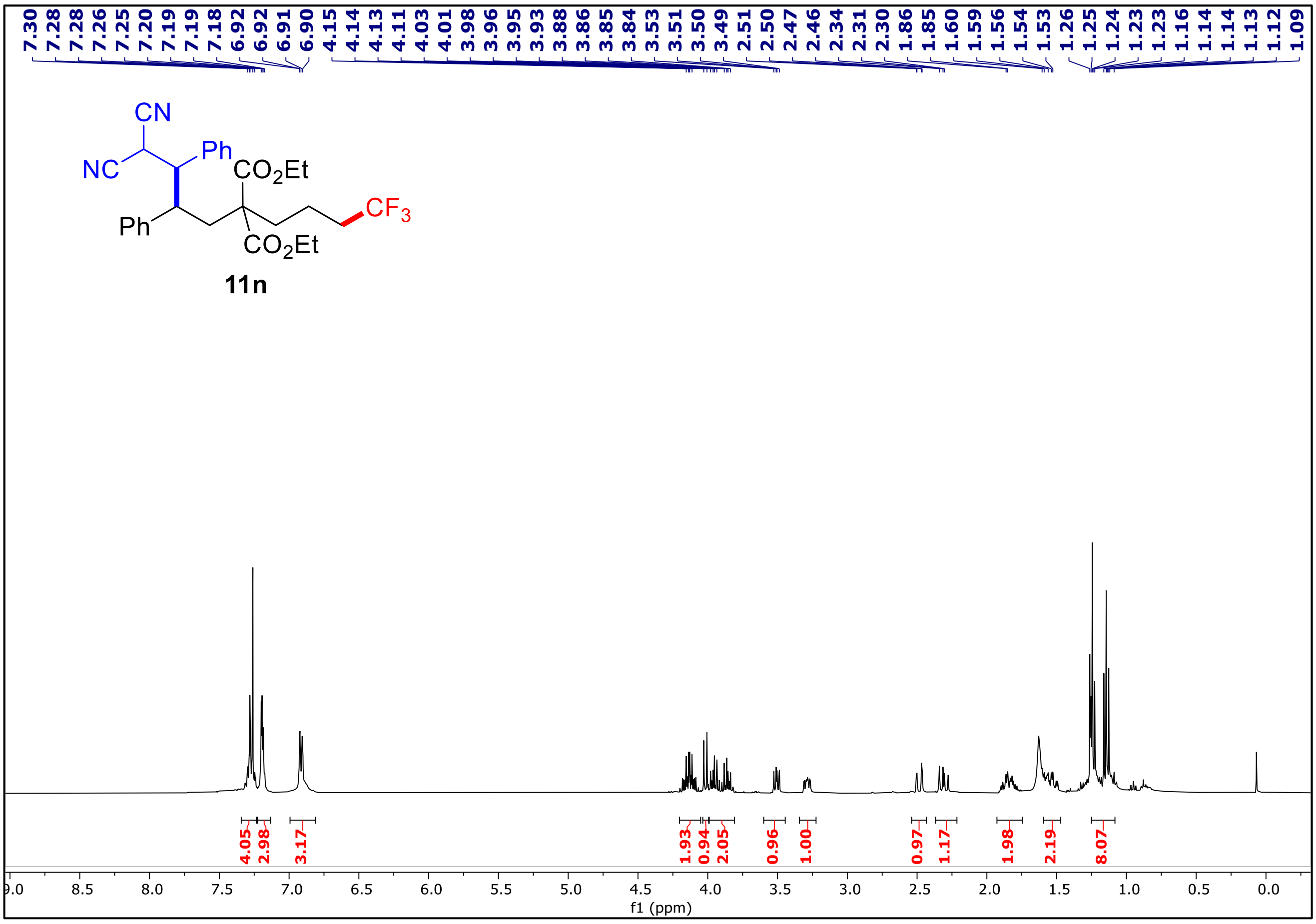

^{1}H NMR of compound **11n′** (400 MHz, $CDCl_3$)

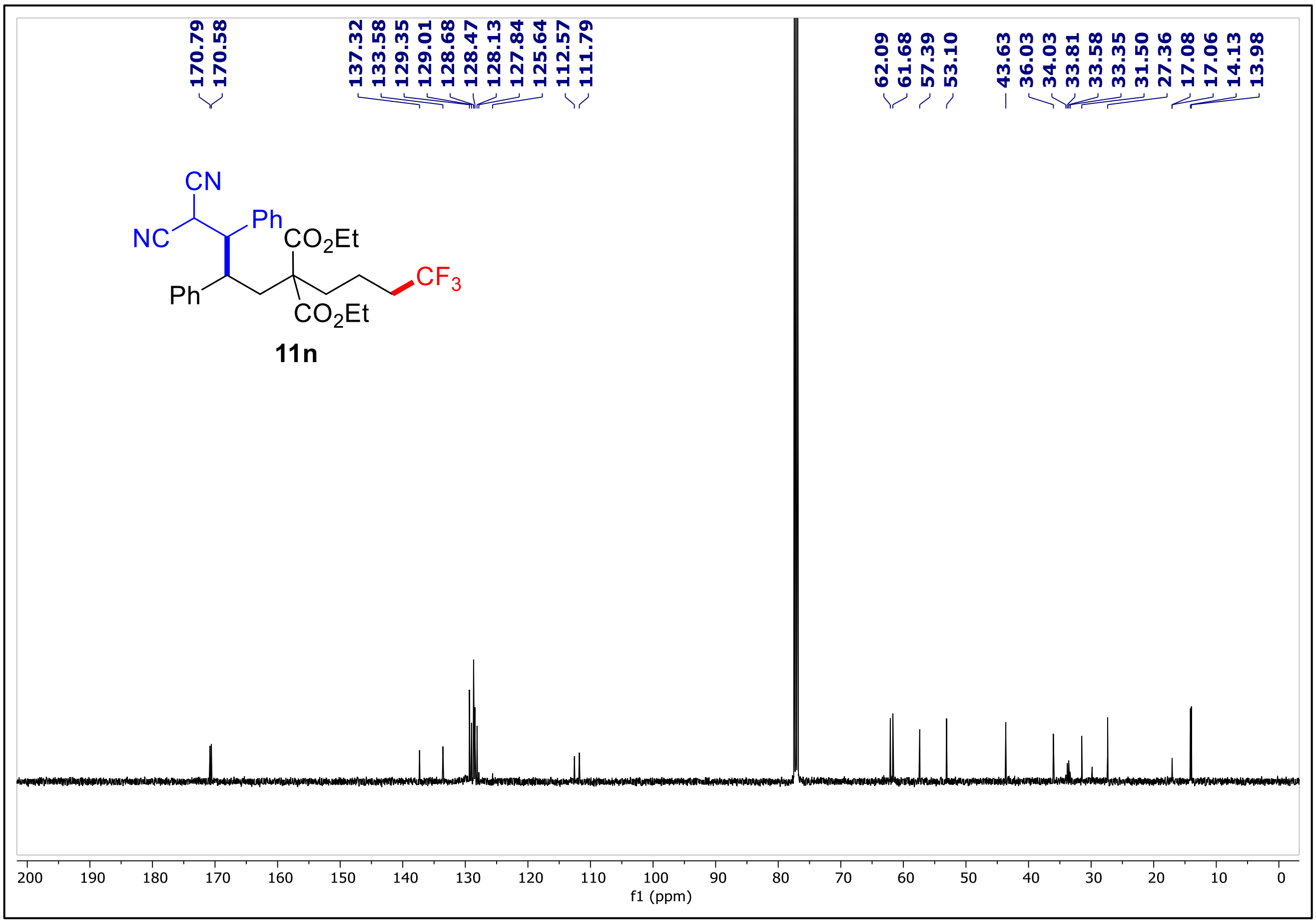

$^{13}C\{^1H\}$ NMR of compound **11n′** (126 MHz, $CDCl_3$)

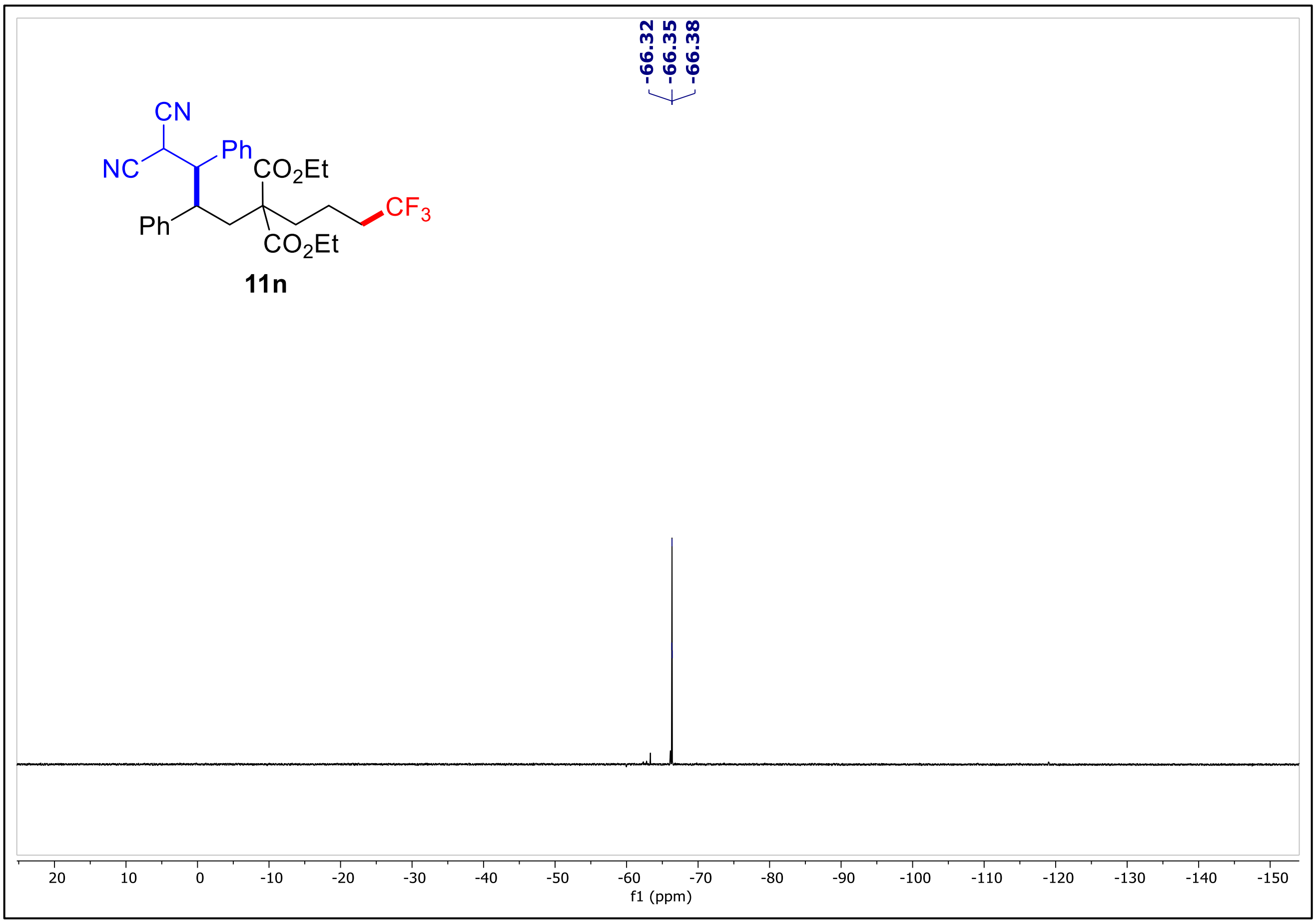

^{19}F NMR of compound **11n′** (376 MHz, $CDCl_3$)

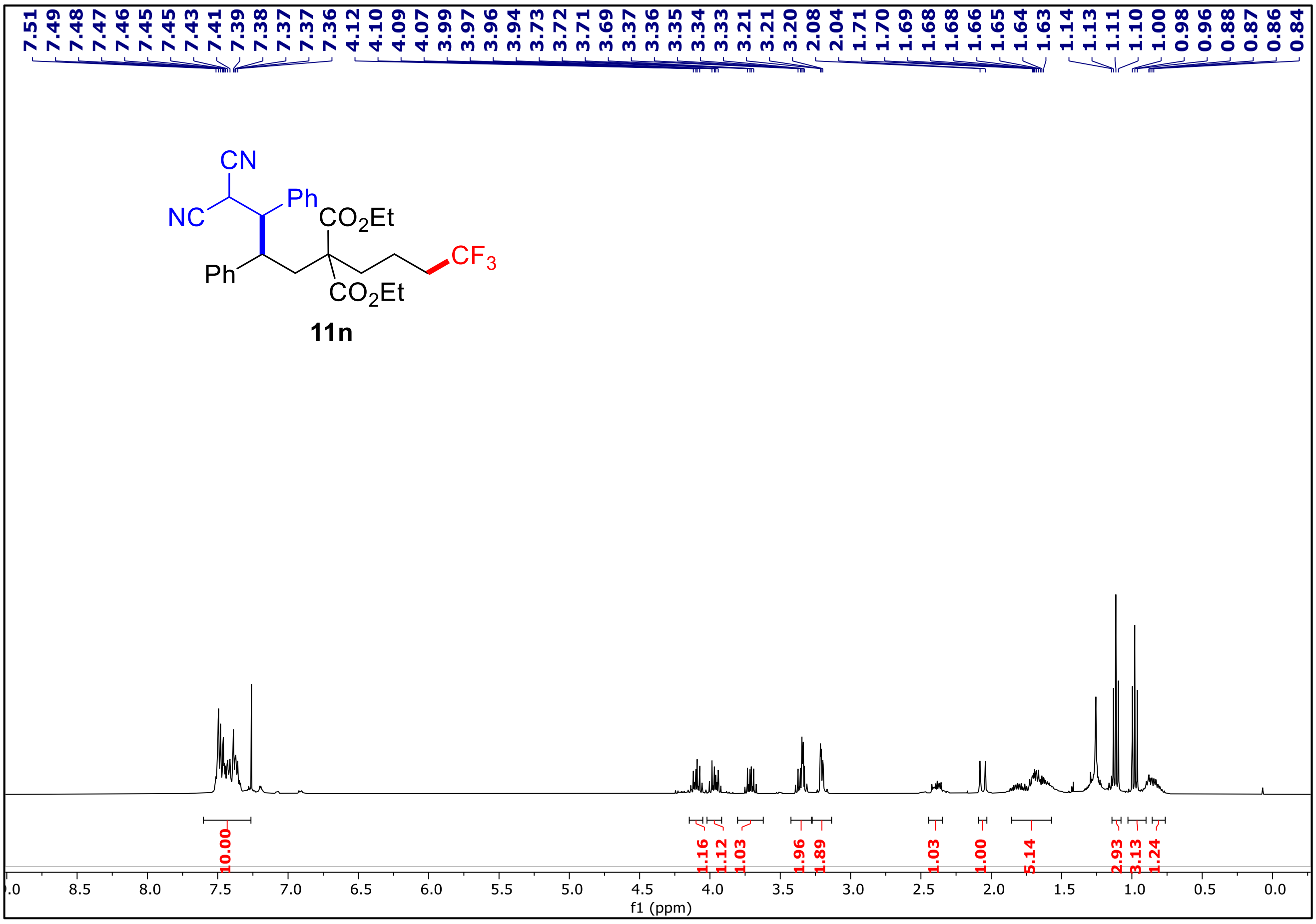

^{1}H NMR of compound **11n″** (400 MHz, CDCl$_3$)

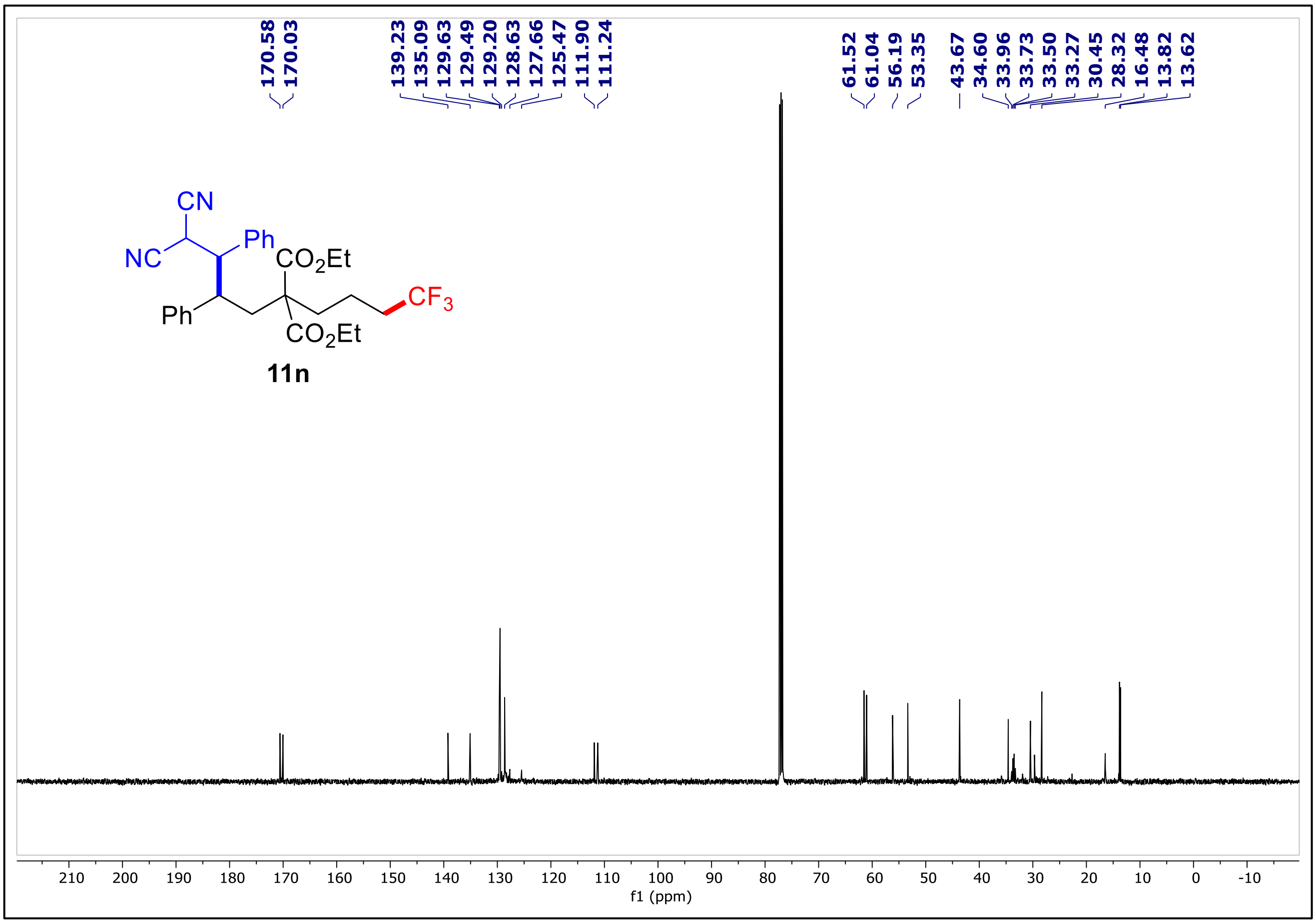

^{13}C{^{1}H} NMR of compound **11n″** (126 MHz, CDCl$_3$)

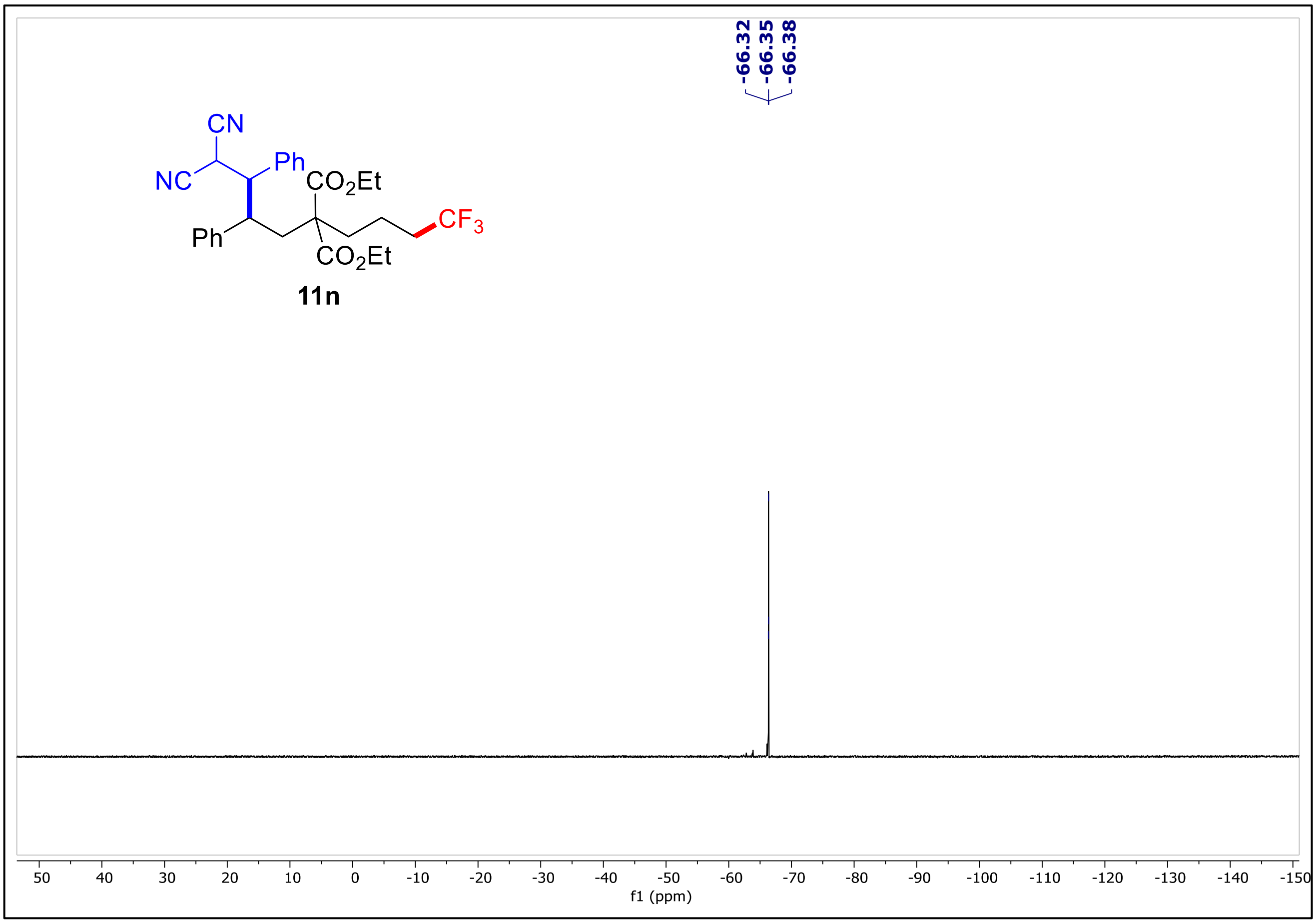

^{19}F NMR of compound **11n″** (376 MHz, $CDCl_3$)

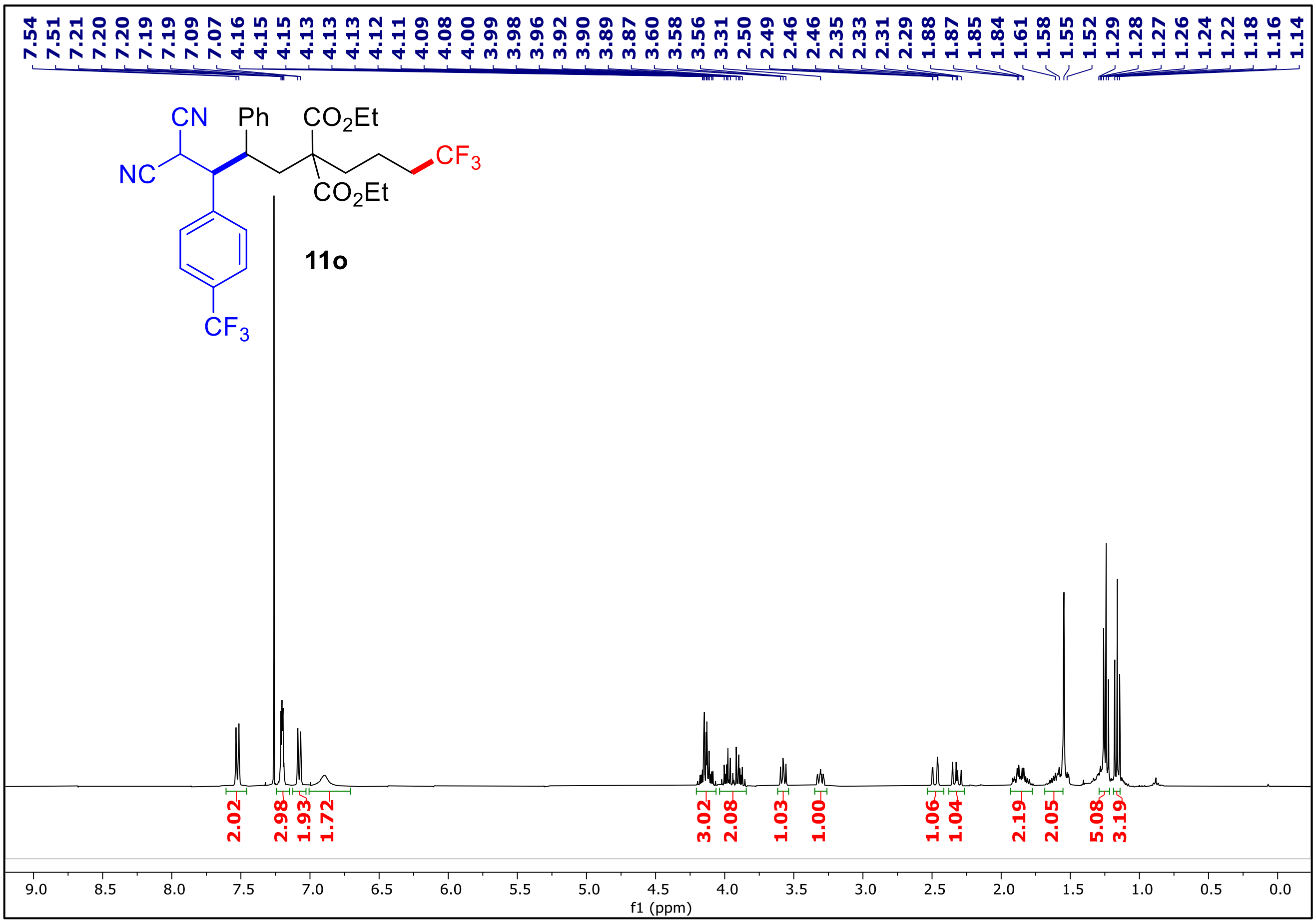

^{1}H NMR of compound **11o′** (400 MHz, $CDCl_3$)

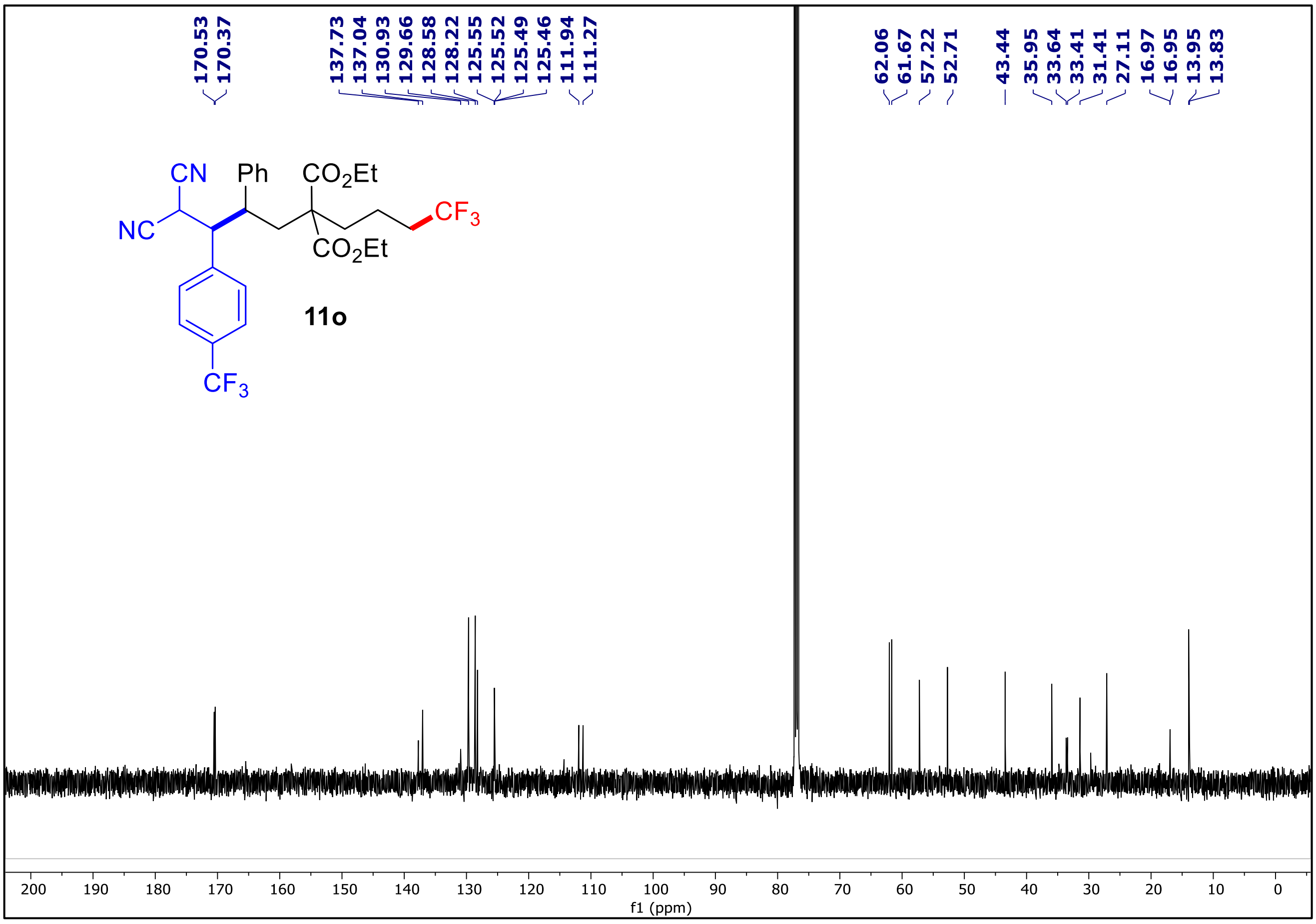

^{13}C{^{1}H} NMR of compound **11o'** (126 MHz, $CDCl_3$)

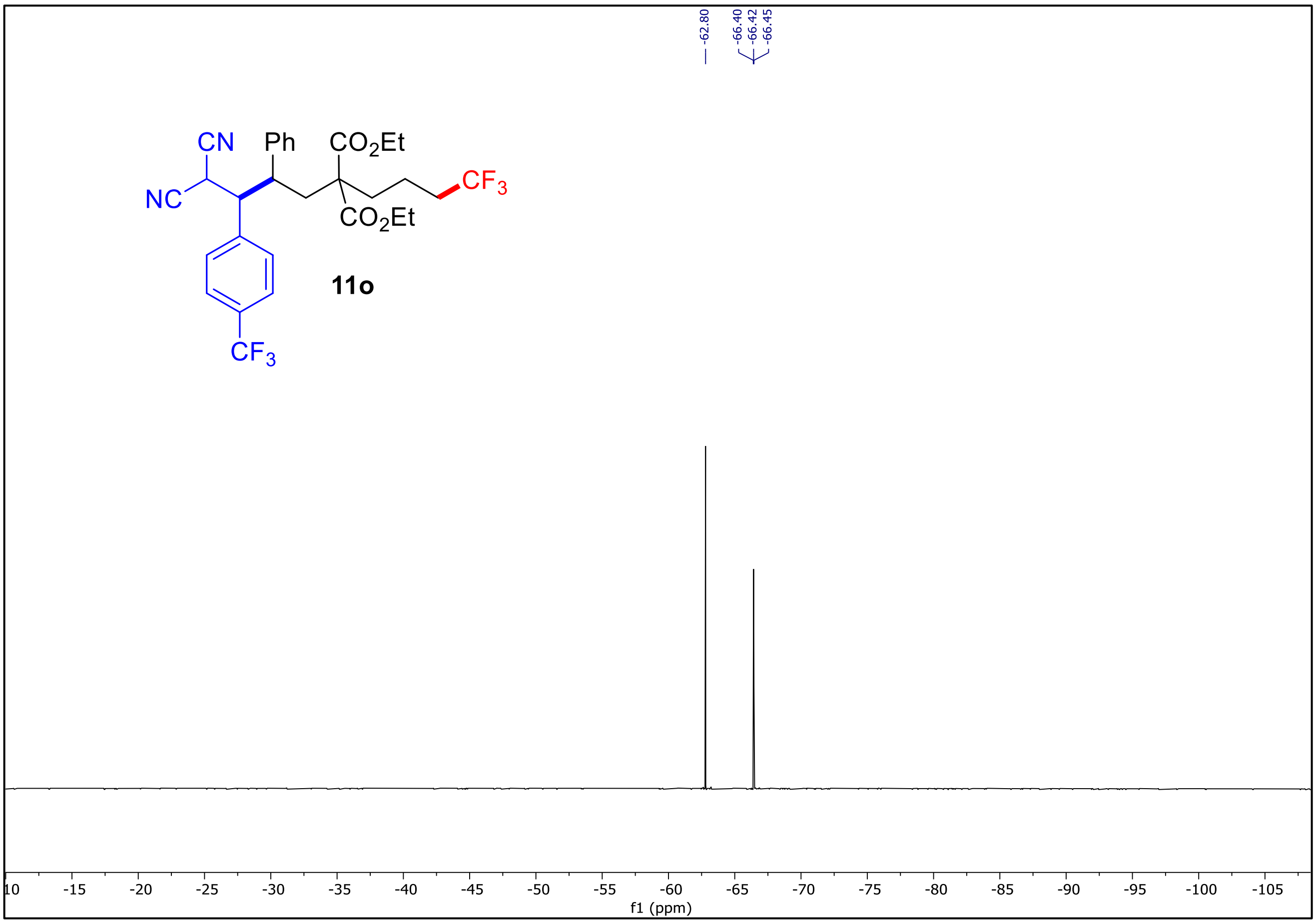

^{19}F NMR of compound **11o'** (471 MHz, $CDCl_3$)

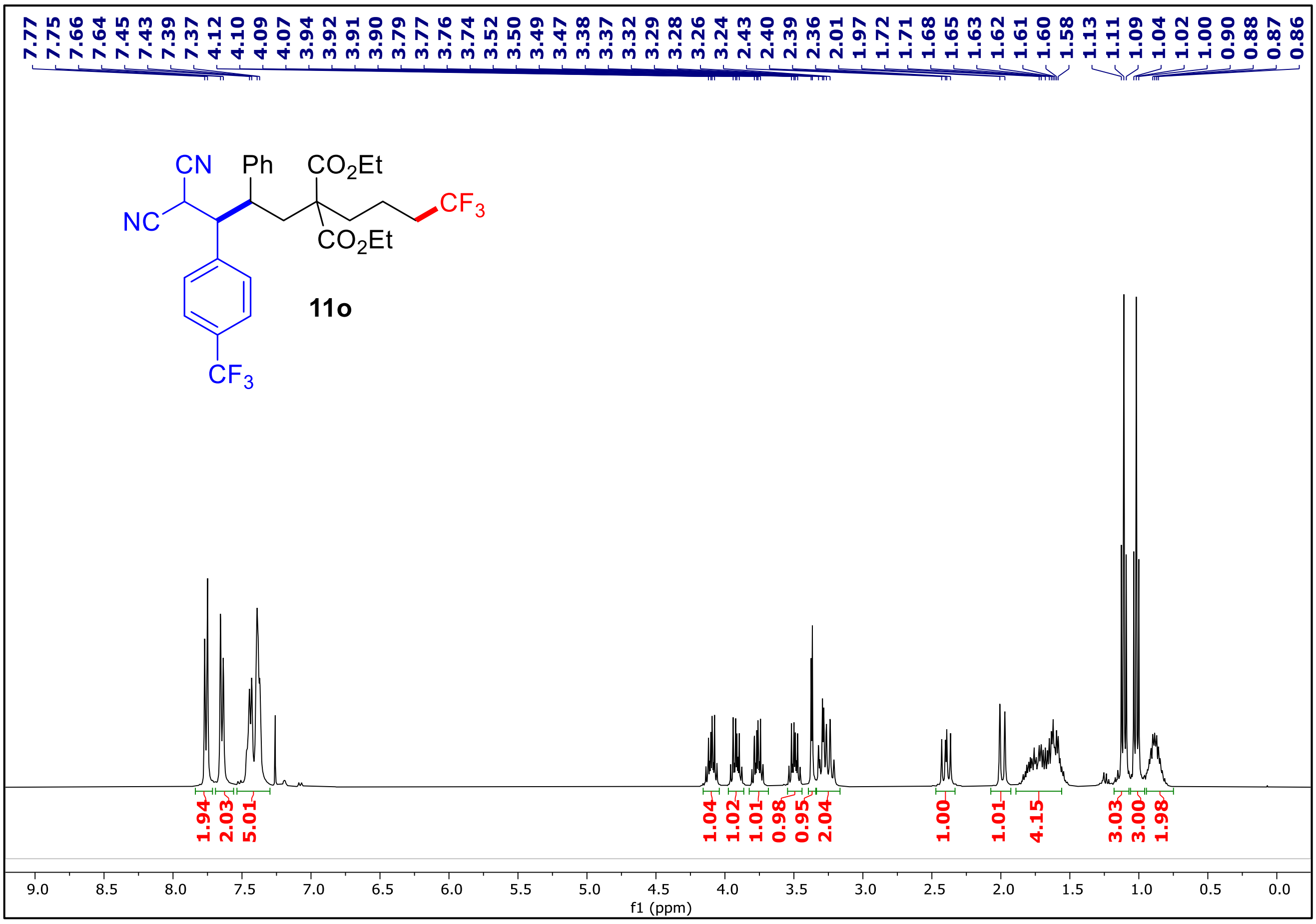

^{1}H NMR of compound **11o”** (400 MHz, $CDCl_3$)

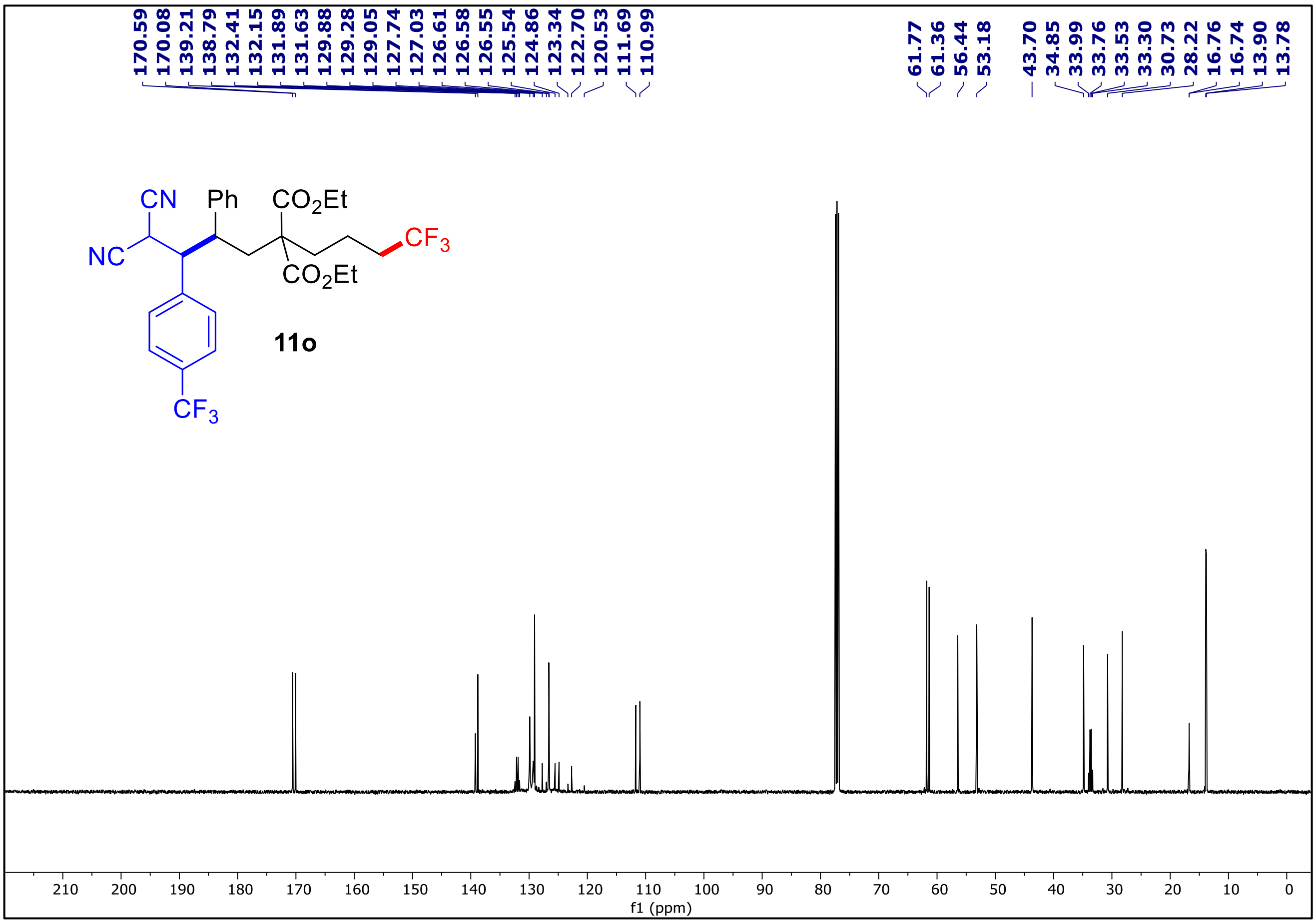

^{13}C{^{1}H} NMR of compound **11o”** (126 MHz, $CDCl_3$)

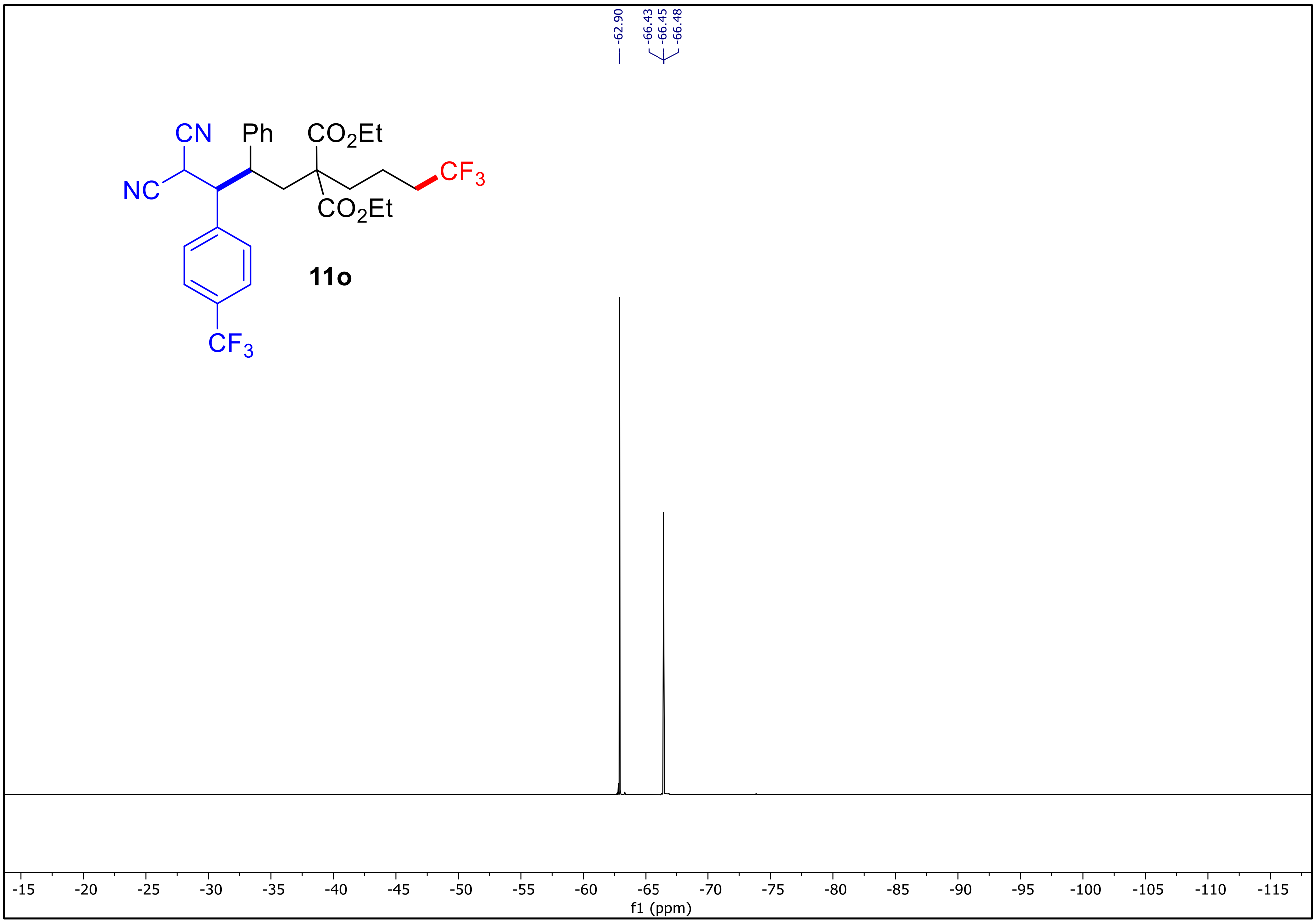

^{19}F NMR of compound **11o″** (471 MHz, $CDCl_3$)

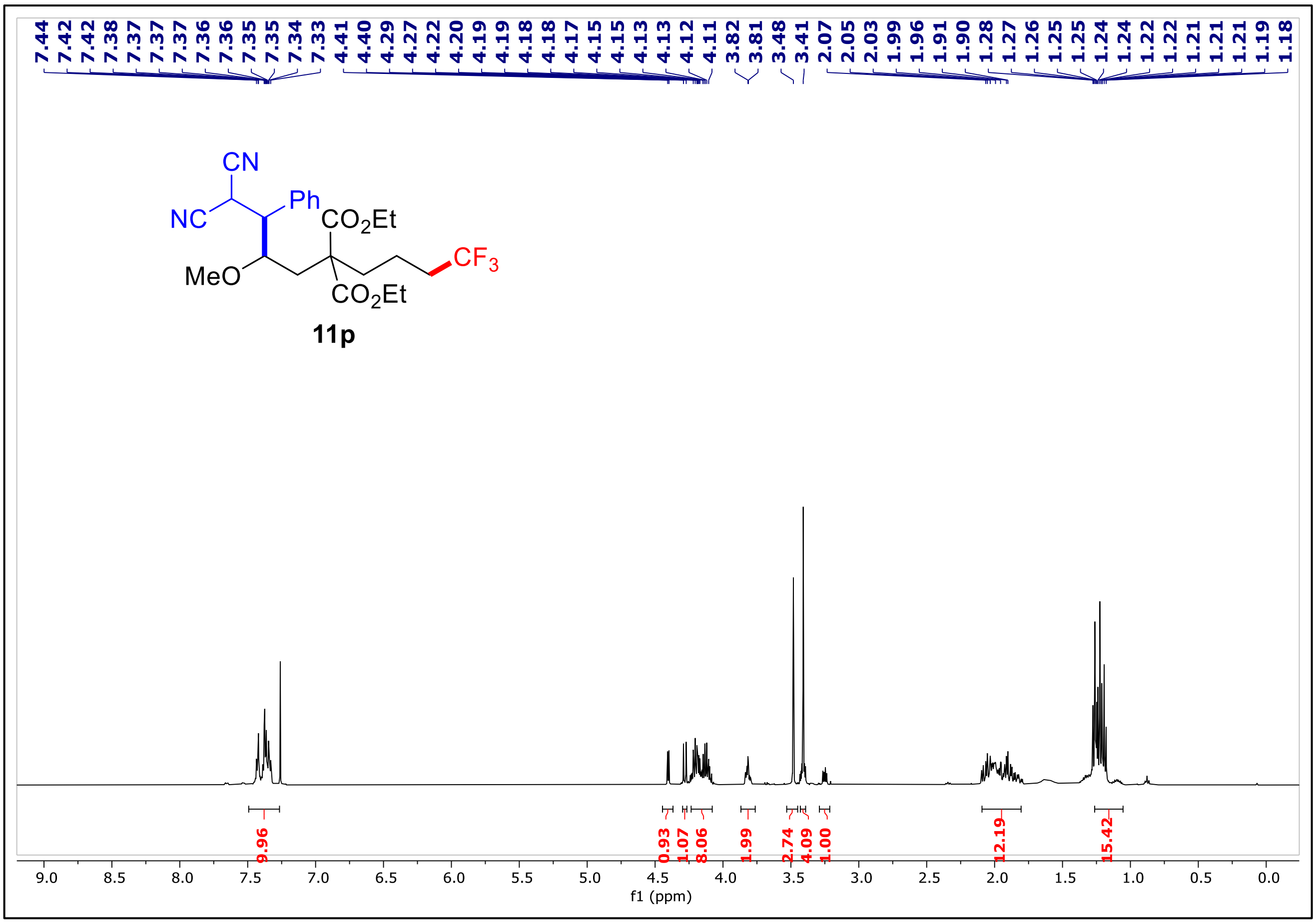

^{1}H NMR of compound **11p** (500 MHz, $CDCl_3$)

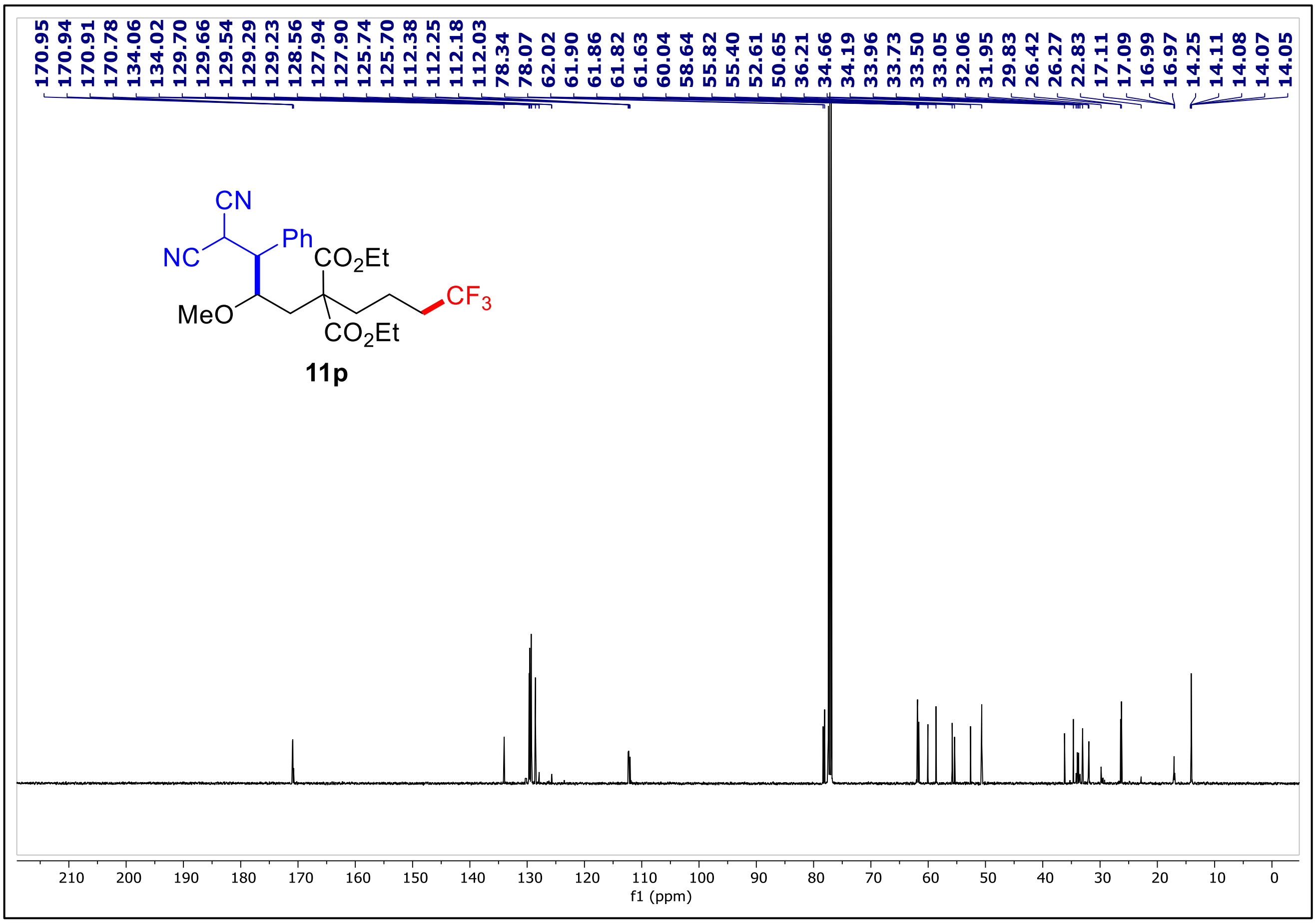

$^{13}C\{^1H\}$ NMR of compound **11p** (126 MHz, $CDCl_3$)

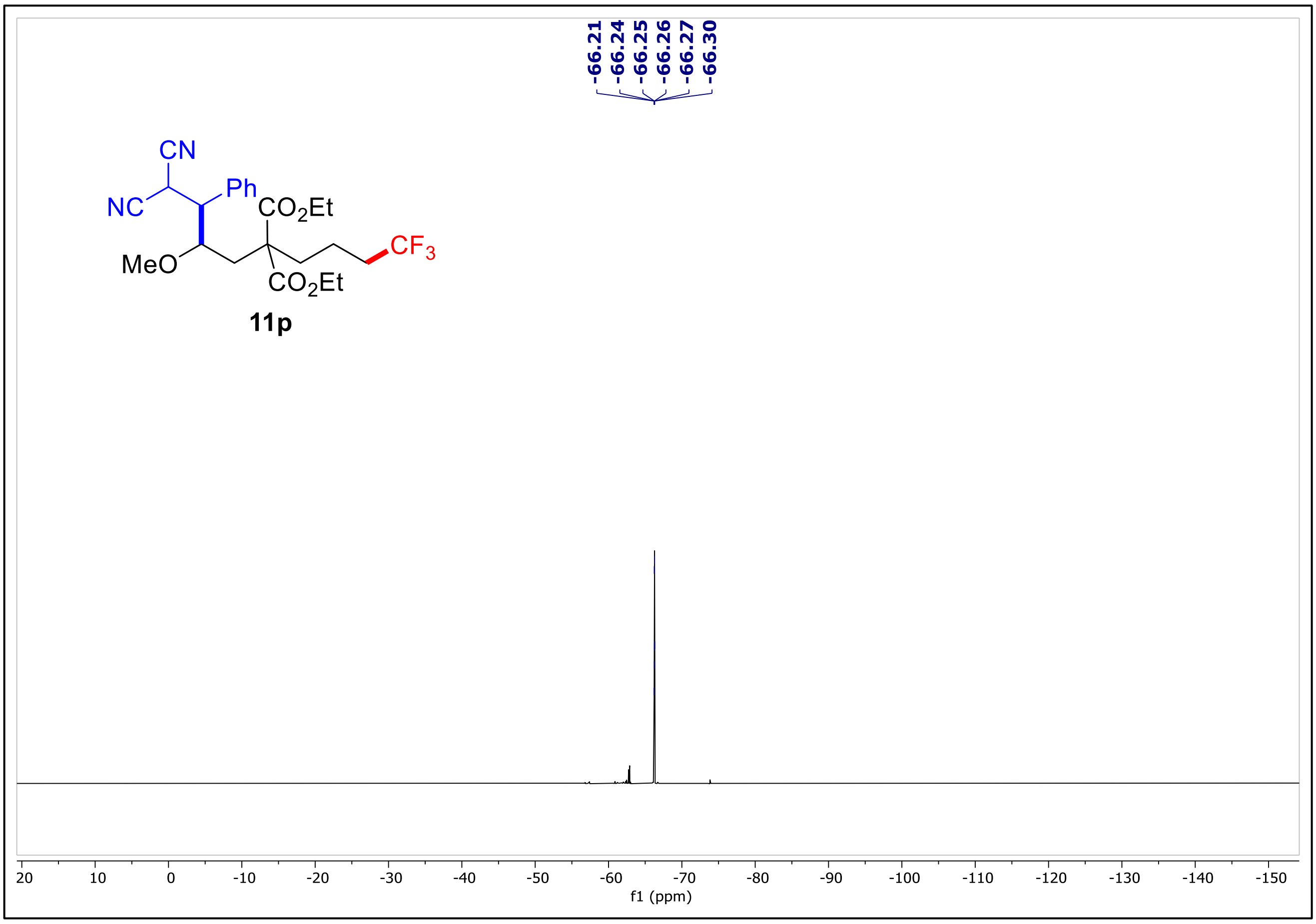

^{19}F NMR of compound **11p** (471 MHz, $CDCl_3$)

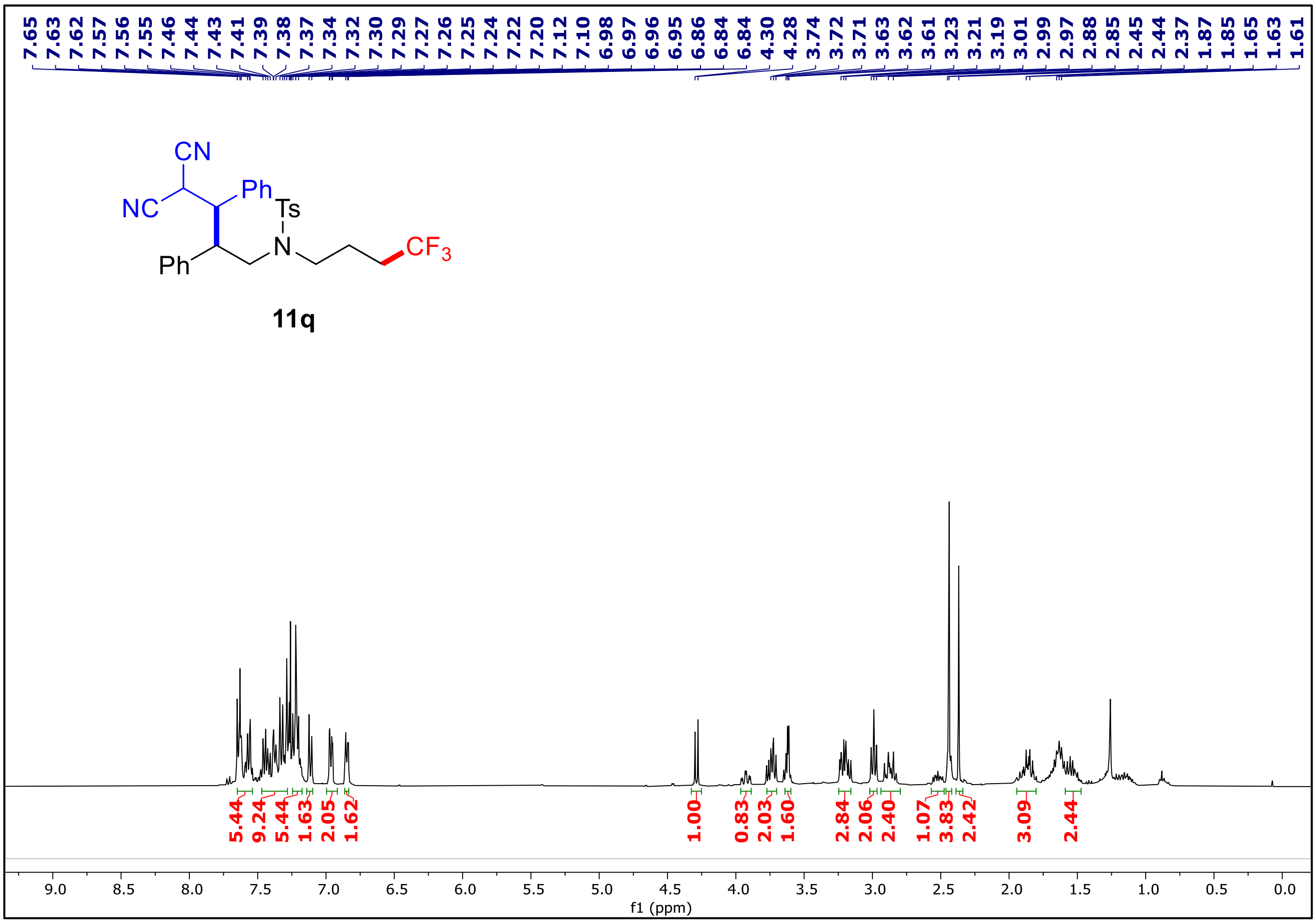

^{1}H NMR of compound **11q** (400 MHz, $CDCl_3$)

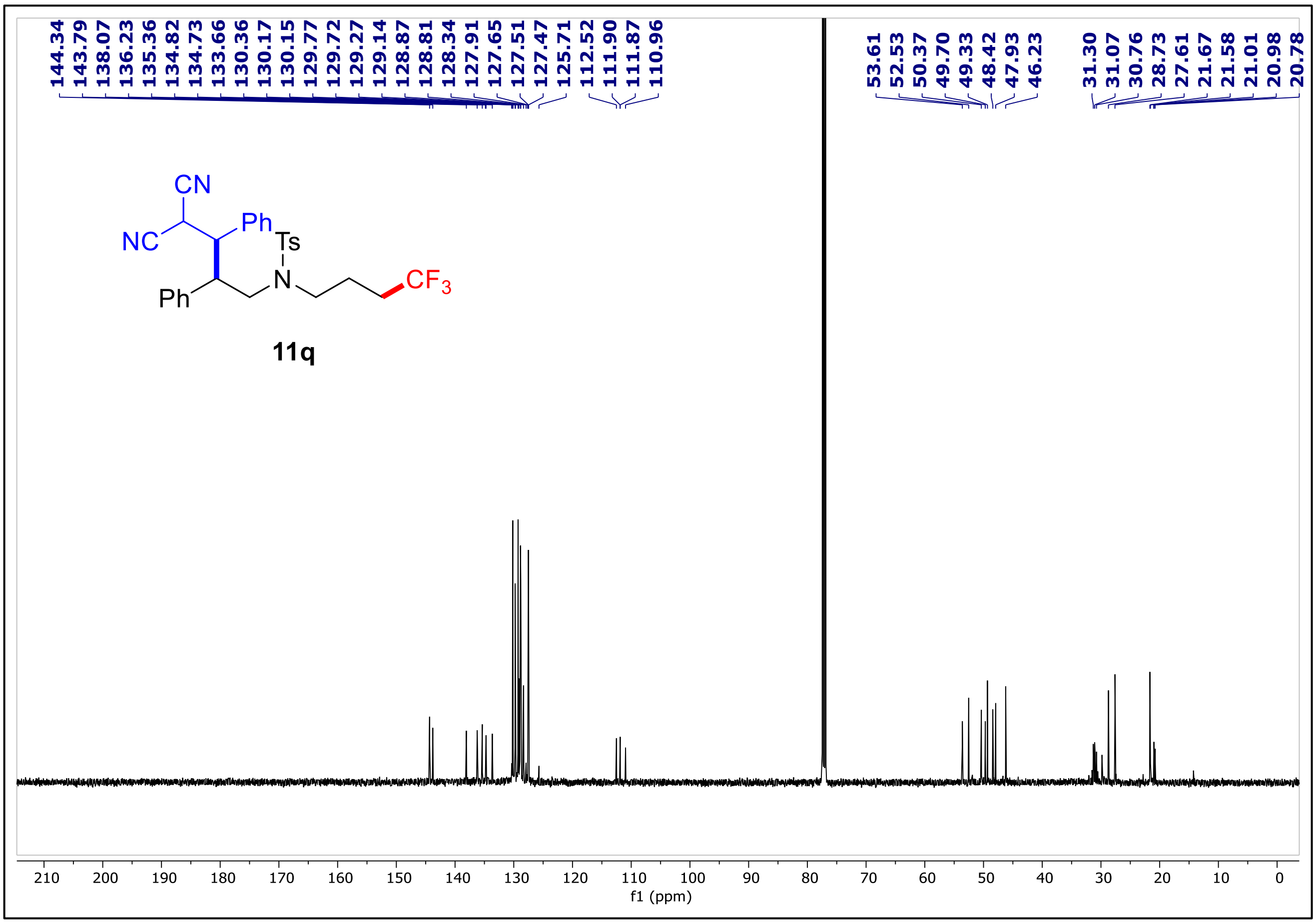

$^{13}C\{^{1}H\}$ NMR of compound **11q** (126 MHz, $CDCl_3$)

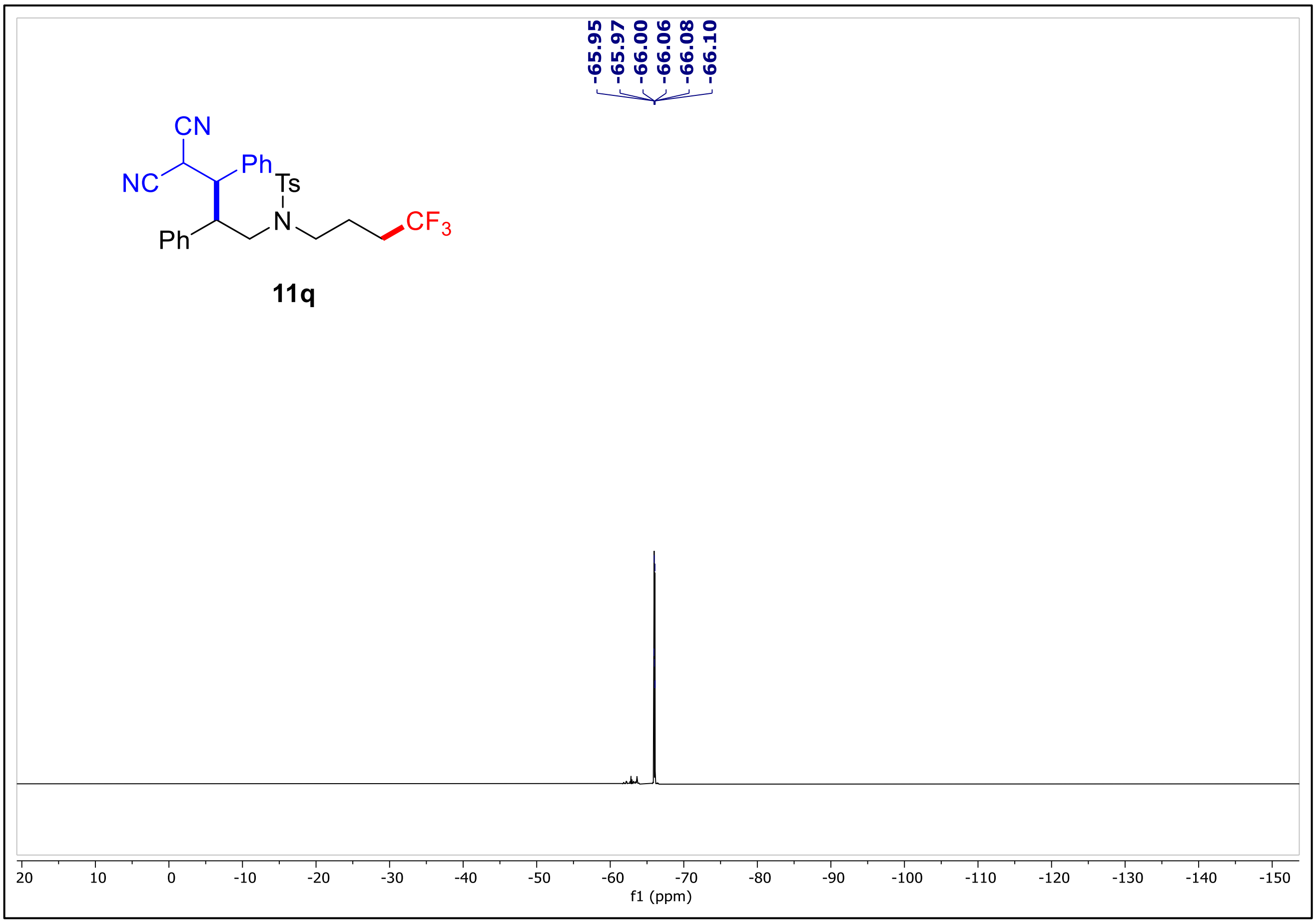

^{19}F NMR of compound **11q** (471 MHz, $CDCl_3$)

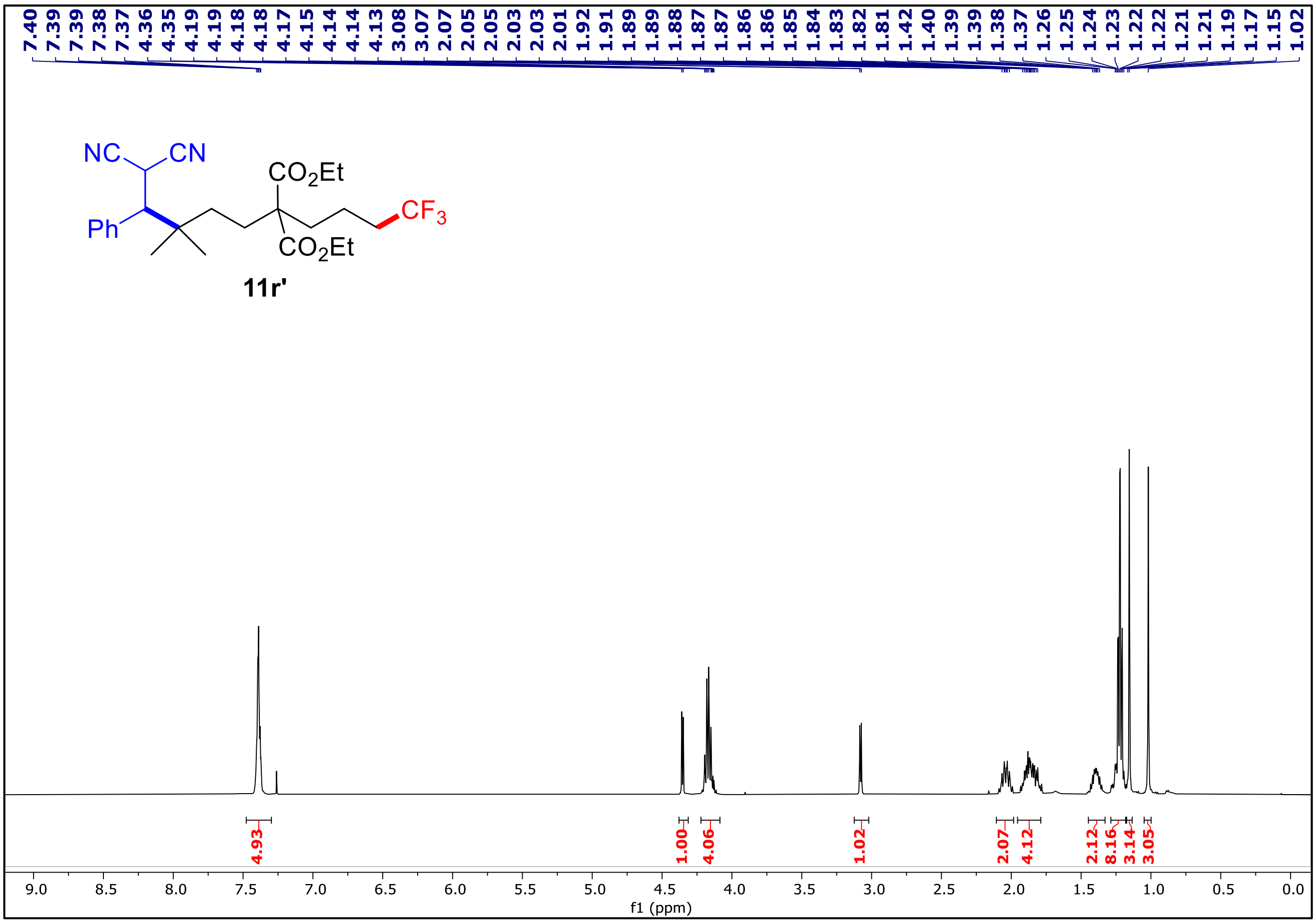

^{1}H NMR of compound **11r′** (500 MHz, $CDCl_3$)

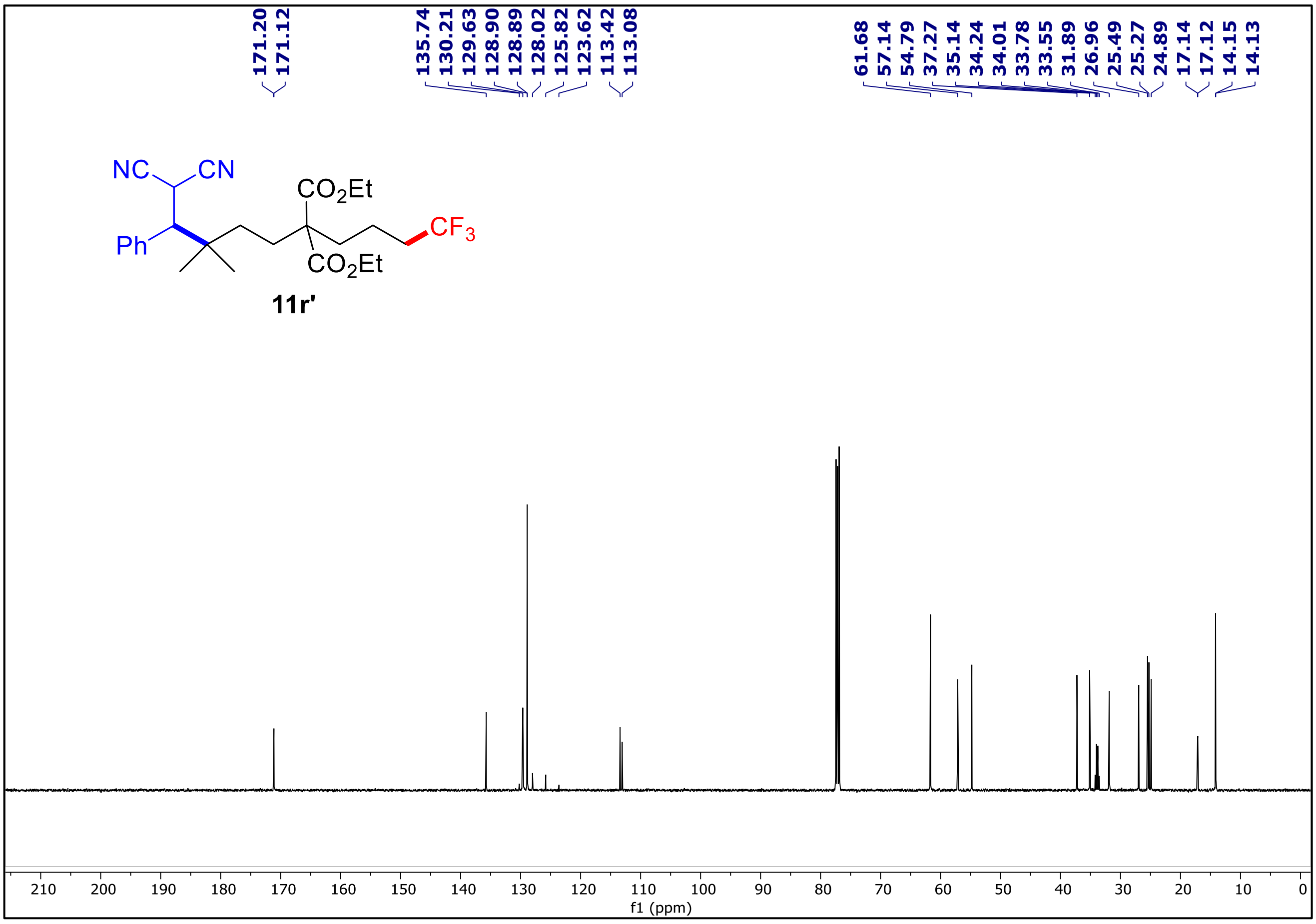

$^{13}C\{^{1}H\}$ NMR of compound **11r′** (126 MHz, $CDCl_3$)

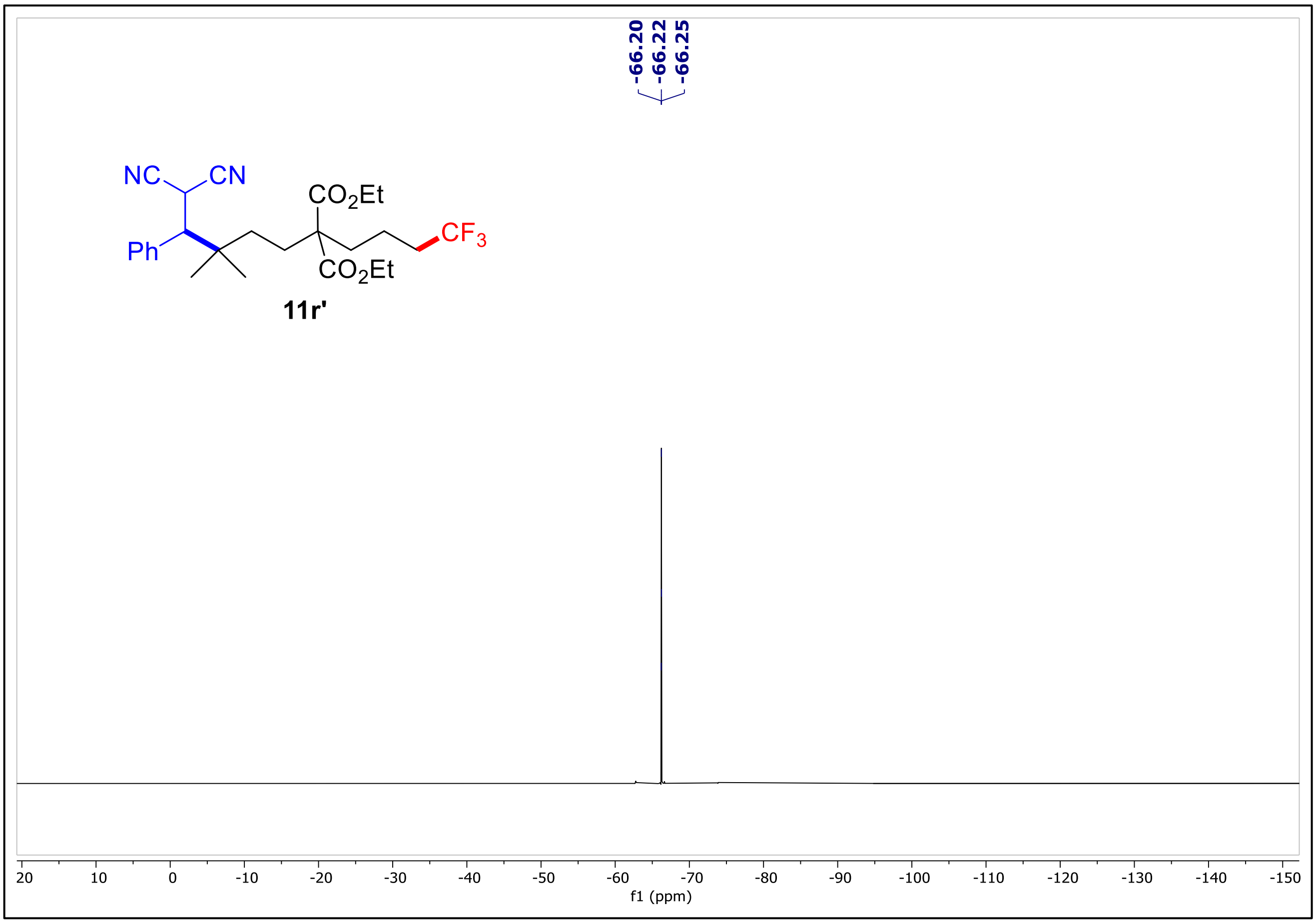

^{19}F NMR of compound **11r′** (471 MHz, $CDCl_3$)

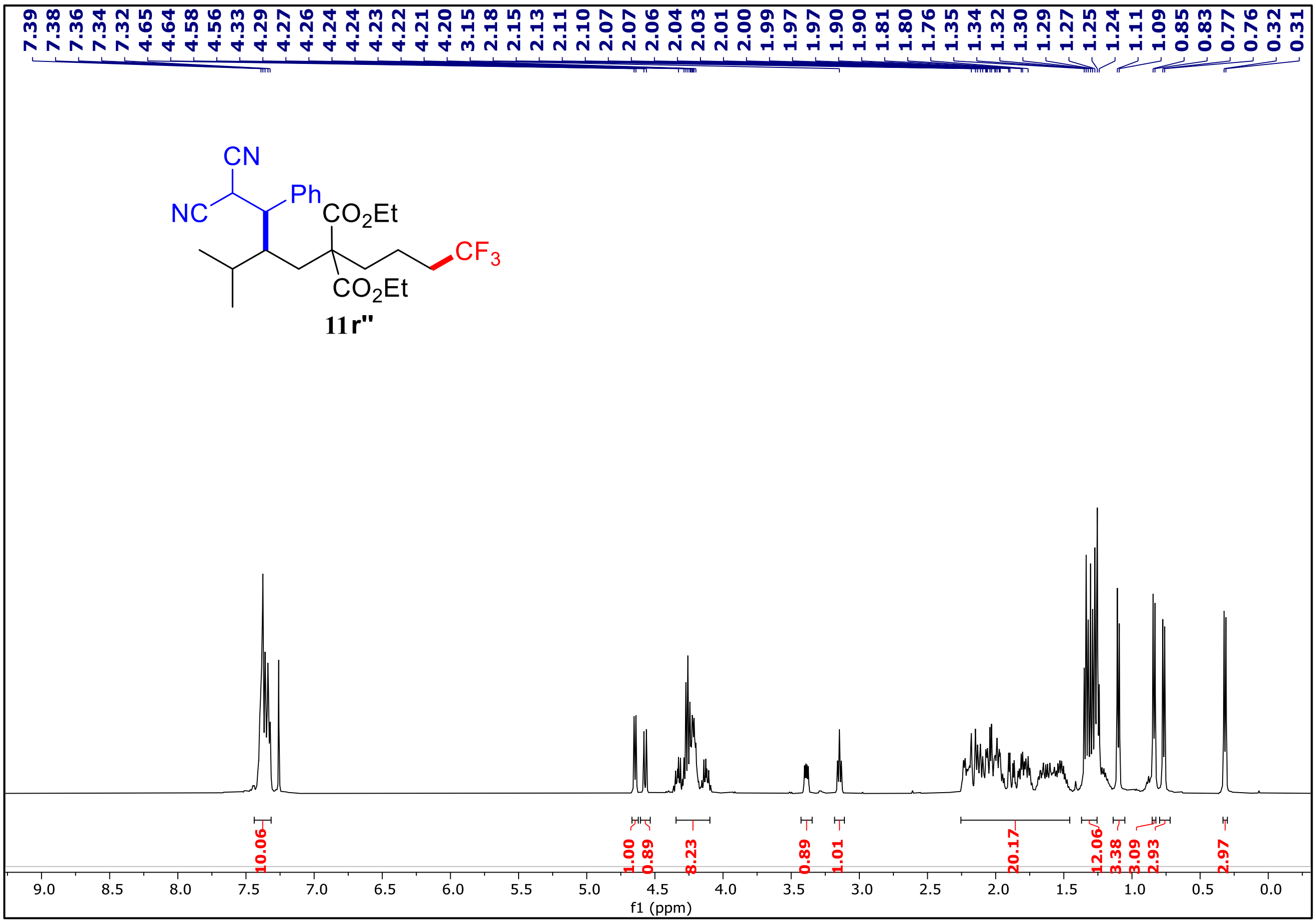

^{1}H NMR of compound **11r''** (500 MHz, $CDCl_3$)

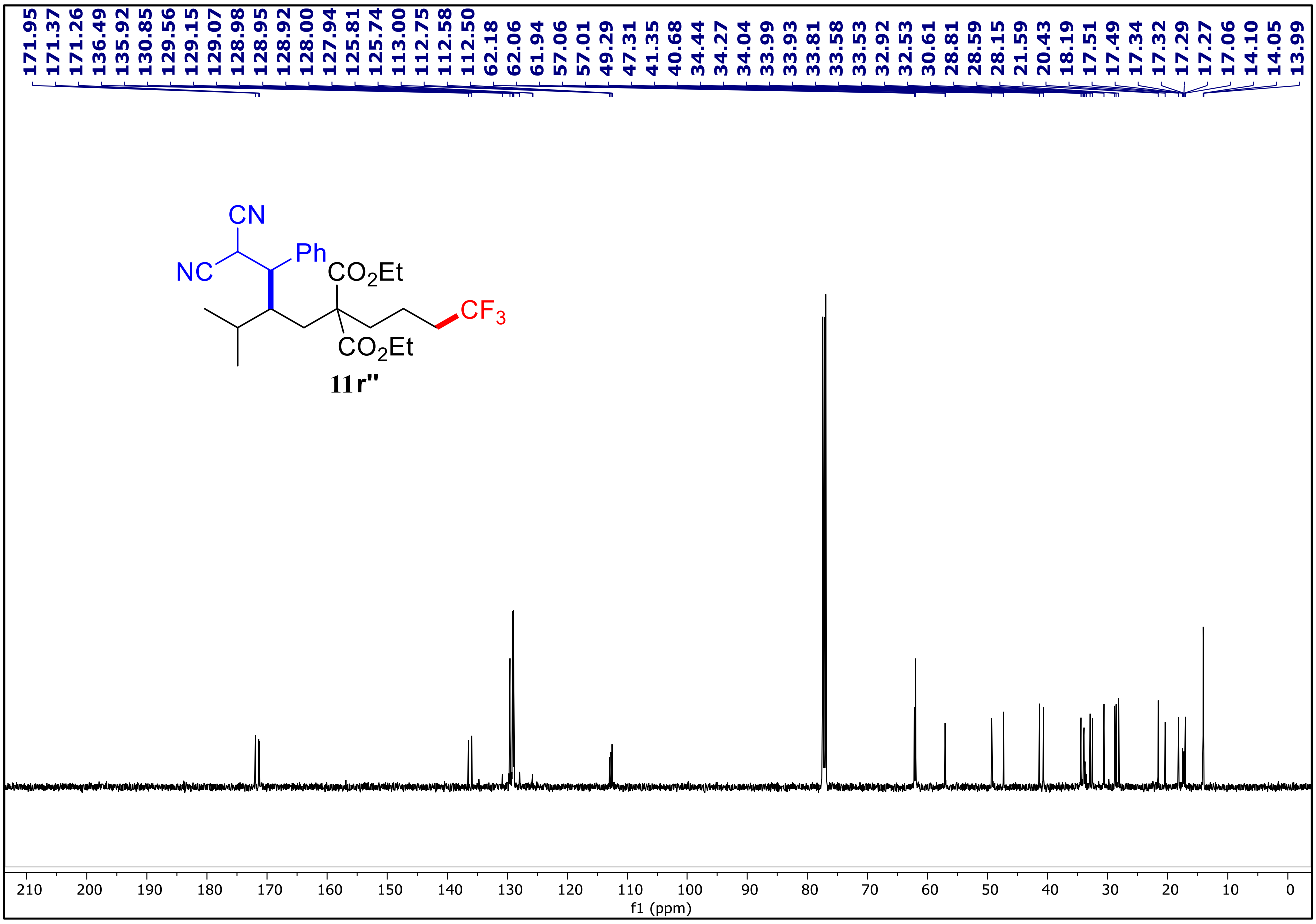

^{13}C{^{1}H} NMR of compound **11r''** (126 MHz, $CDCl_3$)

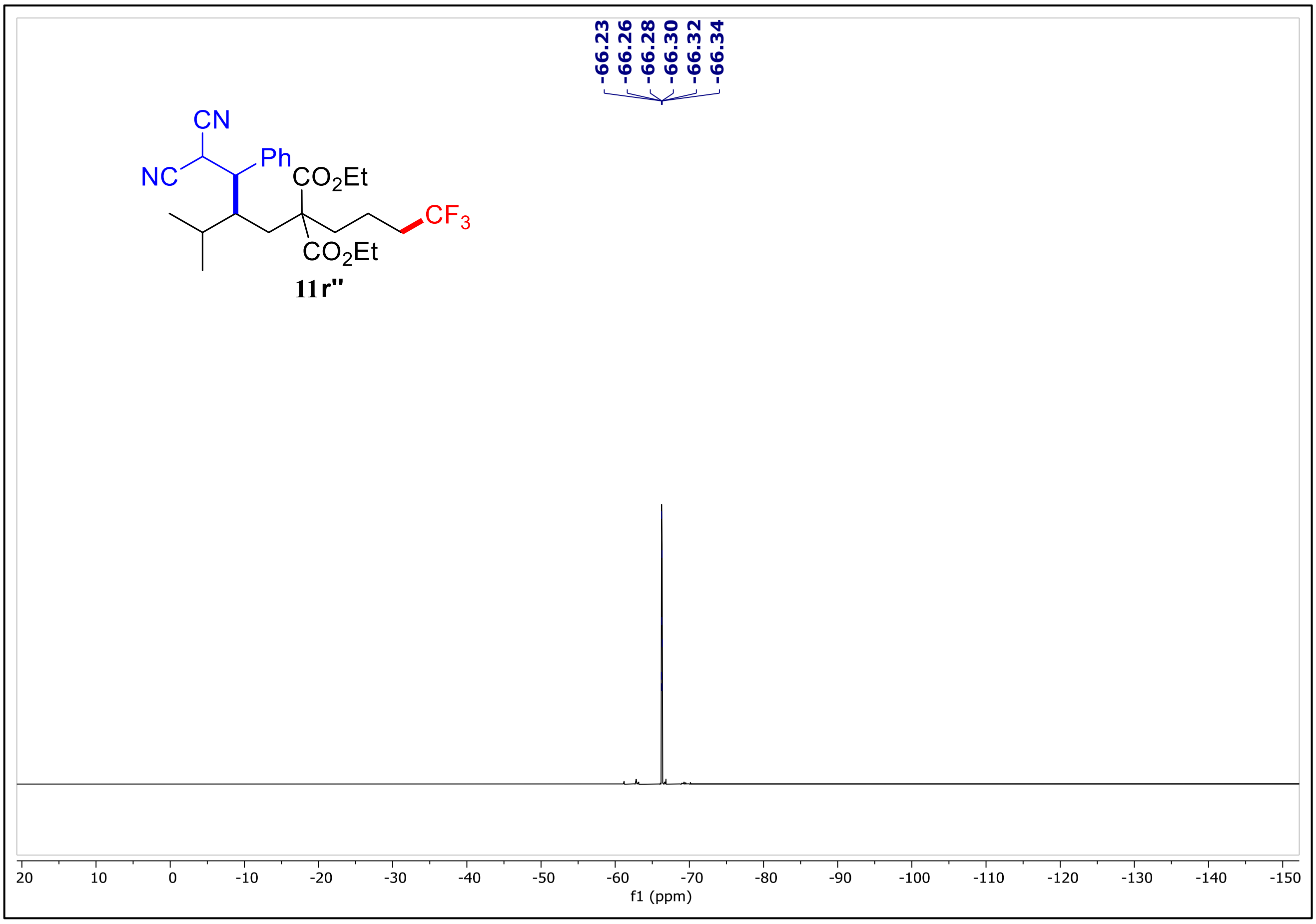

^{19}F NMR of compound **11r''** (471 MHz, $CDCl_3$)

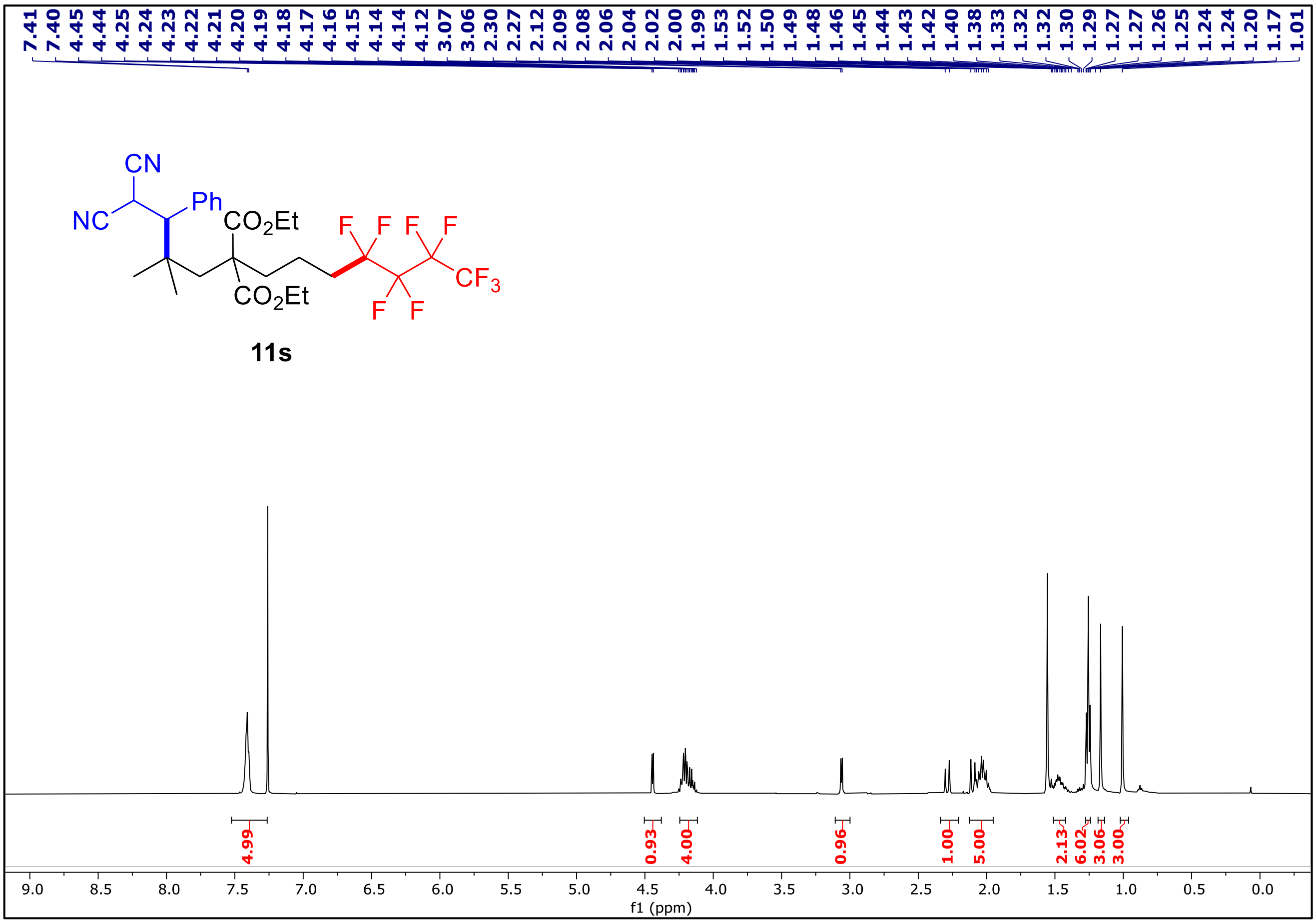

^{1}H NMR of compound **11s** (500 MHz, $CDCl_3$)

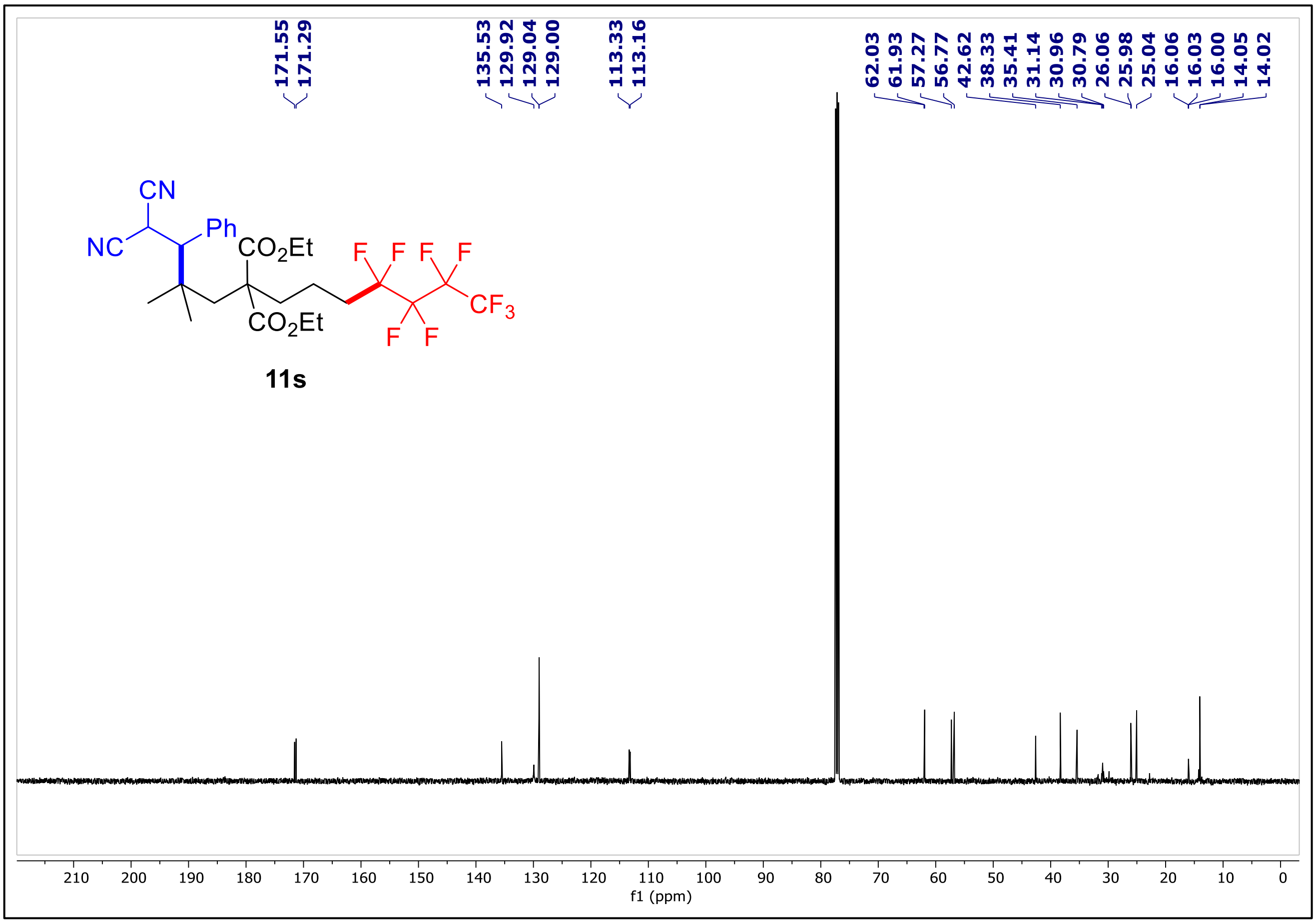

$^{13}C\{^{1}H\}$ NMR of compound **11s** (126 MHz, $CDCl_3$)

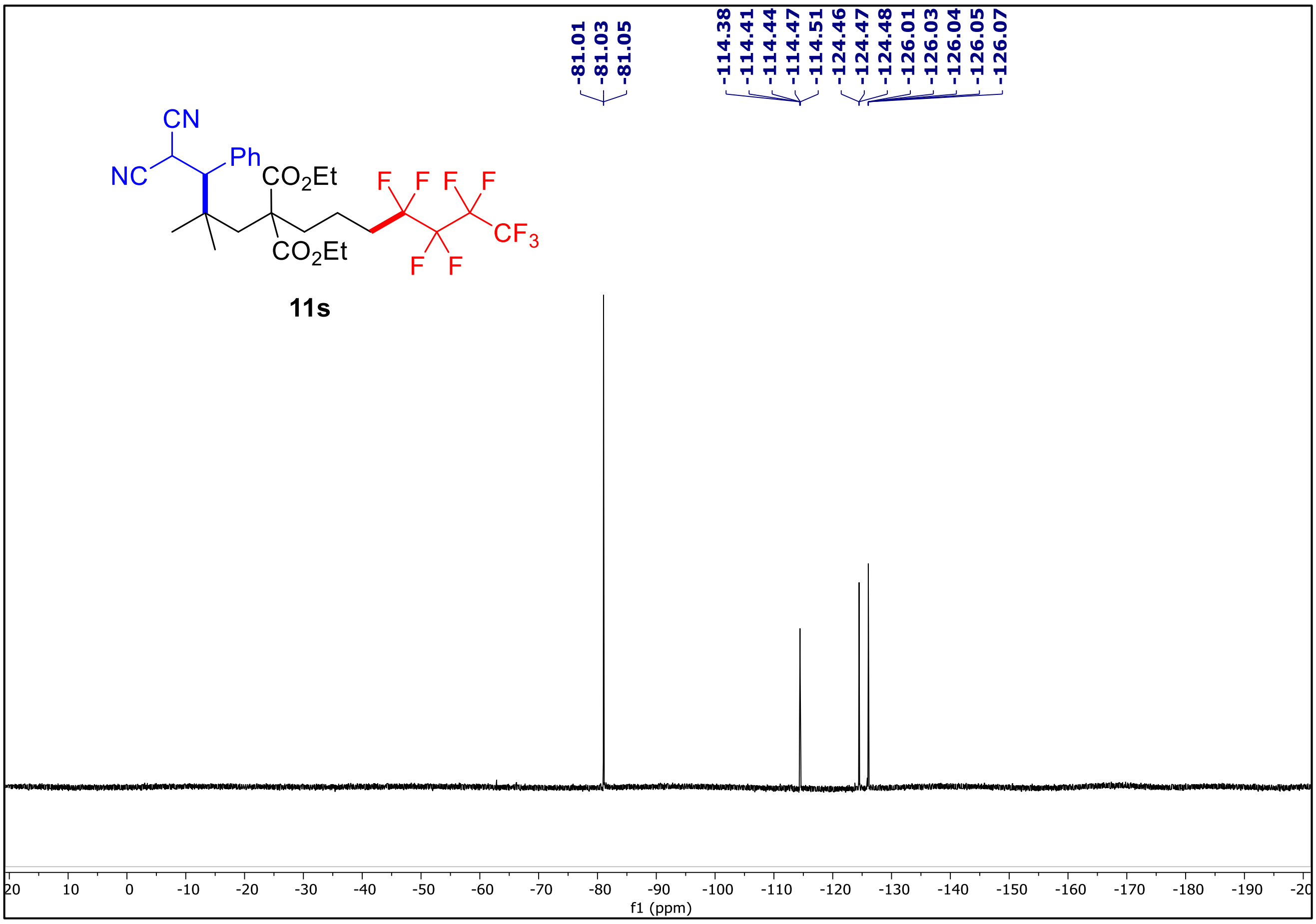

^{19}F NMR of compound **11s** (471 MHz, $CDCl_3$)

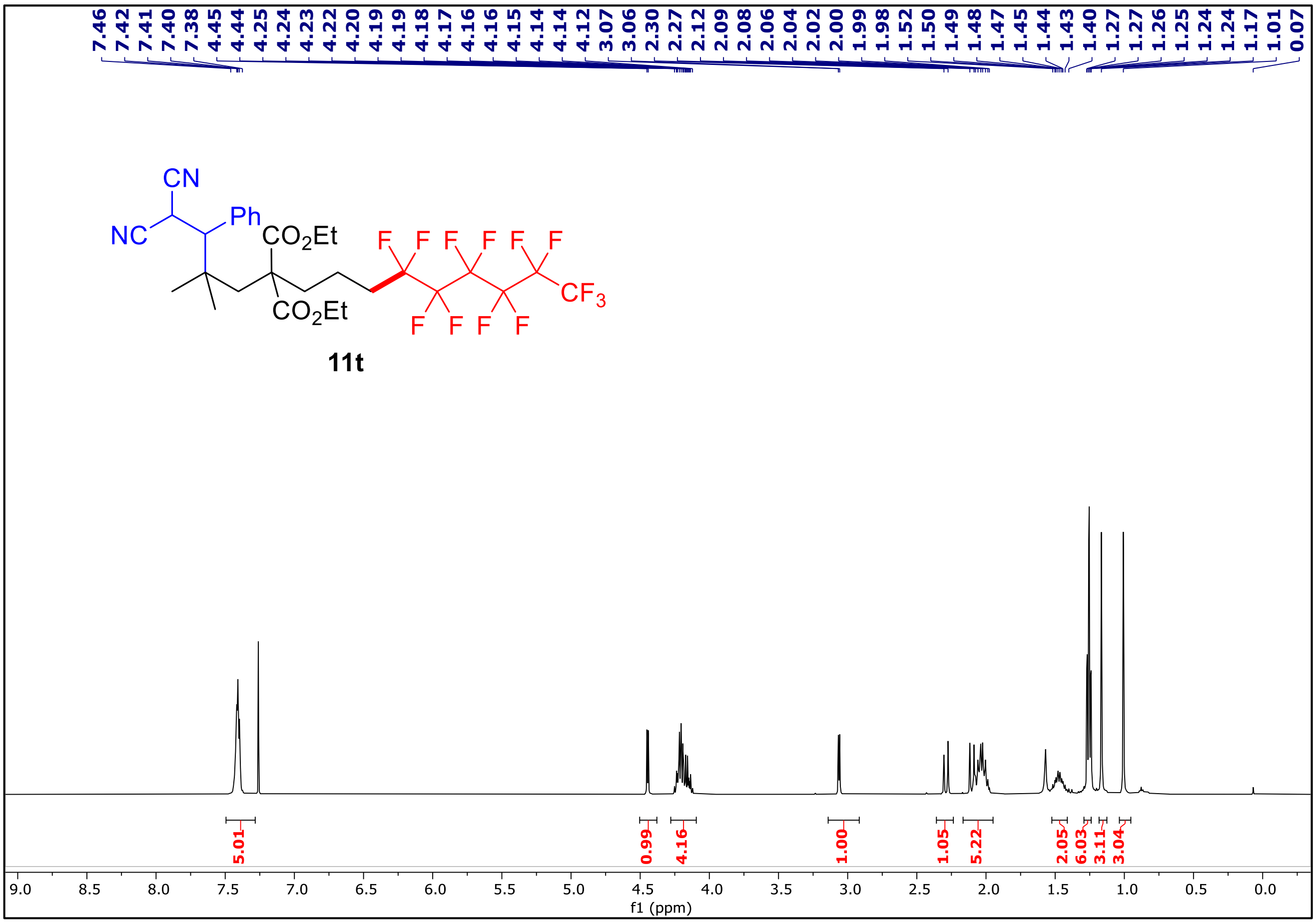

^{1}H NMR of compound **11t** (500 MHz, $CDCl_3$)

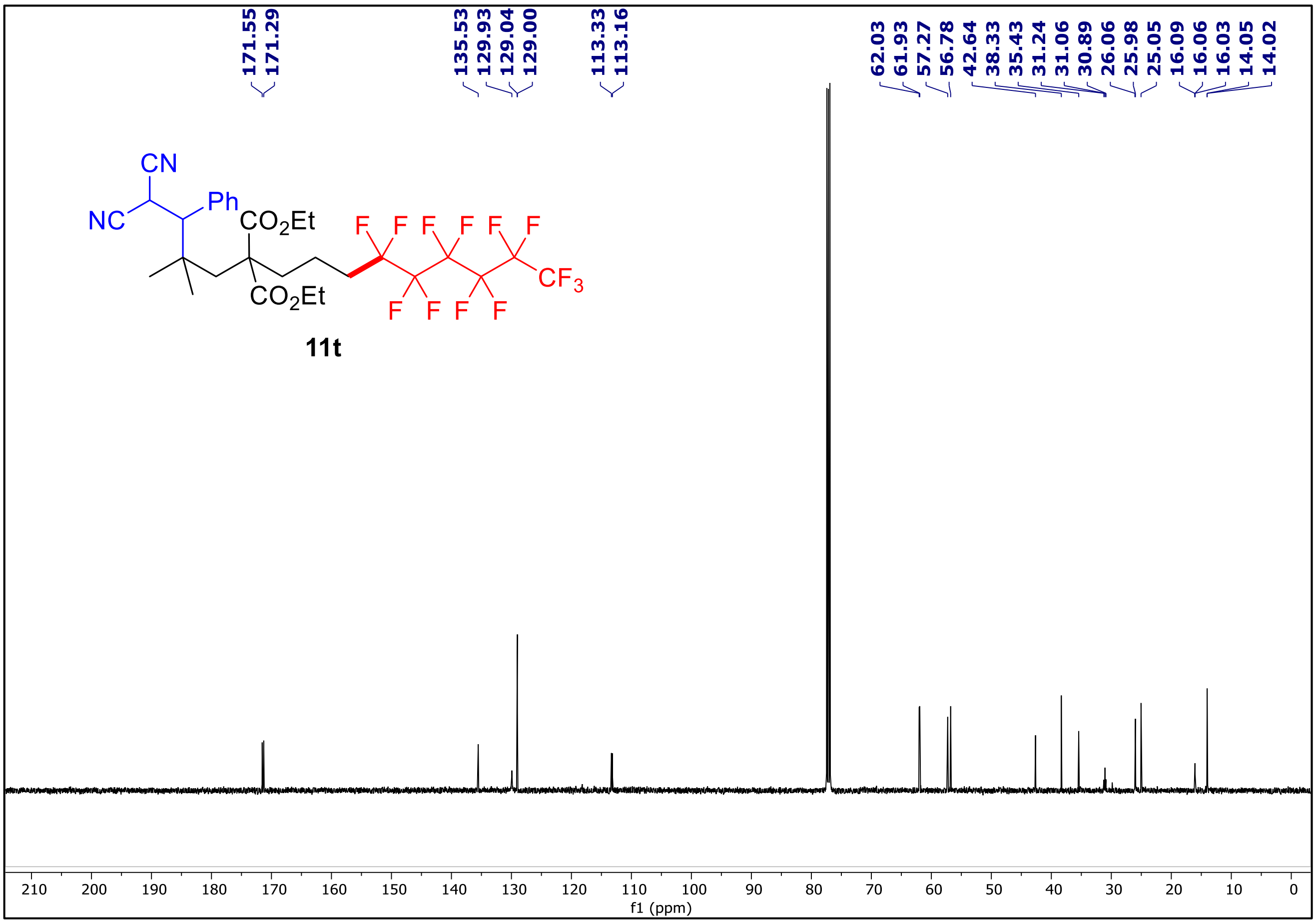

^{13}C{^{1}H} NMR of compound **11t** (126 MHz, $CDCl_3$)

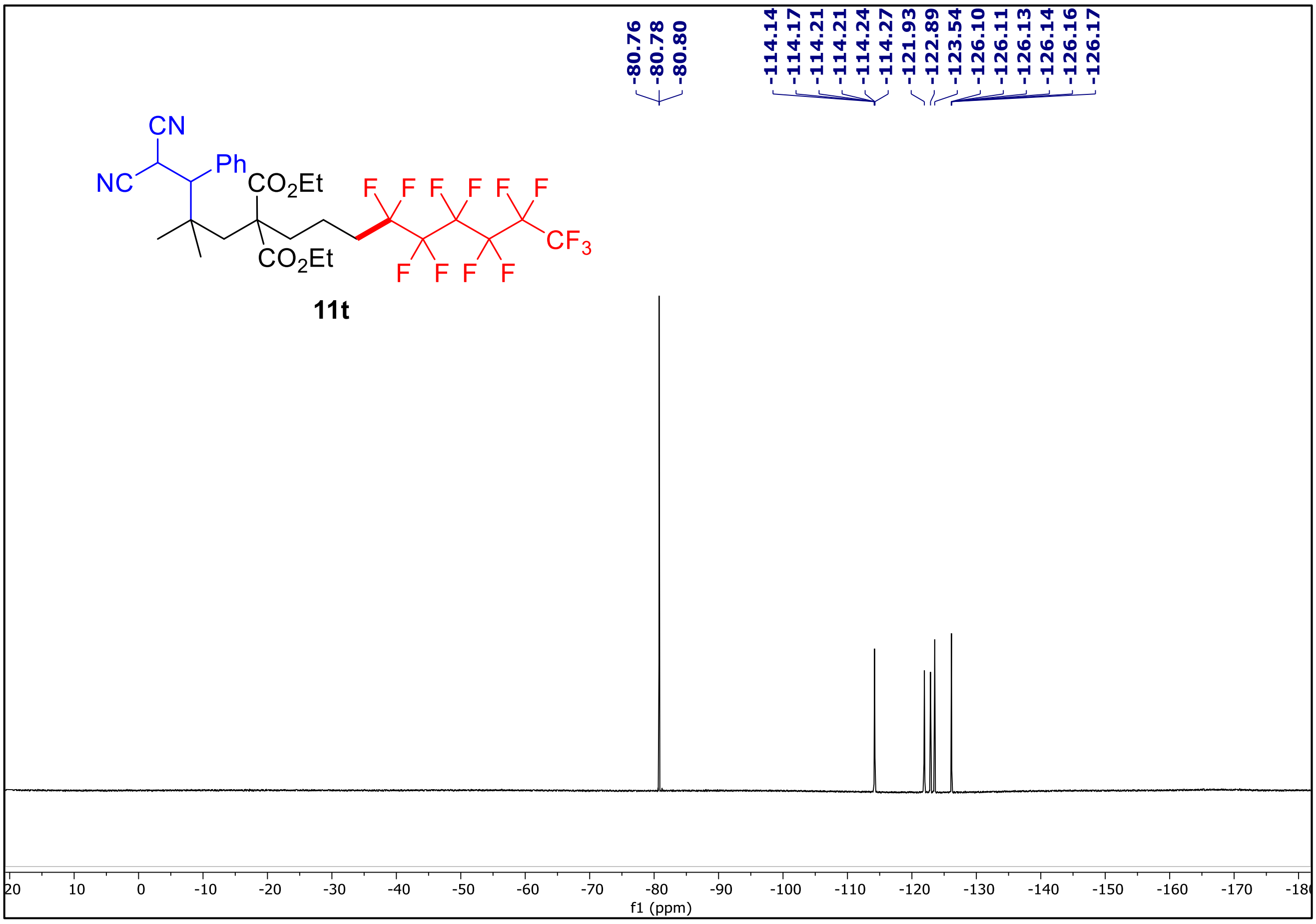

^{19}F NMR of compound **11t** (471 MHz, $CDCl_3$)

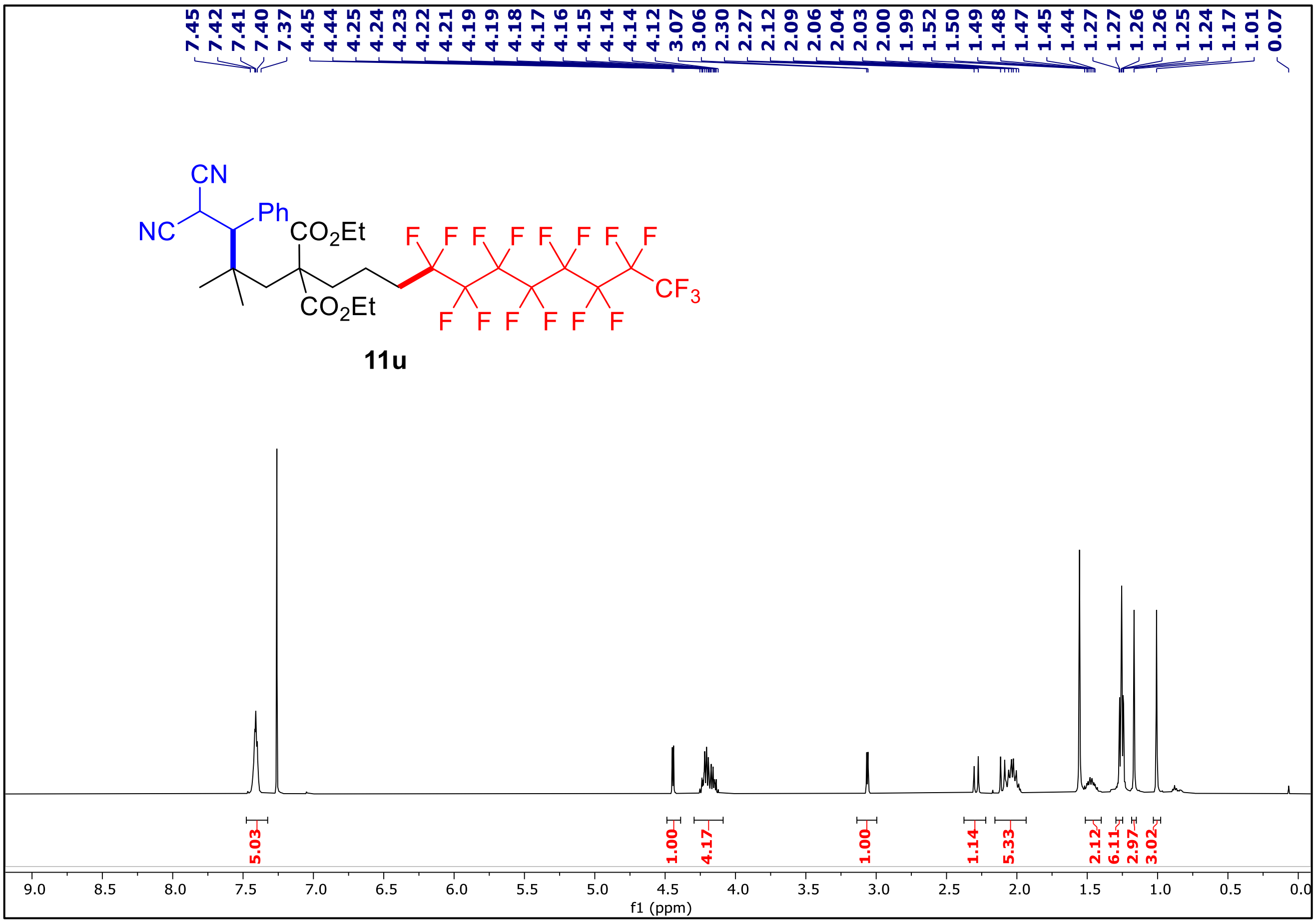

^{1}H NMR of compound **11u** (500 MHz, $CDCl_3$)

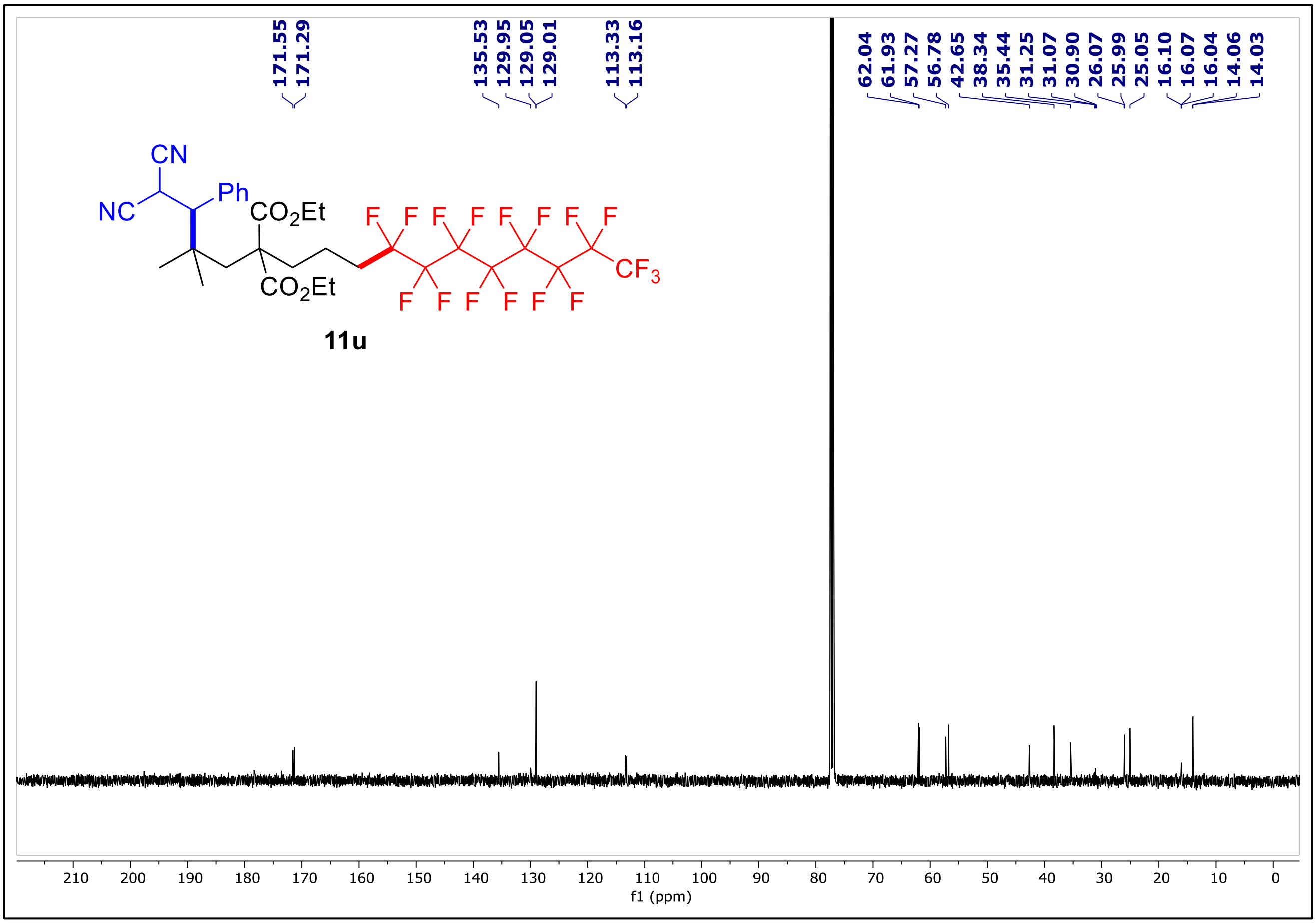

$^{13}C\{^{1}H\}$ NMR of compound **11u** (126 MHz, $CDCl_3$)

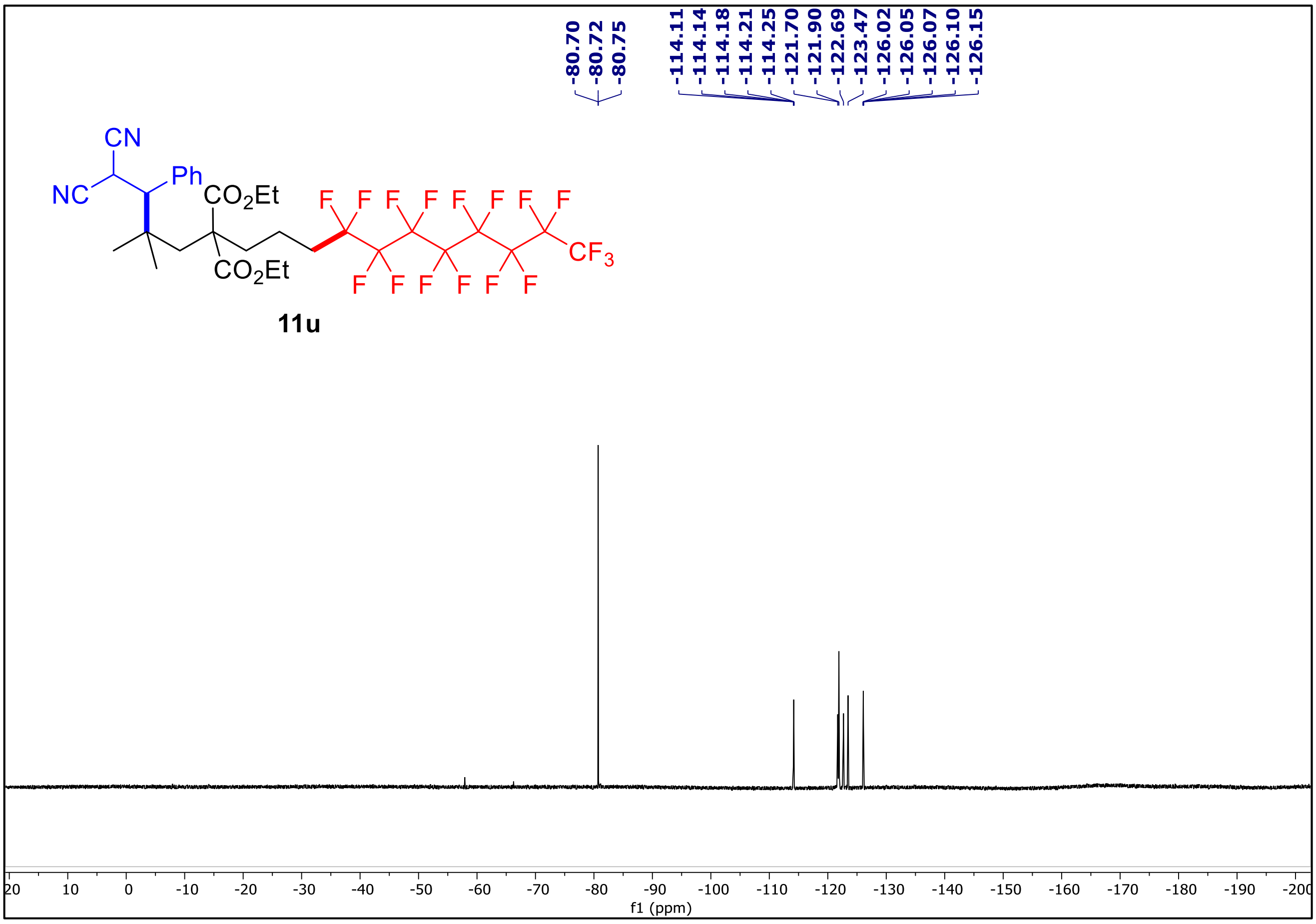

^{19}F NMR of compound **11u** (471 MHz, $CDCl_3$)

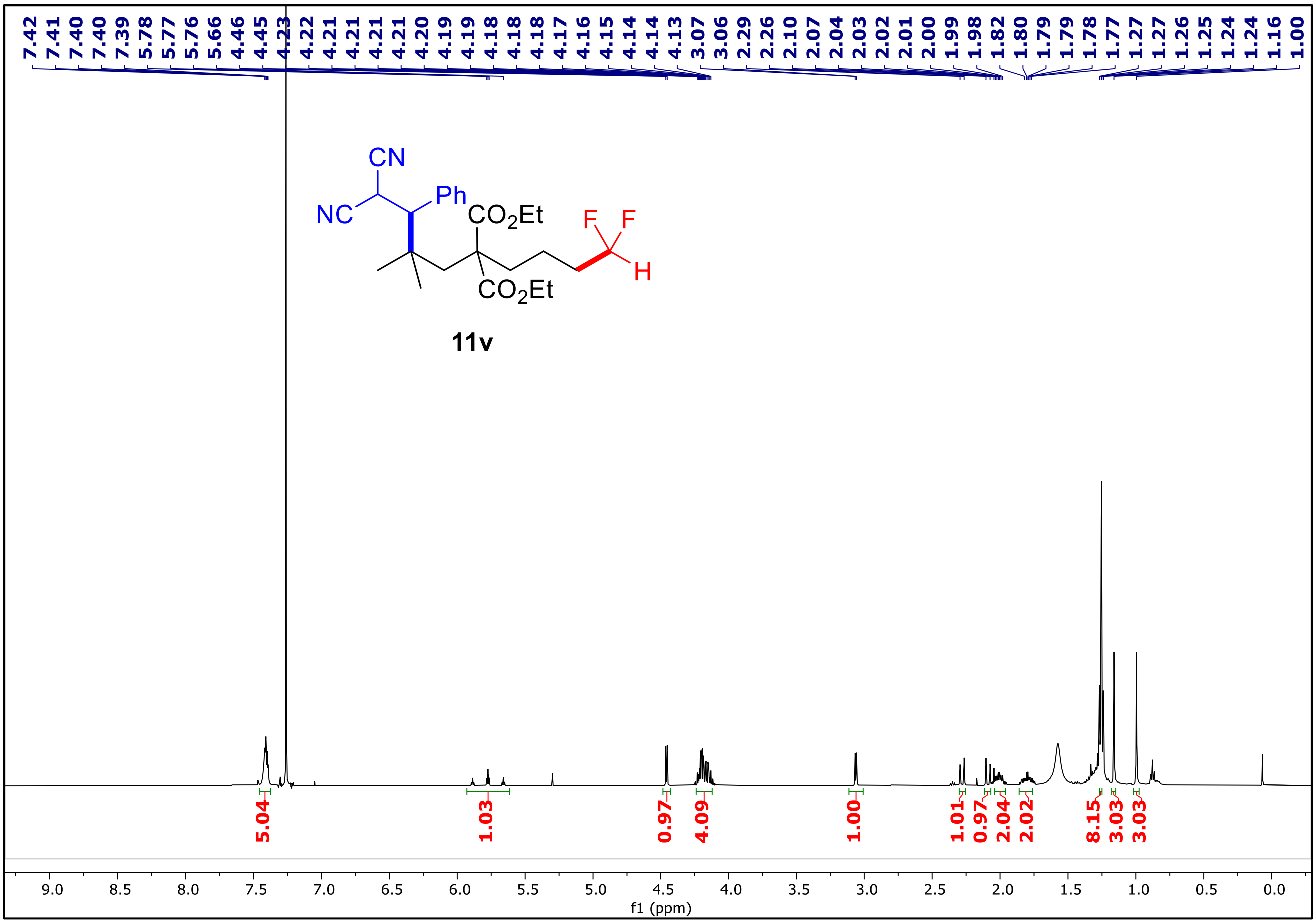

^{1}H NMR of compound **11v** (500 MHz, $CDCl_3$)

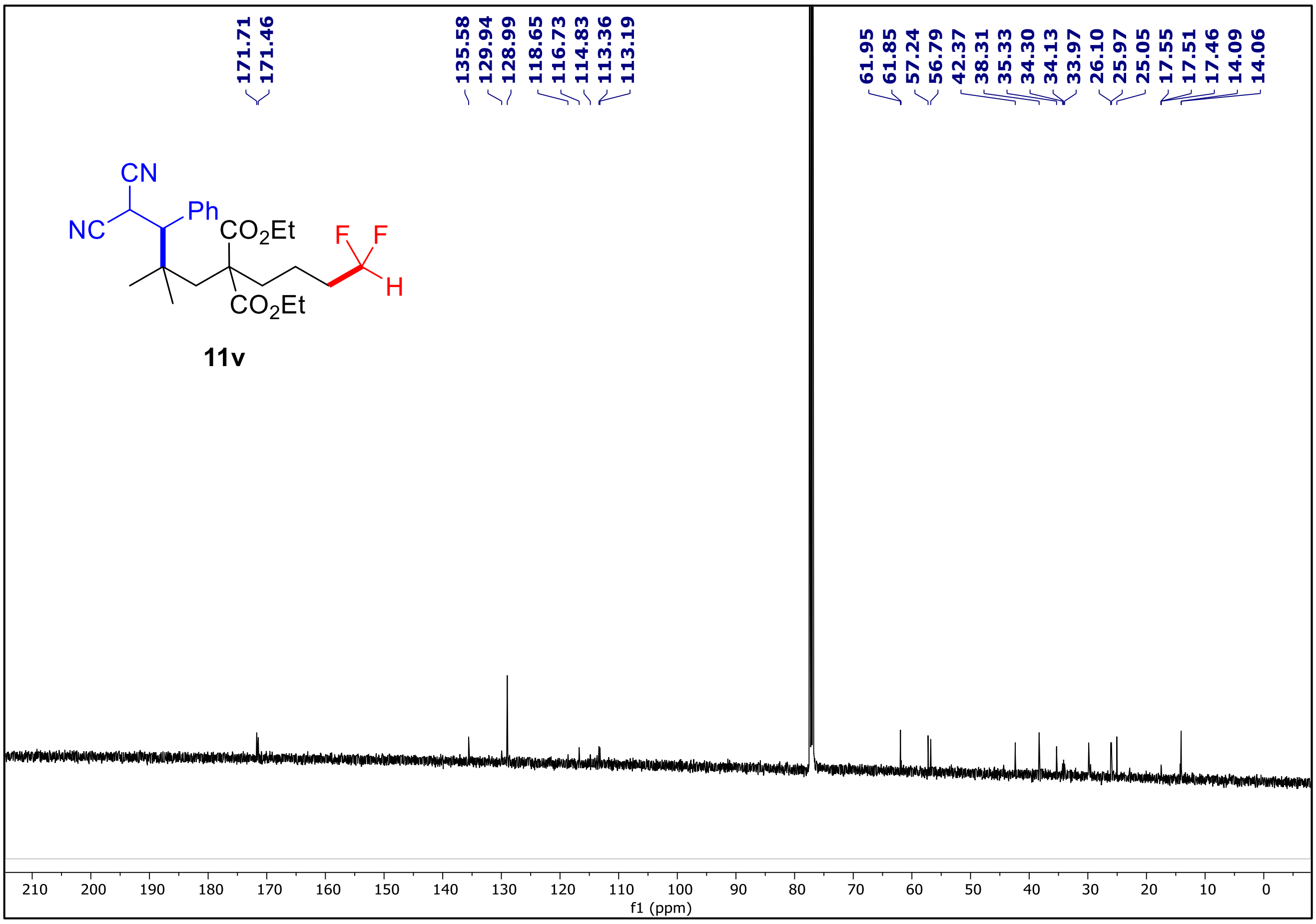

$^{13}C\{^{1}H\}$ NMR of compound **11v** (126 MHz, $CDCl_3$)

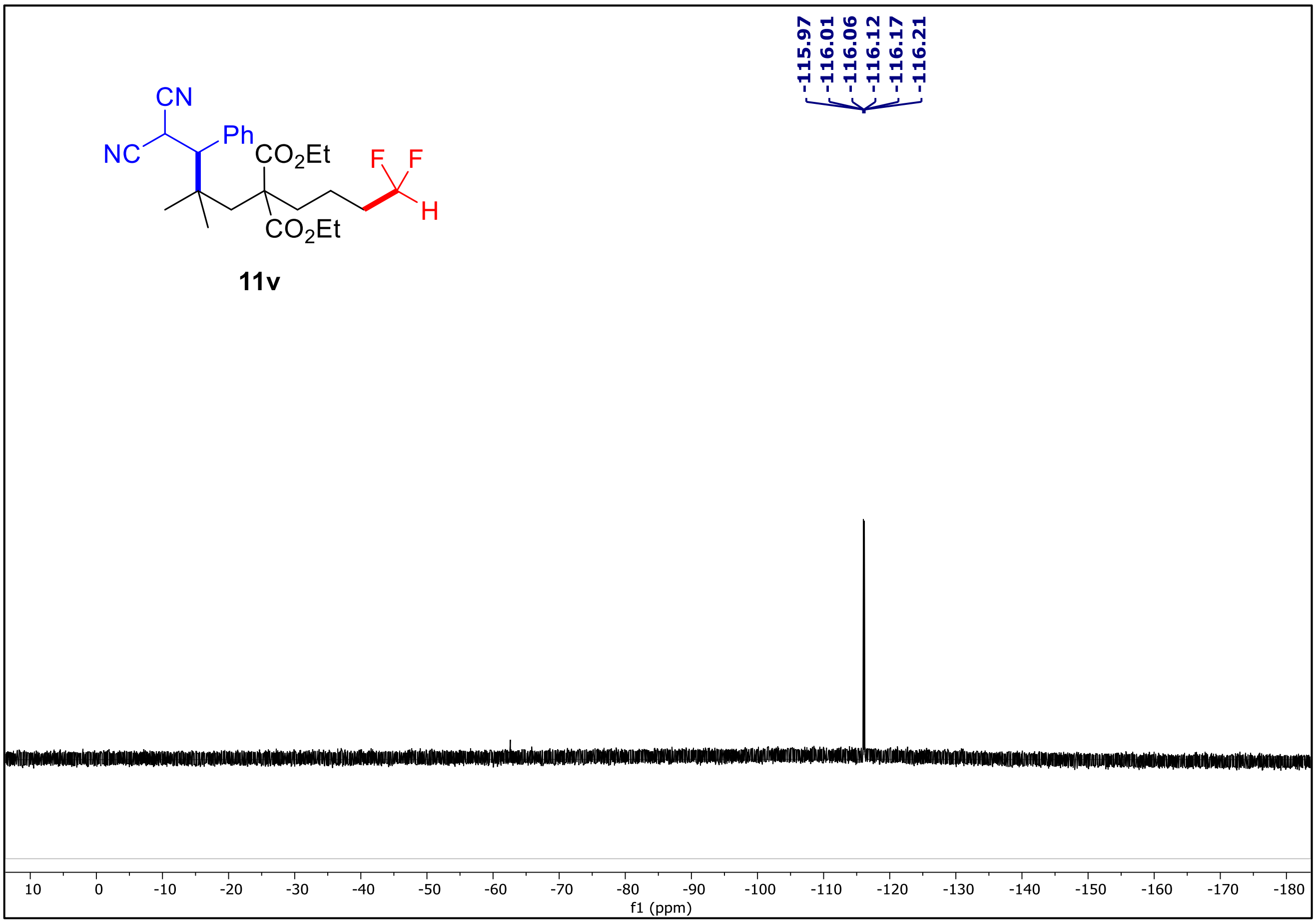

^{19}F NMR of compound **11v** (376 MHz, $CDCl_3$)

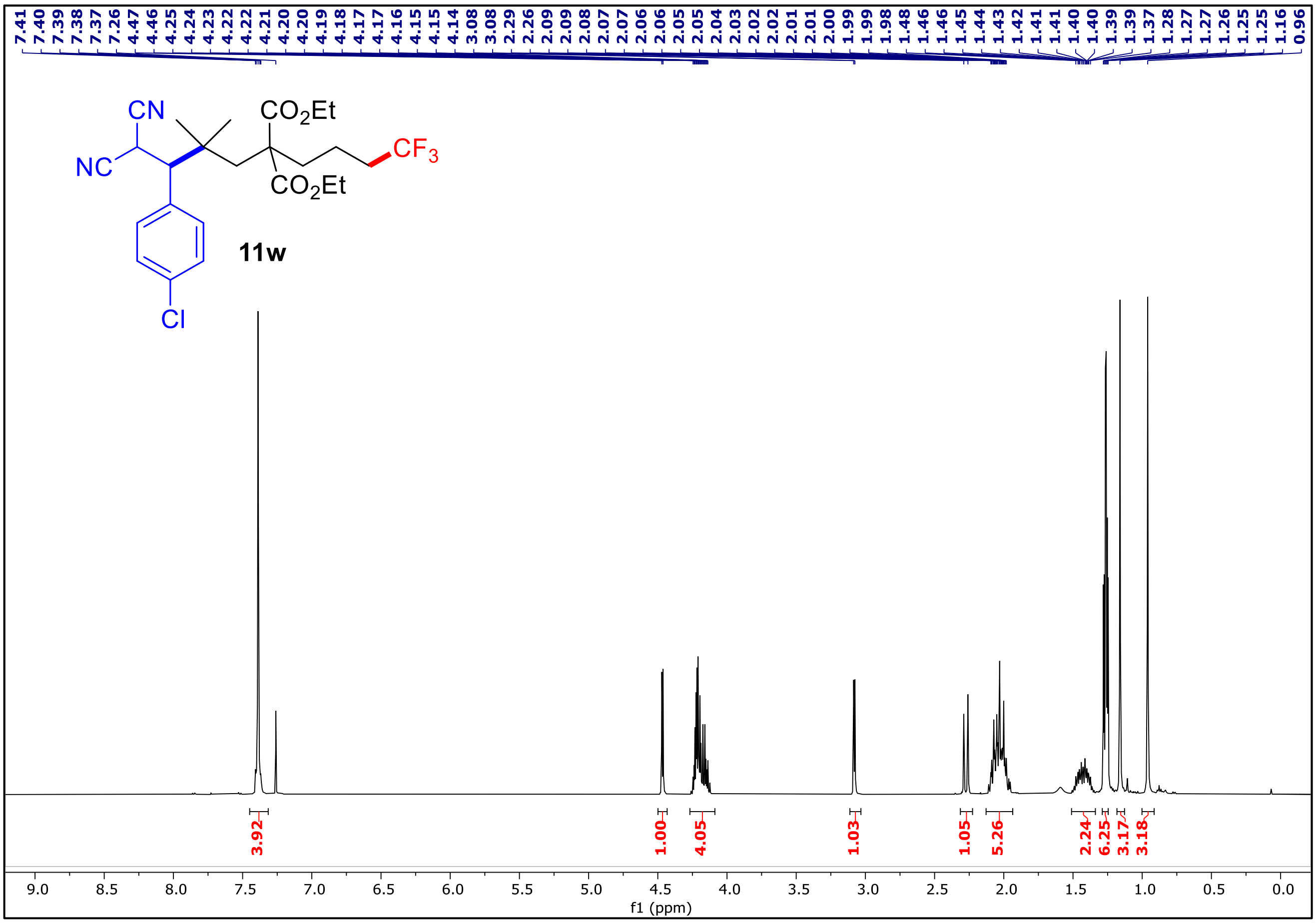

^{1}H NMR of compound **11w** (500 MHz, $CDCl_3$)

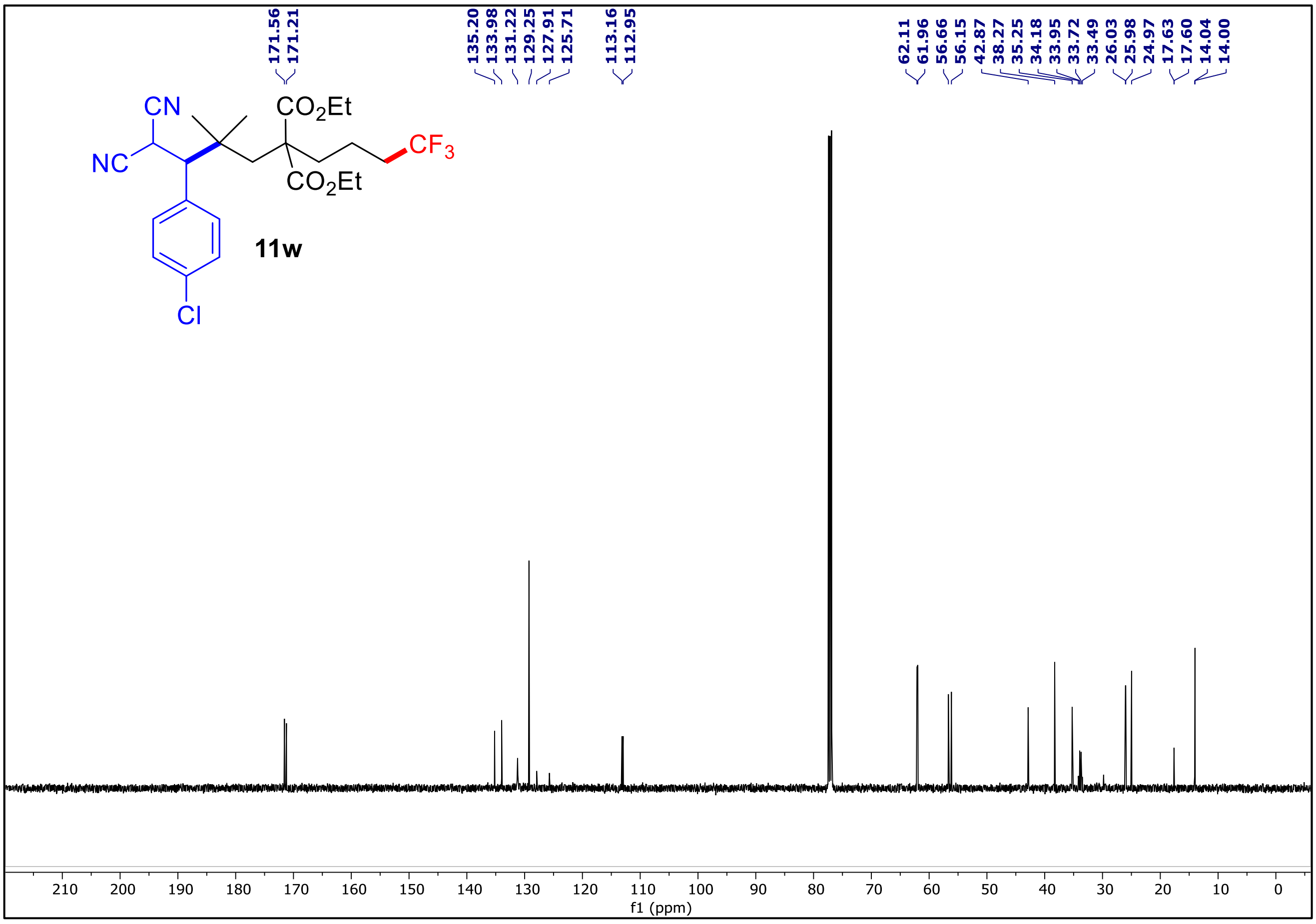

$^{13}C\{^{1}H\}$ NMR of compound **11w** (126 MHz, $CDCl_3$)

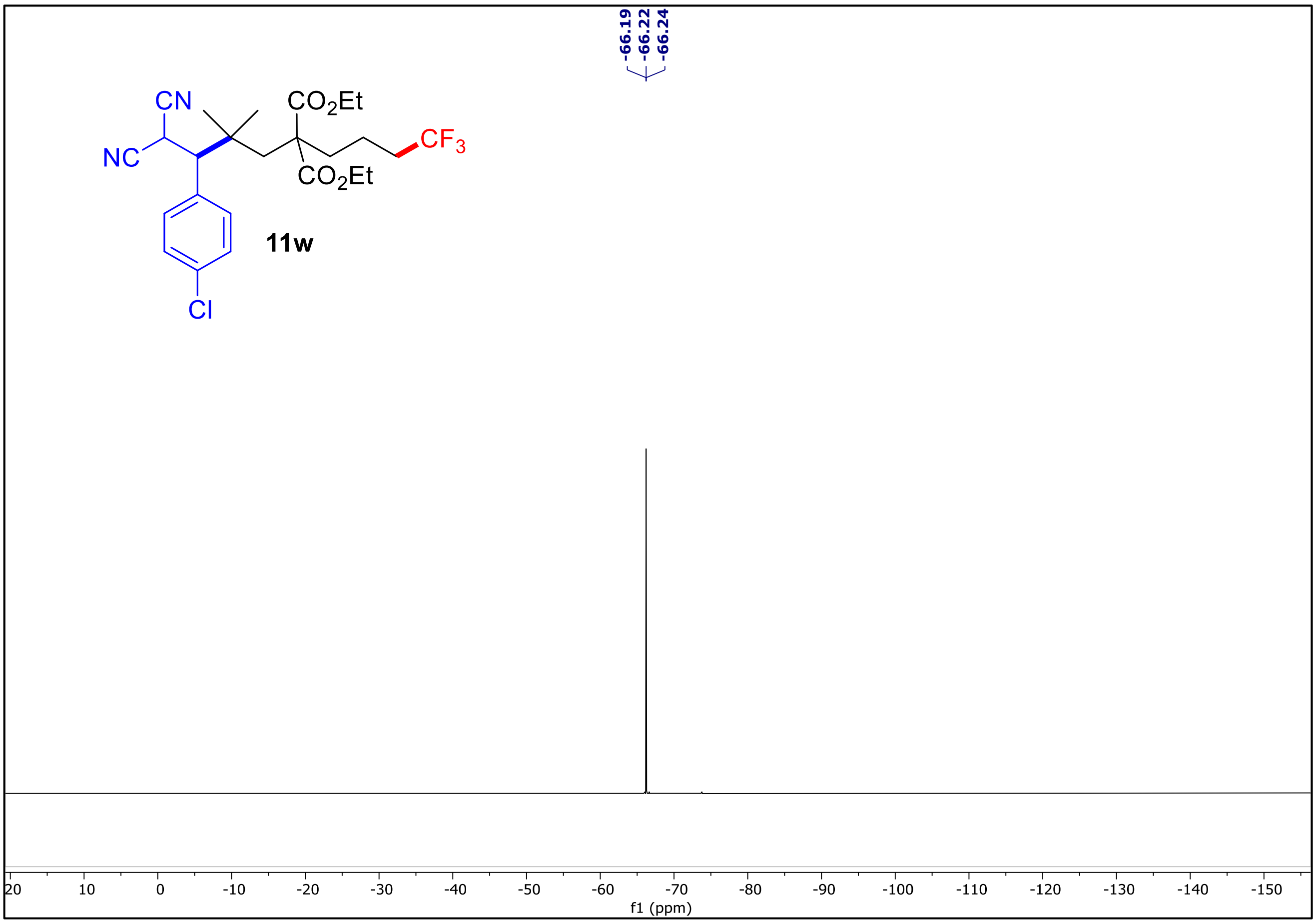

^{19}F NMR of compound **11w** (471 MHz, $CDCl_3$)

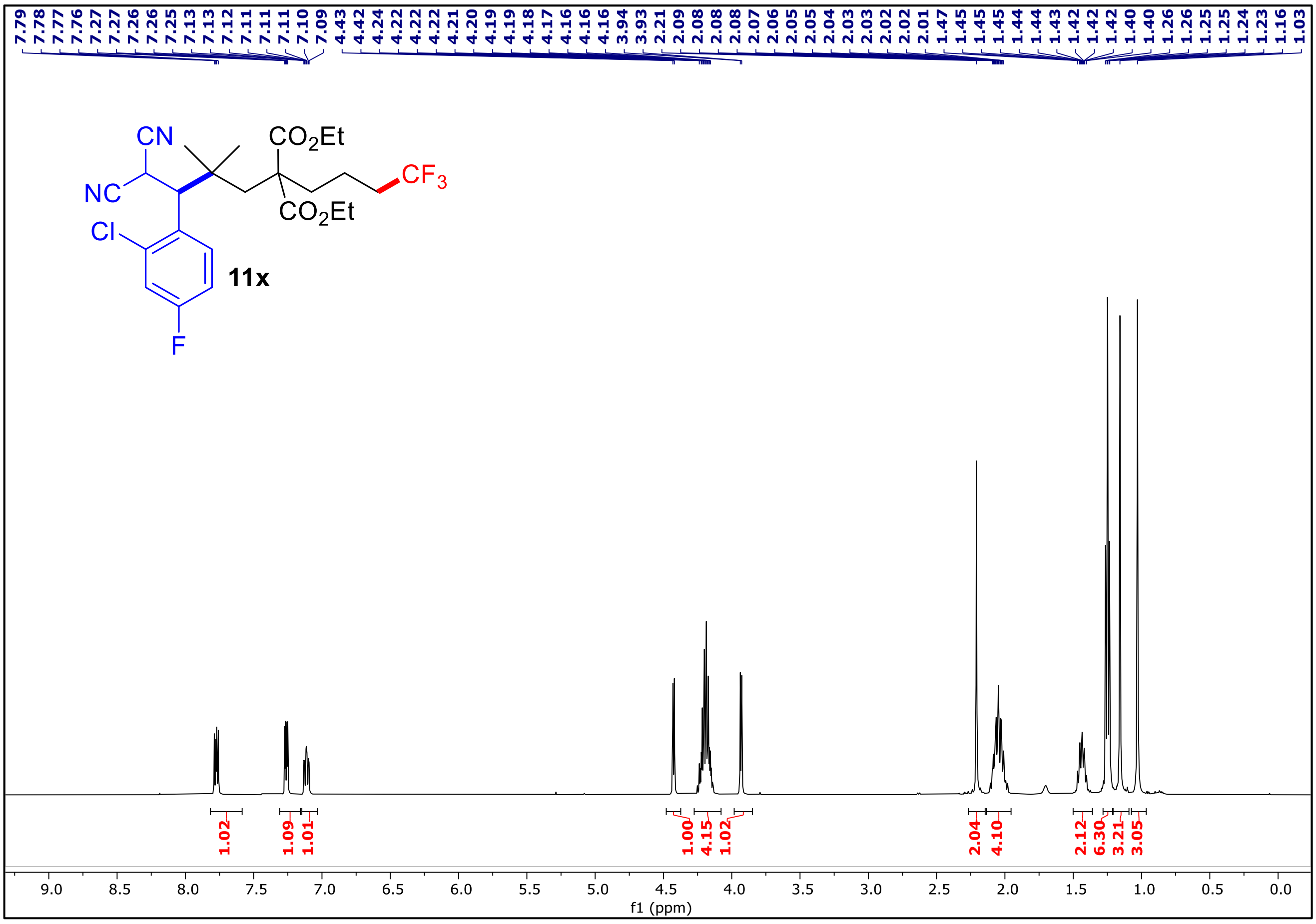

^{1}H NMR of compound **11x** (500 MHz, $CDCl_3$)

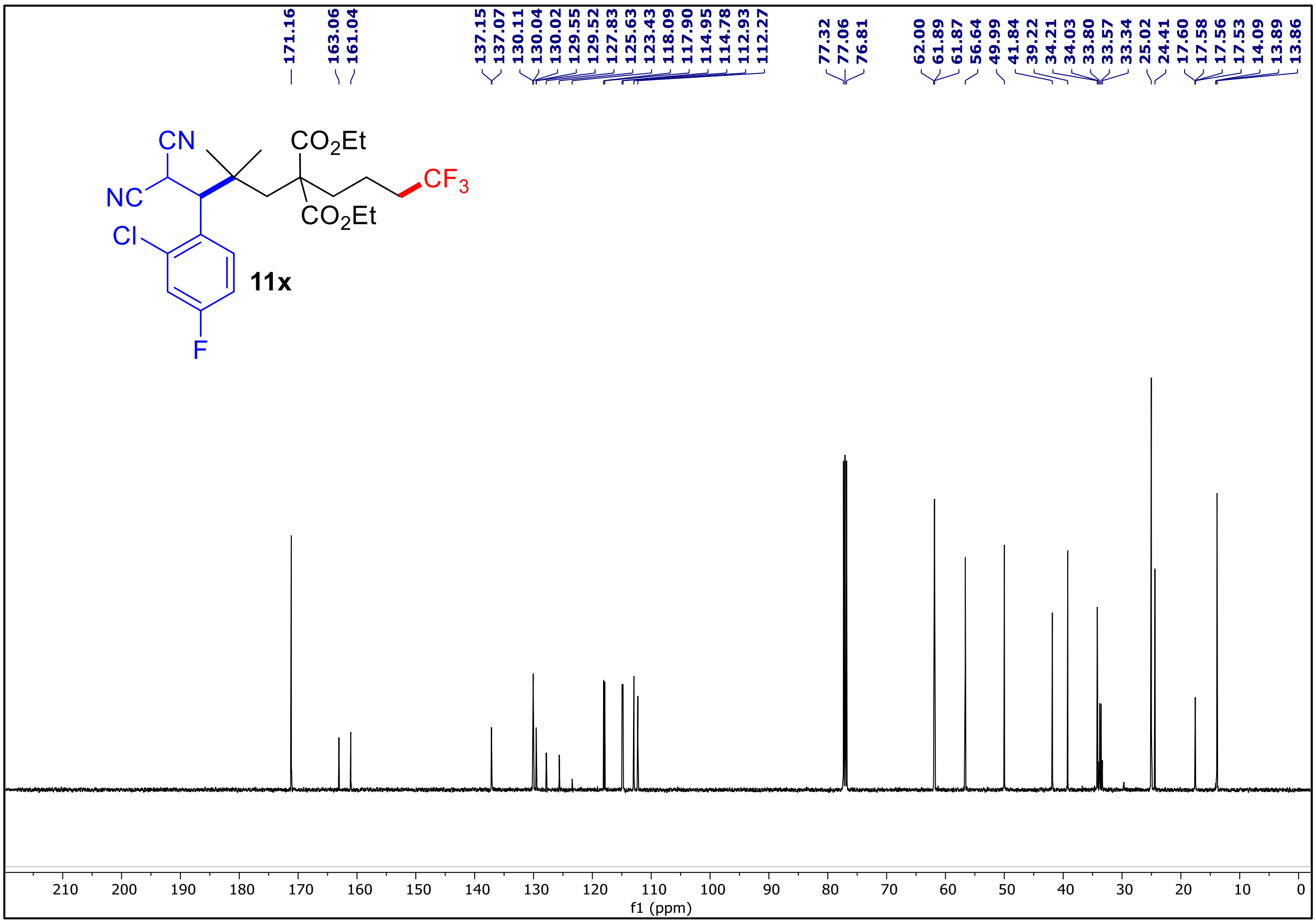

$^{13}C\{^1H\}$ NMR of compound **11x** (126 MHz, $CDCl_3$)

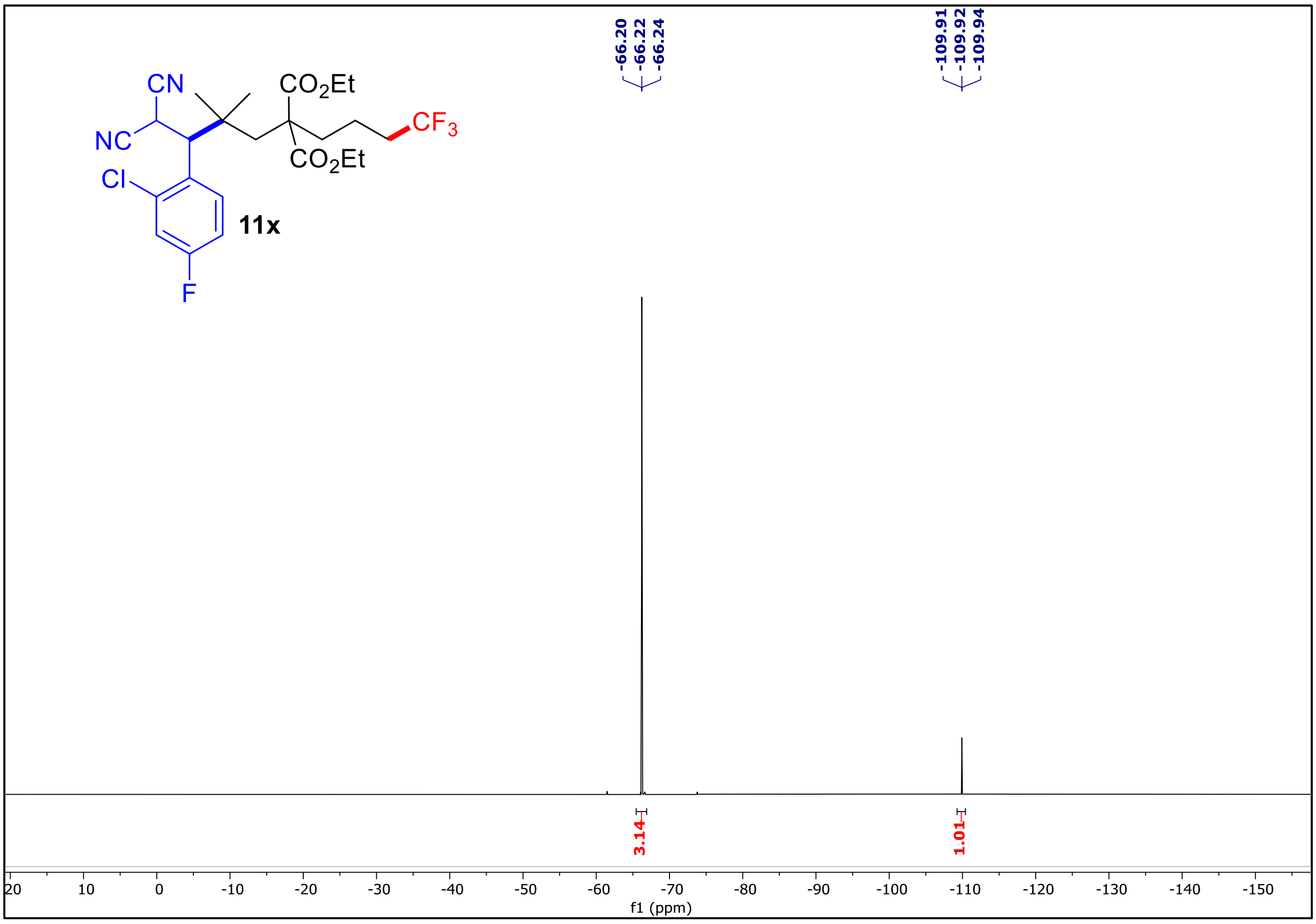

¹⁹F NMR of compound **11x** (471 MHz, $CDCl_3$)

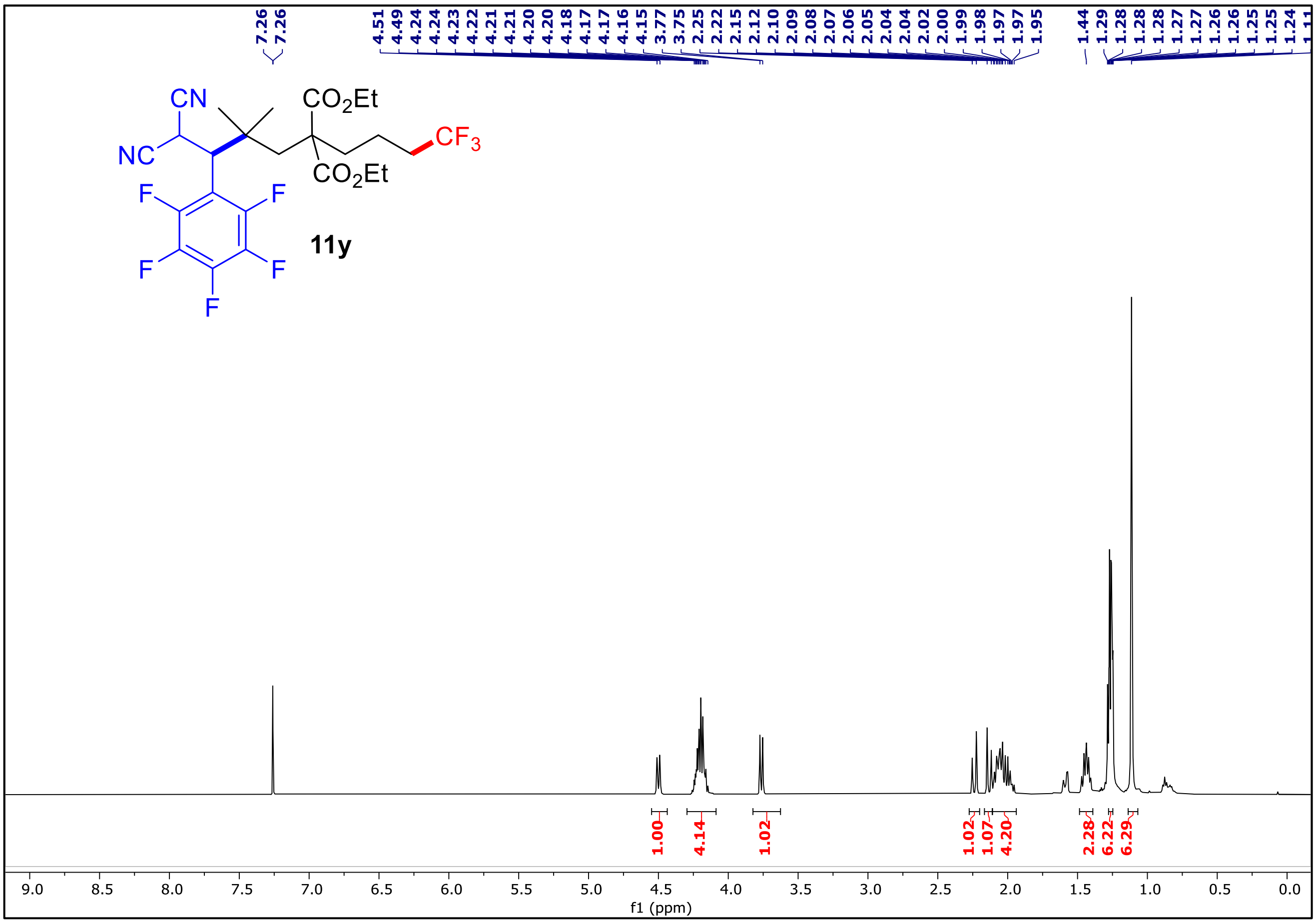

¹H NMR of compound **11y** (500 MHz, $CDCl_3$)

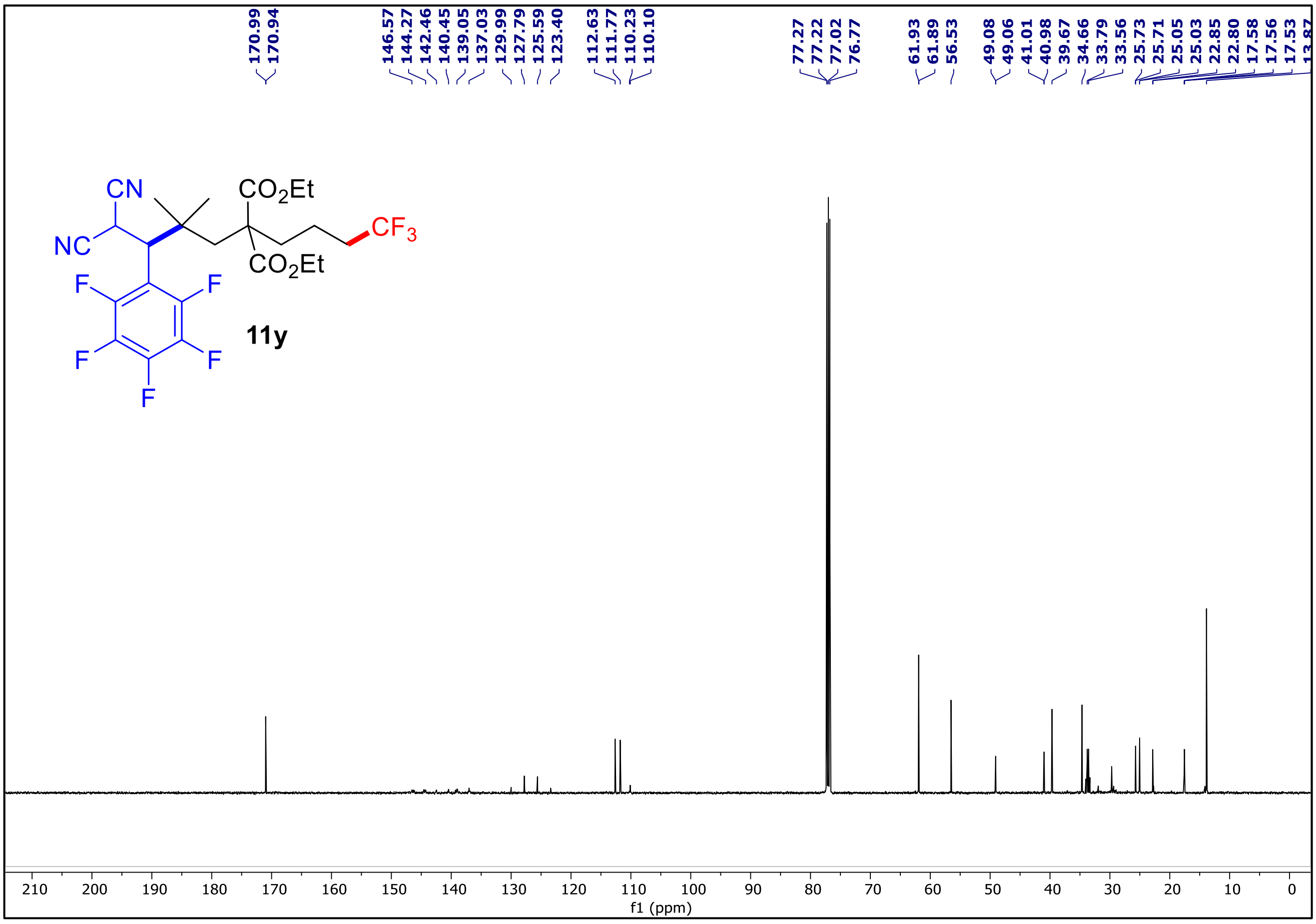

$^{13}C\{^{1}H\}$ NMR of compound **11y** (126 MHz, $CDCl_3$)

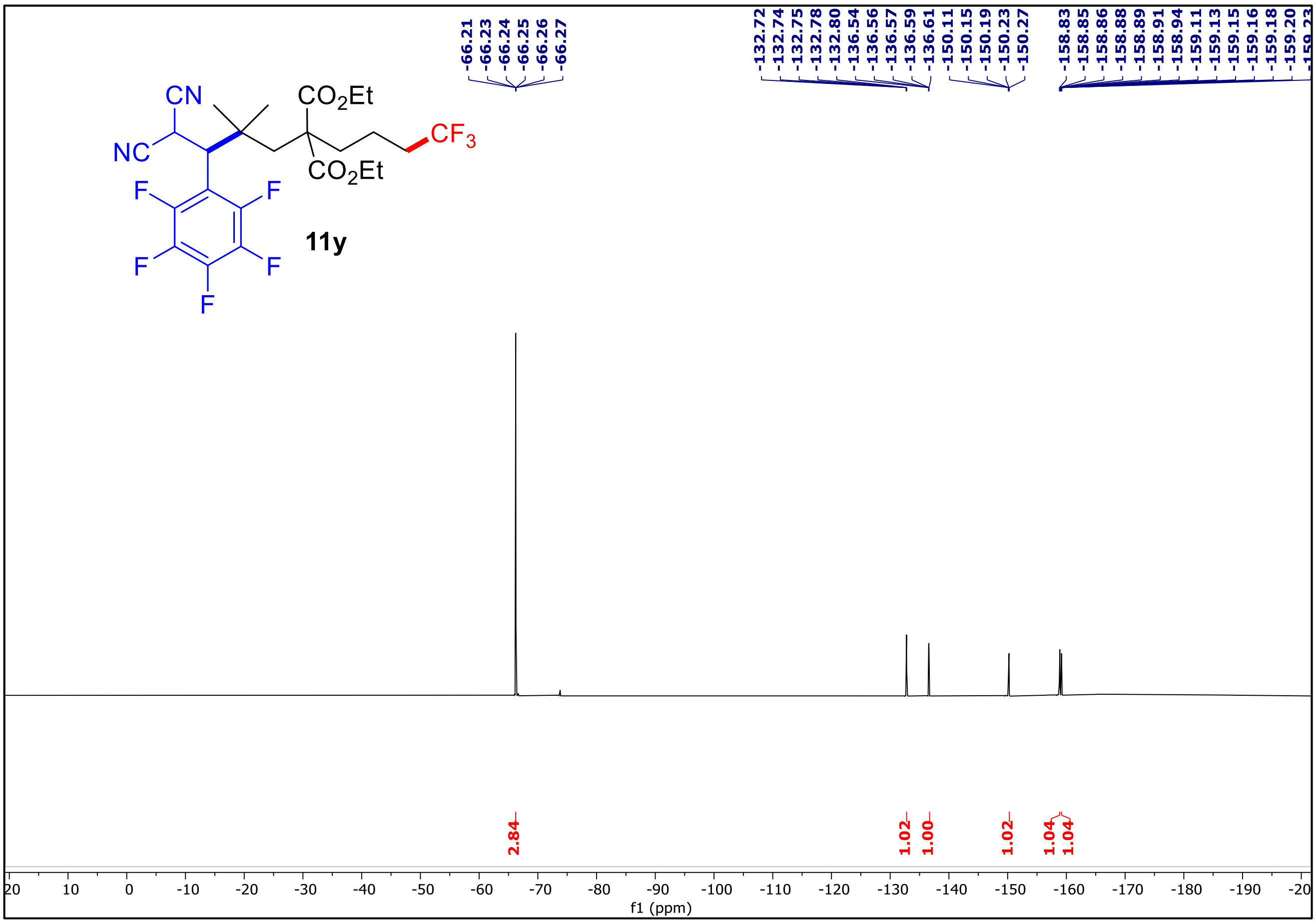

^{19}F NMR of compound **11y** (471 MHz, $CDCl_3$)

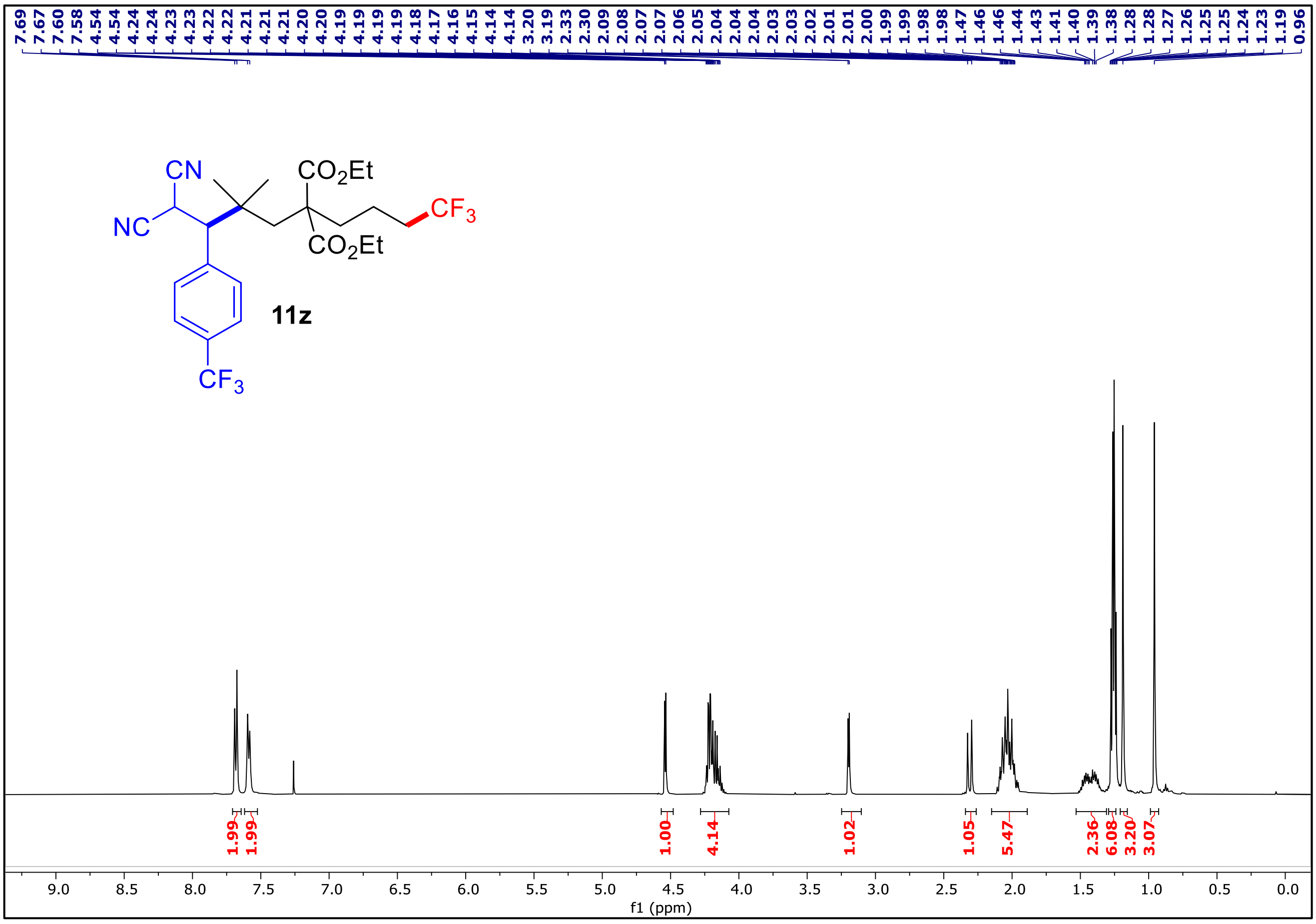

¹H NMR of compound **11z** (500 MHz, CDCl₃)

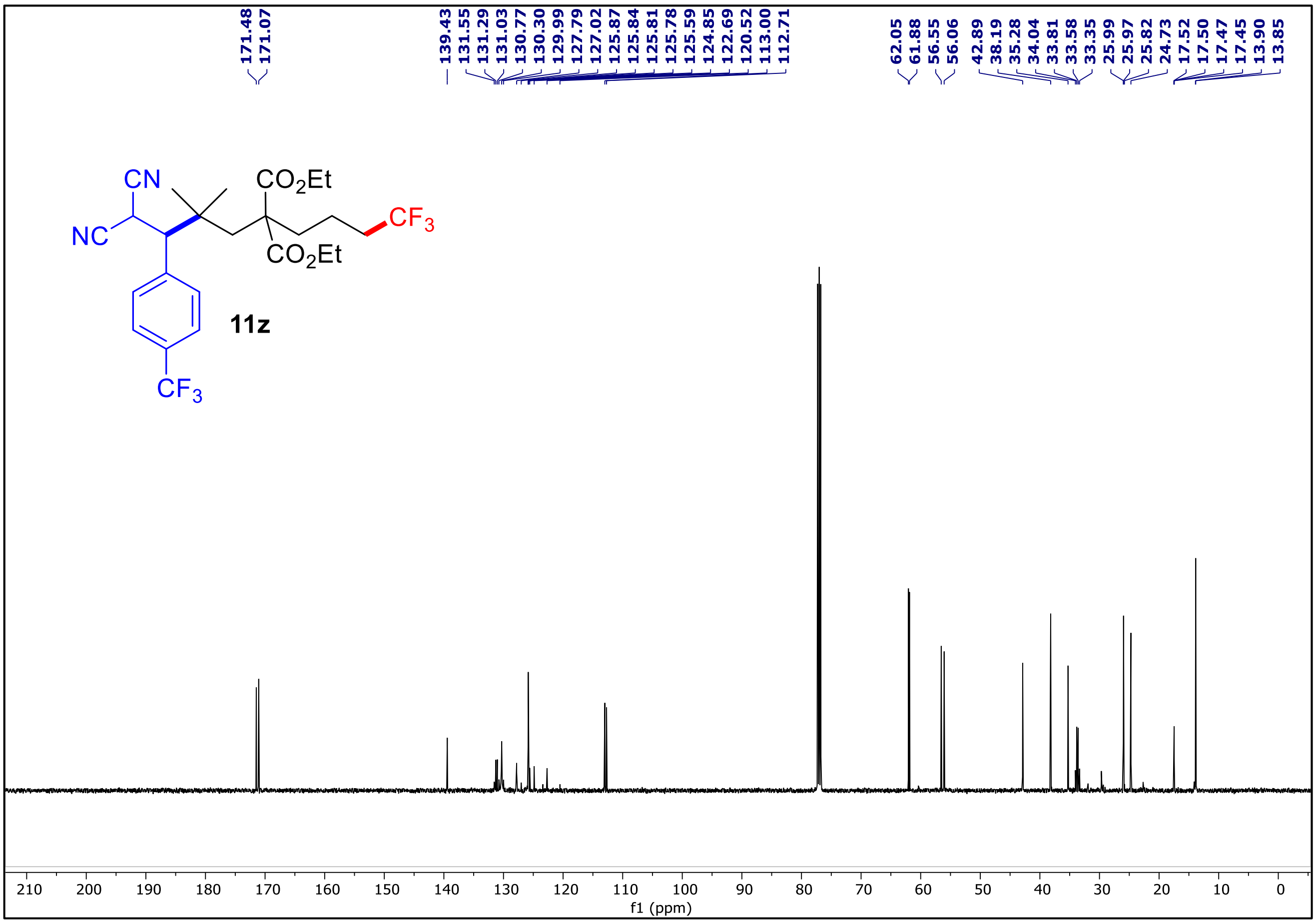

$^{13}C\{^1H\}$ NMR of compound **11z** (126 MHz, CDCl₃)

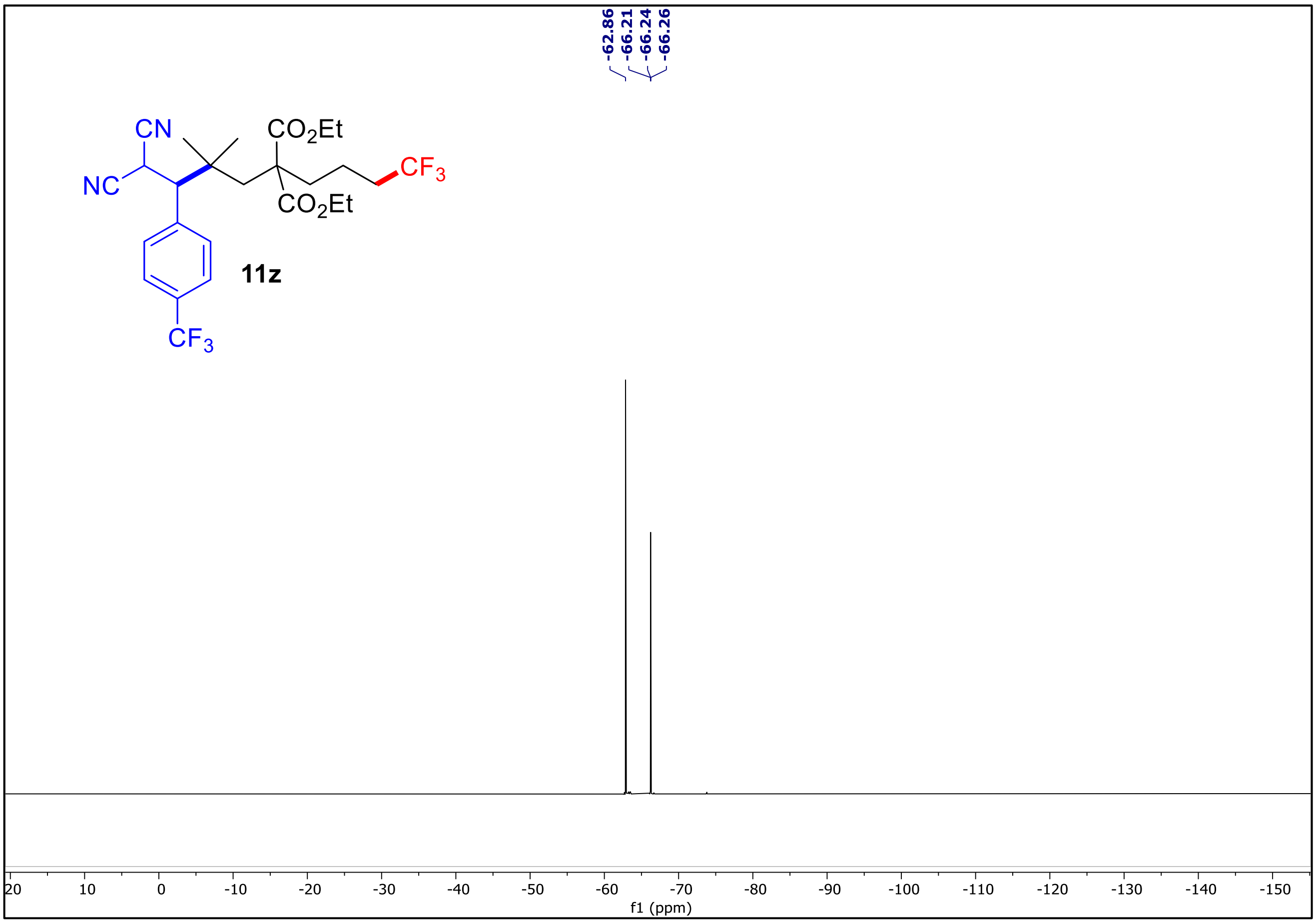

^{19}F NMR of compound **11z** (471 MHz, $CDCl_3$)

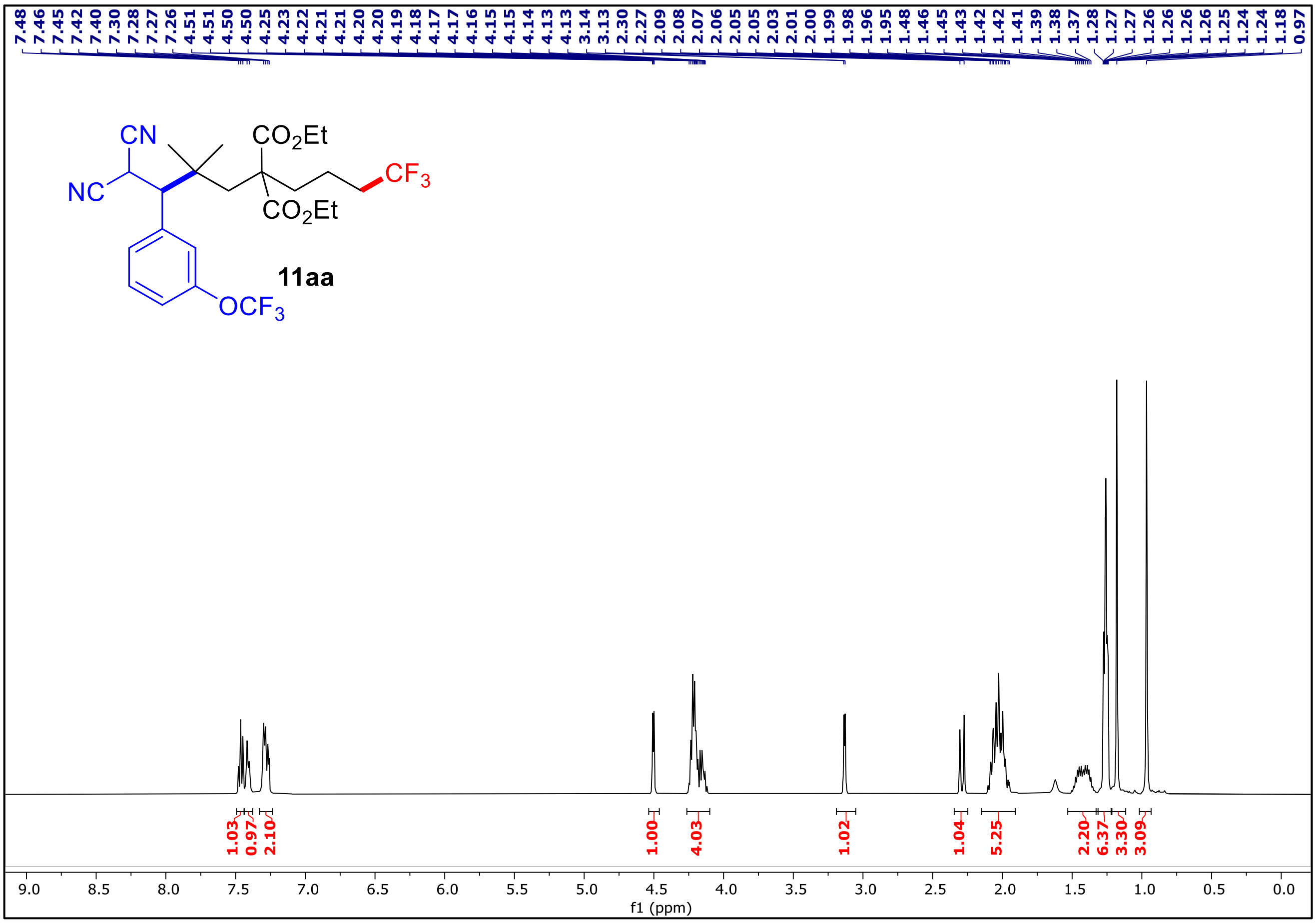

^{1}H NMR of compound **11aa** (500 MHz, $CDCl_3$)

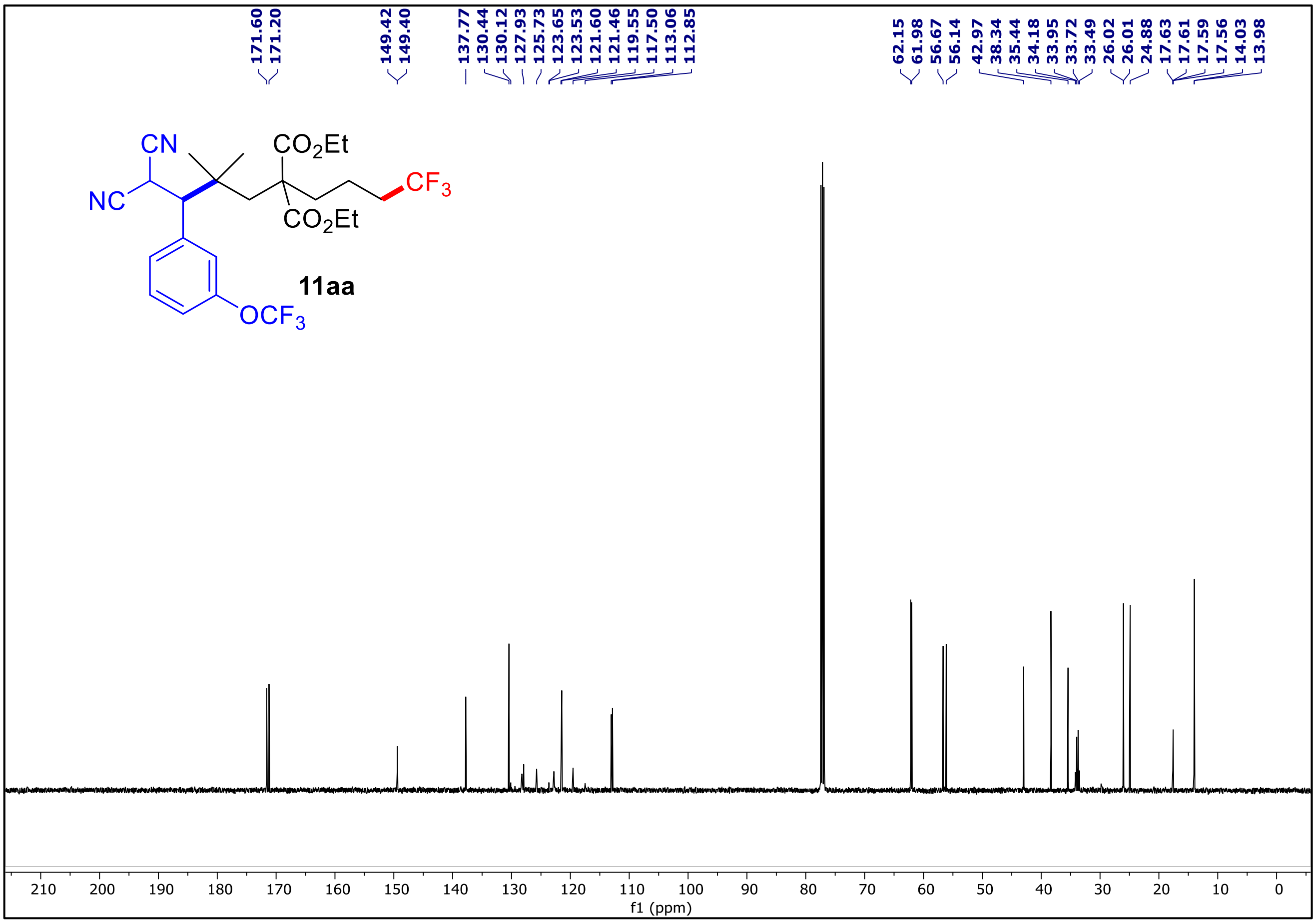

$^{13}C\{^1H\}$ NMR of compound **11aa** (126 MHz, $CDCl_3$)

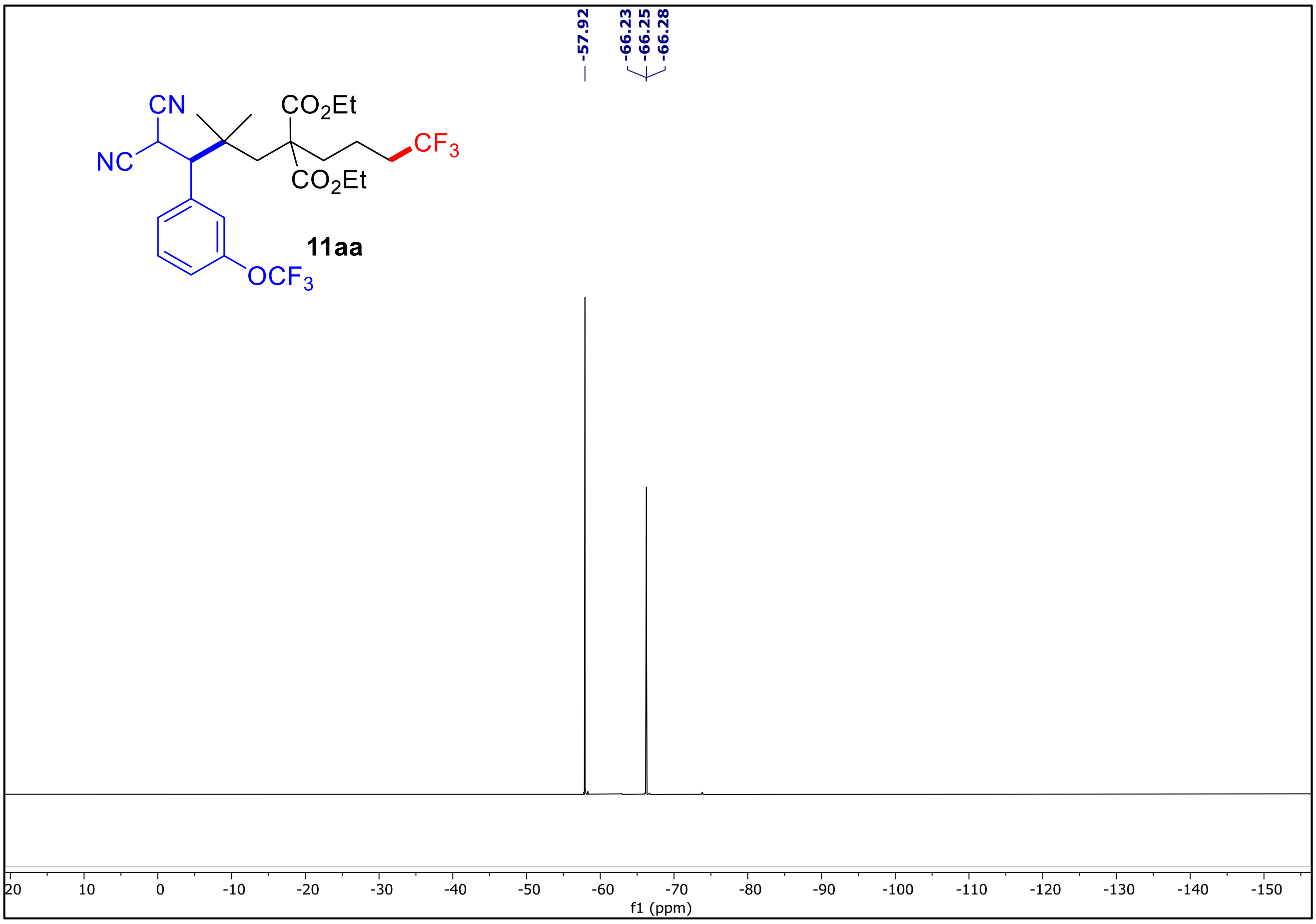

^{19}F NMR of compound **11aa** (471 MHz, $CDCl_3$)

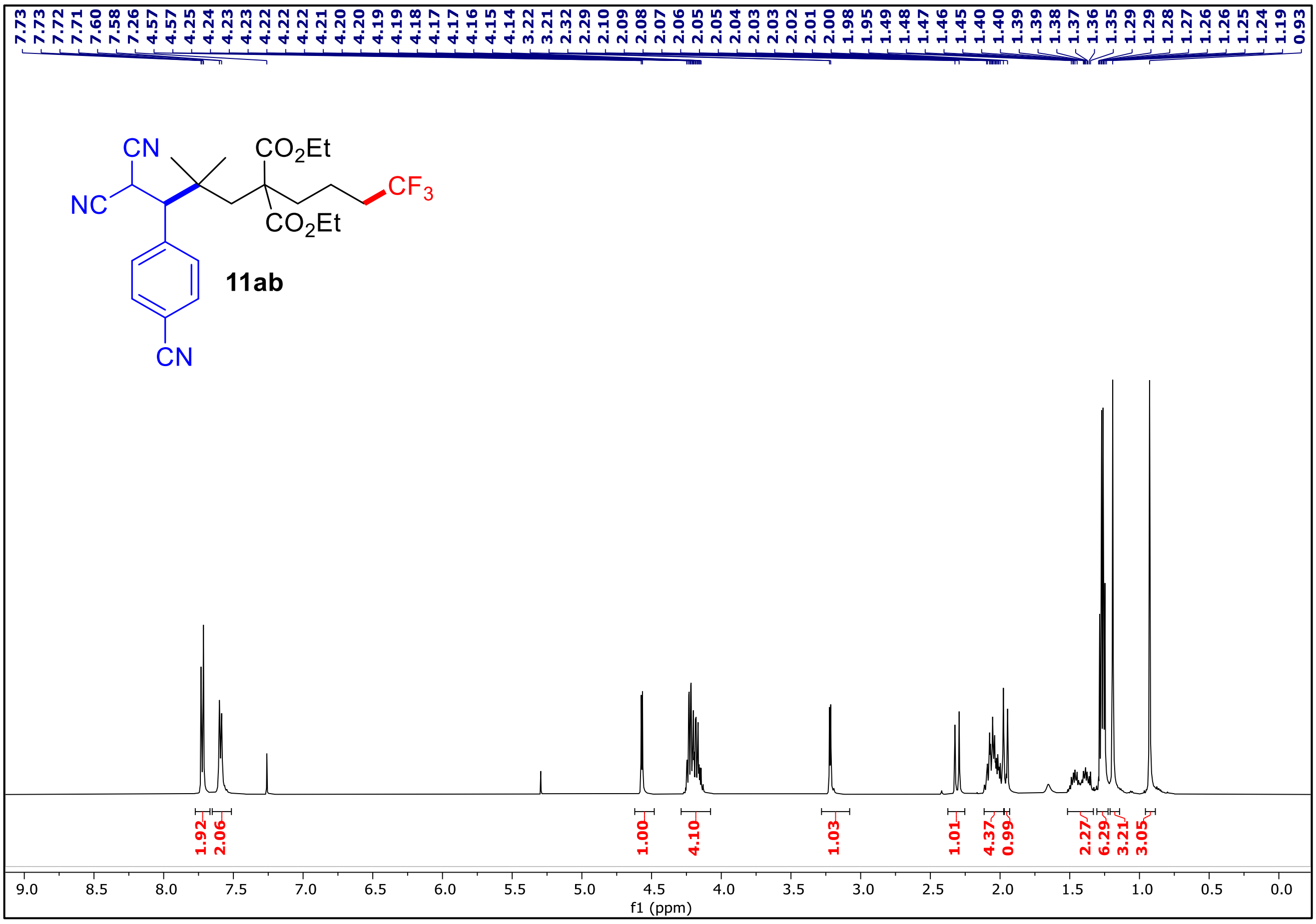

^{1}H NMR of compound **11ab** (500 MHz, $CDCl_3$)

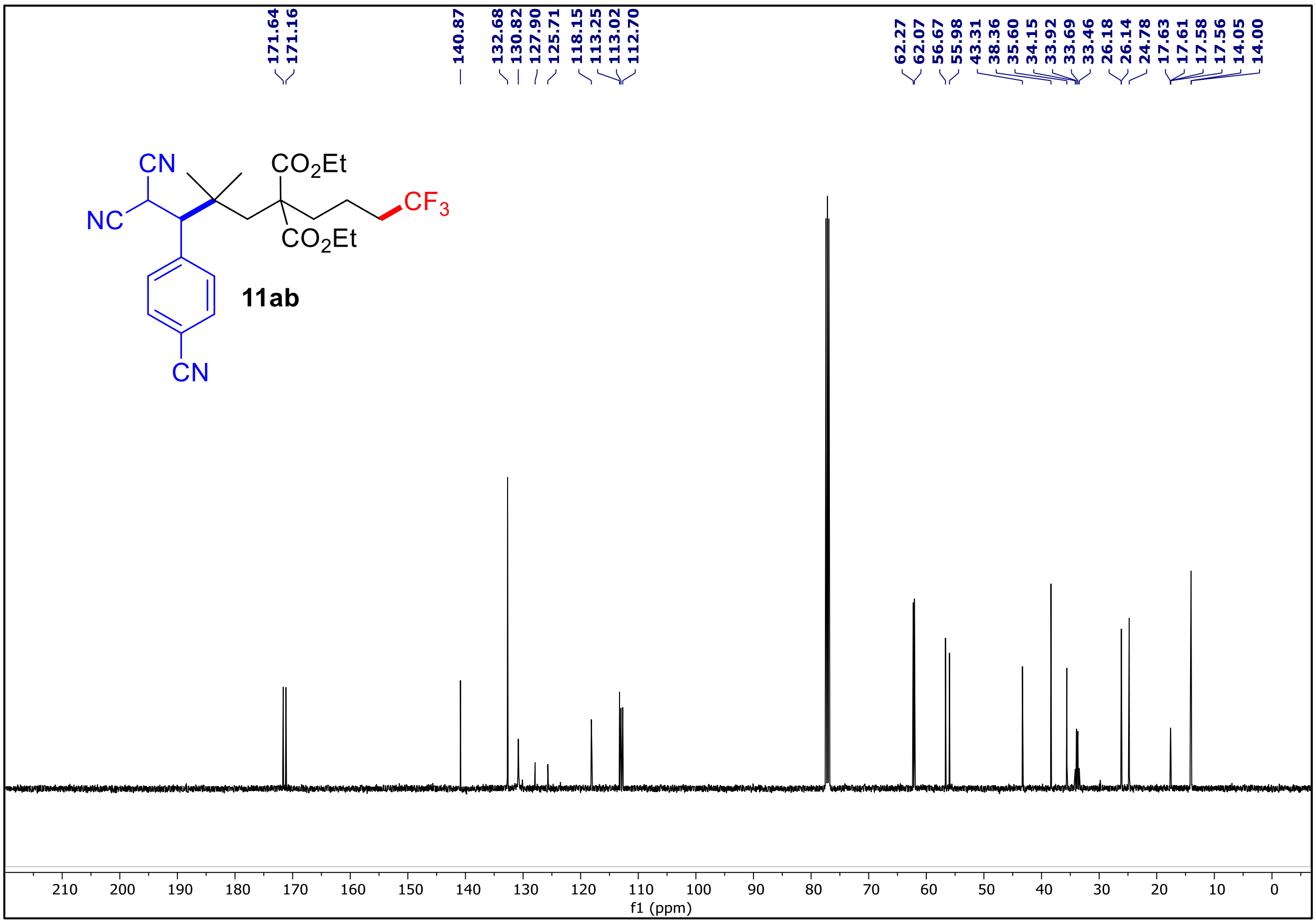

$^{13}C\{^1H\}$ NMR of compound **11ab** (126 MHz, $CDCl_3$)

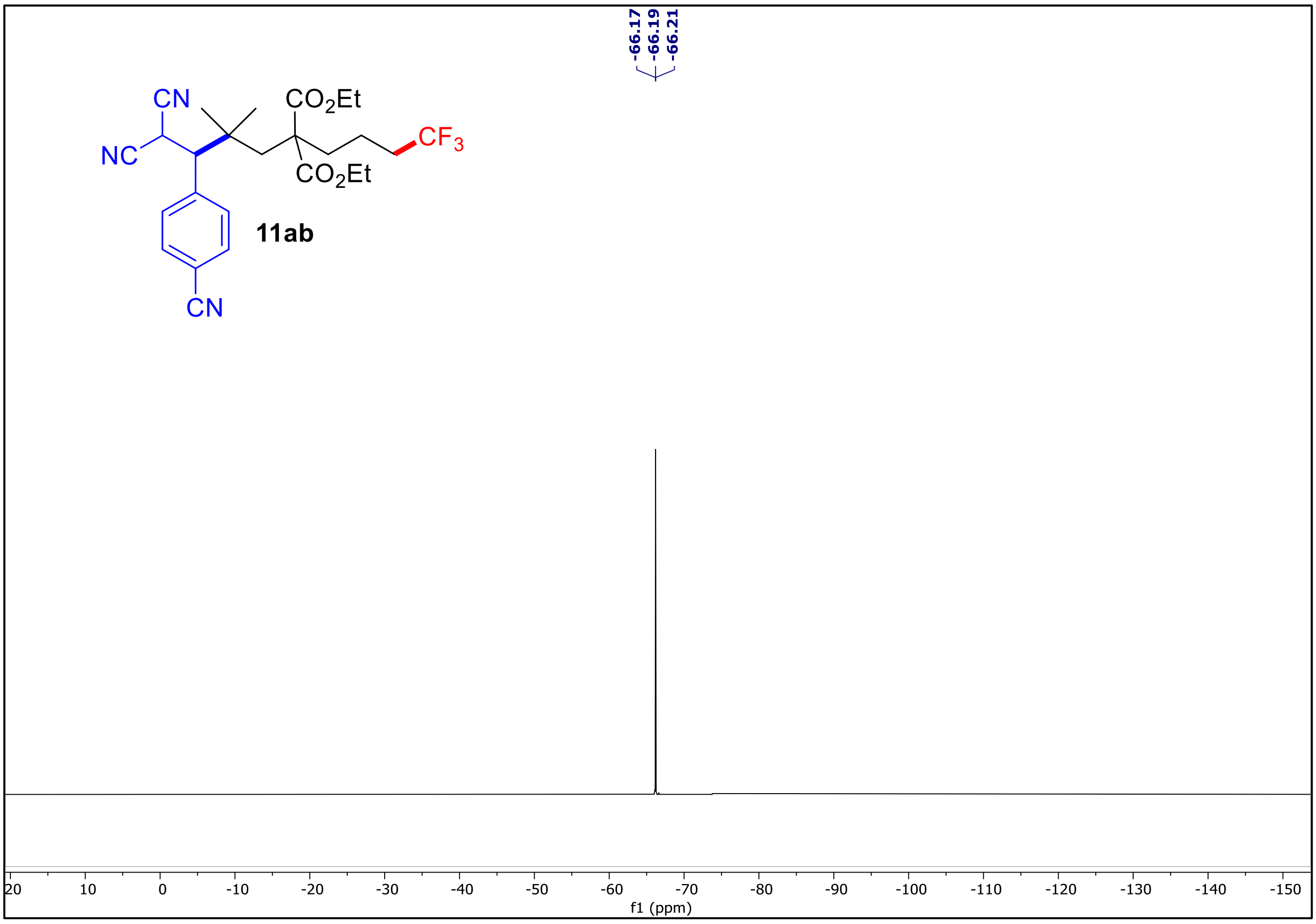

^{19}F NMR of compound **11ab** (471 MHz, $CDCl_3$)

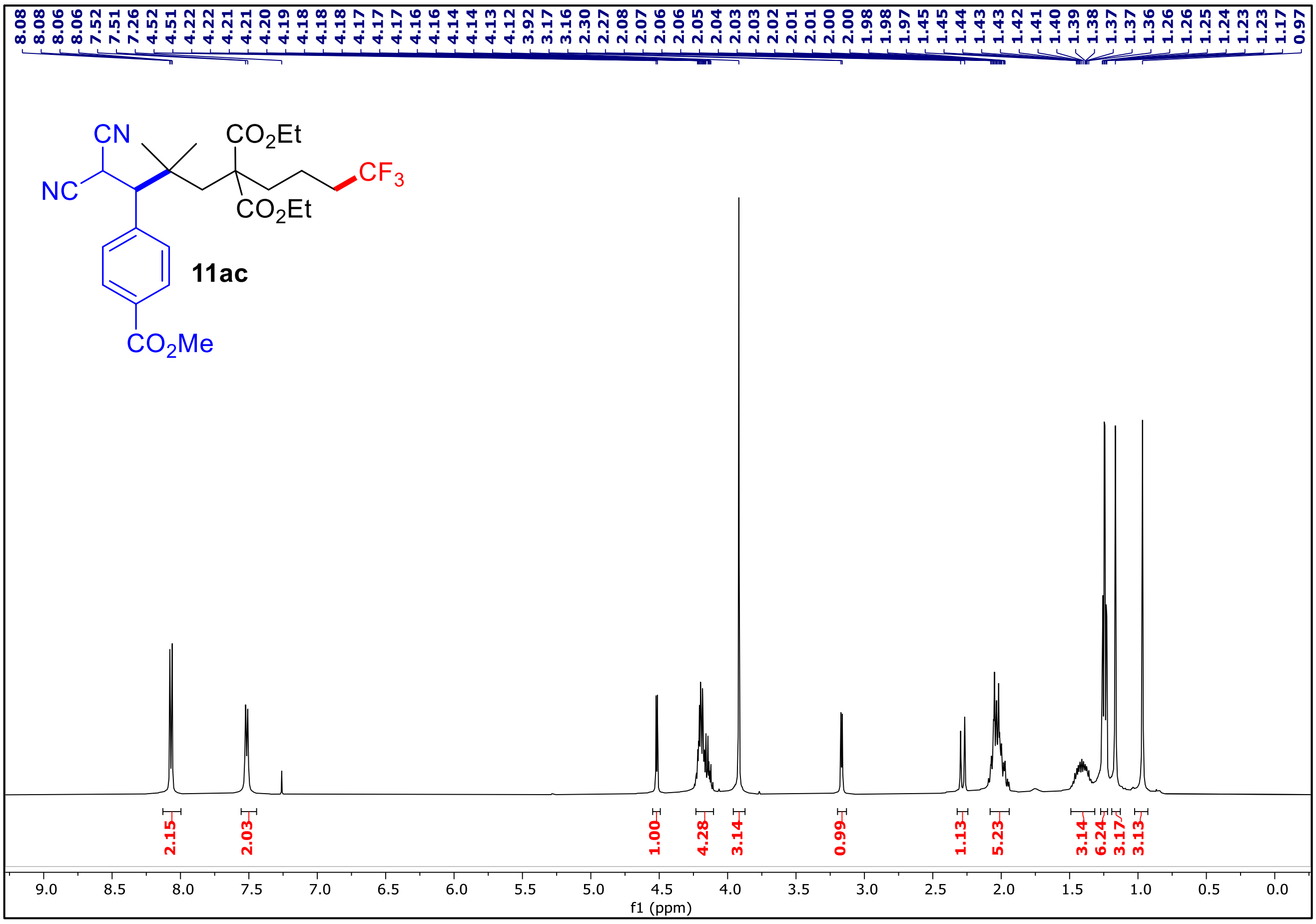

^{1}H NMR of compound **11ac** (500 MHz, $CDCl_3$)

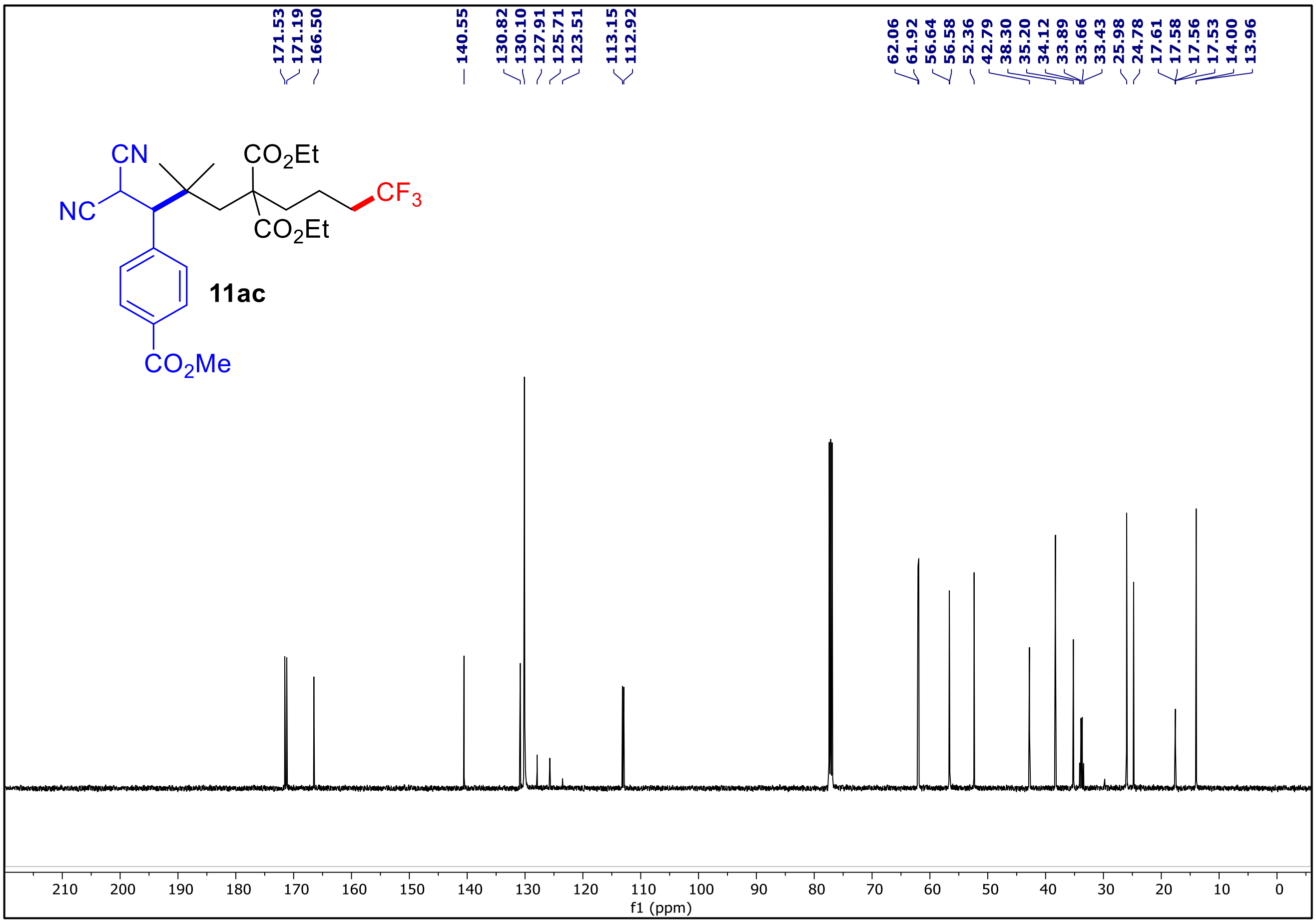

$^{13}C\{^1H\}$ NMR of compound **11ac** (126 MHz, $CDCl_3$)

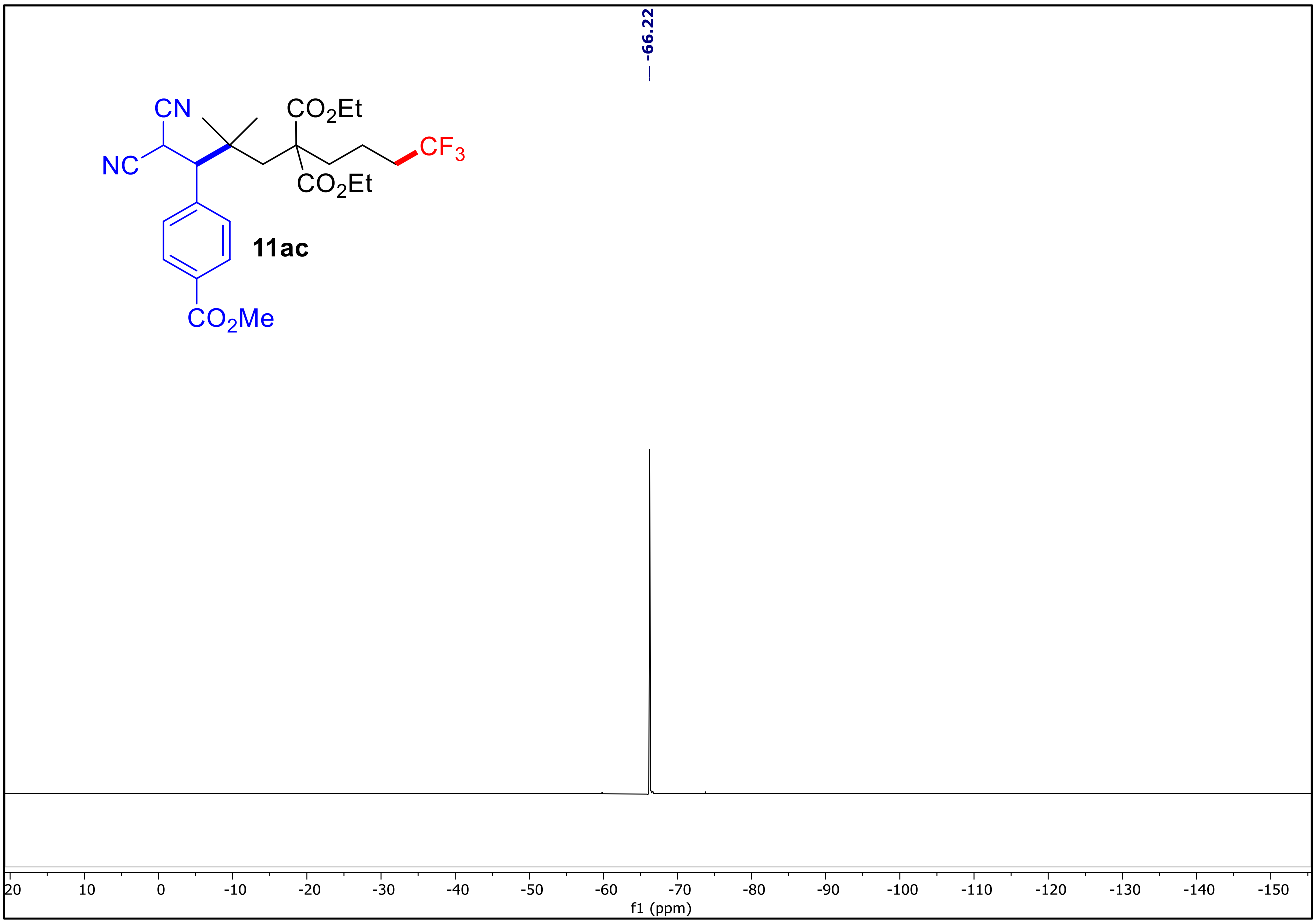

^{19}F NMR of compound **11ac** (471 MHz, $CDCl_3$)

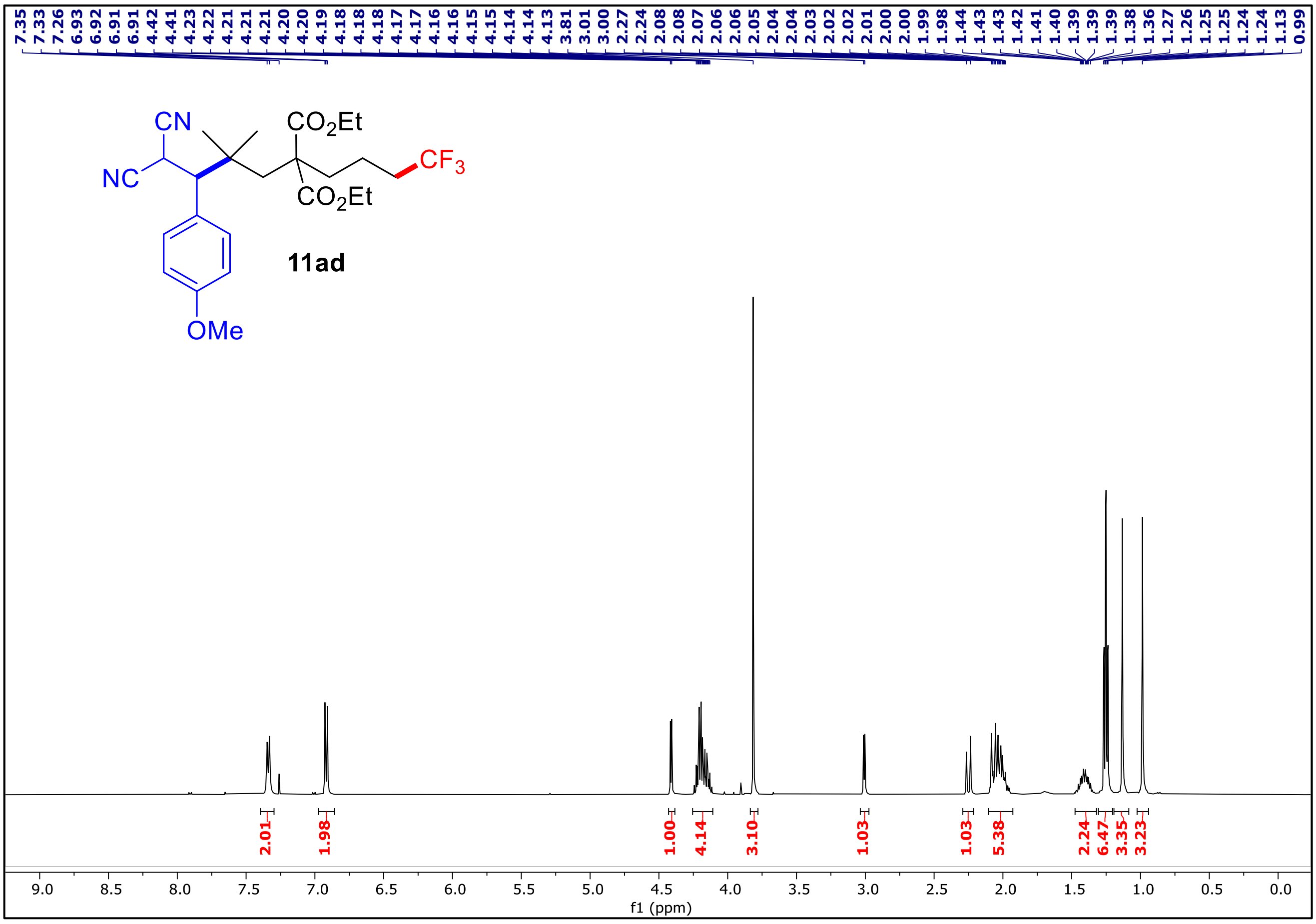

^1H NMR of compound **11ad** (500 MHz, $CDCl_3$)

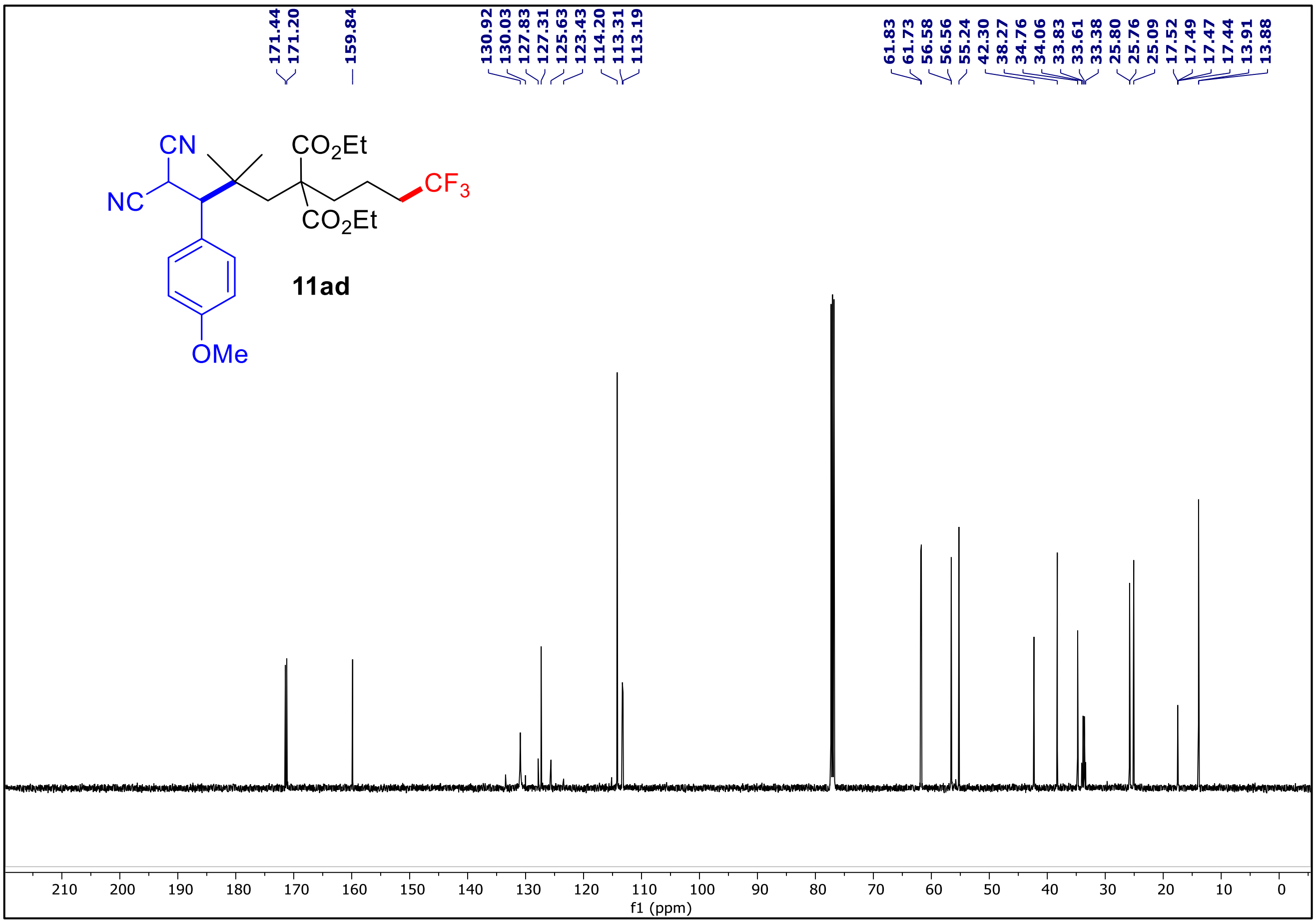

$^{13}C\{^1H\}$ NMR of compound **11ad** (126 MHz, $CDCl_3$)

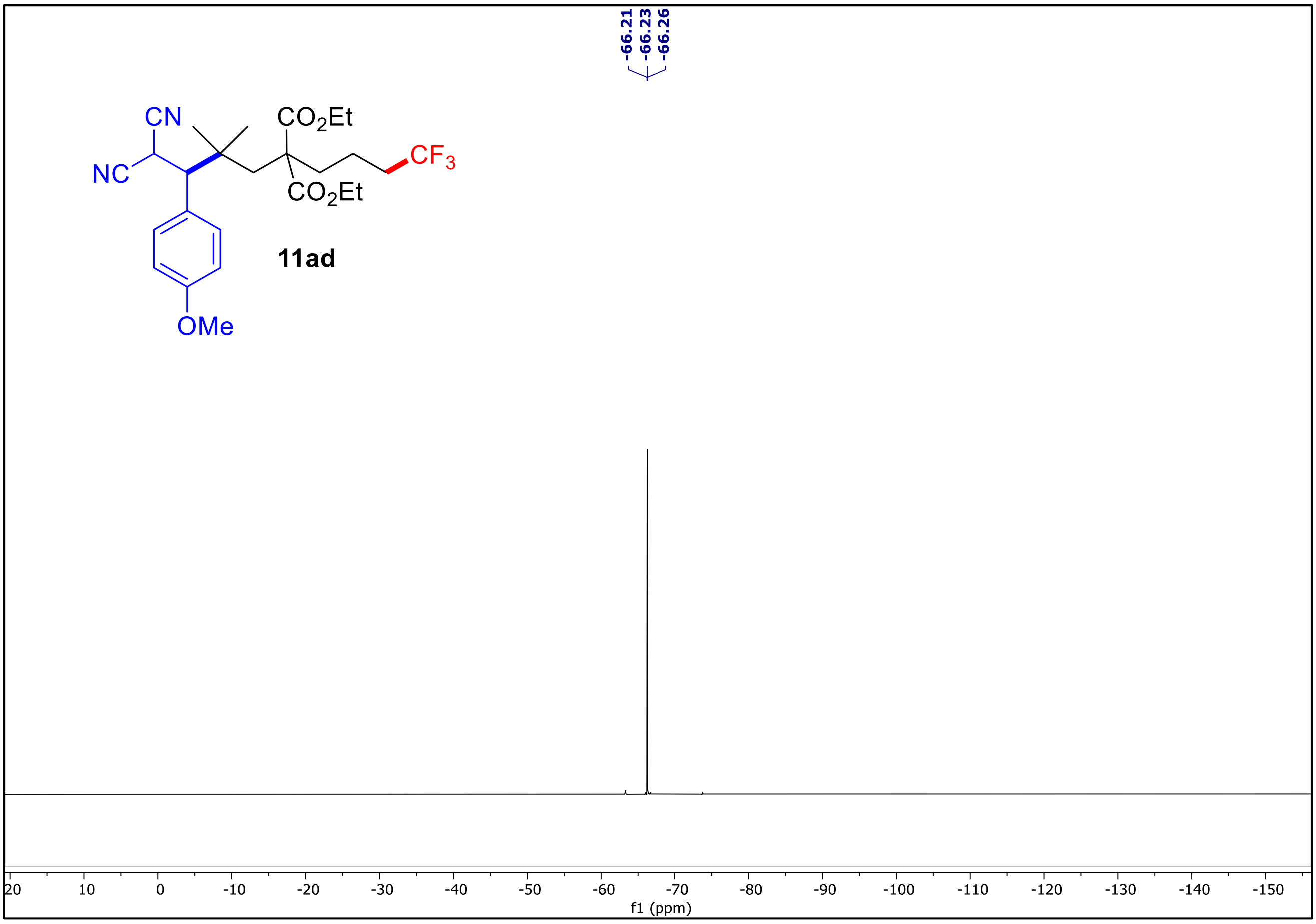

^{19}F NMR of compound **11ad** (471 MHz, $CDCl_3$)

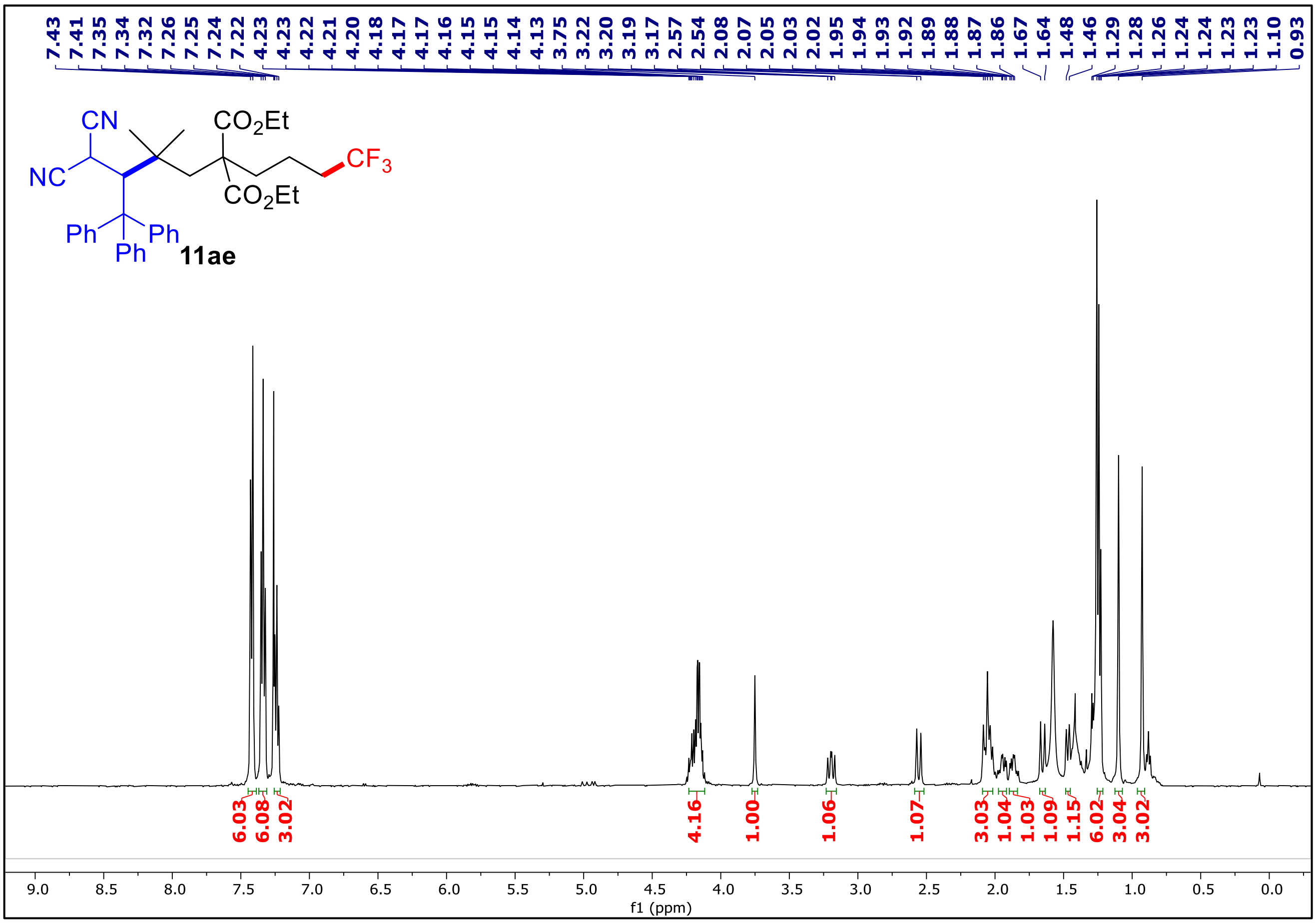

^{1}H NMR of compound **11ae** (500 MHz, $CDCl_3$)

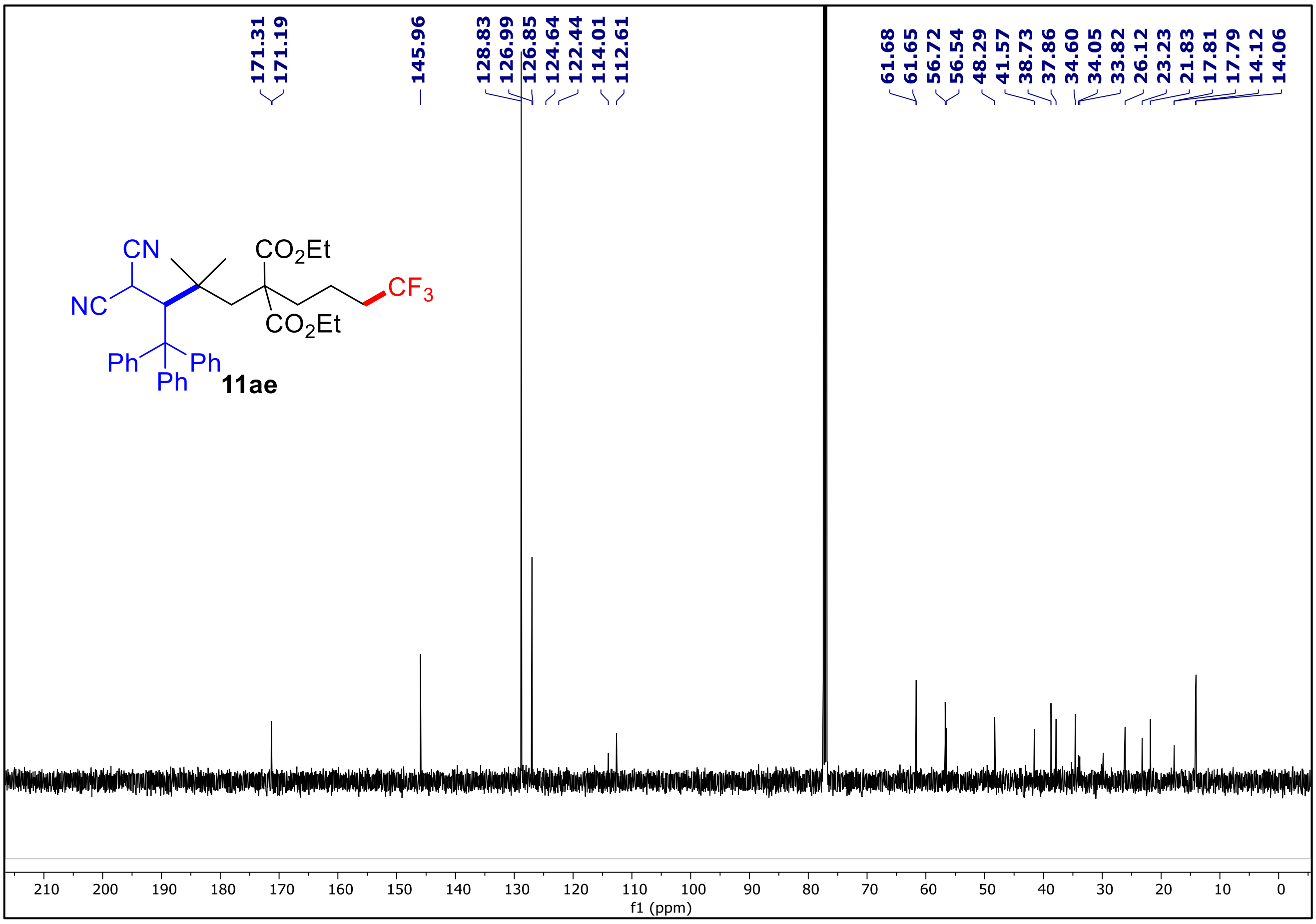

$^{13}C\{^1H\}$ NMR of compound **11ae** (126 MHz, $CDCl_3$)

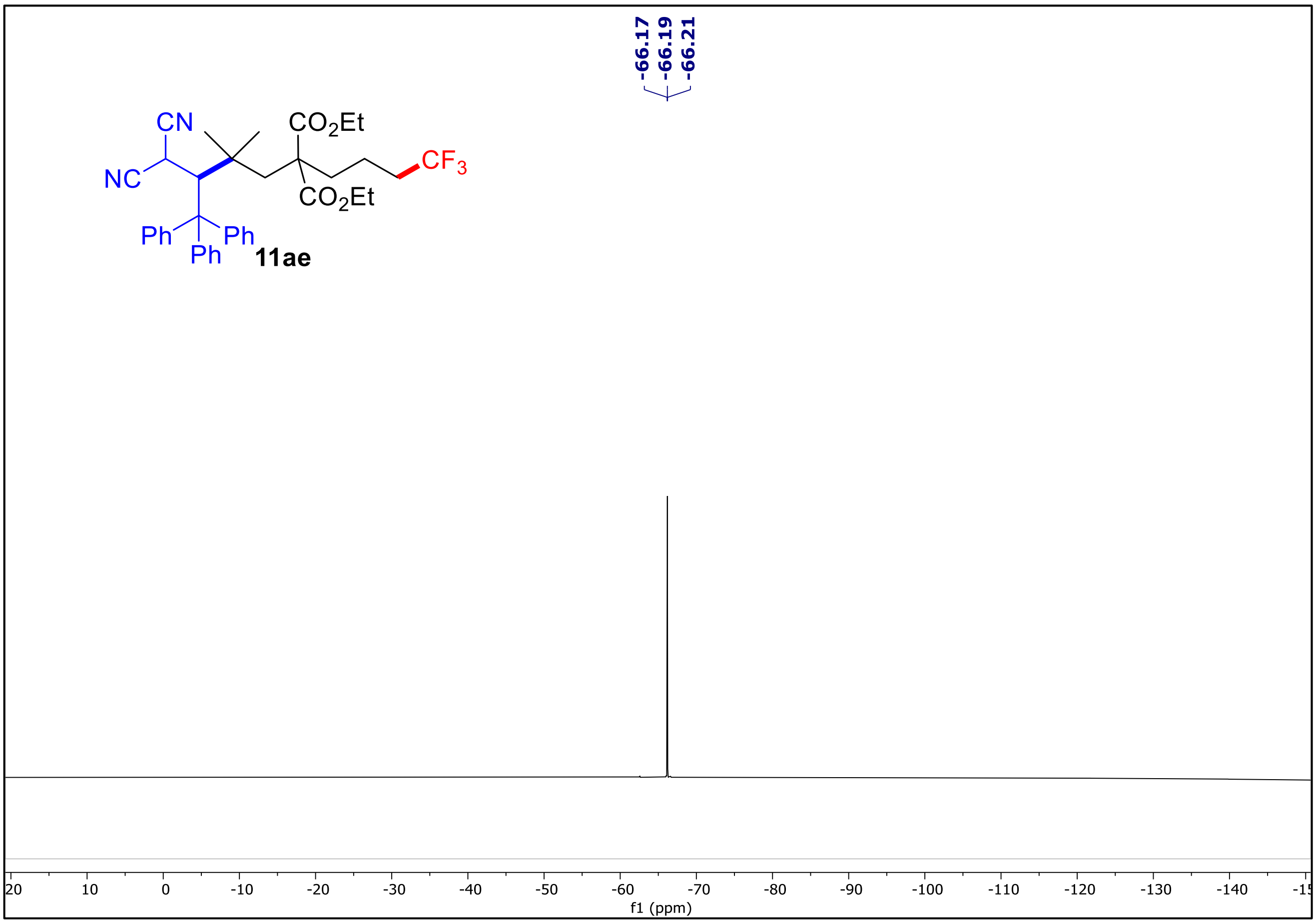

^{19}F NMR of compound **11ae** (471 MHz, $CDCl_3$)

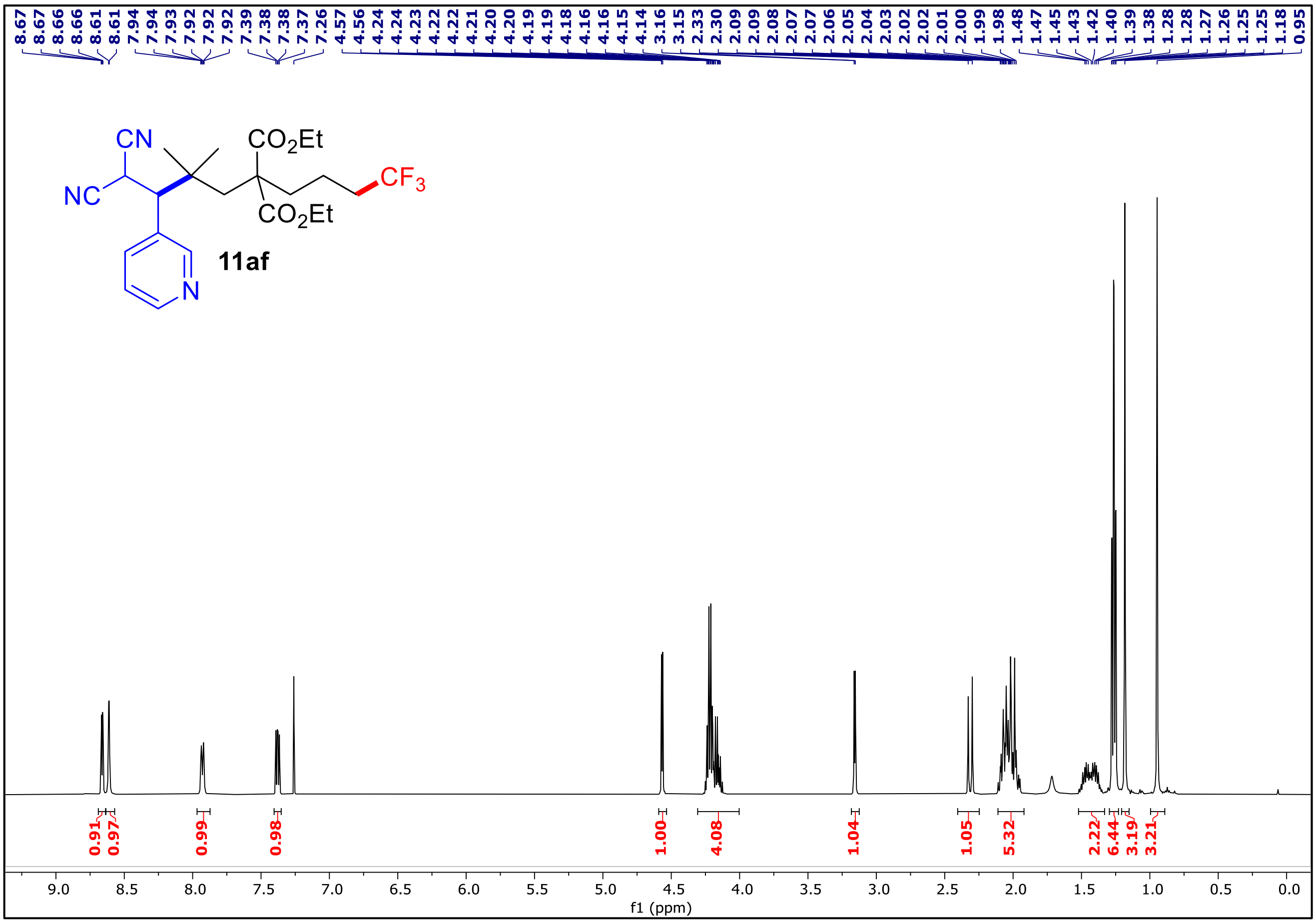

^{1}H NMR of compound **11af** (500 MHz, $CDCl_3$)

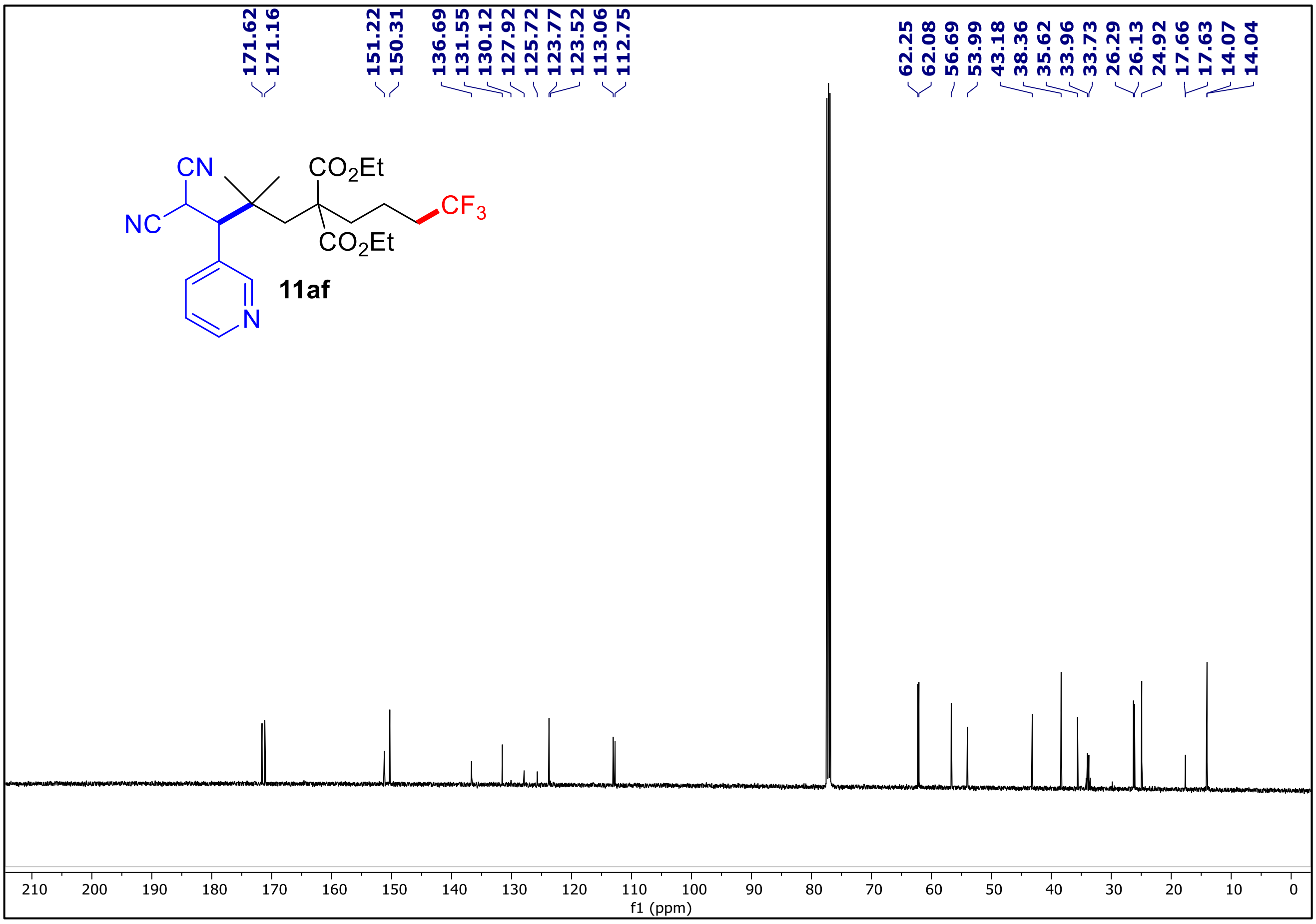

^{13}C{^{1}H} NMR of compound **11af** (126 MHz, $CDCl_3$)

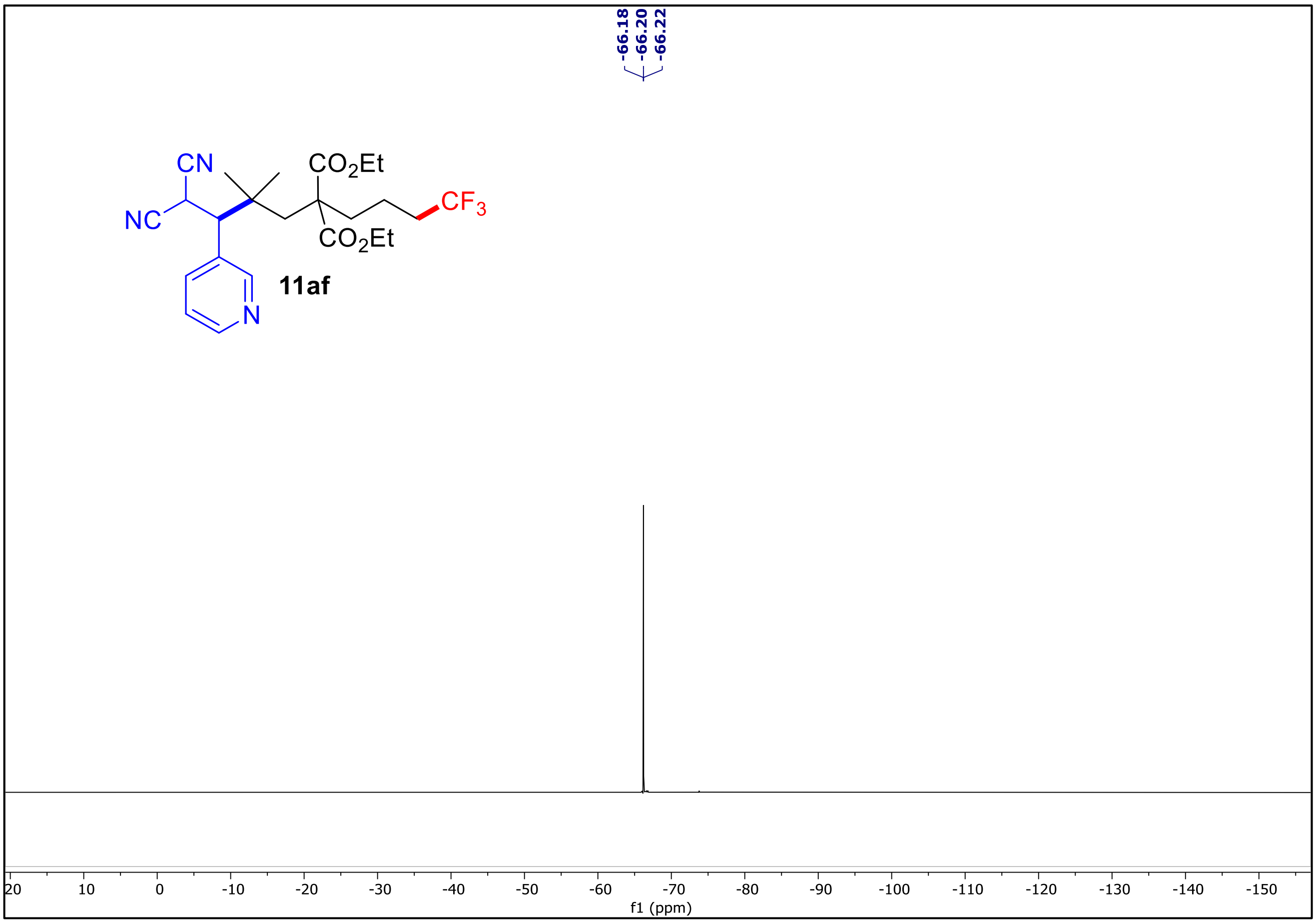

^{19}F NMR of compound **11af** (471 MHz, $CDCl_3$)

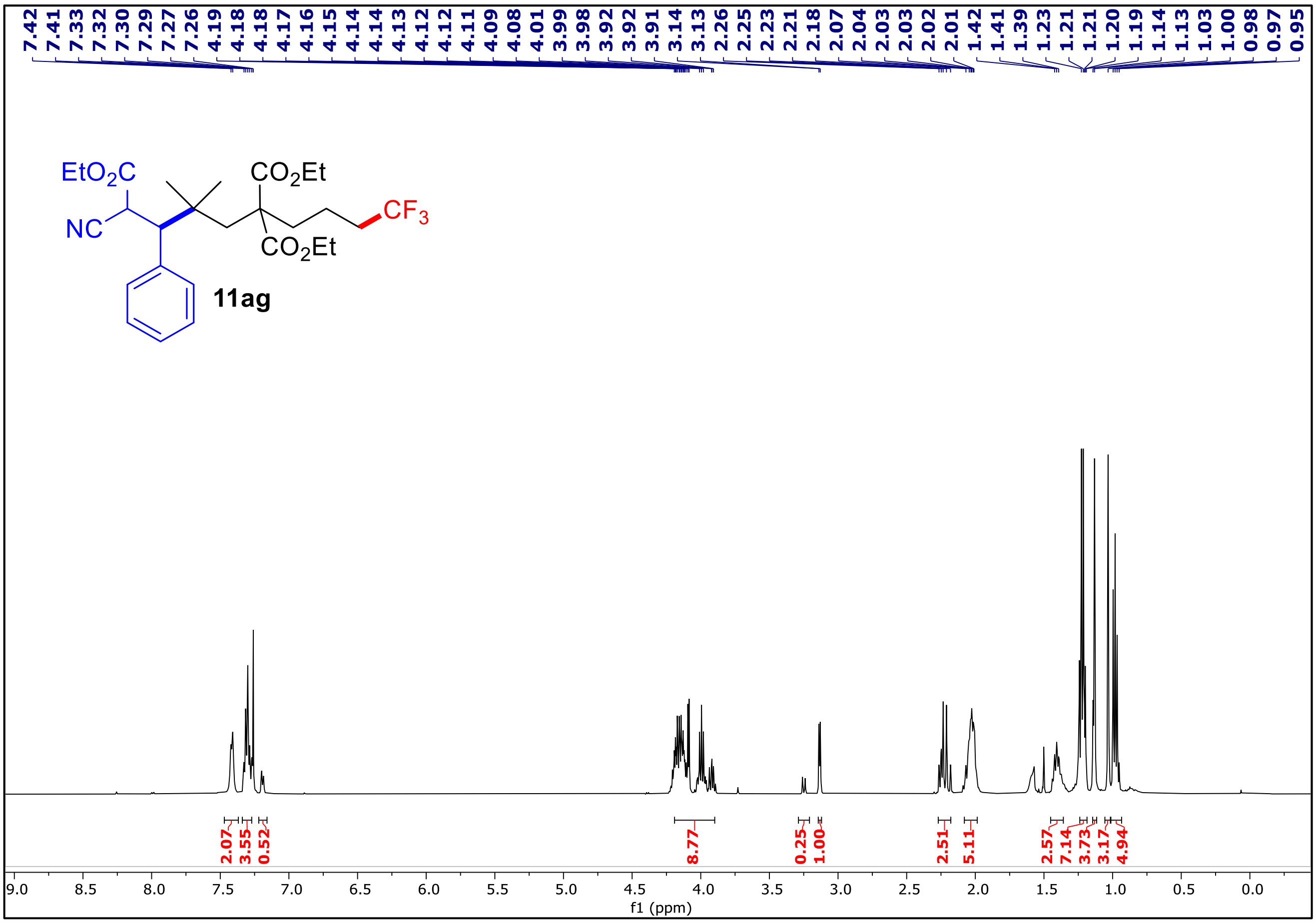

^{1}H NMR of compound **11ag** (500 MHz, $CDCl_3$)

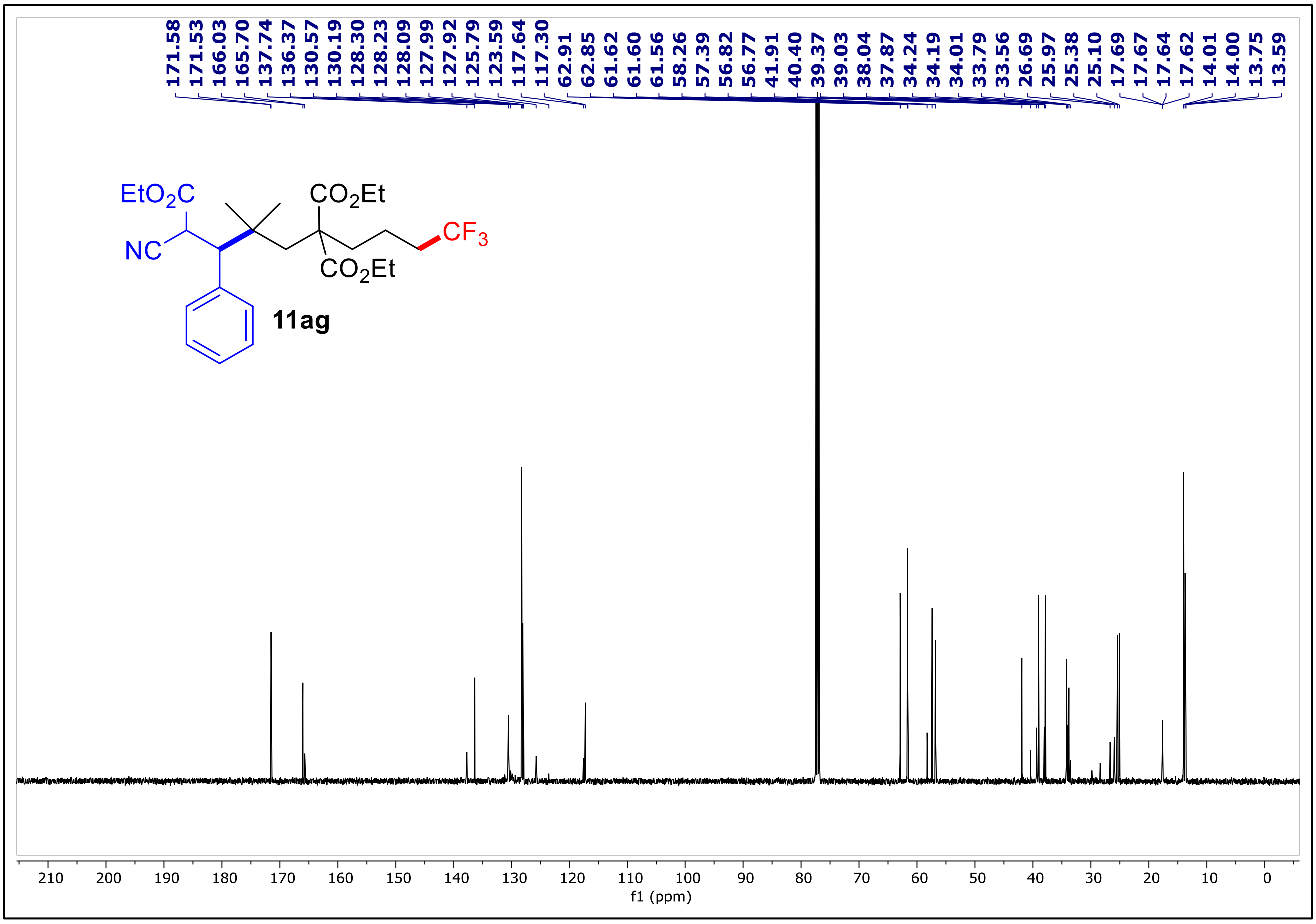

$^{13}C\{^{1}H\}$ NMR of compound **11ag** (126 MHz, $CDCl_3$)

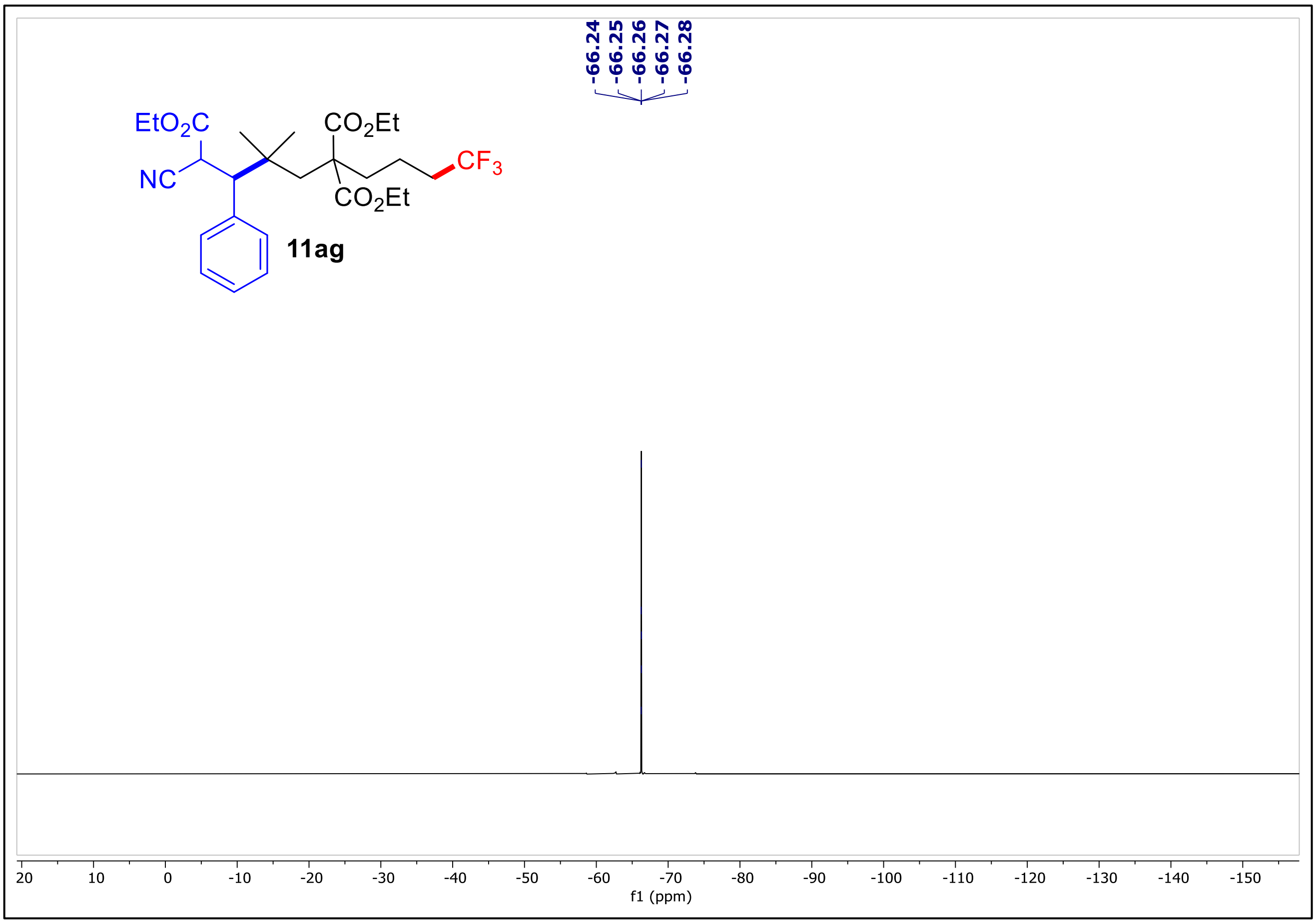

^{19}F NMR of compound **11ag** (471 MHz, $CDCl_3$)

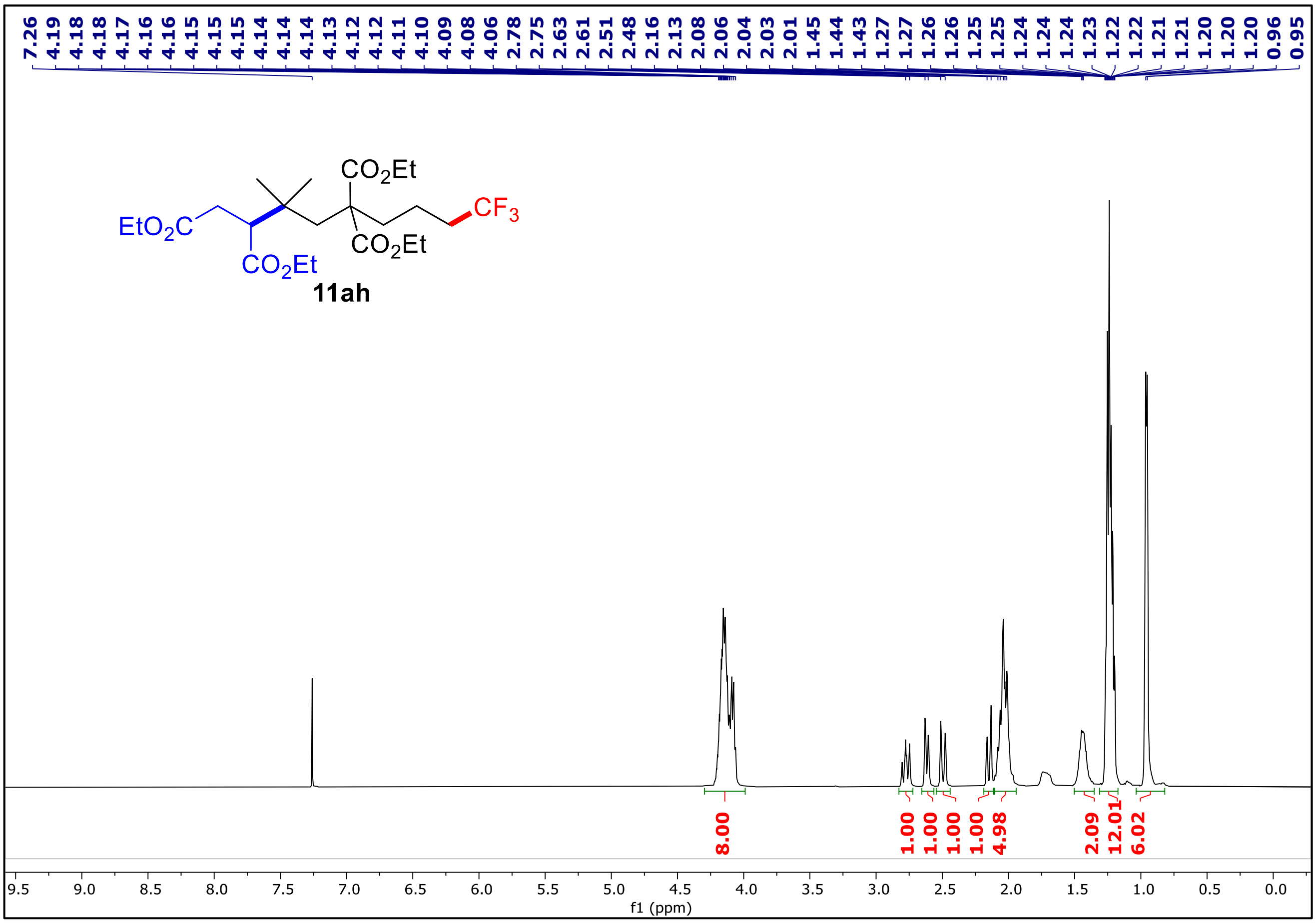

^{1}H NMR of compound **11ah** (500 MHz, $CDCl_3$)

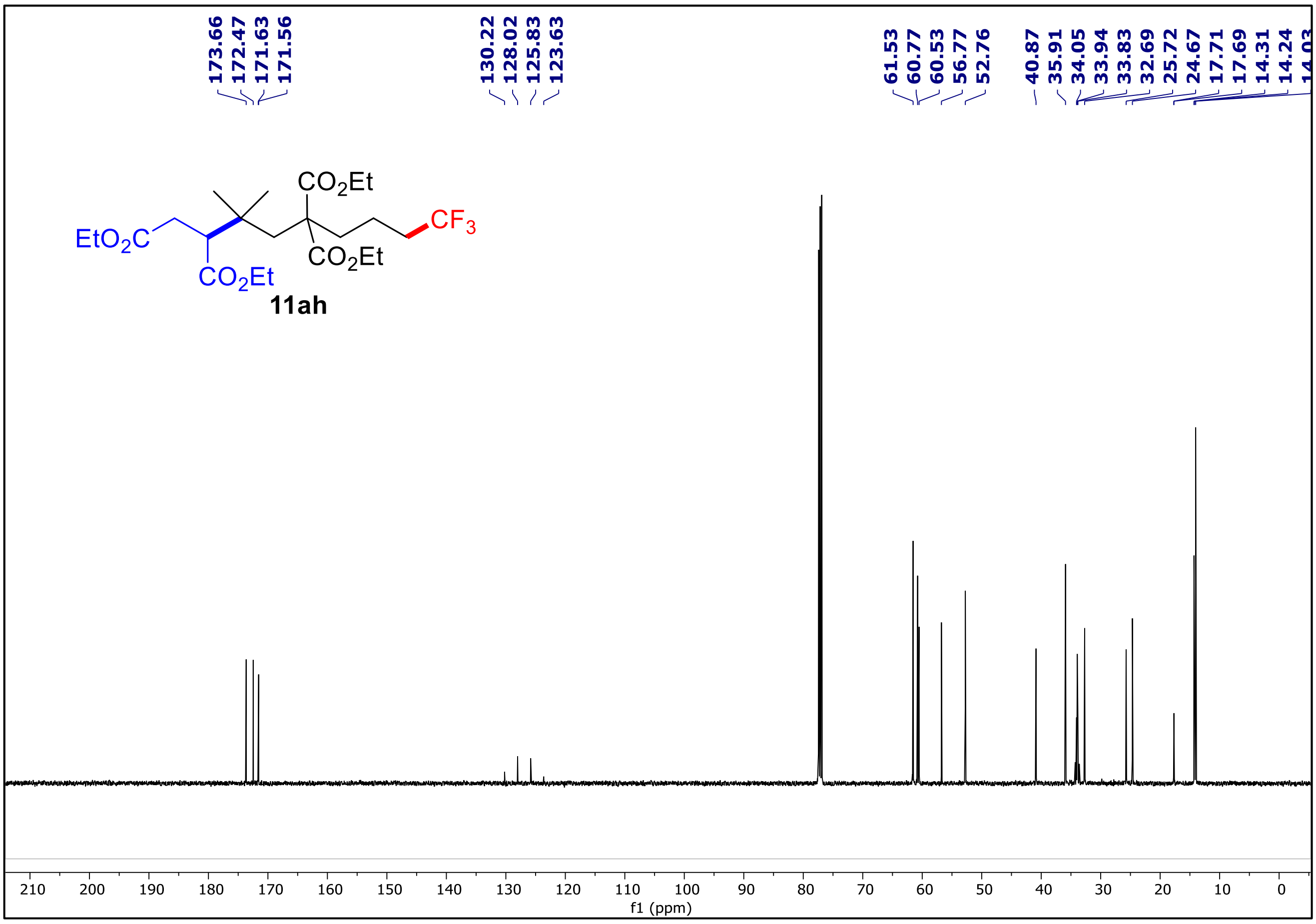

$^{13}C\{^1H\}$ NMR of compound **11ah** (126 MHz, $CDCl_3$)

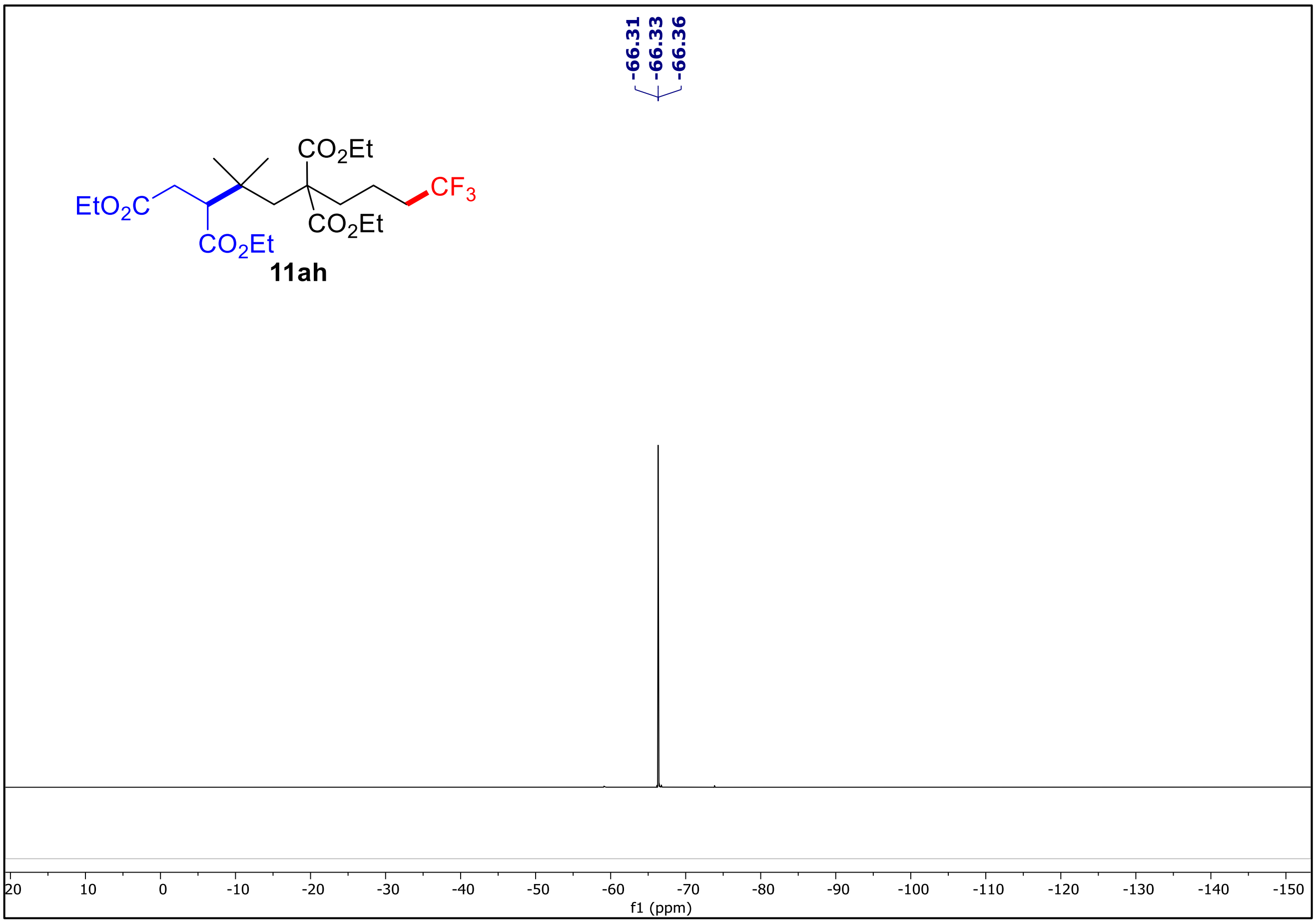

^{19}F NMR of compound **11ah** (471 MHz, $CDCl_3$)

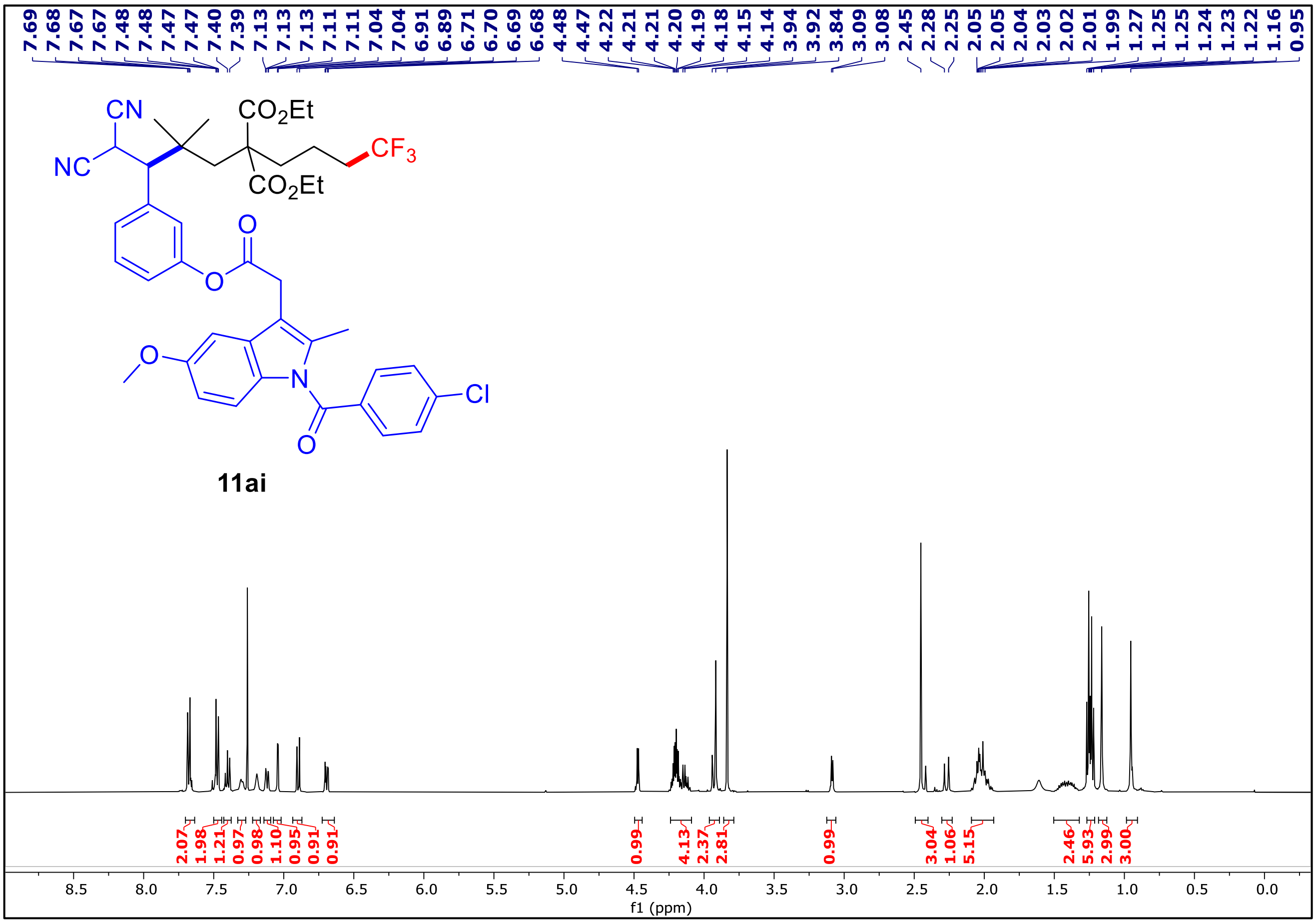

^{1}H NMR of compound **11ai** (500 MHz, $CDCl_3$)

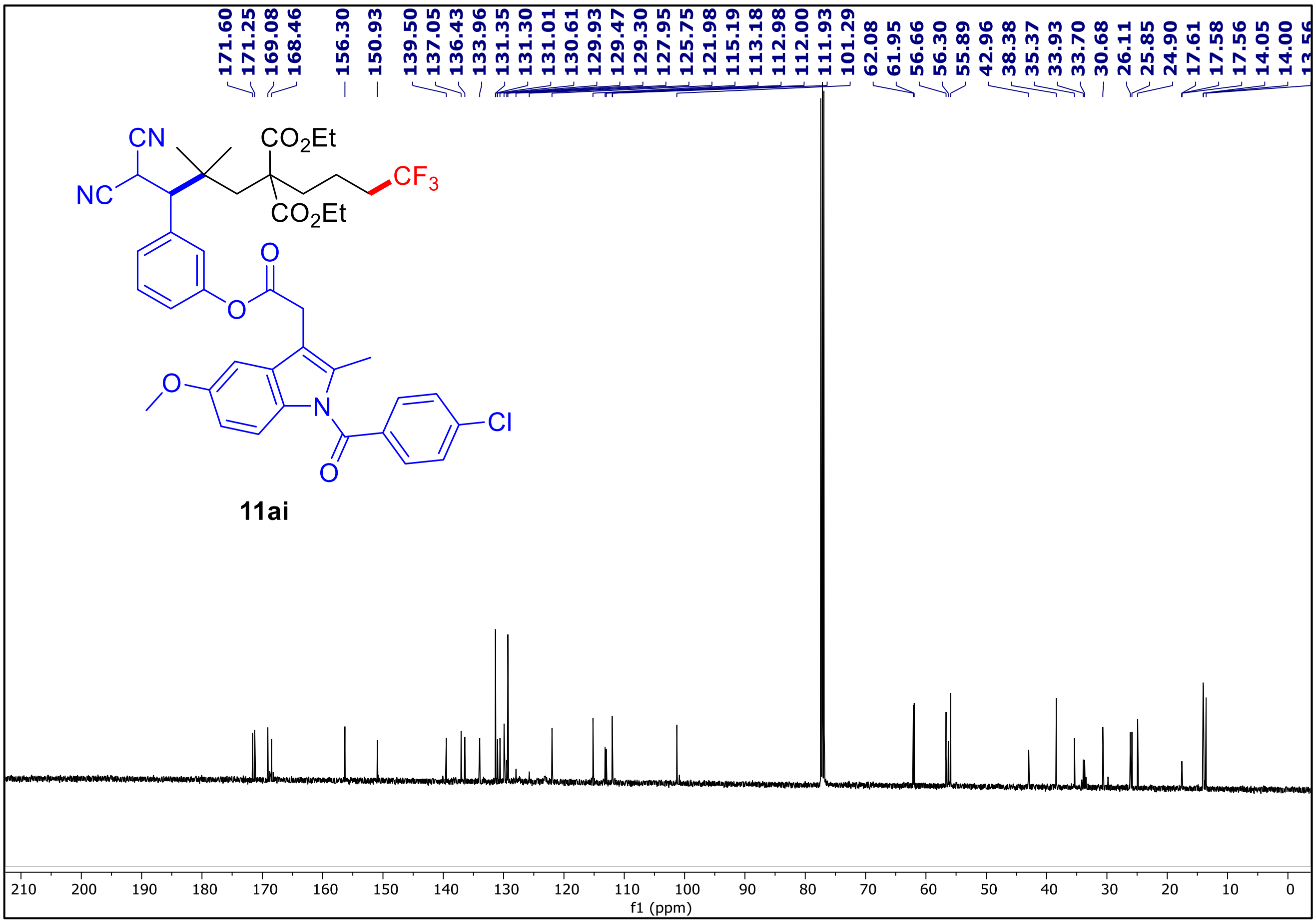

$^{13}C\{^{1}H\}$ NMR of compound **11ai** (126 MHz, $CDCl_3$)

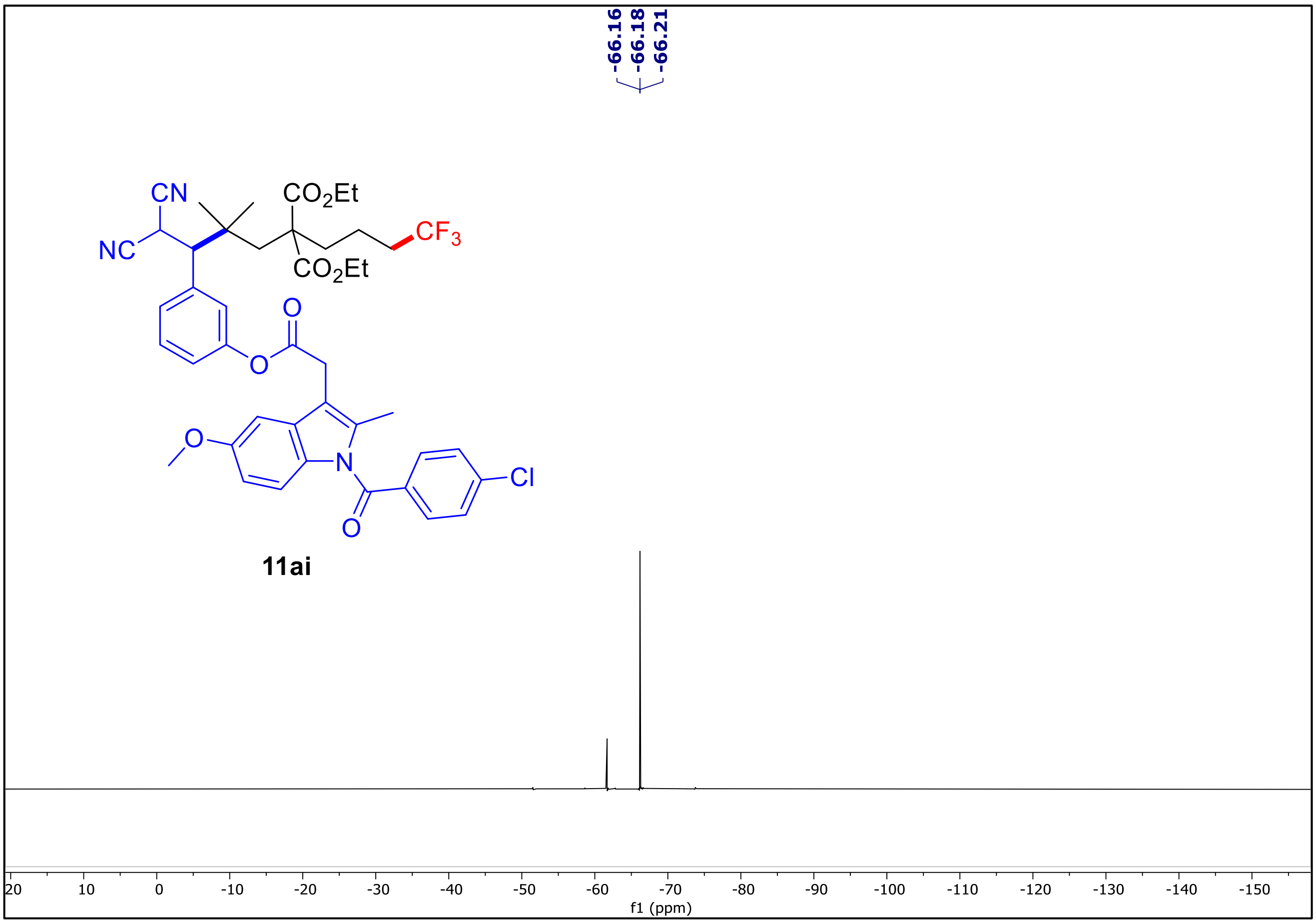

^{19}F NMR of compound **11ai** (471 MHz, $CDCl_3$)

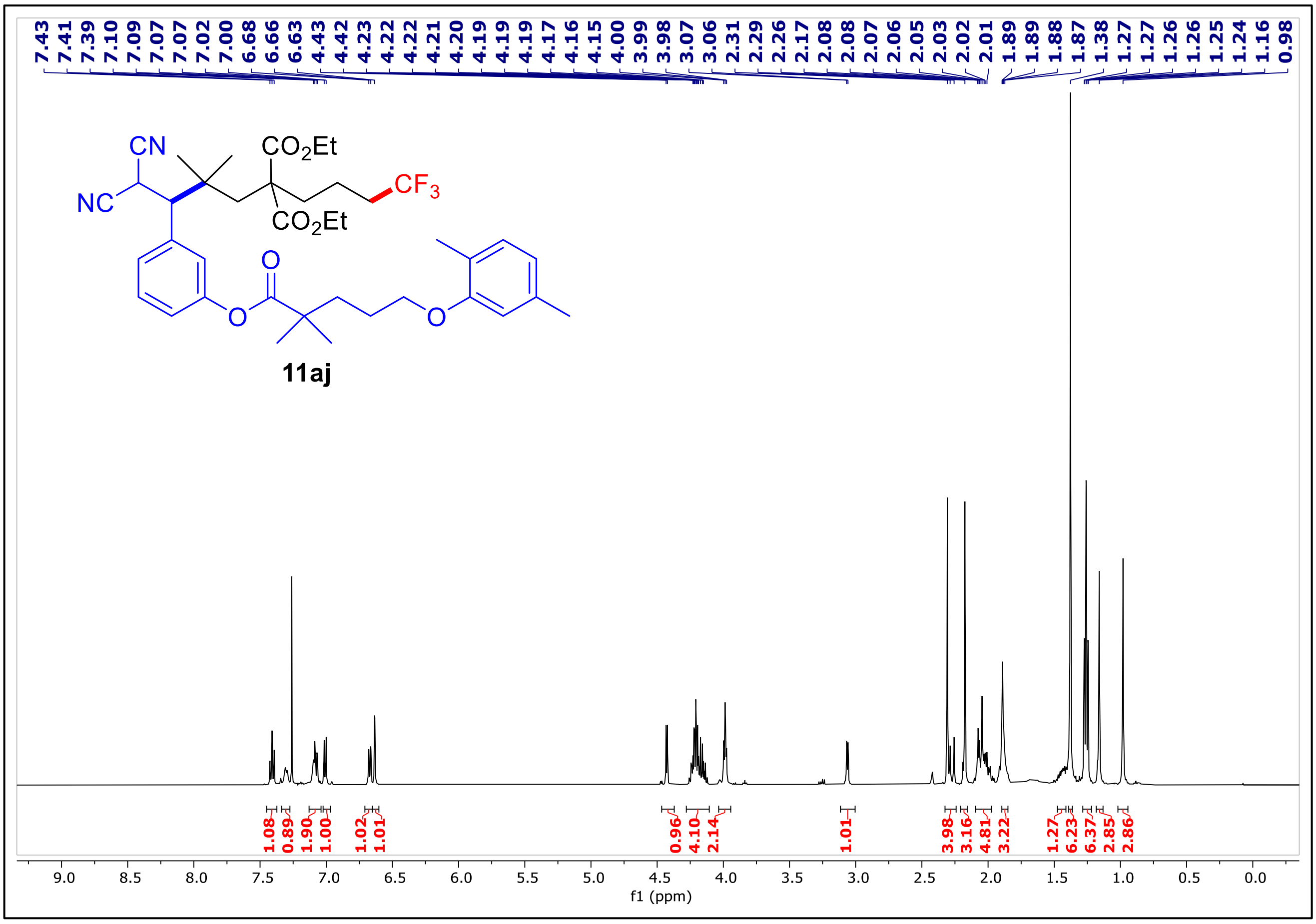

^{1}H NMR of compound **11aj** (500 MHz, $CDCl_3$)

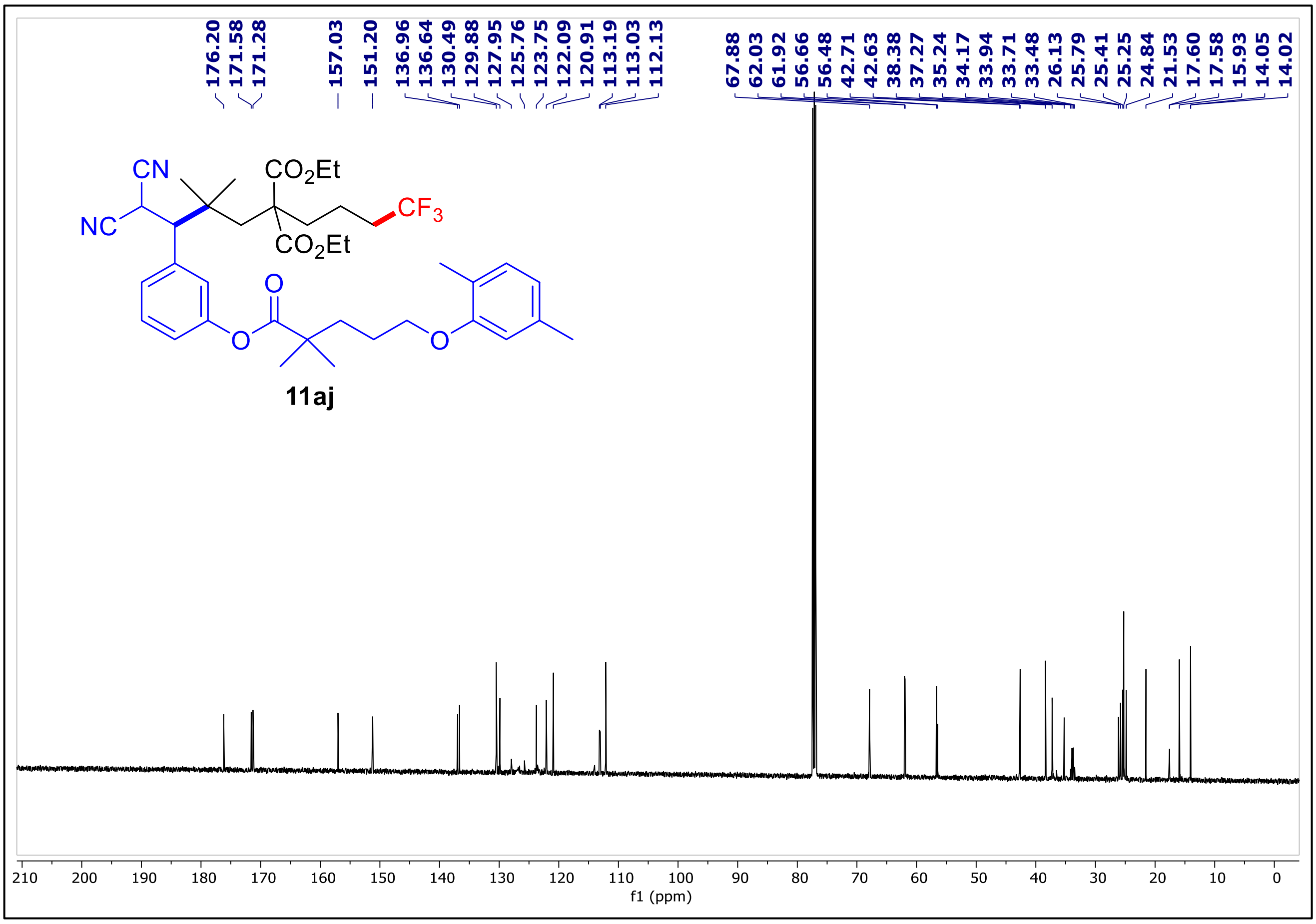

^{13}C{^{1}H} NMR of compound **11aj** (126 MHz, $CDCl_3$)

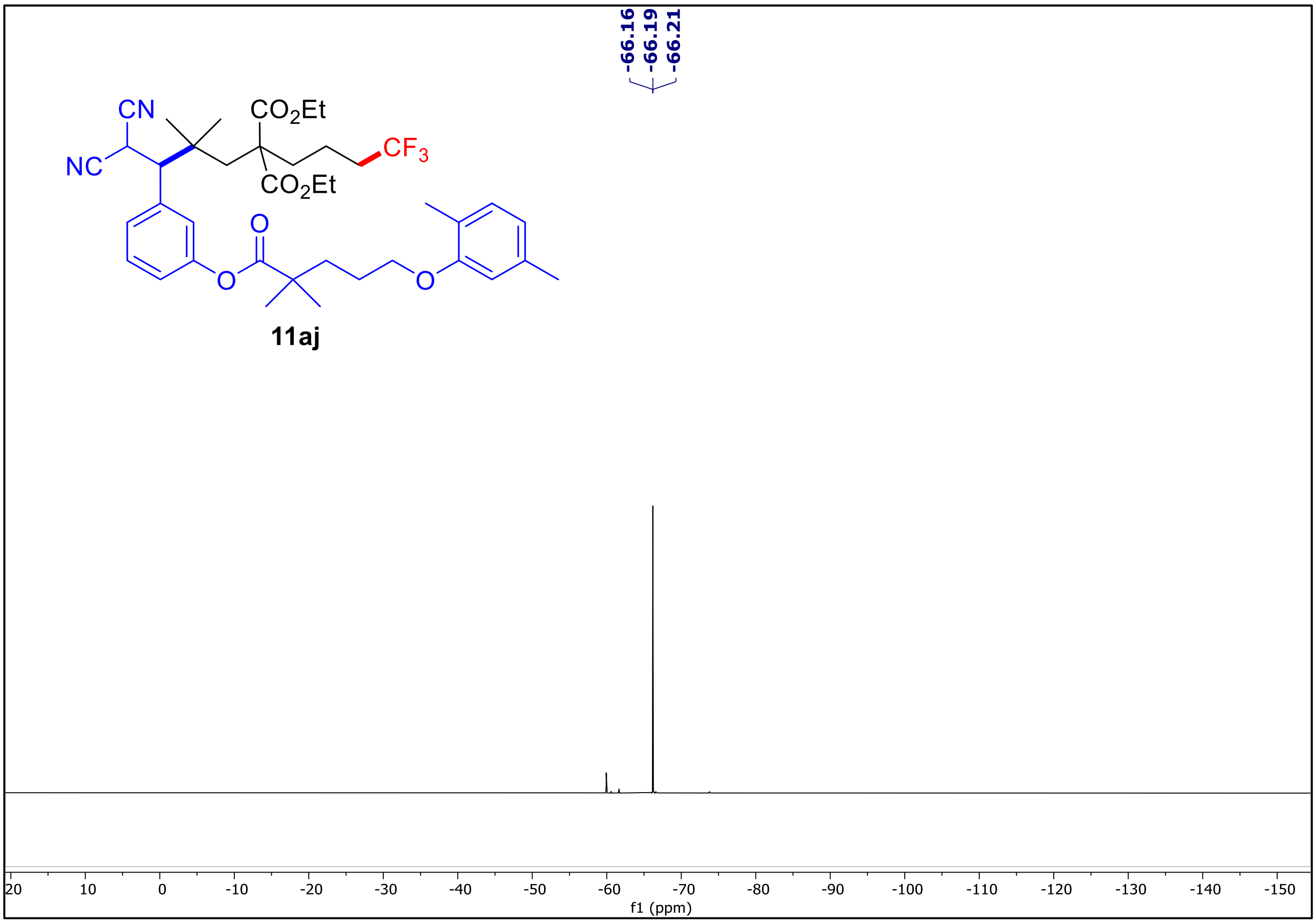

^{19}F NMR of compound **11aj** (471 MHz, $CDCl_3$)

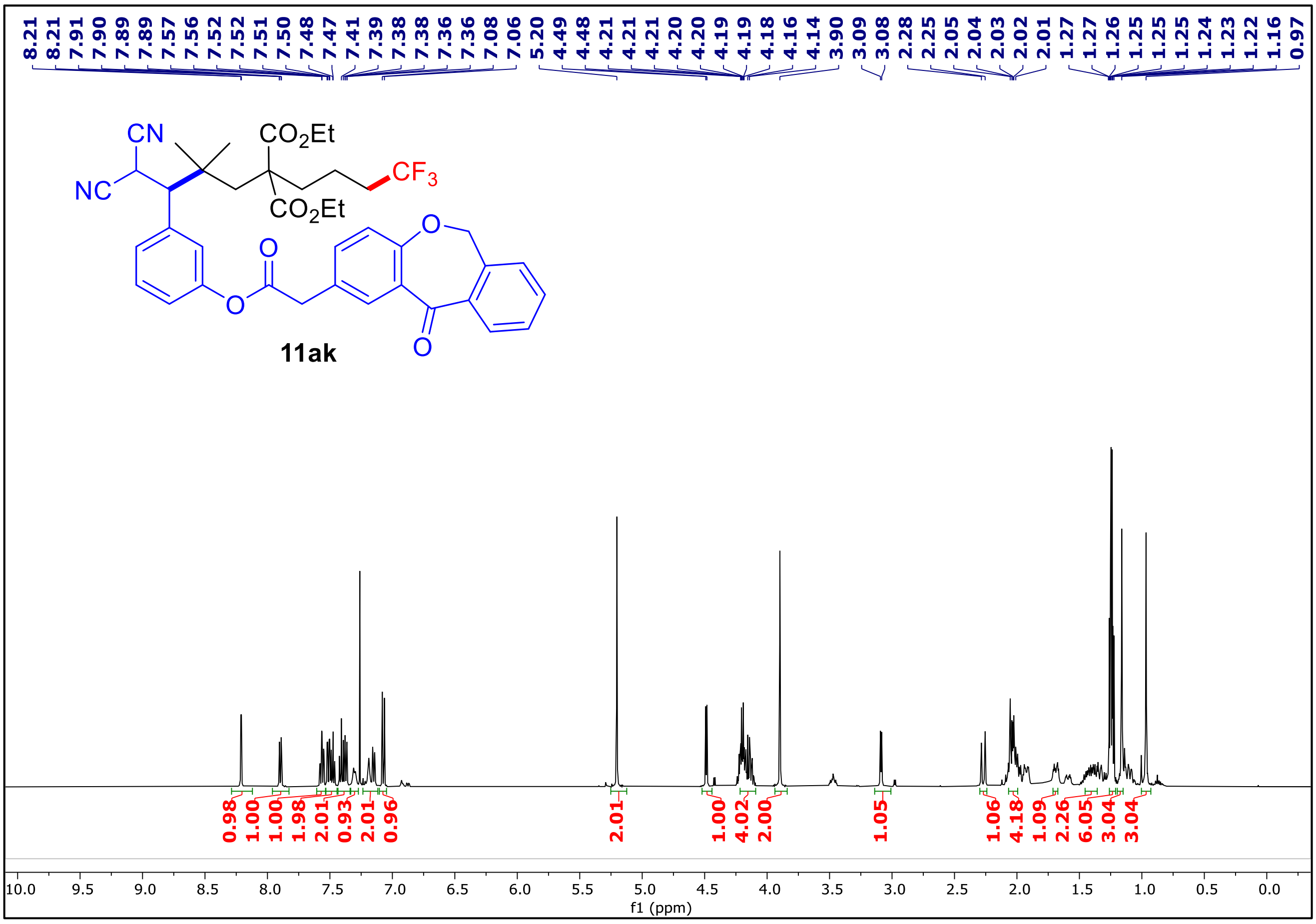

^{1}H NMR of compound **11ak** (500 MHz, $CDCl_3$)

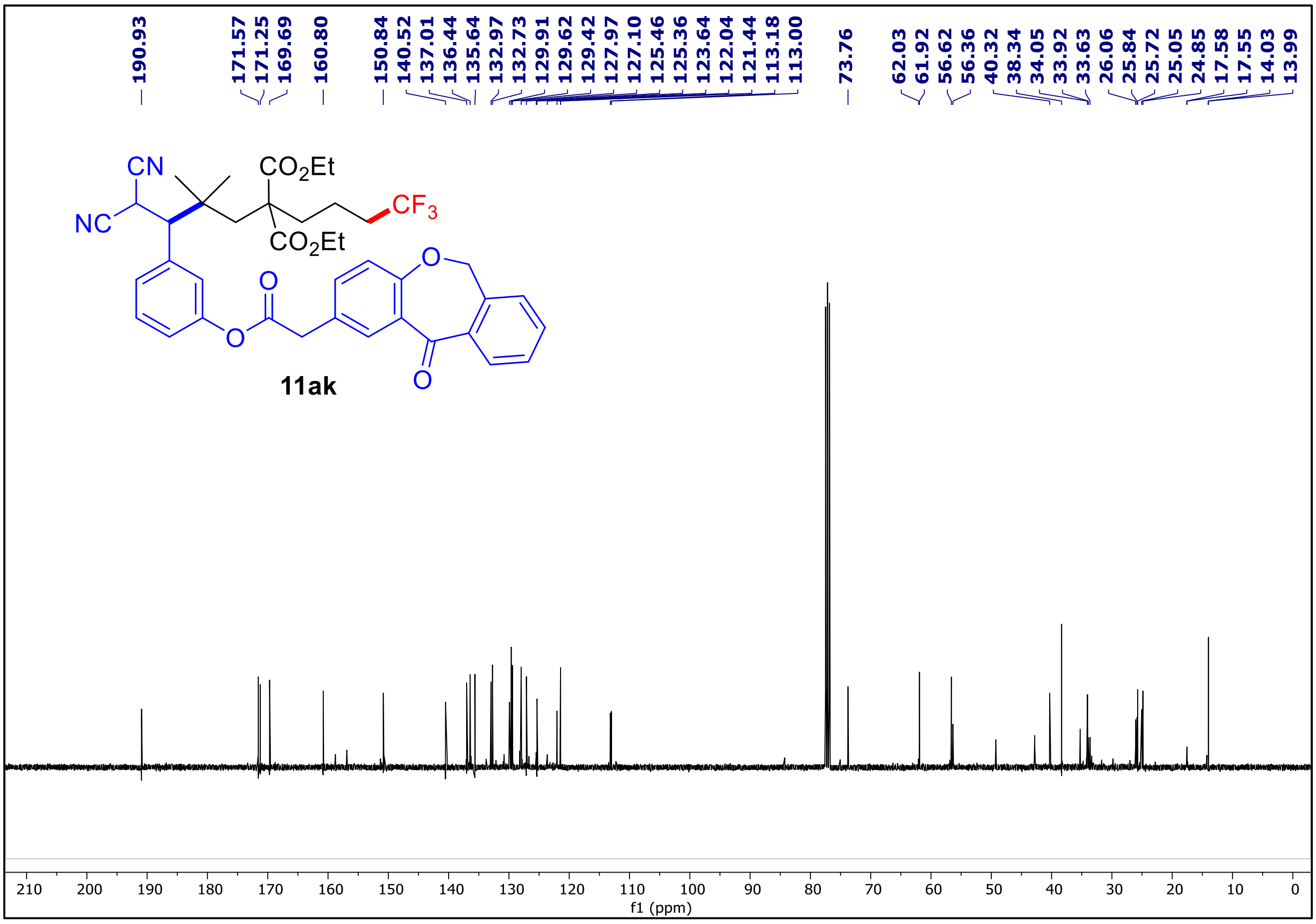

$^{13}C\{^{1}H\}$ NMR of compound **11ak** (126 MHz, $CDCl_3$)

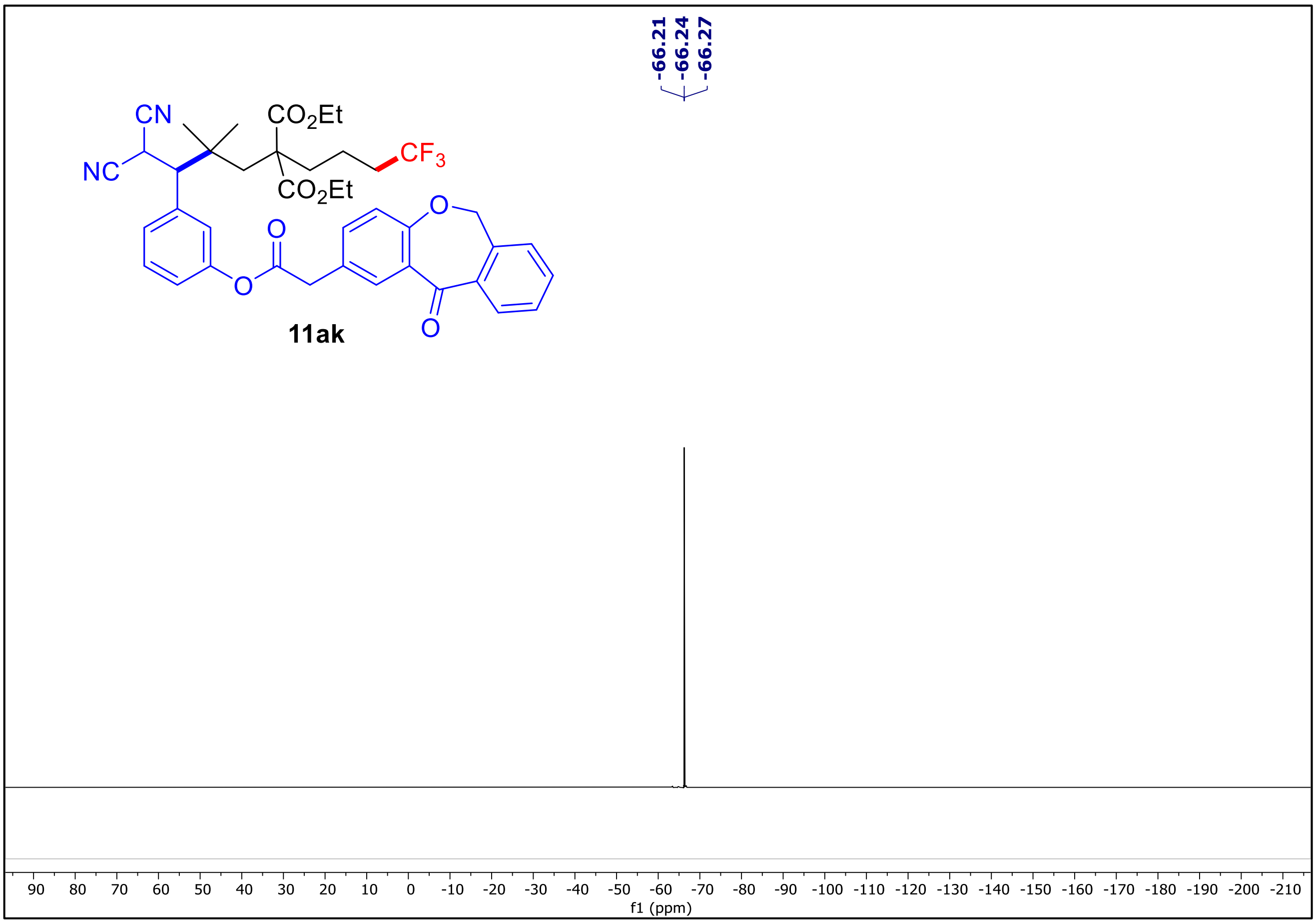

^{19}F NMR of compound **11ak** (376 MHz, $CDCl_3$)

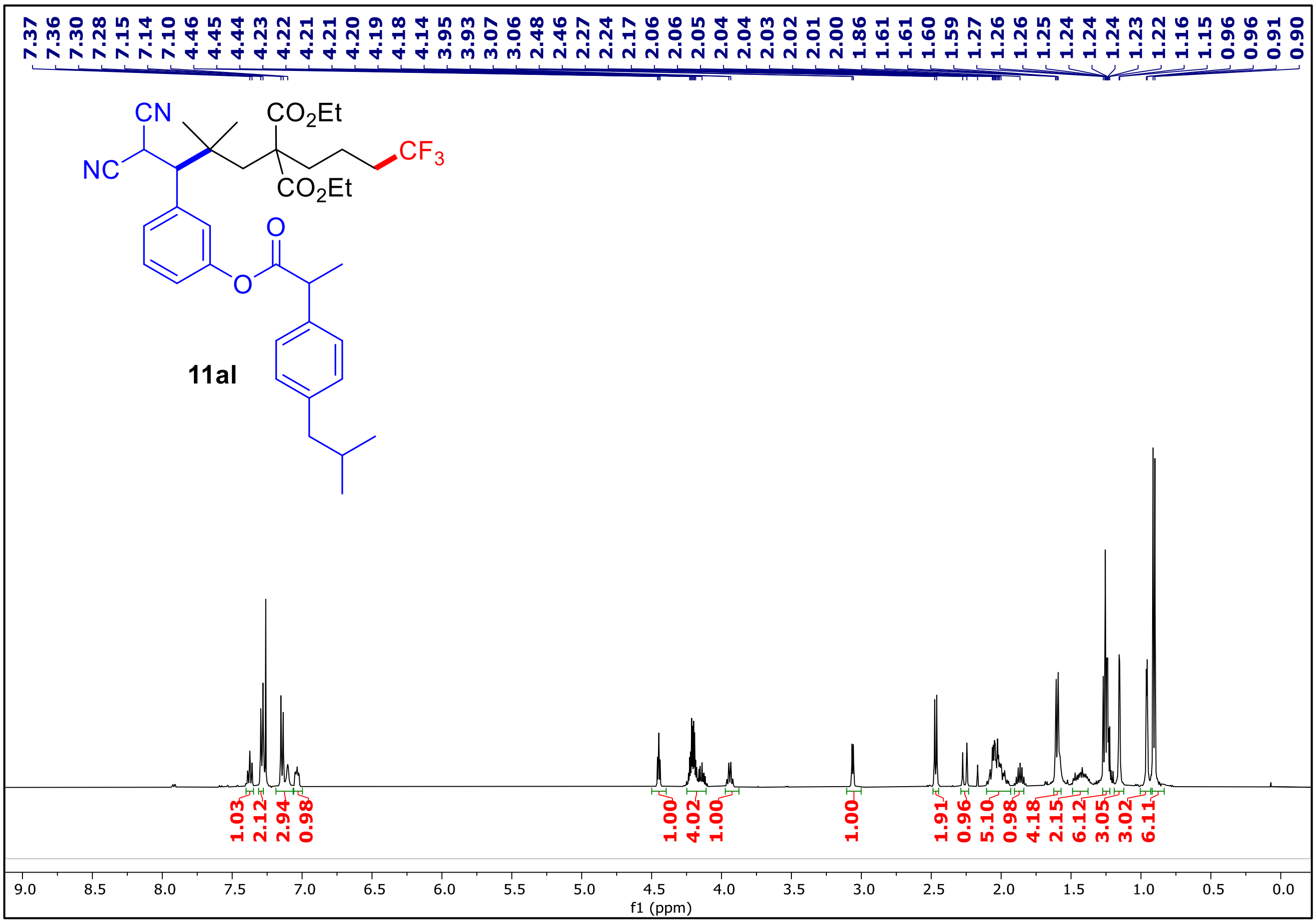

^{1}H NMR of compound **11al** (500 MHz, $CDCl_3$)

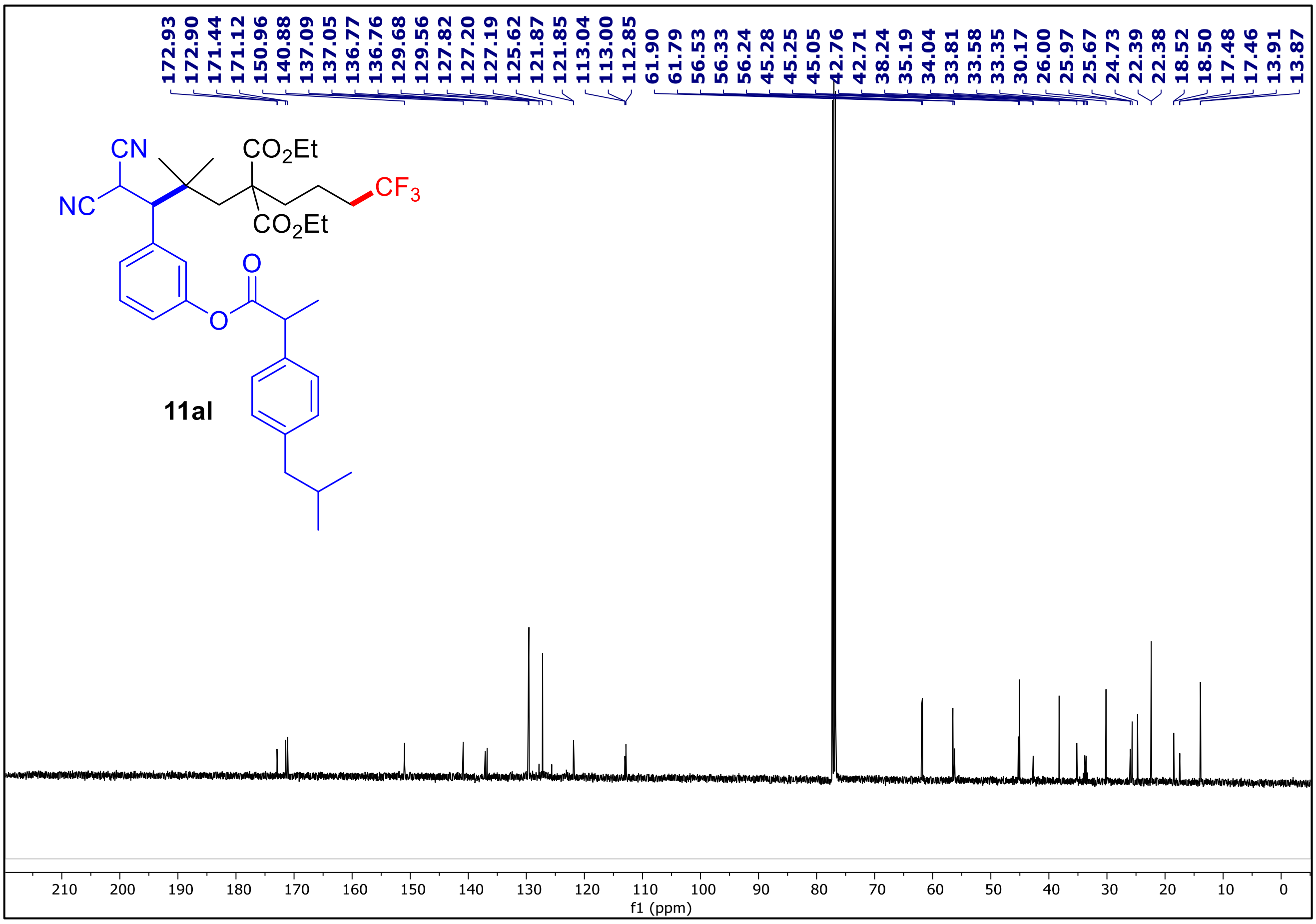

$^{13}C\{^1H\}$ NMR of compound **11al** (126 MHz, $CDCl_3$)

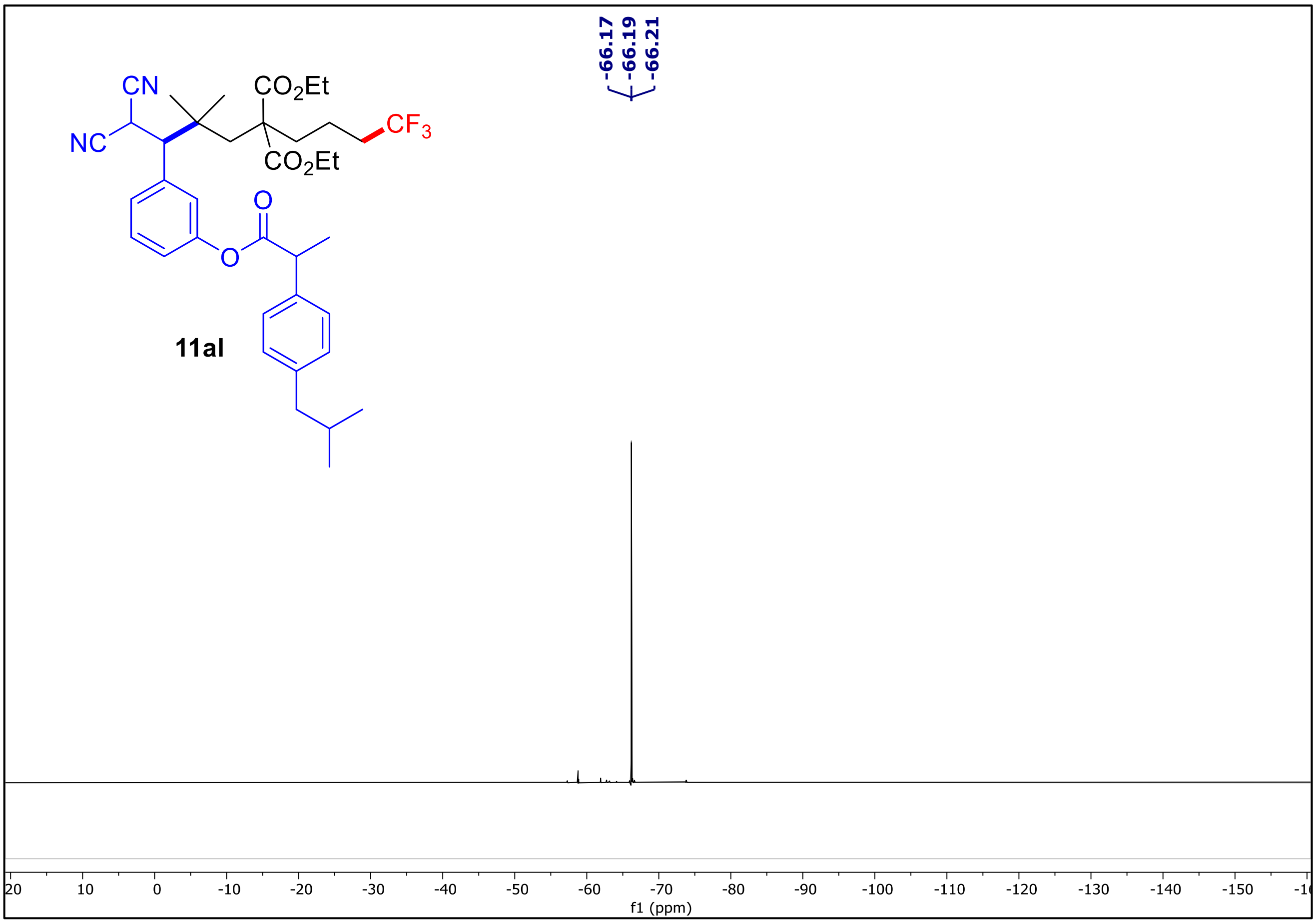

^{19}F NMR of compound **11al** (471 MHz, $CDCl_3$)

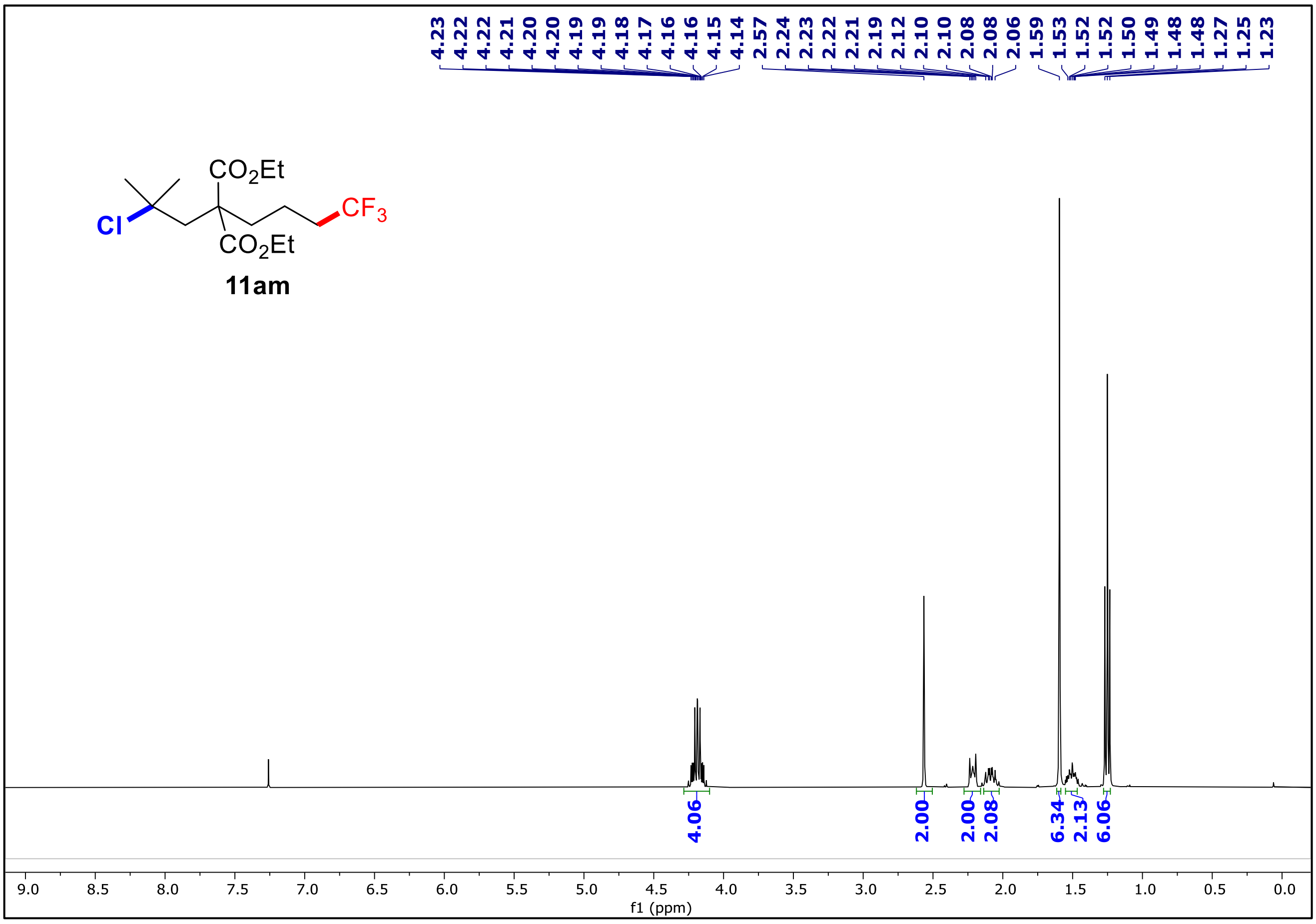

^{1}H NMR of compound **11am** (400 MHz, $CDCl_3$)

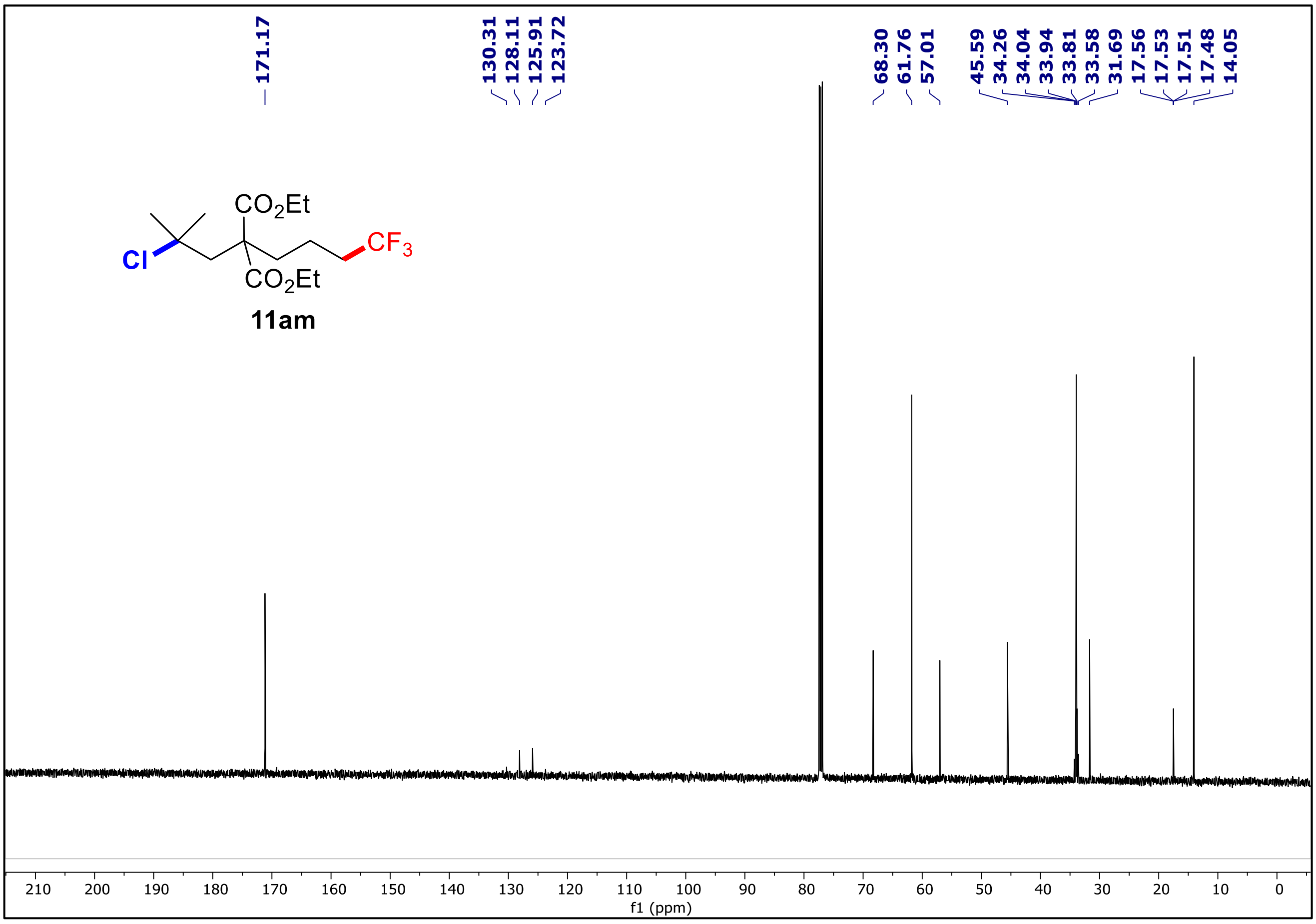

$^{13}C\{^{1}H\}$ NMR of compound **11am** (126 MHz, $CDCl_3$)

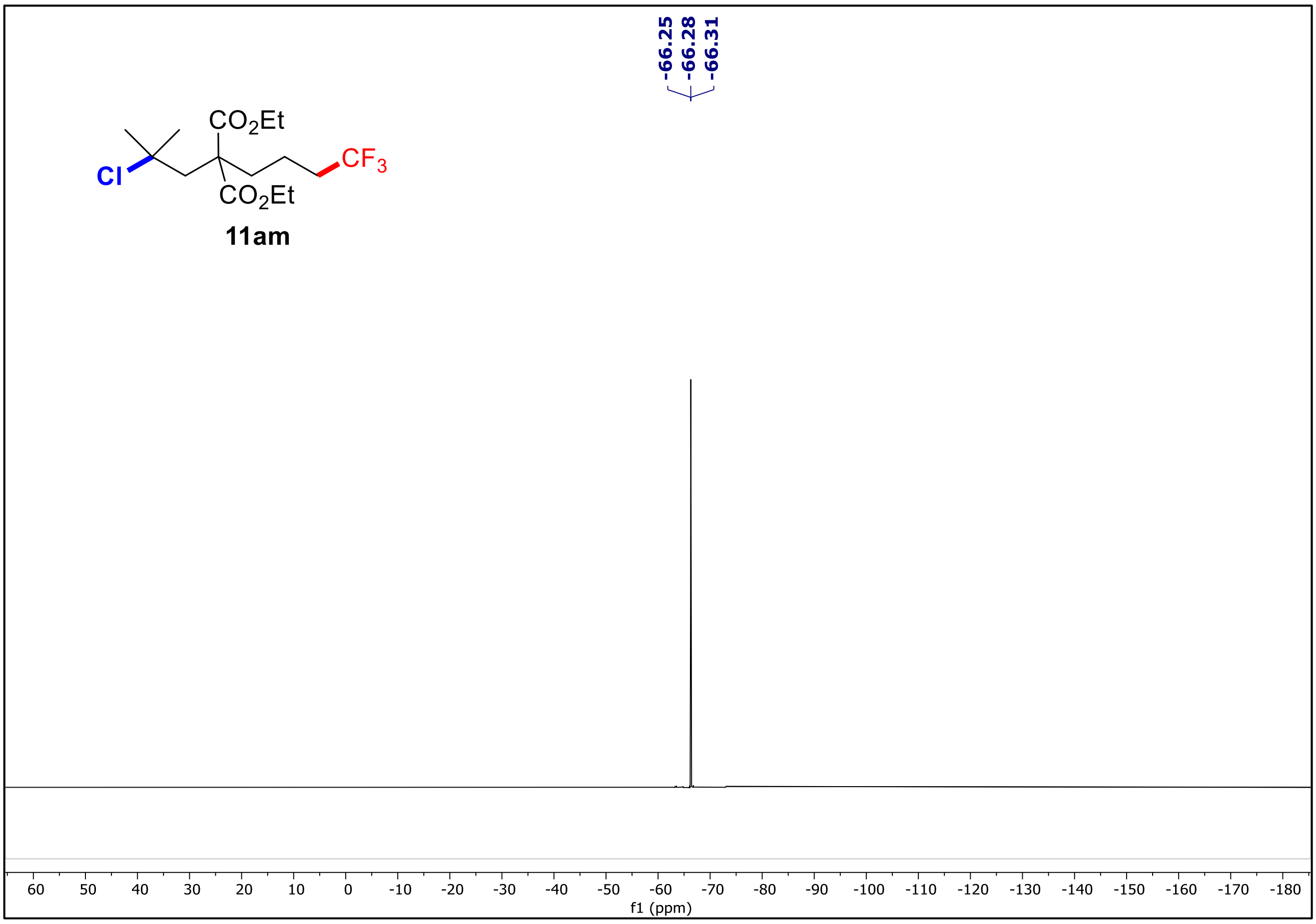

^{19}F NMR of compound **11am** (376 MHz, $CDCl_3$)

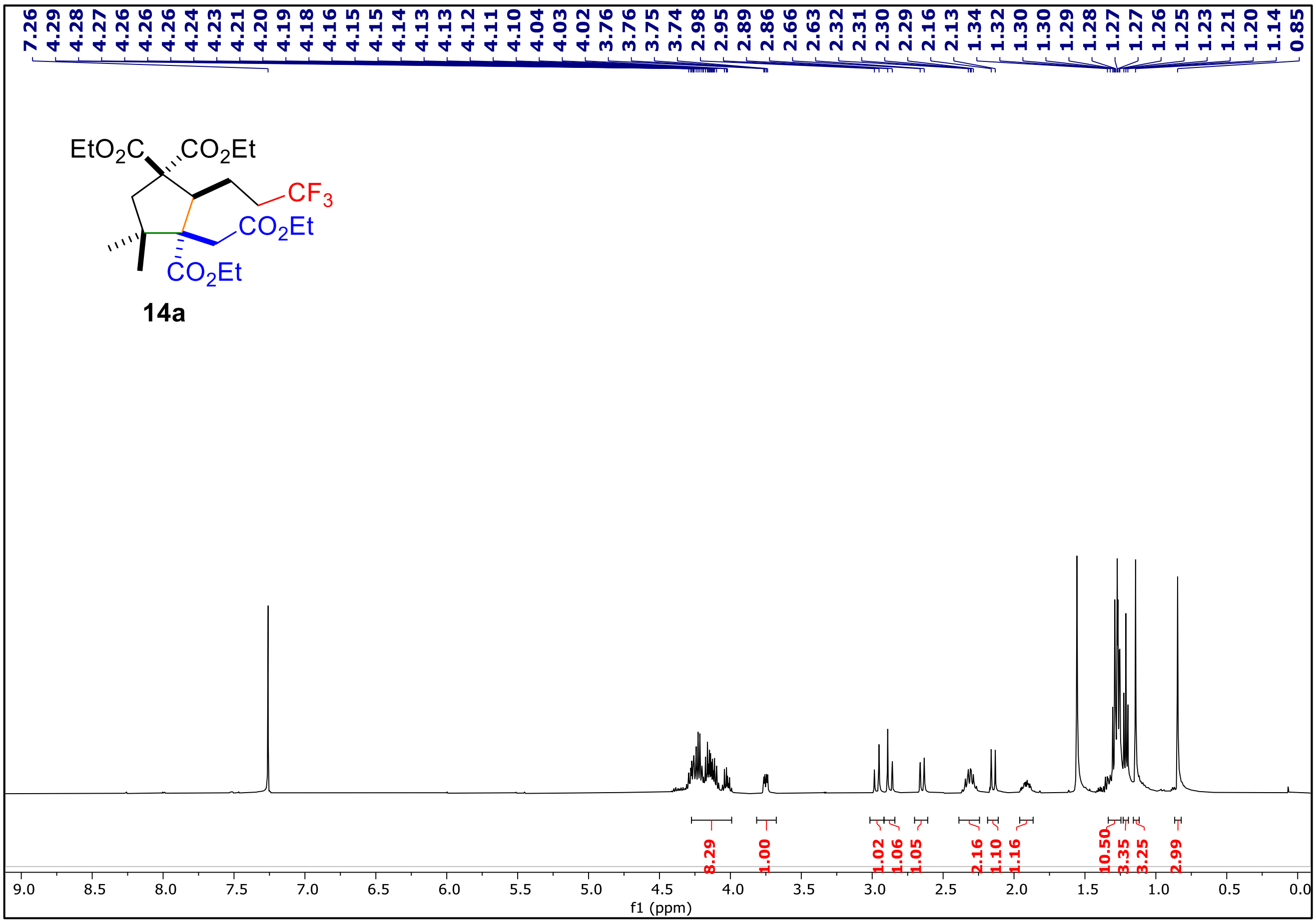

^{1}H NMR of compound **14a** (500 MHz, $CDCl_3$)

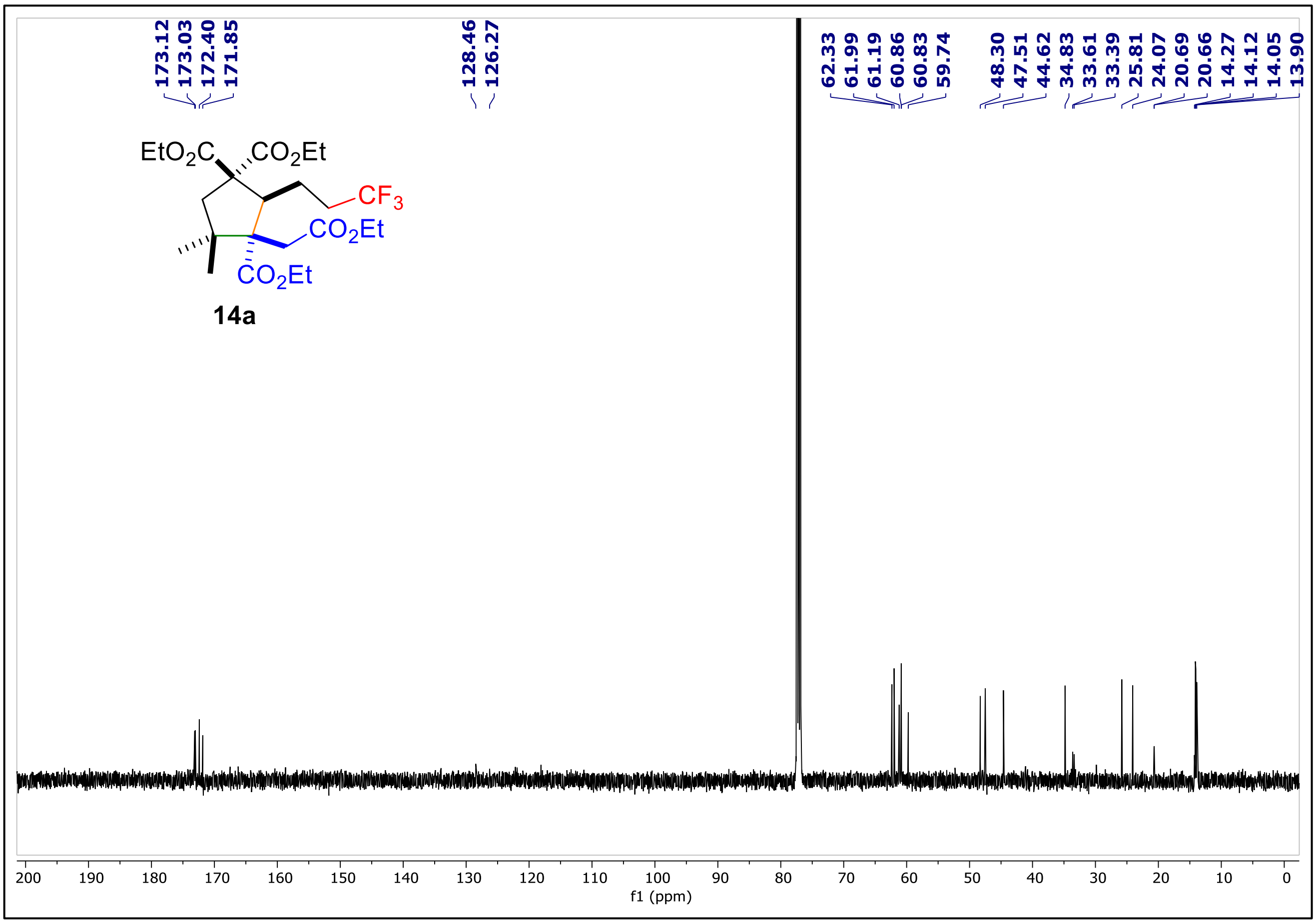

^{13}C{^{1}H} NMR of compound **14a** (126 MHz, $CDCl_3$)

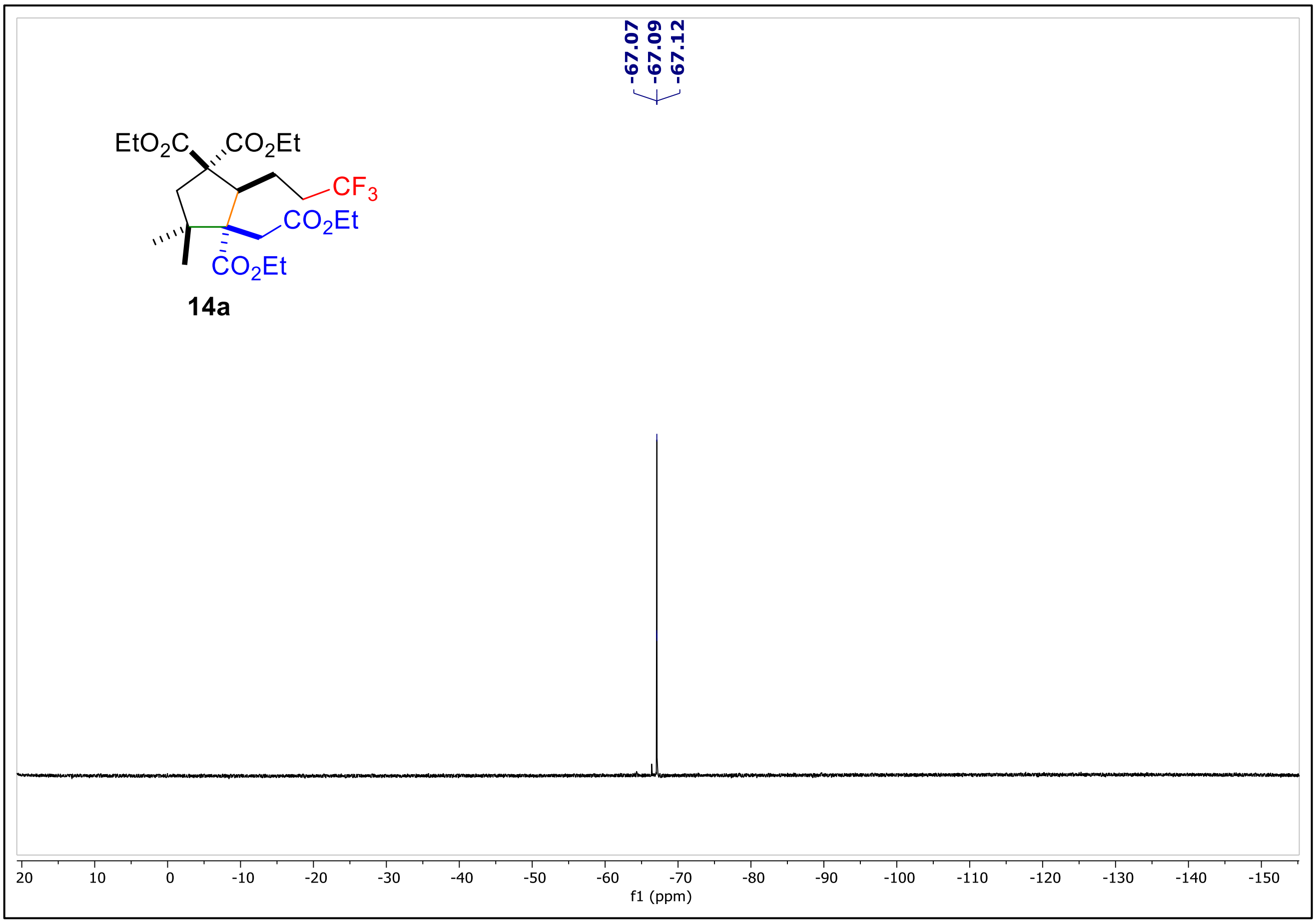

^{19}F NMR of compound **14a** (471 MHz, $CDCl_3$)

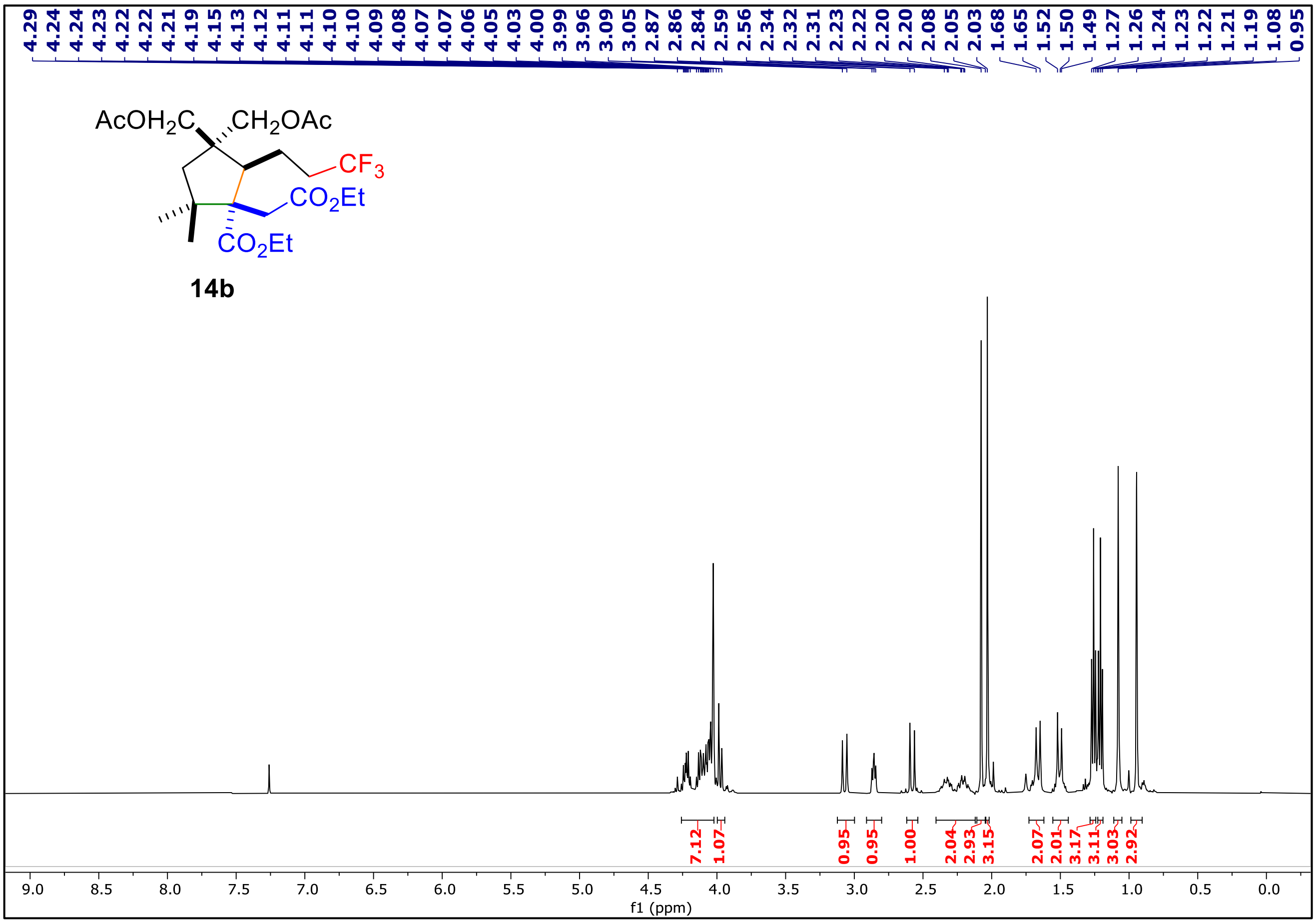

^{1}H NMR of compound **14b** (500 MHz, $CDCl_3$)

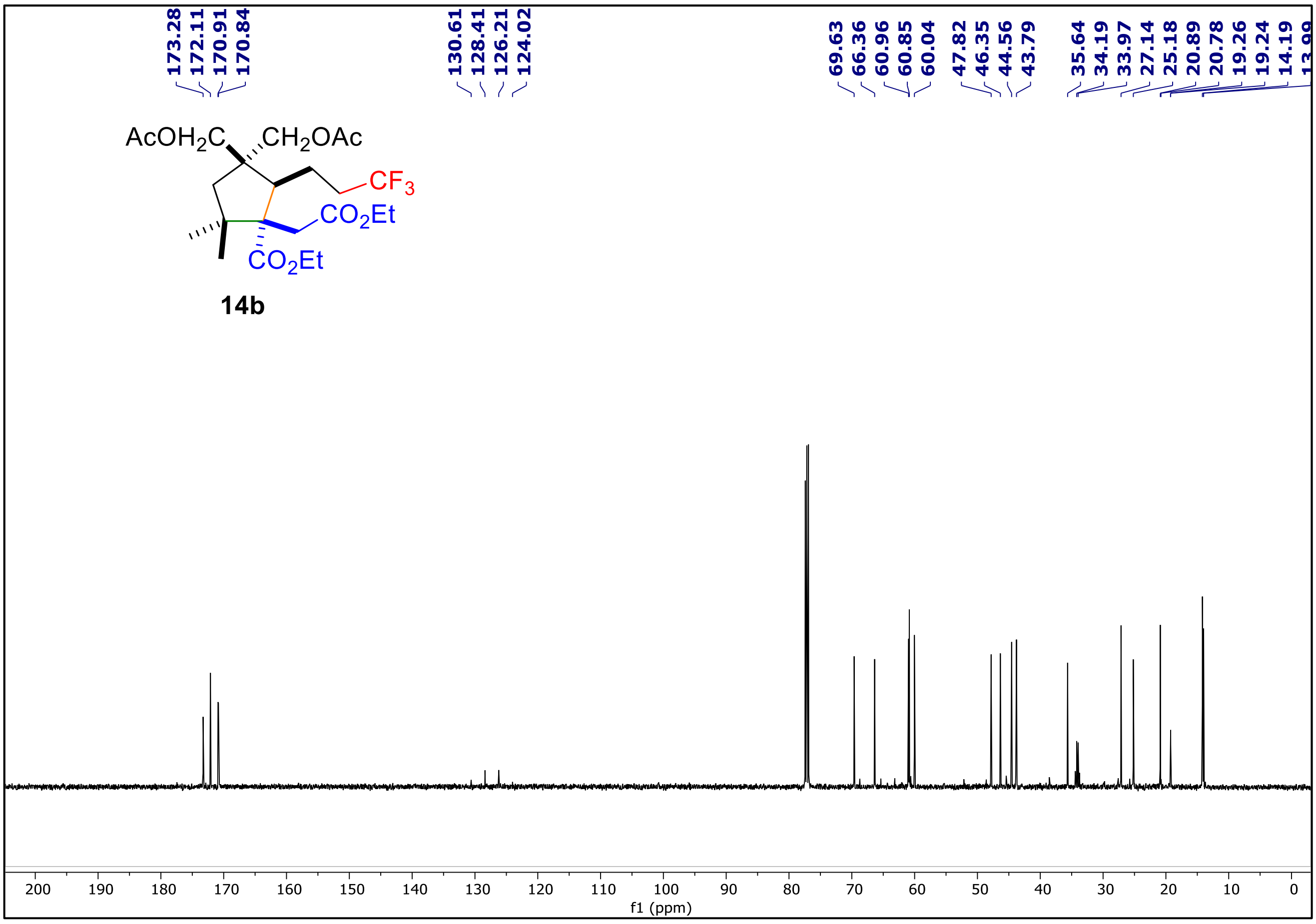

$^{13}C\{^1H\}$ NMR of compound **14b** (126 MHz, $CDCl_3$)

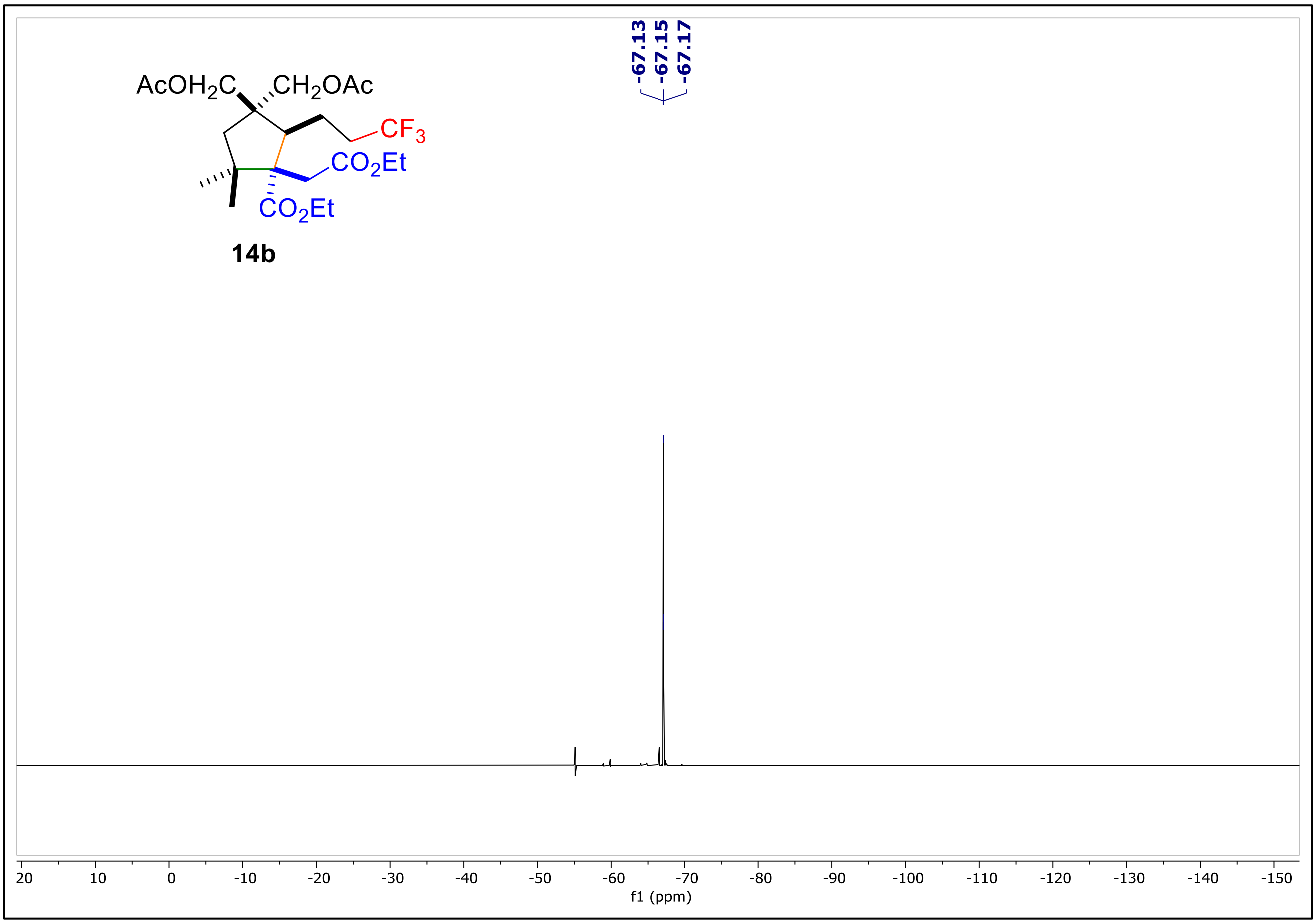

^{19}F NMR of compound **14b** (471 MHz, $CDCl_3$)

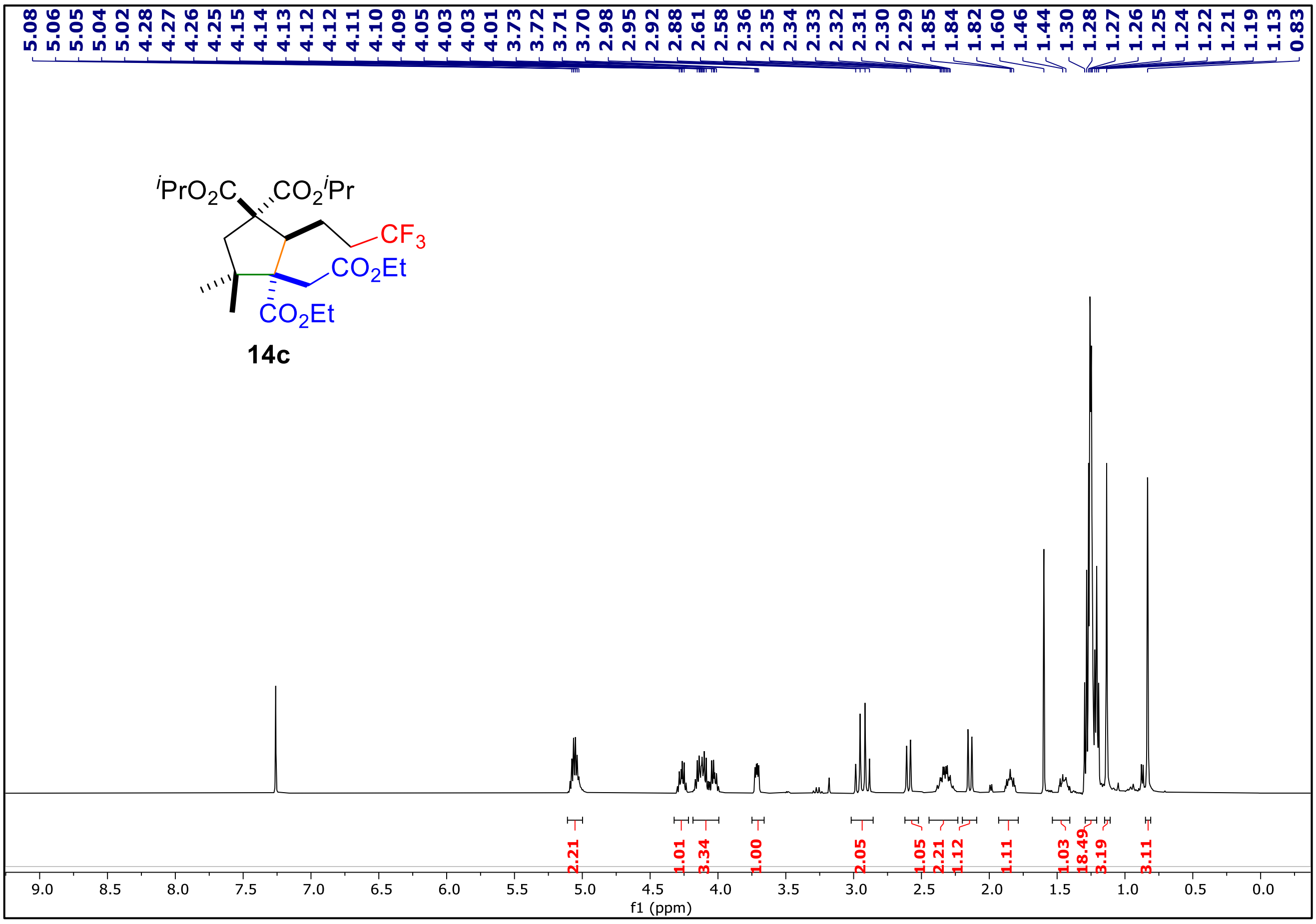

^{1}H NMR of compound **14c** (500 MHz, $CDCl_3$)

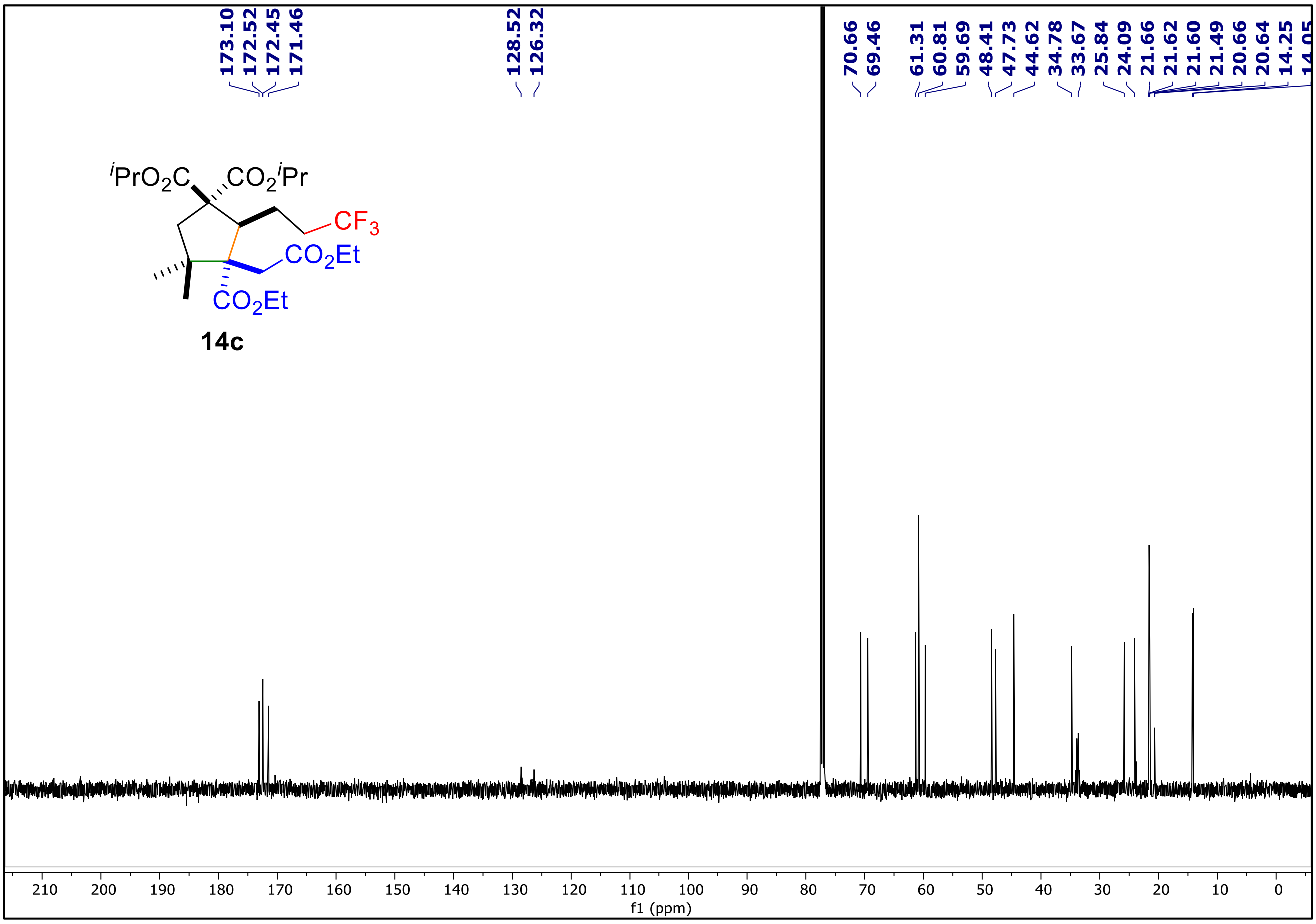

$^{13}C\{^{1}H\}$ NMR of compound **14c** (126 MHz, $CDCl_3$)

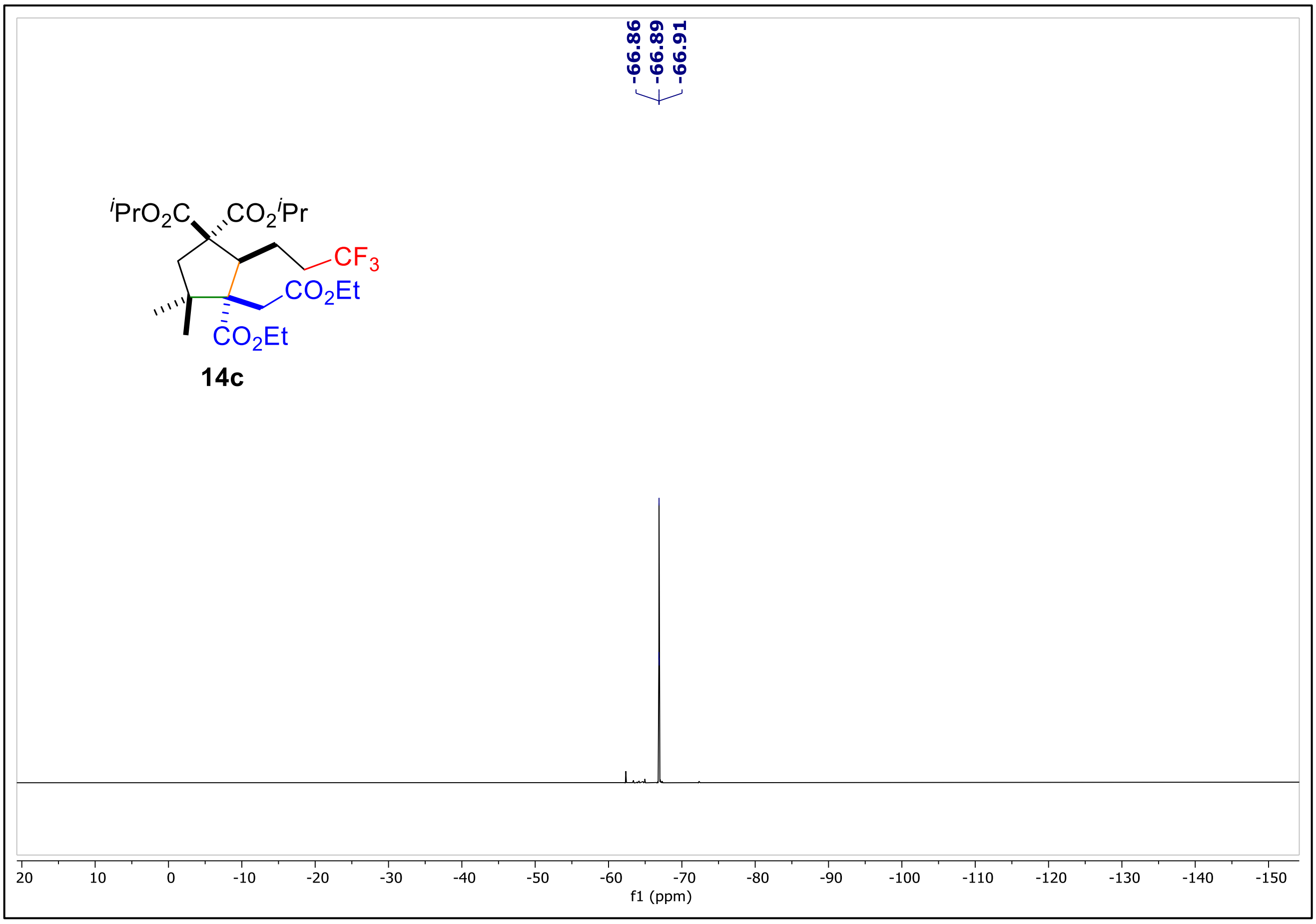

^{19}F NMR of compound **14c** (471 MHz, $CDCl_3$)

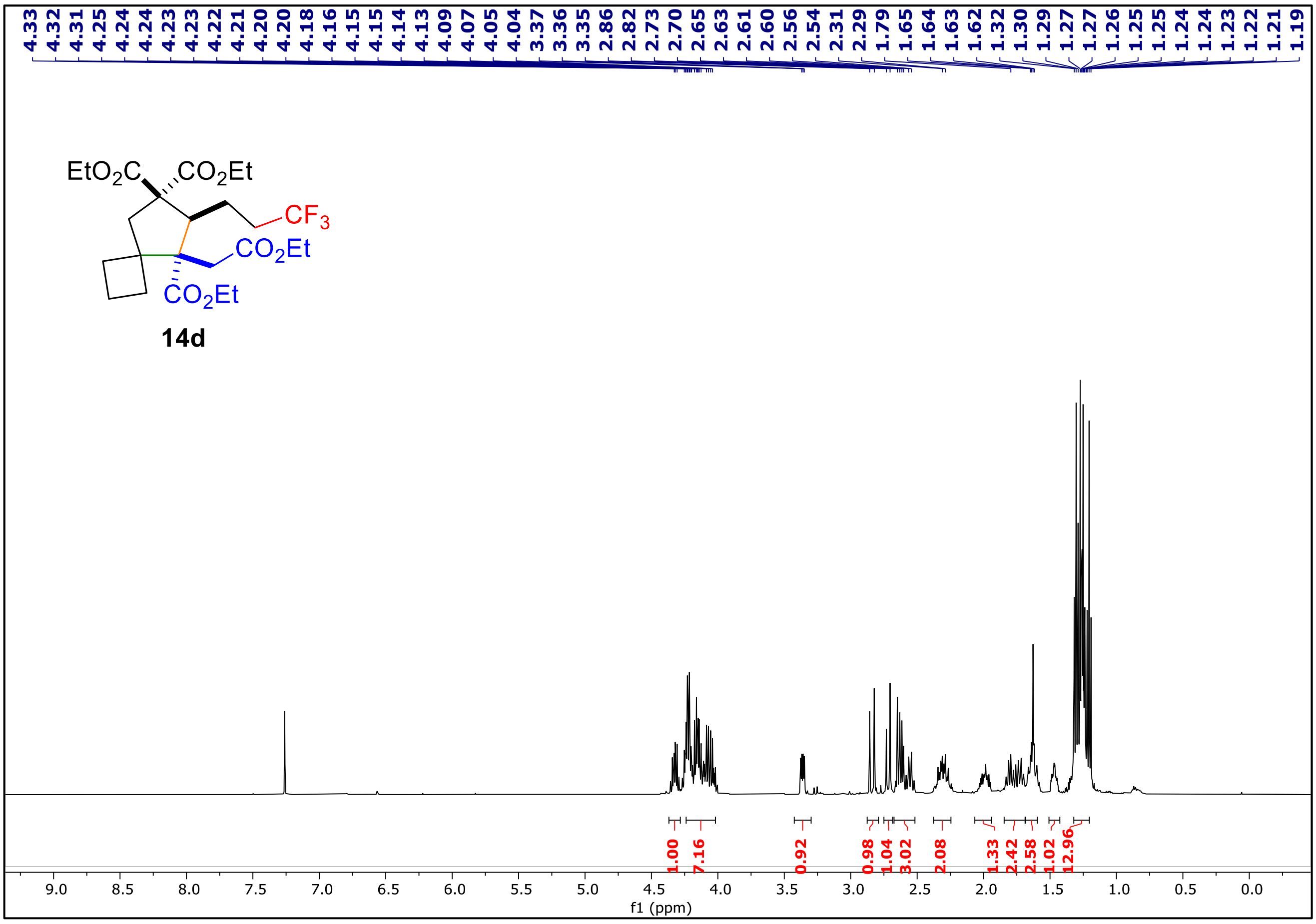

^1H NMR of compound **14d** (500 MHz, $CDCl_3$)

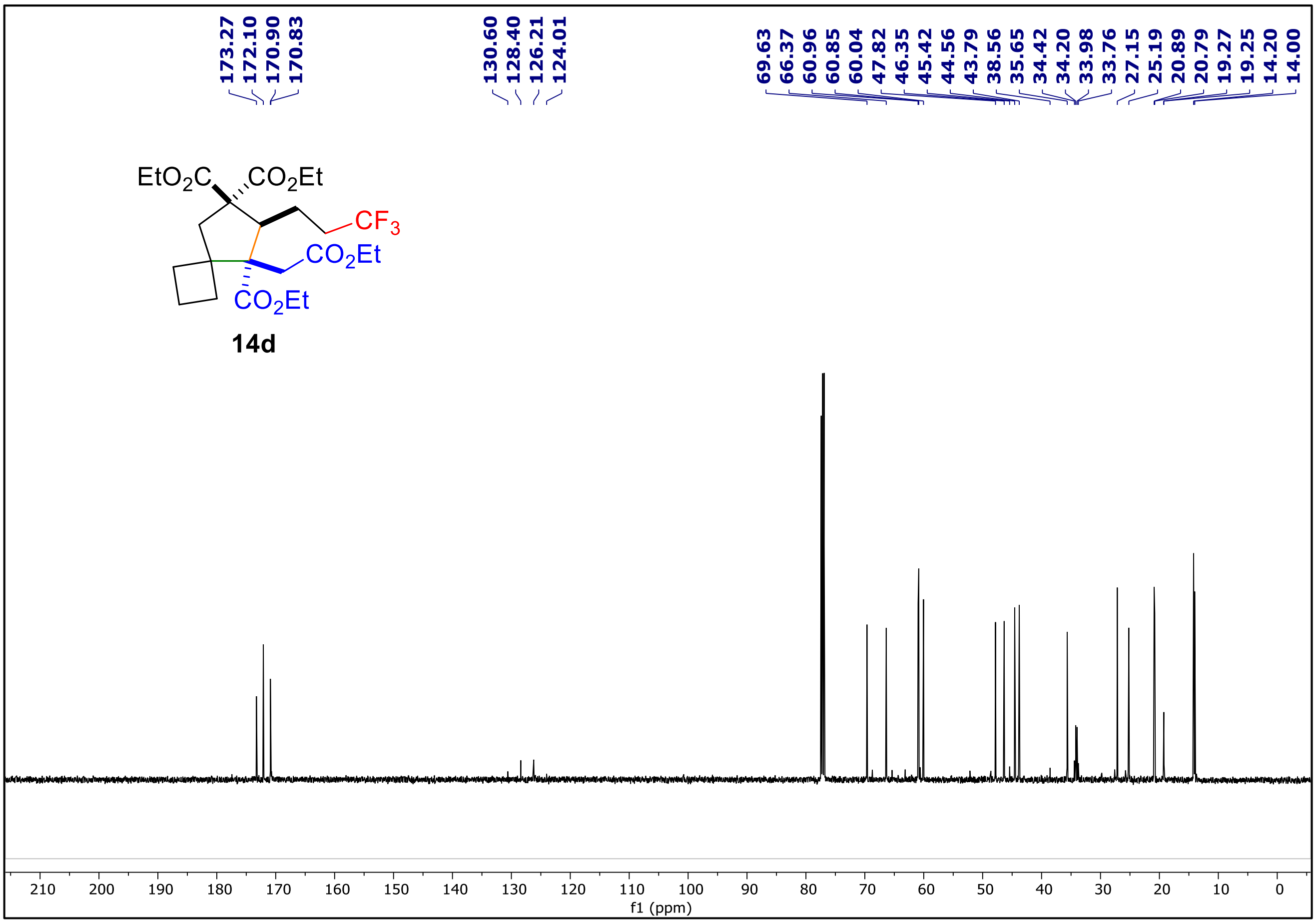

$^{13}C\{^{1}H\}$ NMR of compound **14d** (126 MHz, $CDCl_3$)

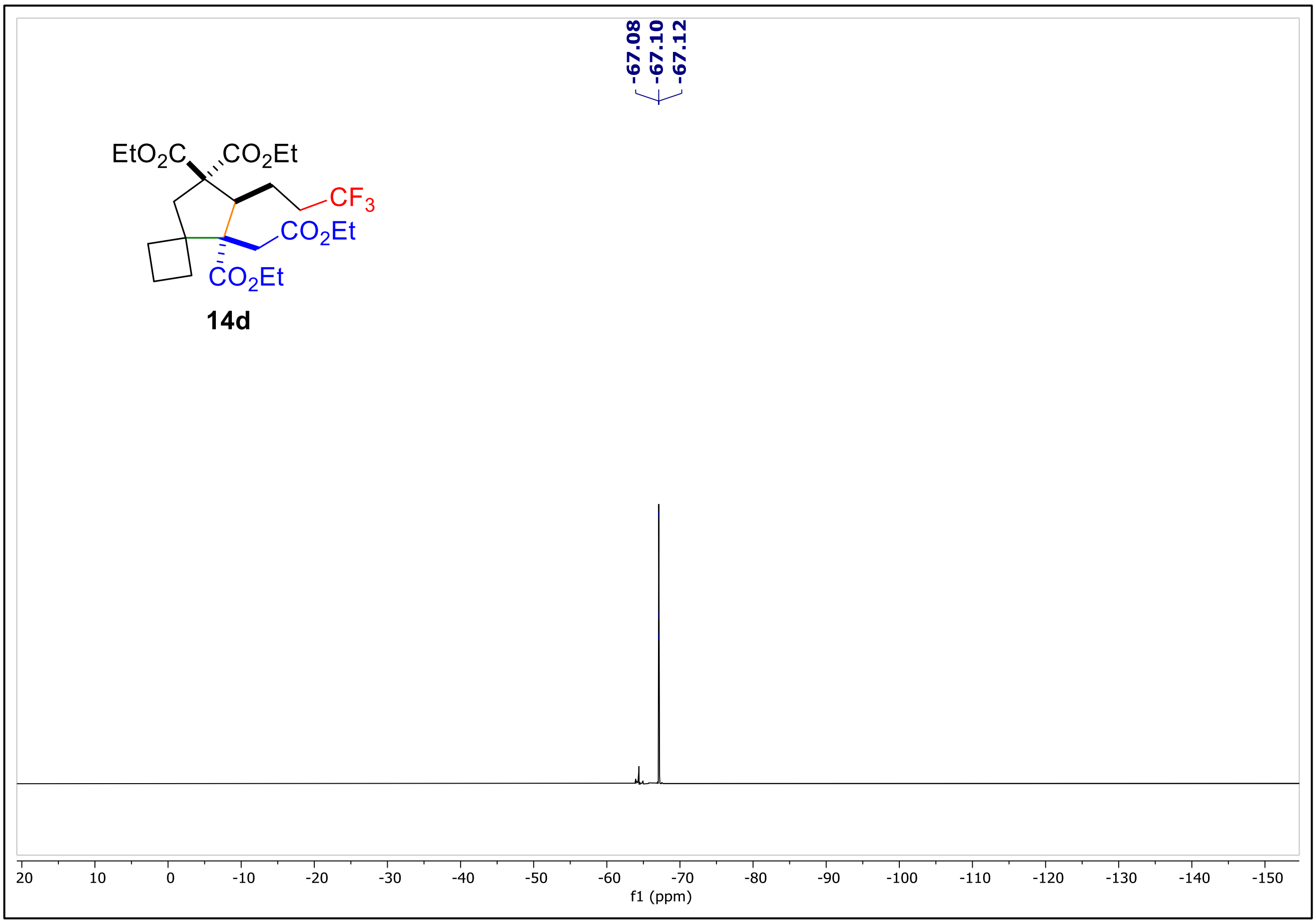

^{19}F NMR of compound **14d** (471 MHz, $CDCl_3$)

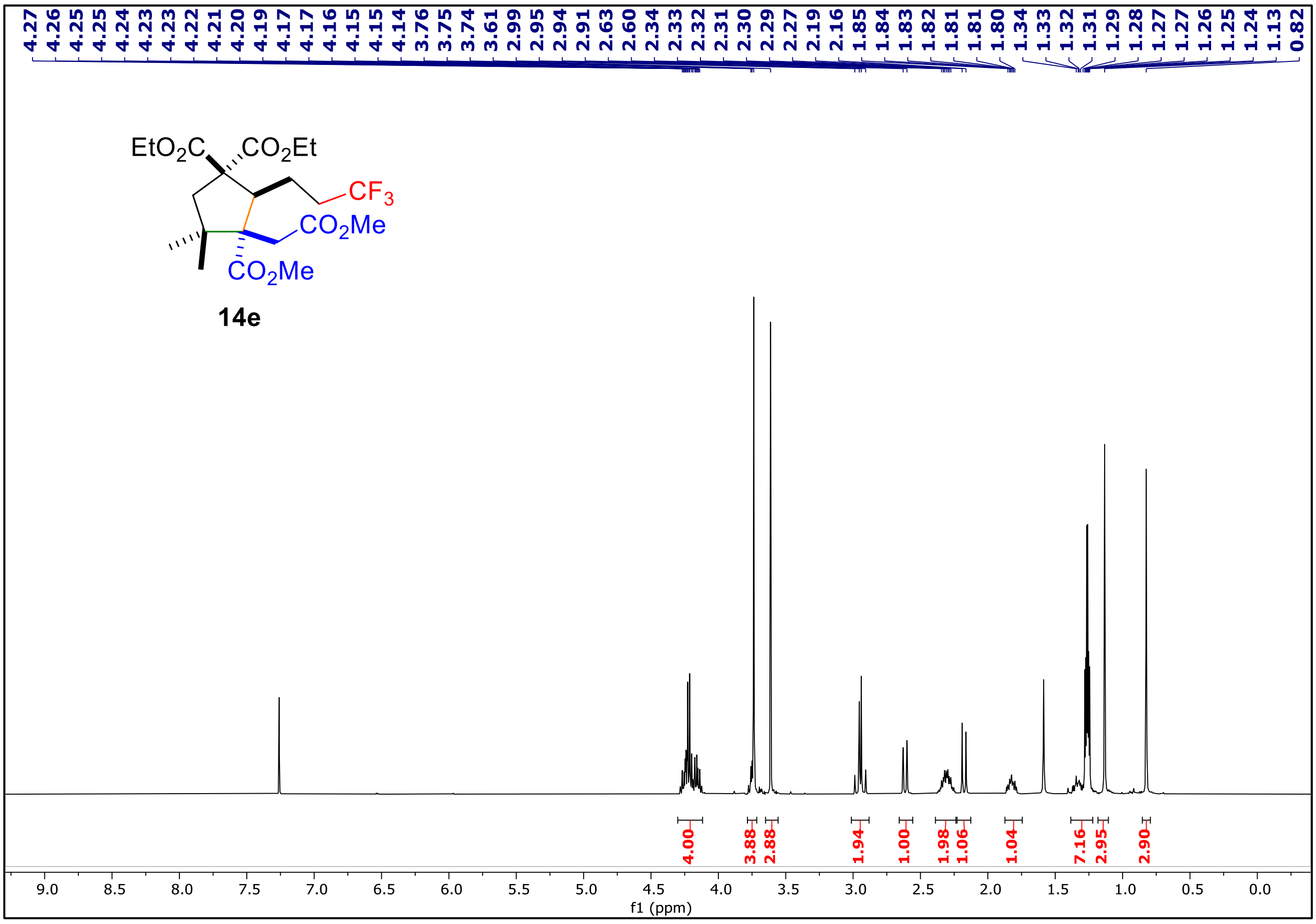

^{1}H NMR of compound **14e** (500 MHz, $CDCl_3$)

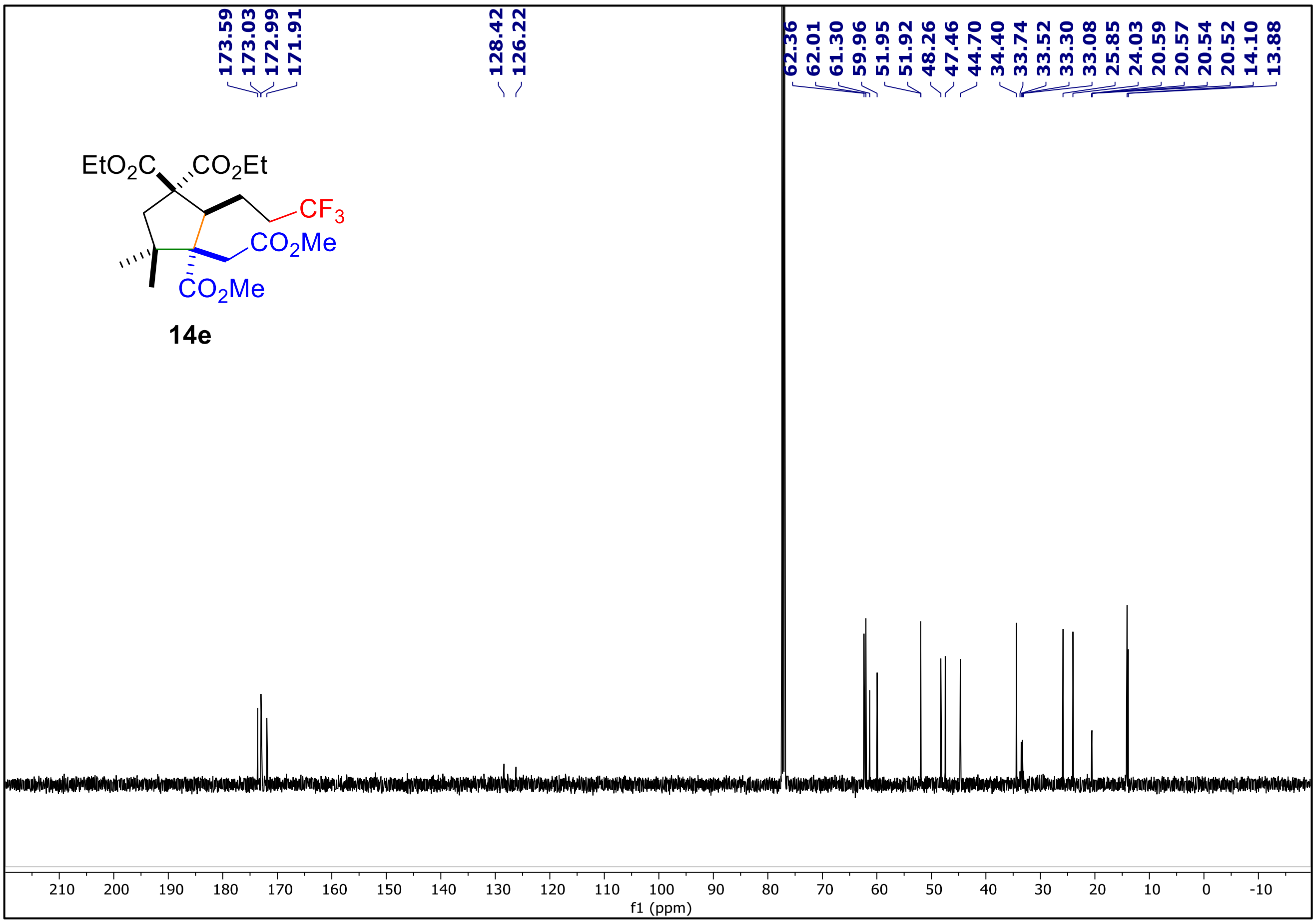

$^{13}C\{^1H\}$ NMR of compound **14e** (126 MHz, $CDCl_3$)

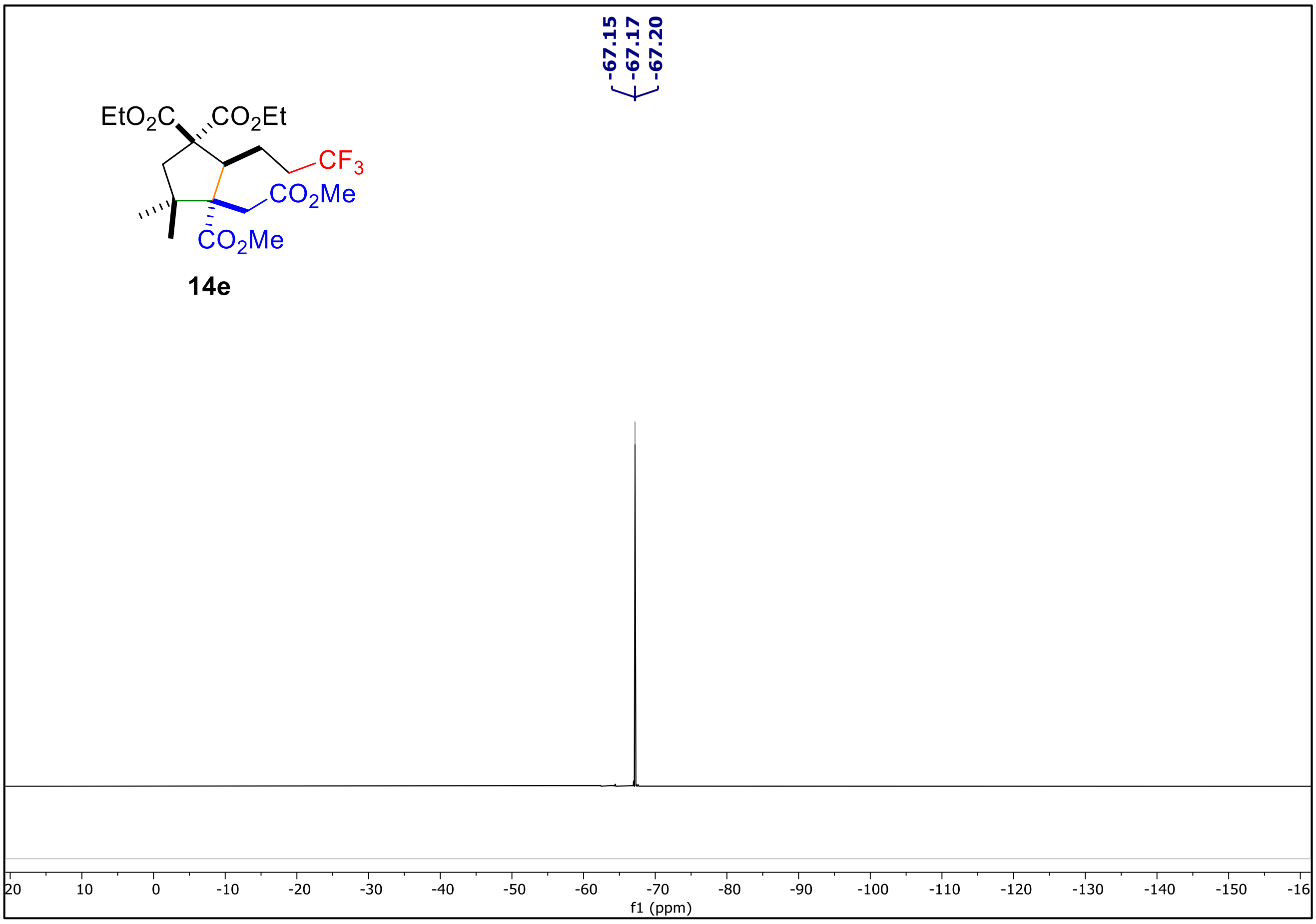

^{19}F NMR of compound **14e** (471 MHz, $CDCl_3$)

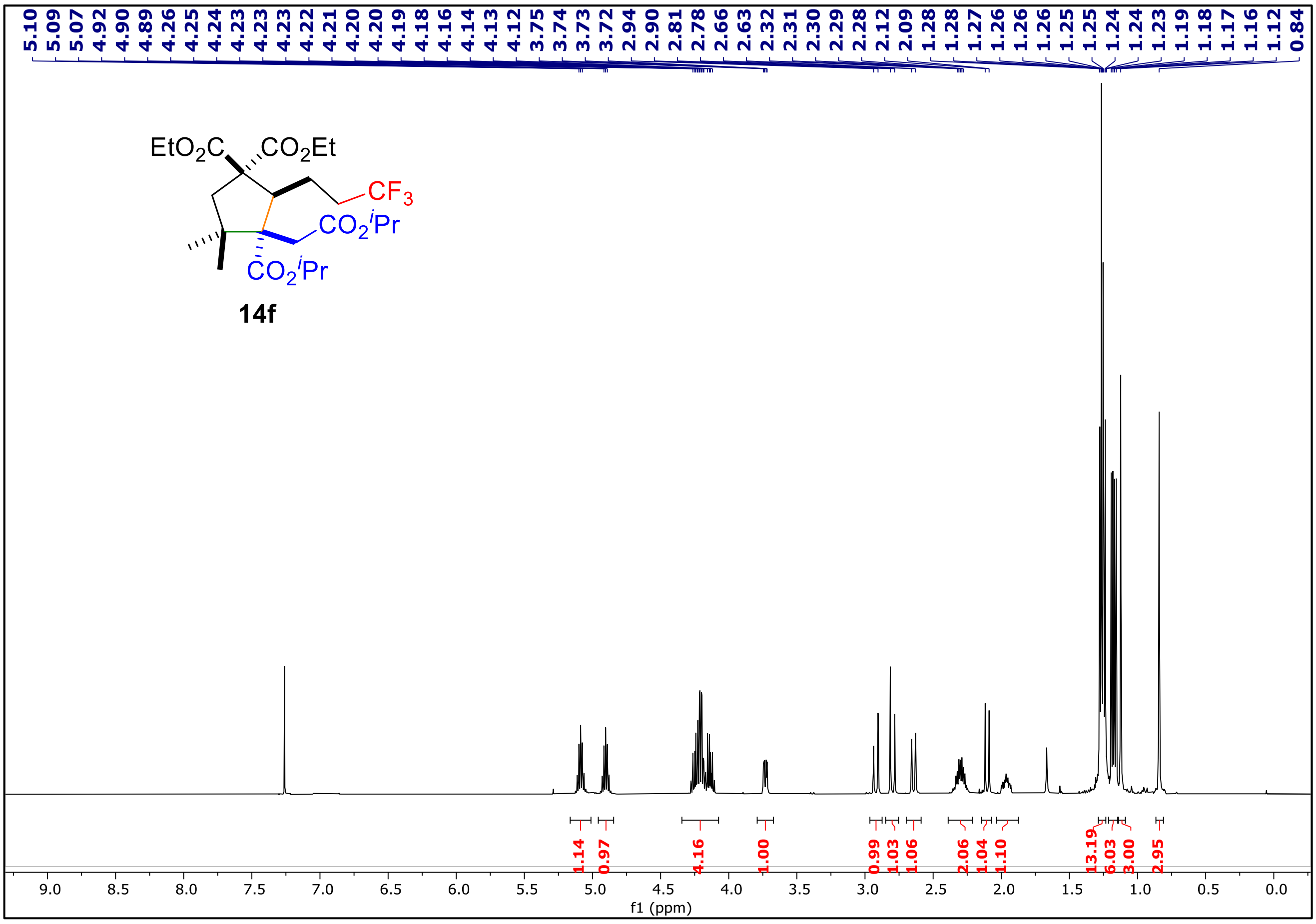

^{1}H NMR of compound **14f** (500 MHz, $CDCl_3$)

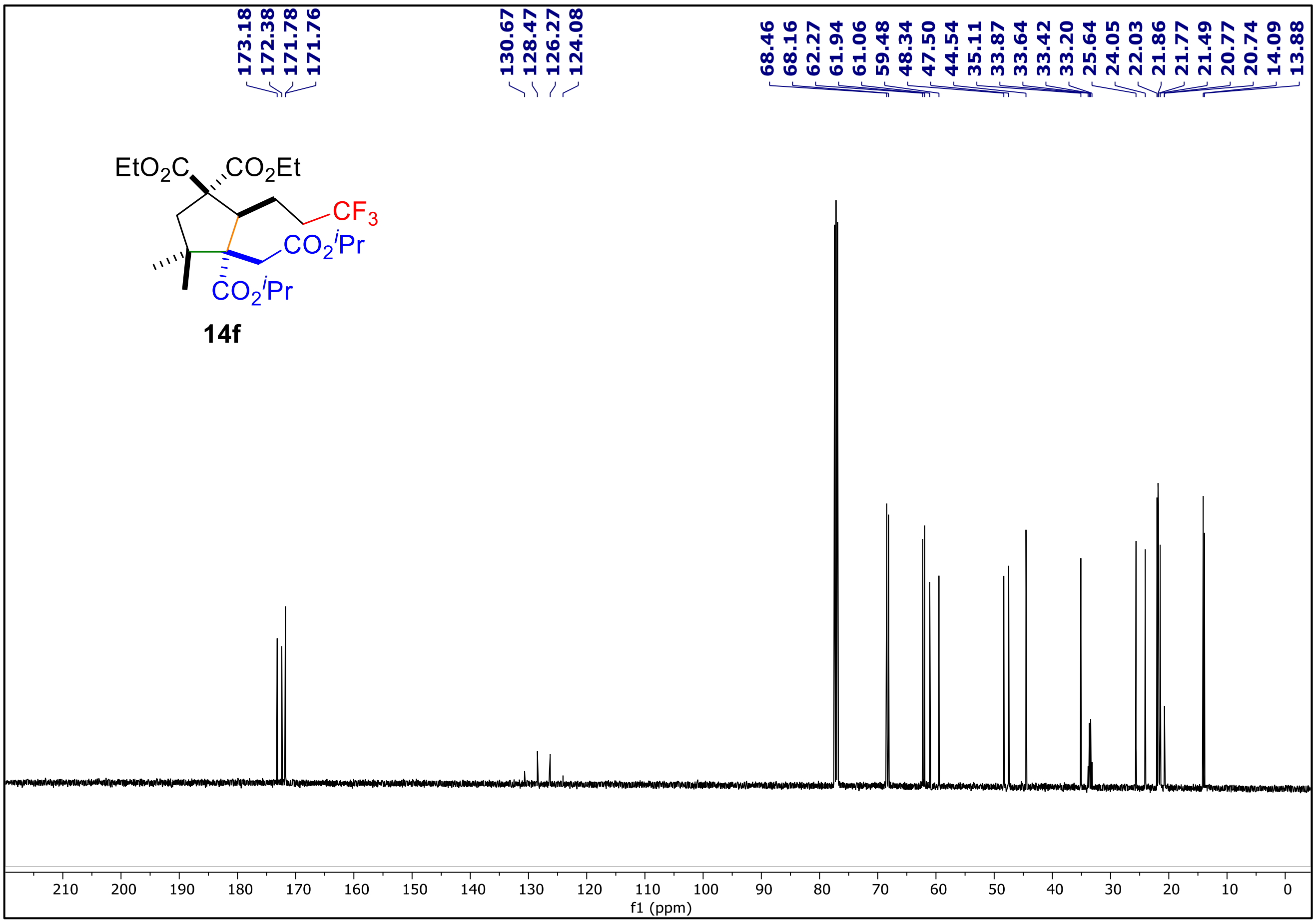

$^{13}C\{^{1}H\}$ NMR of compound **14f** (126 MHz, $CDCl_3$)

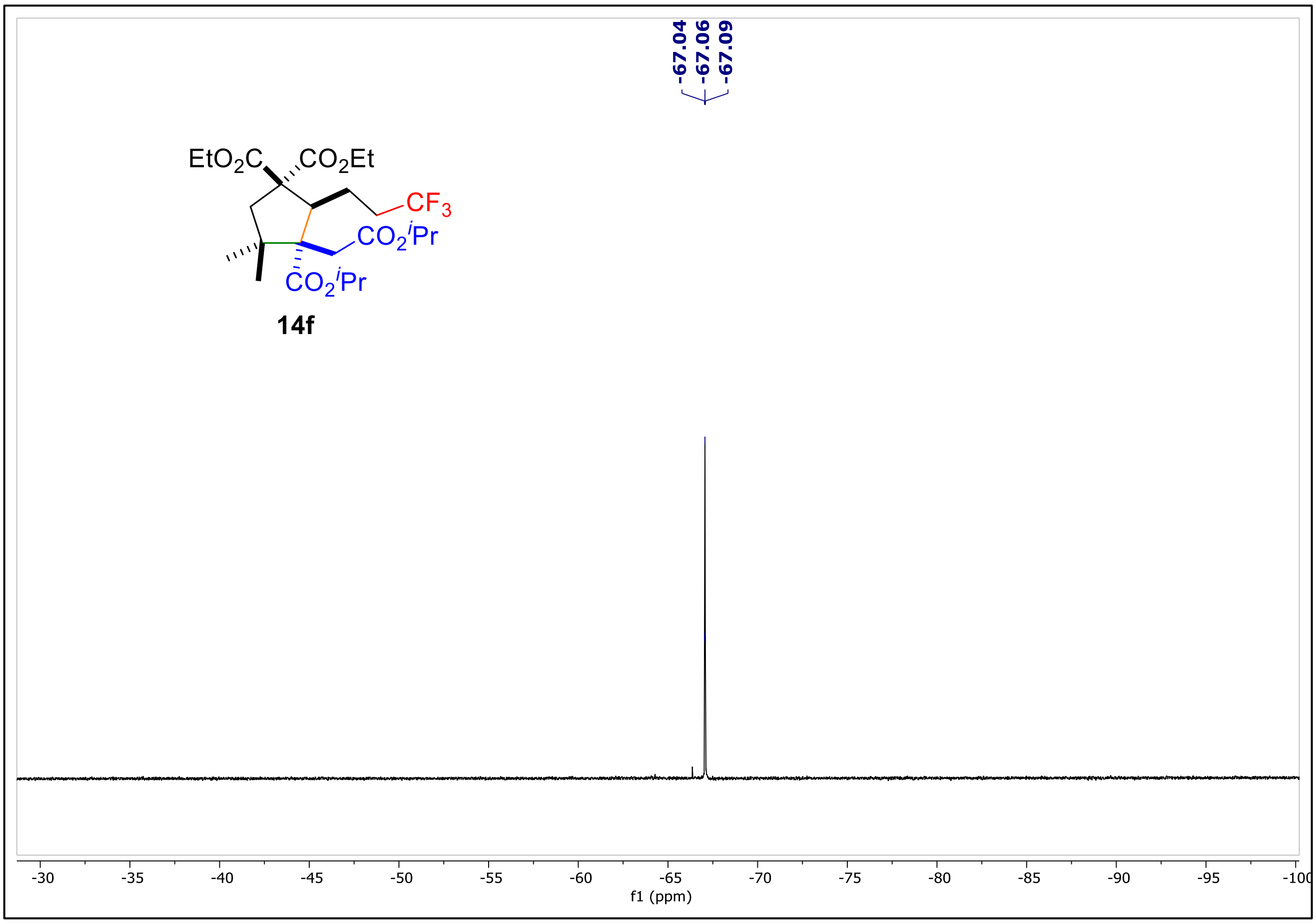

^{19}F NMR of compound **14f** (471 MHz, $CDCl_3$)

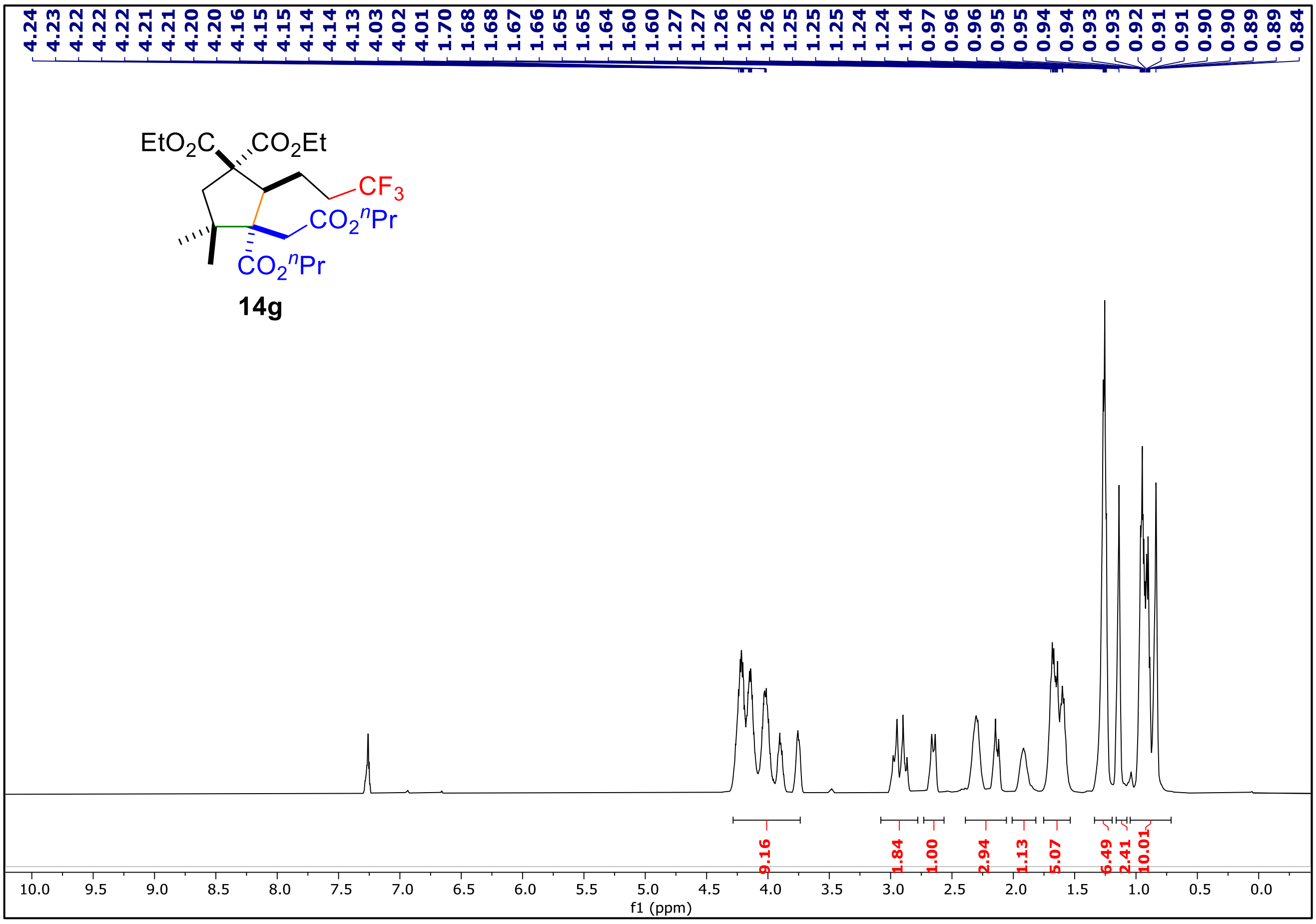

¹H NMR of compound **14g** (500 MHz, CDCl₃)

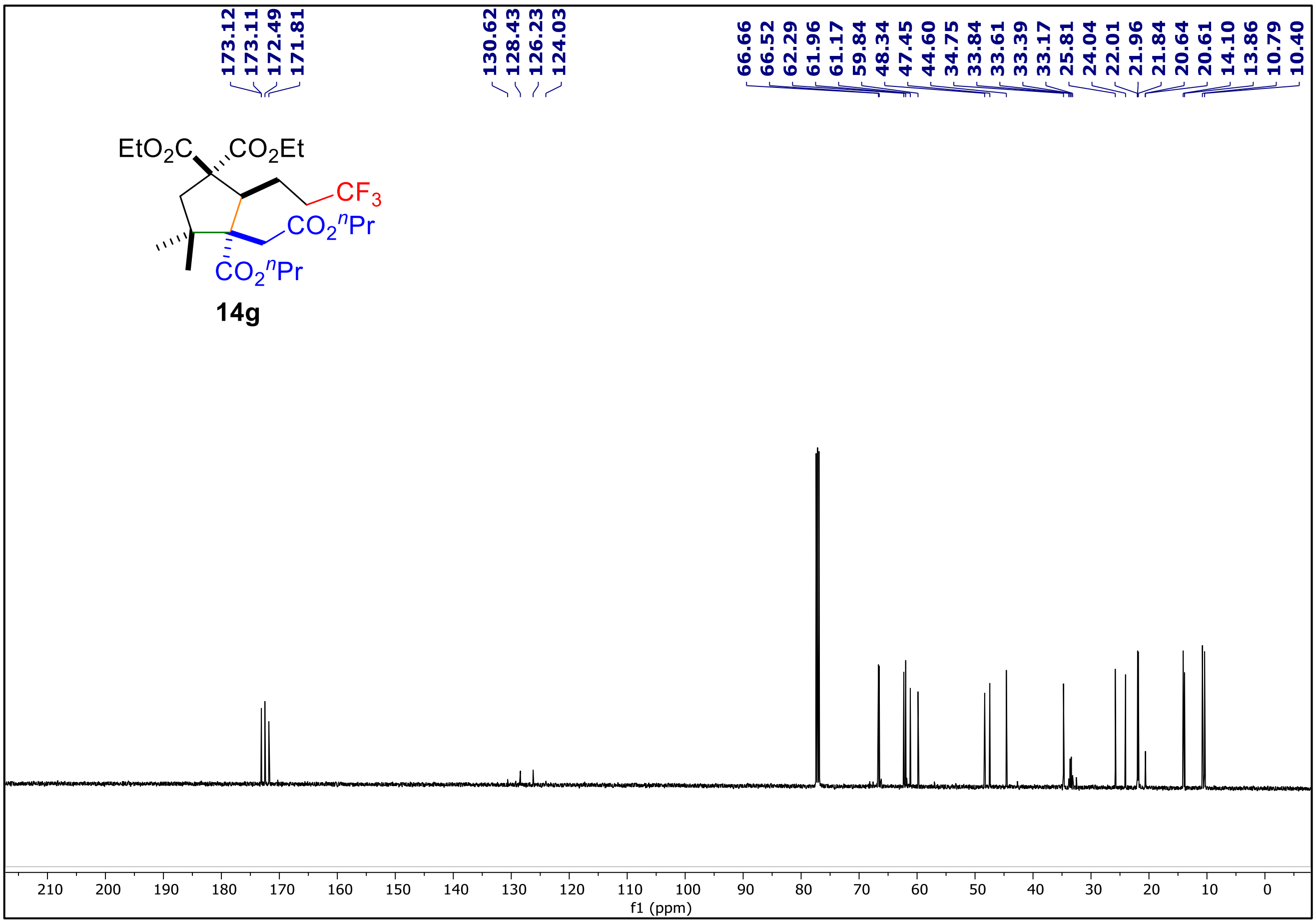

¹³C{¹H} NMR of compound **14g** (126 MHz, CDCl₃)

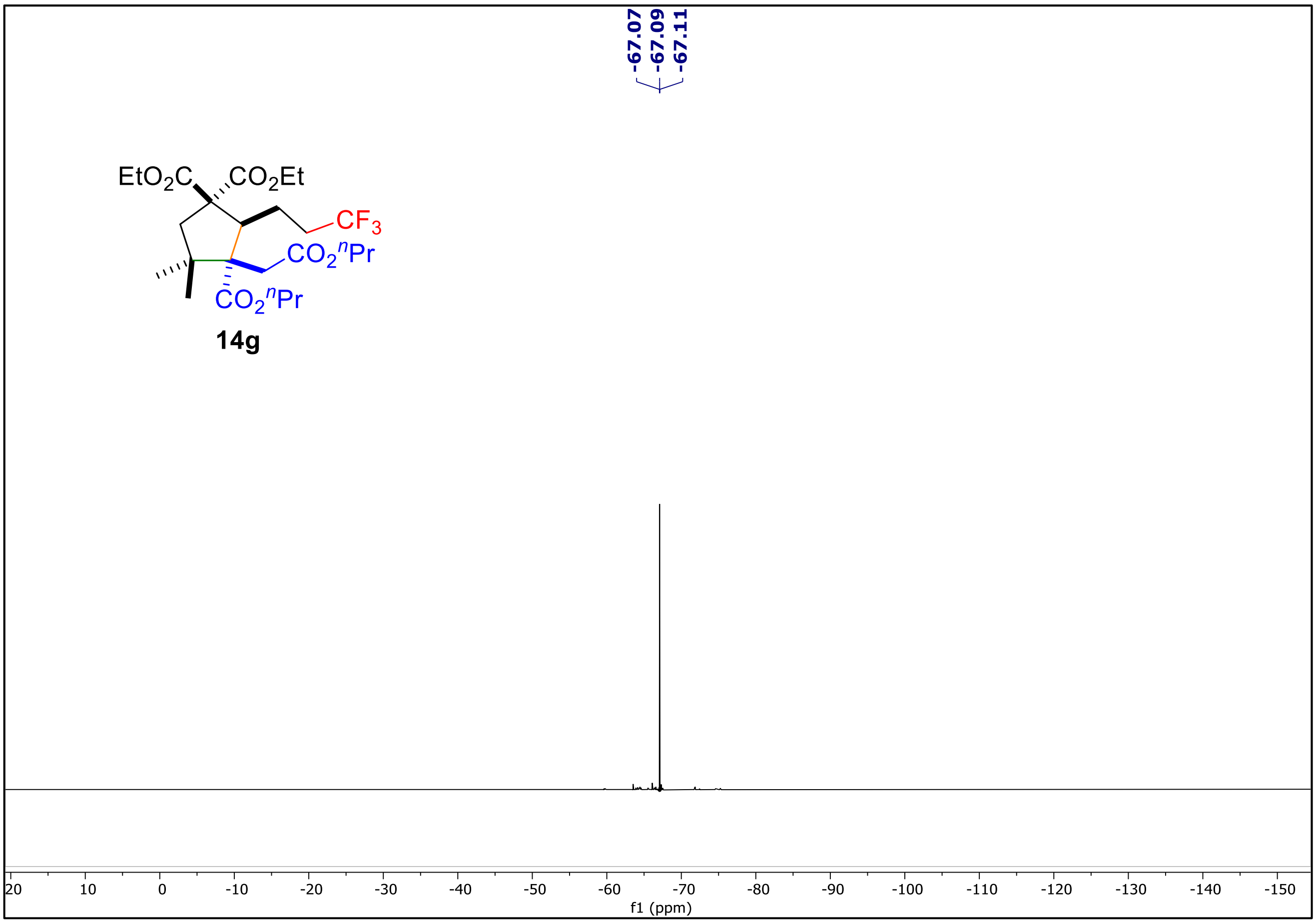

^{19}F NMR of compound **14g** (471 MHz, $CDCl_3$)

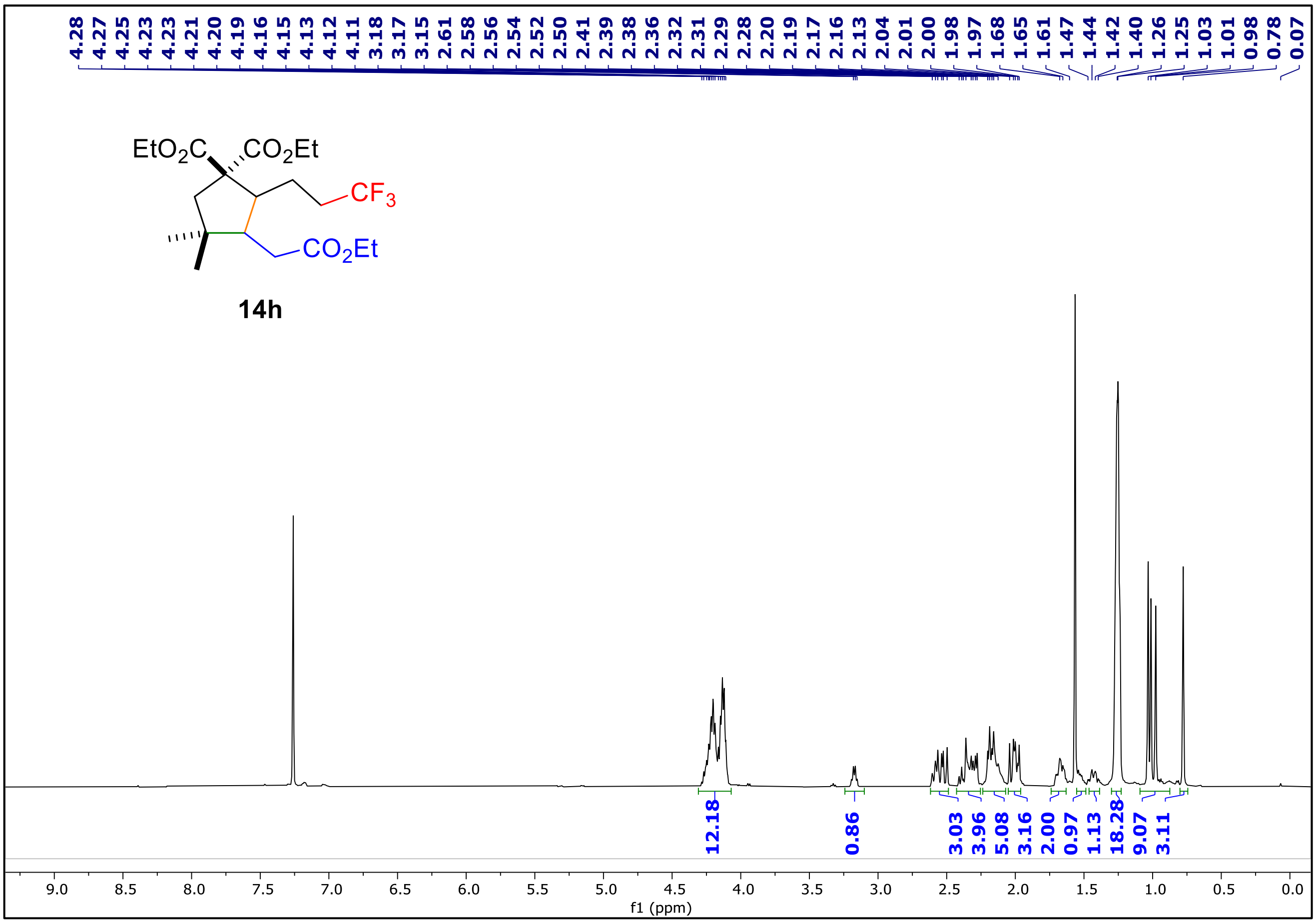

^{1}H NMR of compound **14h** (500 MHz, $CDCl_3$)

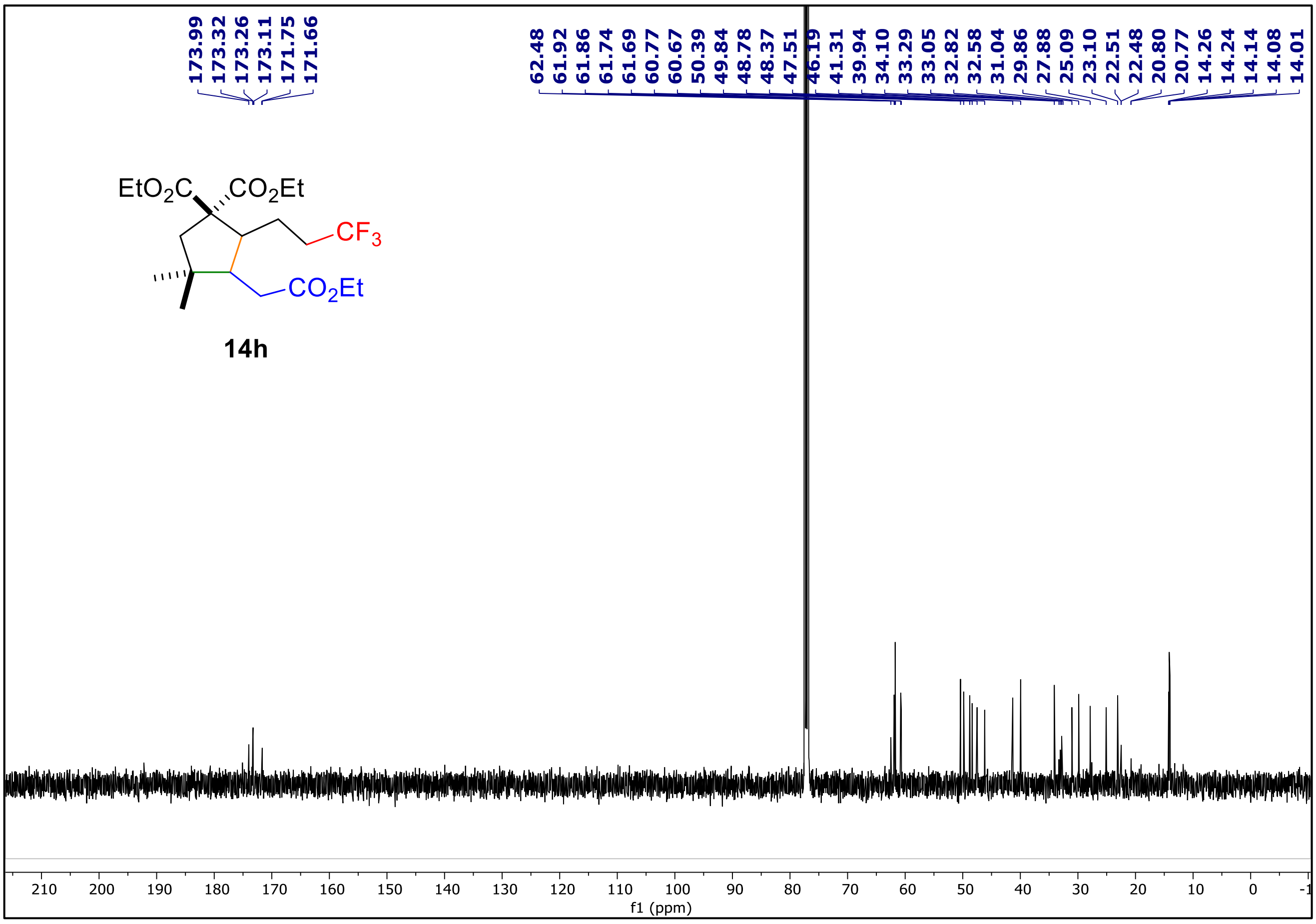

$^{13}C\{^{1}H\}$ NMR of compound **14h** (126 MHz, $CDCl_3$)

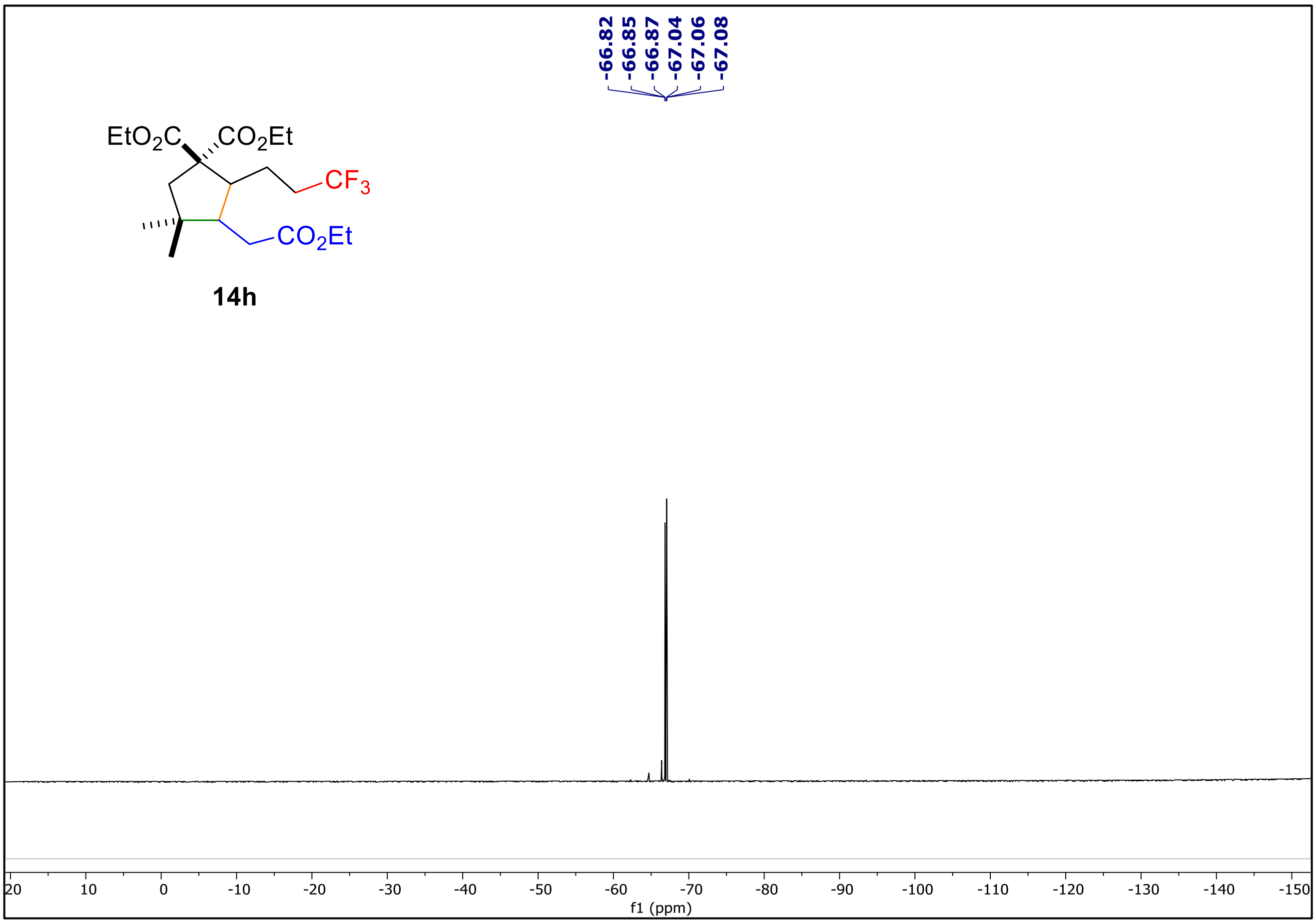

^{19}F NMR of compound **14h** (471 MHz, $CDCl_3$)

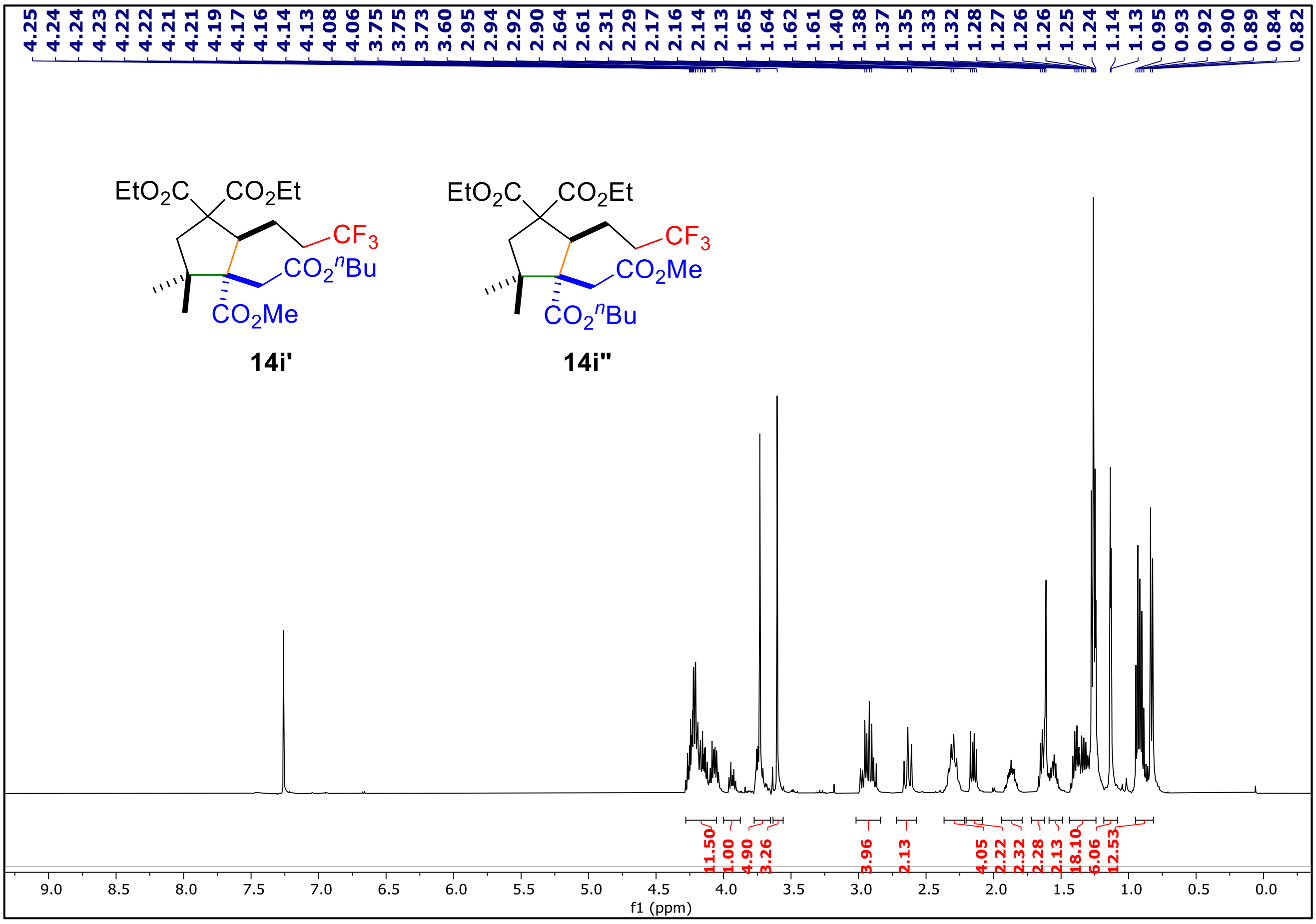

^{1}H NMR of compound **14i** (500 MHz, $CDCl_3$)

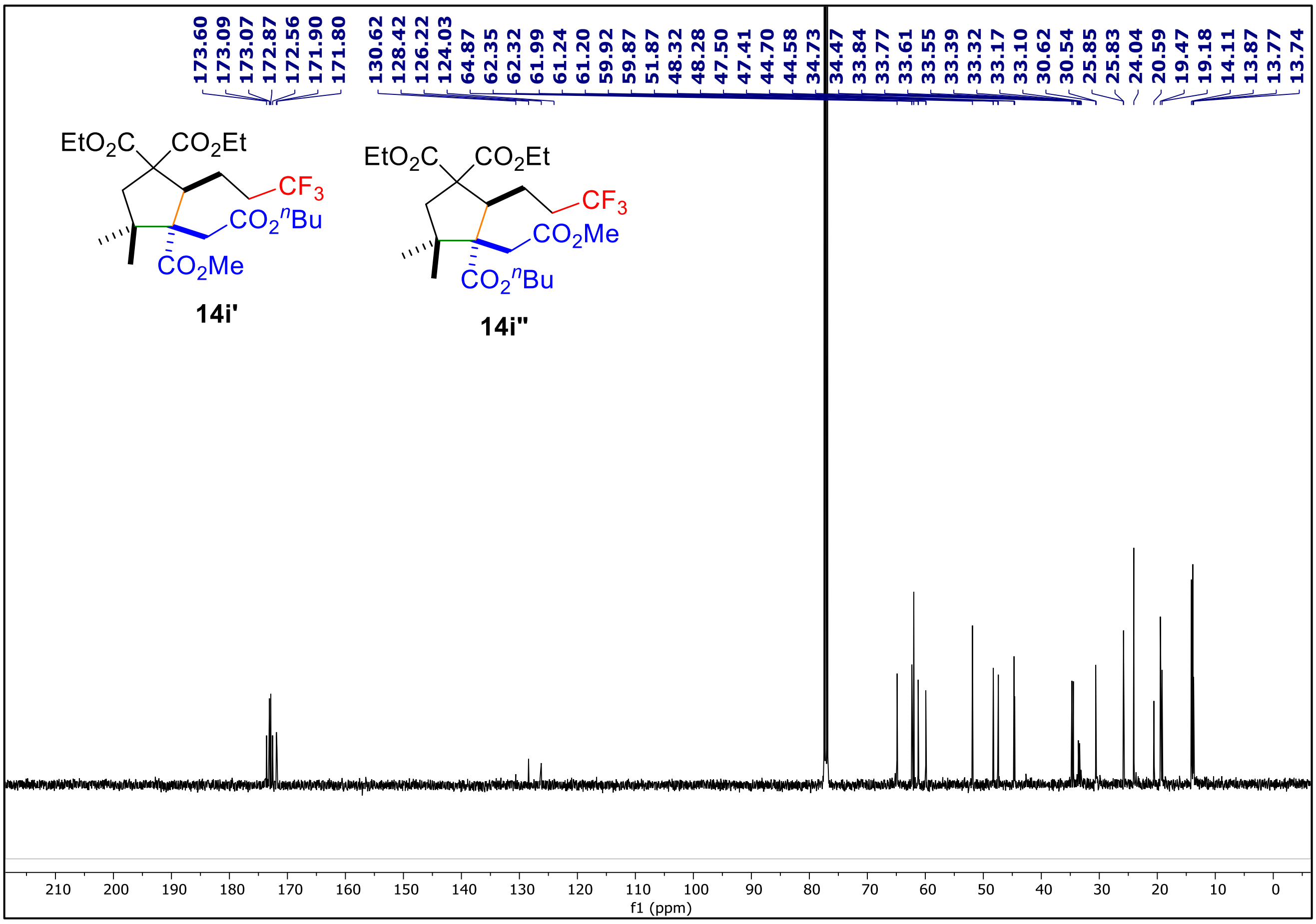

$^{13}C\{^1H\}$ NMR of compound **14i** (126 MHz, $CDCl_3$)

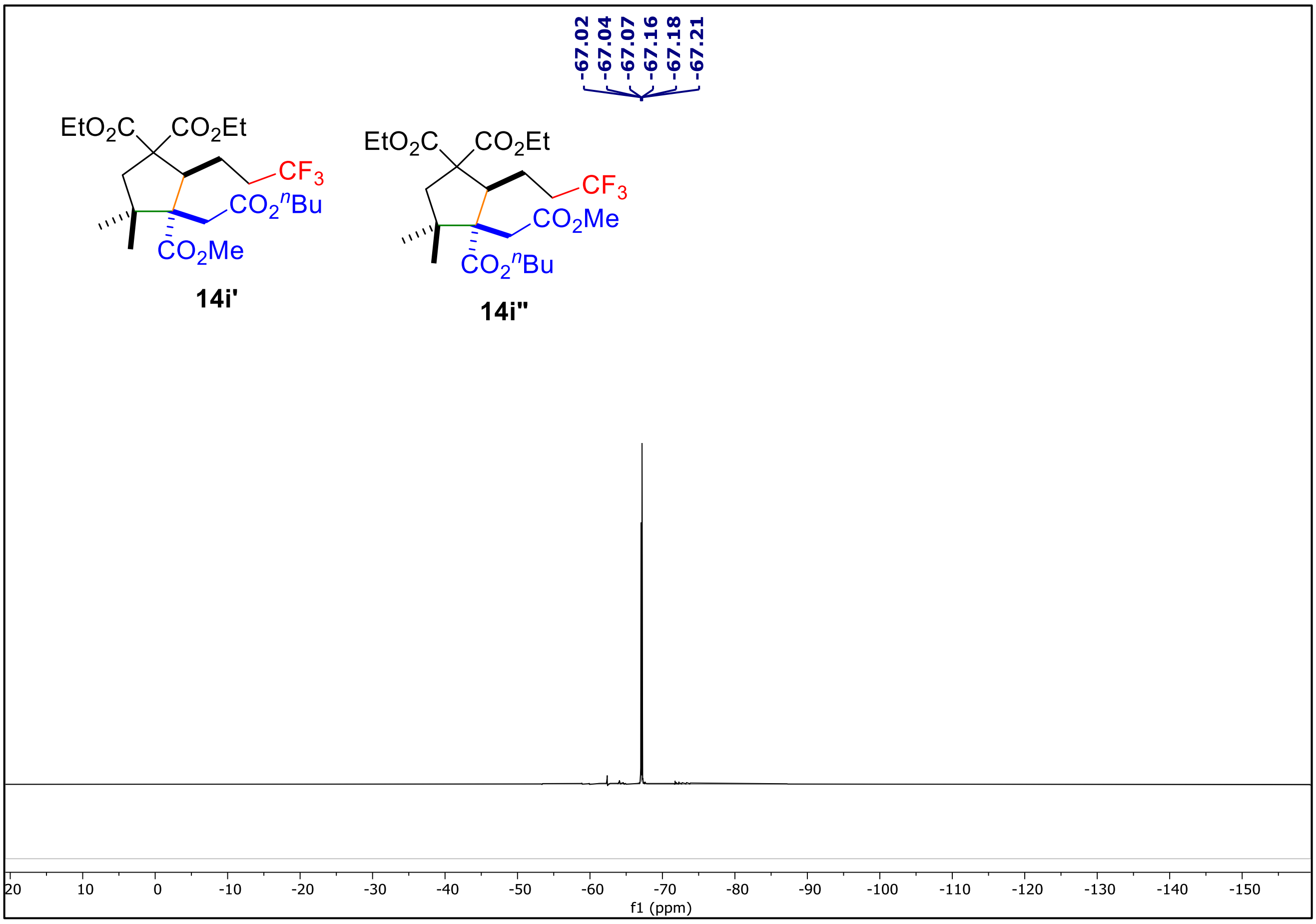

^{19}F NMR of compound **14i** (471 MHz, $CDCl_3$)

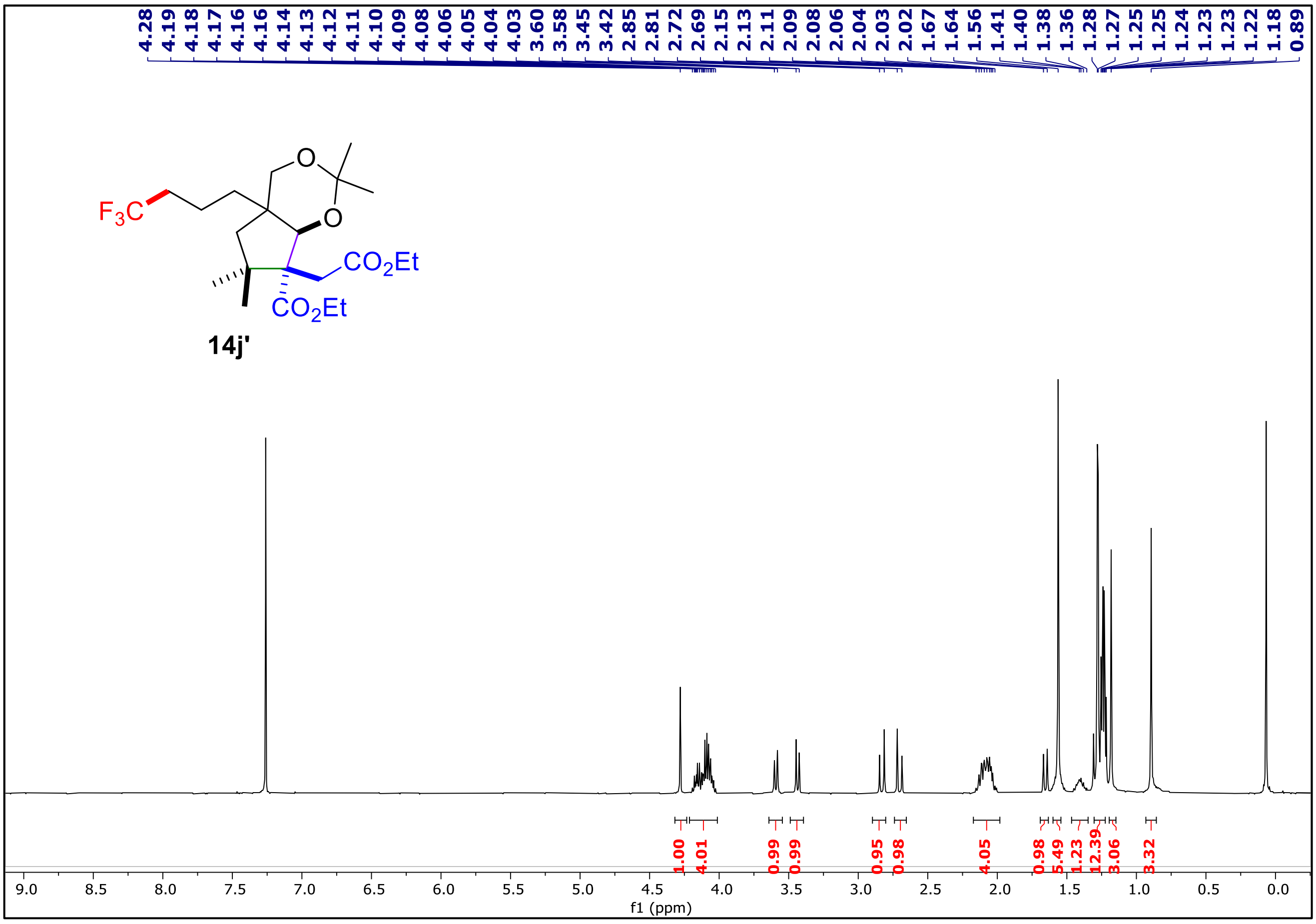

^{1}H NMR of compound **14j’** (500 MHz, $CDCl_3$)

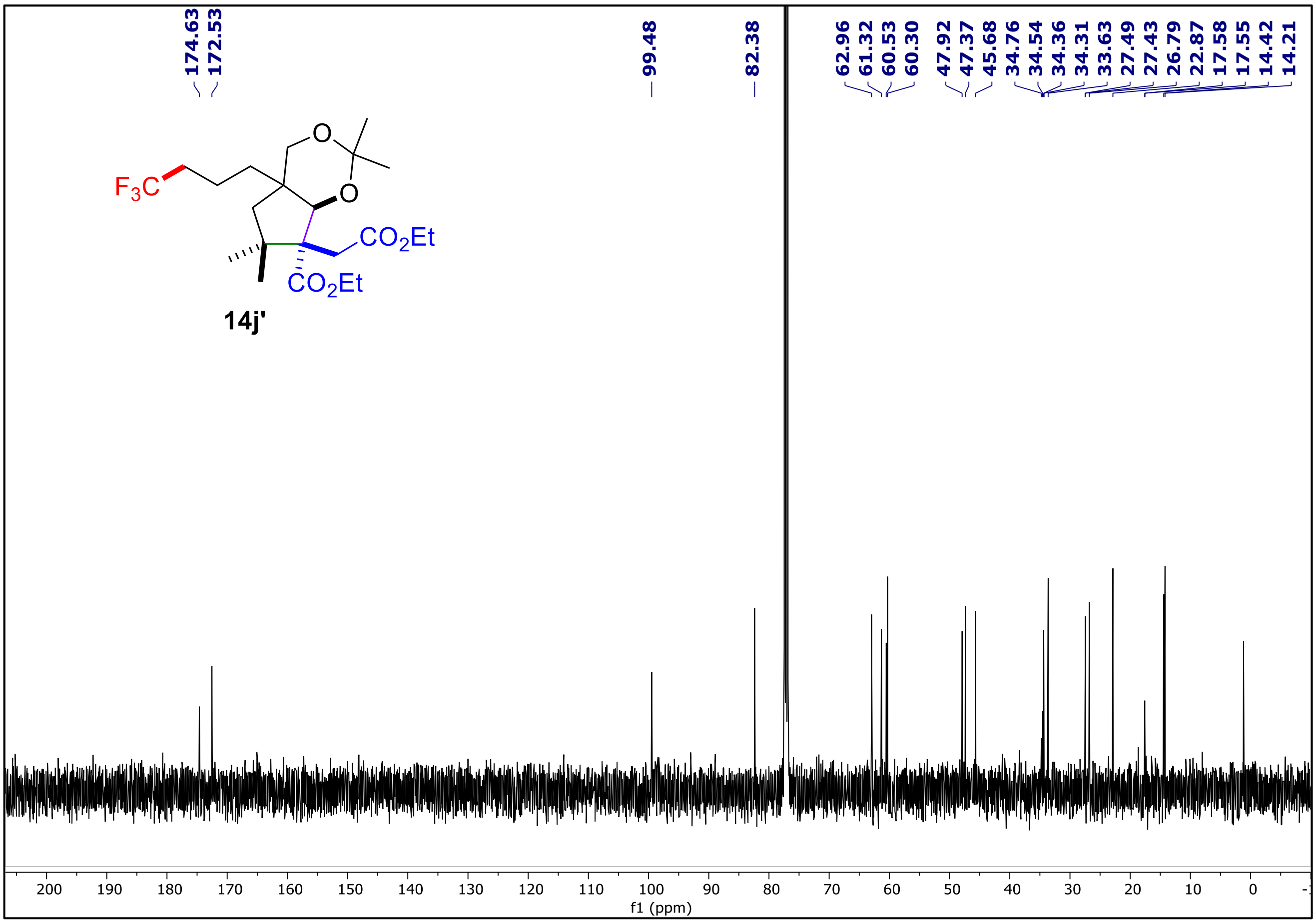

$^{13}C\{^1H\}$ NMR of compound **14j'** (126 MHz, $CDCl_3$)

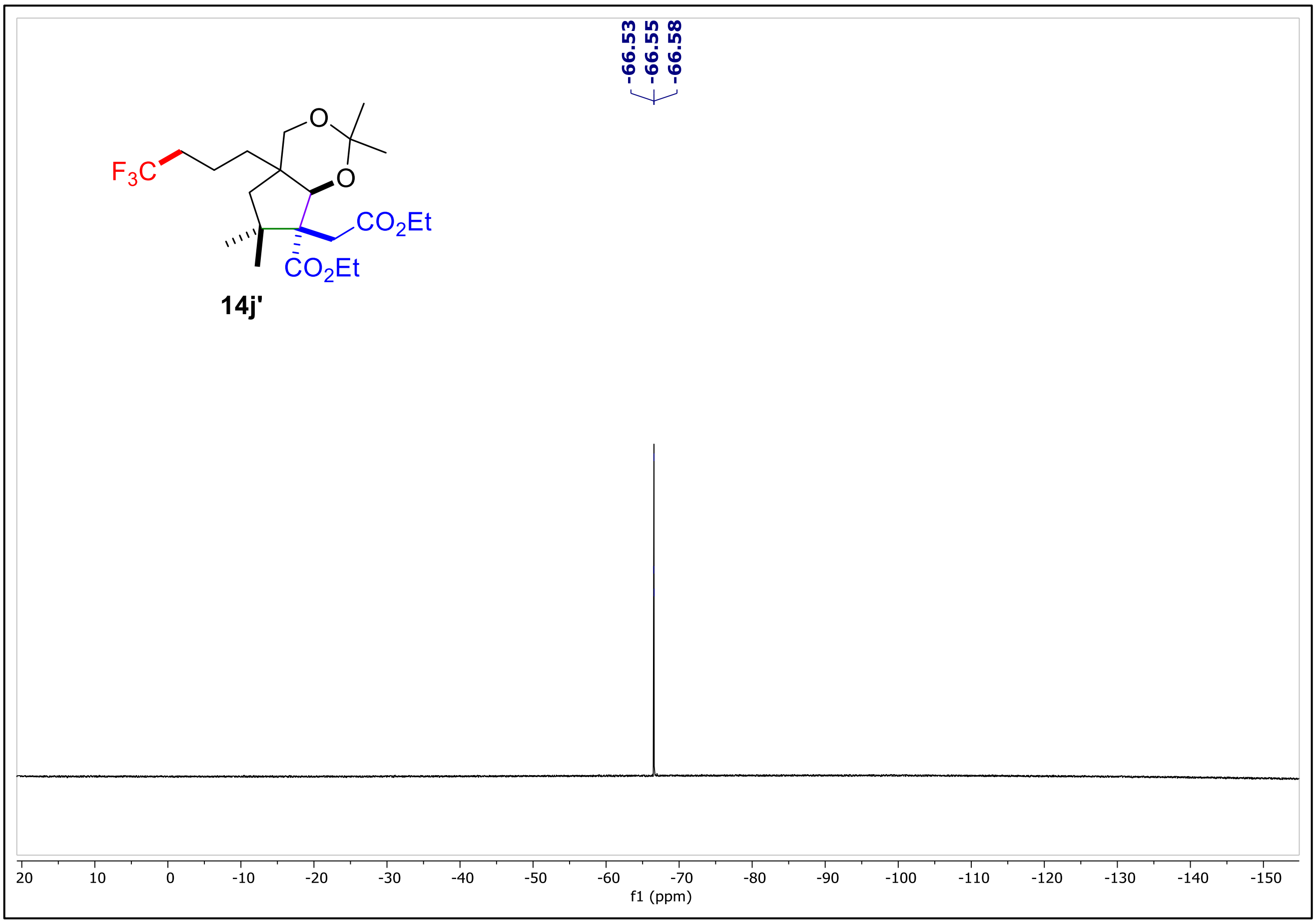

^{19}F NMR of compound **14j'** (471 MHz, $CDCl_3$)

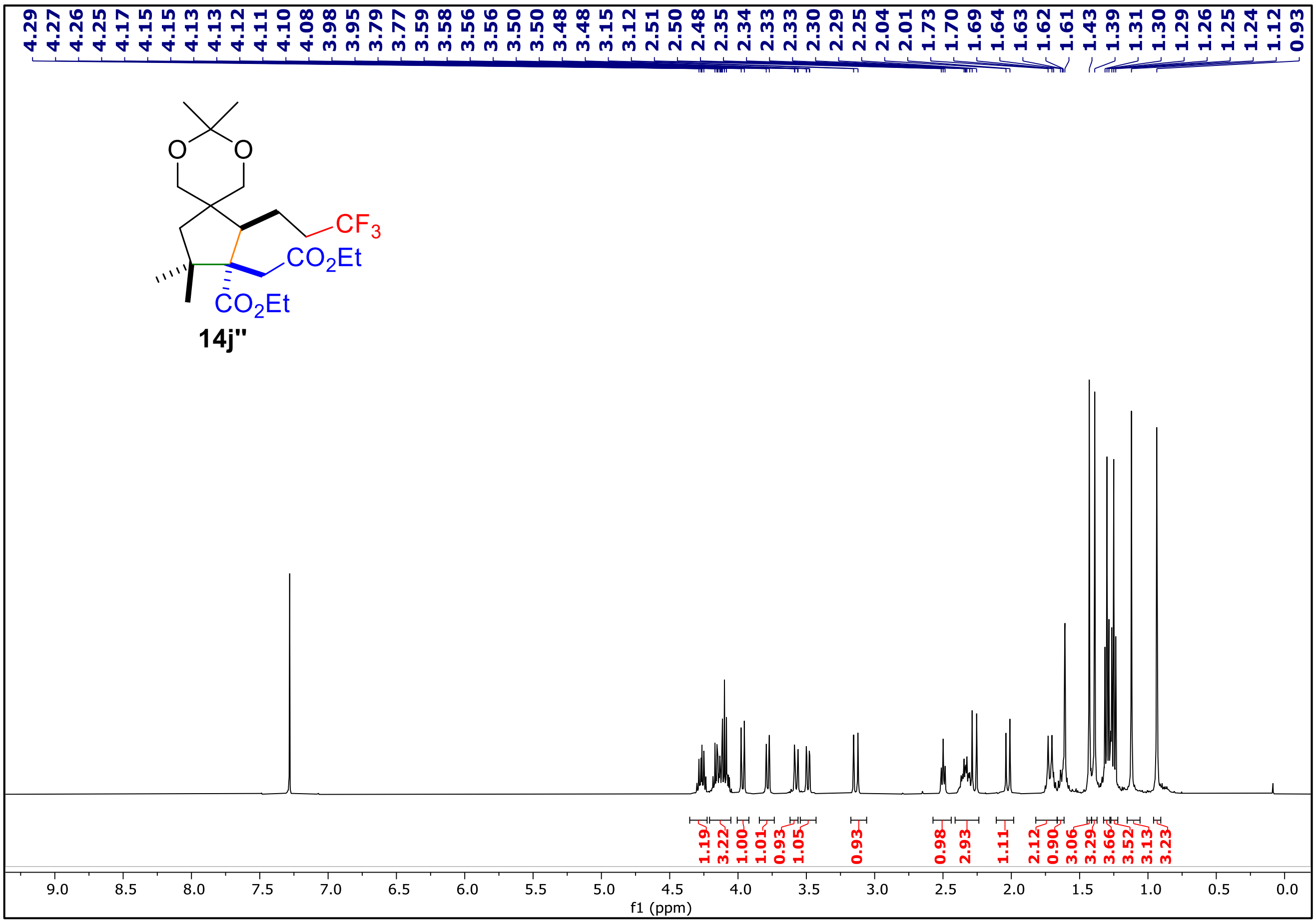

¹H NMR of compound **14j”** (500 MHz, $CDCl_3$)

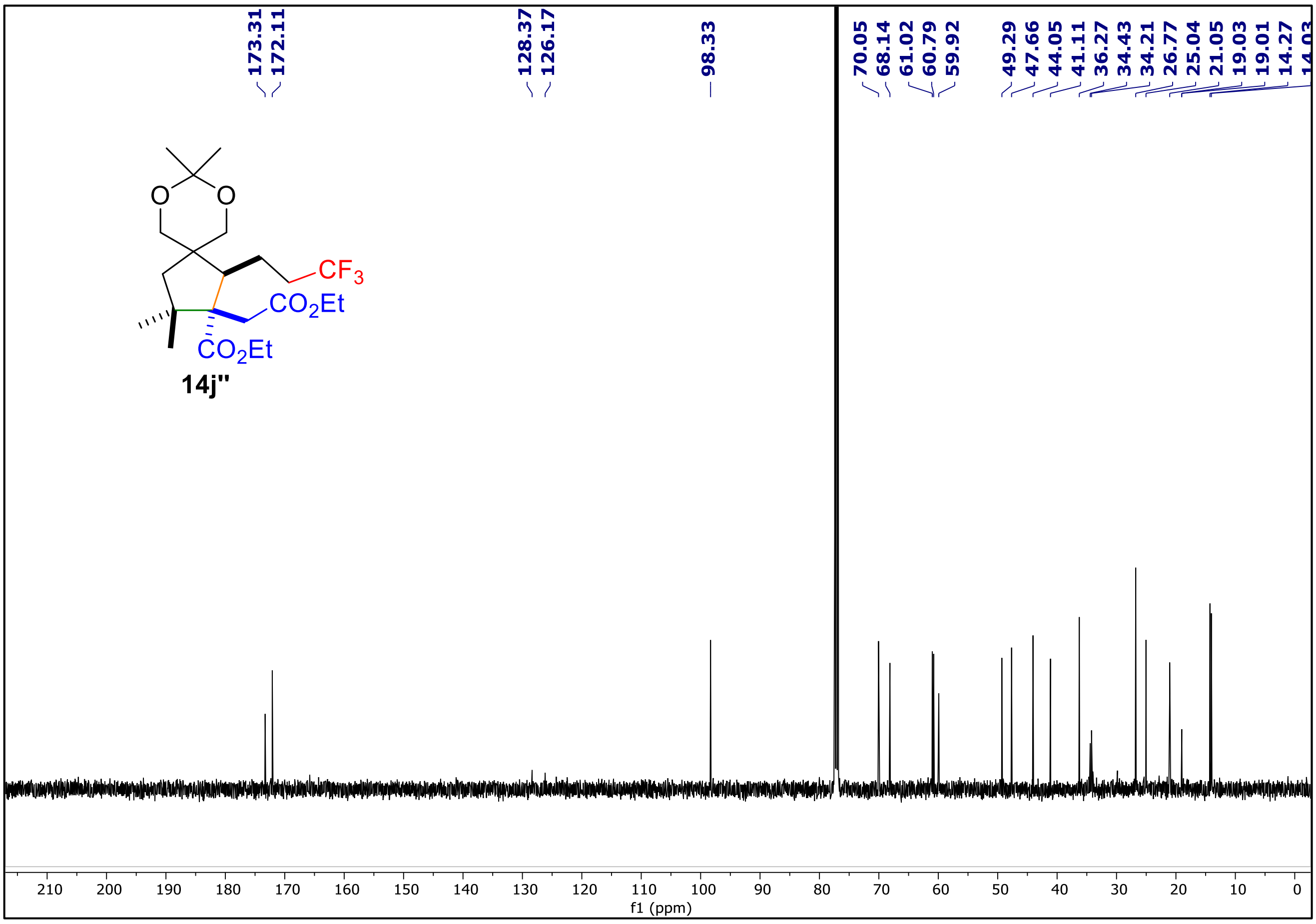

¹³C{¹H} NMR of compound **14j”** (126 MHz, $CDCl_3$)

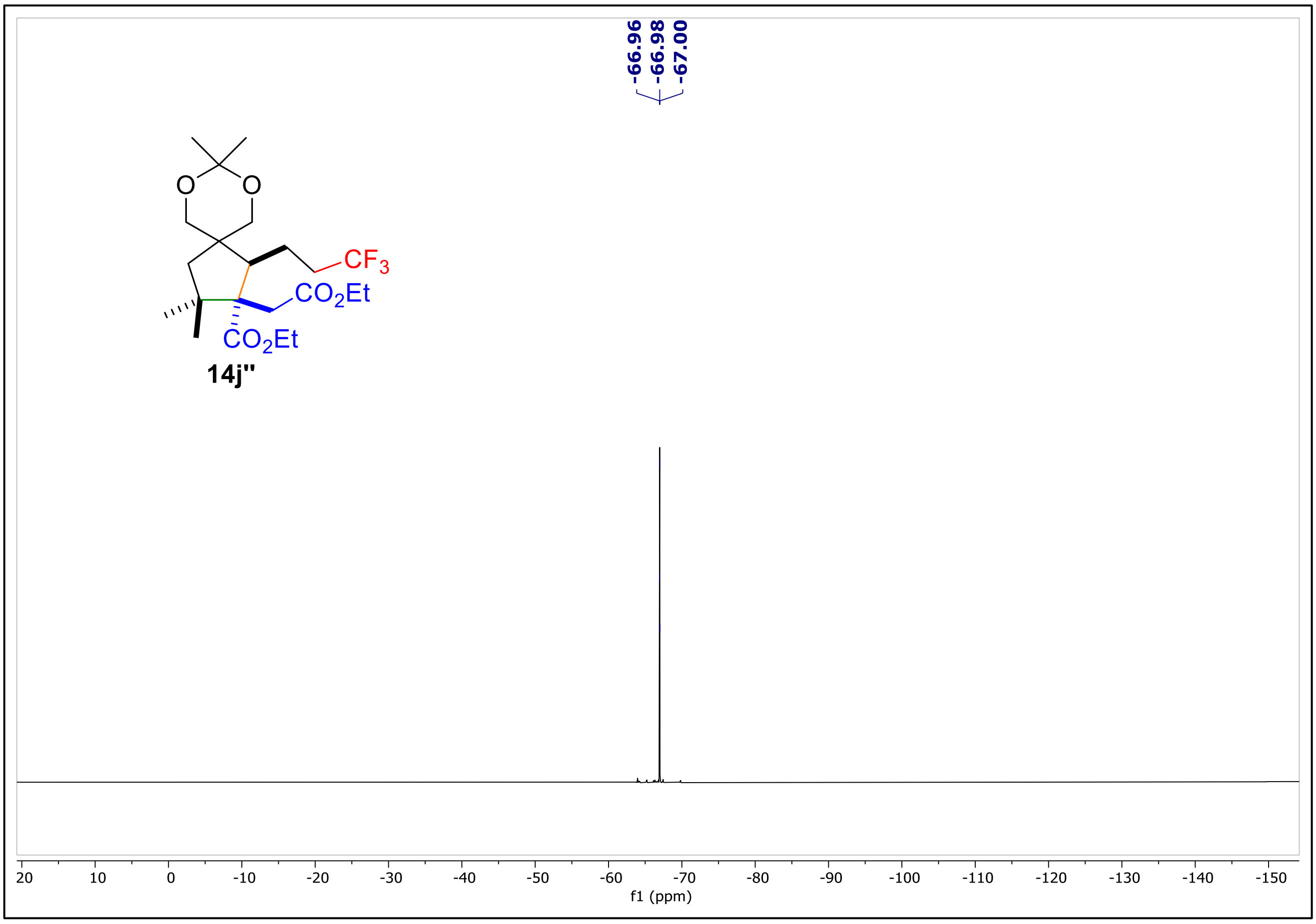

^{19}F NMR of compound **14j”** (471 MHz, $CDCl_3$)

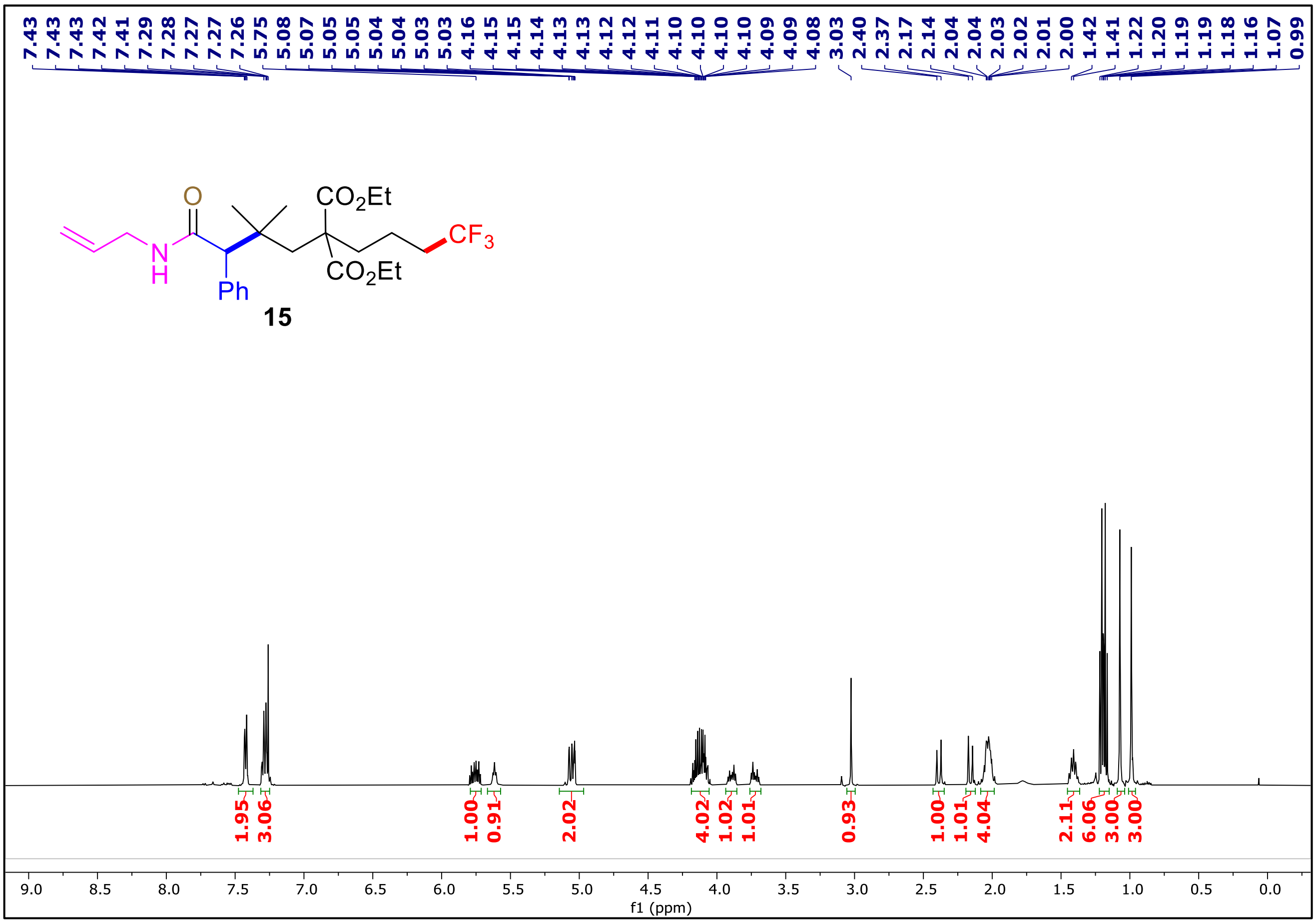

^{1}H NMR of compound **15** (500 MHz, $CDCl_3$)

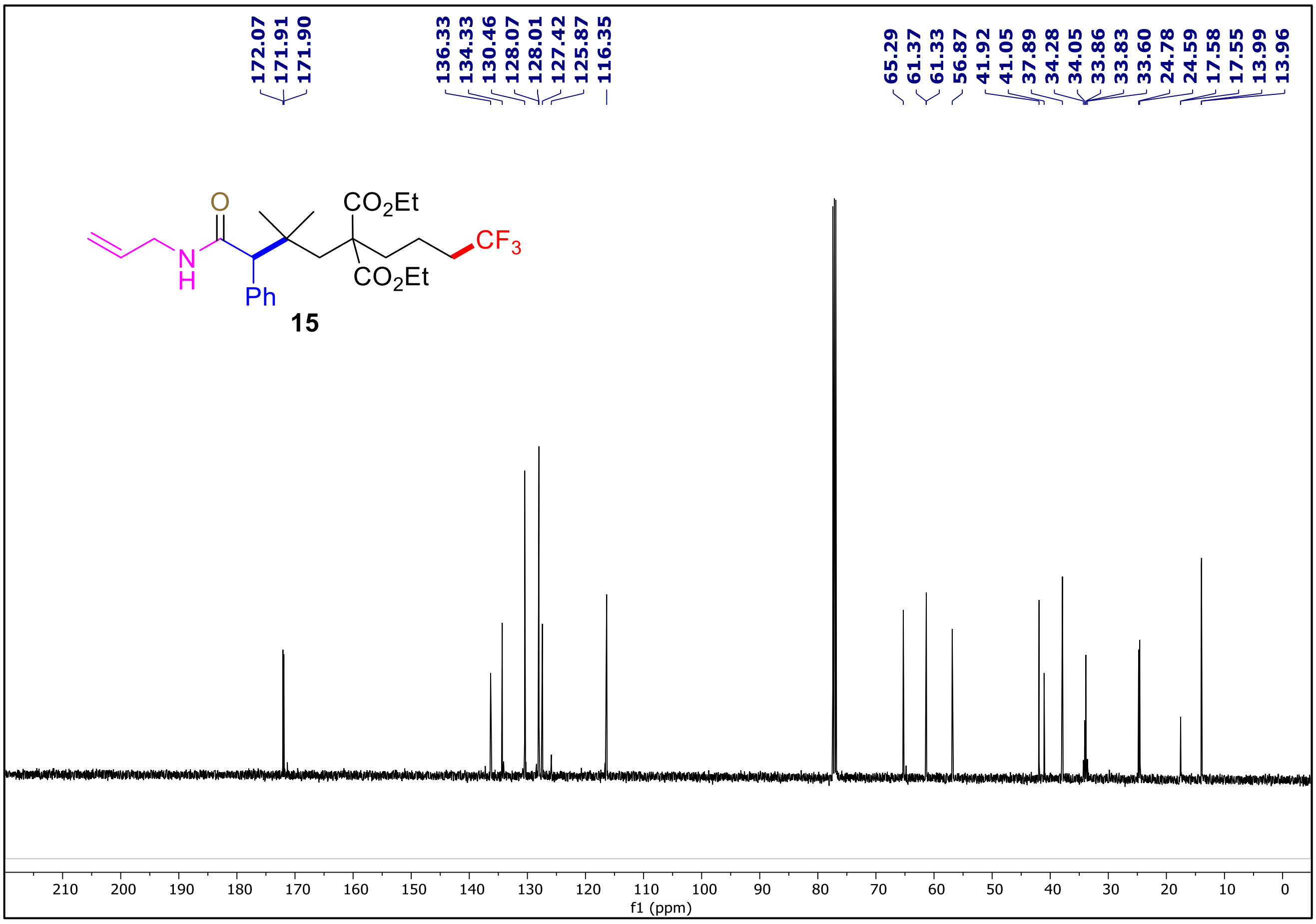

$^{13}C\{^{1}H\}$ NMR of compound **15** (126 MHz, $CDCl_3$)

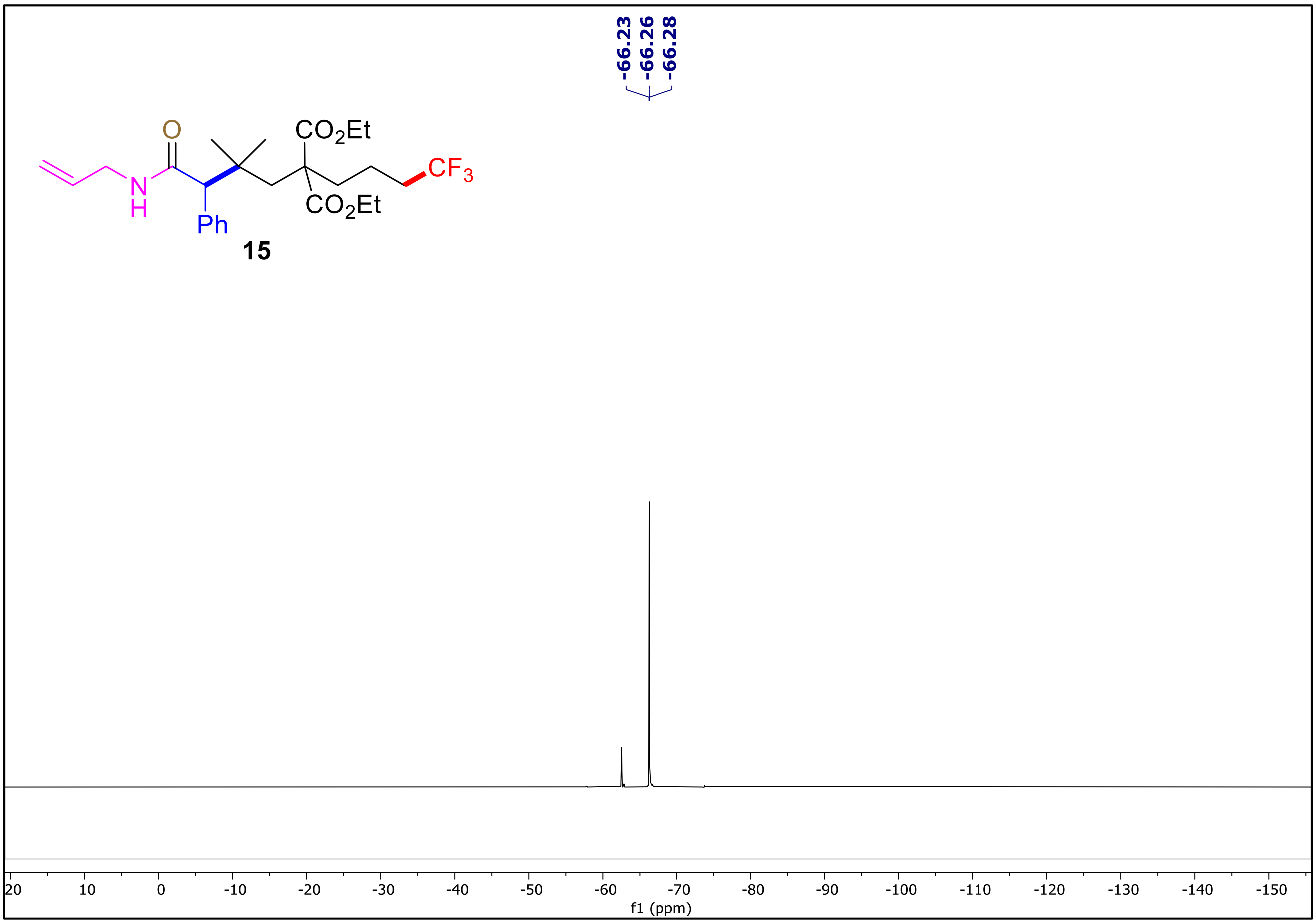

^{19}F NMR of compound **15** (471 MHz, $CDCl_3$)

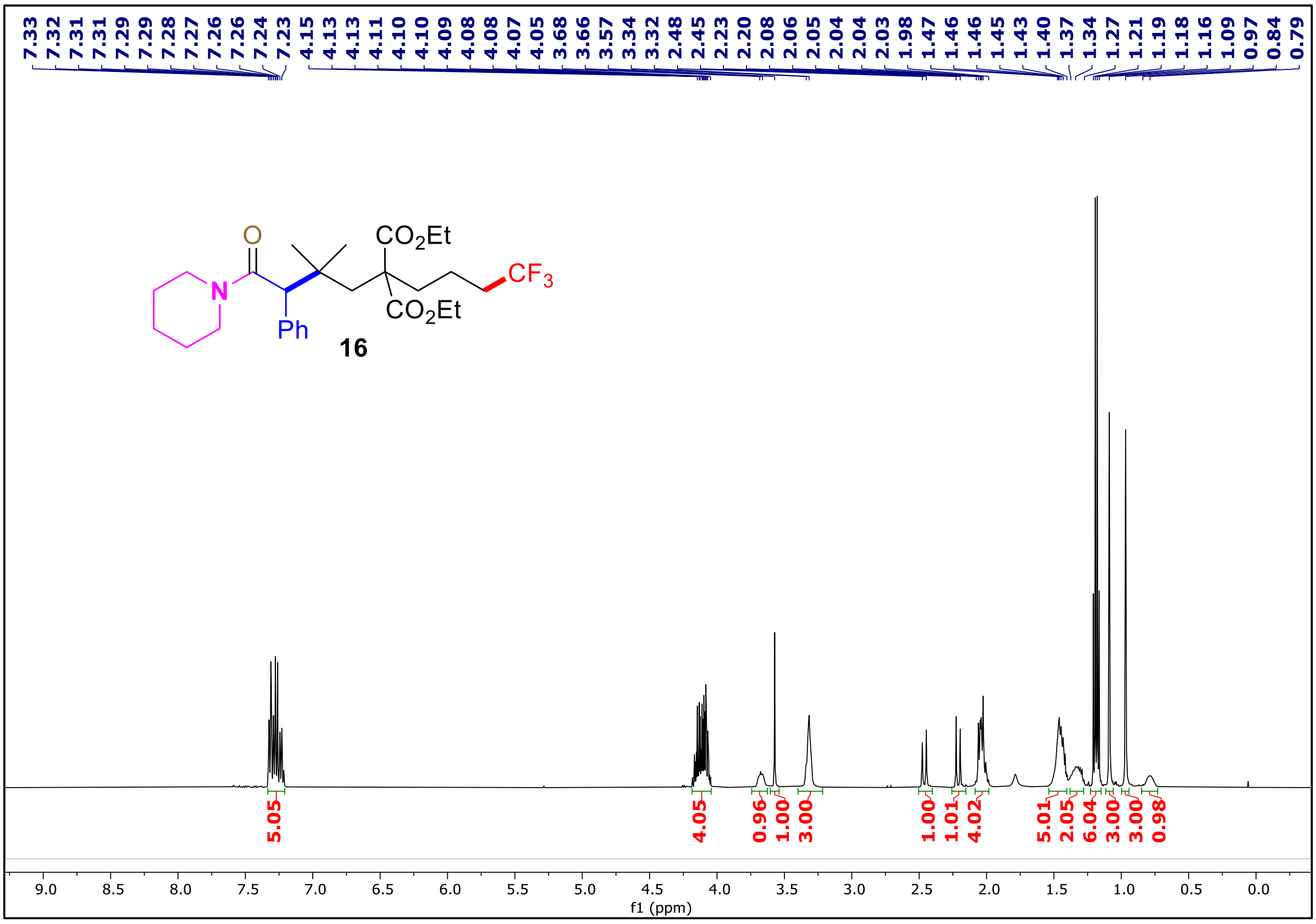

^{1}H NMR of compound **16** (500 MHz, $CDCl_3$)

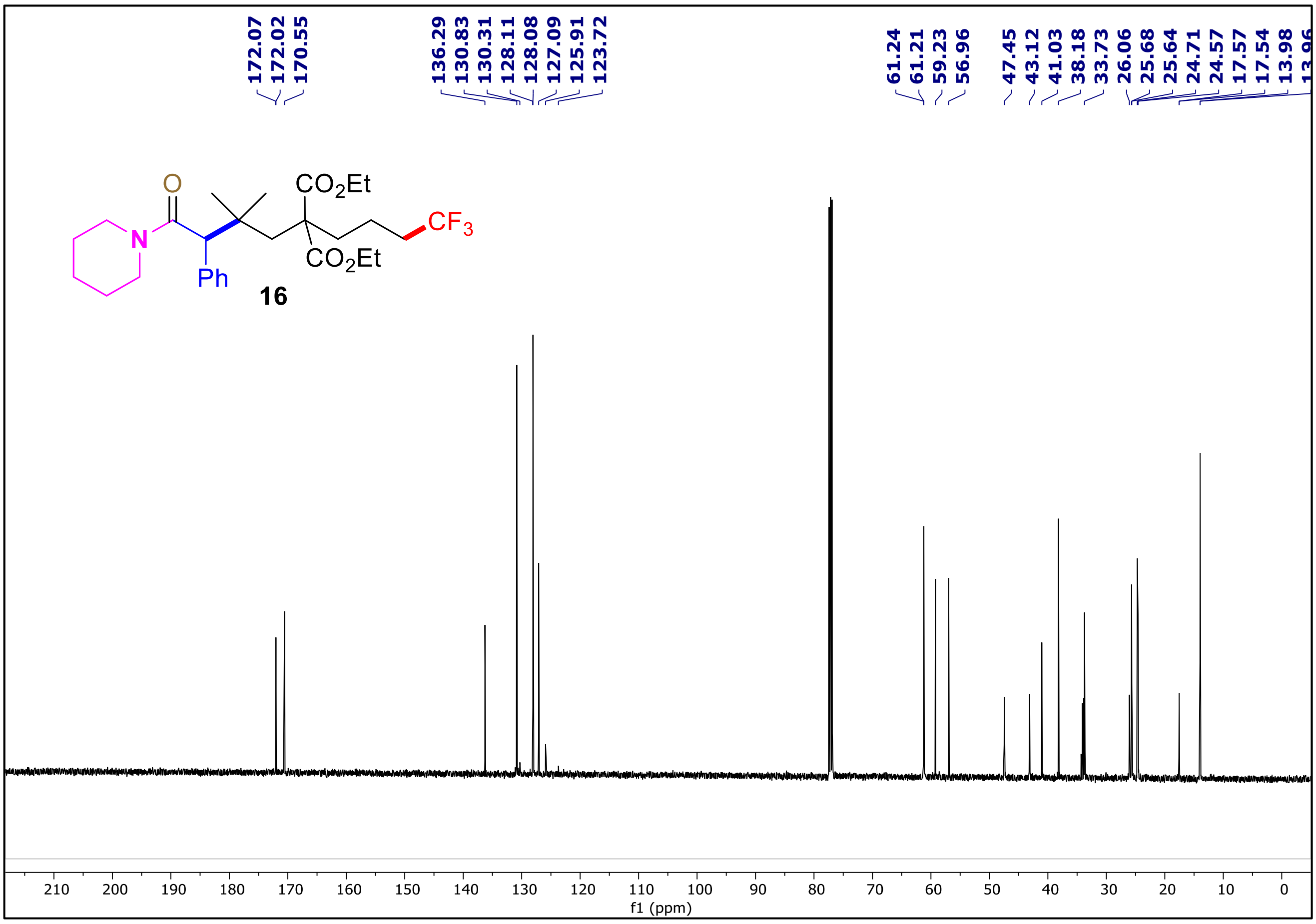

^{13}C{^{1}H} NMR of compound **16** (126 MHz, $CDCl_3$)

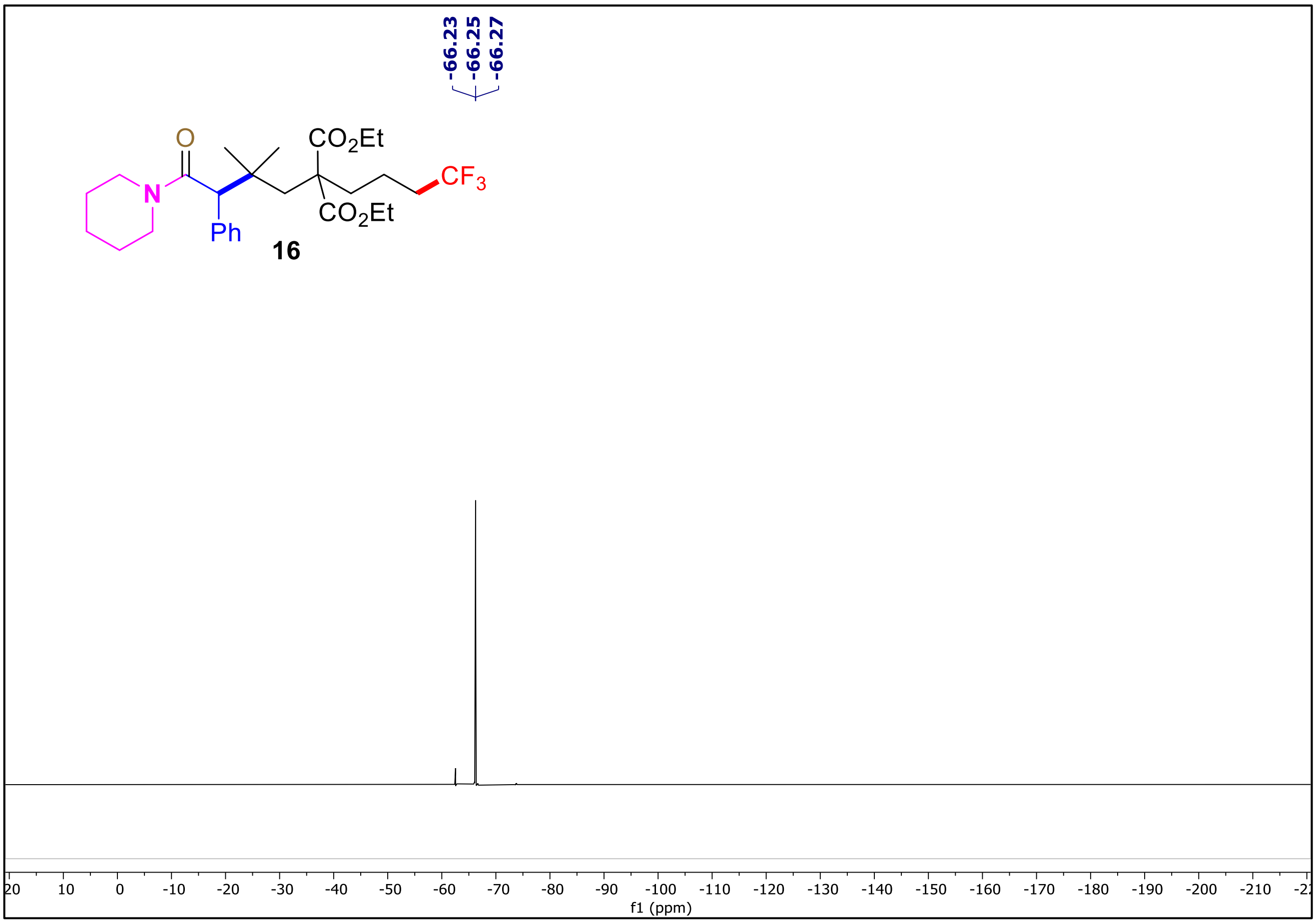

^{19}F NMR of compound **16** (471 MHz, $CDCl_3$)

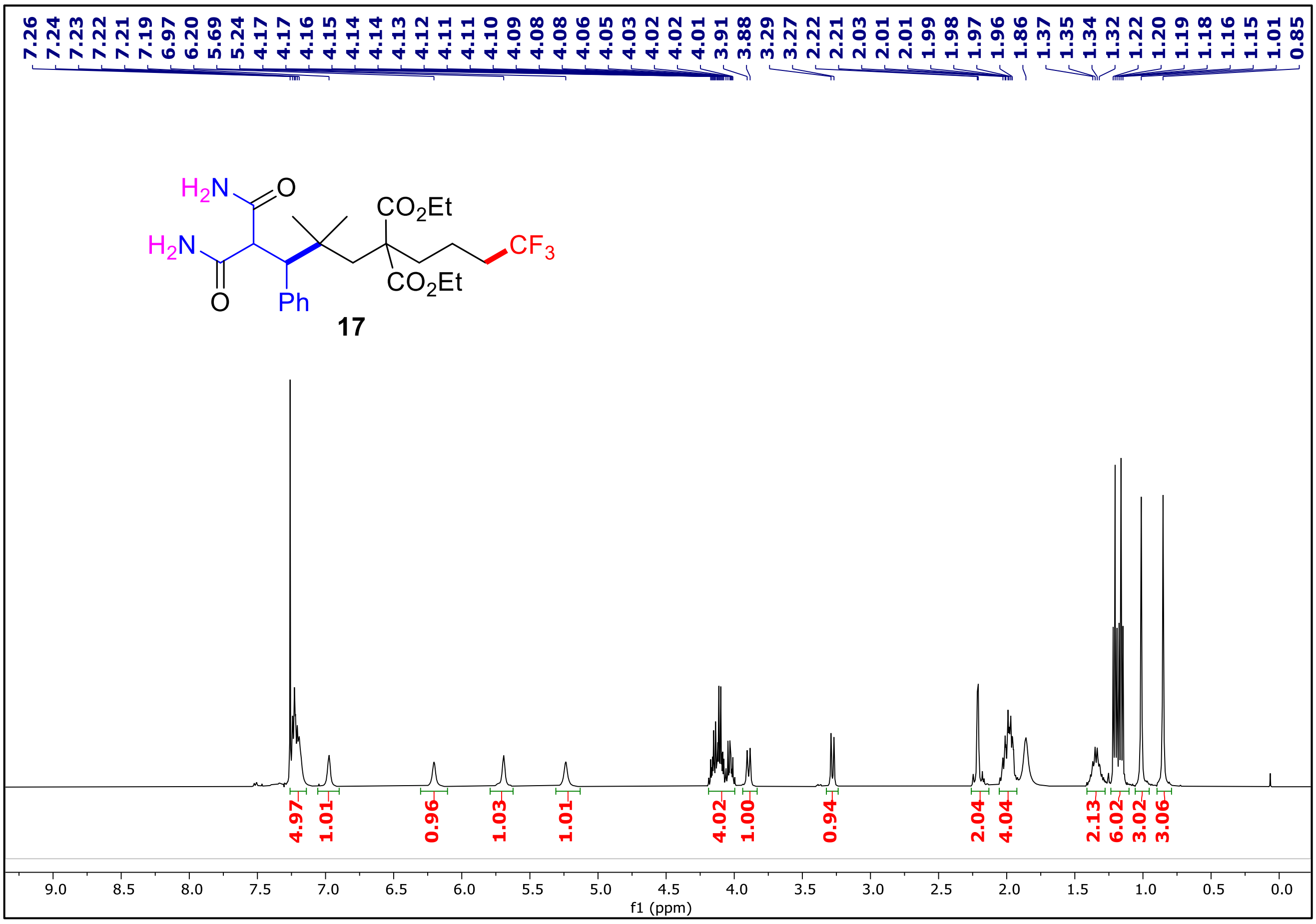

^{1}H NMR of compound **17** (500 MHz, $CDCl_3$)

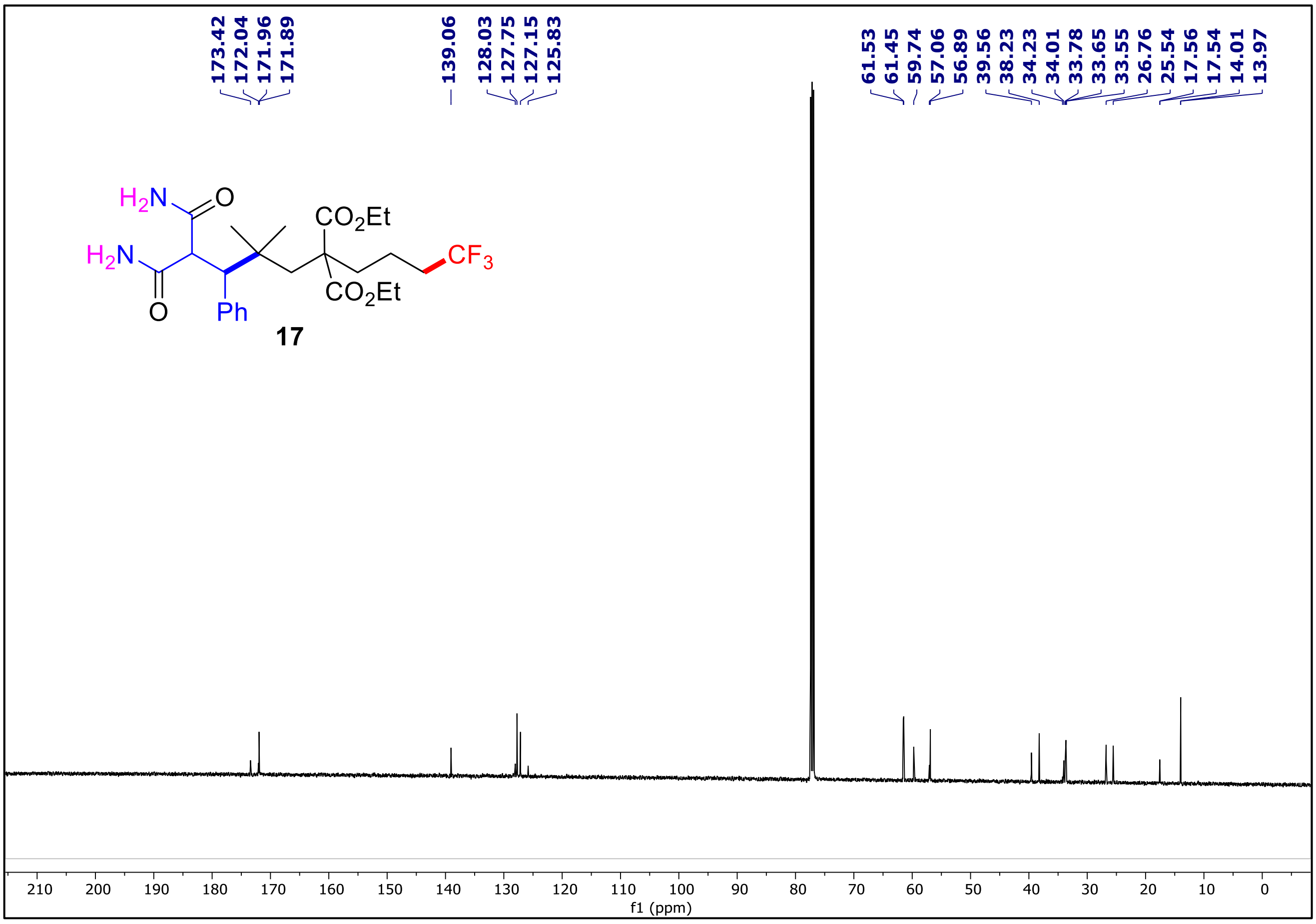

$^{13}C\{^1H\}$ NMR of compound **17** (126 MHz, $CDCl_3$)

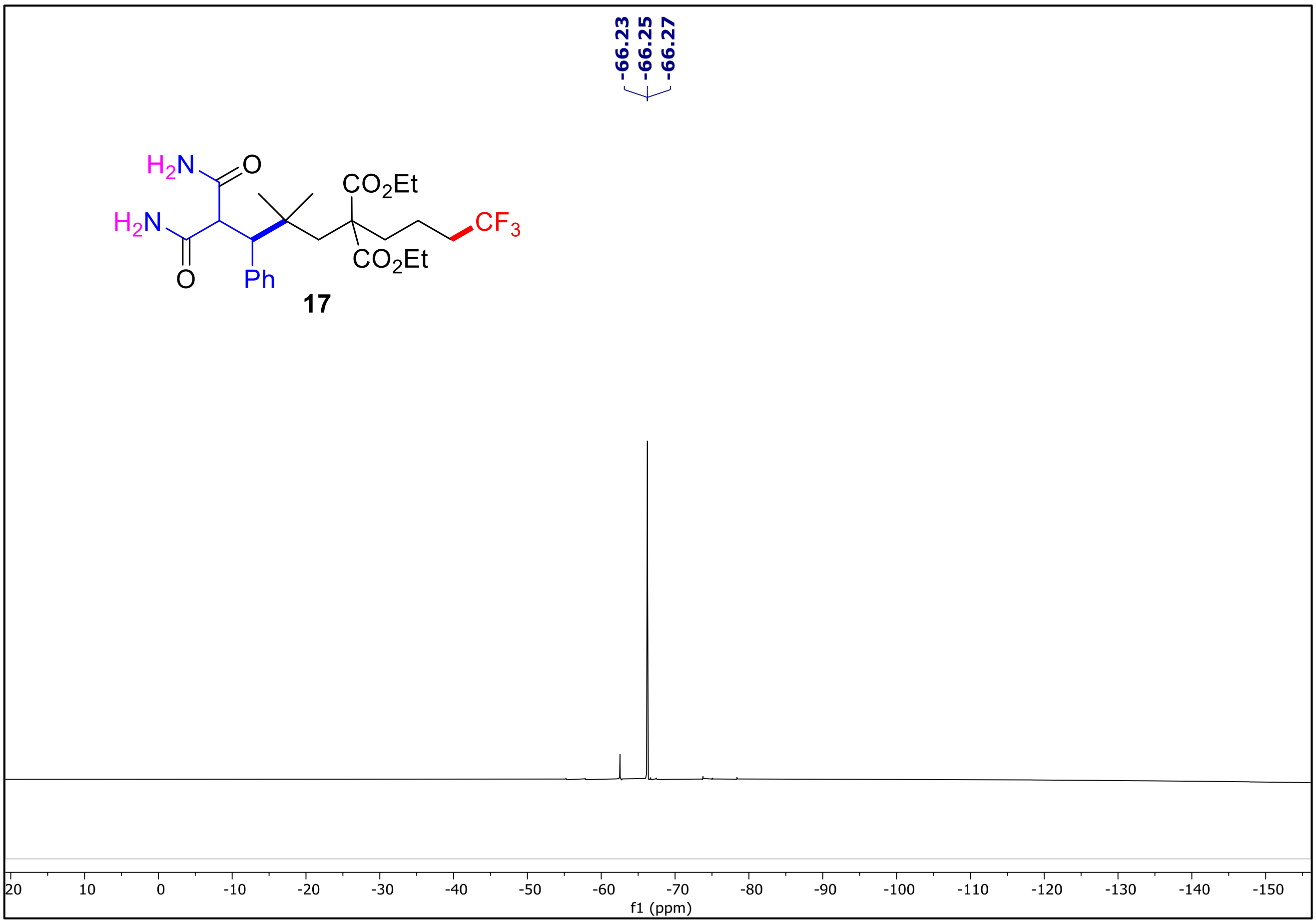

^{19}F NMR of compound **17** (471 MHz, $CDCl_3$)

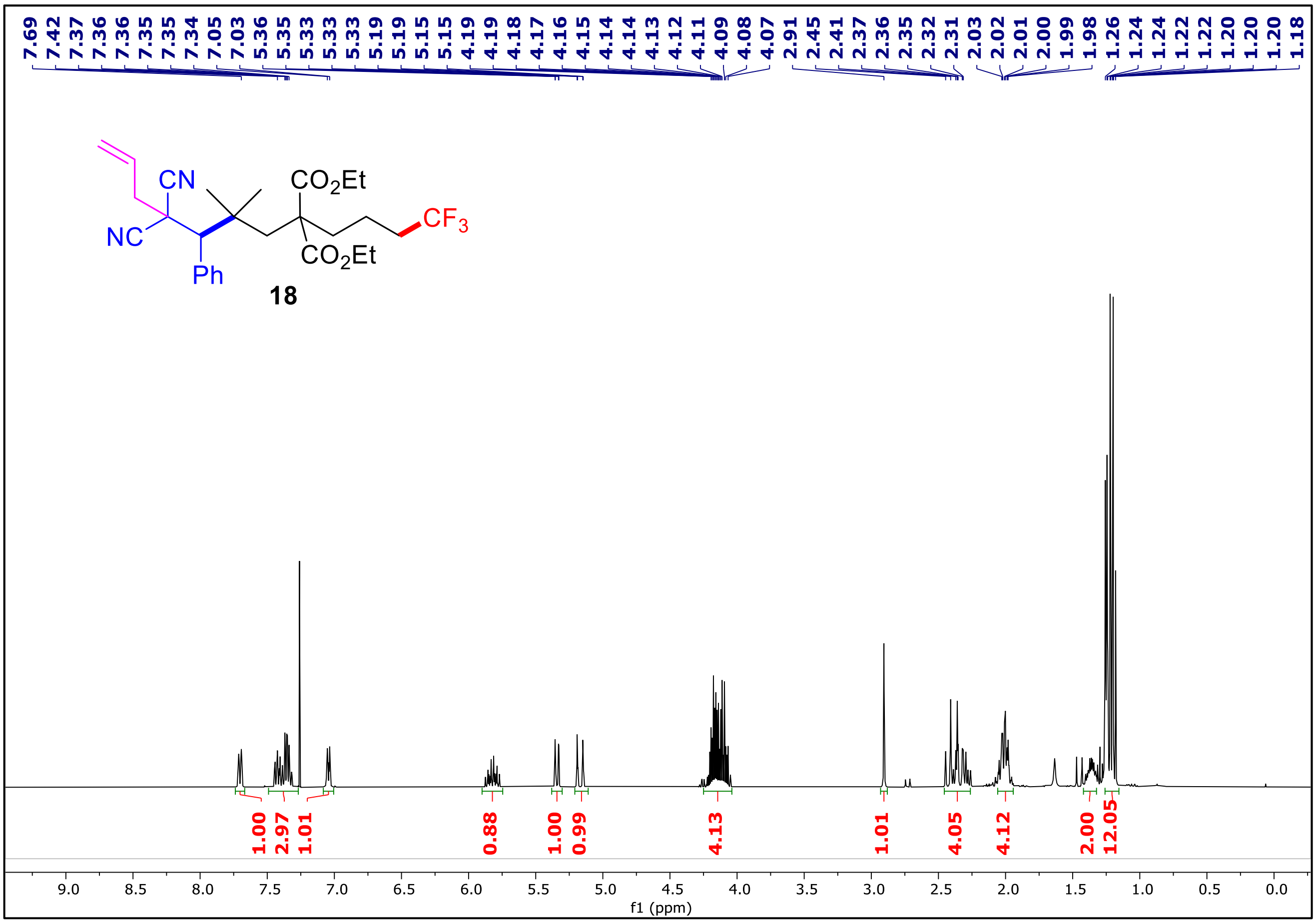

^{1}H NMR of compound **18** (400 MHz, $CDCl_3$)

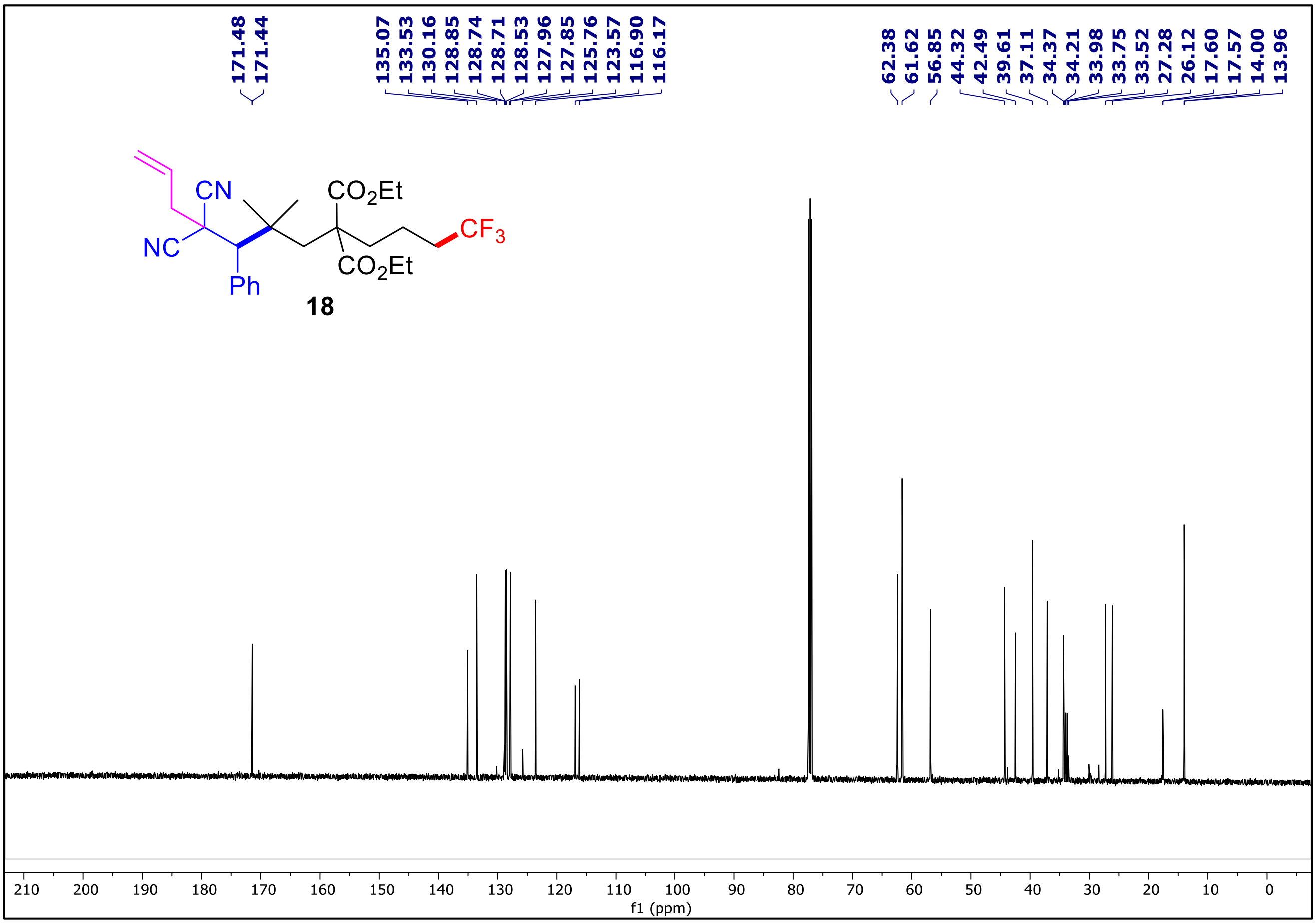

$^{13}C\{^1H\}$ NMR of compound **18** (126 MHz, $CDCl_3$)

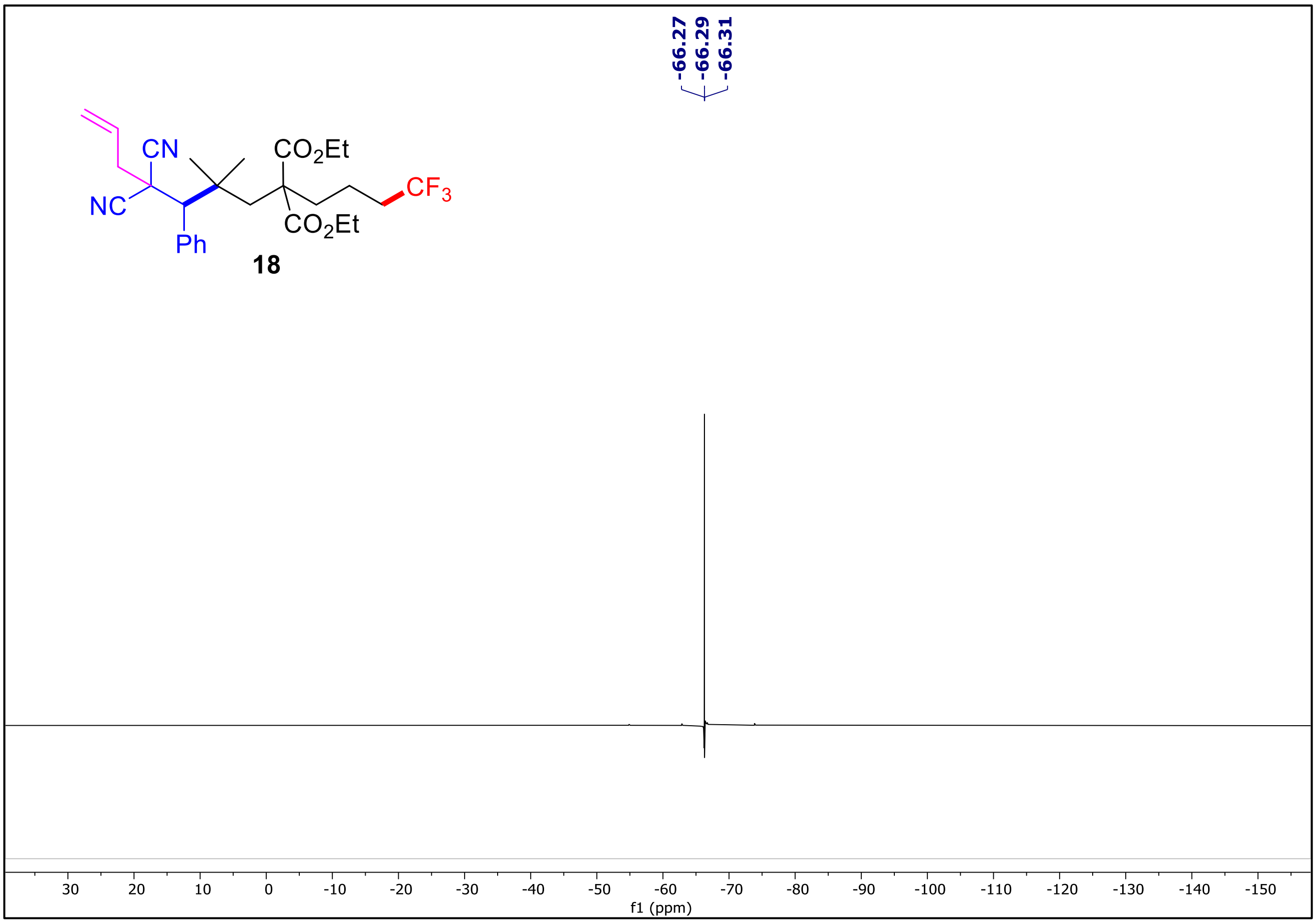

^{19}F NMR of compound **18** (471 MHz, $CDCl_3$)

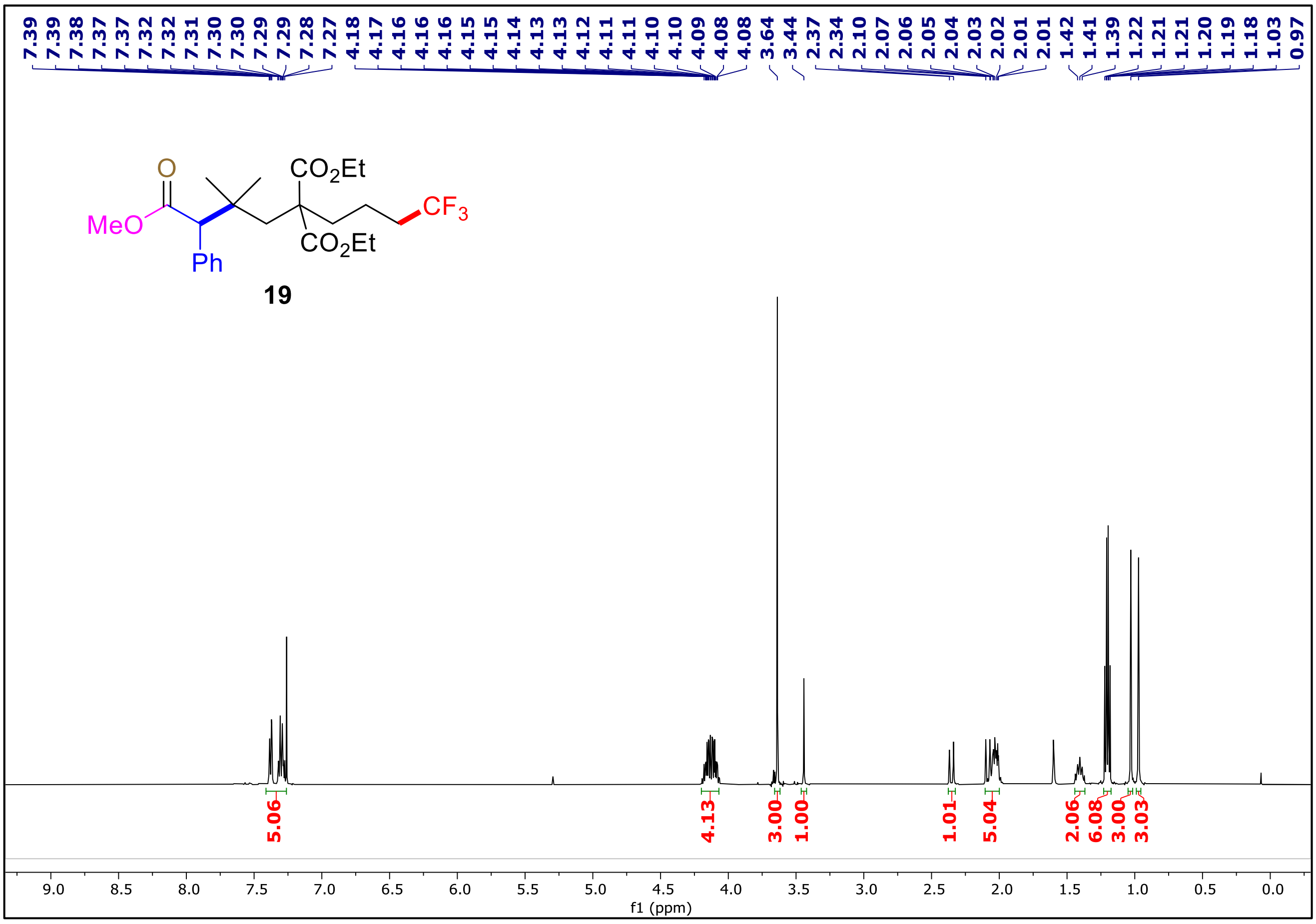

^{1}H NMR of compound **19** (500 MHz, $CDCl_3$)

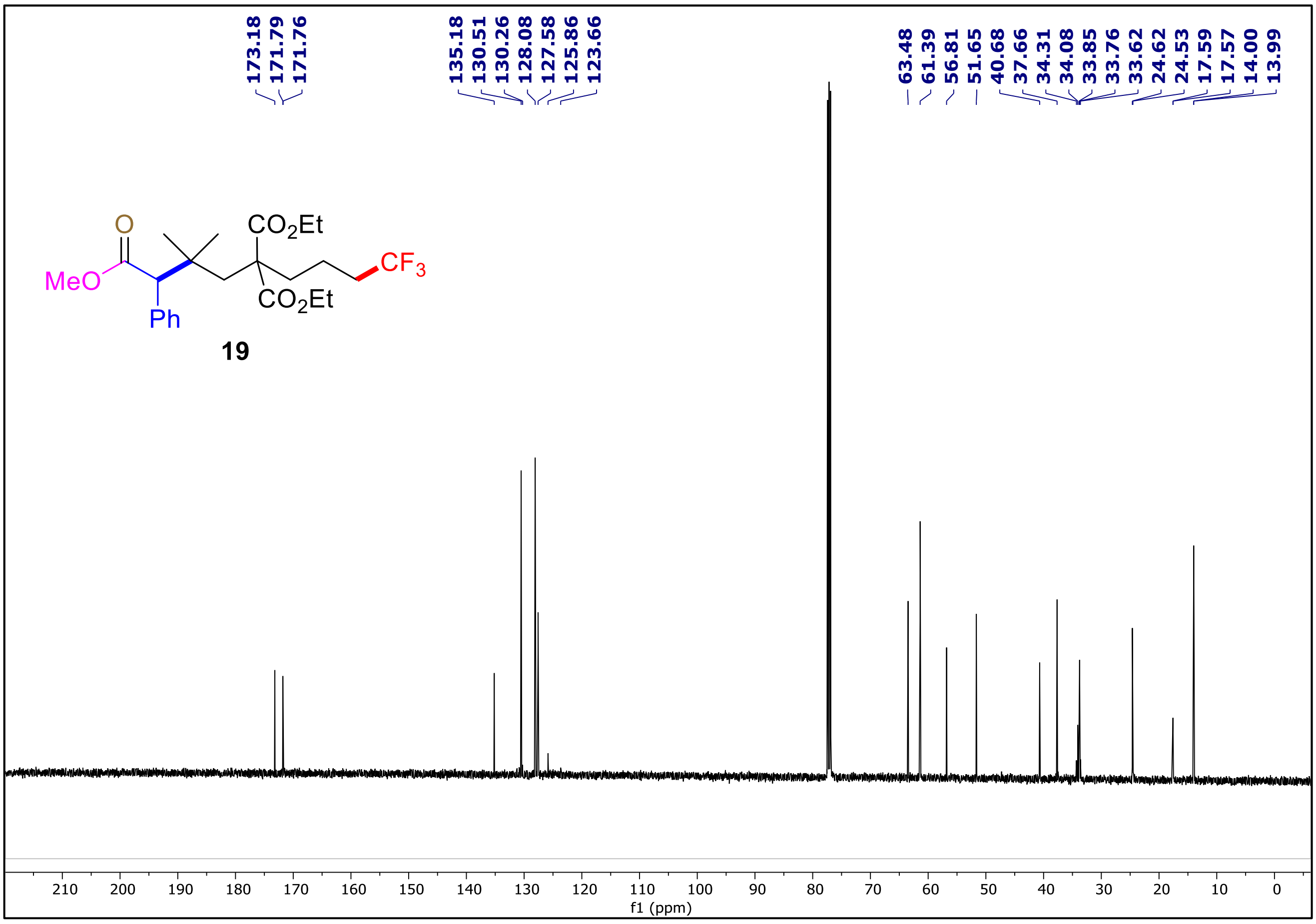

$^{13}C\{^{1}H\}$ NMR of compound **19** (126 MHz, $CDCl_3$)

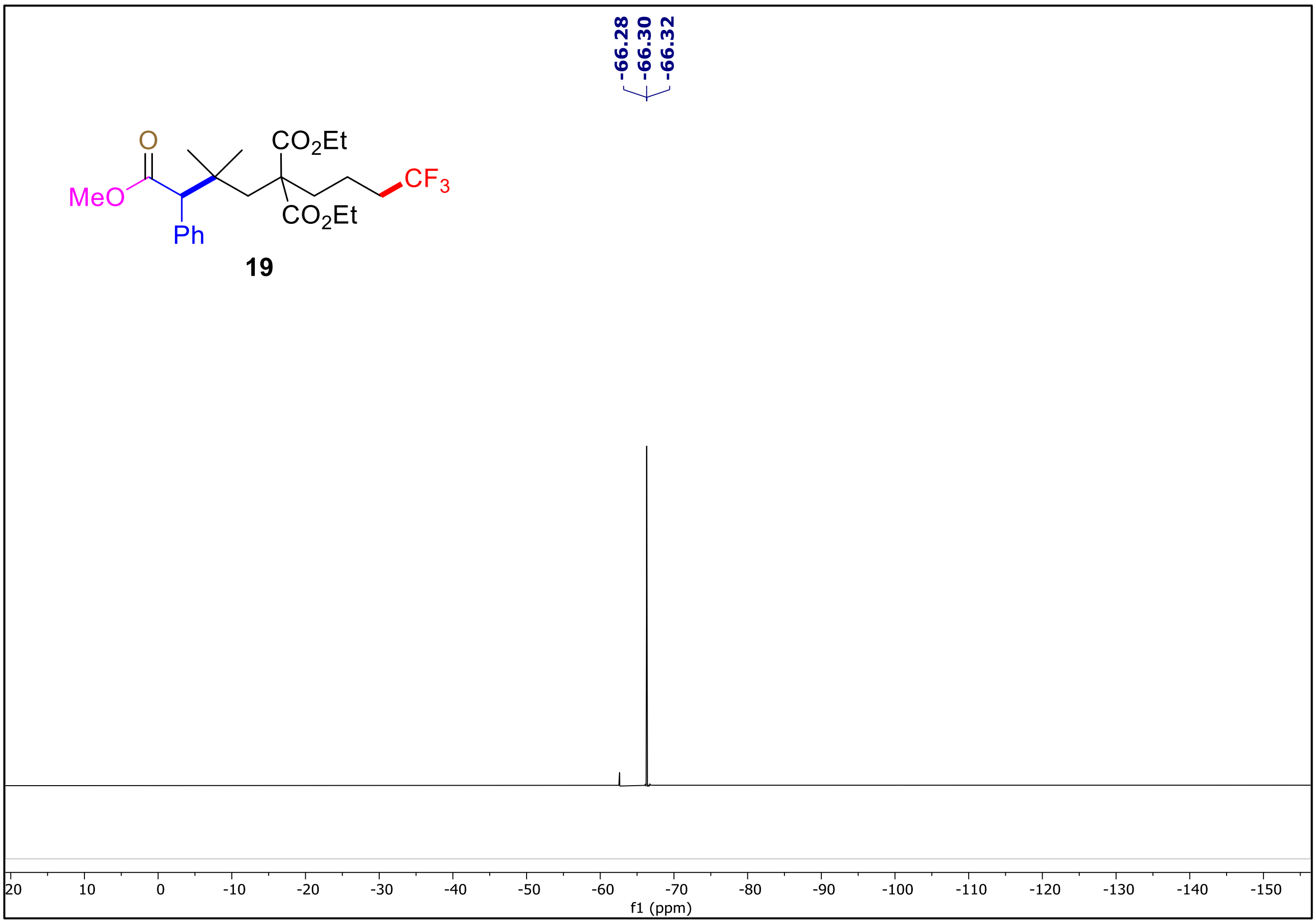

^{19}F NMR of compound **19** (471 MHz, $CDCl_3$)

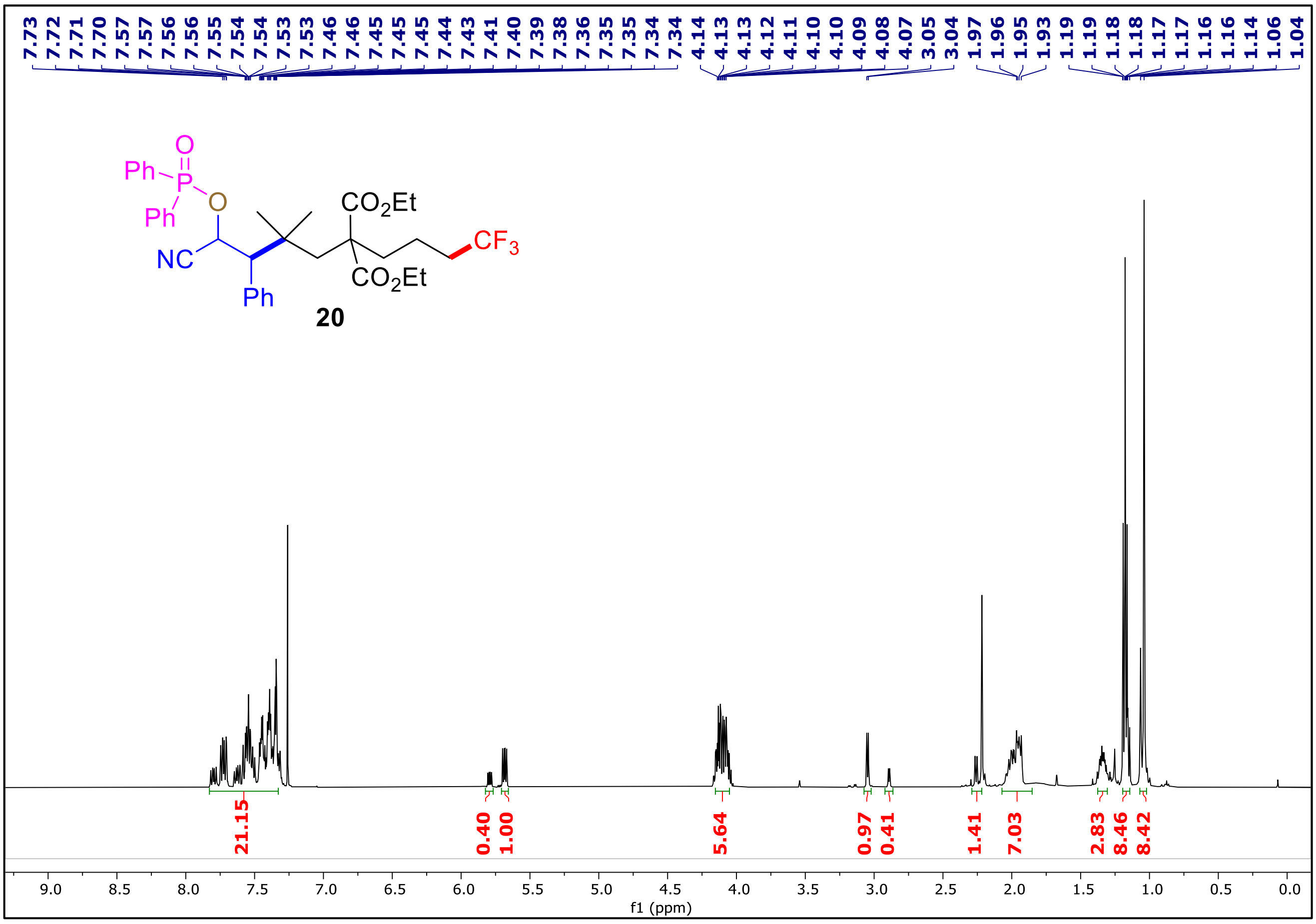

^{1}H NMR of compound **20** (500 MHz, CDCl$_3$)

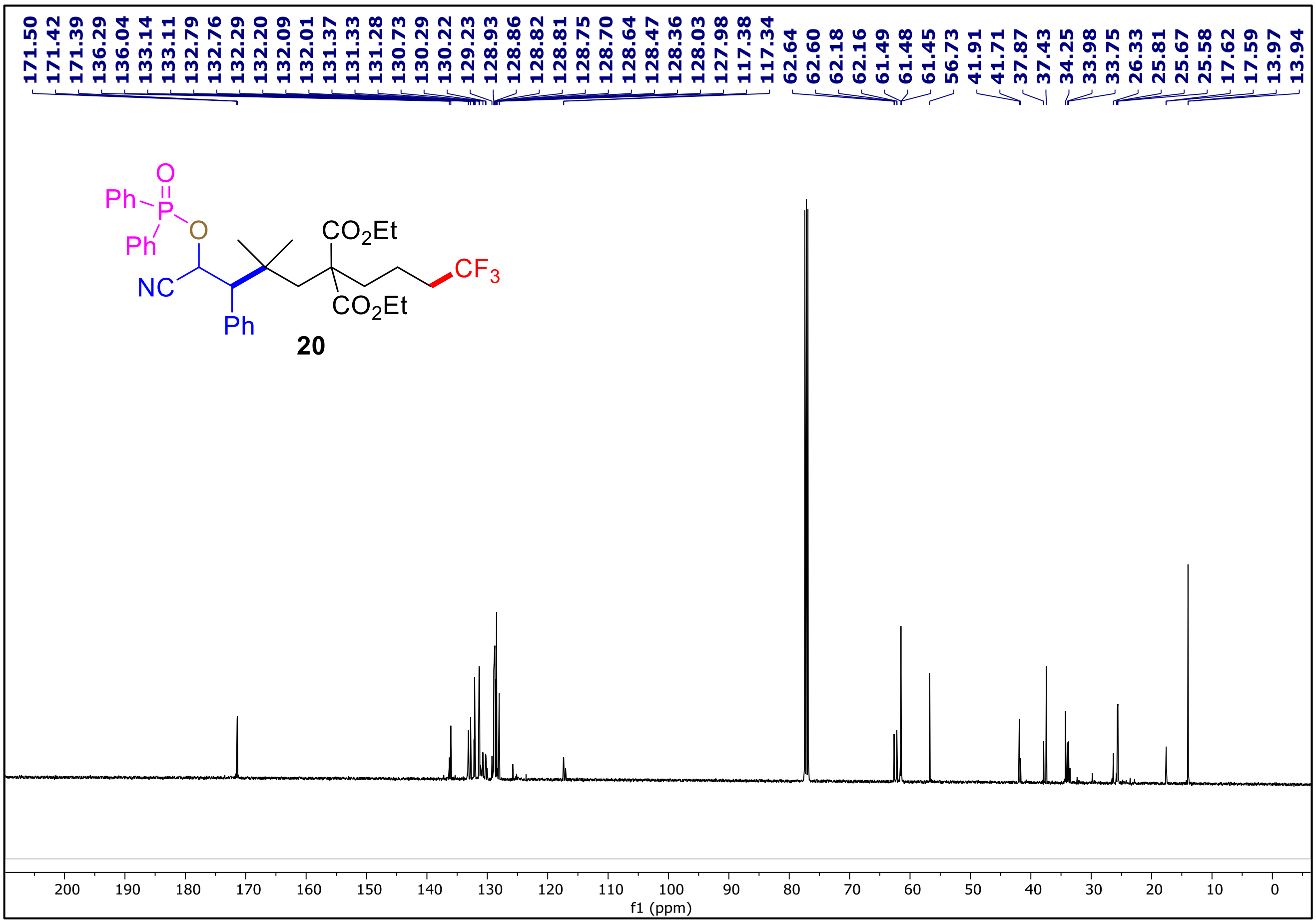

^{13}C{^{1}H} NMR of compound **20** (126 MHz, CDCl$_3$)

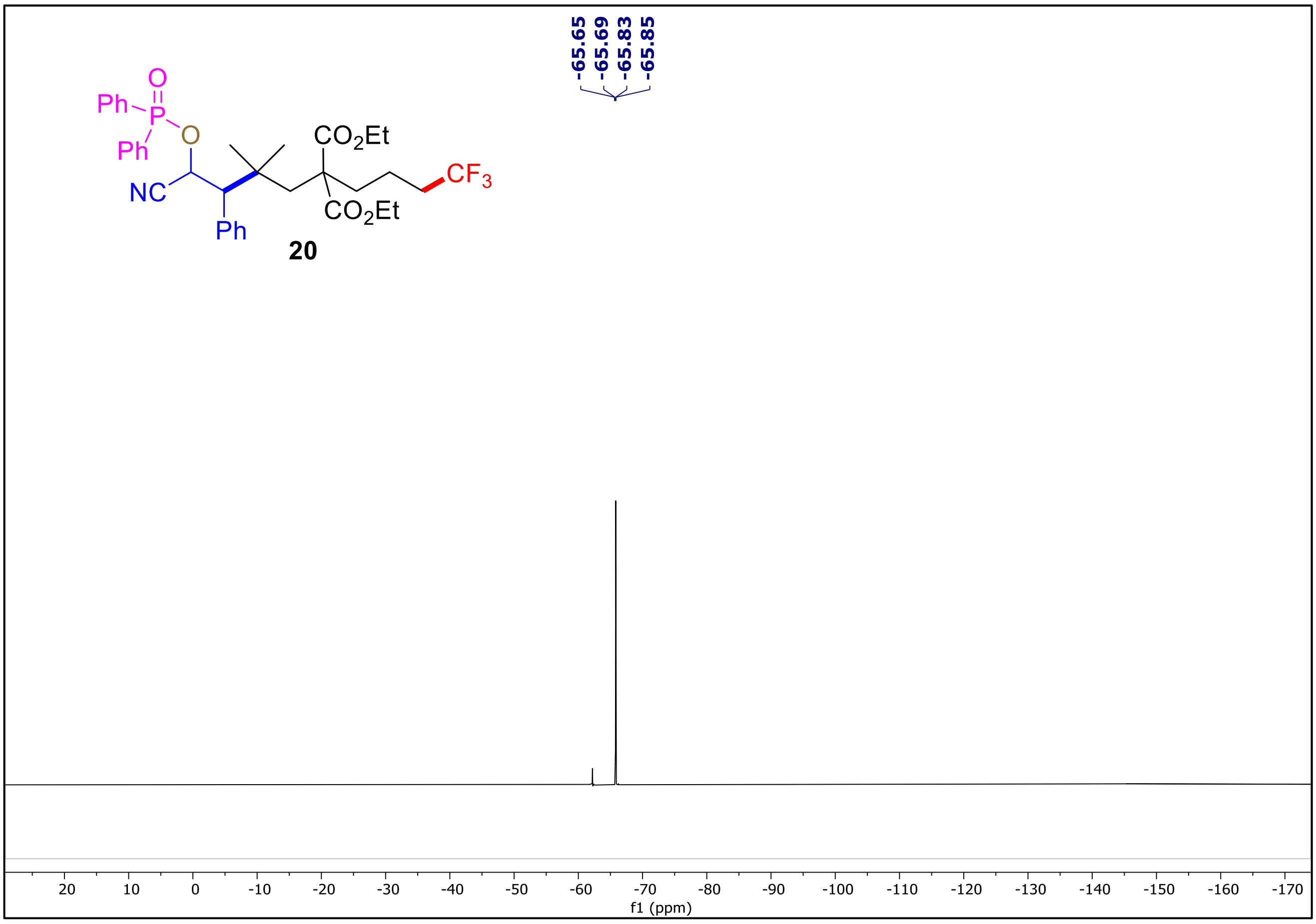

^{19}F NMR of compound **20** (471 MHz, $CDCl_3$)

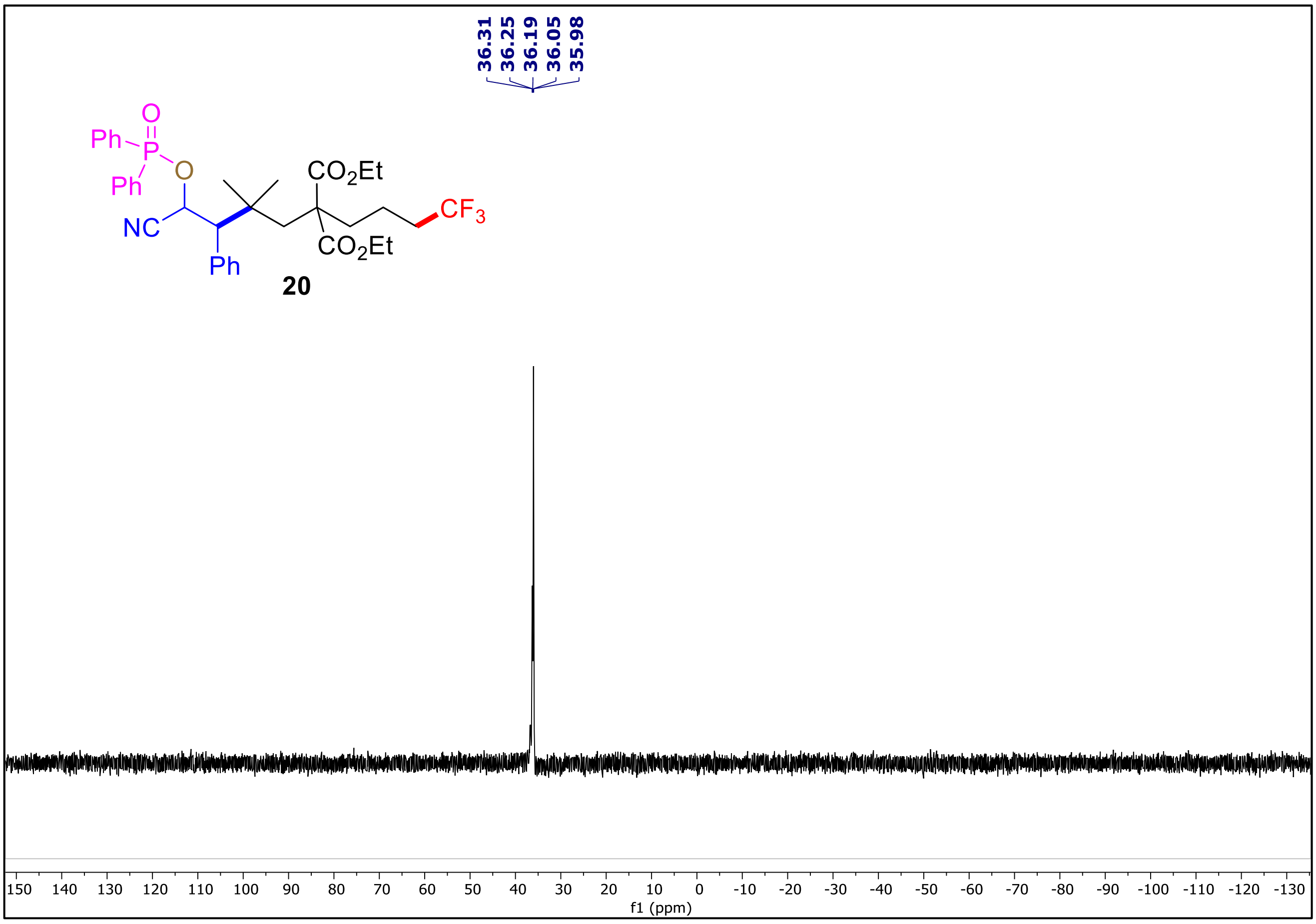

^{31}P NMR of compound **20** (202 MHz, $CDCl_3$)

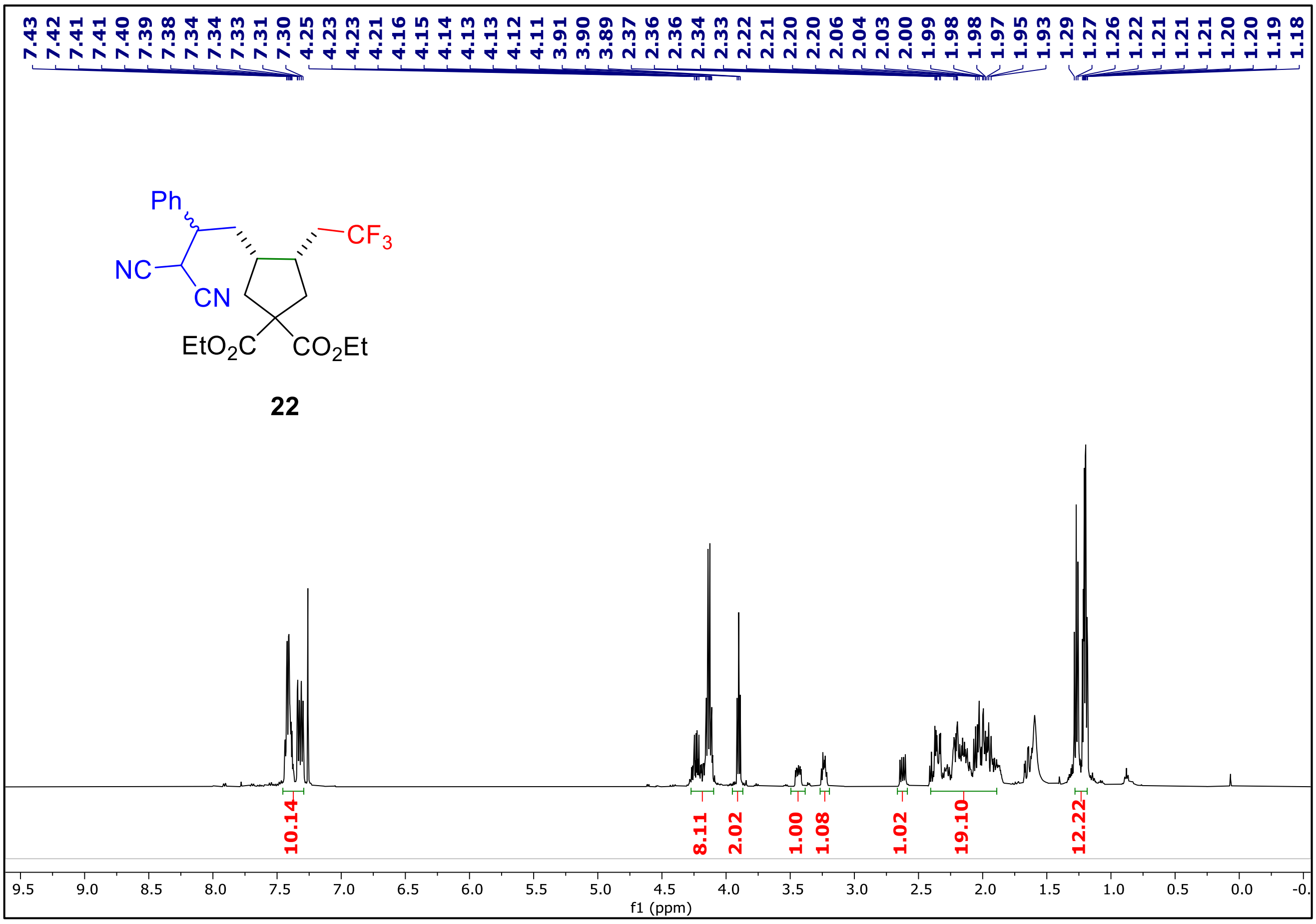

^{1}H NMR of compound **22** (500 MHz, $CDCl_3$)

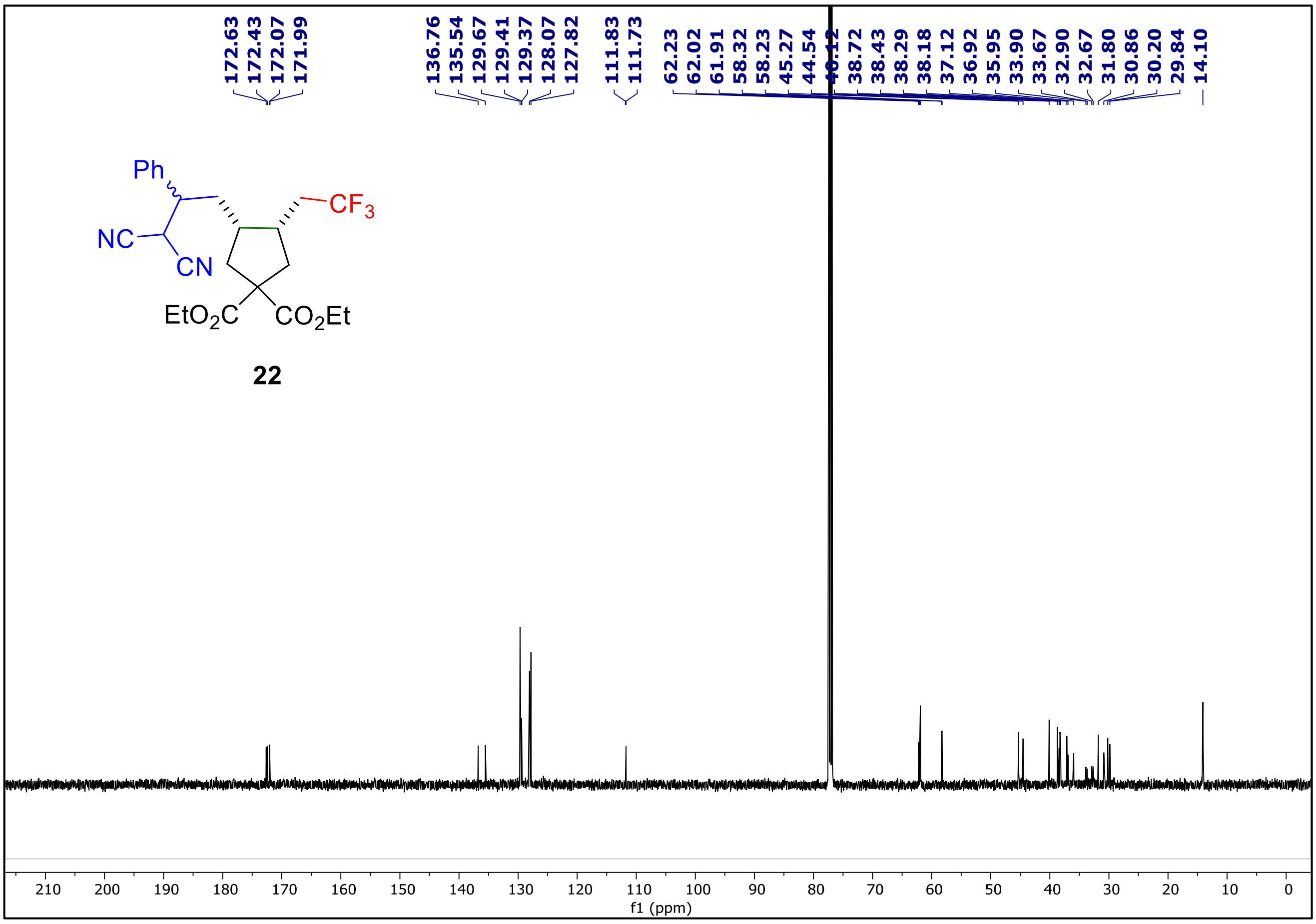

^{13}C{^{1}H} NMR of compound **22** (126 MHz, $CDCl_3$)

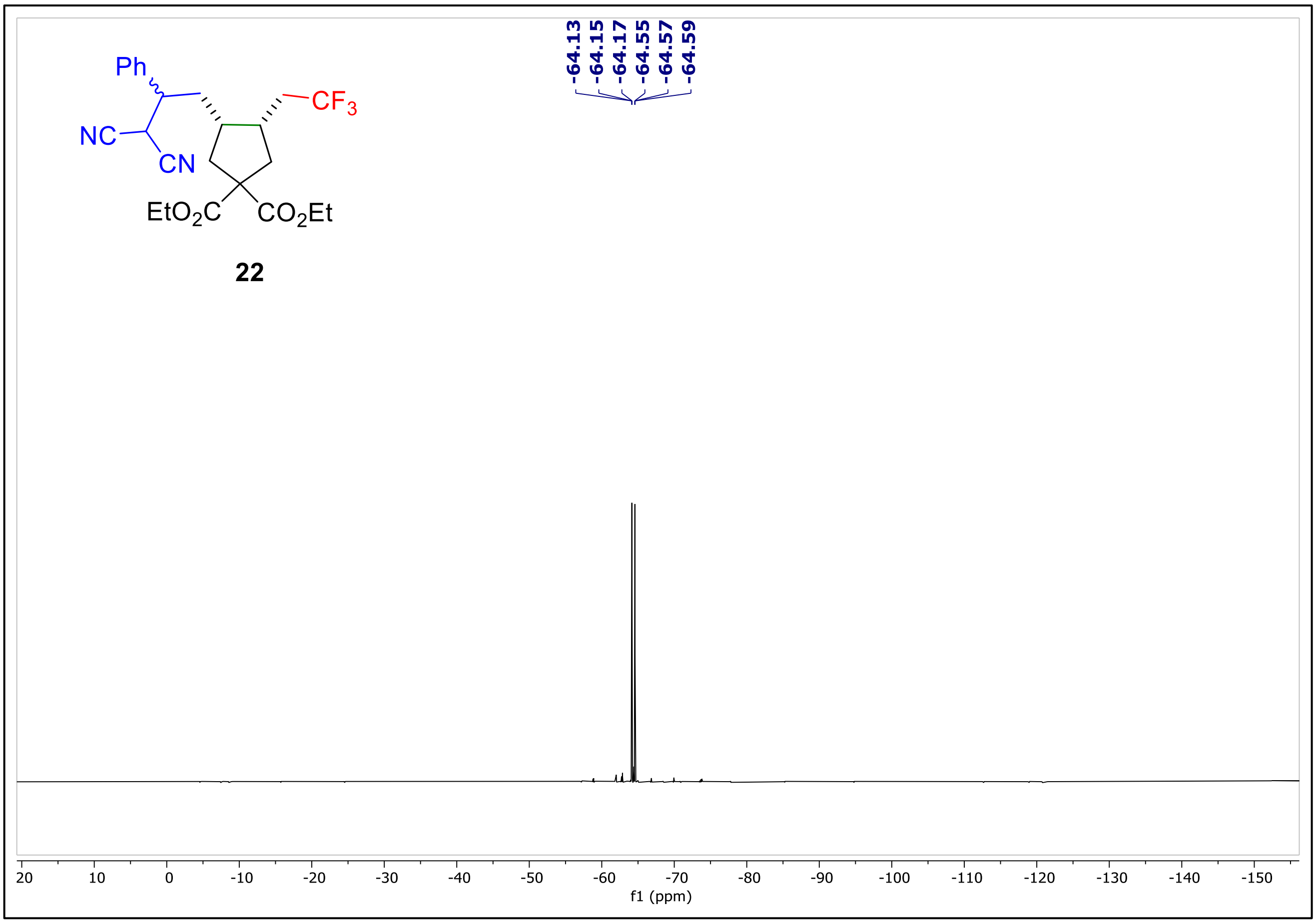

^{19}F NMR of compound 22 (471 MHz, $CDCl_3$)

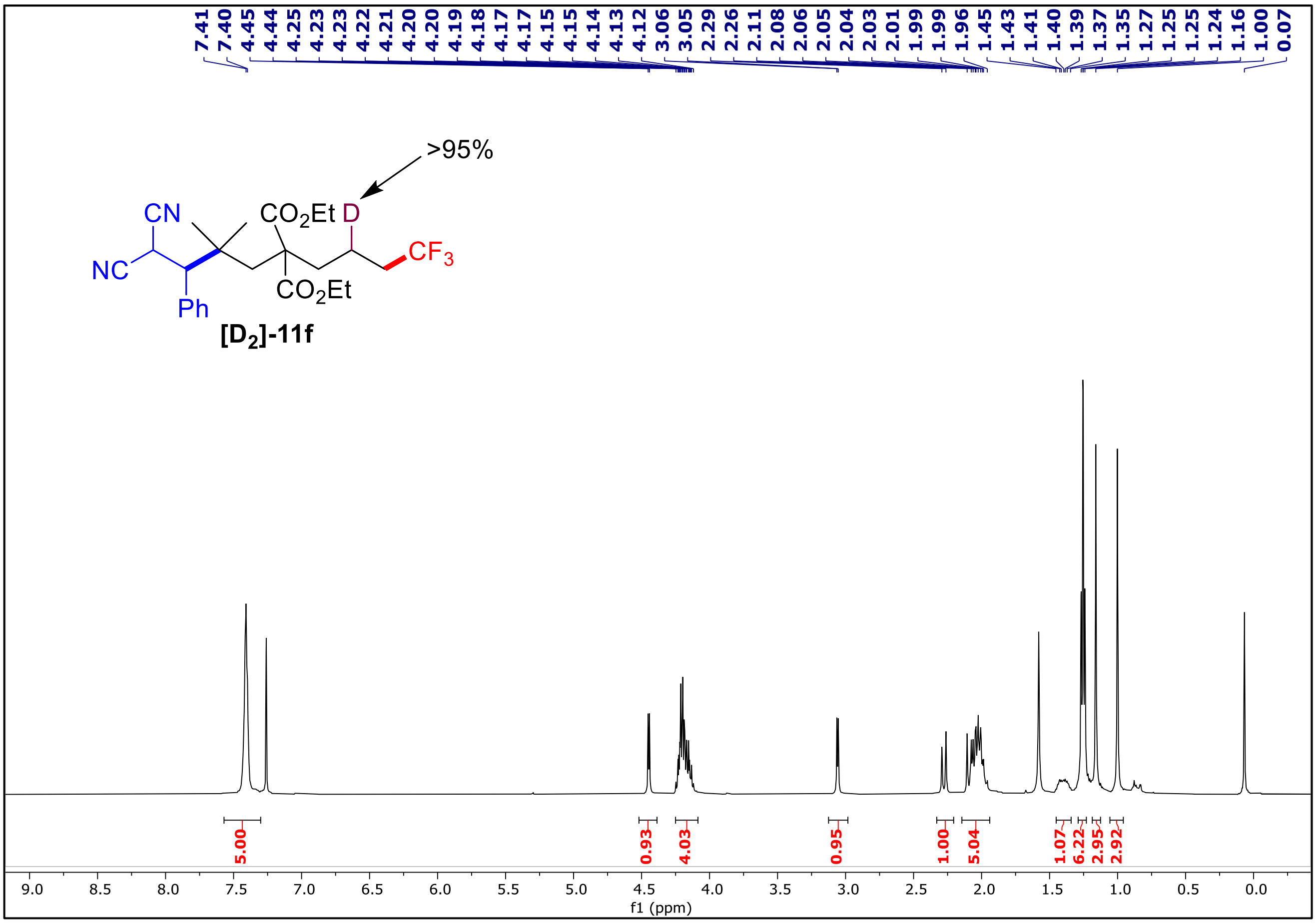

^{1}H NMR of compound **$[D_2]$-11f** (500 MHz, $CDCl_3$)

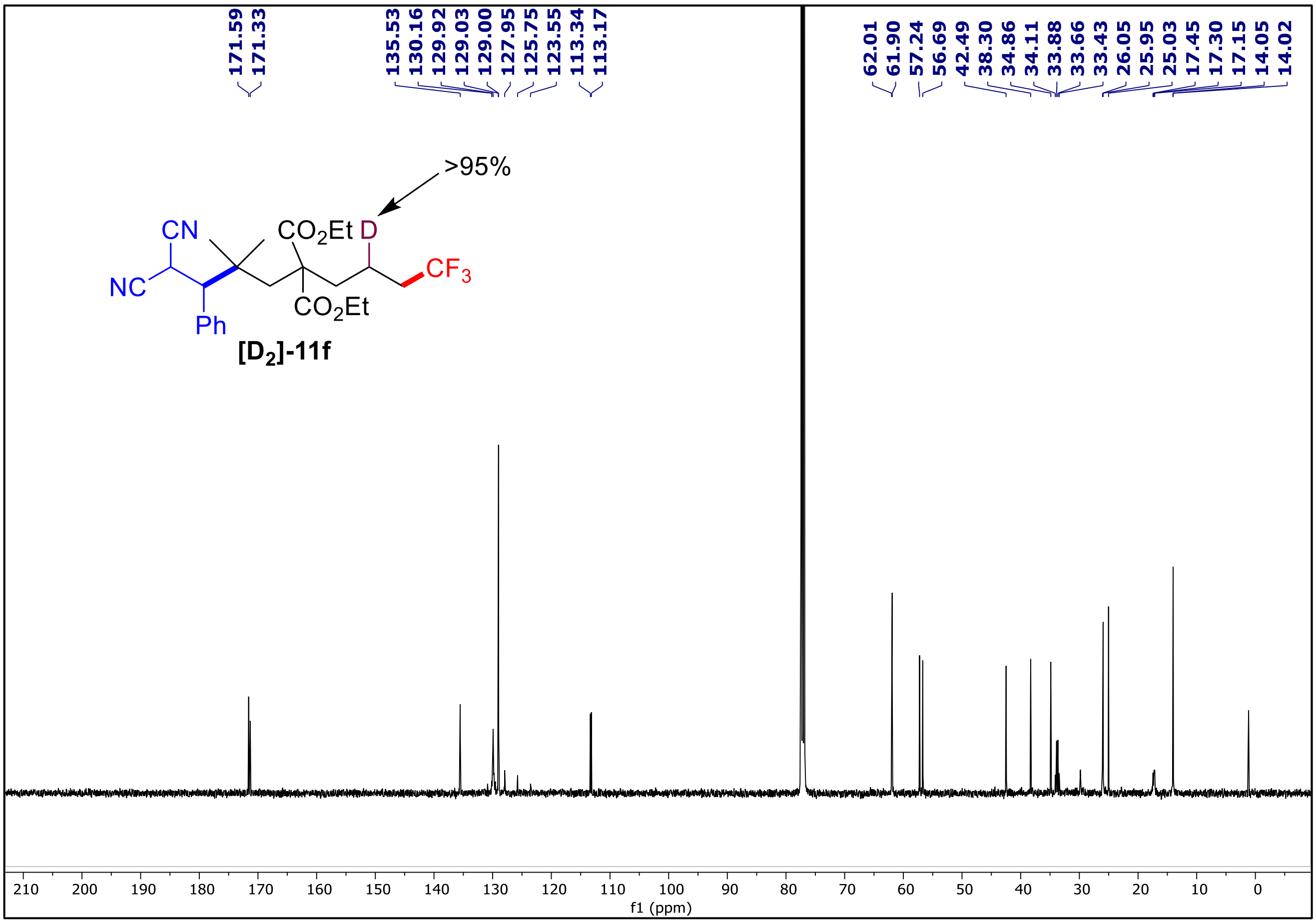

$^{13}C\{^{1}H\}$ NMR of compound **[D$_2$]-11f** (126 MHz, $CDCl_3$)

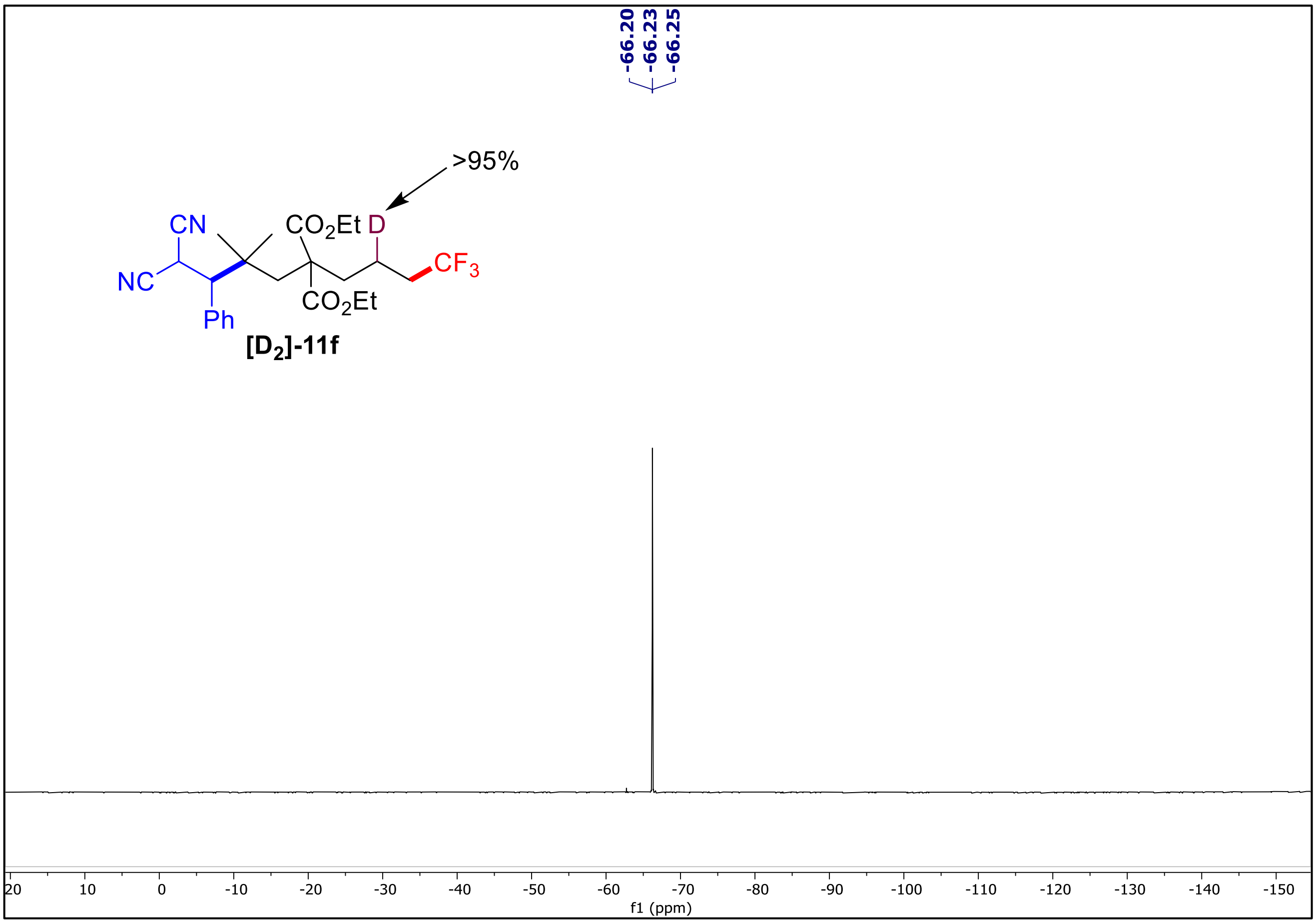

^{19}F NMR of compound **[D$_2$]-11f** (471 MHz, $CDCl_3$)
